# Differentiated embryo chondrocyte plays a crucial role in DNA damage response via transcriptional regulation under hypoxic conditions

**DOI:** 10.1371/journal.pone.0192136

**Published:** 2018-02-21

**Authors:** Hideaki Nakamura, Hidemasa Bono, Keiko Hiyama, Takeshi Kawamoto, Yukio Kato, Takeshi Nakanishi, Masahiko Nishiyama, Eiso Hiyama, Nobuyuki Hirohashi, Eisaburo Sueoka, Lorenz Poellinger, Keiji Tanimoto

**Affiliations:** 1 Department of Radiation Medicine, Research Institute for Radiation Biology and Medicine, Hiroshima University, Hiroshima, Japan; 2 Natural Science Center for Basic Research and Development, Hiroshima University, Hiroshima, Japan; 3 Department of Laboratory Medicine, Saga University Hospital, Saga, Japan; 4 Cell and Molecular Biology, Medical Nobel Institute, Karolinska Institutet, Stockholm, Sweden; 5 Database Center for Life Science, Joint Support-Center for Data Science Research, Research Organization of Information and Systems, Mishima, Japan; 6 Department of Dental and Medical Biochemistry, Hiroshima University, Hiroshima, Japan; 7 Nippon Kayaku Co., Ltd., Tokyo, Japan; 8 Department of Molecular Pharmacology and Oncology, Gunma University Graduate School of Medicine, Gunma, Japan; University College Dublin, IRELAND

## Abstract

Tumor hypoxia contributes to a biologically aggressive phenotype and therapeutic resistance. Recent studies have revealed that hypoxia reduces expression of several DNA damage recognition and repair (DRR) genes *via* both hypoxia-inducible factor (HIF)-independent and -dependent pathways, and this induced genomic instability in cancer cells. We show here that one of the HIF-target genes—differentiated embryo chondrocyte (DEC)—plays a role in DNA damage response *via* transcriptional repression. Comprehensive gene expression and database analyses have revealed systemic repression of DNA-DRR genes in cancer and non-cancer cells under hypoxic conditions. Hypoxic repression in typical cases was confirmed by quantitative RT-PCR and promoter reporter experiments, and knockdown experiments indicated the critical role of DEC2 in such repression. Assessment of histone H2AX phosphorylation revealed that recognition and repair of DNA double-strand breaks (DSBs) induced by bleomycin or γ-ray irradiation were attenuated; moreover, Cleaved Caspase-3 levels were decreased with pre-conditioning under hypoxia: opposing phenomena were ascertained by knockdown of *DEC2*. Finally, pre-conditioning under hypoxia decreased the sensitivity of cancer cells to DSBs, and knockdown of *DEC2* increased γ-ray sensitivity. These data imply that a critical reduction of DNA-DRR occurs *via* DEC-dependent transcriptional repression and suggest that DEC is a potential molecular target for anti-cancer strategies.

## Introduction

Hypoxia is one of the hallmarks of growing solid tumors. The tumor microenvironment is now known to play a critical role in the development of biologically aggressive tumor phenotypes, and also their resistance to chemo- and radio-therapy, through altered expression of various genes [1–4]. Hypoxia-inducible factor-1α (HIF-1α) is a key transcription factor that controls dozens of target genes in a variety of cancers [5–9]. Among the protein products of these genes, differentiated embryo chondrocyte (DEC) 1 and 2 have been reported to regulate expression of *PPARG*, *PER*, *STAT1*, *SREBF1*, *MITF*, *BHLHE40*, and *BHLHE41* in adipogenesis, circadian rhythm, immune system function, cell differentiation, and carcinogenesis [10–14]. Previously, we and others reported that the HIF-DEC signaling pathway -whereby HIF-1 induces DEC1 (also called BHLHE40, STRA13, or SHARP2) and DEC2 (also called BHLHE41 or SHARP1)- plays an important role in transcriptional repression of the mismatch repair gene *MLH1* under hypoxic conditions [15, 16].

Repressed DNA repair functions and mutator phenotypes under hypoxic conditions were first documented some time ago, but details of molecular mechanisms were not clear [17–20]. Recent publications have shown that expression of several DNA damage recognition and repair (DRR) genes -*MLH1*, *RAD51*, *BRCA1*, *MSH2* and *NBS1*-decreases under hypoxic conditions *via* HIF-1-dependent and -independent mechanisms, and that this repression of DNA-DRR leads to genomic instability, carcinogenesis, tumor progression, and treatment failure [21–27]. On the other hand, the precise functional role of the HIF-DEC signaling pathway, especially in hypoxic repression of DNA-DRR gene expression, is not yet fully understood.

In this study, we examined changes in the expression of numerous genes related to DNA-DRR and also the molecular mechanisms of transcriptional regulation under hypoxic conditions, by focusing on the HIF-1-induced transcriptional repressors DEC1 and DEC2 and the influence on DNA damage of cultivation under hypoxic conditions. We demonstrate for the first time that DEC1 and DEC2 are involved in the transcriptional repression of many DNA-DRR genes under hypoxic conditions. In addition, the HIF-DEC signaling pathway impaired the recognition and repair of double-strand breaks (DSBs)—as well as apoptosis—caused by various kinds of DNA-damage-inducing treatments *via* effective repression of transcription in cells subjected to hypoxia. To understand the mechanisms by which malignant phenotypes and genomic instability are acquired in cells exposed to low oxygen is important for developing new diagnostic and therapeutic targets.

## Materials and methods

### Chemicals

Most of chemicals were analytical grade and were purchased from Wako Pure Chemicals (Osaka, Japan) or Sigma (St. Louis, MO). HIF-prolyl hydroxylase inhibitors, FG-4592 and DMOG, were purchased from AdooQ BioScience (Irvine, CA). Bleomycin was kindly provided from Nippon Kayaku Co Company.

### Cell lines and treatment

The oral squamous cell carcinoma cell line, HSC-2, and a human hepatoma cell line, HepG2 (The Japanese Cancer Research Resource Bank), were cultured in DMEM or RPMI 1640 medium (Sigma-Aldrich Japan, Tokyo, Japan) supplemented with 10% fetal bovine serum (BioWhittaker, Verviers, Belgium) and Kanamycin (100 μg/ml). For gene expression analyses, cells were cultured under normoxic (21% pO_2_) or hypoxic (1% pO_2_) conditions for the indicated length of time in a hypoxic chamber. For knockdown analyses, siRNA against *HIF1A*, *DEC1*, *DEC2*, or non-specific siRNA (QIAGEN, Inc., Valencia, CA) were transfected with TransIT^®^-siQUEST™ Transfection Reagent (Mirus Corporation, Madison, WI) in HSC-2 for 12 hours. Then the cells were incubated under normoxic or hypoxic conditions for 24 hours, and cells were then harvested and stored at -80°C until use.

### RNA preparation

Total RNA was prepared from frozen cell pellets with a QIAGEN RNeasy^®^ mini kit (QIAGEN) according to the manufacturer’s instructions.

### Oligonucleotide array analysis

The CodeLink Expression Bioarray System (GE Healthcare, Tokyo, Japan) was used as previously described [28]. Briefly, from 1 μg of total RNA, double-strand cDNA and subsequent biotin-labeled cRNA were synthesized using a CodeLink^TM^ Expression Assay Reagent Kit (GE Healthcare). Ten μg of fragmented cRNA was hybridized to a CodeLink^TM^ Uniset Human 20 K I Bioarray (19,981 probes). After hybridization, the arrays were scanned with an Agilent DNA Microarray Scanner^TM^ (Agilent, Santa Clara, CA), then analyzed with CodeLink^TM^ Expression Analysis Software (GE Healthcare). Expression levels were normalized to the median of the entire spot array using GeneSpring^TM^ GX (Agilent Technologies). The data were submitted to the Gene Expression Omnibus (GSE52795). Gene-annotation enrichment analysis was performed using DAVID Bioinformatics Resources 6.8 (http://david.ncifcrf.gov/home.jsp)).

### Database analyses

The NCBI Gene Expression Omnibus (GEO) [29] database now stores over 2 million records of samples, so we attempted a collective-intelligence approach to assess gene expression data related to hypoxic responses in order to identify hypoxia-related genes in a data-driven way. To normalize the microarray data, we used data from the Affymetrix Human Genome U133 Plus 2.0 Array (GEO accession: GPL570), which is the most often used platform in human studies, and limited our search to “human”. The pair of experiments performed in normoxic or hypoxic conditions was undertaken after careful curation of dataset descriptions and corresponding papers in the citation metadata. As a result, we identified 29 hypoxia-normoxia pairs from among 9 data series (GEO accessions: GSE19197, GSE29641, GSE4086, GSE9234, GSE39042, GSE58049, GSE52315, GSE46054, and GSE53012). After Robust Multichip Average (RMA) normalization [30] by the affy package [31] in R/BioConductor (version 2.15.0), up/down regulated genes were selected from all pairs with a 1.5-fold threshold. The number of experiments in which the gene was up- or down-regulated was counted for all genes.

### Reverse Transcription-PCR

Two μg of total RNA extracted from each cell line were reverse-transcribed using a High-Capacity cDNA Archive^TM^ Kit (Applied Biosystems, Foster City, CA). A two-hundredth aliquot of the cDNA was subjected to real-time RT-PCR using primers (final concentration of 200 nM each) and MGB probe (final concentration of 100 nM, the Universal Probe Library: UPL, Roche Diagnostics, Tokyo, Japan) (shown in S1 Table) sets for *HIF1A*, *DEC1*, and *DEC2*, or the TaqMan^TM^ Gene Expression Assay (Applied Biosystems) for *MLH1*, *MSH2*, *MBD4*, *MRE11A*, *BRCA1*, and *RAD51*, with Pre-Developed TaqMan^TM^ Assay Reagents (Applied Biosystems) for *ACTB* as an internal control. PCR reactions were carried out using 7500 Real-Time PCR System (Applied Biosystems) under the following standard conditions. At least three independent measurements were averaged, and relative gene expression levels were calculated using *ACTB* expression as the denominator for each cell line.

### Plasmid constructions

The DNA fragments (nucleotide positions from -1560 to +99, where the transcriptional start site is designated as +1) including the 5’ region of *MSH2*, -1629 ~ +173 of *MBD4*, -845 ~ +20 of *MRE11A*, -224 ~ +26 of *BRCA1*, and -1345 ~ +517 of *RAD51*, were amplified using PCR from HepG2 genomic DNA and subcloned into luciferase reporter plasmid pGL3-Basic^®^ (Promega, Madison, WI). All reporters were confirmed by sequencing analysis. Details of expression plasmid vectors of DEC1, Δbasic DEC1, DEC2, and luciferase reporter (pGL3-MLH1 Pro) were as previously described [15, 32].

### Luciferase reporter assay

Transient transfection was performed as follows. Each pGL3 promoter reporter with pcDNA-FLAG, p3xFLAG-CMV-DEC1, p3xFLAG-CMV-Δbasic DEC1, or pcDNA-FLAG-DEC2, was mixed with Trans-IT LT1^®^ Transfection Reagent (Mirus). Renilla-luciferase vector (pRL-SV40) (Promega) was used as a transfection efficacy control. After transfection, cells were incubated under normoxic or hypoxic conditions for 36 hours. Luciferase luminescence was measured as previously described [15, 33].

### Bleomycin or γ-ray treatment

Cells were cultured under normoxic or hypoxic conditions for 24 hours until just before treatment. Various doses of bleomycin were applied under normoxic conditions and cytotoxicity was evaluated by conventional MTT dye-reduction assay as previously described [34]. The indicated doses of ^137^Cs γ-ray irradiation were applied with a Gammacell 40 Exactor (MDS Nordion, Ottawa, ON, Canada) under normoxic conditions. Bleomycin or γ-ray treated cells were further incubated until the indicated time points and then harvested for analysis. IC_50_ value or relative MTT tetrazolium reduction activity (relative viability) was calculated using the value at 24 hours as the denominator.

### Immunoblot analysis

For protein-expression analysis, whole-cell extracts were prepared from cultured cells with or without various treatments, as previously described [35]. Twenty-five or fifty μg of extract was blotted onto a PVDF filter following SDS-polyacrylamide gel electrophoresis. Anti-H2AX, anti-γH2AX (Millipore, Temecula, CA), anti-Caspase3, anti-Cleaved Caspase-3 (Cell Signaling Technology, Danvers, MA), anti-HIF-1α (BD Pharmingen, San Diego, CA), anti-DEC1 (Novus Biologicals, Littleton, CO), anti-DEC2 (invitrogen, Rockford, IL), and anti-β-actin (Sigma) were used as primary antibodies. A mouse or rabbit anti-IgG horseradish peroxidase conjugate (Amersham Life Science) was used as a secondary antibody. Immunocomplexes were visualized with the enhanced chemiluminescence reagent ECL Plus (Amersham Life Science). Expression levels of γH2AX or Cleaved Caspase-3 were quantified by measuring densities using ImageJ (public domain open source software; imagej.nih.gov), and at least three independent measurements were averaged. Relative expression levels of γH2AX or Cleaved Caspase-3 were calculated using expression levels of H2AX or Caspase-3 as the denominator for each time point.

### Immunostaining analysis

HepG2 cells were cultured on cover slips under normoxic or hypoxic conditions for 24 hours, and re-oxygenated for 30 minutes. The cells were then exposed to 1 Gy of X-ray, and further cultured for 0.5 and 12 hours under normoxic condition after irradiation. The cells on cover slips were fixed with 4% Paraformaldehyde, permeabilized with 0.5% Triton X-100, and stained with anti-γH2AX (1:1000) as primary antibody. And rhodamine-conjugated sheep anti-mouse immunoglobulin G (IgG) (1:1000) (Chemicon, Temecula, CA) was used as secondary antibody. After several washes, nuclei were stained with Hoechst 33342. Subcellular distributions of γH2AX foci were observed, and pictures were taken using a fluorescence microscope (BZ-8000; KEYENCE Corporation, Osaka, Japan). For each condition, γH2AX foci in 8–25 cells were counted and averages per cell were calculated by authors.

### Statistical analysis

Statistical tests were performed based on the ANOVA test, Student’s *t* test, Dunnett’s test, or Turkey-Kramer HSD test using StatView^®^ version 5.0 software (SAS Institute Inc., Cary, NC, USA).

## Results

### Hypoxia systemically down-regulates the expression of DNA-DRR genes *via* DEC

**Fig 1:**
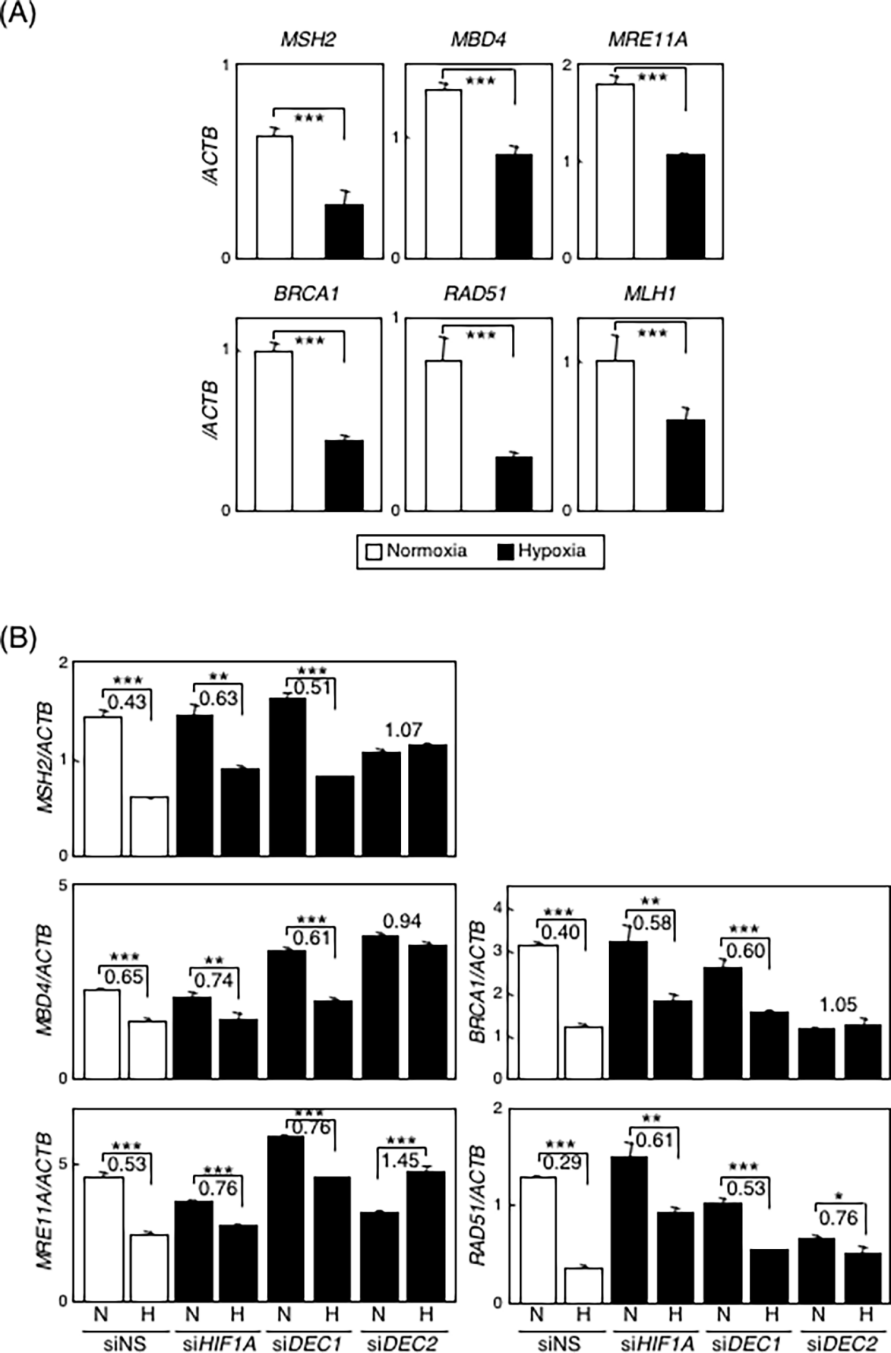
Expressions of selected genes related to DNA-DRR were quantitatively decreased under hypoxic conditions through the HIF-1-DEC pathway. (A) HSC-2 cells were incubated under 21% pO_2_ (normoxia) or 1% pO_2_ (hypoxia) for 24 hours. Expression levels of *MSH2*, *MBD4*, *MRE11A*, *BRCA1*, *RAD51*, and *MLH1* were analyzed by real-time RT-PCR. Relative mRNA levels were calculated as the ratio to that of *ACTB*. (B) Knockdown assays for *HIF1A*, *DEC1*, and *DEC2* were performed using HSC-2 cells. Non-specific siRNA (siNS) or targeted siRNA was transfected into HSC-2 and cultured under normoxic or hypoxic conditions for 24 hours. Expression levels of *MSH2*, *MBD4*, *MRE11A*, *BRCA1*, and *RAD51* were evaluated by real-time RT-PCR. Relative mRNA levels were calculated as the ratio to that of *ACTB*. To compare the effect of knockdown of *HIF1A*, *DEC1*, or *DEC2*, we calculated the ratio of expression under hypoxic conditions compared with that under normoxia; the value is noted over the bar. Columns are the mean of three independent experiments; bars, SD. *P* values calculated with Student’s *t* test are: *, *P* < 0.05; **, *P* < 0.01; ***, *P* < 0.001.

To discriminate gene expression profiles under hypoxic conditions, we performed comprehensive gene-expression analyses of HSC-2 cells cultured under normoxic or hypoxic conditions. Among 19,981 gene probes tested, 2216 were up-regulated (more than 2-fold) and 2488 were down-regulated (less than 2/3-fold) in cells cultured for 24 hours under hypoxic conditions, suggesting that hypoxic stress strongly affects gene expression. As we had expected, many known hypoxia-inducible genes were found among the up-regulated ones (S1 Fig), and subsequent gene-annotation enrichment analysis using DAVID Bioinformatics Resources 6.8 (http://david.ncifcrf.gov/home.jsp)) strongly supported our observation (S2 Table). Interestingly, DAVID also suggested that many genes responsible for DNA-DRR (more than 50 of 130 genes listed in http://sciencepark.mdanderson.org/labs/wood/DNA_Repair_Genes.html) were involved in down-regulated genes, suggesting the occurrence of systemic repression of DNA-damage response (S3 and S4 Tables, and S1 Fig). Analyses of the NCBI Gene Expression Omnibus (GEO) database further supported our observations. It indicated that hypoxic repression of DNA-DRR genes is common among various kinds of cancer and non-cancer cells (S5 and S6 Tables). One-hundred sixty genes in cancer cells and 123 genes in non-cancer cells among 176 genes analyzed were reported to be down-regulated under hypoxic conditions in at least one GEO data set. With some of the down-regulated genes (*MLH1*, *MSH2*, *MBD4*, *MRE11A*, *BRCA1*, and *RAD51*), we confirmed repression under hypoxic conditions through the use of quantitative real-time RT-PCR (Fig 1A). We also observed that expression levels of these genes were down-regulated by treatment with various HIF-activating reagents (S2 Fig). We further evaluated the role of HIF-1α-DEC pathway by using samples in which *HIF1A*, *DEC1*, or *DEC2* had been confirmed to be effectively knocked-down (S3 Fig). Comparing expression levels of these genes under normoxic and hypoxic conditions, all of them were significantly down-regulated under hypoxic conditions with non-specific siRNA transfection. Those repressions under hypoxic conditions were clearly prevented, i.e., significant fold-reduction disappeared, by knock-down of *DEC2* with specific siRNA (siDEC2) transfection, whereas siDEC1 and siHIF1A showed only slight effects, as previously reported [15]. In fact, expression of *MRE11A* under hypoxic conditions increased with *DEC2* knock-down (Fig 1B).

### DEC represses promoter activities of DNA-DRR genes

**Fig 2:**
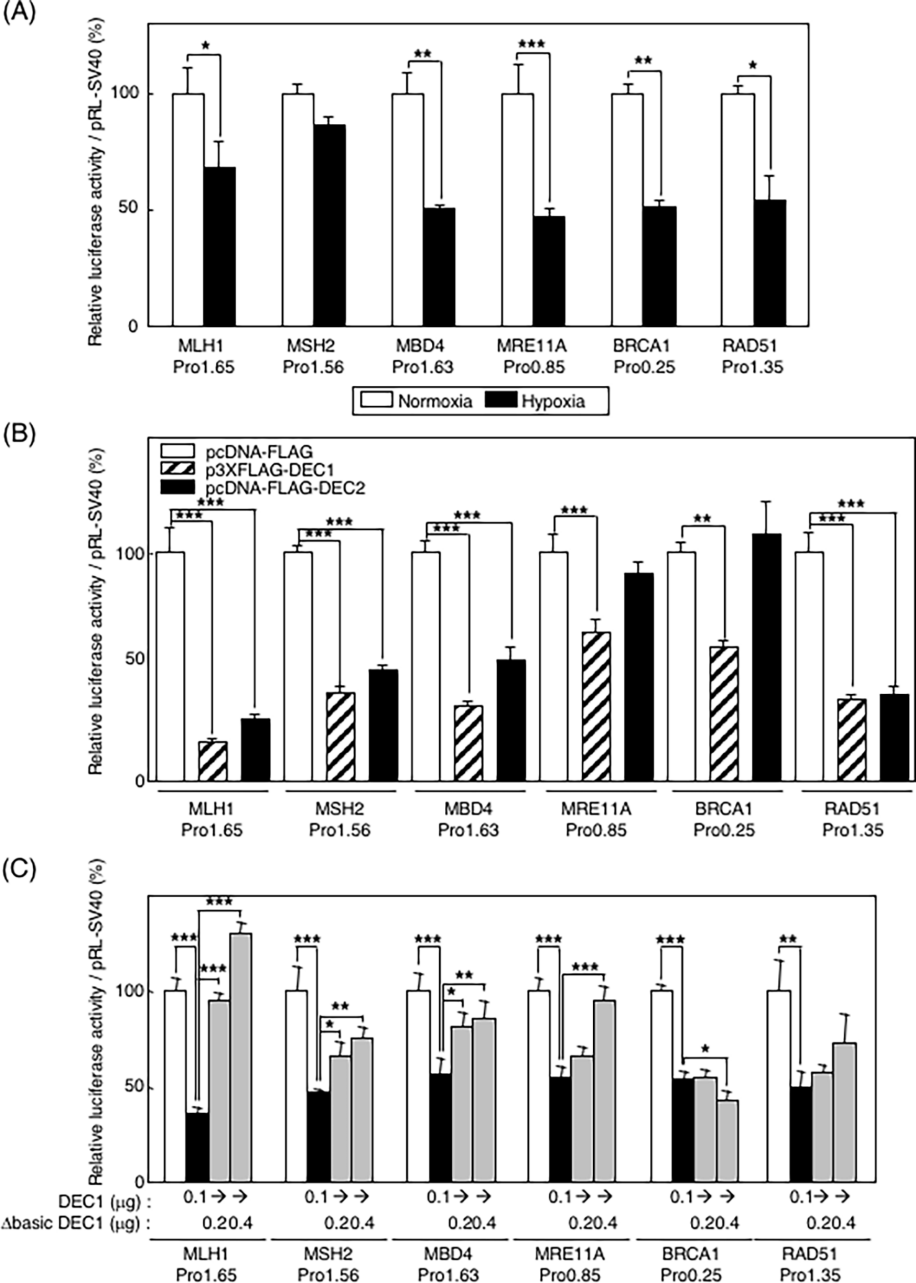
Promoter activities of DNA-DRR genes decreased with DEC. (A) The approximately 200–1600bp length of 5’ region from the transcriptional start site of human *MLH1*, *MSH2*, *MBD4*, *MRE11A*, *BRCA1*, and *RAD51* were subcloned into a luciferase reporter plasmid, and these were designated as pGL3-reporters. Each reporter plasmid was transfected into HepG2, and incubated for 36 hours under normoxic or hypoxic conditions. Luciferase activities were determined with a dual-luciferase assay system. (B) Each reporter and DEC1 or DEC2 expression plasmid were co-transfected into HepG2, and incubated for 36 hours under normoxic conditions. Luciferase activities were determined with a dual-luciferase assay system. (C) Each reporter and DEC1 or Δbasic DEC1 expression plasmid were co-transfected into HepG2, and incubated for 36 hours under normoxic conditions. Luciferase activities were determined with a dual-luciferase assay system. Columns are the mean of three independent experiments; bars, SD. The differences between means were significant (ANOVA *P* < 0.005). *P* values calculated with Dunnett’s test are: *, *P* < 0.05; **, *P* < 0.01; ***, *P* < 0.001.

To clarify the molecular mechanisms of the hypoxic repression of DNA-DRR genes, we subcloned the 5’ regions of human *MLH1*, *MSH2*, *MBD4*, *MRE11A*, *BRCA1*, and *RAD51* (approximately 200–1600 bp length from the transcriptional start site of each gene) into a luciferase reporter plasmid, pGL3-Basic vector. It is notable that all of promoter reporters showed significant luciferase activities under normoxic condition, and those activities were suppressed under hypoxic conditions, except for *MSH2* promoter (Fig 2A). Co-transfection experiments demonstrated that DEC1 significantly repressed all promoter activities tested here. DEC2 also effectively repressed all promoter activities except for those of *MRE11A* and *BRCA1* (Fig 2B). We further performed co-transfection experiments using the promoter reporters, DEC1, and a dominant-negative form of DEC1 that lacks a DNA-binding domain (Δbasic DEC1) [32]. With forced expression, the dominant-negative form of DEC1 acted against the repression activity of DEC1 on the promoters of *MLH1*, *MSH2*, *MBD4*, *MRE11A*, but not on the promotor of *BRCA1*. (Fig 2C)

### Pre-conditioning under hypoxia decreased cellular sensitivity to bleomycin or γ-ray treatment

**Fig 3:**
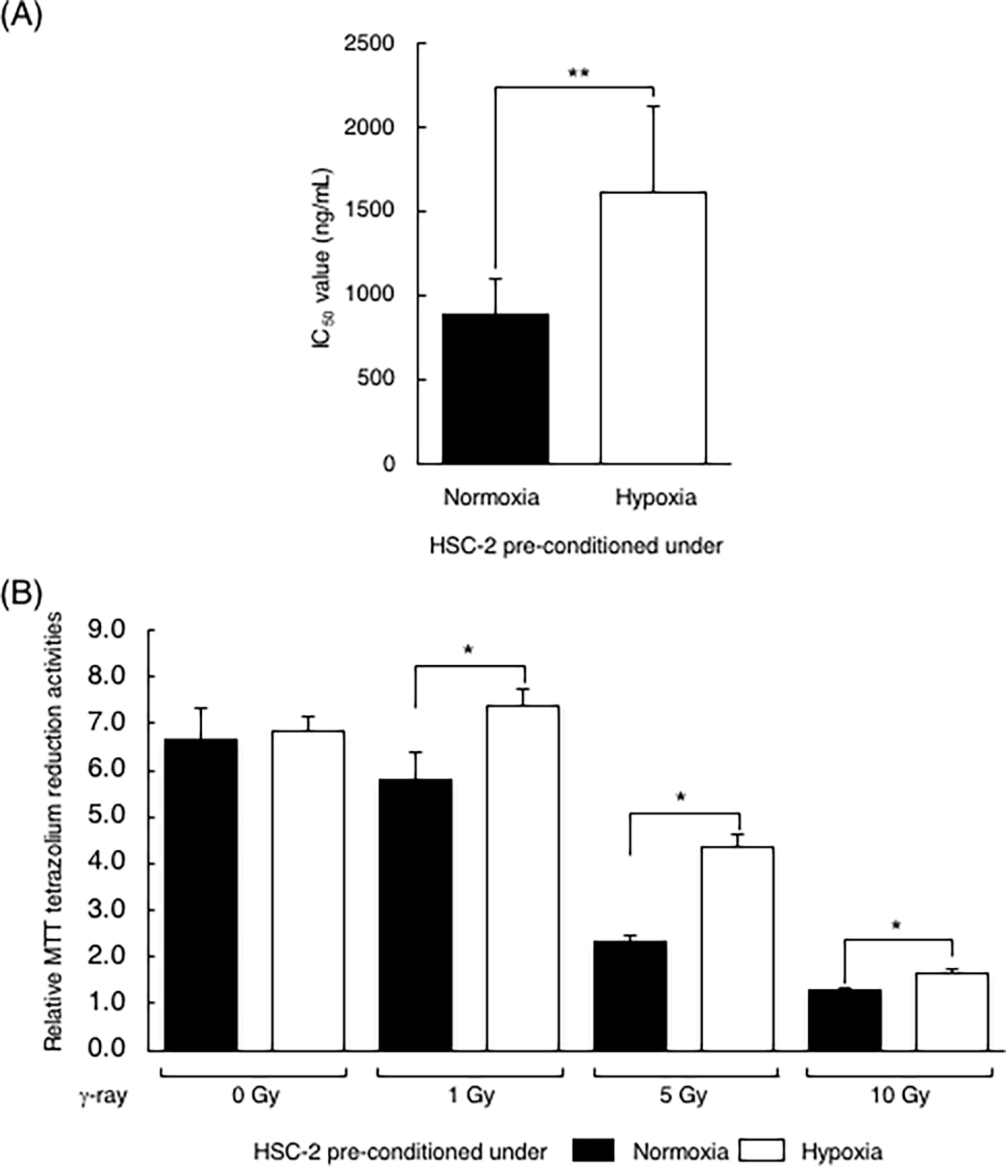
Pre-conditioning under hypoxia decreased cellular sensitivity to treatment, inducing DNA double-strand breaks. (A) Cellular viability after bleomycin treatment in HSC-2, with or without pre-conditioning under hypoxia, were assessed using the MTT assay. IC_50_ value was calculated in order to compare cellular sensitivity to bleomycin. (B) Cellular viability after γ-ray irradiation in HSC-2, with or without pre-conditioning under hypoxia, were assessed with the MTT assay. HSC-2 cells were incubated under normoxic or hypoxic conditions for 24 hours before irradiation. After re-oxygenation, cells were irradiated with the indicated doses. After irradiation, the cells were cultured under normoxic conditions for 96 hours and cellular viabilities were evaluated with the MTT assay. Relative MTT tetrazolium reduction activities (relative viabilities) were calculated as the ratio to the value at 24 hours. Columns are the mean of three independent experiments; bars, SD. *P* values calculated with Student’s *t* test were: *, *P* < 0.05; **, *P* < 0.01.

To clarify the effects of hypoxic repression of DNA-DRR genes in response to DNA damage, we first assessed, using MTT assay, the viability of cancer cells pre-conditioned under hypoxia. To observe cellular responses to Bleomycin or γ-ray treatment (which induces DSBs effectively [36]) independently of hypoxia, HSC-2 cells were incubated under hypoxic conditions for 24 hours, and re-oxygenated just before treatment. MTT assays performed after 72 hours of continuous treatment with bleomycin (at various concentrations) demonstrated an IC_50_ value that was significantly higher for cells pre-conditioned under hypoxia than for cells under normoxia (Fig 3A). Hypoxic pre-conditioned HSC-2 cells treated with three different doses of ^137^Cs γ-ray were also studied with the MTT assay to evaluate their viability after incubation for 96 hours; relative viability was calculated as the ratio of the value to that of the 24 hour-culture. In non-irradiated cells, no remarkable differences in relative cellular viability were observed between normoxic and hypoxic pre-conditioned cells, implying that viability is not affected by pre-conditioning under hypoxia for 24 hours. On the other hand, among γ-ray irradiated cells, viability of hypoxic pre-conditioned cells was significantly higher than that of normoxic cultured cells. Although overall viability was lowest following 10 Gy treatment, the most remarkable difference between normoxic and hypoxic pre-conditioning was observed following 5 Gy irradiation (Fig 3B).

### Recognition and repair of DSBs were attenuated in cells pre-conditioned under hypoxia

**Fig 4:**
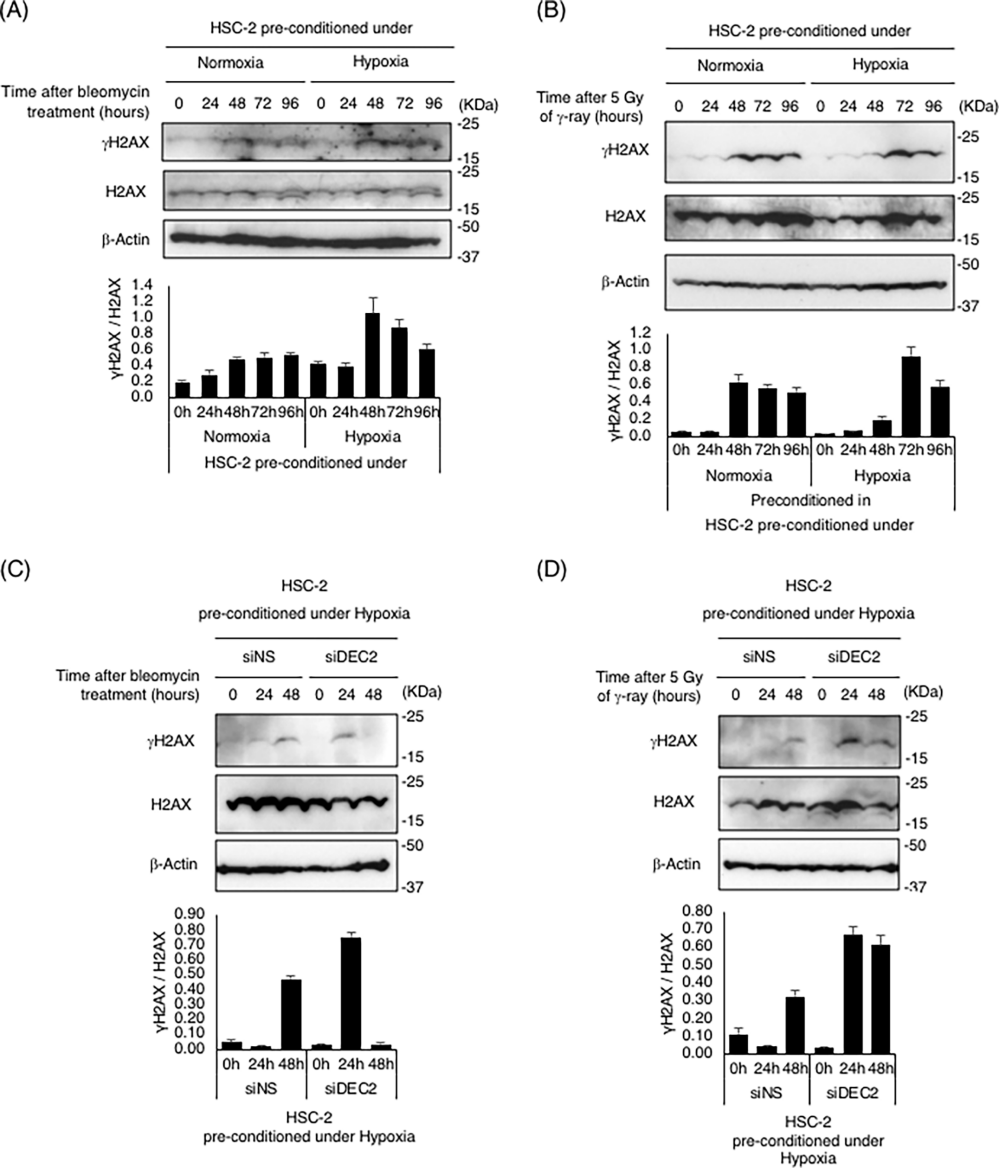
Recognition and repair of DNA double-strand breaks were inhibited *via* DEC2 in hypoxic pre-conditioned cells. Phosphorylate histone H2AX (γH2AX) levels after bleomycin or irradiation treatment in HSC-2 cells with or without pre-conditioning under hypoxia were evaluated with immunoblotting analysis. (A), (B) HSC-2 cells were cultured under normoxic or hypoxic conditions for 24 hours; afterwards, the cells were treated with 500 ng/mL of bleomycin or 5 Gy of γ-ray irradiation, and then the cells were cultured for the indicated lengths of time under normoxic conditions. (C), (D) HSC-2 cells were transfected with either non-specific (siNS) or DEC2-specific (siDEC2) siRNA, and then incubated under hypoxia for 24 hours before irradiation. After re-oxygenation, cells were treated with 500 ng/mL of bleomycin or 5 Gy of γ-ray irradiation. Thereafter, the cells were cultured for the indicated length of time under normoxic conditions. Anti-γH2AX, anti-H2AX and anti-β-actin were used for specific detection of each protein, and representative images are shown. Relative expression levels of γH2AX were calculated with H2AX expression as the denominator for each time point, and are shown in bottom panels. Columns are the mean of three independent experiments; bars, SD.

To clarify the molecular mechanisms of decreased sensitivity to DSB-inducing treatments in cells pre-conditioned under hypoxia, we evaluated the phosphorylation status of histone H2AX (γH2AX) in the cells as a DSB marker [37, 38]. HSC-2 cells were incubated under hypoxic conditions for 24 hours, and then treated with bleomycin for the indicated periods under normoxic conditions. Immunoblotting analyses confirmed that γH2AX level started to increase at 24 hours, and the level continued elevating until 48 hours after bleomycin treatment. After 48 hours, the level started to decrease or remained at a low level, indicating that DSB repair progressed successfully in normoxic cells (Fig 4A). In contrast, the level of γH2AX in cells pre-conditioned under hypoxia increased at 48 hours and remained at a high level, although it eventually started to decrease (Fig 4A). Similar results were obtained following irradiation with 5Gy of γ-ray. In normoxic cells, the level of γH2AX increased at 48 hours after irradiation treatment, and then started to decrease. In pre-conditioned cells, γH2AX had slightly increased at 48 hours, and it peaked at 72 hours after irradiation treatment (Fig 4B). Similar kinetics of γH2AX were also observed in 1 Gy of X-ray irradiated HepG2 cells by immunostaining analysis (S4 Fig). To clarify the role of DEC2 in this process, we performed knock-down experiments using siRNA for *DEC2*. Non-specific siRNA or siDEC2 was transiently transfected 24 hours before pre-conditioning under hypoxia, and after that we performed the foregoing analysis. In siNS transfected cells, the level of γH2AX could be slightly detected at 24 hours, and markedly detected at 48 hours after bleomycin treatment. On the other hand, γH2AX after bleomycin treatment in *DEC2* knocked-down cells increased at 24 hours (Fig 4C). Similar results were obtained following irradiation with 5Gy of γ-ray. In siNS transfected cells, the level of γH2AX was slightly detected at 48 hours, but markedly detected at 24 hours after γ-ray irradiation in *DEC2* knocked-down cells (Fig 4D). These data indicate that knock-down of *DEC2* restored radiation-induced DNA damage responses in hypoxic pre-conditioned cells.

### Hypoxic pre-conditioning altered both DNA damage response and subsequent apoptosis signaling *via* DEC2

**Fig 5:**
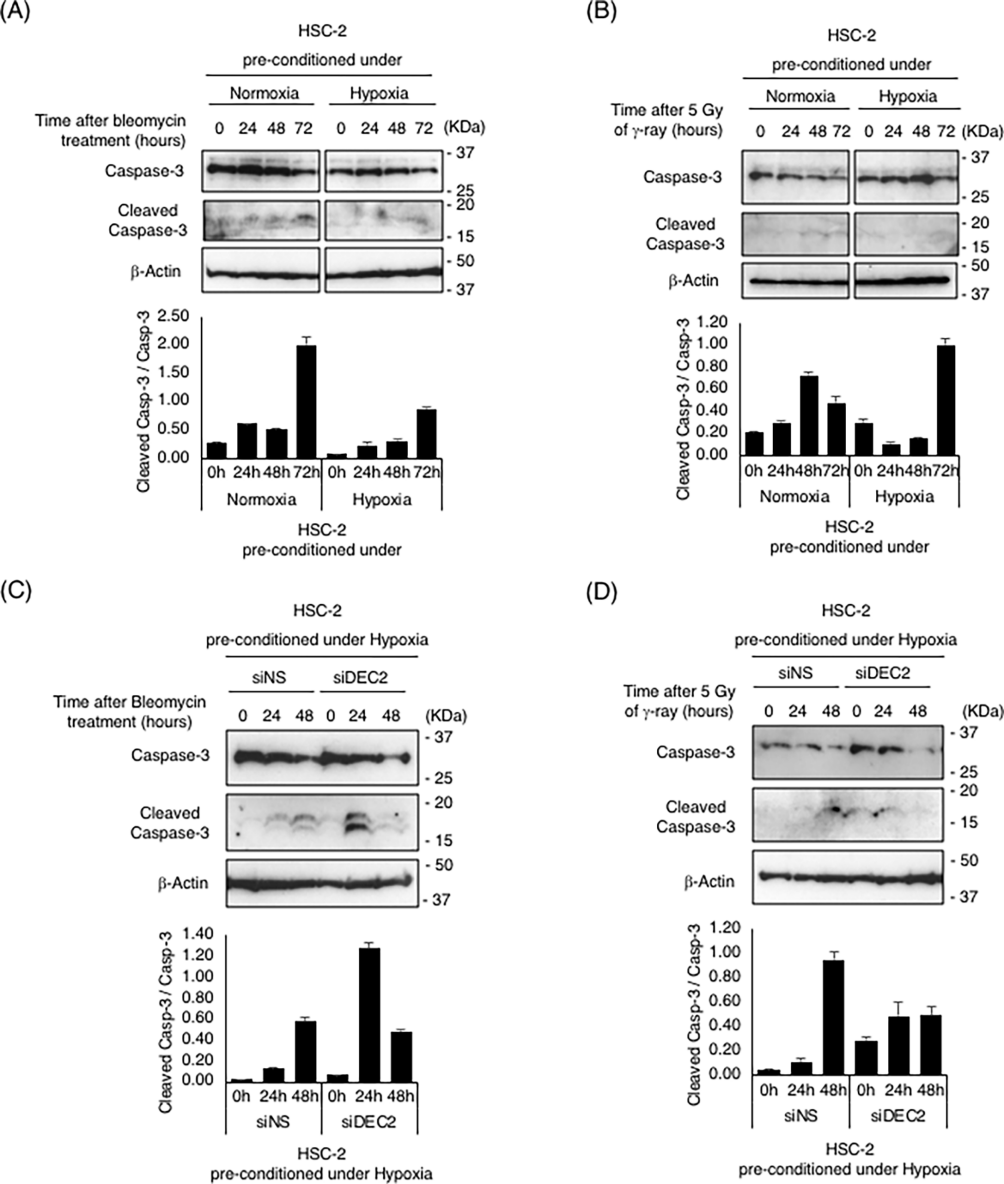
Cleaved Caspase-3 levels were reduced *via* DEC2 in hypoxic pre-conditioned cells. Total Caspase-3 or Cleaved Caspase-3 levels after bleomycin or γ-ray irradiation in HSC-2, with or without hypoxic pre-conditioning, was evaluated using imuunoblotting analysis. (A), (B) HSC-2 cells were cultured under normoxic or hypoxic conditions for 24 hours; afterwards, the cells were exposed to 500 ng/mL of bleomycin or 5 Gy of γ-ray irradiation. After that, the cells were cultured for the indicated length of time under normoxic conditions. (C), (D) siRNA was transiently transfected into cells 24 hours before hypoxic pre-conditioning. Transiently transfected cells were cultured in normoxic or hypoxic conditions for 24 hours. Thereafter, the cells were exposed to 500 ng/mL of bleomycin or 5 Gy of γ-ray irradiation. After that the cells were cultured for the indicated length of time under normoxic conditions. Anti-Caspase-3, anti-Cleaved Caspase-3, and anti-β-actin were used for specific detection of each protein, and representative images are shown. Relative expression levels of Cleaved Caspase-3 were calculated with Caspase-3 expression as the denominator for each time point, and are shown in bottom panels. Columns are the mean of three independent experiments; bars, SD.

To determine whether hypoxic treatment affects the apoptotic response to DNA damage, we evaluated levels of Cleaved Caspase-3 protein. HSC-2 cells were incubated under normoxic or hypoxic conditions for 24 hours, then treated with bleomycin or γ-ray as in the previous experiments. Immunoblotting analyses demonstrated that levels of full-length Caspase-3 protein increased at 24 hours after bleomycin treatment and then decreased until 72 hours in normoxic cells; a similar increase of full-length Caspase-3 protein was observed in hypoxic pre-conditioned cells, but with lower intensity. Whereas increased levels of Cleaved Caspase-3 were observed in normoxic cells, the increase was barely detectable in hypoxic pre-conditioned cells (Fig 5A). Similarly, increased levels of Cleaved Caspase-3 were observed in normoxic cells following to γ-ray irradiation, but the increase was detected only in hypoxic pre-conditioned cells at 72 hours after irradiation (Fig 5B). Knock-down experiments demonstrated that bleomycin-induced Cleaved Caspase-3 strongly increased at 24 hours after bleomycin treatment in *DEC2* inhibited cells (siDEC2), but it became much weaker at 24 hours and then increased until 48 hours (Fig 5C). Similar effects of DEC2 inhibition were observed in γ-ray irradiated cells (Fig 5D). These results suggest that pre-conditioning under hypoxia altered both DNA-damage response and subsequent apoptosis signaling *via* DEC2.

**Fig 6:**
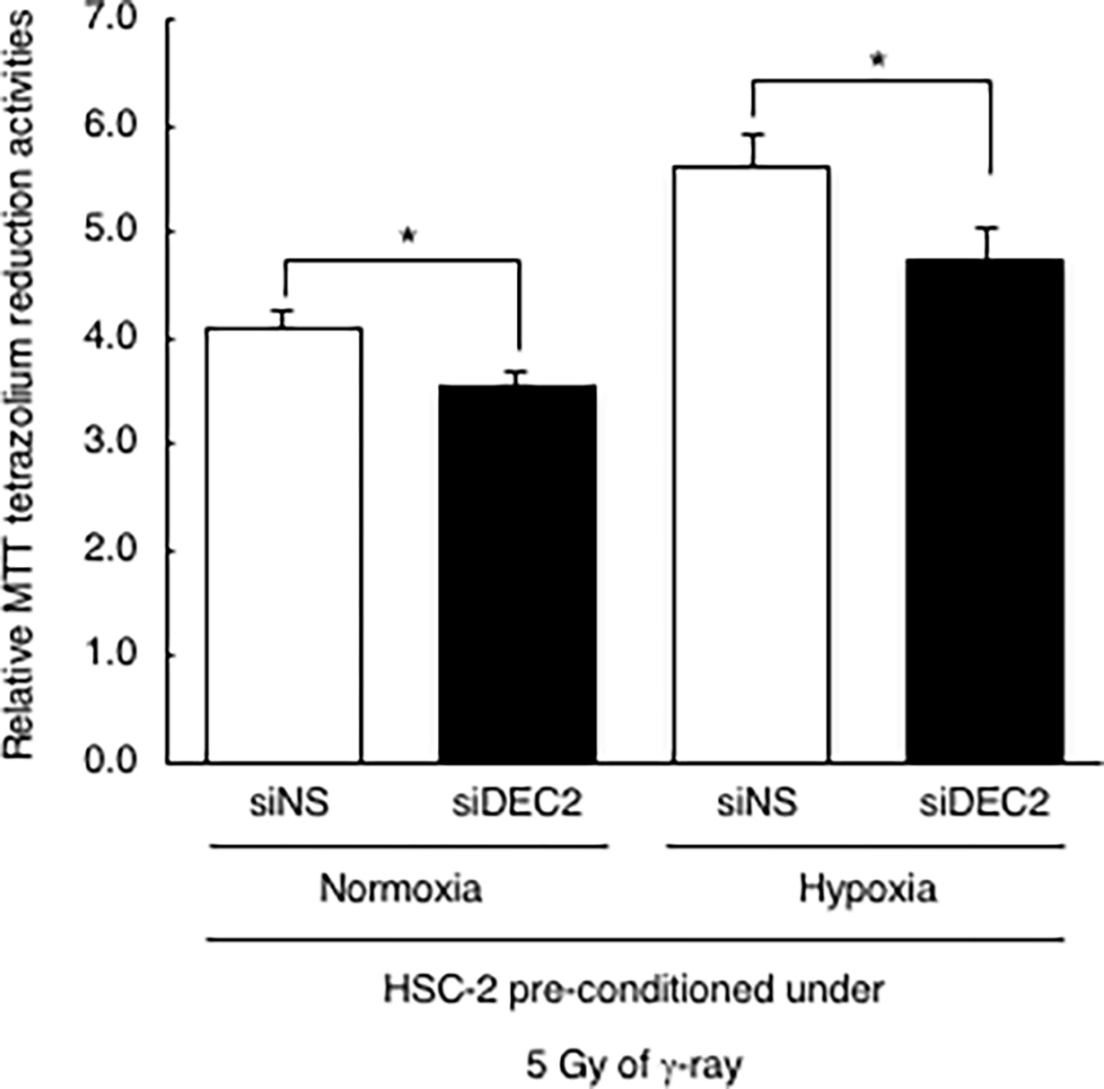
DEC2 decreased sensitivity to DNA damage in hypoxic pre-conditioned cells. Non-specific siRNA (siNS) or siDEC2 was transiently transfected into HSC-2 cells 24 hours before hypoxic pre-conditioning, and after that the cells were incubated under normoxic or hypoxic conditions for 24 hours before irradiation. After re-oxygenation, cells were irradiated with 5 Gy of γ-ray. After irradiation, the cells were cultured under normoxic conditions for 96 hours and cell viability was evaluated with the MTT assay. Relative MTT tetrazolium reduction activities (relative viabilities) were calculated as the ratio to the value at 24 hours. Columns are the mean of three independent experiments; bars, SD. *P* values calculated with Student’s *t* test were: *, *P* < 0.05.

Finally, we assessed the role of DEC2 in the decline of radiation sensitivity: Non-specific siRNA or siDEC2 was transiently transfected 24 hours before hypoxic pre-conditioning, and after that we performed the previous analysis. In siNS transfected cells, hypoxic pre-conditioning conferred resistance to 5 Gy of γ-ray as shown in Fig 3B. It was striking that siDEC2 transfected cells showed significantly lower viability in both normoxic and hypoxic pre-conditioned cells, but especially in hypoxic pre-conditioned cells (Fig 6). These data indicate that DEC2 plays a crucial role in sensitivity to treatments that induce DNA damage.

## Discussion

Under hypoxic conditions, the cellular DNA-repair function becomes impaired, which causes hyper-mutability after DNA damage [17, 39]. This suggests that tumor hypoxia probably causes loss of genomic stability through repression of DNA-DRR functions. Previous studies have also suggested that any of several transcription factors—E2Fs, Myc/Max, Myc/Sp1, or HIF-1α - may participate in the mechanisms of repression of *BRCA1*, *RAD51*, *NBS1*, *MSH6*, or *MSH2*, as well as subsequent acquired genomic instability, under hypoxic conditions [22, 24, 25–27]. We also previously demonstrated that two of the HIF-1-target gene products, DEC1 and DEC2, decreased *MLH1* transcription under hypoxic conditions [15]. In this study, we aimed to clarify the mechanisms of regulation of DNA-damage response occurring through transcriptional regulation under hypoxic conditions. We found that expression of DNA-DRR genes is systemically down-regulated under hypoxic conditions, and that both DNA-damage response and subsequent apoptosis induced by bleomycin or γ-ray irradiation are to some extent attenuated by DECs.

We first performed a set of comprehensive gene-expression analyses of the head and neck cancer cell line, and found that expression of a wide range of DNA-DRR genes decreased under hypoxic conditions through gene-annotation enrichment analysis using DAVID Bioinformatics Resources (S3 and S4 Tables). It is generally known that the major DNA-repair pathways -mismatch repair, nucleotide excision repair, base excision repair, homologous recombination repair, and non-homologous end joining- involve a large number of molecules [40]. Our results show that most genes involved in these pathways exhibit decreased expression under hypoxic conditions, resulting in systemic repression of DNA-damage response, although expression of some of these genes was not drastically repressed. A collective-intelligence approach to analyzing gene-expression data on hypoxic responses in the GEO database strongly supported our results and even allowed us to extend the significance of our findings to other cancer and non-cancer cells (S5 and S6 Tables). This leads to consideration of possible mechanisms of resistance to therapy and acquired mutator phenotypes in various types of cells under hypoxic conditions.

Increasing evidence from hypoxic researches has revealed dynamic changes in gene transcription by crucial transcription factors, i.e., hypoxia-inducible factors, under hypoxic conditions [41–44]. Among the transcription factors involved in those changes, the most prominent primarily activate gene transcription. Although some inhibitory mechanisms involving HIF or other transcription factors, such as REST, have been suggested previously [26, 27, 45], mechanisms of hypoxia-induced transcriptional repression remained largely unknown. We previously demonstrated the functional significance of the HIF-DEC signal pathway in *MLH1* transcription under hypoxia [15]. Other groups further have reported that HIF-1 induces DEC-repressed expression of *PPARG*, *PER*, *STAT1*, *SREBF1*, and *MITF* [10–14]. In the present study, we examined the transcriptional mechanisms of various DNA-DRR genes under hypoxia by focusing on the HIF-DEC signal pathway. We first clarified the functional significance of HIF-1α, DEC1, and DEC2 in regard to expression levels of several DNA-DRR genes, using knock-down analyses. As we had previously shown regarding regulation of *MLH1* transcription, *DEC2* knock-down clearly canceled those hypoxic regulations, and even increased *MRE11A* expression. However, knock-down of *DEC1* did not show any effect on hypoxic-related decrease in expression of those genes. One possible explanation for the lack of a *DEC1* effect is that *DEC1* knock-down significantly increases *DEC2* expression, resulting in enhanced repression of DEC-target genes, as had been previously shown [15]. We also observed that *HIF1A* knock-down mildly affected expression of DNA-DRR, suggesting that there are other pathways, including HIF-2α (EPAS1). Treatments with HIF-activating reagents, prolyl-hydroxylase inhibitors, further supported a role of HIF-DEC signal pathway on regulation of DNA-DRR gene expressions (S2 Fig). Furthermore, forced expression of DEC1 or DEC2 reduced cloned promoter activities of DNA-DRR genes, and those reductions were attenuated with the HDAC inhibitor, TSA, in a dose-dependent manner (S5 Fig), although the effect of TSA on reporter plasmid vector was not clear. Other experiments using a mutant type of DEC1 lacking the DNA binding domain demonstrated that DNA binding activity of DEC1 is critical for repression of some, but not all, of those promoter activities, suggesting the existence of other mechanisms. Analyses of ChIP-sequence data using the UCSC Genome Browser (genome.ucsc.edu) have indicated that DEC1 (BHLHE40) binding signals have been observed near the transcription start sites of a large number of genes, including known target genes [11–13, 15, 46, 47] (S6 and S7 Figs), and 129 of DNA-DRR genes (S8 Fig), suggesting that the DNA-binding activity of DEC1 is involved in the regulation of those genes. These results strongly indicate DECs as important regulators of transcription of DNA-DRR genes under hypoxic conditions.

Ionizing radiation produces intracellular reactive oxygen species (ROS) that damage cellular components, including DNA and proteins. Because oxygen is required for ROS generation, tumor hypoxia contributes to acquiring resistance to the cytotoxic effects of radiotherapy [4, 48]. In fact, tumor hypoxia is clinically known to promote resistance to both chemo- and radio-therapy, and HIF-1α is recognized as a molecular target to enhance the cytotoxic effects of chemo- or radio-therapy on cancer cells thereby improving prognosis [1–4]. In this study, we demonstrated that inhibition of DEC2 restored, at least in part, sensitivity of hypoxic cancer cell lines to bleomycin or radiation treatment, suggesting a potential molecular target for modifying DNA damage-inducing treatments. DECs have been reported to participate in the regulation of cell differentiation, cell-cycle control, and apoptosis [49–53]. DEC1 has been reported to reduce apoptosis and promote survival of cancer cells subjected to serum deprivation or photo-chemotherapeutic agents [51, 52]. DEC2 has also been reported to protect cancer cells from chemotherapeutic agents, leading to prolonged survival of those cells. Furthermore, DECs have been shown to decrease levels of Cleaved Caspase-3 protein in cells subjected to serum deprivation or 5-FU treatment. In our study, hypoxic treatment actually repressed and delayed the Cleavage of Caspase-3 induced by bleomycin or radiation treatment in HSC-2 cells, and inhibition of DEC2 effectively restored apoptosis signaling and sensitivity to radiation treatment. This may be due not only to direct regulation of apoptotic molecules by DECs as previously shown, but also to systemic repression of DNA-DRR genes by DECs, as we have shown in this report.

In summary, we demonstrated that the hypoxia-inducible transcription repressors DEC1 and DEC2 play crucial roles in transcriptional repression of numerous DNA-DRR genes under hypoxic conditions, and the HIF-DEC signaling pathway. DEC2, in particular, participates in DNA damage tolerance in cells exposed to low levels of oxygen. Further investigation of this pathway may provide new insights into the mechanisms and efficacy of DNA damage-inducing treatments in hypoxic cancer cells, as well as providing strategies for protecting normal tissues from the effect of radiation exposure.

## Supporting information

S1 Table Primer sets and MGB probes for real-time RT-PCR. (PDF)

S2 Table Gene-annotation enrichment analysis of hypoxia-upregulated genes in HSC-2 cells was performed using DAVID Bioinformatics Resources 6.8 (http://david.ncifcrf.gov/home.jsp)). (PDF)

S3 Table Gene-annotation enrichment analysis of hypoxia-downregulated genes in HSC-2 cells was performed using DAVID Bioinformatics Resources 6.8 (http://david.ncifcrf.gov/home.jsp)). (PDF)

S4 Table Expressions of DNA-DRR genes under normoxic or hypoxic conditions for 24 hours in HSC-2 cells. (PDF)

S5 Table List of the NCBI Gene Expression Omnibus database analyzed in this study. (PDF)

S6 Table Hypoxic down-regulation of DNA-DRR genes in cancer, and non-cancer, cells analyzed using the NCBI Gene Expression Omnibus database. (PDF)

S1 Fig Scatter plot of gene expressions in HSC-2. A scatter plot of normalized gene expressions in HSC-2 under normoxic versus hypoxic conditions is shown in left panel. Known hypoxia inducible genes (listed in right panel) and DNA-DRR genes (listed in S4 Table) are indicated as red and dark blue dots.
(PDF)

S2 Fig Expressions of DNA-DRR genes were decreased by treatments with HIF-activating reagents. HSC-2 cells were incubated under 21% pO_2_ (N) or 1% pO_2_ (H) for 24 hours, or treated with HIF-activating reagents,10 μM FG-4592, 50 μM 2,2’-Dipyridyl (DP), 200 μM CoCl_2_, 400 μM Dimethyloxalylglycine (DMOG), or 50 μM Desferoxamine mesylate (DFOM) for 24 hours. Expression levels of *MSH2*, *MBD4*, *MRE11A*, *BRCA1*, *RAD51*, and *MLH1* were analyzed by real-time RT-PCR. Relative mRNA levels were calculated as the ratio to that of *ACTB*. Columns are the mean of three independent experiments; bars, SD. The differences between means were significant (ANOVA *P* < 0.005). *P* values calculated with Dunnet test are: *, *P* < 0.05; **, *P* < 0.01; ***, *P* < 0.001.
(PDF)

S3 Fig Knockdown assays for *HIF1A*, *DEC1*, and *DEC2* in HSC-2 cells. Non-specific siRNA (siNS) or targeted siRNA was transfected into HSC-2 and cultured under normoxic or hypoxic conditions for 24 hours. Expression levels of *HIF1A*, *DEC1*, and *DEC2* were evaluated by real-time RT-PCR (left panel). Relative mRNA levels were calculated as the ratio to that of *ACTB*. Columns are the mean of three independent experiments; bars, SD. The differences between means were significant (ANOVA *P* < 0.005). *P* values calculated with Turkey-Kramer HSD test are: *, *P* < 0.05; **, *P* < 0.01; ***, *P* < 0.001. Expression levels of HIF-1α, DEC1, and DEC2 proteins were evaluated using immunostaining analysis (right panel).
(PDF)

S4 Fig Phosphorylate histone H2AX induced by X-ray were inhibited in hypoxic pre-conditioned HepG2 cells. Phosphorylate histone H2AX (γH2AX) levels after X-ray irradiation in HepG2 cells with or without pre-conditioning under hypoxia were evaluated with immunostaining analysis. The cells were stained with primary anti-γH2AX and secondary rhodamine-labeled antibody. Red shows γH2AX foci, and blue is nuclei stained with Hoechst 33342 (left panel). The right-side panel shows the number of γH2AX foci per cell. Total numbers of γH2AX foci and cells were counted in the left panel, and calculated. Columns are the mean of three independent counts; bars, SD. The differences between means were significant (ANOVA *P* < 0.005). *P* values calculated with Turkey-Kramer HSD test are: **, *P* < 0.01; ***, *P* < 0.001.
(PDF)

S5 Fig Suppressed promoter activities of DNA-DRR genes by DEC were increased with TSA treatments. The pGL3-promoter reporters and DEC1 or DEC2 expression plasmid were co-transfected into HepG2 and treated with 10 or 100 ng/ml of Trichostatin A (TSA), histon deacetylase (HDAC) inhibitor, 24 hours before harvesting cells. Luciferase activities were determined with a dual-luciferase assay system. Columns are the mean of three independent experiments; bars, SD. The differences between means were significant (ANOVA *P* < 0.005). *P* values calculated with Turkey-Kramer HSD test are: *, *P* < 0.05; **, *P* < 0.01; ***, *P* < 0.001.
(PDF)

S6 Fig High peaks and signals of DEC1 (also known as BHLHE40) in known target genes. Using the UCSC genome browser database, we analyzed the BHLHE40 ChIP-sequence peaks and signals near the transcription start site of already-reported DEC1 target genes, *BHLHE41* (also known as *DEC2*), *DBP*, *PER1*, and *PER2*.
(PDF)

S7 Fig High peaks and signals of DEC1 (also known as BHLHE40) in the genes regulated by HIF-1-DEC pathway. Using the UCSC genome browser database, we analyzed the BHLHE40 ChIP-sequence peaks and signals near the transcription start site of HIF-1-DEC pathway-regulated genes, *MLH1*, *STAT1*, and *SREBP-1c* (also known as *SREBF1*).
(PDF)

S8 Fig High peaks and signals of DEC1 (also known as BHLHE40) in genes related to DNA-DRR. Using the UCSC genome browser database, we analyzed the BHLHE40 ChIP-sequence peaks and signals near the transcription start site of genes related to DNA-DRR listed in S3 Table.
(PDF)
