# Supplementary material for: Differentiated embryo chondrocyte plays a crucial role in DNA damage response via transcriptional regulation under hypoxic conditions
Source: PLoS One. 2018 Feb 21;13(2):e0192136. doi: 10.1371/journal.pone.0192136 (PMC5821451; doi:10.1371/journal.pone.0192136)
Supplement: S1 Table — (PDF) [file pone.0192136.s001.pdf]

S1 Table. Primer sets and MGB probes for real-time RT-PCR.

HIF1A-F: 5'- GAACCTGATGCTTTAACTTTGCT -3'
HIF1A -R: 5'- TGCTGGTCATCAGTTTCTGTG -3'
HIF1A -probe: UPL #28 (Roche)
DEC1-F: 5'- GACTGGAGCACGGAGACCT -3'
DEC1-R: 5'- GGTGCGGCAATTTGTAGG -3'
DEC1-probe: UPL #56 (Roche)
DEC2-F: 5'- CTACTGCGTGCCCGTCAT -3'
DEC2-R: 5'- CGGTGTCCGTGTCGTTCT -3'
DEC2-probe: UPL #26 (Roche)
