# Supplementary material for: Differentiated embryo chondrocyte plays a crucial role in DNA damage response via transcriptional regulation under hypoxic conditions
Source: PLoS One. 2018 Feb 21;13(2):e0192136. doi: 10.1371/journal.pone.0192136 (PMC5821451; doi:10.1371/journal.pone.0192136)
Supplement: S2 Table — (PDF) [file pone.0192136.s002.pdf]

S2 Table. Gene-annotation enrichment analysis of hypoxia-upregulated genes in HSC2 cells was performed by DAVID Bioinformatics Resources 6.8 (http://david.ncifcrf.gov/home.jsp).

Category	Term	Count	%	PValue
GOTERM_BP_DIRECT	GO:0001525~angiogenesis	55	2.68	7.68E-09
GOTERM_BP_DIRECT	GO:0001666~response to hypoxia	45	2.20	3.30E-08
GOTERM_BP_DIRECT	GO:0007155~cell adhesion	88	4.29	1.01E-07
GOTERM_BP_DIRECT	GO:0043547~positive regulation of GTPase activity	103	5.03	1.04E-07
GOTERM_BP_DIRECT	GO:0030198~extracellular matrix organization	46	2.24	6.76E-07
GOTERM_BP_DIRECT	GO:0007267~cell-cell signaling	55	2.68	7.50E-07
GOTERM_BP_DIRECT	GO:0007165~signal transduction	175	8.54	3.03E-06
GOTERM_BP_DIRECT	GO:0007399~nervous system development	58	2.83	3.65E-06
GOTERM_BP_DIRECT	GO:0006094~gluconeogenesis	17	0.83	6.89E-06
GOTERM_BP_DIRECT	GO:0071456~cellular response to hypoxia	26	1.27	2.10E-05
GOTERM_BP_DIRECT	GO:0002576~platelet degranulation	27	1.32	2.62E-05
GOTERM_BP_DIRECT	GO:0061621~canonical glycolysis	12	0.59	3.71E-05
GOTERM_BP_DIRECT	GO:0002548~monocyte chemotaxis	15	0.73	7.83E-05
GOTERM_BP_DIRECT	GO:0051482~positive regulation of cytosolic calcium ion concentration involved in phospholipase C-activating G-protein coupled signaling pathway	12	0.59	8.40E-05
GOTERM_BP_DIRECT	GO:0048675~axon extension	11	0.54	9.84E-05
GOTERM_BP_DIRECT	GO:0006874~cellular calcium ion homeostasis	24	1.17	1.05E-04
GOTERM_BP_DIRECT	GO:0070098~chemokine-mediated signaling pathway	20	0.98	1.35E-04
GOTERM_BP_DIRECT	GO:0007268~chemical synaptic transmission	46	2.24	1.54E-04
GOTERM_BP_DIRECT	GO:0006954~inflammatory response	65	3.17	1.85E-04
GOTERM_BP_DIRECT	GO:0051930~regulation of sensory perception of pain	11	0.54	2.18E-04
GOTERM_BP_DIRECT	GO:0071356~cellular response to tumor necrosis factor	26	1.27	2.29E-04
GOTERM_BP_DIRECT	GO:0007229~integrin-mediated signaling pathway	24	1.17	2.86E-04
GOTERM_BP_DIRECT	GO:0007568~aging	34	1.66	3.36E-04
GOTERM_BP_DIRECT	GO:0043524~negative regulation of neuron apoptotic process	29	1.42	3.41E-04
GOTERM_BP_DIRECT	GO:0006955~immune response	69	3.37	4.39E-04
GOTERM_BP_DIRECT	GO:0007204~positive regulation of cytosolic calcium ion concentration	29	1.42	4.41E-04
GOTERM_BP_DIRECT	GO:0002027~regulation of heart rate	12	0.59	4.55E-04
GOTERM_BP_DIRECT	GO:0070374~positive regulation of ERK1 and ERK2 cascade	35	1.71	4.81E-04
GOTERM_BP_DIRECT	GO:0030593~neutrophil chemotaxis	18	0.88	4.86E-04
GOTERM_BP_DIRECT	GO:0007166~cell surface receptor signaling pathway	49	2.39	4.89E-04
GOTERM_BP_DIRECT	GO:0006935~chemotaxis	27	1.32	5.06E-04
GOTERM_BP_DIRECT	GO:0008219~cell death	13	0.63	5.80E-04
GOTERM_BP_DIRECT	GO:0035556~intracellular signal transduction	66	3.22	6.04E-04
GOTERM_BP_DIRECT	GO:0045909~positive regulation of vasodilation	11	0.54	6.06E-04
GOTERM_BP_DIRECT	GO:0001662~behavioral fear response	11	0.54	6.06E-04
GOTERM_BP_DIRECT	GO:0006096~glycolytic process	12	0.59	6.07E-04
GOTERM_BP_DIRECT	GO:0009968~negative regulation of signal transduction	12	0.59	6.07E-04
GOTERM_BP_DIRECT	GO:0090037~positive regulation of protein kinase C signaling	6	0.29	6.16E-04
GOTERM_BP_DIRECT	GO:0043410~positive regulation of MAPK cascade	20	0.98	8.23E-04
GOTERM_BP_DIRECT	GO:0009611~response to wounding	17	0.83	8.33E-04
GOTERM_BP_DIRECT	GO:0006909~phagocytosis	14	0.68	0.001
GOTERM_BP_DIRECT	GO:0015758~glucose transport	11	0.54	0.002
GOTERM_BP_DIRECT	GO:0007605~sensory perception of sound	27	1.32	0.002
GOTERM_BP_DIRECT	GO:0001938~positive regulation of endothelial cell proliferation	17	0.83	0.002
GOTERM_BP_DIRECT	GO:0060412~ventricular septum morphogenesis	10	0.49	0.003
GOTERM_BP_DIRECT	GO:0005977~glycogen metabolic process	10	0.49	0.003
GOTERM_BP_DIRECT	GO:0038083~peptidyl-tyrosine autophosphorylation	12	0.59	0.003
GOTERM_BP_DIRECT	GO:0071300~cellular response to retinoic acid	17	0.83	0.003
GOTERM_BP_DIRECT	GO:0071347~cellular response to interleukin-1	17	0.83	0.003
GOTERM_BP_DIRECT	GO:0060326~cell chemotaxis	16	0.78	0.003
GOTERM_BP_DIRECT	GO:0010628~positive regulation of gene expression	44	2.15	0.003
GOTERM_BP_DIRECT	GO:0043065~positive regulation of apoptotic process	49	2.39	0.003
GOTERM_BP_DIRECT	GO:0006468~protein phosphorylation	69	3.37	0.004
GOTERM_BP_DIRECT	GO:1901385~regulation of voltage-gated calcium channel activity	5	0.24	0.004
GOTERM_BP_DIRECT	GO:0019229~regulation of vasoconstriction	8	0.39	0.004
GOTERM_BP_DIRECT	GO:0048010~vascular endothelial growth factor receptor signaling pathway	17	0.83	0.004
GOTERM_BP_DIRECT	GO:0008360~regulation of cell shape	27	1.32	0.004
GOTERM_BP_DIRECT	GO:0051056~regulation of small GTPase mediated signal transduction	26	1.27	0.004
GOTERM_BP_DIRECT	GO:1902476~chloride transmembrane transport	20	0.98	0.004
GOTERM_BP_DIRECT	GO:0042127~regulation of cell proliferation	33	1.61	0.005
GOTERM_BP_DIRECT	GO:0045987~positive regulation of smooth muscle contraction	8	0.39	0.005
GOTERM_BP_DIRECT	GO:0032496~response to lipopolysaccharide	30	1.46	0.005
GOTERM_BP_DIRECT	GO:0030168~platelet activation	23	1.12	0.005
GOTERM_BP_DIRECT	GO:1903779~regulation of cardiac conduction	14	0.68	0.006
GOTERM_BP_DIRECT	GO:0030049~muscle filament sliding	11	0.54	0.006
GOTERM_BP_DIRECT	GO:0015701~bicarbonate transport	12	0.59	0.006
GOTERM_BP_DIRECT	GO:0019731~antibacterial humoral response	12	0.59	0.006
GOTERM_BP_DIRECT	GO:0045778~positive regulation of ossification	6	0.29	0.006
GOTERM_BP_DIRECT	GO:0007169~transmembrane receptor protein tyrosine kinase signaling pathway	20	0.98	0.006
GOTERM_BP_DIRECT	GO:1904706~negative regulation of vascular smooth muscle cell proliferation	5	0.24	0.007
GOTERM_BP_DIRECT	GO:0043171~peptide catabolic process	8	0.39	0.007
GOTERM_BP_DIRECT	GO:0060048~cardiac muscle contraction	12	0.59	0.007
GOTERM_BP_DIRECT	GO:0061337~cardiac conduction	12	0.59	0.007
GOTERM_BP_DIRECT	GO:0007507~heart development	32	1.56	0.007
GOTERM_BP_DIRECT	GO:0060079~excitatory postsynaptic potential	9	0.44	0.008
GOTERM_BP_DIRECT	GO:0030335~positive regulation of cell migration	32	1.56	0.008
GOTERM_BP_DIRECT	GO:0001764~neuron migration	21	1.02	0.008
GOTERM_BP_DIRECT	GO:0006633~fatty acid biosynthetic process	13	0.63	0.008
GOTERM_BP_DIRECT	GO:0010595~positive regulation of endothelial cell migration	12	0.59	0.008
GOTERM_BP_DIRECT	GO:0008217~regulation of blood pressure	15	0.73	0.008
GOTERM_BP_DIRECT	GO:0014068~positive regulation of phosphatidylinositol 3-kinase signaling	15	0.73	0.008
GOTERM_BP_DIRECT	GO:0051966~regulation of synaptic transmission, glutamatergic	8	0.39	0.009
GOTERM_BP_DIRECT	GO:0006941~striated muscle contraction	6	0.29	0.009
GOTERM_BP_DIRECT	GO:0007417~central nervous system development	23	1.12	0.009
GOTERM_BP_DIRECT	GO:0043691~reverse cholesterol transport	7	0.34	0.009
GOTERM_BP_DIRECT	GO:0002690~positive regulation of leukocyte chemotaxis	7	0.34	0.009
GOTERM_BP_DIRECT	GO:0006898~receptor-mediated endocytosis	32	1.56	0.009
GOTERM_BP_DIRECT	GO:0051209~release of sequestered calcium ion into cytosol	11	0.54	0.010

GOTERM_BP_DIRECT	GO:0050729~positive regulation of inflammatory response	16	0.78	0.010
GOTERM_BP_DIRECT	GO:0018401~peptidyl-proline hydroxylation to 4-hydroxy-L-proline	4	0.20	0.011
GOTERM_BP_DIRECT	GO:0006930~substrate-dependent cell migration, cell extension	5	0.24	0.011
GOTERM_BP_DIRECT	GO:0014824~artery smooth muscle contraction	5	0.24	0.011
GOTERM_BP_DIRECT	GO:0070102~interleukin-6-mediated signaling pathway	5	0.24	0.011
GOTERM_BP_DIRECT	GO:0030099~myeloid cell differentiation	8	0.39	0.011
GOTERM_BP_DIRECT	GO:0007411~axon guidance	28	1.37	0.011
GOTERM_BP_DIRECT	GO:0035176~social behavior	12	0.59	0.012
GOTERM_BP_DIRECT	GO:0030336~negative regulation of cell migration	19	0.93	0.012
GOTERM_BP_DIRECT	GO:0001894~tissue homeostasis	7	0.34	0.012
GOTERM_BP_DIRECT	GO:0001659~temperature homeostasis	7	0.34	0.012
GOTERM_BP_DIRECT	GO:0050965~detection of temperature stimulus involved in sensory perception of pain	6	0.29	0.013
GOTERM_BP_DIRECT	GO:0071354~cellular response to interleukin-6	6	0.29	0.013
GOTERM_BP_DIRECT	GO:0018108~peptidyl-tyrosine phosphorylation	27	1.32	0.013
GOTERM_BP_DIRECT	GO:0050731~positive regulation of peptidyl-tyrosine phosphorylation	17	0.83	0.013
GOTERM_BP_DIRECT	GO:0007565~female pregnancy	18	0.88	0.014
GOTERM_BP_DIRECT	GO:0006810~transport	52	2.54	0.014
GOTERM_BP_DIRECT	GO:0021549~cerebellum development	10	0.49	0.015
GOTERM_BP_DIRECT	GO:0007173~epidermal growth factor receptor signaling pathway	13	0.63	0.015
GOTERM_BP_DIRECT	GO:0070588~calcium ion transmembrane transport	22	1.07	0.016
GOTERM_BP_DIRECT	GO:0007275~multicellular organism development	73	3.56	0.016
GOTERM_BP_DIRECT	GO:0019732~antifungal humoral response	5	0.24	0.017
GOTERM_BP_DIRECT	GO:0006000~fructose metabolic process	5	0.24	0.017
GOTERM_BP_DIRECT	GO:0051156~glucose 6-phosphate metabolic process	5	0.24	0.017
GOTERM_BP_DIRECT	GO:0043267~negative regulation of potassium ion transport	5	0.24	0.017
GOTERM_BP_DIRECT	GO:0007626~locomotory behavior	17	0.83	0.017
GOTERM_BP_DIRECT	GO:0043434~response to peptide hormone	11	0.54	0.017
GOTERM_BP_DIRECT	GO:0030502~negative regulation of bone mineralization	6	0.29	0.017
GOTERM_BP_DIRECT	GO:0006198~cAMP catabolic process	6	0.29	0.017
GOTERM_BP_DIRECT	GO:0051928~positive regulation of calcium ion transport	8	0.39	0.018
GOTERM_BP_DIRECT	GO:0001958~endochondral ossification	8	0.39	0.018
GOTERM_BP_DIRECT	GO:0051491~positive regulation of filopodium assembly	8	0.39	0.018
GOTERM_BP_DIRECT	GO:0030574~collagen catabolic process	14	0.68	0.018
GOTERM_BP_DIRECT	GO:0001974~blood vessel remodeling	9	0.44	0.018
GOTERM_BP_DIRECT	GO:0050873~brown fat cell differentiation	9	0.44	0.018
GOTERM_BP_DIRECT	GO:0008284~positive regulation of cell proliferation	66	3.22	0.018
GOTERM_BP_DIRECT	GO:0006469~negative regulation of protein kinase activity	19	0.93	0.018
GOTERM_BP_DIRECT	GO:0038084~vascular endothelial growth factor signaling pathway	4	0.20	0.020
GOTERM_BP_DIRECT	GO:0032367~intracellular cholesterol transport	4	0.20	0.020
GOTERM_BP_DIRECT	GO:0002686~negative regulation of leukocyte migration	4	0.20	0.020
GOTERM_BP_DIRECT	GO:0043627~response to estrogen	14	0.68	0.020
GOTERM_BP_DIRECT	GO:0050728~negative regulation of inflammatory response	16	0.78	0.021
GOTERM_BP_DIRECT	GO:0008277~regulation of G-protein coupled receptor protein signaling pathway	10	0.49	0.021
GOTERM_BP_DIRECT	GO:0010629~negative regulation of gene expression	24	1.17	0.021
GOTERM_BP_DIRECT	GO:0019233~sensory perception of pain	12	0.59	0.021
GOTERM_BP_DIRECT	GO:0016337~single organismal cell-cell adhesion	19	0.93	0.022
GOTERM_BP_DIRECT	GO:0050790~regulation of catalytic activity	14	0.68	0.023
GOTERM_BP_DIRECT	GO:0007200~phospholipase C-activating G-protein coupled receptor signaling pathway	14	0.68	0.023
GOTERM_BP_DIRECT	GO:0003151~outflow tract morphogenesis	11	0.54	0.023
GOTERM_BP_DIRECT	GO:0010613~positive regulation of cardiac muscle hypertrophy	6	0.29	0.023
GOTERM_BP_DIRECT	GO:0090026~positive regulation of monocyte chemotaxis	6	0.29	0.023
GOTERM_BP_DIRECT	GO:0007596~blood coagulation	30	1.46	0.024
GOTERM_BP_DIRECT	GO:0033197~response to vitamin E	5	0.24	0.024
GOTERM_BP_DIRECT	GO:0002446~neutrophil mediated immunity	5	0.24	0.024
GOTERM_BP_DIRECT	GO:0045787~positive regulation of cell cycle	9	0.44	0.025
GOTERM_BP_DIRECT	GO:0045332~phospholipid translocation	7	0.34	0.026
GOTERM_BP_DIRECT	GO:0061418~regulation of transcription from RNA polymerase II promoter in response to hypoxia	8	0.39	0.026
GOTERM_BP_DIRECT	GO:2000352~negative regulation of endothelial cell apoptotic process	8	0.39	0.026
GOTERM_BP_DIRECT	GO:0048247~lymphocyte chemotaxis	8	0.39	0.026
GOTERM_BP_DIRECT	GO:0031532~actin cytoskeleton reorganization	11	0.54	0.026
GOTERM_BP_DIRECT	GO:0007193~adenylate cyclase-inhibiting G-protein coupled receptor signaling pathway	11	0.54	0.026
GOTERM_BP_DIRECT	GO:0051781~positive regulation of cell division	11	0.54	0.026
GOTERM_BP_DIRECT	GO:0010976~positive regulation of neuron projection development	17	0.83	0.028
GOTERM_BP_DIRECT	GO:0008203~cholesterol metabolic process	14	0.68	0.028
GOTERM_BP_DIRECT	GO:0030193~regulation of blood coagulation	6	0.29	0.030
GOTERM_BP_DIRECT	GO:0060307~regulation of ventricular cardiac muscle cell membrane repolarization	6	0.29	0.030
GOTERM_BP_DIRECT	GO:0044849~estrous cycle	6	0.29	0.030
GOTERM_BP_DIRECT	GO:0032331~negative regulation of chondrocyte differentiation	6	0.29	0.030
GOTERM_BP_DIRECT	GO:0006641~triglyceride metabolic process	9	0.44	0.030
GOTERM_BP_DIRECT	GO:0006629~lipid metabolic process	26	1.27	0.030
GOTERM_BP_DIRECT	GO:0050829~defense response to Gram-negative bacterium	12	0.59	0.031
GOTERM_BP_DIRECT	GO:0030388~fructose 1,6-bisphosphate metabolic process	4	0.20	0.031
GOTERM_BP_DIRECT	GO:0048842~positive regulation of axon extension involved in axon guidance	4	0.20	0.031
GOTERM_BP_DIRECT	GO:0048066~developmental pigmentation	4	0.20	0.031
GOTERM_BP_DIRECT	GO:0070474~positive regulation of uterine smooth muscle contraction	4	0.20	0.031
GOTERM_BP_DIRECT	GO:0060373~regulation of ventricular cardiac muscle cell membrane depolarization	4	0.20	0.031
GOTERM_BP_DIRECT	GO:0016525~negative regulation of angiogenesis	13	0.63	0.031
GOTERM_BP_DIRECT	GO:0006968~cellular defense response	13	0.63	0.031
GOTERM_BP_DIRECT	GO:0060395~SMAD protein signal transduction	13	0.63	0.031
GOTERM_BP_DIRECT	GO:0051965~positive regulation of synapse assembly	13	0.63	0.031
GOTERM_BP_DIRECT	GO:0045471~response to ethanol	19	0.93	0.032
GOTERM_BP_DIRECT	GO:0048871~multicellular organismal homeostasis	3	0.15	0.032
GOTERM_BP_DIRECT	GO:0003190~atrioventricular valve formation	3	0.15	0.032
GOTERM_BP_DIRECT	GO:1903413~cellular response to bile acid	3	0.15	0.032
GOTERM_BP_DIRECT	GO:0007566~embryo implantation	10	0.49	0.032
GOTERM_BP_DIRECT	GO:0051496~positive regulation of stress fiber assembly	10	0.49	0.032
GOTERM_BP_DIRECT	GO:0010447~response to acidic pH	5	0.24	0.033
GOTERM_BP_DIRECT	GO:0006069~ethanol oxidation	5	0.24	0.033
GOTERM_BP_DIRECT	GO:0071498~cellular response to fluid shear stress	5	0.24	0.033

GOTERM_BP_DIRECT	GO:0035815~positive regulation of renal sodium excretion	5	0.24	0.033
GOTERM_BP_DIRECT	GO:0021702~cerebellar Purkinje cell differentiation	5	0.24	0.033
GOTERM_BP_DIRECT	GO:0071560~cellular response to transforming growth factor beta stimulus	11	0.54	0.034
GOTERM_BP_DIRECT	GO:0021762~substantia nigra development	11	0.54	0.034
GOTERM_BP_DIRECT	GO:0001570~vasculogenesis	12	0.59	0.035
GOTERM_BP_DIRECT	GO:0000165~MAPK cascade	39	1.90	0.036
GOTERM_BP_DIRECT	GO:0007179~transforming growth factor beta receptor signaling pathway	17	0.83	0.037
GOTERM_BP_DIRECT	GO:0030217~T cell differentiation	8	0.39	0.037
GOTERM_BP_DIRECT	GO:0044344~cellular response to fibroblast growth factor stimulus	8	0.39	0.037
GOTERM_BP_DIRECT	GO:0045429~positive regulation of nitric oxide biosynthetic process	10	0.49	0.037
GOTERM_BP_DIRECT	GO:0030155~regulation of cell adhesion	10	0.49	0.037
GOTERM_BP_DIRECT	GO:0033280~response to vitamin D	6	0.29	0.038
GOTERM_BP_DIRECT	GO:0006970~response to osmotic stress	6	0.29	0.038
GOTERM_BP_DIRECT	GO:0050870~positive regulation of T cell activation	6	0.29	0.038
GOTERM_BP_DIRECT	GO:0048041~focal adhesion assembly	7	0.34	0.038
GOTERM_BP_DIRECT	GO:0051602~response to electrical stimulus	7	0.34	0.038
GOTERM_BP_DIRECT	GO:0035235~ionotropic glutamate receptor signaling pathway	7	0.34	0.038
GOTERM_BP_DIRECT	GO:0007266~Rho protein signal transduction	11	0.54	0.039
GOTERM_BP_DIRECT	GO:0007612~learning	12	0.59	0.039
GOTERM_BP_DIRECT	GO:0048813~dendrite morphogenesis	9	0.44	0.041
GOTERM_BP_DIRECT	GO:0019221~cytokine-mediated signaling pathway	22	1.07	0.042
GOTERM_BP_DIRECT	GO:0045944~positive regulation of transcription from RNA polymerase II promoter	124	6.05	0.042
GOTERM_BP_DIRECT	GO:0010977~negative regulation of neuron projection development	10	0.49	0.043
GOTERM_BP_DIRECT	GO:0071318~cellular response to ATP	5	0.24	0.044
GOTERM_BP_DIRECT	GO:0048246~macrophage chemotaxis	5	0.24	0.044
GOTERM_BP_DIRECT	GO:0042542~response to hydrogen peroxide	11	0.54	0.044
GOTERM_BP_DIRECT	GO:0007205~protein kinase C-activating G-protein coupled receptor signaling pathway	8	0.39	0.044
GOTERM_BP_DIRECT	GO:0043552~positive regulation of phosphatidylinositol 3-kinase activity	8	0.39	0.044
GOTERM_BP_DIRECT	GO:0034220~ion transmembrane transport	32	1.56	0.044
GOTERM_BP_DIRECT	GO:0060135~maternal process involved in female pregnancy	7	0.34	0.046
GOTERM_BP_DIRECT	GO:0051899~membrane depolarization	7	0.34	0.046
GOTERM_BP_DIRECT	GO:0002040~sprouting angiogenesis	7	0.34	0.046
GOTERM_BP_DIRECT	GO:0001701~in utero embryonic development	29	1.42	0.046
GOTERM_BP_DIRECT	GO:0007172~signal complex assembly	4	0.20	0.046
GOTERM_BP_DIRECT	GO:0007216~G-protein coupled glutamate receptor signaling pathway	4	0.20	0.046
GOTERM_BP_DIRECT	GO:0031102~neuron projection regeneration	4	0.20	0.046
GOTERM_BP_DIRECT	GO:0002467~germinal center formation	4	0.20	0.046
GOTERM_BP_DIRECT	GO:0090190~positive regulation of branching involved in ureteric bud morphogenesis	6	0.29	0.047
GOTERM_BP_DIRECT	GO:0060291~long-term synaptic potentiation	9	0.44	0.047
GOTERM_BP_DIRECT	GO:0008283~cell proliferation	51	2.49	0.048
GOTERM_BP_DIRECT	GO:0008015~blood circulation	10	0.49	0.048
GOTERM_BP_DIRECT	GO:0001755~neural crest cell migration	10	0.49	0.048
GOTERM_BP_DIRECT	GO:0032147~activation of protein kinase activity	10	0.49	0.048
GOTERM_BP_DIRECT	GO:0051216~cartilage development	12	0.59	0.049
