# Supplementary material for: Differentiated embryo chondrocyte plays a crucial role in DNA damage response via transcriptional regulation under hypoxic conditions
Source: PLoS One. 2018 Feb 21;13(2):e0192136. doi: 10.1371/journal.pone.0192136 (PMC5821451; doi:10.1371/journal.pone.0192136)
Supplement: S3 Table — (PDF) [file pone.0192136.s003.pdf]

S3 Table. Gene-annotation enrichment analysis of hypoxia-downregulated genes in HSC2 cells was performed by DAVID Bioinformatics Resources 6.8 (http://david.ncifcrf.gov/home.jsp).

Category	Term	Count	%	PValue
GOTERM_BP_DIRECT	GO:0006260~DNA replication	67	2.88	4.83E-21
GOTERM_BP_DIRECT	GO:0006364~rRNA processing	71	3.05	7.06E-15
GOTERM_BP_DIRECT	GO:0070125~mitochondrial translational elongation	36	1.55	2.88E-11
GOTERM_BP_DIRECT	GO:0070126~mitochondrial translational termination	34	1.46	9.52E-10
GOTERM_BP_DIRECT	GO:0000082~G1/S transition of mitotic cell cycle	36	1.55	9.48E-09
GOTERM_BP_DIRECT	GO:0000398~mRNA splicing, via spliceosome	59	2.54	2.31E-08
GOTERM_BP_DIRECT	GO:0006270~DNA replication initiation	18	0.77	3.62E-08
GOTERM_BP_DIRECT	GO:0006281~DNA repair	60	2.58	8.14E-08
GOTERM_BP_DIRECT	GO:0051301~cell division	77	3.31	7.57E-07
GOTERM_BP_DIRECT	GO:0000722~telomere maintenance via recombination	16	0.69	1.87E-06
GOTERM_BP_DIRECT	GO:0007067~mitotic nuclear division	58	2.49	2.91E-06
GOTERM_BP_DIRECT	GO:0000732~strand displacement	14	0.60	3.70E-06
GOTERM_BP_DIRECT	GO:0006412~translation	58	2.49	5.58E-06
GOTERM_BP_DIRECT	GO:0006283~transcription-coupled nucleotide-excision repair	25	1.08	6.03E-06
GOTERM_BP_DIRECT	GO:0000731~DNA synthesis involved in DNA repair	16	0.69	7.43E-06
GOTERM_BP_DIRECT	GO:1900034~regulation of cellular response to heat	25	1.08	7.83E-06
GOTERM_BP_DIRECT	GO:0000724~double-strand break repair via homologous recombination	24	1.03	2.05E-05
GOTERM_BP_DIRECT	GO:0007062~sister chromatid cohesion	29	1.25	4.38E-05
GOTERM_BP_DIRECT	GO:0006310~DNA recombination	25	1.08	5.14E-05
GOTERM_BP_DIRECT	GO:0000387~spliceosomal snRNP assembly	13	0.56	6.14E-05
GOTERM_BP_DIRECT	GO:0006406~mRNA export from nucleus	28	1.20	6.76E-05
GOTERM_BP_DIRECT	GO:0008283~cell proliferation	72	3.10	9.19E-05
GOTERM_BP_DIRECT	GO:0034080~CENP-A containing nucleosome assembly	16	0.69	1.31E-04
GOTERM_BP_DIRECT	GO:0006189~'de novo' IMP biosynthetic process	6	0.26	1.63E-04
GOTERM_BP_DIRECT	GO:0006271~DNA strand elongation involved in DNA replication	9	0.39	1.66E-04
GOTERM_BP_DIRECT	GO:1904874~positive regulation of telomerase RNA localization to Cajal body	9	0.39	1.66E-04
GOTERM_BP_DIRECT	GO:0031145~anaphase-promoting complex-dependent catabolic process	23	0.99	1.90E-04
GOTERM_BP_DIRECT	GO:0006418~tRNA aminoacylation for protein translation	15	0.65	2.09E-04
GOTERM_BP_DIRECT	GO:0008033~tRNA processing	14	0.60	3.35E-04
GOTERM_BP_DIRECT	GO:0006390~transcription from mitochondrial promoter	7	0.30	5.05E-04
GOTERM_BP_DIRECT	GO:1901796~regulation of signal transduction by p53 class mediator	30	1.29	5.51E-04
GOTERM_BP_DIRECT	GO:0006369~termination of RNA polymerase II transcription	19	0.82	6.22E-04
GOTERM_BP_DIRECT	GO:0006396~RNA processing	25	1.08	6.84E-04
GOTERM_BP_DIRECT	GO:0006284~base-excision repair	13	0.56	7.28E-04
GOTERM_BP_DIRECT	GO:0006405~RNA export from nucleus	17	0.73	8.20E-04
GOTERM_BP_DIRECT	GO:0000723~telomere maintenance	13	0.56	9.69E-04
GOTERM_BP_DIRECT	GO:0016032~viral process	57	2.45	0.001
GOTERM_BP_DIRECT	GO:0008380~RNA splicing	36	1.55	0.001
GOTERM_BP_DIRECT	GO:0000470~maturation of LSU-rRNA	8	0.34	0.001
GOTERM_BP_DIRECT	GO:0006729~tetrahydrobiopterin biosynthetic process	6	0.26	0.001
GOTERM_BP_DIRECT	GO:0006457~protein folding	38	1.63	0.001
GOTERM_BP_DIRECT	GO:0006361~transcription initiation from RNA polymerase I promoter	12	0.52	0.002
GOTERM_BP_DIRECT	GO:0006164~purine nucleotide biosynthetic process	7	0.30	0.002
GOTERM_BP_DIRECT	GO:0006626~protein targeting to mitochondrion	12	0.52	0.002
GOTERM_BP_DIRECT	GO:0036297~interstrand cross-link repair	15	0.65	0.002
GOTERM_BP_DIRECT	GO:0051437~positive regulation of ubiquitin-protein ligase activity involved in regulation of mitotic cell cycle transition	20	0.86	0.002
GOTERM_BP_DIRECT	GO:0032508~DNA duplex unwinding	14	0.60	0.002
GOTERM_BP_DIRECT	GO:0016925~protein sumoylation	27	1.16	0.002
GOTERM_BP_DIRECT	GO:0042273~ribosomal large subunit biogenesis	10	0.43	0.002
GOTERM_BP_DIRECT	GO:0015949~nucleobase-containing small molecule interconversion	10	0.43	0.002
GOTERM_BP_DIRECT	GO:0007005~mitochondrion organization	20	0.86	0.002
GOTERM_BP_DIRECT	GO:0010388~cullin deneddylation	6	0.26	0.002
GOTERM_BP_DIRECT	GO:1904851~positive regulation of establishment of protein localization to telomere	6	0.26	0.002
GOTERM_BP_DIRECT	GO:0015886~heme transport	6	0.26	0.002
GOTERM_BP_DIRECT	GO:0006362~transcription elongation from RNA polymerase I promoter	11	0.47	0.003
GOTERM_BP_DIRECT	GO:0000027~ribosomal large subunit assembly	9	0.39	0.003
GOTERM_BP_DIRECT	GO:0048025~negative regulation of mRNA splicing, via spliceosome	9	0.39	0.003
GOTERM_BP_DIRECT	GO:0009168~purine ribonucleoside monophosphate biosynthetic process	7	0.30	0.003
GOTERM_BP_DIRECT	GO:0070986~left/right axis specification	7	0.30	0.003
GOTERM_BP_DIRECT	GO:0006521~regulation of cellular amino acid metabolic process	15	0.65	0.003
GOTERM_BP_DIRECT	GO:0006363~termination of RNA polymerase I transcription	11	0.47	0.003
GOTERM_BP_DIRECT	GO:0032543~mitochondrial translation	12	0.52	0.003
GOTERM_BP_DIRECT	GO:0006303~double-strand break repair via nonhomologous end joining	17	0.73	0.004
GOTERM_BP_DIRECT	GO:0007004~telomere maintenance via telomerase	8	0.34	0.004
GOTERM_BP_DIRECT	GO:0051170~nuclear import	8	0.34	0.004
GOTERM_BP_DIRECT	GO:0007093~mitotic cell cycle checkpoint	11	0.47	0.004
GOTERM_BP_DIRECT	GO:0006268~DNA unwinding involved in DNA replication	6	0.26	0.004
GOTERM_BP_DIRECT	GO:0051436~negative regulation of ubiquitin-protein ligase activity involved in mitotic cell cycle	18	0.77	0.006
GOTERM_BP_DIRECT	GO:0055114~oxidation-reduction process	96	4.13	0.006
GOTERM_BP_DIRECT	GO:0006974~cellular response to DNA damage stimulus	40	1.72	0.006
GOTERM_BP_DIRECT	GO:0042276~error-prone translesion synthesis	8	0.34	0.006
GOTERM_BP_DIRECT	GO:0042148~strand invasion	5	0.22	0.006
GOTERM_BP_DIRECT	GO:0046826~negative regulation of protein export from nucleus	5	0.22	0.006
GOTERM_BP_DIRECT	GO:1900264~positive regulation of DNA-directed DNA polymerase activity	5	0.22	0.006
GOTERM_BP_DIRECT	GO:0000707~meiotic DNA recombinase assembly	4	0.17	0.007
GOTERM_BP_DIRECT	GO:0043488~regulation of mRNA stability	23	0.99	0.008
GOTERM_BP_DIRECT	GO:0006298~mismatch repair	11	0.47	0.009
GOTERM_BP_DIRECT	GO:0000375~RNA splicing, via transesterification reactions	9	0.39	0.009
GOTERM_BP_DIRECT	GO:0000070~mitotic sister chromatid segregation	9	0.39	0.009
GOTERM_BP_DIRECT	GO:0000077~DNA damage checkpoint	10	0.43	0.009
GOTERM_BP_DIRECT	GO:0007051~spindle organization	7	0.30	0.010
GOTERM_BP_DIRECT	GO:0042769~DNA damage response, detection of DNA damage	11	0.47	0.011
GOTERM_BP_DIRECT	GO:0042254~ribosome biogenesis	11	0.47	0.011
GOTERM_BP_DIRECT	GO:0031100~organ regeneration	13	0.56	0.011
GOTERM_BP_DIRECT	GO:0072321~chaperone-mediated protein transport	5	0.22	0.011
GOTERM_BP_DIRECT	GO:1904871~positive regulation of protein localization to Cajal body	5	0.22	0.011
GOTERM_BP_DIRECT	GO:0034551~mitochondrial respiratory chain complex III assembly	5	0.22	0.011
GOTERM_BP_DIRECT	GO:0085020~protein K6-linked ubiquitination	5	0.22	0.011
GOTERM_BP_DIRECT	GO:0008334~histone mRNA metabolic process	6	0.26	0.011
GOTERM_BP_DIRECT	GO:0000245~spliceosomal complex assembly	9	0.39	0.011
GOTERM_BP_DIRECT	GO:0045930~negative regulation of mitotic cell cycle	9	0.39	0.011
GOTERM_BP_DIRECT	GO:0016571~histone methylation	7	0.30	0.014
GOTERM_BP_DIRECT	GO:0032212~positive regulation of telomere maintenance via telomerase	10	0.43	0.014
GOTERM_BP_DIRECT	GO:0006400~tRNA modification	9	0.39	0.015
GOTERM_BP_DIRECT	GO:0006302~double-strand break repair	16	0.69	0.015
GOTERM_BP_DIRECT	GO:0006221~pyrimidine nucleotide biosynthetic process	4	0.17	0.016
GOTERM_BP_DIRECT	GO:0090502~RNA phosphodiester bond hydrolysis, endonucleolytic	14	0.60	0.016
GOTERM_BP_DIRECT	GO:0000460~maturation of 5.8S rRNA	6	0.26	0.016
GOTERM_BP_DIRECT	GO:0032259~methylation	17	0.73	0.017
GOTERM_BP_DIRECT	GO:0007077~mitotic nuclear envelope disassembly	12	0.52	0.017
GOTERM_BP_DIRECT	GO:0000463~maturation of LSU-rRNA from tricistronic rRNA transcript (SSU-rRNA, 5.8S rRNA, LSU-rRNA)	5	0.22	0.018
GOTERM_BP_DIRECT	GO:0045662~negative regulation of myoblast differentiation	8	0.34	0.019
GOTERM_BP_DIRECT	GO:0006977~DNA damage response, signal transduction by p53 class mediator resulting in cell cycle arrest	15	0.65	0.019
GOTERM_BP_DIRECT	GO:0051321~meiotic cell cycle	10	0.43	0.021
GOTERM_BP_DIRECT	GO:0030490~maturation of SSU-rRNA	6	0.26	0.023
GOTERM_BP_DIRECT	GO:0090263~positive regulation of canonical Wnt signaling pathway	24	1.03	0.023

GOTERM_BP_DIRECT	GO:0006297~nucleotide-excision repair, DNA gap filling	8	0.34	0.024
GOTERM_BP_DIRECT	GO:0070987~error-free translesion synthesis	7	0.30	0.024
GOTERM_BP_DIRECT	GO:0045739~positive regulation of DNA repair	9	0.39	0.027
GOTERM_BP_DIRECT	GO:0007131~reciprocal meiotic recombination	9	0.39	0.027
GOTERM_BP_DIRECT	GO:0061351~neural precursor cell proliferation	5	0.22	0.027
GOTERM_BP_DIRECT	GO:0051085~chaperone mediated protein folding requiring cofactor	5	0.22	0.027
GOTERM_BP_DIRECT	GO:0043985~histone H4-R3 methylation	4	0.17	0.029
GOTERM_BP_DIRECT	GO:0006360~transcription from RNA polymerase I promoter	4	0.17	0.029
GOTERM_BP_DIRECT	GO:0006415~translational termination	4	0.17	0.029
GOTERM_BP_DIRECT	GO:0006913~nucleocytoplasmic transport	8	0.34	0.029
GOTERM_BP_DIRECT	GO:0042026~protein refolding	6	0.26	0.031
GOTERM_BP_DIRECT	GO:0045190~isotype switching	6	0.26	0.031
GOTERM_BP_DIRECT	GO:0001649~osteoblast differentiation	21	0.90	0.032
GOTERM_BP_DIRECT	GO:0050679~positive regulation of epithelial cell proliferation	14	0.60	0.032
GOTERM_BP_DIRECT	GO:0006413~translational initiation	26	1.12	0.032
GOTERM_BP_DIRECT	GO:0006296~nucleotide-excision repair, DNA incision, 5'-to lesion	10	0.43	0.035
GOTERM_BP_DIRECT	GO:0001523~retinoid metabolic process	14	0.60	0.036
GOTERM_BP_DIRECT	GO:0071897~DNA biosynthetic process	8	0.34	0.036
GOTERM_BP_DIRECT	GO:0006336~DNA replication-independent nucleosome assembly	8	0.34	0.036
GOTERM_BP_DIRECT	GO:0010212~response to ionizing radiation	12	0.52	0.036
GOTERM_BP_DIRECT	GO:0031284~positive regulation of guanylate cyclase activity	5	0.22	0.039
GOTERM_BP_DIRECT	GO:0051292~nuclear pore complex assembly	5	0.22	0.039
GOTERM_BP_DIRECT	GO:0001682~tRNA 5'-leader removal	5	0.22	0.039
GOTERM_BP_DIRECT	GO:0010763~positive regulation of fibroblast migration	5	0.22	0.039
GOTERM_BP_DIRECT	GO:0071480~cellular response to gamma radiation	7	0.30	0.039
GOTERM_BP_DIRECT	GO:0006335~DNA replication-dependent nucleosome assembly	9	0.39	0.039
GOTERM_BP_DIRECT	GO:0006409~tRNA export from nucleus	9	0.39	0.039
GOTERM_BP_DIRECT	GO:0000381~regulation of alternative mRNA splicing, via spliceosome	10	0.43	0.041
GOTERM_BP_DIRECT	GO:0007623~circadian rhythm	16	0.69	0.042
GOTERM_BP_DIRECT	GO:1904117~cellular response to vasopressin	3	0.13	0.043
GOTERM_BP_DIRECT	GO:0000453~enzyme-directed rRNA 2'-O-methylation	3	0.13	0.043
GOTERM_BP_DIRECT	GO:0031627~telomeric loop formation	3	0.13	0.043
GOTERM_BP_DIRECT	GO:1904667~negative regulation of ubiquitin protein ligase activity	3	0.13	0.043
GOTERM_BP_DIRECT	GO:0042780~tRNA 3'-end processing	3	0.13	0.043
GOTERM_BP_DIRECT	GO:0030308~negative regulation of cell growth	23	0.99	0.044
GOTERM_BP_DIRECT	GO:0002479~antigen processing and presentation of exogenous peptide antigen via MHC class I, TAP-dependent	14	0.60	0.045
GOTERM_BP_DIRECT	GO:0030336~negative regulation of cell migration	19	0.82	0.045
GOTERM_BP_DIRECT	GO:0031848~protection from non-homologous end joining at telomere	4	0.17	0.046
GOTERM_BP_DIRECT	GO:0043305~negative regulation of mast cell degranulation	4	0.17	0.046
GOTERM_BP_DIRECT	GO:0019919~peptidyl-arginine methylation, to asymmetrical-dimethyl arginine	4	0.17	0.046
GOTERM_BP_DIRECT	GO:0001561~fatty acid alpha-oxidation	4	0.17	0.046
GOTERM_BP_DIRECT	GO:0060687~regulation of branching involved in prostate gland morphogenesis	4	0.17	0.046
GOTERM_BP_DIRECT	GO:0051297~centrosome organization	9	0.39	0.046
GOTERM_BP_DIRECT	GO:0008285~negative regulation of cell proliferation	62	2.67	0.047
GOTERM_BP_DIRECT	GO:0045668~negative regulation of osteoblast differentiation	10	0.43	0.047
GOTERM_BP_DIRECT	GO:0042795~snRNA transcription from RNA polymerase II promoter	15	0.65	0.049
GOTERM_BP_DIRECT	GO:0050821~protein stabilization	25	1.08	0.050
