# Supplementary material for: Differentiated embryo chondrocyte plays a crucial role in DNA damage response via transcriptional regulation under hypoxic conditions
Source: PLoS One. 2018 Feb 21;13(2):e0192136. doi: 10.1371/journal.pone.0192136 (PMC5821451; doi:10.1371/journal.pone.0192136)
Supplement: S4 Table — (PDF) [file pone.0192136.s004.pdf]

S4 Table. Expressions of DNA-DRR genes under normoxic or hypoxic conditions for 24 hours in HSC-2 cells.

No	Gene	Normoxia	24hr hypoxia	Fold	Accession #
1	ALKBH3 (DEPC-1, ABH3)	1.01	0.99	0.99	NM_139178
2	APEX1	0.72	0.28	0.39	M92444
3	APEX2	0.83	0.44	0.54	NM_014481
4	APTX (aprataxin)	0.97	0.60	0.62	NM_017692
5	ATM	1.57	1.21	0.77	U82828
		2.34	2.17	0.93	U82828
		1.08	1.08	1.00	U33841
		0.87	1.03	1.19	U82828
		1.48	2.12	1.43	NM_000051
6	ATR	2.80	1.15	0.41	NM_001184
7	ATRIP (TREX1)	0.96	0.40	0.41	NM_016381
		1.04	0.72	0.69	NM_016381
8	BLM	0.91	0.34	0.38	NM_000057
9	BRCA1	0.96	0.39	0.40	NM_007303
10	BRCA2	1.18	1.01	0.86	NM_000059
11	CCNH	3.22	1.77	0.55	NM_001239
		0.27	0.56	2.04	AF147385
12	CDK7	1.21	0.99	0.82	NM_001799
13	CETN2	0.66	0.66	1.00	NM_004344
14	CHAF1A (CAF1)	0.70	0.22	0.31	NM_005483
15	CHEK1	1.07	0.52	0.48	NM_001274
16	CHEK2	1.49	0.96	0.64	NM_007194
17	DCLRE1A (SNM1)	0.62	0.34	0.55	D42045
18	DCLRE1B (SNM1B)	1.25	0.71	0.57	NM_022836
19	DCLRE1C (Artemis)	0.93	0.94	1.00	NM_022487
20	DDB1	1.53	1.68	1.10	L40326
		0.81	1.14	1.41	NM_001923
21	DDB2	1.00	1.38	1.37	NM_000107
22	DMC1	0.01	0.01	1.00	NM_007068
23	DUT	0.76	0.48	0.63	NM_001948
24	ENDOV (FLJ35220)	0.73	0.71	0.97	AK056045
25	ERCC1	0.82	0.74	0.90	NM_001983
		0.82	0.90	1.10	BM556010
26	ERCC2 (XPD)	0.62	0.59	0.94	NM_000400
27	ERCC3 (XPB)	0.88	0.74	0.83	NM_000122
28	ERCC4 (XPF)	1.02	0.87	0.86	NM_005236
		1.12	1.02	0.91	L76568
29	ERCC5 (XPG)	1.09	1.62	1.49	NM_000123
30	ERCC6 (CSB)	0.59	1.24	2.11	NM_000124
31	ERCC8, (CKN1, CSA)	0.63	0.40	0.63	NM_000082
32	EXO1 (HEX1)	0.62	0.23	0.36	NM_003686
33	FANCA	1.26	0.91	0.73	NM_000135
34	FANCC	0.74	0.42	0.57	BC006303
		1.03	0.62	0.60	BC006303
		0.64	0.56	0.87	NM_000136
35	FANCE	1.09	0.82	0.75	NM_021922
36	FANCF	0.82	0.71	0.86	NM_022725
37	FANCG (XRCC9)	0.96	1.03	1.06	NM_004629
38	FEN1 (DNase IV)	0.86	0.32	0.37	NM_004111
39	GTF2H1	0.88	0.68	0.77	NM_005316
40	GTF2H2	0.87	0.46	0.52	NM_001515
41	GTF2H3	1.51	1.18	0.78	NM_001516
42	GTF2H4	0.64	0.46	0.72	NM_001517
43	H2AFX (H2AX)	1.20	0.48	0.41	NM_002105
44	HEL308	0.69	0.69	0.99	NM_133636
45	HUS1	1.15	0.99	0.86	NM_004507
46	LIG1	0.91	0.57	0.62	NM_000234
		1.24	0.80	0.65	AB050468
47	LIG3	1.31	0.94	0.72	NM_013975
		1.06	0.82	0.77	NM_013975
48	LIG4	0.67	0.80	1.19	NM_002312
49	MAD2L2 (REV7)	0.74	0.40	0.53	NM_006341
		1.08	1.29	1.19	AK027327
50	MBD4	1.55	0.82	0.53	NM_003925

51	MGMT	0.71	0.78	1.10	NM_002412
52	MLH1	1.47	0.96	**0.65**	NM_000249
53	MLH3	0.54	0.62	1.14	AF195658
		0.85	1.46	1.73	NM_014381
54	MMS19L (MMS19)	0.78	1.02	1.31	NM_022362
55	MNAT1	0.61	0.71	1.16	NM_002431
56	MPG	0.56	0.60	1.06	NM_002434
57	MRE11A	2.25	1.36	**0.61**	NM_005590
58	MSH2	0.58	0.25	**0.43**	NM_000251
59	MSH3	0.90	0.82	0.91	NM_002439
60	MSH4	0.44	0.74	1.67	NM_002440
61	MSH5	0.91	0.69	0.76	NM_002441
62	MSH6	1.13	1.02	0.90	NM_000179
63	MUS81	0.93	0.97	1.04	NM_025128
64	MUTYH (MYH)	0.58	0.28	**0.48**	NM_012222
65	NBN (NBS1)	0.92	0.52	**0.57**	NM_002485
65	NBN (NBS1)	0.74	0.46	**0.61**	AF058696
66	NEIL1	0.20	0.41	2.06	NM_024608
67	NTHL1 (NTH1)	0.66	0.26	**0.40**	NM_002528
68	NUDT1 (MTH1)	1.15	0.92	0.80	NM_002452
69	OGG1	1.13	0.91	0.81	NM_002542
70	PARP1 (ADPRT)	0.88	0.42	**0.48**	NM_001618
71	PARP2 (ADPRTL2)	0.51	0.41	0.81	NM_005484
72	PCNA	1.32	0.38	**0.29**	NM_002592
73	PMS1	1.15	0.49	**0.43**	NM_000534
74	PMS2	0.08	0.76	9.32	NM_000535
75	PMS2L4 (PMS6)	0.80	0.92	1.15	D38438
76	PNKP	0.81	0.76	0.94	NM_007254
77	POLD1	0.25	0.29	1.12	NM_002691
78	POLE	0.93	0.89	0.95	NM_006231
79	POLG	0.88	1.07	1.22	NM_002693
80	POLH	1.21	0.93	0.77	NM_006502
81	POLI (RAD30B)	0.52	1.31	2.50	NM_007195
82	POLK (DINB1)	1.29	1.54	1.19	NM_016218
83	POLL	1.19	0.79	**0.66**	NM_013274
84	POLM	0.88	0.78	0.89	NM_013284
85	POLQ	1.25	0.58	**0.47**	NM_006596
86	PRKDC	1.53	1.07	0.70	U47077
		1.32	1.02	0.77	NM_006904
87	RAD1	0.80	0.48	**0.60**	NM_002853
88	RAD17 (RAD24)	0.87	0.86	0.99	NM_002873
89	RAD18	1.38	0.68	**0.49**	NM_020165
90	RAD23A (HR23A)	0.11	0.48	4.59	NM_005053
91	RAD23B (HR23B)	0.68	0.77	1.13	NM_002874
92	RAD50	1.41	1.37	0.97	NM_005732
93	RAD51	0.74	0.38	**0.51**	NM_002875
94	RAD51L1 (RAD51B)	1.00	0.59	**0.59**	NM_002877
95	RAD51L3 (RAD51D)	0.77	0.41	**0.53**	NM_002878
96	RAD52	0.91	1.09	1.20	NM_002879
		0.90	1.71	1.89	U12134
97	RAD54B	0.66	0.49	0.74	NM_012415
98	RAD54L	0.98	0.44	**0.45**	NM_003579
99	RECQL (RECQ1)	0.60	0.72	1.20	NM_002907
100	RECQL4	1.04	1.15	1.10	NM_004260
101	RECQL5	0.72	0.42	**0.59**	NM_004259
		1.14	1.03	0.91	AB042823
101	RECQL5	0.78	0.95	1.22	AB042824
102	REV1L (REV1)	1.00	1.01	1.01	AK025176
		1.01	1.06	1.05	NM_016316
103	REV3L (POLZ)	0.76	0.77	1.01	NM_002912
104	RPA1	0.70	0.70	1.00	NM_002945
105	RPA2	1.22	0.57	**0.47**	NM_002946
106	RPA3	1.75	1.01	**0.58**	NM_002947
107	RRM2B (p53R2)	0.36	0.26	0.73	AB036063
108	SHFM1 (DSS1)	0.86	0.81	0.95	NM_006304
109	SMUG1	0.73	0.65	0.90	NM_014311
		1.07	1.20	1.12	AK001789
110	SPO11	1.01	0.99	0.99	NM_012444

111	TDG	0.63	0.55	0.87	NM_003211
112	TDP1	0.66	0.68	1.02	NM_018319
113	TP53	0.55	0.39	0.70	NM_000546
114	TREX1 (DNase III)	0.96	0.40	**0.41**	NM_016381
		1.04	0.72	0.69	NM_016381
115	TREX2	0.60	0.24	**0.40**	NM_017518
		1.29	1.13	0.88	AL122036
116	UBE2A (RAD6A)	1.22	0.98	0.80	NM_003336
117	UBE2B (RAD6B)	0.04	0.79	18.73	NM_003337
118	UBE2N (UBC13)	0.84	0.75	0.89	NM_003348
119	UBE2V2 (MMS2)	0.91	1.55	1.70	NM_003350
120	UNG	0.43	0.15	**0.36**	NM_003362
121	WRN	1.05	0.57	**0.55**	AF181897
		1.40	1.39	0.99	NM_000553
		0.31	0.84	2.69	AF181896
122	XAB2 (HCNP)	0.97	0.57	**0.59**	NM_020196
123	XPA	1.32	1.01	0.76	NM_000380
124	XPC	1.23	1.08	0.88	AF261901
		0.98	1.02	1.03	NM_004628
125	XRCC1	0.61	0.72	1.19	NM_006297
126	XRCC2	1.22	0.43	**0.35**	NM_005431
127	XRCC3	1.03	0.41	**0.40**	NM_005432
128	XRCC4	0.08	0.16	2.03	NM_003401
129	XRCC5 (Ku80)	0.84	0.72	0.86	NM_021141
130	XRCC6, (G22P1, Ku70)	0.86	0.72	0.84	NM_001469
		1.22	1.10	0.90	AF052148
