# Supplementary material for: Differentiated embryo chondrocyte plays a crucial role in DNA damage response via transcriptional regulation under hypoxic conditions
Source: PLoS One. 2018 Feb 21;13(2):e0192136. doi: 10.1371/journal.pone.0192136 (PMC5821451; doi:10.1371/journal.pone.0192136)
Supplement: S5 Table — (PDF) [file pone.0192136.s005.pdf]

S5 Table. List of the NCBI Gene Expression Omnibus database analyzed in this study.

	Hypoxia	Normoxia	Cancer or non-cancer	Cell line name	Notes	Experiment	
GSE17353	GSM433798	GSM433796	non-cancer	EPC2	immortal-epithelium	1%	24h
	GSM433799	GSM433797	non-cancer	EPC2	immortal-epithelium	1%	24h
	GSM433802	GSM433800	non-cancer	EPC2	immortal-epithelium	0.20%	24h
	GSM433803	GSM433801	non-cancer	EPC2	immortal-epithelium	0.20%	24h
GSE19197	GSM475953	GSM475952	cancer	sknmc	sarcoma	1%	24h
	GSM475955	GSM475954	cancer	tc252	sarcoma	1%	24h
GSE29641	GSM734522	GSM734515	cancer	DU145	prostate	0%	24h
	GSM734530	GSM734523	cancer	HT29	colorectal	0%	24h
	GSM734538	GSM734531	cancer	MCF7	breast	0%	24h
GSE4086	GSM93566	GSM93568	cancer	B-lymphocyte	lymphoma	0.10%	29h
	GSM93567	GSM93569	cancer	B-lymphocyte	lymphoma	0.10%	29h
GSE9234	GSM234659	GSM234656	cancer	HT29	colorectal		
	GSM234660	GSM234657	cancer	HT29	colorectal		
	GSM234661	GSM234658	cancer	HT29	colorectal		
GSE9649	GSM243736	GSM243730	non-cancer	HMEC	mammary epithelium	2%	24h
	GSM243737	GSM243731	non-cancer	HMEC	mammary epithelium	2%	24h
	GSM243738	GSM243732	non-cancer	HMEC	mammary epithelium	2%	24h
GSE39042	GSM954536	GSM954535	cancer	MDA-MB-231	breast	1%	16h
	GSM954542	GSM954541	cancer	MDA-MB-231	breast	1%	16h
	GSM954548	GSM954547	cancer	MDA-MB-231	breast	1%	16h
GSE50378	GSM1217731	GSM1217732	non-cancer	HUVEC	vein endothelium	1%	24h
GSE58049	GSM1400089	GSM1400088	cancer	HCT116	colorectal		
	GSM1400093	GSM1400092	cancer	HCT116	colorectal		
	GSM1400094	GSM1400096	cancer	HCT116	colorectal		
GSE52315	GSM1262783	GSM1262782	cancer	MM1S	lymphoblast		24h
	GSM1262786	GSM1262784	cancer	MM1S	lymphoblast		24h
	GSM1262787	GSM1262785	cancer	MM1S	lymphoblast		24h
GSE46054	GSM1122648	GSM1122645	cancer	Hela	cervix	1%	24h
GSE33115	GSM820138	GSM820143	non-cancer	HUVEC	vein endothelium		12h
	GSM820139	GSM820144	non-cancer	HUVEC	vein endothelium		12h
GSE53012	GSM1280332	GSM1280335	cancer	PC-3	prostate	1%	72h
	GSM1280333	GSM1280336	cancer	PC-3	prostate	1%	72h
	GSM1280334	GSM1280337	cancer	PC-3	prostate	1%	72h
	GSM1280341	GSM1280344	cancer	SK-OV-3	ovary	1%	72h
	GSM1280342	GSM1280345	cancer	SK-OV-3	ovary	1%	72h
	GSM1280343	GSM1280346	cancer	SK-OV-3	ovary	1%	72h
	GSM1280350	GSM1280353	cancer	WM793B		1%	72h
	GSM1280351	GSM1280354	cancer	WM793B		1%	72h
	GSM1280352	GSM1280355	cancer	WM793B		1%	72h
