# Supplementary material for: Differentiated embryo chondrocyte plays a crucial role in DNA damage response via transcriptional regulation under hypoxic conditions
Source: PLoS One. 2018 Feb 21;13(2):e0192136. doi: 10.1371/journal.pone.0192136 (PMC5821451; doi:10.1371/journal.pone.0192136)
Supplement: S6 Table — (PDF) [file pone.0192136.s006.pdf]

S6 Table. Hypoxic down-regulation of DNA-DRR genes in cancer and non-cancer cells analyzed using the NCBI Gene Expression Omnibus database.

No	ID	In cancer cells UP	DOWN	UN	In Non-cancer cells UP	DOWN	UN	Gene Symbol	Description
1	223758_s_at	0	23	6	1	6	3	GTF2H2	general transcription factor IIH, polypeptide 2, 44kDa [Source:HGNC Symbol;Acc:4656]
2	223758_s_at	0	23	6	1	6	3	GTF2H2C	general transcription factor IIH, polypeptide 2C [Source:HGNC Symbol;Acc:31394]
3	227766_at	0	21	8	0	6	4	LIG4	ligase IV, DNA, ATP-dependent [Source:HGNC Symbol;Acc:6601]
4	209903_s_at	0	20	9	0	5	5	ATR	ataxia telangiectasia and Rad3 related [Source:HGNC Symbol;Acc:882]
5	209902_at	0	19	10	1	5	4	ATR	ataxia telangiectasia and Rad3 related [Source:HGNC Symbol;Acc:882]
6	223511_at	0	19	10	1	5	4	SPRTN	SprT-like N-terminal domain [Source:HGNC Symbol;Acc:25356]
7	204531_s_at	1	19	9	0	4	6	BRCA1	breast cancer 1, early onset [Source:HGNC Symbol;Acc:1100]
8	222889_at	0	19	10	0	3	7	DCLRE1B	DNA cross-link repair 1B [Source:HGNC Symbol;Acc:17641]
9	204768_s_at	2	19	8	1	2	7	FEN1	flap structure-specific endonuclease 1 [Source:HGNC Symbol;Acc:3650]
10	201202_at	1	19	9	0	2	8	PCNA	proliferating cell nuclear antigen [Source:HGNC Symbol;Acc:8729]
11	209804_at	0	19	10	0	1	9	DCLRE1A	DNA cross-link repair 1A [Source:HGNC Symbol;Acc:17660]
12	230177_at	0	18	11	0	7	3	GTF2H2C	general transcription factor IIH, polypeptide 2C [Source:HGNC Symbol;Acc:31394]
13	206235_at	3	18	8	1	5	4	LIG4	ligase IV, DNA, ATP-dependent [Source:HGNC Symbol;Acc:6601]
14	209421_at	0	18	11	1	5	4	MSH2	mutS homolog 2, colon cancer, nonpolyposis type 1 (E. coli) [Source:HGNC Symbol;Acc:7325]
15	209849_s_at	0	18	11	0	4	6	RAD51C	RAD51 homolog C (S. cerevisiae) [Source:HGNC Symbol;Acc:9820]
16	204767_s_at	1	18	10	1	1	8	FEN1	flap structure-specific endonuclease 1 [Source:HGNC Symbol;Acc:3650]
17	201523_x_at	0	18	11	0	1	9	UBE2N	ubiquitin-conjugating enzyme E2N [Source:HGNC Symbol;Acc:12492]
18	222233_s_at	0	17	12	0	6	4	DCLRE1C	DNA cross-link repair 1C [Source:HGNC Symbol;Acc:17642]
19	213007_at	1	17	11	0	4	6	FANCI	Fanconi anemia, complementation group I [Source:HGNC Symbol;Acc:25568]
20	206066_s_at	0	17	12	1	4	5	RAD51C	RAD51 homolog C (S. cerevisiae) [Source:HGNC Symbol;Acc:9820]
21	202330_s_at	4	17	8	1	2	7	UNG	uracil-DNA glycosylase [Source:HGNC Symbol;Acc:12572]
22	205394_at	3	17	9	0	1	9	CHEK1	checkpoint kinase 1 [Source:HGNC Symbol;Acc:1925]
23	205162_at	0	17	12	0	0	10	ERCC8	excision repair cross-complementing rodent repair deficiency, complementation group 8 [Source:HGNC Symbol;Acc:3439]
24	235215_at	1	16	12	0	5	5	ERCC4	excision repair cross-complementing rodent repair deficiency, complementation group 4 [Source:HGNC Symbol;Acc:3436]
25	202907_s_at	0	16	13	1	5	4	NBN	nibrin [Source:HGNC Symbol;Acc:7652]
26	212751_at	0	16	13	0	5	5	UBE2N	ubiquitin-conjugating enzyme E2N [Source:HGNC Symbol;Acc:12492]
27	208694_at	1	16	12	0	3	7	PRKDC	protein kinase, DNA-activated, catalytic polypeptide [Source:HGNC Symbol;Acc:9413]
28	204603_at	2	16	11	0	2	8	EXO1	exonuclease 1 [Source:HGNC Symbol;Acc:3511]
29	235609_at	4	16	9	0	1	9	BRIP1	BRCA1 interacting protein C-terminal helicase 1 [Source:HGNC Symbol;Acc:20473]
30	218689_at	0	16	13	0	1	9	FANCF	Fanconi anemia, complementation group F [Source:HGNC Symbol;Acc:3587]
31	223785_at	2	16	11	1	1	8	FANCI	Fanconi anemia, complementation group I [Source:HGNC Symbol;Acc:25568]
32	206554_x_at	0	16	13	0	1	9	SETMAR	SET domain and mariner transposase fusion gene [Source:HGNC Symbol;Acc:10762]
33	205393_s_at	4	16	9	1	0	9	CHEK1	checkpoint kinase 1 [Source:HGNC Symbol;Acc:1925]
34	221540_x_at	0	15	14	0	6	4	GTF2H2	general transcription factor IIH, polypeptide 2, 44kDa [Source:HGNC Symbol;Acc:4656]
35	221540_x_at	0	15	14	0	6	4	GTF2H2C	general transcription factor IIH, polypeptide 2C [Source:HGNC Symbol;Acc:31394]
36	208643_s_at	0	15	14	1	5	4	XRCC5	X-ray repair complementing defective repair in Chinese hamster cells 5 (double-strand-break rejoining) [Source:HGNC Symbol;Acc:12833]
37	208955_at	2	15	12	0	4	6	DUT	deoxyuridine triphosphatase [Source:HGNC Symbol;Acc:3078]
38	239346_at	0	15	14	0	4	6	GTF2H3	general transcription factor IIH, polypeptide 3, 34kDa [Source:HGNC Symbol;Acc:4657]
39	219530_at	0	15	14	0	4	6	PALB2	partner and localizer of BRCA2 [Source:HGNC Symbol;Acc:26144]
40	205733_at	0	15	14	0	3	7	BLM	Bloom syndrome, RecQ helicase-like [Source:HGNC Symbol;Acc:1058]
41	202633_at	0	15	14	0	3	7	TOPBP1	topoisomerase (DNA) II binding protein 1 [Source:HGNC Symbol;Acc:17008]
42	211851_x_at	0	15	14	0	1	9	BRCA1	breast cancer 1, early onset [Source:HGNC Symbol;Acc:1100]
43	238075_at	1	15	13	0	0	10	CHEK1	checkpoint kinase 1 [Source:HGNC Symbol;Acc:1925]
44	205436_s_at	0	15	14	1	0	9	H2AFX	H2A histone family, member X [Source:HGNC Symbol;Acc:4739]
45	213677_s_at	0	14	15	1	5	4	PMS1	PMS1 postmeiotic segregation increased 1 (S. cerevisiae) [Source:HGNC Symbol;Acc:9121]
46	202520_s_at	0	14	15	0	4	6	MLH1	mutL homolog 1, colon cancer, nonpolyposis type 2 (E. coli) [Source:HGNC Symbol;Acc:7127]
47	205024_s_at	1	14	14	0	3	7	RAD51	RAD51 homolog (S. cerevisiae) [Source:HGNC Symbol;Acc:9817]
48	208644_at	0	14	15	0	2	8	PARP1	poly (ADP-ribose) polymerase 1 [Source:HGNC Symbol;Acc:270]
49	214426_x_at	0	14	15	0	1	9	CHAF1A	chromatin assembly factor 1, subunit A (p150) [Source:HGNC Symbol;Acc:1910]
50	218397_at	1	14	14	0	1	9	FANCL	Fanconi anemia, complementation group L [Source:HGNC Symbol;Acc:20748]
51	201524_x_at	0	14	15	0	0	10	UBE2N	ubiquitin-conjugating enzyme E2N [Source:HGNC Symbol;Acc:12492]
52	228286_at	0	13	16	0	4	6	GEN1	Gen endonuclease homolog 1 (Drosophila) [Source:HGNC Symbol;Acc:26881]
53	203344_s_at	0	13	16	0	4	6	RBBP8	retinoblastoma binding protein 8 [Source:HGNC Symbol;Acc:9891]
54	1554883_a_at	0	13	16	0	3	7	ERCC8	excision repair cross-complementing rodent repair deficiency, complementation group 8 [Source:HGNC Symbol;Acc:3439]
55	225625_at	0	13	16	0	2	8	ALKBH2	alkB, alkylation repair homolog 2 (E. coli) [Source:HGNC Symbol;Acc:32487]
56	205395_s_at	2	13	14	0	2	8	MRE11A	MRE11 meiotic recombination 11 homolog A (S. cerevisiae) [Source:HGNC Symbol;Acc:7230]
57	205301_s_at	0	13	16	0	2	8	OGG1	8-oxoguanine DNA glycosylase [Source:HGNC Symbol;Acc:8125]
58	203103_s_at	0	13	16	0	2	8	PRPF19	PRP19/PSO4 pre-mRNA processing factor 19 homolog (S. cerevisiae) [Source:HGNC Symbol;Acc:17896]
59	214727_at	1	13	15	0	1	9	BRCA2	breast cancer 2, early onset [Source:HGNC Symbol;Acc:1101]
60	203976_s_at	0	13	16	0	1	9	CHAF1A	chromatin assembly factor 1, subunit A (p150) [Source:HGNC Symbol;Acc:1910]
61	208956_x_at	2	13	14	0	0	10	DUT	deoxyuridine triphosphatase [Source:HGNC Symbol;Acc:3078]
62	209932_s_at	0	13	16	0	0	10	DUT	deoxyuridine triphosphatase [Source:HGNC Symbol;Acc:3078]
63	202451_at	0	12	17	2	5	3	GTF2H1	general transcription factor IIH, polypeptide 1, 62kDa [Source:HGNC Symbol;Acc:4655]
64	224200_s_at	2	12	15	0	4	6	RAD18	RAD18 homolog (S. cerevisiae) [Source:HGNC Symbol;Acc:18278]

65	205667_at	0	12	17	0	3	7	WRN	Werner syndrome, RecQ helicase-like [Source:HGNC Symbol;Acc:12791]
66	218527_at	0	12	17	0	2	8	APTX	aprataxin [Source:HGNC Symbol;Acc:15984]
67	219502_at	2	12	15	0	2	8	NEIL3	nei endonuclease VIII-like 3 (E. coli) [Source:HGNC Symbol;Acc:24573]
68	209965_s_at	0	12	17	0	1	9	RAD51D	RAD51 homolog D (S. cerevisiae) [Source:HGNC Symbol;Acc:9823]
69	213008_at	3	12	14	0	0	10	FANCI	Fanconi anemia, complementation group I [Source:HGNC Symbol;Acc:25568]
70	234733_s_at	0	12	17	1	0	9	FANCM	Fanconi anemia, complementation group M [Source:HGNC Symbol;Acc:23168]
71	242560_at	1	11	17	0	5	5	FANCD2	Fanconi anemia, complementation group D2 [Source:HGNC Symbol;Acc:3585]
72	236620_at	3	11	15	0	4	6	RIF1	RAP1 interacting factor homolog (yeast) [Source:HGNC Symbol;Acc:23207]
73	228736_at	0	11	18	0	2	8	HELQ	helicase, POLQ-like [Source:HGNC Symbol;Acc:18536]
74	201756_at	0	11	18	0	2	8	RPA2	replication protein A2, 32kDa [Source:HGNC Symbol;Acc:10290]
75	222713_s_at	1	11	17	0	1	9	FANCF	Fanconi anemia, complementation group F [Source:HGNC Symbol;Acc:3587]
76	219494_at	6	11	12	2	1	7	RAD54B	RAD54 homolog B (S. cerevisiae) [Source:HGNC Symbol;Acc:17228]
77	203975_s_at	1	11	17	0	0	10	CHAF1A	chromatin assembly factor 1, subunit A (p150) [Source:HGNC Symbol;Acc:1910]
78	235478_at	1	11	17	0	0	10	DCLRE1C	DNA cross-link repair 1C [Source:HGNC Symbol;Acc:17642]
79	207727_s_at	0	11	18	0	0	10	MUTYH	mutY homolog (E. coli) [Source:HGNC Symbol;Acc:7527]
80	214700_x_at	2	10	17	0	4	6	RIF1	RAP1 interacting factor homolog (yeast) [Source:HGNC Symbol;Acc:23207]
81	202453_s_at	1	10	18	1	3	6	GTF2H1	general transcription factor IIH, polypeptide 1, 62kDa [Source:HGNC Symbol;Acc:4655]
82	208368_s_at	4	10	15	0	2	8	BRCA2	breast cancer 2, early onset [Source:HGNC Symbol;Acc:1101]
83	209349_at	0	10	19	1	2	7	RAD50	RAD50 homolog (S. cerevisiae) [Source:HGNC Symbol;Acc:9816]
84	226503_at	0	10	19	0	2	8	RIF1	RAP1 interacting factor homolog (yeast) [Source:HGNC Symbol;Acc:23207]
85	207891_s_at	0	10	19	0	2	8	TREX2	three prime repair exonuclease 2 [Source:HGNC Symbol;Acc:12270]
86	205072_s_at	3	10	16	1	2	7	XRCC4	X-ray repair complementing defective repair in Chinese hamster cells 4 [Source:HGNC Symbol;Acc:12831]
87	203806_s_at	0	10	19	0	1	9	FANCA	Fanconi anemia, complementation group A [Source:HGNC Symbol;Acc:3582]
88	1557218_s_at	1	10	18	0	1	9	FANCB	Fanconi anemia, complementation group B [Source:HGNC Symbol;Acc:3583]
89	223417_at	1	10	18	0	1	9	RAD18	RAD18 homolog (S. cerevisiae) [Source:HGNC Symbol;Acc:18278]
90	209507_at	2	10	17	0	1	9	RPA3	replication protein A3, 14kDa [Source:HGNC Symbol;Acc:10291]
91	213334_x_at	0	10	19	0	1	9	TREX2	three prime repair exonuclease 2 [Source:HGNC Symbol;Acc:12270]
92	226366_at	0	9	20	1	6	3	SHPRH	SNF2 histone linker PHD RING helicase, E3 ubiquitin protein ligase [Source:HGNC Symbol;Acc:19336]
93	210543_s_at	0	9	20	1	5	4	PRKDC	protein kinase, DNA-activated, catalytic polypeptide [Source:HGNC Symbol;Acc:9413]
94	208070_s_at	1	9	19	1	5	4	REV3L	REV3-like, polymerase (DNA directed), zeta, catalytic subunit [Source:HGNC Symbol;Acc:9968]
95	1554277_s_at	1	9	19	0	2	8	FANCM	Fanconi anemia, complementation group M [Source:HGNC Symbol;Acc:23168]
96	1557217_a_at	1	9	19	0	1	9	FANCB	Fanconi anemia, complementation group B [Source:HGNC Symbol;Acc:3583]
97	1554341_a_at	1	9	19	0	1	9	HELQ	helicase, POLQ-like [Source:HGNC Symbol;Acc:18536]
98	202911_at	0	9	20	0	1	9	MSH6	mutS homolog 6 (E. coli) [Source:HGNC Symbol;Acc:7329]
99	201746_at	1	9	19	0	1	9	TP53	tumor protein p53 [Source:HGNC Symbol;Acc:11998]
100	228734_at	2	9	18	1	1	8	UBE2V2	ubiquitin-conjugating enzyme E2 variant 2 [Source:HGNC Symbol;Acc:12495]
101	203564_at	0	9	20	2	0	8	FANCG	Fanconi anemia, complementation group G [Source:HGNC Symbol;Acc:3588]
102	216026_s_at	0	9	20	0	0	10	POLE	polymerase (DNA directed), epsilon, catalytic subunit [Source:HGNC Symbol;Acc:9177]
103	37793_r_at	2	9	18	0	0	10	RAD51D	RAD51 homolog D (S. cerevisiae) [Source:HGNC Symbol;Acc:9823]
104	205672_at	0	9	20	0	0	10	XPA	xeroderma pigmentosum, complementation group A [Source:HGNC Symbol;Acc:12814]
105	204093_at	0	8	21	0	5	5	CCNH	cyclin H [Source:HGNC Symbol;Acc:1594]
106	211450_s_at	0	8	21	1	5	4	MSH6	mutS homolog 6 (E. coli) [Source:HGNC Symbol;Acc:7329]
107	212917_x_at	1	8	20	0	3	7	RECQL	RecQ protein-like (DNA helicase Q1-like) [Source:HGNC Symbol;Acc:9948]
108	212918_at	1	8	20	0	3	7	RECQL	RecQ protein-like (DNA helicase Q1-like) [Source:HGNC Symbol;Acc:9948]
109	203743_s_at	1	8	20	0	3	7	TDG	thymine-DNA glycosylase [Source:HGNC Symbol;Acc:11700]
110	235253_at	0	8	21	0	2	8	RAD1	RAD1 homolog (S. pombe) [Source:HGNC Symbol;Acc:9806]
111	201528_at	0	8	21	0	2	8	RPA1	replication protein A1, 70kDa [Source:HGNC Symbol;Acc:10289]
112	1552937_s_at	0	8	21	0	1	9	ATRIP	ATR interacting protein [Source:HGNC Symbol;Acc:33499]
113	239289_x_at	0	8	21	0	1	9	FAN1	FANCD2/FANCI-associated nuclease 1 [Source:HGNC Symbol;Acc:29170]
114	1553244_at	3	8	18	0	1	9	FANCB	Fanconi anemia, complementation group B [Source:HGNC Symbol;Acc:3583]
115	203422_at	2	8	19	0	1	9	POLD1	polymerase (DNA directed), delta 1, catalytic subunit [Source:HGNC Symbol;Acc:9175]
116	1552937_s_at	0	8	21	0	1	9	TREX1	three prime repair exonuclease 1 [Source:HGNC Symbol;Acc:12269]
117	221703_at	2	8	19	0	0	10	BRIP1	BRCA1 interacting protein C-terminal helicase 1 [Source:HGNC Symbol;Acc:20473]
118	223545_at	0	8	21	0	0	10	FANCD2	Fanconi anemia, complementation group D2 [Source:HGNC Symbol;Acc:3585]
119	203577_at	2	8	19	0	0	10	GTF2H4	general transcription factor IIH, polypeptide 4, 52kDa [Source:HGNC Symbol;Acc:4658]
120	213520_at	0	8	21	0	0	10	RECQL4	RecQ protein-like 4 [Source:HGNC Symbol;Acc:9949]
121	210813_s_at	2	8	19	1	0	9	XRCC4	X-ray repair complementing defective repair in Chinese hamster cells 4 [Source:HGNC Symbol;Acc:12831]
122	202905_x_at	0	7	22	0	5	5	NBN	nibrin [Source:HGNC Symbol;Acc:7652]
123	207405_s_at	1	7	21	0	4	6	RAD17	RAD17 homolog (S. pombe) [Source:HGNC Symbol;Acc:9807]
124	219510_at	1	7	21	0	3	7	POLQ	polymerase (DNA directed), theta [Source:HGNC Symbol;Acc:9186]
125	211300_s_at	3	7	19	1	2	7	TP53	tumor protein p53 [Source:HGNC Symbol;Acc:11998]
126	226127_at	0	7	22	0	1	9	ALKBH3	alkB, alkylation repair homolog 3 (E. coli) [Source:HGNC Symbol;Acc:30141]
127	242711_x_at	1	7	21	0	1	9	FANCM	Fanconi anemia, complementation group M [Source:HGNC Symbol;Acc:23168]
128	205760_s_at	1	7	21	2	1	7	OGG1	8-oxoguanine DNA glycosylase [Source:HGNC Symbol;Acc:8125]
129	204558_at	0	7	22	0	1	9	RAD54L	RAD54-like (S. cerevisiae) [Source:HGNC Symbol;Acc:9826]
130	203160_s_at	0	7	22	0	1	9	RNF8	ring finger protein 8, E3 ubiquitin protein ligase [Source:HGNC Symbol;Acc:10071]

131	236675_at	1	7	21	1	1	8	RPA1	replication protein A1, 70kDa [Source:HGNC Symbol;Acc:10289]
132	210027_s_at	0	7	22	0	0	10	APEX1	APEX nuclease (multifunctional DNA repair enzyme) 1 [Source:HGNC Symbol;Acc:587]
133	205260_s_at	4	7	18	0	0	10	MLH3	mutL homolog 3 (E. coli) [Source:HGNC Symbol;Acc:7128]
134	218903_s_at	1	7	21	0	0	10	NABP2	nucleic acid binding protein 2 [Source:HGNC Symbol;Acc:28412]
135	209731_at	1	7	21	0	0	10	NTHL1	nth endonuclease III-like 1 (E. coli) [Source:HGNC Symbol;Acc:8028]
136	204766_s_at	0	7	22	1	0	9	NUDT1	nudix (nucleoside diphosphate linked moiety X)-type motif 1 [Source:HGNC Symbol;Acc:8048]
137	205023_at	2	7	20	0	0	10	RAD51	RAD51 homolog (S. cerevisiae) [Source:HGNC Symbol;Acc:9817]
138	222629_at	0	7	22	0	0	10	REV1	REV1, polymerase (DNA directed) [Source:HGNC Symbol;Acc:14060]
139	205071_x_at	3	7	19	0	0	10	XRCC4	X-ray repair complementing defective repair in Chinese hamster cells 4 [Source:HGNC Symbol;Acc:12831]
140	223342_at	1	6	22	0	6	4	RRM2B	ribonucleotide reductase M2 B (TP53 inducible) [Source:HGNC Symbol;Acc:17296]
141	209580_s_at	0	6	23	1	5	4	MBD4	methyl-CpG binding domain protein 4 [Source:HGNC Symbol;Acc:6919]
142	214047_s_at	0	6	23	0	4	6	MBD4	methyl-CpG binding domain protein 4 [Source:HGNC Symbol;Acc:6919]
143	205091_x_at	2	6	21	0	4	6	RECQL	RecQ protein-like (DNA helicase Q1-like) [Source:HGNC Symbol;Acc:9948]
144	217618_x_at	3	6	20	0	2	8	HUS1	HUS1 checkpoint homolog (S. pombe) [Source:HGNC Symbol;Acc:5309]
145	226044_at	1	6	22	1	2	7	TDP1	tyrosyl-DNA phosphodiesterase 1 [Source:HGNC Symbol;Acc:18884]
146	203616_at	2	6	21	0	1	9	POLB	polymerase (DNA directed), beta [Source:HGNC Symbol;Acc:9174]
147	210216_x_at	0	6	23	1	1	8	RAD1	RAD1 homolog (S. pombe) [Source:HGNC Symbol;Acc:9806]
148	228535_at	1	6	22	0	1	9	RAD1	RAD1 homolog (S. pombe) [Source:HGNC Symbol;Acc:9806]
149	238656_at	0	6	23	0	1	9	RAD50	RAD50 homolog (S. cerevisiae) [Source:HGNC Symbol;Acc:9816]
150	210255_at	0	6	23	0	1	9	RAD51B	RAD51 homolog B (S. cerevisiae) [Source:HGNC Symbol;Acc:9822]
151	216880_at	3	6	20	0	1	9	RAD51B	RAD51 homolog B (S. cerevisiae) [Source:HGNC Symbol;Acc:9822]
152	211468_s_at	1	6	22	1	1	8	RECQL5	RecQ protein-like 5 [Source:HGNC Symbol;Acc:9950]
153	204408_at	0	6	23	0	0	10	APEX2	APEX nuclease (apurinic/apyrimidinic endonuclease) 2 [Source:HGNC Symbol;Acc:17889]
154	202176_at	0	6	23	0	0	10	ERCC3	excision repair cross-complementing rodent repair deficiency, complementation group 3 [Source:HGNC Symbol;Acc:3435]
155	205647_at	3	6	20	0	0	10	RAD52	RAD52 homolog (S. cerevisiae) [Source:HGNC Symbol;Acc:9824]
156	201899_s_at	0	6	23	0	0	10	UBE2A	ubiquitin-conjugating enzyme E2A [Source:HGNC Symbol;Acc:12472]
157	216299_s_at	1	6	22	0	0	10	XRCC3	X-ray repair complementing defective repair in Chinese hamster cells 3 [Source:HGNC Symbol;Acc:12830]
158	223261_at	1	5	23	0	4	6	POLK	polymerase (DNA directed) kappa [Source:HGNC Symbol;Acc:9183]
159	219678_x_at	0	5	24	1	3	6	DCLRE1C	DNA cross-link repair 1C [Source:HGNC Symbol;Acc:17642]
160	202906_s_at	1	5	23	0	3	7	NBN	nibrin [Source:HGNC Symbol;Acc:7652]
161	211228_s_at	0	5	24	1	3	6	RAD17	RAD17 homolog (S. pombe) [Source:HGNC Symbol;Acc:9807]
162	210568_s_at	1	5	23	0	3	7	RECQL	RecQ protein-like (DNA helicase Q1-like) [Source:HGNC Symbol;Acc:9948]
163	202266_at	0	5	24	1	3	6	TDP2	tyrosyl-DNA phosphodiesterase 2 [Source:HGNC Symbol;Acc:17768]
164	201529_s_at	0	5	24	1	2	7	RPA1	replication protein A1, 70kDa [Source:HGNC Symbol;Acc:10289]
165	203678_at	0	5	24	0	1	9	FAN1	FANCD2/FANCI-associated nuclease 1 [Source:HGNC Symbol;Acc:29170]
166	220255_at	0	5	24	1	1	8	FANCE	Fanconi anemia, complementation group E [Source:HGNC Symbol;Acc:3586]
167	204884_s_at	1	5	23	1	1	8	HUS1	HUS1 checkpoint homolog (S. pombe) [Source:HGNC Symbol;Acc:5309]
168	204461_x_at	0	5	24	0	1	9	RAD1	RAD1 homolog (S. pombe) [Source:HGNC Symbol;Acc:9806]
169	233893_s_at	4	5	20	0	1	9	UVSSA	UV-stimulated scaffold protein A [Source:HGNC Symbol;Acc:29304]
170	200792_at	0	5	24	0	1	9	XRCC6	X-ray repair complementing defective repair in Chinese hamster cells 6 [Source:HGNC Symbol;Acc:4055]
171	219490_s_at	1	5	23	1	0	9	DCLRE1B	DNA cross-link repair 1B [Source:HGNC Symbol;Acc:17641]
172	242927_at	1	5	23	1	0	9	DCLRE1C	DNA cross-link repair 1C [Source:HGNC Symbol;Acc:17642]
173	203805_s_at	0	5	24	0	0	10	FANCA	Fanconi anemia, complementation group A [Source:HGNC Symbol;Acc:3582]
174	205189_s_at	0	5	24	0	0	10	FANCC	Fanconi anemia, complementation group C [Source:HGNC Symbol;Acc:3584]
175	202726_at	3	5	21	1	0	9	LIG1	ligase I, DNA, ATP-dependent [Source:HGNC Symbol;Acc:6598]
176	218463_s_at	0	5	24	0	0	10	MUS81	MUS81 endonuclease homolog (S. cerevisiae) [Source:HGNC Symbol;Acc:29814]
177	1554743_x_at	2	5	22	0	0	10	PMS1	PMS1 postmeiotic segregation increased 1 (S. cerevisiae) [Source:HGNC Symbol;Acc:9121]
178	238670_at	2	5	22	0	0	10	RAD18	RAD18 homolog (S. cerevisiae) [Source:HGNC Symbol;Acc:18278]
179	1553015_a_at	0	5	24	0	0	10	RECQL4	RecQ protein-like 4 [Source:HGNC Symbol;Acc:9949]
180	230411_at	0	5	24	0	0	10	UBE2V2	ubiquitin-conjugating enzyme E2 variant 2 [Source:HGNC Symbol;Acc:12495]
181	217299_s_at	1	4	24	1	6	3	NBN	nibrin [Source:HGNC Symbol;Acc:7652]
182	211763_s_at	1	4	24	0	5	5	UBE2B	ubiquitin-conjugating enzyme E2B [Source:HGNC Symbol;Acc:12473]
183	211297_s_at	5	4	20	3	3	4	CDK7	cyclin-dependent kinase 7 [Source:HGNC Symbol;Acc:1778]
184	226832_at	3	4	22	0	3	7	RNF168	ring finger protein 168, E3 ubiquitin protein ligase [Source:HGNC Symbol;Acc:26661]
185	207598_x_at	2	4	23	1	3	6	XRCC2	X-ray repair complementing defective repair in Chinese hamster cells 2 [Source:HGNC Symbol;Acc:12829]
186	238992_at	5	4	20	0	2	8	POLI	polymerase (DNA directed) iota [Source:HGNC Symbol;Acc:9182]
187	204460_s_at	0	4	25	0	2	8	RAD1	RAD1 homolog (S. pombe) [Source:HGNC Symbol;Acc:9806]
188	234464_s_at	0	4	25	0	1	9	EME1	essential meiotic endonuclease 1 homolog 1 (S. pombe) [Source:HGNC Symbol;Acc:24965]
189	1569867_at	2	4	23	0	1	9	EME2	essential meiotic endonuclease 1 homolog 2 (S. pombe) [Source:HGNC Symbol;Acc:27289]
190	215470_at	10	4	15	0	1	9	GTF2H2C	general transcription factor IIH, polypeptide 2C [Source:HGNC Symbol;Acc:31394]
191	219715_s_at	0	4	25	0	1	9	TDP1	tyrosyl-DNA phosphodiesterase 1 [Source:HGNC Symbol;Acc:18884]
192	241379_at	1	4	24	0	0	10	APLF	aprataxin and PNKP like factor [Source:HGNC Symbol;Acc:28724]
193	236584_at	1	4	24	0	0	10	C1orf86	chromosome 1 open reading frame 86 [Source:HGNC Symbol;Acc:26428]
194	234465_at	1	4	24	0	0	10	EME1	essential meiotic endonuclease 1 homolog 1 (S. pombe) [Source:HGNC Symbol;Acc:24965]
195	212525_s_at	0	4	25	1	0	9	H2AFX	H2A histone family, member X [Source:HGNC Symbol;Acc:4739]
196	204838_s_at	3	4	22	0	0	10	MLH3	mutL homolog 3 (E. coli) [Source:HGNC Symbol;Acc:7128]

197	210630_s_at	2	4	23	0	0	10	RAD52	RAD52 homolog (S. cerevisiae) [Source:HGNC Symbol;Acc:9824]
198	220549_at	6	4	19	0	0	10	RAD54B	RAD54 homolog B (S. cerevisiae) [Source:HGNC Symbol;Acc:17228]
199	204828_at	3	4	22	0	0	10	RAD9A	RAD9 homolog A (S. pombe) [Source:HGNC Symbol;Acc:9827]
200	233780_at	4	4	21	1	0	9	RIF1	RAP1 interacting factor homolog (yeast) [Source:HGNC Symbol;Acc:23207]
201	1554060_s_at	2	4	23	0	0	10	SETMAR	SET domain and mariner transposase fusion gene [Source:HGNC Symbol;Acc:10762]
202	218685_s_at	0	4	25	0	0	10	SMUG1	single-strand-selective monofunctional uracil-DNA glycosylase 1 [Source:HGNC Symbol;Acc:17148]
203	209375_at	1	4	24	0	0	10	XPC	xeroderma pigmentosum, complementation group C [Source:HGNC Symbol;Acc:12816]
204	222104_x_at	1	3	25	1	4	5	GTF2H3	general transcription factor IIH, polypeptide 3, 34kDa [Source:HGNC Symbol;Acc:4657]
205	208393_s_at	1	3	25	0	4	6	RAD50	RAD50 homolog (S. cerevisiae) [Source:HGNC Symbol;Acc:9816]
206	202983_at	1	3	25	0	3	7	HLTF	helicase-like transcription factor [Source:HGNC Symbol;Acc:11099]
207	223260_s_at	2	3	24	0	3	7	POLK	polymerase (DNA directed) kappa [Source:HGNC Symbol;Acc:9183]
208	210826_x_at	0	3	26	1	3	6	RAD17	RAD17 homolog (S. pombe) [Source:HGNC Symbol;Acc:9807]
209	208642_s_at	0	3	26	0	3	7	XRCC5	X-ray repair complementing defective repair in Chinese hamster cells 5 (double-strand-break rejoining) [Source:HGNC Symbol;Acc:12833]
210	1569868_s_at	1	3	25	1	1	8	EME2	essential meiotic endonuclease 1 homolog 2 (S. pombe) [Source:HGNC Symbol;Acc:27289]
211	213468_at	2	3	24	0	1	9	ERCC2	excision repair cross-complementing rodent repair deficiency, complementation group 2 [Source:HGNC Symbol;Acc:3434]
212	236976_at	2	3	24	0	1	9	FANCA	Fanconi anemia, complementation group A [Source:HGNC Symbol;Acc:3582]
213	207348_s_at	3	3	23	0	1	9	LIG3	ligase III, DNA, ATP-dependent [Source:HGNC Symbol;Acc:6600]
214	212913_at	4	3	22	0	1	9	MSH5	mutS homolog 5 (E. coli) [Source:HGNC Symbol;Acc:7328]
215	214086_s_at	3	3	23	0	1	9	PARP2	poly (ADP-ribose) polymerase 2 [Source:HGNC Symbol;Acc:272]
216	209805_at	6	3	20	1	1	8	PMS2	PMS2 postmeiotic segregation increased 2 (S. cerevisiae) [Source:HGNC Symbol;Acc:9122]
217	1557700_at	3	3	23	0	1	9	POLH	polymerase (DNA directed), eta [Source:HGNC Symbol;Acc:9181]
218	210416_s_at	0	3	26	0	0	10	CHEK2	checkpoint kinase 2 [Source:HGNC Symbol;Acc:16627]
219	203409_at	10	3	16	1	0	9	DDB2	damage-specific DNA binding protein 2, 48kDa [Source:HGNC Symbol;Acc:2718]
220	220716_at	0	3	26	0	0	10	ERCC8	excision repair cross-complementing rodent repair deficiency, complementation group 8 [Source:HGNC Symbol;Acc:3439]
221	203061_s_at	1	3	25	0	0	10	MDC1	mediator of DNA-damage checkpoint 1 [Source:HGNC Symbol;Acc:21163]
222	203062_s_at	0	3	26	0	0	10	MDC1	mediator of DNA-damage checkpoint 1 [Source:HGNC Symbol;Acc:21163]
223	204880_at	1	3	25	3	0	7	MGMT	O-6-methylguanine-DNA methyltransferase [Source:HGNC Symbol;Acc:7059]
224	214525_x_at	0	3	26	0	0	10	MLH3	mutL homolog 3 (E. coli) [Source:HGNC Symbol;Acc:7128]
225	1554742_at	2	3	24	0	0	10	PMS1	PMS1 postmeiotic segregation increased 1 (S. cerevisiae) [Source:HGNC Symbol;Acc:9121]
226	241820_at	2	3	24	0	0	10	RIF1	RAP1 interacting factor homolog (yeast) [Source:HGNC Symbol;Acc:23207]
227	203161_s_at	2	3	24	1	0	9	RNF8	ring finger protein 8, E3 ubiquitin protein ligase [Source:HGNC Symbol;Acc:10071]
228	232147_at	2	3	24	0	0	10	SLX4	SLX4 structure-specific endonuclease subunit homolog (S. cerevisiae) [Source:HGNC Symbol;Acc:23845]
229	212672_at	2	2	25	0	3	7	ATM	ataxia telangiectasia mutated [Source:HGNC Symbol;Acc:795]
230	204123_at	1	2	26	0	3	7	LIG3	ligase III, DNA, ATP-dependent [Source:HGNC Symbol;Acc:6600]
231	223598_at	2	2	25	0	3	7	RAD23B	RAD23 homolog B (S. cerevisiae) [Source:HGNC Symbol;Acc:9813]
232	218428_s_at	0	2	27	0	3	7	REV1	REV1, polymerase (DNA directed) [Source:HGNC Symbol;Acc:14060]
233	210410_s_at	4	2	23	0	2	8	MSH5	mutS homolog 5 (E. coli) [Source:HGNC Symbol;Acc:7328]
234	226585_at	1	2	26	0	2	8	NEIL2	nei endonuclease VIII-like 2 (E. coli) [Source:HGNC Symbol;Acc:18956]
235	238748_at	0	2	27	0	2	8	RAD18	RAD18 homolog (S. cerevisiae) [Source:HGNC Symbol;Acc:18278]
236	202334_s_at	1	2	26	1	2	7	UBE2B	ubiquitin-conjugating enzyme E2B [Source:HGNC Symbol;Acc:12473]
237	203565_s_at	0	2	27	1	1	8	MNAT1	menage a trois homolog 1, cyclin H assembly factor (Xenopus laevis) [Source:HGNC Symbol;Acc:7181]
238	215773_x_at	5	2	22	0	1	9	PARP2	poly (ADP-ribose) polymerase 2 [Source:HGNC Symbol;Acc:272]
239	219380_x_at	4	2	23	2	1	7	POLH	polymerase (DNA directed), eta [Source:HGNC Symbol;Acc:9181]
240	222879_s_at	6	2	21	0	1	9	POLH	polymerase (DNA directed), eta [Source:HGNC Symbol;Acc:9181]
241	214872_at	2	2	25	0	1	9	RIF1	RAP1 interacting factor homolog (yeast) [Source:HGNC Symbol;Acc:23207]
242	1553127_a_at	4	2	23	1	1	8	RNF168	ring finger protein 168, E3 ubiquitin protein ligase [Source:HGNC Symbol;Acc:26661]
243	238813_at	1	2	26	3	0	7	APEX2	APEX nuclease (apurinic/apyrimidinic endonuclease) 2 [Source:HGNC Symbol;Acc:17889]
244	1555100_at	2	2	25	0	0	10	APLF	aprataxin and PNKP like factor [Source:HGNC Symbol;Acc:28724]
245	222658_s_at	0	2	27	0	0	10	APTX	aprataxin [Source:HGNC Symbol;Acc:15984]
246	230108_at	0	2	27	1	0	9	ERCC6	excision repair cross-complementing rodent repair deficiency, complementation group 6 [Source:HGNC Symbol;Acc:3438]
247	1554882_at	1	2	26	0	0	10	ERCC8	excision repair cross-complementing rodent repair deficiency, complementation group 8 [Source:HGNC Symbol;Acc:3439]
248	1554342_s_at	0	2	27	0	0	10	HELQ	helicase, POLQ-like [Source:HGNC Symbol;Acc:18536]
249	217216_x_at	1	2	26	0	0	10	MLH3	mutL homolog 3 (E. coli) [Source:HGNC Symbol;Acc:7128]
250	211334_at	2	2	25	1	0	9	MRE11A	MRE11 meiotic recombination 11 homolog A (S. cerevisiae) [Source:HGNC Symbol;Acc:7230]
251	1557701_s_at	6	2	21	1	0	9	POLH	polymerase (DNA directed), eta [Source:HGNC Symbol;Acc:9181]
252	240098_at	2	2	25	0	0	10	RIF1	RAP1 interacting factor homolog (yeast) [Source:HGNC Symbol;Acc:23207]
253	218317_x_at	1	2	26	0	0	10	SLX1A	SLX1 structure-specific endonuclease subunit homolog A (S. cerevisiae) [Source:HGNC Symbol;Acc:20922]
254	218317_x_at	1	2	26	0	0	10	SLX1B	SLX1 structure-specific endonuclease subunit homolog B (S. cerevisiae) [Source:HGNC Symbol;Acc:28748]
255	223684_s_at	0	2	27	0	0	10	SMUG1	single-strand-selective monofunctional uracil-DNA glycosylase 1 [Source:HGNC Symbol;Acc:17148]
256	236177_s_at	0	2	27	0	0	10	TP53BP2	tumor protein p53 binding protein, 2 [Source:HGNC Symbol;Acc:12000]
257	211788_s_at	1	2	26	2	0	8	TREX2	three prime repair exonuclease 2 [Source:HGNC Symbol;Acc:12270]
258	209579_s_at	0	1	28	0	2	8	MBD4	methyl-CpG binding domain protein 4 [Source:HGNC Symbol;Acc:6919]
259	203120_at	2	1	26	1	2	7	TP53BP2	tumor protein p53 binding protein, 2 [Source:HGNC Symbol;Acc:12000]
260	208442_s_at	5	1	23	0	1	9	ATM	ataxia telangiectasia mutated [Source:HGNC Symbol;Acc:795]
261	210858_x_at	3	1	25	0	1	9	ATM	ataxia telangiectasia mutated [Source:HGNC Symbol;Acc:795]
262	205875_s_at	4	1	24	0	1	9	ATRIP	ATR interacting protein [Source:HGNC Symbol;Acc:33499]

263	1556309_s_at	0	1	28	1	1	8	C1orf86	chromosome 1 open reading frame 86 [Source:HGNC Symbol;Acc:26428]
264	236499_at	1	1	27	1	1	8	C1orf86	chromosome 1 open reading frame 86 [Source:HGNC Symbol;Acc:26428]
265	208382_s_at	2	1	26	1	1	8	DMC1	DMC1 dosage suppressor of mck1 homolog, meiosis-specific homologous recombination (yeast) [Source:HGNC Symbol;Acc:2927]
266	210158_at	2	1	26	0	1	9	ERCC4	excision repair cross-complementing rodent repair deficiency, complementation group 4 [Source:HGNC Symbol;Acc:3436]
267	213357_at	1	1	27	0	1	9	GTF2H5	general transcription factor IIH, polypeptide 5 [Source:HGNC Symbol;Acc:21157]
268	223234_at	0	1	28	0	1	9	MAD2L2	MAD2 mitotic arrest deficient-like 2 (yeast) [Source:HGNC Symbol;Acc:6764]
269	214048_at	7	1	21	0	1	9	MBD4	methyl-CpG binding domain protein 4 [Source:HGNC Symbol;Acc:6919]
270	201222_s_at	2	1	26	1	1	8	RAD23B	RAD23 homolog B (S. cerevisiae) [Source:HGNC Symbol;Acc:9813]
271	1560171_at	6	1	22	0	1	9	SHPRH	SNF2 histone linker PHD RING helicase, E3 ubiquitin protein ligase [Source:HGNC Symbol;Acc:19336]
272	205875_s_at	4	1	24	0	1	9	TREX1	three prime repair exonuclease 1 [Source:HGNC Symbol;Acc:12269]
273	1553387_at	4	1	24	0	0	10	ATM	ataxia telangiectasia mutated [Source:HGNC Symbol;Acc:795]
274	214816_x_at	1	1	27	1	0	9	C19orf40	chromosome 19 open reading frame 40 [Source:HGNC Symbol;Acc:28467]
275	208386_x_at	1	1	27	0	0	10	DMC1	DMC1 dosage suppressor of mck1 homolog, meiosis-specific homologous recombination (yeast) [Source:HGNC Symbol;Acc:2927]
276	229939_at	3	1	25	0	0	10	ENDOV	endonuclease V [Source:HGNC Symbol;Acc:26640]
277	238401_at	5	1	23	1	0	9	ENDOV	endonuclease V [Source:HGNC Symbol;Acc:26640]
278	238402_s_at	1	1	27	1	0	9	ENDOV	endonuclease V [Source:HGNC Symbol;Acc:26640]
279	228131_at	3	1	25	5	0	5	ERCC1	excision repair cross-complementing rodent repair deficiency, complementation group 1 (includes overlapping antisense sequence) [Source:HGNC Symbol;Acc:3433]
280	207347_at	6	1	22	0	0	10	ERCC6	excision repair cross-complementing rodent repair deficiency, complementation group 6 [Source:HGNC Symbol;Acc:3438]
281	215530_at	1	1	27	0	0	10	FANCA	Fanconi anemia, complementation group A [Source:HGNC Symbol;Acc:3582]
282	231971_at	0	1	28	0	0	10	FANCM	Fanconi anemia, complementation group M [Source:HGNC Symbol;Acc:23168]
283	241477_at	2	1	26	0	0	10	NEIL1	nei endonuclease VIII-like 1 (E. coli) [Source:HGNC Symbol;Acc:18448]
284	1558556_at	1	1	27	0	0	10	OGG1	8-oxoguanine DNA glycosylase [Source:HGNC Symbol;Acc:8125]
285	215272_at	5	1	23	0	0	10	OGG1	8-oxoguanine DNA glycosylase [Source:HGNC Symbol;Acc:8125]
286	218961_s_at	0	1	28	0	0	10	PNKP	polynucleotide kinase 3'-phosphatase [Source:HGNC Symbol;Acc:9154]
287	217636_at	3	1	25	0	0	10	POLG	polymerase (DNA directed), gamma [Source:HGNC Symbol;Acc:9179]
288	233852_at	2	1	26	1	0	9	POLH	polymerase (DNA directed), eta [Source:HGNC Symbol;Acc:9181]
289	222238_s_at	8	1	20	1	0	9	POLM	polymerase (DNA directed), mu [Source:HGNC Symbol;Acc:9185]
290	228964_at	10	1	18	5	0	5	PRDM1	PR domain containing 1, with ZNF domain [Source:HGNC Symbol;Acc:9346]
291	201039_s_at	2	1	26	0	0	10	RAD23A	RAD23 homolog A (S. cerevisiae) [Source:HGNC Symbol;Acc:9812]
292	212889_x_at	0	1	28	0	0	10	RAD23A	RAD23 homolog A (S. cerevisiae) [Source:HGNC Symbol;Acc:9812]
293	225495_x_at	2	1	26	0	0	10	RAD23A	RAD23 homolog A (S. cerevisiae) [Source:HGNC Symbol;Acc:9812]
294	1554496_at	1	1	27	0	0	10	RAD51B	RAD51 homolog B (S. cerevisiae) [Source:HGNC Symbol;Acc:9822]
295	211904_x_at	1	1	27	1	0	9	RAD52	RAD52 homolog (S. cerevisiae) [Source:HGNC Symbol;Acc:9824]
296	1562022_s_at	0	1	28	1	0	9	RAD9A	RAD9 homolog A (S. pombe) [Source:HGNC Symbol;Acc:9827]
297	233781_s_at	4	1	24	0	0	10	RIF1	RAP1 interacting factor homolog (yeast) [Source:HGNC Symbol;Acc:23207]
298	212696_s_at	0	1	28	0	0	10	RNF4	ring finger protein 4 [Source:HGNC Symbol;Acc:10067]
299	221143_at	3	1	25	0	0	10	RPA4	replication protein A4, 30kDa [Source:HGNC Symbol;Acc:30305]
300	1554059_at	0	1	28	1	0	9	SETMAR	SET domain and mariner transposase fusion gene [Source:HGNC Symbol;Acc:10762]
301	201898_s_at	1	1	27	0	0	10	UBE2A	ubiquitin-conjugating enzyme E2A [Source:HGNC Symbol;Acc:12472]
302	202333_s_at	2	1	26	0	0	10	UBE2B	ubiquitin-conjugating enzyme E2B [Source:HGNC Symbol;Acc:12473]
303	202335_s_at	1	1	27	0	0	10	UBE2B	ubiquitin-conjugating enzyme E2B [Source:HGNC Symbol;Acc:12473]
304	1555107_a_at	3	1	25	0	0	10	UVSSA	UV-stimulated scaffold protein A [Source:HGNC Symbol;Acc:29304]
305	210812_at	2	1	26	1	0	9	XRCC4	X-ray repair complementing defective repair in Chinese hamster cells 4 [Source:HGNC Symbol;Acc:12831]
306	209096_at	0	0	29	0	3	7	UBE2V2	ubiquitin-conjugating enzyme E2 variant 2 [Source:HGNC Symbol;Acc:12495]
307	210947_s_at	1	0	28	0	2	8	MSH3	mutS homolog 3 (E. coli) [Source:HGNC Symbol;Acc:7326]
308	219317_at	12	0	17	1	2	7	POLI	polymerase (DNA directed) iota [Source:HGNC Symbol;Acc:9182]
309	201223_s_at	2	0	27	0	2	8	RAD23B	RAD23 homolog B (S. cerevisiae) [Source:HGNC Symbol;Acc:9813]
310	233288_at	2	0	27	0	1	9	ATR	ataxia telangiectasia and Rad3 related [Source:HGNC Symbol;Acc:882]
311	208619_at	1	0	28	0	1	9	DDB1	damage-specific DNA binding protein 1, 127kDa [Source:HGNC Symbol;Acc:2717]
312	203719_at	2	0	27	0	1	9	ERCC1	excision repair cross-complementing rodent repair deficiency, complementation group 1 (includes overlapping antisense sequence) [Source:HGNC Symbol;Acc:3433]
313	202414_at	8	0	21	2	1	7	ERCC5	excision repair cross-complementing rodent repair deficiency, complementation group 5 [Source:HGNC Symbol;Acc:3437]
314	242656_at	5	0	24	0	1	9	GTF2H1	general transcription factor IIH, polypeptide 1, 62kDa [Source:HGNC Symbol;Acc:4655]
315	205887_x_at	3	0	26	1	1	8	MSH3	mutS homolog 3 (E. coli) [Source:HGNC Symbol;Acc:7326]
316	217635_s_at	7	0	22	1	1	8	POLG	polymerase (DNA directed), gamma [Source:HGNC Symbol;Acc:9179]
317	201046_s_at	1	0	28	0	1	9	RAD23A	RAD23 homolog A (S. cerevisiae) [Source:HGNC Symbol;Acc:9812]
318	241526_at	0	0	29	2	0	8	APLF	aprataxin and PNKP like factor [Source:HGNC Symbol;Acc:28724]
319	222338_x_at	0	0	29	0	0	10	APTX	aprataxin [Source:HGNC Symbol;Acc:15984]
320	243132_at	2	0	27	0	0	10	APTX	aprataxin [Source:HGNC Symbol;Acc:15984]
321	1554631_at	1	0	28	0	0	10	ATM	ataxia telangiectasia mutated [Source:HGNC Symbol;Acc:795]
322	1570352_at	3	0	26	0	0	10	ATM	ataxia telangiectasia mutated [Source:HGNC Symbol;Acc:795]
323	34689_at	4	0	25	0	0	10	ATRIP	ATR interacting protein [Source:HGNC Symbol;Acc:33499]
324	1558544_at	0	0	29	0	0	10	C1orf86	chromosome 1 open reading frame 86 [Source:HGNC Symbol;Acc:26428]
325	229113_s_at	1	0	28	0	0	10	C1orf86	chromosome 1 open reading frame 86 [Source:HGNC Symbol;Acc:26428]
326	236498_s_at	1	0	28	1	0	9	C1orf86	chromosome 1 open reading frame 86 [Source:HGNC Symbol;Acc:26428]
327	203229_s_at	6	0	23	0	0	10	CLK2	CDC-like kinase 2 [Source:HGNC Symbol;Acc:2069]
328	236185_at	2	0	27	0	0	10	DCLRE1A	DNA cross-link repair 1A [Source:HGNC Symbol;Acc:17660]

329	1556024_at	1	0	28	0	0	10	EME2	essential meiotic endonuclease 1 homolog 2 (S. pombe) [Source:HGNC Symbol;Acc:27289]
330	203720_s_at	0	0	29	0	0	10	ERCC1	excision repair cross-complementing rodent repair deficiency, complementation group 1 (includes overlapping antisense sequence) [Source:HGNC Symbol;Acc:3433]
331	235399_at	1	0	28	0	0	10	ERCC2	excision repair cross-complementing rodent repair deficiency, complementation group 2 [Source:HGNC Symbol;Acc:3434]
332	1568889_at	2	0	27	0	0	10	FANCD2	Fanconi anemia, complementation group D2 [Source:HGNC Symbol;Acc:3585]
333	1568891_x_at	1	0	28	0	0	10	FANCD2	Fanconi anemia, complementation group D2 [Source:HGNC Symbol;Acc:3585]
334	204883_s_at	6	0	23	0	0	10	HUS1	HUS1 checkpoint homolog (S. pombe) [Source:HGNC Symbol;Acc:5309]
335	1555169_at	3	0	26	0	0	10	LIG3	ligase III, DNA, ATP-dependent [Source:HGNC Symbol;Acc:6600]
336	202167_s_at	10	0	19	0	0	10	MMS19	MMS19 nucleotide excision repair homolog (S. cerevisiae) [Source:HGNC Symbol;Acc:13824]
337	237593_at	3	0	26	1	0	9	MNAT1	menage a trois homolog 1, cyclin H assembly factor (Xenopus laevis) [Source:HGNC Symbol;Acc:7181]
338	203686_at	0	0	29	0	0	10	MPG	N-methylpurine-DNA glycosylase [Source:HGNC Symbol;Acc:7211]
339	229122_x_at	2	0	27	0	0	10	MPG	N-methylpurine-DNA glycosylase [Source:HGNC Symbol;Acc:7211]
340	210533_at	0	0	29	1	0	9	MSH4	mutS homolog 4 (E. coli) [Source:HGNC Symbol;Acc:7327]
341	221406_s_at	4	0	25	0	0	10	MSH5	mutS homolog 5 (E. coli) [Source:HGNC Symbol;Acc:7328]
342	219396_s_at	3	0	26	1	0	9	NEIL1	nei endonuclease VIII-like 1 (E. coli) [Source:HGNC Symbol;Acc:18448]
343	219418_at	0	0	29	0	0	10	NHEJ1	nonhomologous end-joining factor 1 [Source:HGNC Symbol;Acc:25737]
344	204752_x_at	5	0	24	0	0	10	PARP2	poly (ADP-ribose) polymerase 2 [Source:HGNC Symbol;Acc:272]
345	209940_at	3	0	26	5	0	5	PARP3	poly (ADP-ribose) polymerase family, member 3 [Source:HGNC Symbol;Acc:273]
346	202861_at	19	0	10	0	0	10	PER1	period circadian clock 1 [Source:HGNC Symbol;Acc:8845]
347	242832_at	3	0	26	0	0	10	PER1	period circadian clock 1 [Source:HGNC Symbol;Acc:8845]
348	36829_at	13	0	16	0	0	10	PER1	period circadian clock 1 [Source:HGNC Symbol;Acc:8845]
349	234907_x_at	1	0	28	0	0	10	POLB	polymerase (DNA directed), beta [Source:HGNC Symbol;Acc:9174]
350	1561940_at	1	0	28	0	0	10	POLE	polymerase (DNA directed), epsilon, catalytic subunit [Source:HGNC Symbol;Acc:9177]
351	203366_at	4	0	25	1	0	9	POLG	polymerase (DNA directed), gamma [Source:HGNC Symbol;Acc:9179]
352	1555317_at	4	0	25	0	0	10	POLK	polymerase (DNA directed) kappa [Source:HGNC Symbol;Acc:9183]
353	221049_s_at	2	0	27	0	0	10	POLL	polymerase (DNA directed), lambda [Source:HGNC Symbol;Acc:9184]
354	233454_at	0	0	29	0	0	10	POLN	polymerase (DNA directed) nu [Source:HGNC Symbol;Acc:18870]
355	242804_at	3	0	26	1	0	9	POLN	polymerase (DNA directed) nu [Source:HGNC Symbol;Acc:18870]
356	217192_s_at	7	0	22	2	0	8	PRDM1	PR domain containing 1, with ZNF domain [Source:HGNC Symbol;Acc:9346]
357	1561122_a_at	2	0	27	0	0	10	RAD51B	RAD51 homolog B (S. cerevisiae) [Source:HGNC Symbol;Acc:9822]
358	210309_at	0	0	29	0	0	10	RECQL5	RecQ protein-like 5 [Source:HGNC Symbol;Acc:9950]
359	221686_s_at	0	0	29	0	0	10	RECQL5	RecQ protein-like 5 [Source:HGNC Symbol;Acc:9950]
360	34063_at	0	0	29	0	0	10	RECQL5	RecQ protein-like 5 [Source:HGNC Symbol;Acc:9950]
361	222628_s_at	0	0	29	0	0	10	REV1	REV1, polymerase (DNA directed) [Source:HGNC Symbol;Acc:14060]
362	243592_at	3	0	26	0	0	10	REV1	REV1, polymerase (DNA directed) [Source:HGNC Symbol;Acc:14060]
363	202276_at	1	0	28	0	0	10	SHFM1	split hand/foot malformation (ectrodactyly) type 1 [Source:HGNC Symbol;Acc:10845]
364	222094_at	4	0	25	0	0	10	SLX1A	SLX1 structure-specific endonuclease subunit homolog A (S. cerevisiae) [Source:HGNC Symbol;Acc:20922]
365	233334_x_at	0	0	29	0	0	10	SLX1A	SLX1 structure-specific endonuclease subunit homolog A (S. cerevisiae) [Source:HGNC Symbol;Acc:20922]
366	222094_at	4	0	25	0	0	10	SLX1B	SLX1 structure-specific endonuclease subunit homolog B (S. cerevisiae) [Source:HGNC Symbol;Acc:28748]
367	233334_x_at	0	0	29	0	0	10	SLX1B	SLX1 structure-specific endonuclease subunit homolog B (S. cerevisiae) [Source:HGNC Symbol;Acc:28748]
368	239687_at	1	0	28	2	0	8	SLX4	SLX4 structure-specific endonuclease subunit homolog (S. cerevisiae) [Source:HGNC Symbol;Acc:23845]
369	222259_s_at	0	0	29	0	0	10	SPO11	SPO11 meiotic protein covalently bound to DSB homolog (S. cerevisiae) [Source:HGNC Symbol;Acc:11250]
370	34689_at	4	0	25	0	0	10	TREX1	three prime repair exonuclease 1 [Source:HGNC Symbol;Acc:12269]
371	218110_at	5	0	24	0	0	10	XAB2	XPA binding protein 2 [Source:HGNC Symbol;Acc:14089]
372	203655_at	0	0	29	0	0	10	XRCC1	X-ray repair complementing defective repair in Chinese hamster cells 1 [Source:HGNC Symbol;Acc:12828]
