# Supplementary material for: Differentiated embryo chondrocyte plays a crucial role in DNA damage response via transcriptional regulation under hypoxic conditions
Source: PLoS One. 2018 Feb 21;13(2):e0192136. doi: 10.1371/journal.pone.0192136 (PMC5821451; doi:10.1371/journal.pone.0192136)
Supplement: S1 Fig — A scatter plot of normalized gene expressions in HSC-2 under normoxic versus hypoxic conditions is shown in left panel. Known hypoxia inducible genes (listed in right panel) and DNA-DRR genes (listed in S4 Table) are indicated as red and dark blue dots. (PDF) [file pone.0192136.s007.pdf]

S1 Fig. Scatter plot of gene expressions in HSC2. A scatter plot of normalized gene expressions in HSC2 under normoxic versus hypoxic conditions is shown in left panel. Known hypoxia inducible genes (listed in right panel) and DNA-DRR genes (listed in S3 Table) are indicated as red and dark blue dots.

S1 Fig

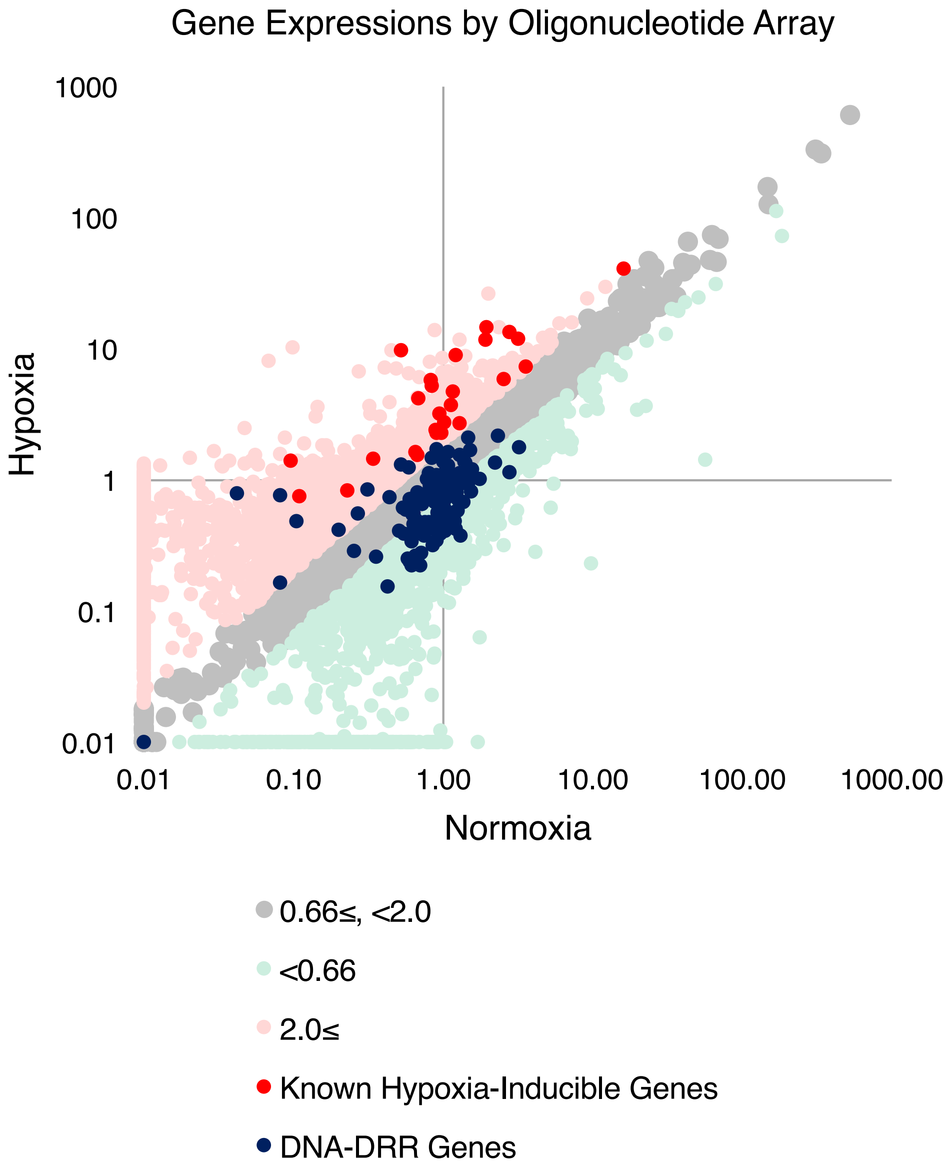

Known Hypoxia-Inducible Genes

Normoxia	Hypoxia	Fold	Gene Symbol	Genbank
0.68	4.18	6.12	ADM	NM_001124
0.84	5.23	6.20	ADM	D43639
0.98	2.30	2.35	DEC1	NM_003670
0.34	1.45	4.23	DEC2	NM_030762
0.83	5.82	7.02	BNIP3	AF002697
1.13	3.75	3.32	BNIP3	NM_004052
0.23	0.84	3.63	CA9	NM_001216
0.66	1.62	2.46	EGLN1	AF334711
0.67	1.55	2.30	EGLN1	NM_022051
0.10	1.41	14.68	EGLN3	NM_022073
0.11	0.75	6.86	EPO	NM_000799
0.89	2.42	2.71	LDHA	NM_005566
1.95	14.71	7.55	LOX	NM_002317
1.21	8.97	7.39	LOX	L16895
2.80	13.40	4.79	LOX	AF039291
1.29	2.70	2.10	MMP2	BC002576
2.54	5.87	2.31	MMP2	NM_004530
0.52	9.76	18.59	NDRG1	NM_006096
0.95	3.19	3.36	PDK1	NM_002610
1.16	4.72	4.05	PGK1	NM_000291
1.93	11.79	6.12	SERPINE1	NM_000602
1.01	2.77	2.73	SLC2A1	NM_006516
3.17	11.93	3.77	SLC2A3	NM_006931
3.61	7.27	2.01	SOX9	NM_000346
16.33	41.03	2.51	TIMP3	NM_000362
0.91	2.29	2.51	VEGF	AF022375
