# Supplementary material for: Differentiated embryo chondrocyte plays a crucial role in DNA damage response via transcriptional regulation under hypoxic conditions
Source: PLoS One. 2018 Feb 21;13(2):e0192136. doi: 10.1371/journal.pone.0192136 (PMC5821451; doi:10.1371/journal.pone.0192136)
Supplement: S2 Fig — HSC-2 cells were incubated under 21% pO2 (N) or 1% pO2 (H) for 24 hours, or treated with HIF-activating reagents,10 μM FG-4592, 50 μM 2,2’-Dipyridyl (DP), 200 μM CoCl2, 400 μM Dimethyloxalylglycine (DMOG), or 50 μM Desferoxamine mesylate (DFOM) for 24 hours. Expression levels of MSH2, MBD4, MRE11A, BRCA1, RAD51, and MLH1 were analyzed by real-time RT-PCR. Relative mRNA levels were calculated as the ratio to that of ACTB. Columns are the mean of three independent experiments; bars, SD. The differences between means were significant (ANOVA P < 0.005). P values calculated with Dunnet test are: *, P < 0.05; **, P < 0.01; ***, P < 0.001. (PDF) [file pone.0192136.s008.pdf]

S2 Fig. Expressions of DNA-DRR genes were decreased by treatments with HIF-activating reagents. HSC-2 cells were incubated under 21% pO_2 (N) or 1% pO_2 (H) for 24 hours, or treated with HIF-activating reagents,10 µM FG-4592, 50 µM 2,2'-Dipyridyl (DP), 200 µM $CoCl_2$, 400 µM Dimethyloxalylglycine (DMOG), or 50 µM Desferoxamine mesylate (DFOM) for 24 hours. Expression levels of *MSH2*, *MBD4*, *MRE11A*, *BRCA1*, *RAD51*, and *MLH1* were analyzed by real-time RT-PCR. Relative mRNA levels were calculated as the ratio to that of *ACTB*. Columns are the mean of three independent experiments; bars, SD. The differences between means were significant (ANOVA $P < 0.005$). P values calculated with Dunnet test are: *, $P < 0.05$; **, $P < 0.01$; ***, $P < 0.001$.

S2 Fig

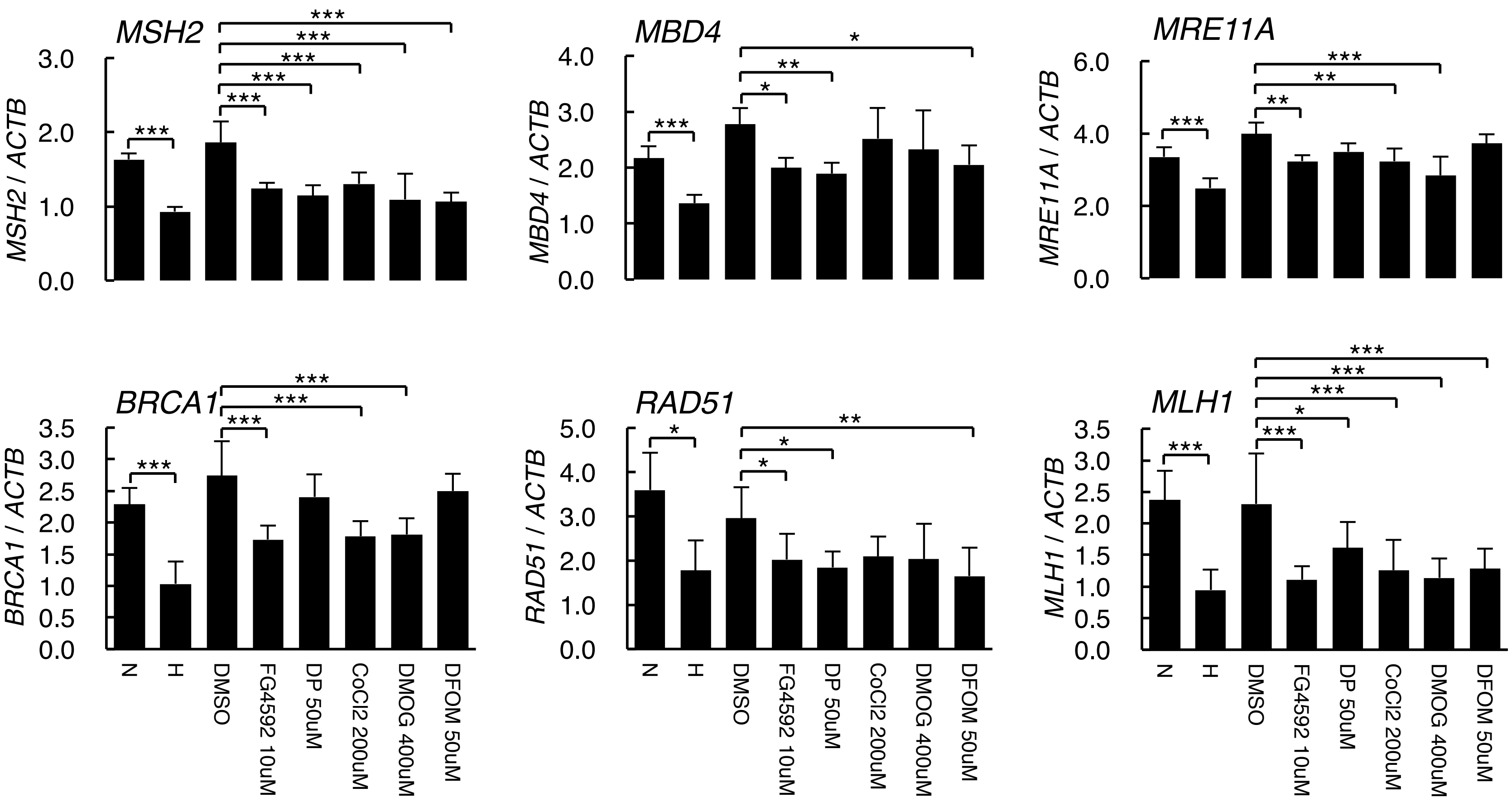

MSH2
MSH2 / ACTB
MBD4
MBD4 / ACTB
MRE11A
MRE11A / ACTB
BRCA1
BRCA1 / ACTB
RAD51
RAD51 / ACTB
MLH1
MLH1 / ACTB
N
H
DMSO
FG4592 10uM
DP 50uM
CoCl2 200uM
DMOG 400uM
DFOM 50uM
