# Supplementary material for: Differentiated embryo chondrocyte plays a crucial role in DNA damage response via transcriptional regulation under hypoxic conditions
Source: PLoS One. 2018 Feb 21;13(2):e0192136. doi: 10.1371/journal.pone.0192136 (PMC5821451; doi:10.1371/journal.pone.0192136)
Supplement: S3 Fig — Non-specific siRNA (siNS) or targeted siRNA was transfected into HSC-2 and cultured under normoxic or hypoxic conditions for 24 hours. Expression levels of HIF1A, DEC1, and DEC2 were evaluated by real-time RT-PCR (left panel). Relative mRNA levels were calculated as the ratio to that of ACTB. Columns are the mean of three independent experiments; bars, SD. The differences between means were significant (ANOVA P < 0.005). P values calculated with Turkey-Kramer HSD test are: *, P < 0.05; **, P < 0.01; ***, P < 0.001. Expression levels of HIF-1α, DEC1, and DEC2 proteins were evaluated using immunostaining analysis (right panel). (PDF) [file pone.0192136.s009.pdf]

S3 Fig. Knockdown assays for *HIF1A*, *DEC1*, and *DEC2* in HSC2 cells. Non-specific siRNA (siNS) or targeted siRNA was transfected into HSC-2 and cultured under normoxic or hypoxic conditions for 24 hours. Expression levels of *HIF1A*, *DEC1*, and *DEC2* were evaluated by real-time RT-PCR (left panel). Relative mRNA levels were calculated as the ratio to that of *ACTB*. Columns are the mean of three independent experiments; bars, SD. The differences between means were significant (ANOVA $P < 0.005$). P values calculated with Turkey-Kramer HSD test are: *, $P < 0.05$; **, $P < 0.01$; ***, $P < 0.001$. Expression levels of HIF-1α, DEC1, and DEC2 proteins were evaluated using immunostaining analysis (right panel).

S3 Fig

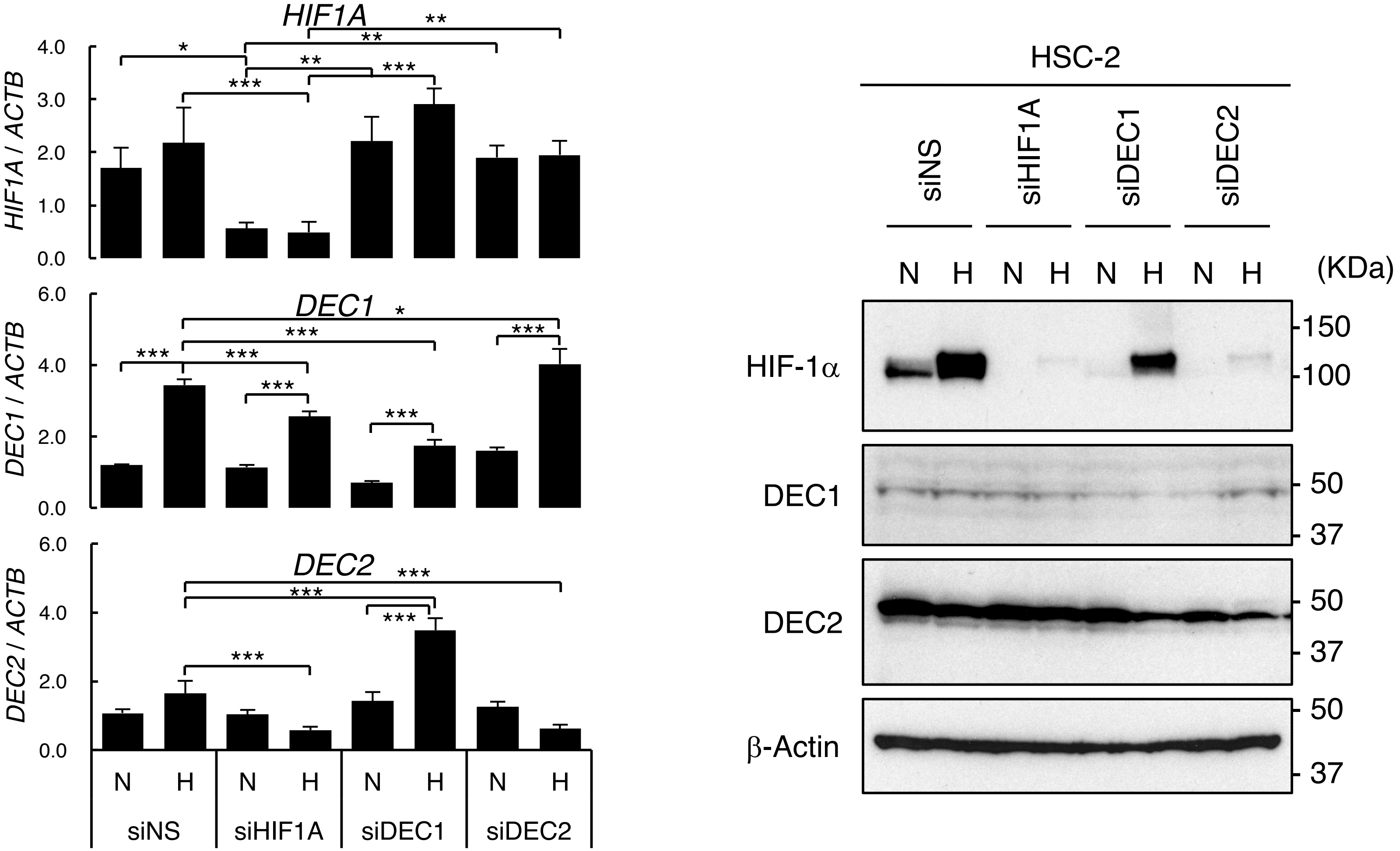
HIF1A
HIF1A / ACTB
DEC1
DEC1 / ACTB
DEC2
DEC2 / ACTB
N H N H N H N H
siNS siHIF1A siDEC1 siDEC2
HSC-2
siNS
siHIF1A
siDEC1
siDEC2
N H N H N H N H
(KDa)
HIF-1α
DEC1
DEC2
β-Actin
150
100
50
37
50
37
50
37
