# Supplementary material for: Differentiated embryo chondrocyte plays a crucial role in DNA damage response via transcriptional regulation under hypoxic conditions
Source: PLoS One. 2018 Feb 21;13(2):e0192136. doi: 10.1371/journal.pone.0192136 (PMC5821451; doi:10.1371/journal.pone.0192136)
Supplement: S4 Fig — Phosphorylate histone H2AX (γH2AX) levels after X-ray irradiation in HepG2 cells with or without pre-conditioning under hypoxia were evaluated with immunostaining analysis. The cells were stained with primary anti-γH2AX and secondary rhodamine-labeled antibody. Red shows γH2AX foci, and blue is nuclei stained with Hoechst 33342 (left panel). The right-side panel shows the number of γH2AX foci per cell. Total numbers of γH2AX foci and cells were counted in the left panel, and calculated. Columns are the mean of three independent counts; bars, SD. The differences between means were significant (ANOVA P < 0.005). P values calculated with Turkey-Kramer HSD test are: **, P < 0.01; ***, P < 0.001. (PDF) [file pone.0192136.s010.pdf]

S4 Fig. Phosphorylate histone H2AX induced by X-ray were inhibited in hypoxic pre-conditioned HepG2 cells. Phosphorylate histone H2AX (γH2AX) levels after X-ray irradiation in HepG2 cells with or without pre-conditioned under hypoxia were evaluated using immunostaining analysis. The cells were stained with primary anti-γH2AX and secondary rhodamine-labeled antibody. Red shows γH2AX foci, and blue is nuclei stained with Hoechst 33342 (left panel). The right-side panel shows the number of γH2AX foci per cell. Total numbers of γH2AX foci and cells were calculated in the left panel. Columns are the mean of three independent experiments; bars, SD. *P* values calculated with ANOVA test and Turkey-Kramer test are: **, $P < 0.01$; ***, $P < 0.001$.

S4 Fig

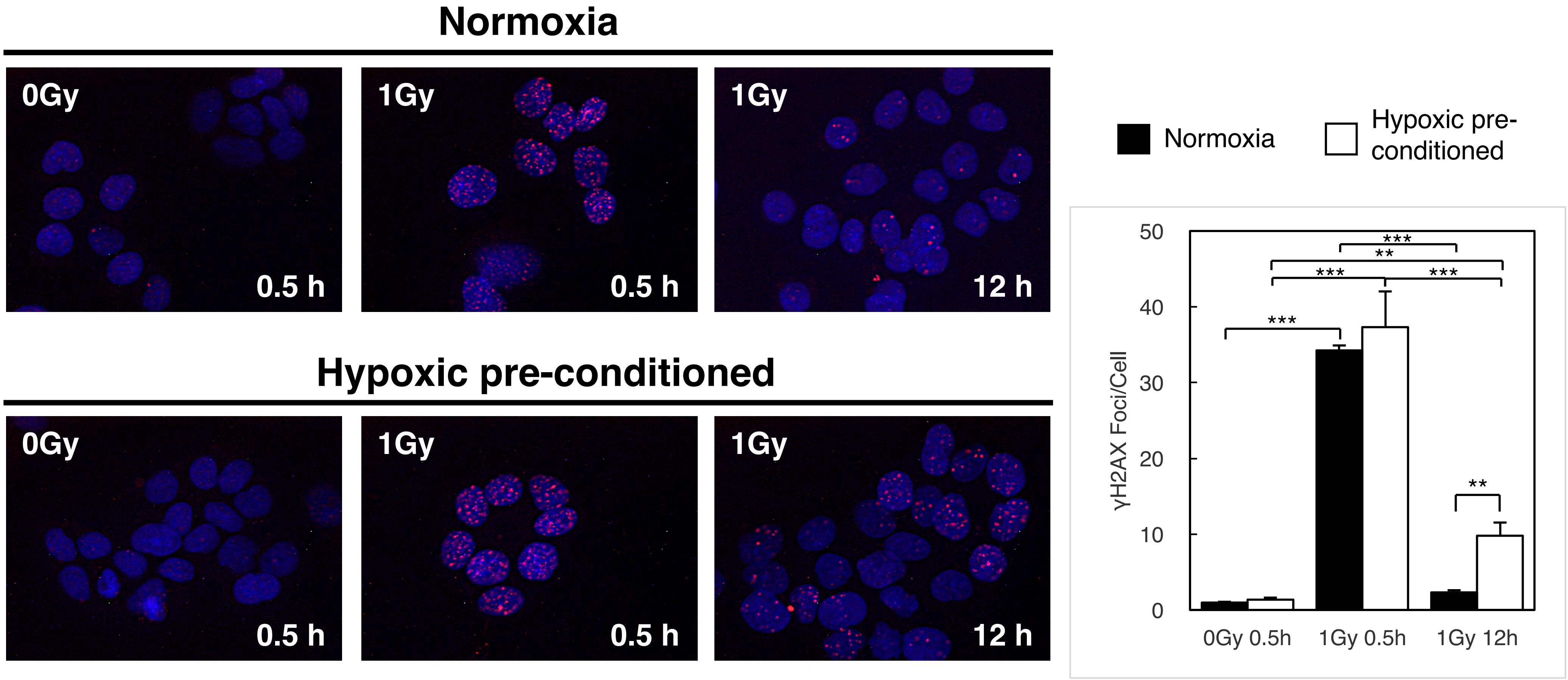

Normoxia
0Gy
0.5 h
1Gy
0.5 h
1Gy
12 h
Hypoxic pre-conditioned
0Gy
0.5 h
1Gy
0.5 h
1Gy
12 h
Normoxia
Hypoxic pre-conditioned
γH2AX Foci/Cell
50
40
30
20
10
0
***
**
***
***
***
**
0Gy 0.5h
1Gy 0.5h
1Gy 12h
