# Supplementary material for: Differentiated embryo chondrocyte plays a crucial role in DNA damage response via transcriptional regulation under hypoxic conditions
Source: PLoS One. 2018 Feb 21;13(2):e0192136. doi: 10.1371/journal.pone.0192136 (PMC5821451; doi:10.1371/journal.pone.0192136)
Supplement: S5 Fig — The pGL3-promoter reporters and DEC1 or DEC2 expression plasmid were co-transfected into HepG2 and treated with 10 or 100 ng/ml of Trichostatin A (TSA), histon deacetylase (HDAC) inhibitor, 24 hours before harvesting cells. Luciferase activities were determined with a dual-luciferase assay system. Columns are the mean of three independent experiments; bars, SD. The differences between means were significant (ANOVA P < 0.005). P values calculated with Turkey-Kramer HSD test are: *, P < 0.05; **, P < 0.01; ***, P < 0.001. (PDF) [file pone.0192136.s011.pdf]

S5 Fig. Suppressed promoter activities of DNA-DRR genes by DEC were increased with TSA treatments. The pGL3-promoter reporters and DEC1 or DEC2 expression plasmid were co-transfected into HepG2 and treated with 10 or 100 ng/ml of Trichostatin A (TSA), histon deacetylase (HDAC) inhibitor, 24 hours before harvesting cells. Luciferase activities were determined with a dual-luciferase assay system. Columns are the mean of three independent experiments; bars, SD. The differences between means were significant (ANOVA $P < 0.005$). P values calculated with Turkey-Kramer HSD test are: *, $P < 0.05$; **, $P < 0.01$; ***, $P < 0.001$.

S5 Fig

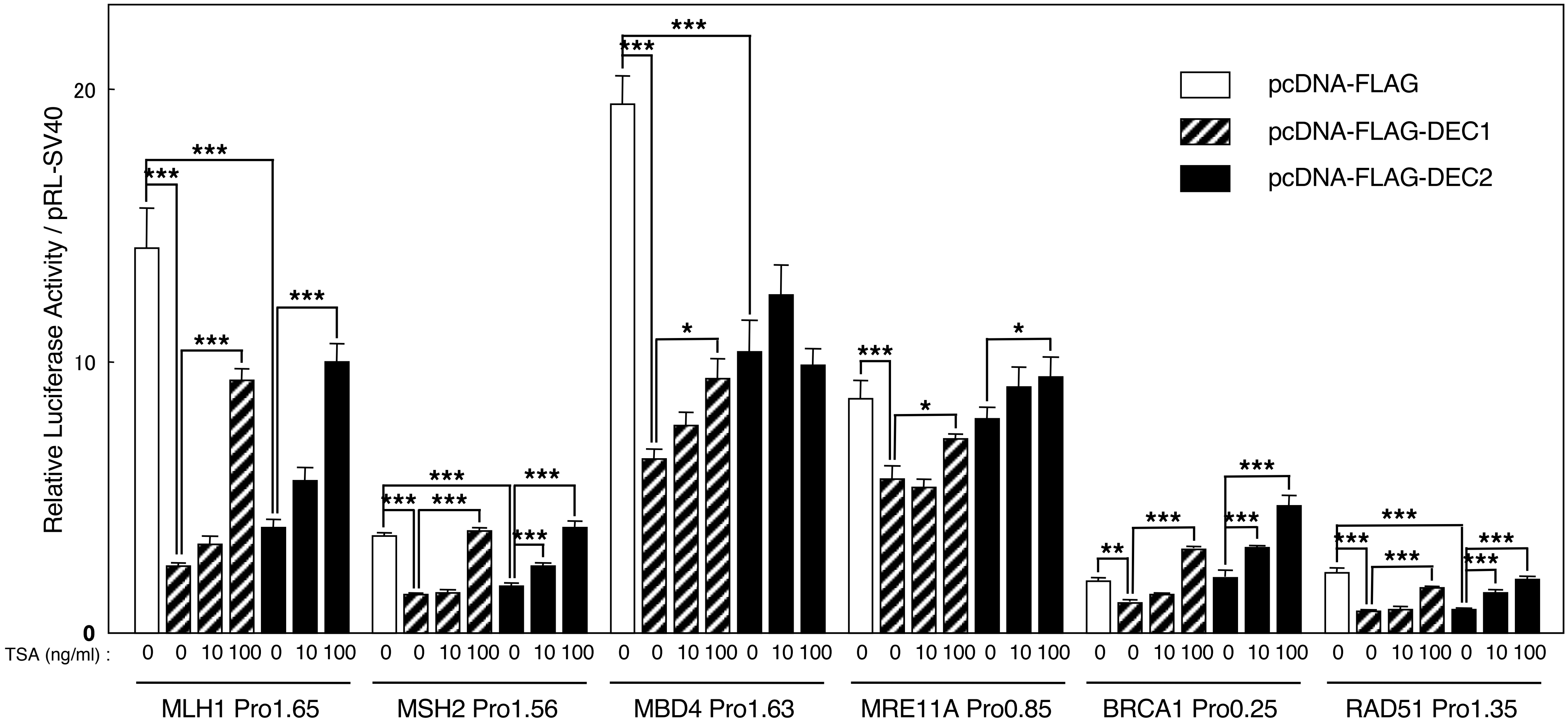

pcDNA-FLAG
pcDNA-FLAG-DEC1
pcDNA-FLAG-DEC2
Relative Luciferase Activity / pRL-SV40
0
10
20
TSA (ng/ml) :
0 0 10 100 0 10 100
MLH1 Pro1.65
MSH2 Pro1.56
MBD4 Pro1.63
MRE11A Pro0.85
BRCA1 Pro0.25
RAD51 Pro1.35
