# Supplementary material for: Differentiated embryo chondrocyte plays a crucial role in DNA damage response via transcriptional regulation under hypoxic conditions
Source: PLoS One. 2018 Feb 21;13(2):e0192136. doi: 10.1371/journal.pone.0192136 (PMC5821451; doi:10.1371/journal.pone.0192136)
Supplement: S6 Fig — Using the UCSC genome browser database, we analyzed the BHLHE40 ChIP-sequence peaks and signals near the transcription start site of already-reported DEC1 target genes, BHLHE41 (also known as DEC2), DBP, PER1, and PER2. (PDF) [file pone.0192136.s012.pdf]

S6 Fig. Known DEC1 target genes, *DEC2* (also known as *BHLHE41*), *DBP*, *PER1*, and *PER2*. Using the UCSC genome browser database, we analyzed the DEC1 (also known as BHLHE40) ChIP-sequence peaks and signals in the promoter region of the genes.

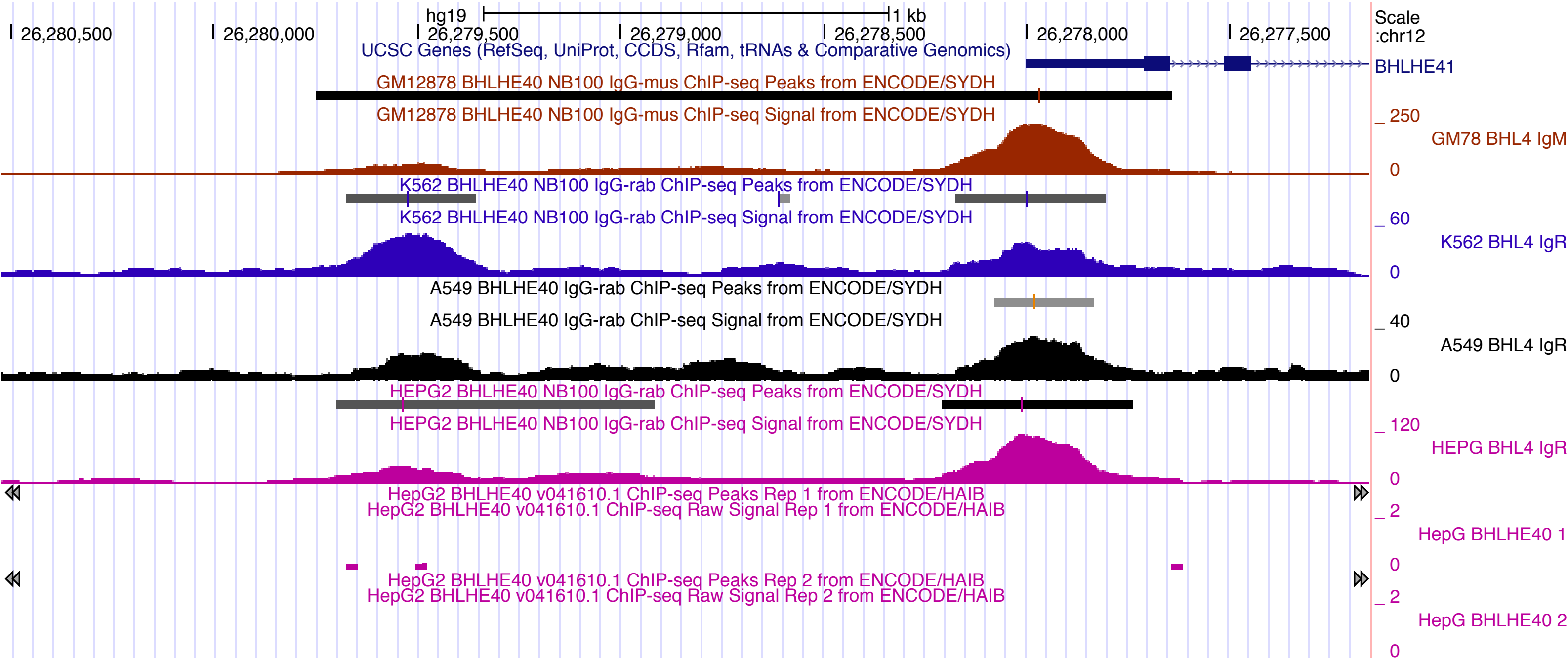

hg19
1 kb
Scale
:chr12
26,280,500
26,280,000
26,279,500
26,279,000
26,278,500
26,278,000
26,277,500
UCSC Genes (RefSeq, UniProt, CCDS, Rfam, tRNAs & Comparative Genomics)
BHLHE41
GM12878 BHLHE40 NB100 IgG-mus ChIP-seq Peaks from ENCODE/SYDH
GM12878 BHLHE40 NB100 IgG-mus ChIP-seq Signal from ENCODE/SYDH
250
GM78 BHL4 IgM
0
K562 BHLHE40 NB100 IgG-rab ChIP-seq Peaks from ENCODE/SYDH
K562 BHLHE40 NB100 IgG-rab ChIP-seq Signal from ENCODE/SYDH
60
K562 BHL4 IgR
0
A549 BHLHE40 IgG-rab ChIP-seq Peaks from ENCODE/SYDH
A549 BHLHE40 IgG-rab ChIP-seq Signal from ENCODE/SYDH
40
A549 BHL4 IgR
0
HEPG2 BHLHE40 NB100 IgG-rab ChIP-seq Peaks from ENCODE/SYDH
HEPG2 BHLHE40 NB100 IgG-rab ChIP-seq Signal from ENCODE/SYDH
120
HEPG BHL4 IgR
0
HepG2 BHLHE40 v041610.1 ChIP-seq Peaks Rep 1 from ENCODE/HAIB
HepG2 BHLHE40 v041610.1 ChIP-seq Raw Signal Rep 1 from ENCODE/HAIB
2
HepG BHLHE40 1
0
HepG2 BHLHE40 v041610.1 ChIP-seq Peaks Rep 2 from ENCODE/HAIB
HepG2 BHLHE40 v041610.1 ChIP-seq Raw Signal Rep 2 from ENCODE/HAIB
2
HepG BHLHE40 2
0

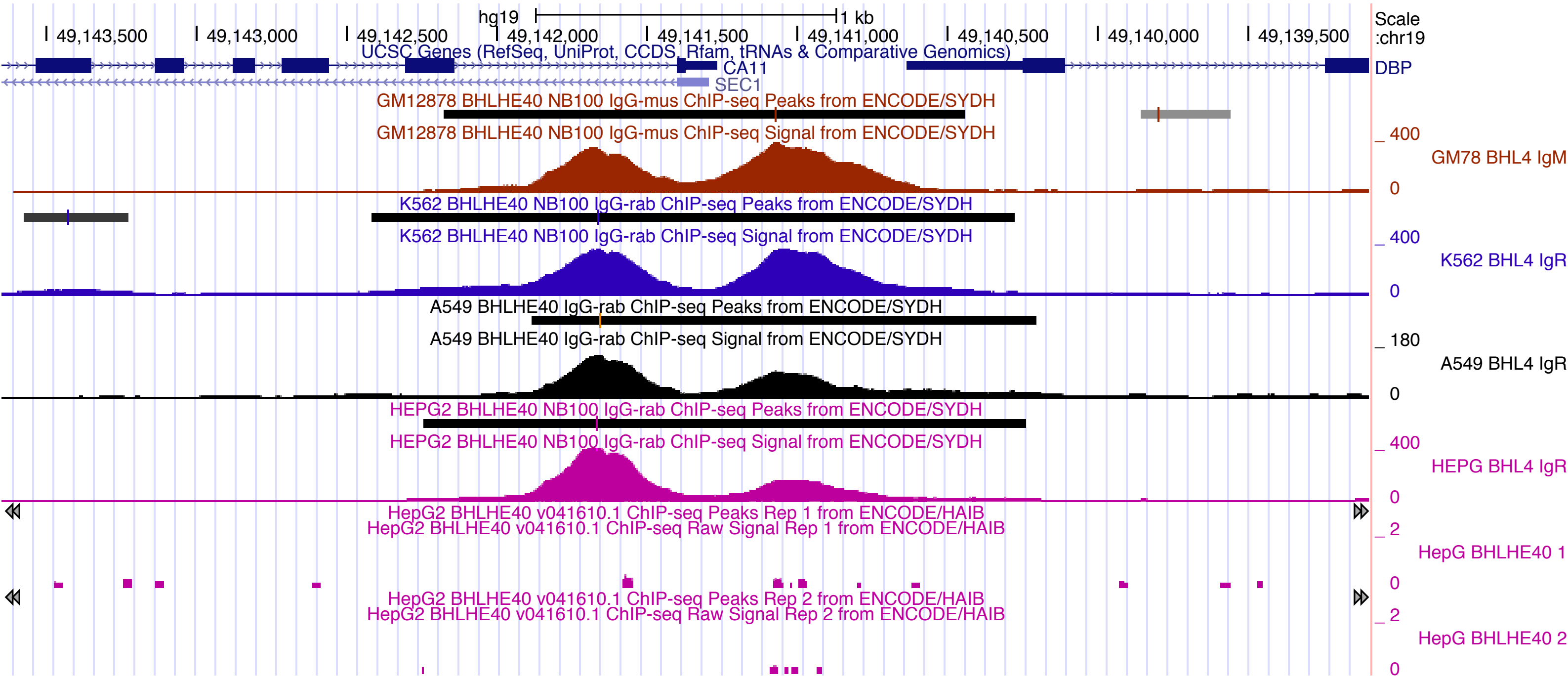

hg19
1 kb
Scale
:chr19
49,143,500
49,143,000
49,142,500
49,142,000
49,141,500
49,141,000
49,140,500
49,140,000
49,139,500
UCSC Genes (RefSeq, UniProt, CCDS, Rfam, tRNAs & Comparative Genomics)
CA11
SEC1
DBP
GM12878 BHLHE40 NB100 IgG-mus ChIP-seq Peaks from ENCODE/SYDH
GM12878 BHLHE40 NB100 IgG-mus ChIP-seq Signal from ENCODE/SYDH
400
GM78 BHL4 IgM
0
K562 BHLHE40 NB100 IgG-rab ChIP-seq Peaks from ENCODE/SYDH
K562 BHLHE40 NB100 IgG-rab ChIP-seq Signal from ENCODE/SYDH
400
K562 BHL4 IgR
0
A549 BHLHE40 IgG-rab ChIP-seq Peaks from ENCODE/SYDH
A549 BHLHE40 IgG-rab ChIP-seq Signal from ENCODE/SYDH
180
A549 BHL4 IgR
0
HEPG2 BHLHE40 NB100 IgG-rab ChIP-seq Peaks from ENCODE/SYDH
HEPG2 BHLHE40 NB100 IgG-rab ChIP-seq Signal from ENCODE/SYDH
400
HEPG BHL4 IgR
0
HepG2 BHLHE40 v041610.1 ChIP-seq Peaks Rep 1 from ENCODE/HAIB
HepG2 BHLHE40 v041610.1 ChIP-seq Raw Signal Rep 1 from ENCODE/HAIB
2
HepG BHLHE40 1
0
HepG2 BHLHE40 v041610.1 ChIP-seq Peaks Rep 2 from ENCODE/HAIB
HepG2 BHLHE40 v041610.1 ChIP-seq Raw Signal Rep 2 from ENCODE/HAIB
2
HepG BHLHE40 2
0

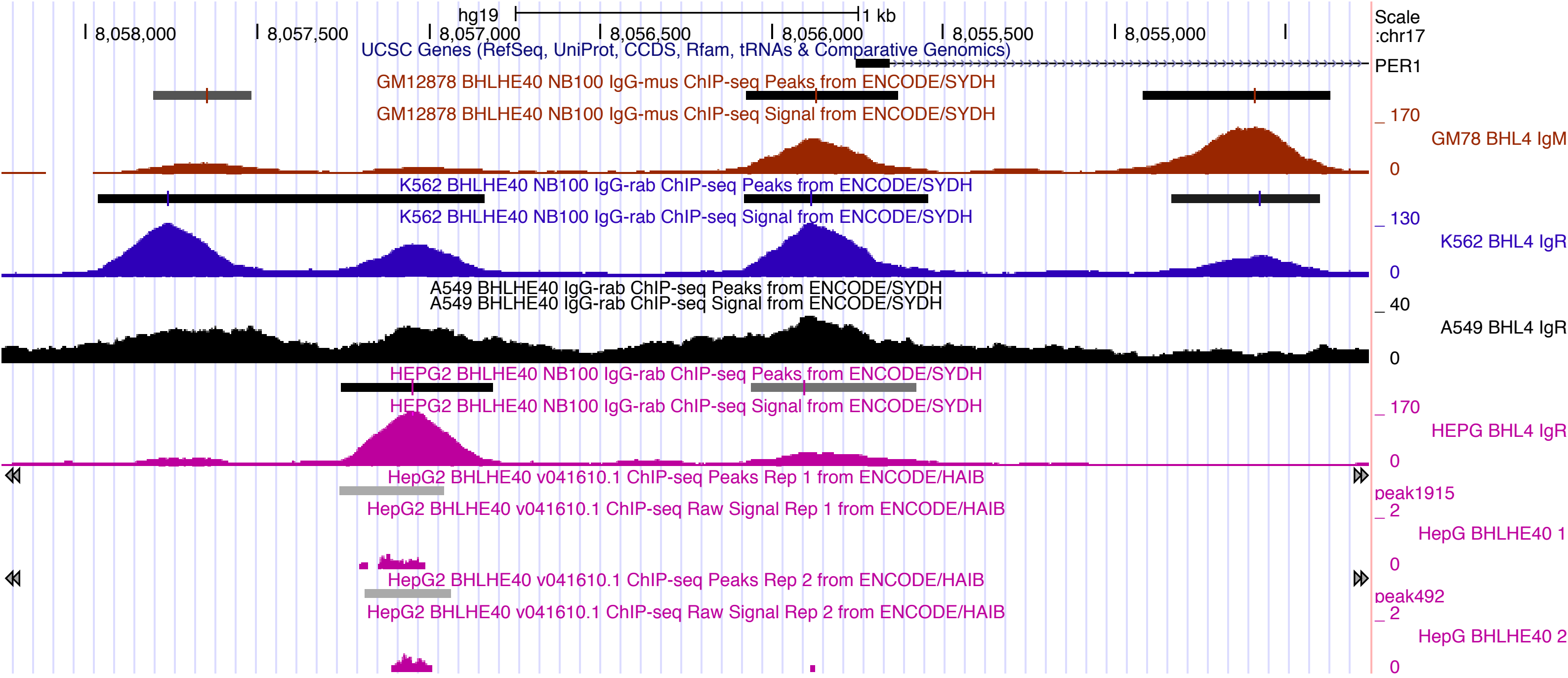

hg19
1 kb
Scale
:chr17
8,058,000
8,057,500
8,057,000
8,056,500
8,056,000
8,055,500
8,055,000
UCSC Genes (RefSeq, UniProt, CCDS, Rfam, tRNAs & Comparative Genomics)
PER1
GM12878 BHLHE40 NB100 IgG-mus ChIP-seq Peaks from ENCODE/SYDH
GM12878 BHLHE40 NB100 IgG-mus ChIP-seq Signal from ENCODE/SYDH
_ 170
GM78 BHL4 IgM
0
K562 BHLHE40 NB100 IgG-rab ChIP-seq Peaks from ENCODE/SYDH
K562 BHLHE40 NB100 IgG-rab ChIP-seq Signal from ENCODE/SYDH
_ 130
K562 BHL4 IgR
0
A549 BHLHE40 IgG-rab ChIP-seq Peaks from ENCODE/SYDH
A549 BHLHE40 IgG-rab ChIP-seq Signal from ENCODE/SYDH
_ 40
A549 BHL4 IgR
0
HEPG2 BHLHE40 NB100 IgG-rab ChIP-seq Peaks from ENCODE/SYDH
HEPG2 BHLHE40 NB100 IgG-rab ChIP-seq Signal from ENCODE/SYDH
_ 170
HEPG BHL4 IgR
0
HepG2 BHLHE40 v041610.1 ChIP-seq Peaks Rep 1 from ENCODE/HAIB
peak1915
HepG2 BHLHE40 v041610.1 ChIP-seq Raw Signal Rep 1 from ENCODE/HAIB
_ 2
HepG BHLHE40 1
0
HepG2 BHLHE40 v041610.1 ChIP-seq Peaks Rep 2 from ENCODE/HAIB
peak492
HepG2 BHLHE40 v041610.1 ChIP-seq Raw Signal Rep 2 from ENCODE/HAIB
_ 2
HepG BHLHE40 2
0

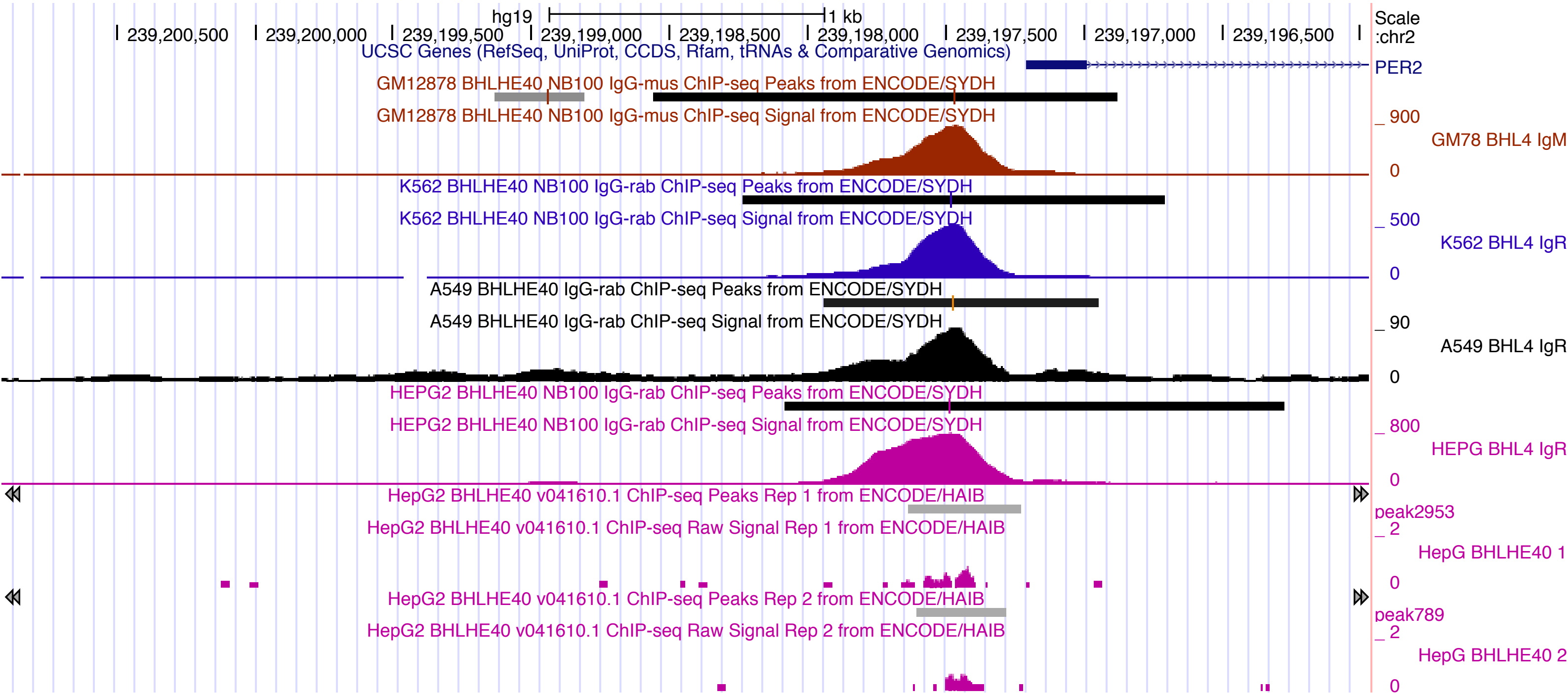

Scale
hg19
1 kb
:chr2
239,200,500
239,200,000
239,199,500
239,199,000
239,198,500
239,198,000
239,197,500
239,197,000
239,196,500
UCSC Genes (RefSeq, UniProt, CCDS, Rfam, tRNAs & Comparative Genomics)
PER2
GM12878 BHLHE40 NB100 IgG-mus ChIP-seq Peaks from ENCODE/SYDH
GM12878 BHLHE40 NB100 IgG-mus ChIP-seq Signal from ENCODE/SYDH
900
GM78 BHL4 IgM
0
K562 BHLHE40 NB100 IgG-rab ChIP-seq Peaks from ENCODE/SYDH
K562 BHLHE40 NB100 IgG-rab ChIP-seq Signal from ENCODE/SYDH
500
K562 BHL4 IgR
0
A549 BHLHE40 IgG-rab ChIP-seq Peaks from ENCODE/SYDH
A549 BHLHE40 IgG-rab ChIP-seq Signal from ENCODE/SYDH
90
A549 BHL4 IgR
0
HEPG2 BHLHE40 NB100 IgG-rab ChIP-seq Peaks from ENCODE/SYDH
HEPG2 BHLHE40 NB100 IgG-rab ChIP-seq Signal from ENCODE/SYDH
800
HEPG BHL4 IgR
0
HepG2 BHLHE40 v041610.1 ChIP-seq Peaks Rep 1 from ENCODE/HAIB
peak2953
HepG2 BHLHE40 v041610.1 ChIP-seq Raw Signal Rep 1 from ENCODE/HAIB
2
HepG BHLHE40 1
0
HepG2 BHLHE40 v041610.1 ChIP-seq Peaks Rep 2 from ENCODE/HAIB
peak789
HepG2 BHLHE40 v041610.1 ChIP-seq Raw Signal Rep 2 from ENCODE/HAIB
2
HepG BHLHE40 2
0
