# Supplementary material for: Differentiated embryo chondrocyte plays a crucial role in DNA damage response via transcriptional regulation under hypoxic conditions
Source: PLoS One. 2018 Feb 21;13(2):e0192136. doi: 10.1371/journal.pone.0192136 (PMC5821451; doi:10.1371/journal.pone.0192136)
Supplement: S7 Fig — Using the UCSC genome browser database, we analyzed the BHLHE40 ChIP-sequence peaks and signals near the transcription start site of HIF-1-DEC pathway-regulated genes, MLH1, STAT1, and SREBP-1c (also known as SREBF1). (PDF) [file pone.0192136.s013.pdf]

S7 Fig. Genes regulated by the HIF-1-DEC pathway, *MLH1*, *STAT1*, and *SREBF1* (also known as *SREBP1*). Using the UCSC genome browser database, we analyzed the DEC1 (also known as BHLHE40) ChIP-sequence peaks and signals in the promoter region of the genes.

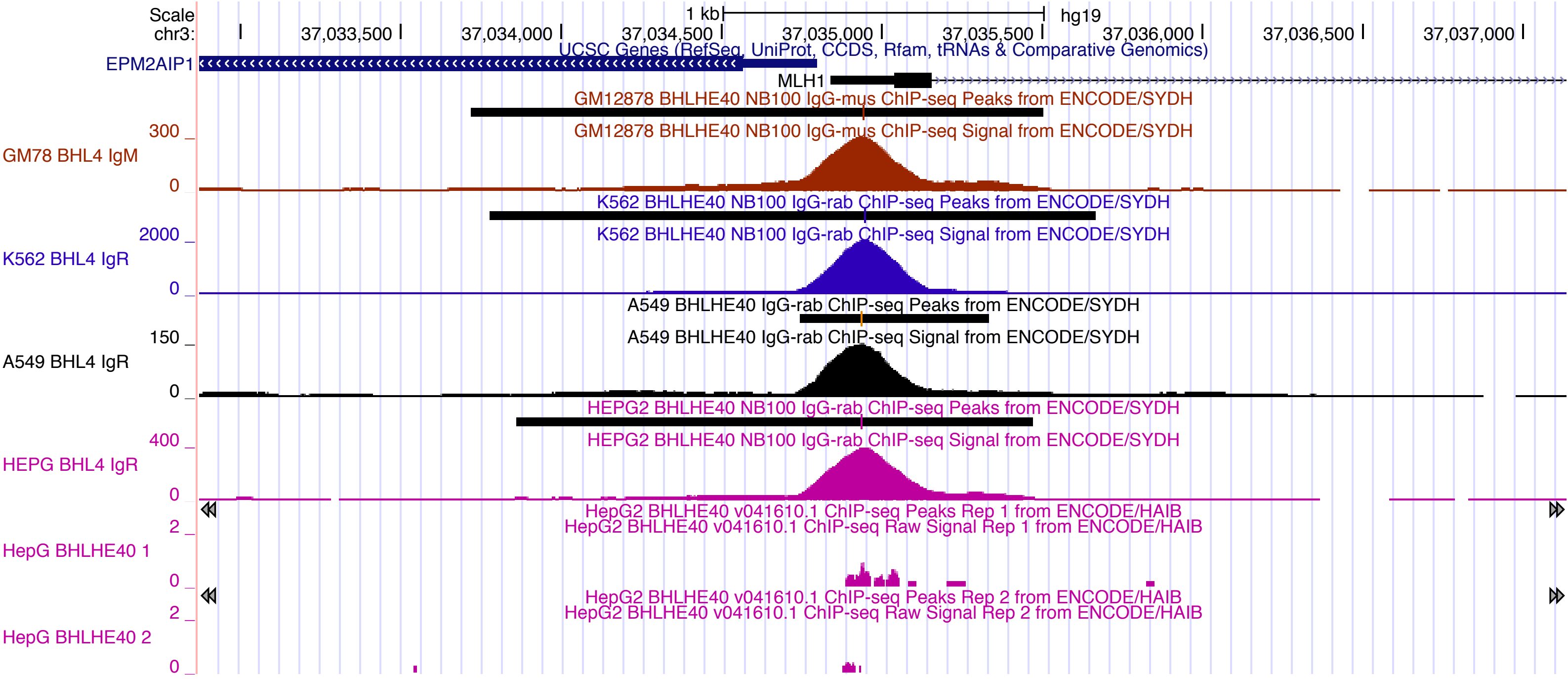

Scale
chr3:
1 kb
hg19
37,033,500
37,034,000
37,034,500
37,035,000
37,035,500
37,036,000
37,036,500
37,037,000
UCSC Genes (RefSeq, UniProt, CCDS, Rfam, tRNAs & Comparative Genomics)
EPM2AIP1
MLH1
GM12878 BHLHE40 NB100 IgG-mus ChIP-seq Peaks from ENCODE/SYDH
GM12878 BHLHE40 NB100 IgG-mus ChIP-seq Signal from ENCODE/SYDH
GM78 BHL4 IgM
300
0
K562 BHLHE40 NB100 IgG-rab ChIP-seq Peaks from ENCODE/SYDH
K562 BHLHE40 NB100 IgG-rab ChIP-seq Signal from ENCODE/SYDH
K562 BHL4 IgR
2000
0
A549 BHLHE40 IgG-rab ChIP-seq Peaks from ENCODE/SYDH
A549 BHLHE40 IgG-rab ChIP-seq Signal from ENCODE/SYDH
A549 BHL4 IgR
150
0
HEPG2 BHLHE40 NB100 IgG-rab ChIP-seq Peaks from ENCODE/SYDH
HEPG2 BHLHE40 NB100 IgG-rab ChIP-seq Signal from ENCODE/SYDH
HEPG BHL4 IgR
400
0
HepG2 BHLHE40 v041610.1 ChIP-seq Peaks Rep 1 from ENCODE/HAIB
HepG2 BHLHE40 v041610.1 ChIP-seq Raw Signal Rep 1 from ENCODE/HAIB
HepG BHLHE40 1
2
0
HepG2 BHLHE40 v041610.1 ChIP-seq Peaks Rep 2 from ENCODE/HAIB
HepG2 BHLHE40 v041610.1 ChIP-seq Raw Signal Rep 2 from ENCODE/HAIB
HepG BHLHE40 2
2
0

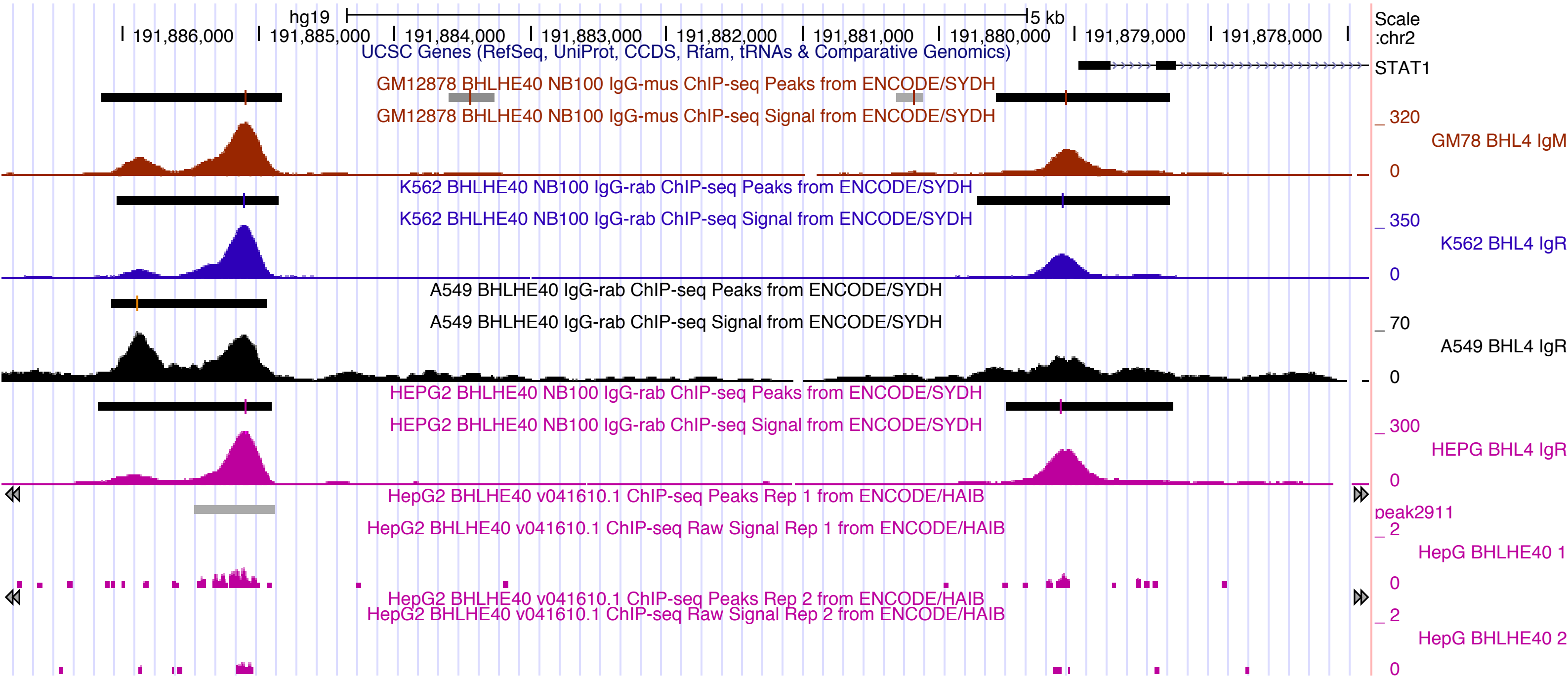

hg19
5 kb
Scale
:chr2
191,886,000
191,885,000
191,884,000
191,883,000
191,882,000
191,881,000
191,880,000
191,879,000
191,878,000
UCSC Genes (RefSeq, UniProt, CCDS, Rfam, tRNAs & Comparative Genomics)
STAT1
GM12878 BHLHE40 NB100 IgG-mus ChIP-seq Peaks from ENCODE/SYDH
GM12878 BHLHE40 NB100 IgG-mus ChIP-seq Signal from ENCODE/SYDH
320
GM78 BHL4 IgM
0
K562 BHLHE40 NB100 IgG-rab ChIP-seq Peaks from ENCODE/SYDH
K562 BHLHE40 NB100 IgG-rab ChIP-seq Signal from ENCODE/SYDH
350
K562 BHL4 IgR
0
A549 BHLHE40 IgG-rab ChIP-seq Peaks from ENCODE/SYDH
A549 BHLHE40 IgG-rab ChIP-seq Signal from ENCODE/SYDH
70
A549 BHL4 IgR
0
HEPG2 BHLHE40 NB100 IgG-rab ChIP-seq Peaks from ENCODE/SYDH
HEPG2 BHLHE40 NB100 IgG-rab ChIP-seq Signal from ENCODE/SYDH
300
HEPG BHL4 IgR
0
HepG2 BHLHE40 v041610.1 ChIP-seq Peaks Rep 1 from ENCODE/HAIB
peak2911
HepG2 BHLHE40 v041610.1 ChIP-seq Raw Signal Rep 1 from ENCODE/HAIB
2
HepG BHLHE40 1
0
HepG2 BHLHE40 v041610.1 ChIP-seq Peaks Rep 2 from ENCODE/HAIB
HepG2 BHLHE40 v041610.1 ChIP-seq Raw Signal Rep 2 from ENCODE/HAIB
2
HepG BHLHE40 2
0

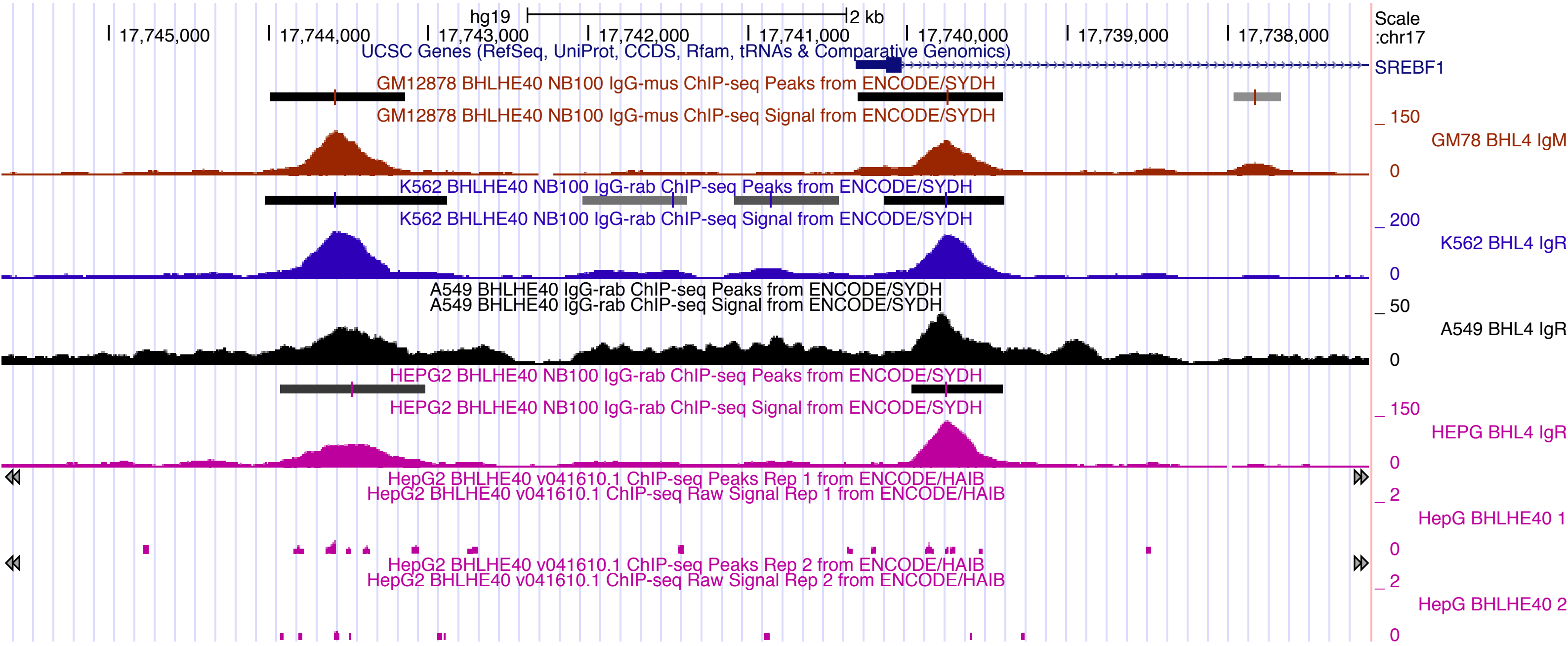

hg19
2 kb
Scale
:chr17
17,745,000
17,744,000
17,743,000
17,742,000
17,741,000
17,740,000
17,739,000
17,738,000
UCSC Genes (RefSeq, UniProt, CCDS, Rfam, tRNAs & Comparative Genomics)
SREBF1
GM12878 BHLHE40 NB100 IgG-mus ChIP-seq Peaks from ENCODE/SYDH
GM12878 BHLHE40 NB100 IgG-mus ChIP-seq Signal from ENCODE/SYDH
_ 150
GM78 BHL4 IgM
0
K562 BHLHE40 NB100 IgG-rab ChIP-seq Peaks from ENCODE/SYDH
K562 BHLHE40 NB100 IgG-rab ChIP-seq Signal from ENCODE/SYDH
_ 200
K562 BHL4 IgR
0
A549 BHLHE40 IgG-rab ChIP-seq Peaks from ENCODE/SYDH
A549 BHLHE40 IgG-rab ChIP-seq Signal from ENCODE/SYDH
_ 50
A549 BHL4 IgR
0
HEPG2 BHLHE40 NB100 IgG-rab ChIP-seq Peaks from ENCODE/SYDH
HEPG2 BHLHE40 NB100 IgG-rab ChIP-seq Signal from ENCODE/SYDH
_ 150
HEPG BHL4 IgR
0
HepG2 BHLHE40 v041610.1 ChIP-seq Peaks Rep 1 from ENCODE/HAIB
HepG2 BHLHE40 v041610.1 ChIP-seq Raw Signal Rep 1 from ENCODE/HAIB
_ 2
HepG BHLHE40 1
0
HepG2 BHLHE40 v041610.1 ChIP-seq Peaks Rep 2 from ENCODE/HAIB
HepG2 BHLHE40 v041610.1 ChIP-seq Raw Signal Rep 2 from ENCODE/HAIB
_ 2
HepG BHLHE40 2
0
