# Supplementary material for: Differentiated embryo chondrocyte plays a crucial role in DNA damage response via transcriptional regulation under hypoxic conditions
Source: PLoS One. 2018 Feb 21;13(2):e0192136. doi: 10.1371/journal.pone.0192136 (PMC5821451; doi:10.1371/journal.pone.0192136)
Supplement: S8 Fig — Using the UCSC genome browser database, we analyzed the BHLHE40 ChIP-sequence peaks and signals near the transcription start site of genes related to DNA-DRR listed in S3 Table. (PDF) [file pone.0192136.s014.pdf]

**S8 Fig.** Genes related to DNA-DRR listed in S3 Table. Using the UCSC genome browser database, we analyzed the DEC1 (also known as BHLHE40) ChIP-sequence peaks and signals in the promoter region of the genes.

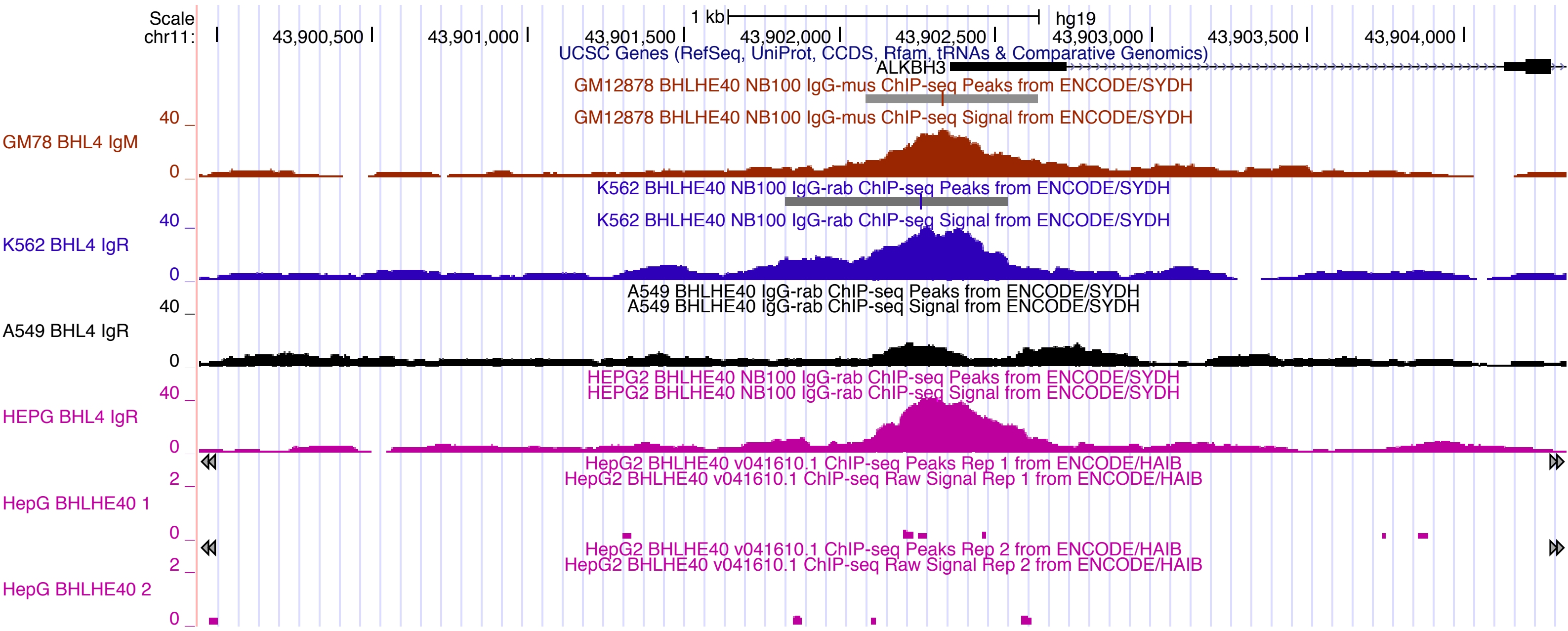

Scale
chr11:
1 kb
hg19
43,900,500
43,901,000
43,901,500
43,902,000
43,902,500
43,903,000
43,903,500
43,904,000
UCSC Genes (RefSeq, UniProt, CCDS, Rfam, tRNAs & Comparative Genomics)
ALKBH3
GM12878 BHLHE40 NB100 IgG-mus ChIP-seq Peaks from ENCODE/SYDH
GM12878 BHLHE40 NB100 IgG-mus ChIP-seq Signal from ENCODE/SYDH
GM78 BHL4 IgM
K562 BHLHE40 NB100 IgG-rab ChIP-seq Peaks from ENCODE/SYDH
K562 BHLHE40 NB100 IgG-rab ChIP-seq Signal from ENCODE/SYDH
K562 BHL4 IgR
A549 BHLHE40 IgG-rab ChIP-seq Peaks from ENCODE/SYDH
A549 BHLHE40 IgG-rab ChIP-seq Signal from ENCODE/SYDH
A549 BHL4 IgR
HEPG2 BHLHE40 NB100 IgG-rab ChIP-seq Peaks from ENCODE/SYDH
HEPG2 BHLHE40 NB100 IgG-rab ChIP-seq Signal from ENCODE/SYDH
HEPG BHL4 IgR
HepG2 BHLHE40 v041610.1 ChIP-seq Peaks Rep 1 from ENCODE/HAIB
HepG2 BHLHE40 v041610.1 ChIP-seq Raw Signal Rep 1 from ENCODE/HAIB
HepG BHLHE40 1
HepG2 BHLHE40 v041610.1 ChIP-seq Peaks Rep 2 from ENCODE/HAIB
HepG2 BHLHE40 v041610.1 ChIP-seq Raw Signal Rep 2 from ENCODE/HAIB
HepG BHLHE40 2

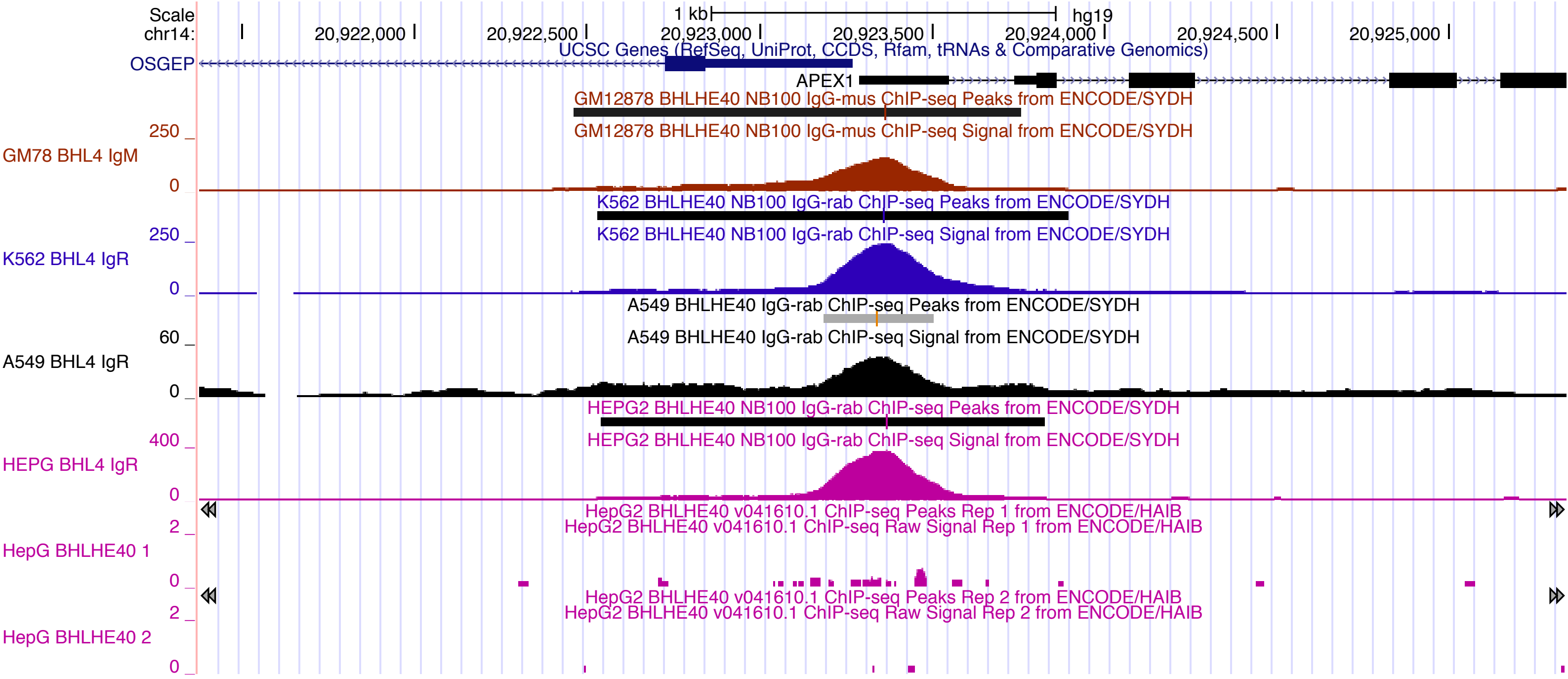
Scale
chr14:
1 kb
hg19
20,922,000
20,922,500
20,923,000
20,923,500
20,924,000
20,924,500
20,925,000
UCSC Genes (RefSeq, UniProt, CCDS, Rfam, tRNAs & Comparative Genomics)
OSGEP
APEX1
GM12878 BHLHE40 NB100 IgG-mus ChIP-seq Peaks from ENCODE/SYDH
GM12878 BHLHE40 NB100 IgG-mus ChIP-seq Signal from ENCODE/SYDH
GM78 BHL4 IgM
250
0
K562 BHLHE40 NB100 IgG-rab ChIP-seq Peaks from ENCODE/SYDH
K562 BHLHE40 NB100 IgG-rab ChIP-seq Signal from ENCODE/SYDH
K562 BHL4 IgR
250
0
A549 BHLHE40 IgG-rab ChIP-seq Peaks from ENCODE/SYDH
A549 BHLHE40 IgG-rab ChIP-seq Signal from ENCODE/SYDH
A549 BHL4 IgR
60
0
HEPG2 BHLHE40 NB100 IgG-rab ChIP-seq Peaks from ENCODE/SYDH
HEPG2 BHLHE40 NB100 IgG-rab ChIP-seq Signal from ENCODE/SYDH
HEPG BHL4 IgR
400
0
HepG2 BHLHE40 v041610.1 ChIP-seq Peaks Rep 1 from ENCODE/HAIB
HepG2 BHLHE40 v041610.1 ChIP-seq Raw Signal Rep 1 from ENCODE/HAIB
HepG BHLHE40 1
2
0
HepG2 BHLHE40 v041610.1 ChIP-seq Peaks Rep 2 from ENCODE/HAIB
HepG2 BHLHE40 v041610.1 ChIP-seq Raw Signal Rep 2 from ENCODE/HAIB
HepG BHLHE40 2
2
0

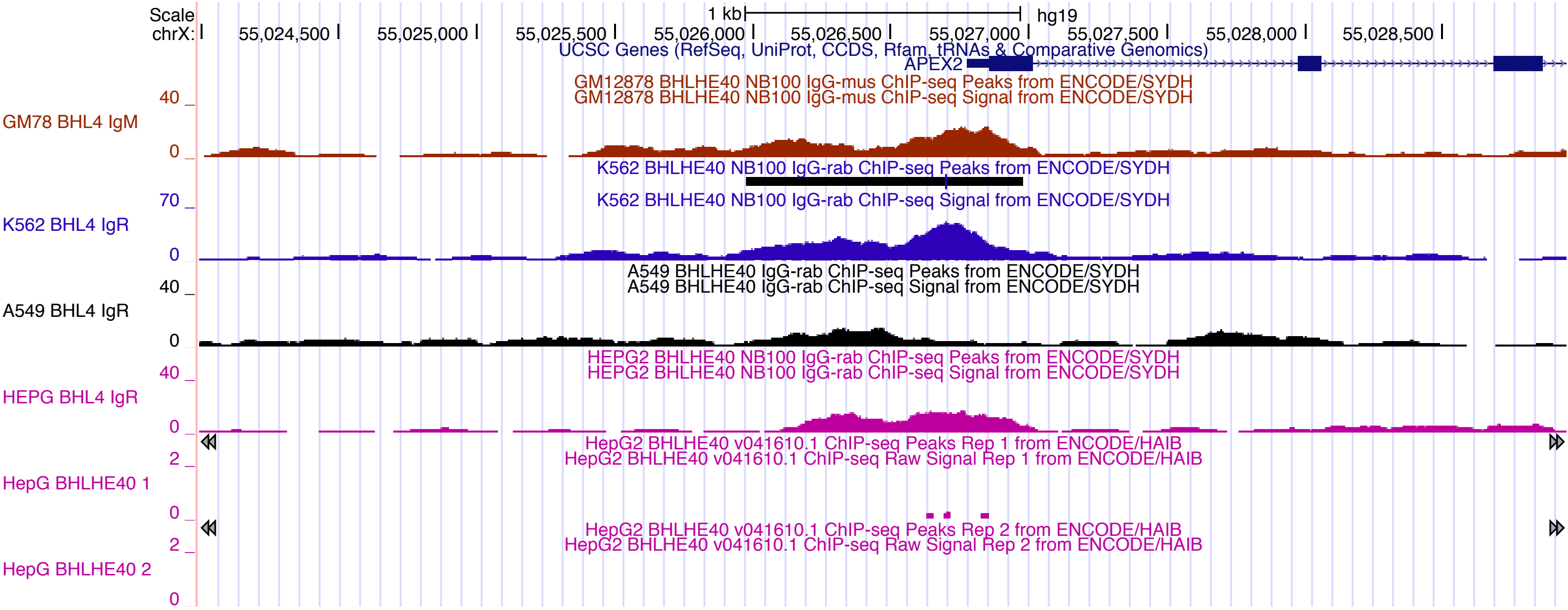
Scale
chrX:
1 kb
hg19
55,024,500
55,025,000
55,025,500
55,026,000
55,026,500
55,027,000
55,027,500
55,028,000
55,028,500
UCSC Genes (RefSeq, UniProt, CCDS, Rfam, tRNAs & Comparative Genomics)
APEX2
GM12878 BHLHE40 NB100 IgG-mus ChIP-seq Peaks from ENCODE/SYDH
GM12878 BHLHE40 NB100 IgG-mus ChIP-seq Signal from ENCODE/SYDH
GM78 BHL4 IgM
K562 BHLHE40 NB100 IgG-rab ChIP-seq Peaks from ENCODE/SYDH
K562 BHLHE40 NB100 IgG-rab ChIP-seq Signal from ENCODE/SYDH
K562 BHL4 IgR
A549 BHLHE40 IgG-rab ChIP-seq Peaks from ENCODE/SYDH
A549 BHLHE40 IgG-rab ChIP-seq Signal from ENCODE/SYDH
A549 BHL4 IgR
HEPG2 BHLHE40 NB100 IgG-rab ChIP-seq Peaks from ENCODE/SYDH
HEPG2 BHLHE40 NB100 IgG-rab ChIP-seq Signal from ENCODE/SYDH
HEPG BHL4 IgR
HepG2 BHLHE40 v041610.1 ChIP-seq Peaks Rep 1 from ENCODE/HAIB
HepG2 BHLHE40 v041610.1 ChIP-seq Raw Signal Rep 1 from ENCODE/HAIB
HepG BHLHE40 1
HepG2 BHLHE40 v041610.1 ChIP-seq Peaks Rep 2 from ENCODE/HAIB
HepG2 BHLHE40 v041610.1 ChIP-seq Raw Signal Rep 2 from ENCODE/HAIB
HepG BHLHE40 2

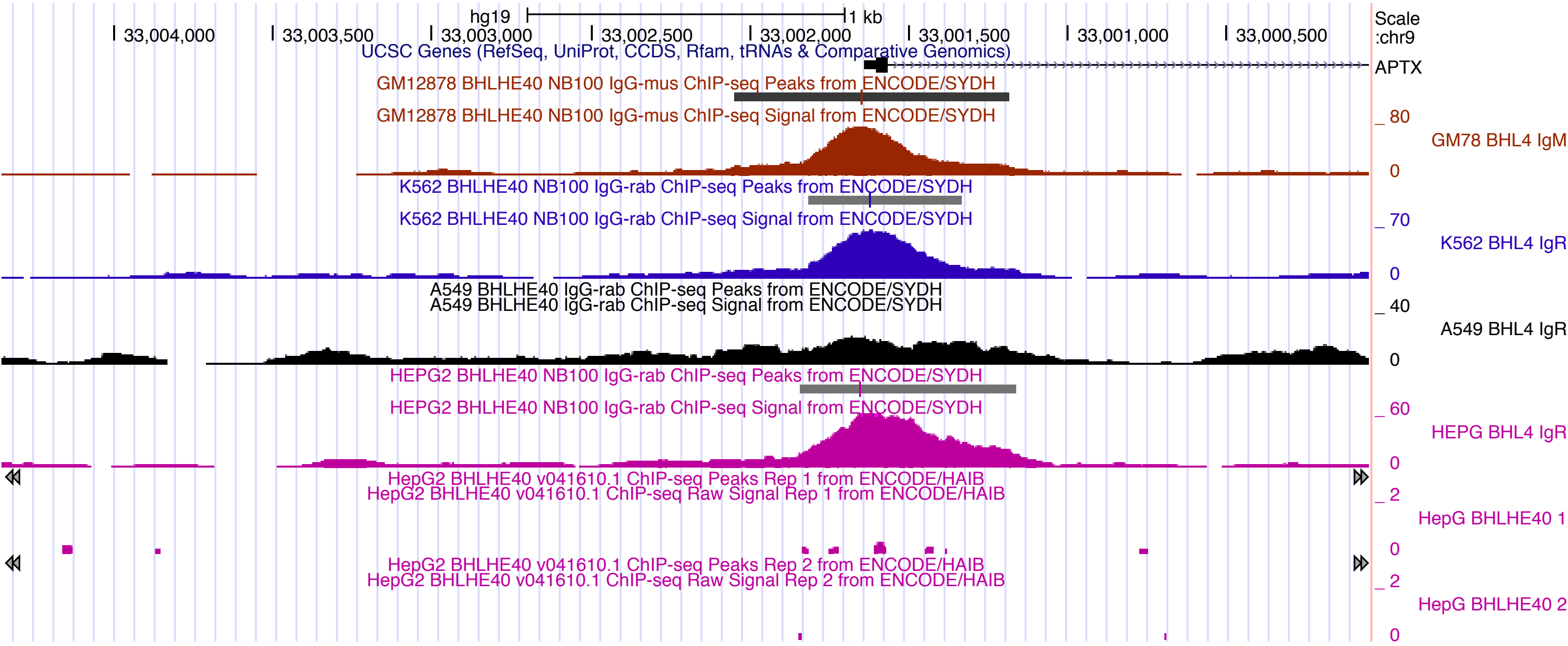

hg19
1 kb
Scale
:chr9
33,004,000
33,003,500
33,003,000
33,002,500
33,002,000
33,001,500
33,001,000
33,000,500
UCSC Genes (RefSeq, UniProt, CCDS, Rfam, tRNAs & Comparative Genomics)
APTX
GM12878 BHLHE40 NB100 IgG-mus ChIP-seq Peaks from ENCODE/SYDH
GM12878 BHLHE40 NB100 IgG-mus ChIP-seq Signal from ENCODE/SYDH
_ 80
GM78 BHL4 IgM
0
K562 BHLHE40 NB100 IgG-rab ChIP-seq Peaks from ENCODE/SYDH
K562 BHLHE40 NB100 IgG-rab ChIP-seq Signal from ENCODE/SYDH
_ 70
K562 BHL4 IgR
0
A549 BHLHE40 IgG-rab ChIP-seq Peaks from ENCODE/SYDH
A549 BHLHE40 IgG-rab ChIP-seq Signal from ENCODE/SYDH
_ 40
A549 BHL4 IgR
0
HEPG2 BHLHE40 NB100 IgG-rab ChIP-seq Peaks from ENCODE/SYDH
HEPG2 BHLHE40 NB100 IgG-rab ChIP-seq Signal from ENCODE/SYDH
_ 60
HEPG BHL4 IgR
0
HepG2 BHLHE40 v041610.1 ChIP-seq Peaks Rep 1 from ENCODE/HAIB
HepG2 BHLHE40 v041610.1 ChIP-seq Raw Signal Rep 1 from ENCODE/HAIB
_ 2
HepG BHLHE40 1
0
HepG2 BHLHE40 v041610.1 ChIP-seq Peaks Rep 2 from ENCODE/HAIB
HepG2 BHLHE40 v041610.1 ChIP-seq Raw Signal Rep 2 from ENCODE/HAIB
_ 2
HepG BHLHE40 2
0

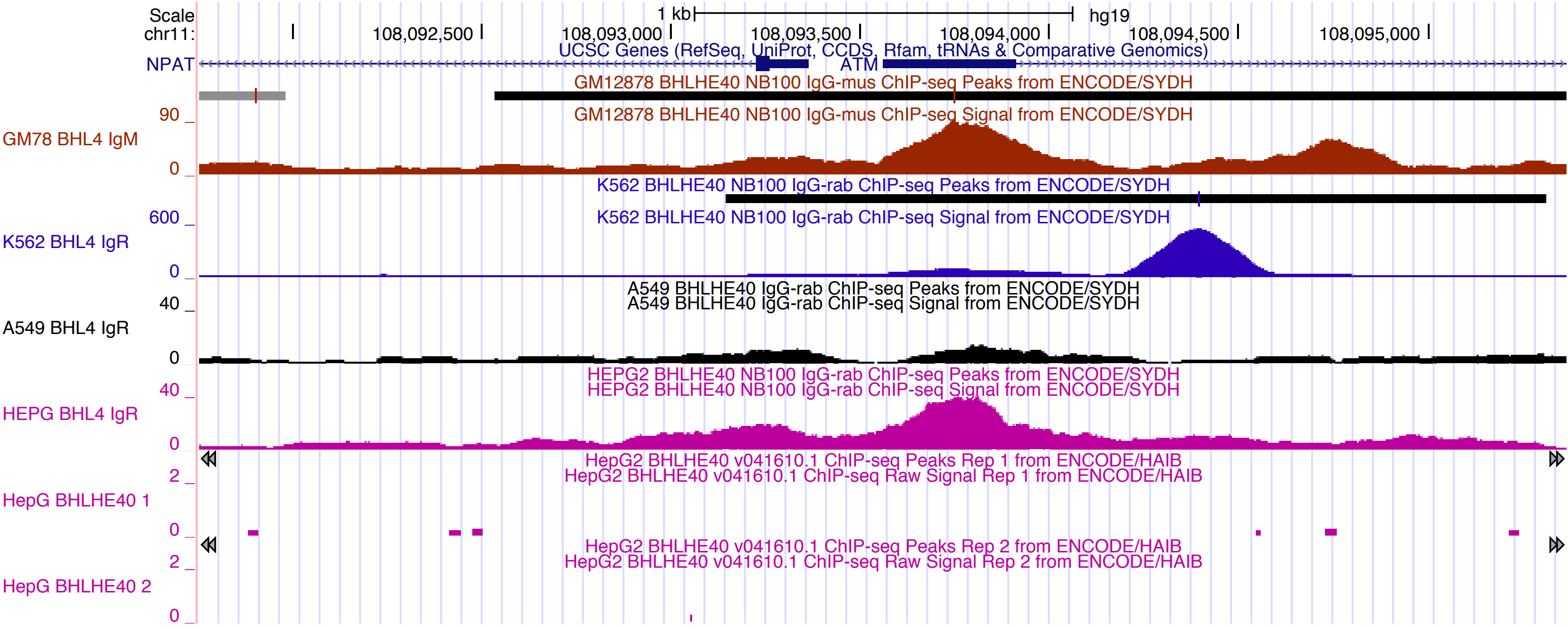
Scale
chr11:
1 kb
hg19
108,092,500
108,093,000
108,093,500
108,094,000
108,094,500
108,095,000
UCSC Genes (RefSeq, UniProt, CCDS, Rfam, tRNAs & Comparative Genomics)
NPAT
ATM
GM12878 BHLHE40 NB100 IgG-mus ChIP-seq Peaks from ENCODE/SYDH
GM12878 BHLHE40 NB100 IgG-mus ChIP-seq Signal from ENCODE/SYDH
GM78 BHL4 IgM
90
0
K562 BHLHE40 NB100 IgG-rab ChIP-seq Peaks from ENCODE/SYDH
K562 BHLHE40 NB100 IgG-rab ChIP-seq Signal from ENCODE/SYDH
K562 BHL4 IgR
600
0
A549 BHLHE40 IgG-rab ChIP-seq Peaks from ENCODE/SYDH
A549 BHLHE40 IgG-rab ChIP-seq Signal from ENCODE/SYDH
A549 BHL4 IgR
40
0
HEPG2 BHLHE40 NB100 IgG-rab ChIP-seq Peaks from ENCODE/SYDH
HEPG2 BHLHE40 NB100 IgG-rab ChIP-seq Signal from ENCODE/SYDH
HEPG BHL4 IgR
40
0
HepG2 BHLHE40 v041610.1 ChIP-seq Peaks Rep 1 from ENCODE/HAIB
HepG2 BHLHE40 v041610.1 ChIP-seq Raw Signal Rep 1 from ENCODE/HAIB
HepG BHLHE40 1
2
0
HepG2 BHLHE40 v041610.1 ChIP-seq Peaks Rep 2 from ENCODE/HAIB
HepG2 BHLHE40 v041610.1 ChIP-seq Raw Signal Rep 2 from ENCODE/HAIB
HepG BHLHE40 2
2
0

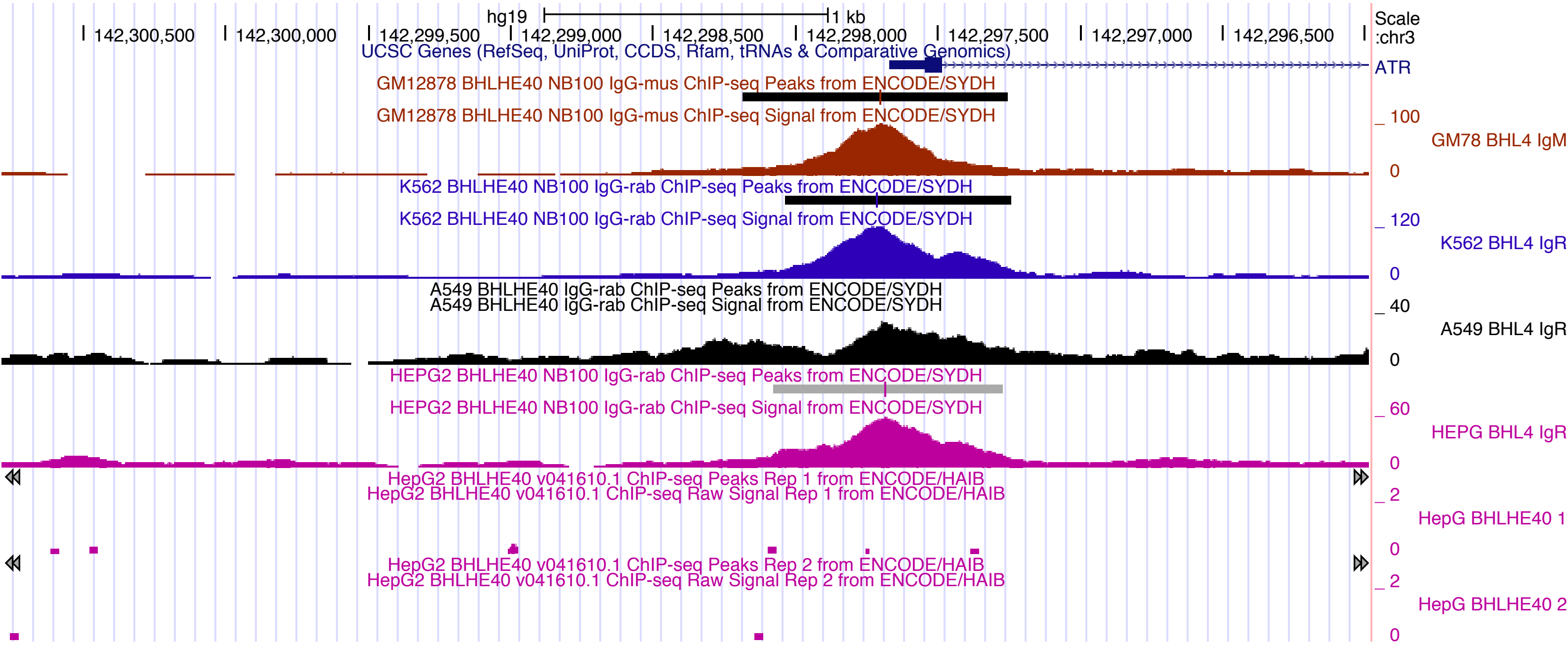
hg19
1 kb
Scale
:chr3
142,300,500
142,300,000
142,299,500
142,299,000
142,298,500
142,298,000
142,297,500
142,297,000
142,296,500
UCSC Genes (RefSeq, UniProt, CCDS, Rfam, tRNAs & Comparative Genomics)
ATR
GM12878 BHLHE40 NB100 IgG-mus ChIP-seq Peaks from ENCODE/SYDH
GM12878 BHLHE40 NB100 IgG-mus ChIP-seq Signal from ENCODE/SYDH
GM78 BHL4 IgM
K562 BHLHE40 NB100 IgG-rab ChIP-seq Peaks from ENCODE/SYDH
K562 BHLHE40 NB100 IgG-rab ChIP-seq Signal from ENCODE/SYDH
K562 BHL4 IgR
A549 BHLHE40 IgG-rab ChIP-seq Peaks from ENCODE/SYDH
A549 BHLHE40 IgG-rab ChIP-seq Signal from ENCODE/SYDH
A549 BHL4 IgR
HEPG2 BHLHE40 NB100 IgG-rab ChIP-seq Peaks from ENCODE/SYDH
HEPG2 BHLHE40 NB100 IgG-rab ChIP-seq Signal from ENCODE/SYDH
HEPG BHL4 IgR
HepG2 BHLHE40 v041610.1 ChIP-seq Peaks Rep 1 from ENCODE/HAIB
HepG2 BHLHE40 v041610.1 ChIP-seq Raw Signal Rep 1 from ENCODE/HAIB
HepG BHLHE40 1
HepG2 BHLHE40 v041610.1 ChIP-seq Peaks Rep 2 from ENCODE/HAIB
HepG2 BHLHE40 v041610.1 ChIP-seq Raw Signal Rep 2 from ENCODE/HAIB
HepG BHLHE40 2
100
0
120
0
40
0
60
0
2
0
2
0

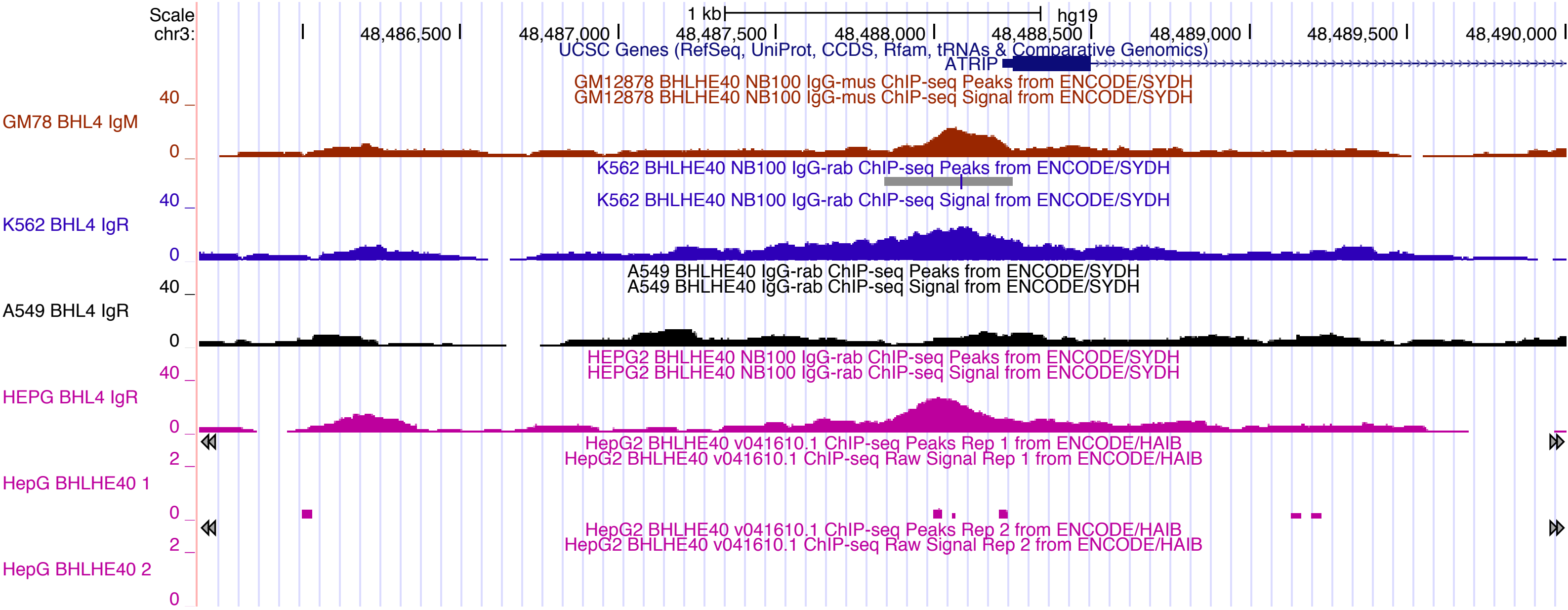

Scale
chr3:
1 kb
hg19
48,486,500
48,487,000
48,487,500
48,488,000
48,488,500
48,489,000
48,489,500
48,490,000
UCSC Genes (RefSeq, UniProt, CCDS, Rfam, tRNAs & Comparative Genomics)
ATRIP
GM12878 BHLHE40 NB100 IgG-mus ChIP-seq Peaks from ENCODE/SYDH
GM12878 BHLHE40 NB100 IgG-mus ChIP-seq Signal from ENCODE/SYDH
GM78 BHL4 IgM
K562 BHLHE40 NB100 IgG-rab ChIP-seq Peaks from ENCODE/SYDH
K562 BHLHE40 NB100 IgG-rab ChIP-seq Signal from ENCODE/SYDH
K562 BHL4 IgR
A549 BHLHE40 IgG-rab ChIP-seq Peaks from ENCODE/SYDH
A549 BHLHE40 IgG-rab ChIP-seq Signal from ENCODE/SYDH
A549 BHL4 IgR
HEPG2 BHLHE40 NB100 IgG-rab ChIP-seq Peaks from ENCODE/SYDH
HEPG2 BHLHE40 NB100 IgG-rab ChIP-seq Signal from ENCODE/SYDH
HEPG BHL4 IgR
HepG2 BHLHE40 v041610.1 ChIP-seq Peaks Rep 1 from ENCODE/HAIB
HepG2 BHLHE40 v041610.1 ChIP-seq Raw Signal Rep 1 from ENCODE/HAIB
HepG BHLHE40 1
HepG2 BHLHE40 v041610.1 ChIP-seq Peaks Rep 2 from ENCODE/HAIB
HepG2 BHLHE40 v041610.1 ChIP-seq Raw Signal Rep 2 from ENCODE/HAIB
HepG BHLHE40 2
40
0
2

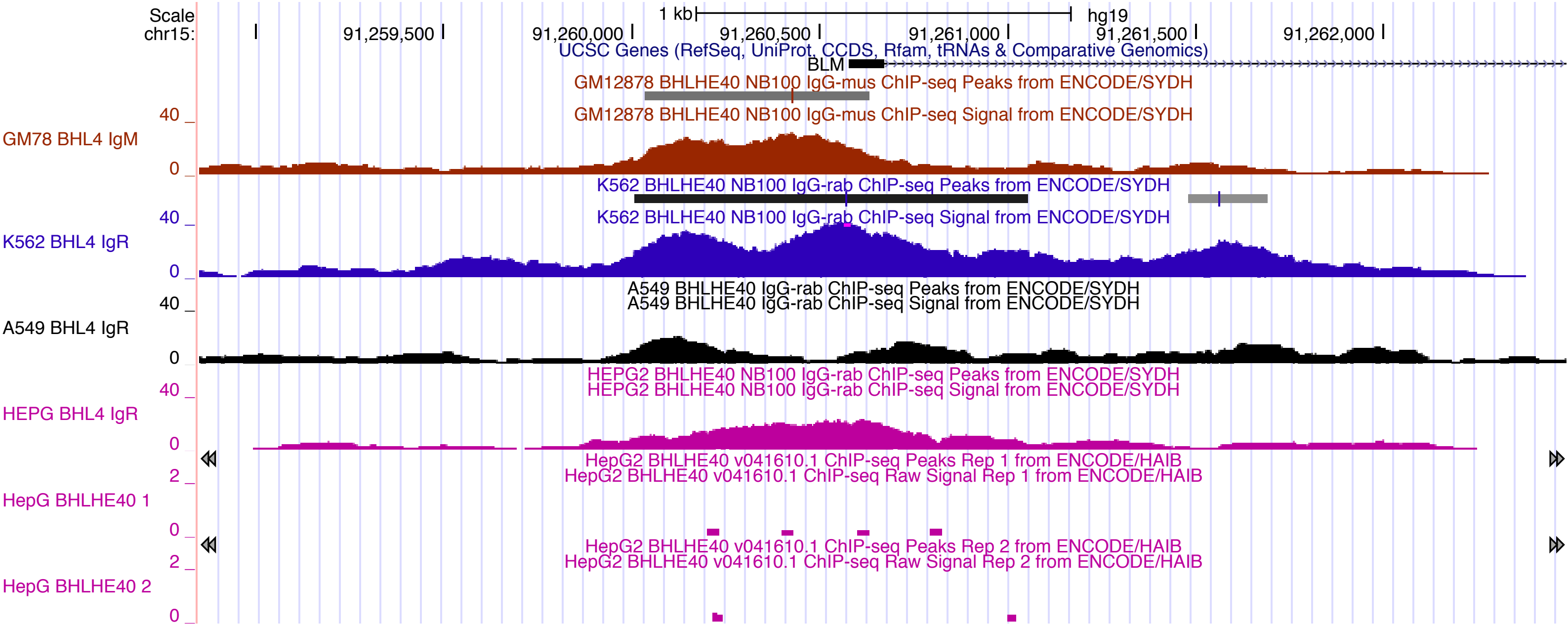
Scale
chr15:
1 kb
hg19
91,259,500
91,260,000
91,260,500
91,261,000
91,261,500
91,262,000
UCSC Genes (RefSeq, UniProt, CCDS, Rfam, tRNAs & Comparative Genomics)
BLM
GM12878 BHLHE40 NB100 IgG-mus ChIP-seq Peaks from ENCODE/SYDH
GM12878 BHLHE40 NB100 IgG-mus ChIP-seq Signal from ENCODE/SYDH
GM78 BHL4 IgM
K562 BHLHE40 NB100 IgG-rab ChIP-seq Peaks from ENCODE/SYDH
K562 BHLHE40 NB100 IgG-rab ChIP-seq Signal from ENCODE/SYDH
K562 BHL4 IgR
A549 BHLHE40 IgG-rab ChIP-seq Peaks from ENCODE/SYDH
A549 BHLHE40 IgG-rab ChIP-seq Signal from ENCODE/SYDH
A549 BHL4 IgR
HEPG2 BHLHE40 NB100 IgG-rab ChIP-seq Peaks from ENCODE/SYDH
HEPG2 BHLHE40 NB100 IgG-rab ChIP-seq Signal from ENCODE/SYDH
HEPG BHL4 IgR
HepG2 BHLHE40 v041610.1 ChIP-seq Peaks Rep 1 from ENCODE/HAIB
HepG2 BHLHE40 v041610.1 ChIP-seq Raw Signal Rep 1 from ENCODE/HAIB
HepG BHLHE40 1
HepG2 BHLHE40 v041610.1 ChIP-seq Peaks Rep 2 from ENCODE/HAIB
HepG2 BHLHE40 v041610.1 ChIP-seq Raw Signal Rep 2 from ENCODE/HAIB
HepG BHLHE40 2

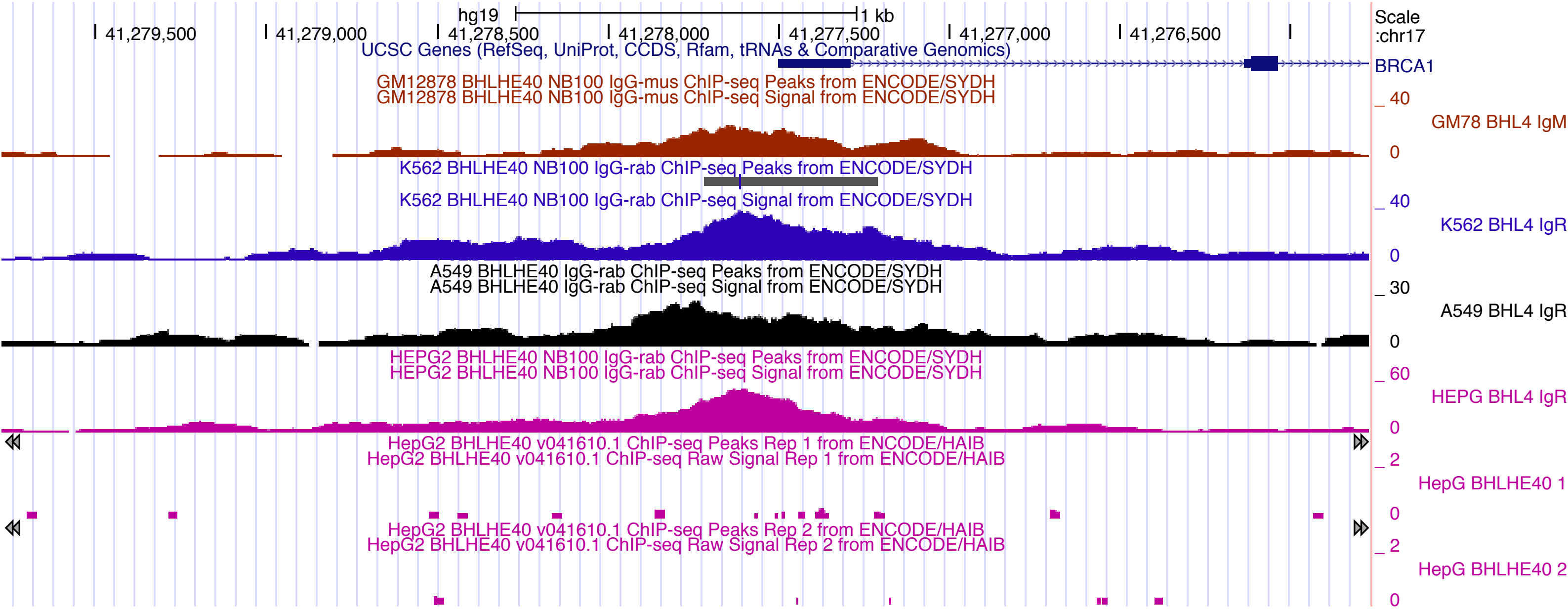

Scale
hg19
1 kb
chr17:
41,279,500
41,279,000
41,278,500
41,278,000
41,277,500
41,277,000
41,276,500
UCSC Genes (RefSeq, UniProt, CCDS, Rfam, tRNAs & Comparative Genomics)
BRCA1
GM12878 BHLHE40 NB100 IgG-mus ChIP-seq Peaks from ENCODE/SYDH
GM12878 BHLHE40 NB100 IgG-mus ChIP-seq Signal from ENCODE/SYDH
GM78 BHL4 IgM
K562 BHLHE40 NB100 IgG-rab ChIP-seq Peaks from ENCODE/SYDH
K562 BHLHE40 NB100 IgG-rab ChIP-seq Signal from ENCODE/SYDH
K562 BHL4 IgR
A549 BHLHE40 IgG-rab ChIP-seq Peaks from ENCODE/SYDH
A549 BHLHE40 IgG-rab ChIP-seq Signal from ENCODE/SYDH
A549 BHL4 IgR
HEPG2 BHLHE40 NB100 IgG-rab ChIP-seq Peaks from ENCODE/SYDH
HEPG2 BHLHE40 NB100 IgG-rab ChIP-seq Signal from ENCODE/SYDH
HEPG BHL4 IgR
HepG2 BHLHE40 v041610.1 ChIP-seq Peaks Rep 1 from ENCODE/HAIB
HepG2 BHLHE40 v041610.1 ChIP-seq Raw Signal Rep 1 from ENCODE/HAIB
HepG BHLHE40 1
HepG2 BHLHE40 v041610.1 ChIP-seq Peaks Rep 2 from ENCODE/HAIB
HepG2 BHLHE40 v041610.1 ChIP-seq Raw Signal Rep 2 from ENCODE/HAIB
HepG BHLHE40 2

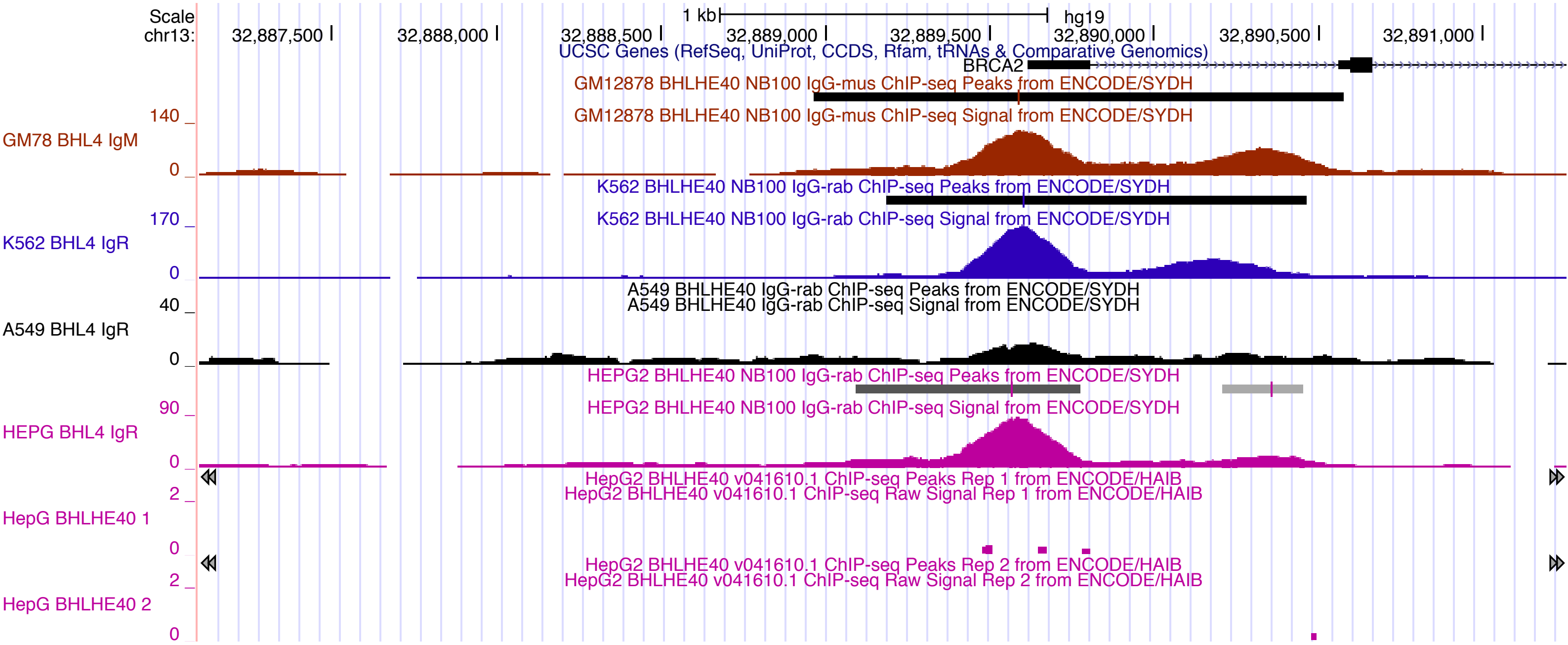

Scale
chr13:
1 kb
hg19
32,887,500
32,888,000
32,888,500
32,889,000
32,889,500
32,890,000
32,890,500
32,891,000
UCSC Genes (RefSeq, UniProt, CCDS, Rfam, tRNAs & Comparative Genomics)
BRCA2
GM12878 BHLHE40 NB100 IgG-mus ChIP-seq Peaks from ENCODE/SYDH
GM12878 BHLHE40 NB100 IgG-mus ChIP-seq Signal from ENCODE/SYDH
GM78 BHL4 IgM
140
0
K562 BHLHE40 NB100 IgG-rab ChIP-seq Peaks from ENCODE/SYDH
K562 BHLHE40 NB100 IgG-rab ChIP-seq Signal from ENCODE/SYDH
K562 BHL4 IgR
170
0
A549 BHLHE40 IgG-rab ChIP-seq Peaks from ENCODE/SYDH
A549 BHLHE40 IgG-rab ChIP-seq Signal from ENCODE/SYDH
A549 BHL4 IgR
40
0
HEPG2 BHLHE40 NB100 IgG-rab ChIP-seq Peaks from ENCODE/SYDH
HEPG2 BHLHE40 NB100 IgG-rab ChIP-seq Signal from ENCODE/SYDH
HEPG BHL4 IgR
90
0
HepG2 BHLHE40 v041610.1 ChIP-seq Peaks Rep 1 from ENCODE/HAIB
HepG2 BHLHE40 v041610.1 ChIP-seq Raw Signal Rep 1 from ENCODE/HAIB
HepG BHLHE40 1
2
0
HepG2 BHLHE40 v041610.1 ChIP-seq Peaks Rep 2 from ENCODE/HAIB
HepG2 BHLHE40 v041610.1 ChIP-seq Raw Signal Rep 2 from ENCODE/HAIB
HepG BHLHE40 2
2
0

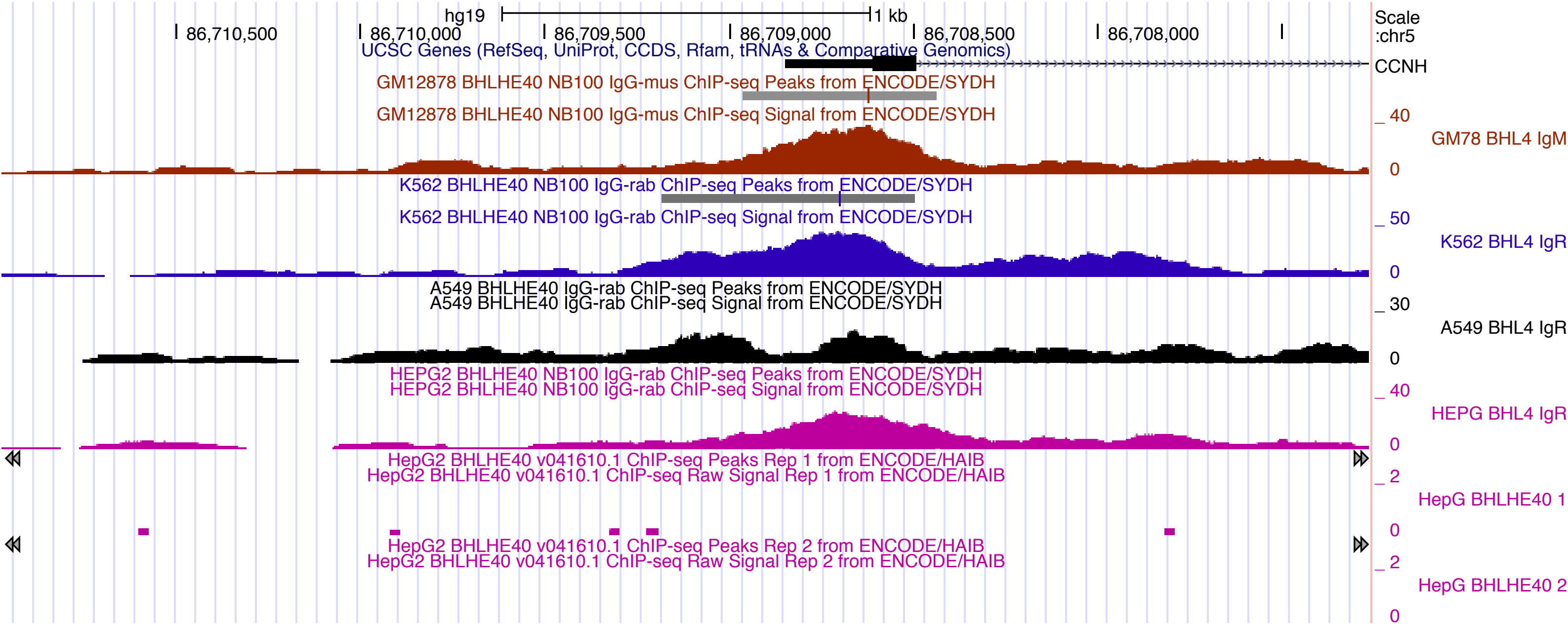

hg19
1 kb
Scale
:chr5
86,710,500
86,710,000
86,709,500
86,709,000
86,708,500
86,708,000
UCSC Genes (RefSeq, UniProt, CCDS, Rfam, tRNAs & Comparative Genomics)
CCNH
GM12878 BHLHE40 NB100 IgG-mus ChIP-seq Peaks from ENCODE/SYDH
GM12878 BHLHE40 NB100 IgG-mus ChIP-seq Signal from ENCODE/SYDH
GM78 BHL4 IgM
K562 BHLHE40 NB100 IgG-rab ChIP-seq Peaks from ENCODE/SYDH
K562 BHLHE40 NB100 IgG-rab ChIP-seq Signal from ENCODE/SYDH
K562 BHL4 IgR
A549 BHLHE40 IgG-rab ChIP-seq Peaks from ENCODE/SYDH
A549 BHLHE40 IgG-rab ChIP-seq Signal from ENCODE/SYDH
A549 BHL4 IgR
HEPG2 BHLHE40 NB100 IgG-rab ChIP-seq Peaks from ENCODE/SYDH
HEPG2 BHLHE40 NB100 IgG-rab ChIP-seq Signal from ENCODE/SYDH
HEPG BHL4 IgR
HepG2 BHLHE40 v041610.1 ChIP-seq Peaks Rep 1 from ENCODE/HAIB
HepG2 BHLHE40 v041610.1 ChIP-seq Raw Signal Rep 1 from ENCODE/HAIB
HepG BHLHE40 1
HepG2 BHLHE40 v041610.1 ChIP-seq Peaks Rep 2 from ENCODE/HAIB
HepG2 BHLHE40 v041610.1 ChIP-seq Raw Signal Rep 2 from ENCODE/HAIB
HepG BHLHE40 2
_ 40
0
_ 50
0
_ 30
0
_ 40
0
_ 2
0
_ 2
0

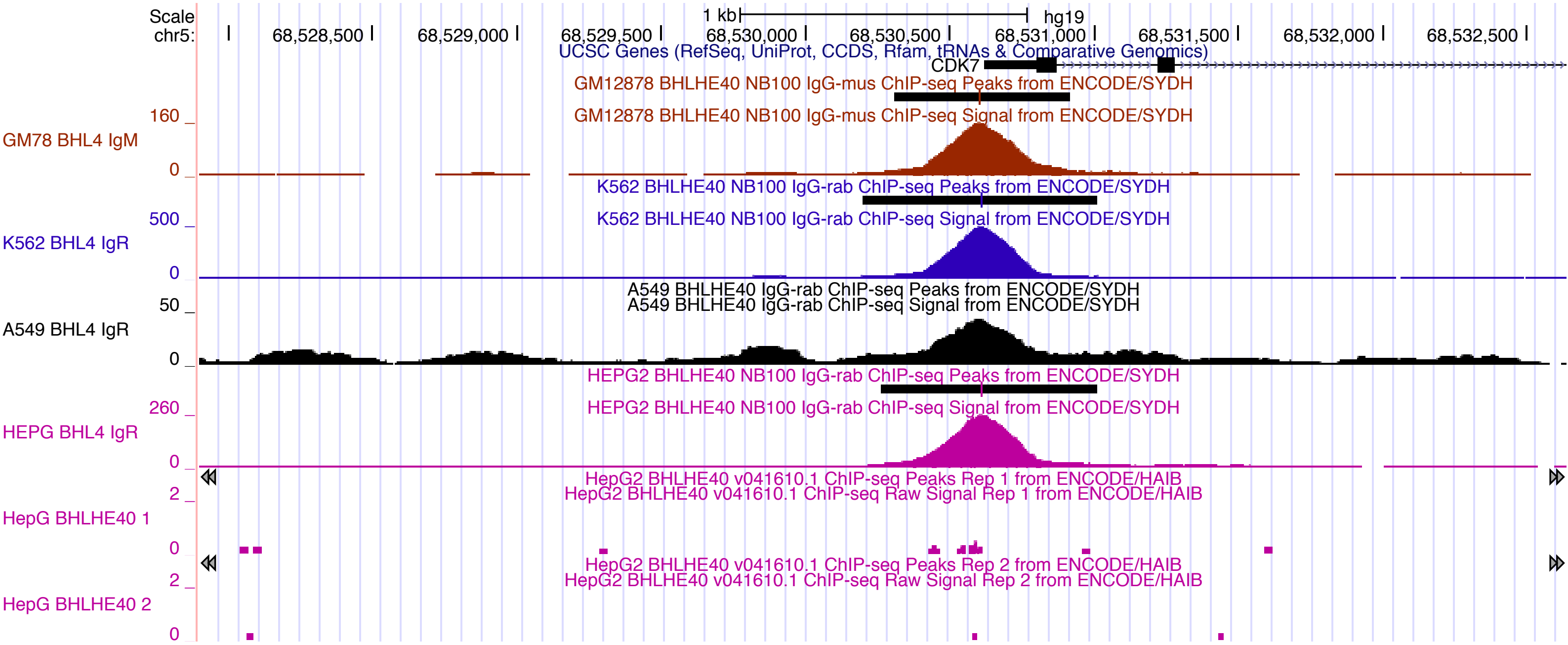

Scale
chr5:
1 kb
hg19
68,528,500
68,529,000
68,529,500
68,530,000
68,530,500
68,531,000
68,531,500
68,532,000
68,532,500
UCSC Genes (RefSeq, UniProt, CCDS, Rfam, tRNAs & Comparative Genomics)
CDK7
GM12878 BHLHE40 NB100 IgG-mus ChIP-seq Peaks from ENCODE/SYDH
GM12878 BHLHE40 NB100 IgG-mus ChIP-seq Signal from ENCODE/SYDH
GM78 BHL4 IgM
160
0
K562 BHLHE40 NB100 IgG-rab ChIP-seq Peaks from ENCODE/SYDH
K562 BHLHE40 NB100 IgG-rab ChIP-seq Signal from ENCODE/SYDH
K562 BHL4 IgR
500
0
A549 BHLHE40 IgG-rab ChIP-seq Peaks from ENCODE/SYDH
A549 BHLHE40 IgG-rab ChIP-seq Signal from ENCODE/SYDH
A549 BHL4 IgR
50
0
HEPG2 BHLHE40 NB100 IgG-rab ChIP-seq Peaks from ENCODE/SYDH
HEPG2 BHLHE40 NB100 IgG-rab ChIP-seq Signal from ENCODE/SYDH
HEPG BHL4 IgR
260
0
HepG2 BHLHE40 v041610.1 ChIP-seq Peaks Rep 1 from ENCODE/HAIB
HepG2 BHLHE40 v041610.1 ChIP-seq Raw Signal Rep 1 from ENCODE/HAIB
HepG BHLHE40 1
2
0
HepG2 BHLHE40 v041610.1 ChIP-seq Peaks Rep 2 from ENCODE/HAIB
HepG2 BHLHE40 v041610.1 ChIP-seq Raw Signal Rep 2 from ENCODE/HAIB
HepG BHLHE40 2
2
0

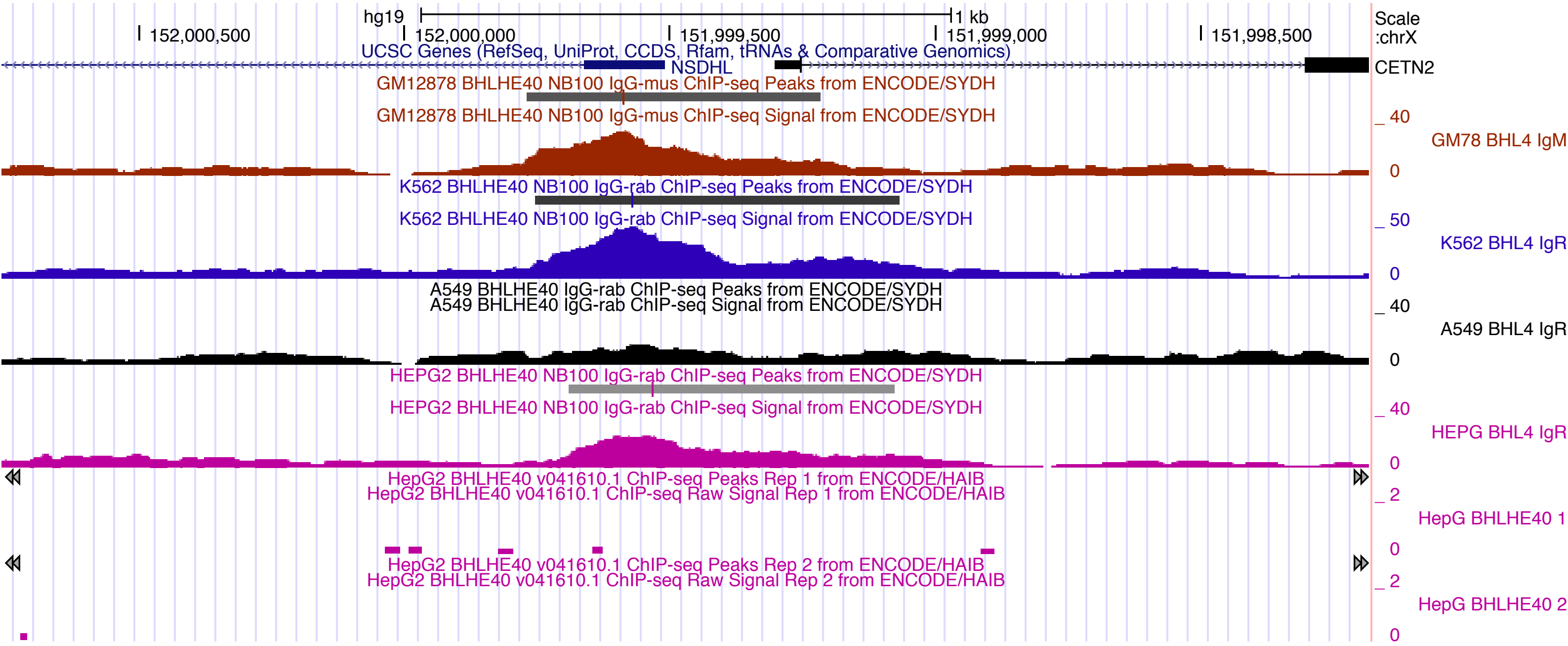
Scale
chrX
hg19
1 kb
152,000,500
152,000,000
151,999,500
151,999,000
151,998,500
UCSC Genes (RefSeq, UniProt, CCDS, Rfam, tRNAs & Comparative Genomics)
NSDHL
CETN2
GM12878 BHLHE40 NB100 IgG-mus ChIP-seq Peaks from ENCODE/SYDH
GM12878 BHLHE40 NB100 IgG-mus ChIP-seq Signal from ENCODE/SYDH
GM78 BHL4 IgM
K562 BHLHE40 NB100 IgG-rab ChIP-seq Peaks from ENCODE/SYDH
K562 BHLHE40 NB100 IgG-rab ChIP-seq Signal from ENCODE/SYDH
K562 BHL4 IgR
A549 BHLHE40 IgG-rab ChIP-seq Peaks from ENCODE/SYDH
A549 BHLHE40 IgG-rab ChIP-seq Signal from ENCODE/SYDH
A549 BHL4 IgR
HEPG2 BHLHE40 NB100 IgG-rab ChIP-seq Peaks from ENCODE/SYDH
HEPG2 BHLHE40 NB100 IgG-rab ChIP-seq Signal from ENCODE/SYDH
HEPG BHL4 IgR
HepG2 BHLHE40 v041610.1 ChIP-seq Peaks Rep 1 from ENCODE/HAIB
HepG2 BHLHE40 v041610.1 ChIP-seq Raw Signal Rep 1 from ENCODE/HAIB
HepG BHLHE40 1
HepG2 BHLHE40 v041610.1 ChIP-seq Peaks Rep 2 from ENCODE/HAIB
HepG2 BHLHE40 v041610.1 ChIP-seq Raw Signal Rep 2 from ENCODE/HAIB
HepG BHLHE40 2

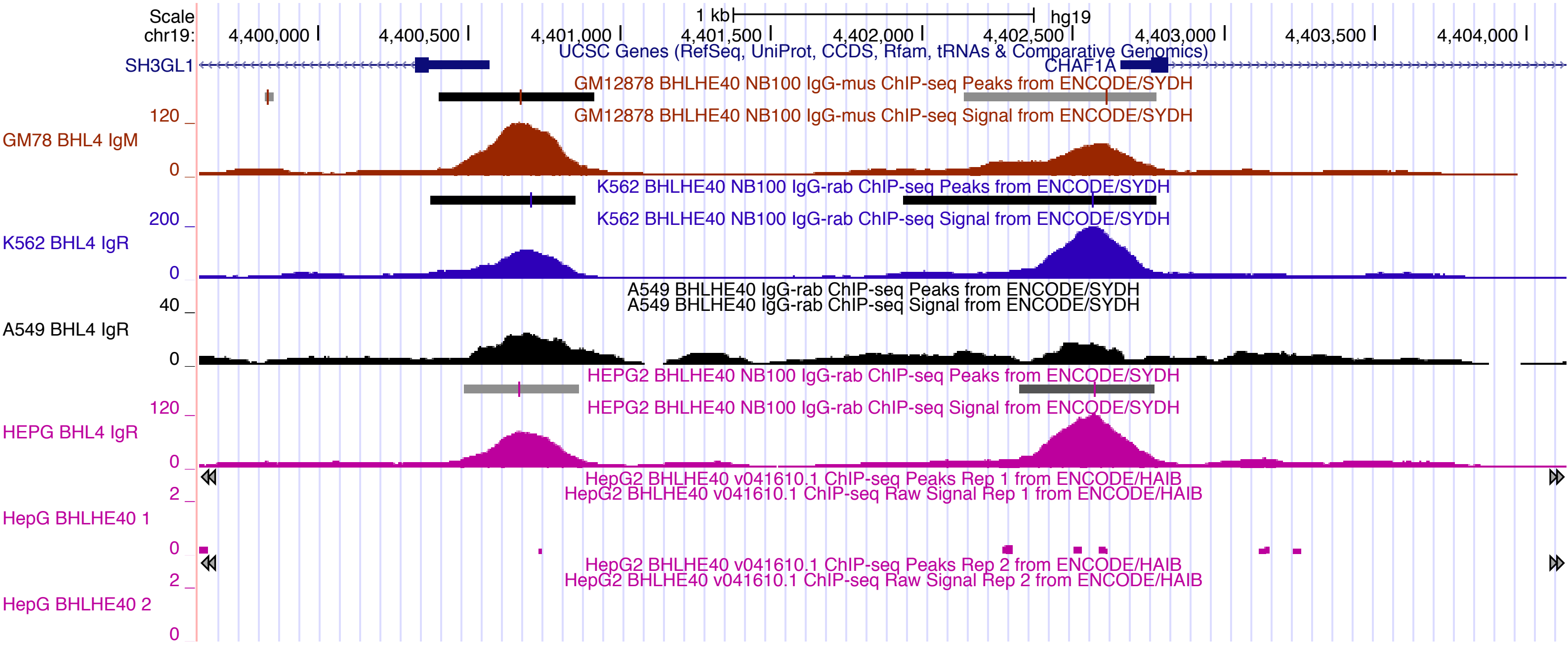
Scale
chr19:
1 kb
hg19
4,400,000
4,400,500
4,401,000
4,401,500
4,402,000
4,402,500
4,403,000
4,403,500
4,404,000
UCSC Genes (RefSeq, UniProt, CCDS, Rfam, tRNAs & Comparative Genomics)
SH3GL1
CHAF1A
GM12878 BHLHE40 NB100 IgG-mus ChIP-seq Peaks from ENCODE/SYDH
GM12878 BHLHE40 NB100 IgG-mus ChIP-seq Signal from ENCODE/SYDH
GM78 BHL4 IgM
120
0
K562 BHLHE40 NB100 IgG-rab ChIP-seq Peaks from ENCODE/SYDH
K562 BHLHE40 NB100 IgG-rab ChIP-seq Signal from ENCODE/SYDH
K562 BHL4 IgR
200
0
A549 BHLHE40 IgG-rab ChIP-seq Peaks from ENCODE/SYDH
A549 BHLHE40 IgG-rab ChIP-seq Signal from ENCODE/SYDH
A549 BHL4 IgR
40
0
HEPG2 BHLHE40 NB100 IgG-rab ChIP-seq Peaks from ENCODE/SYDH
HEPG2 BHLHE40 NB100 IgG-rab ChIP-seq Signal from ENCODE/SYDH
HEPG BHL4 IgR
120
0
HepG2 BHLHE40 v041610.1 ChIP-seq Peaks Rep 1 from ENCODE/HAIB
HepG2 BHLHE40 v041610.1 ChIP-seq Raw Signal Rep 1 from ENCODE/HAIB
HepG BHLHE40 1
2
0
HepG2 BHLHE40 v041610.1 ChIP-seq Peaks Rep 2 from ENCODE/HAIB
HepG2 BHLHE40 v041610.1 ChIP-seq Raw Signal Rep 2 from ENCODE/HAIB
HepG BHLHE40 2
2
0

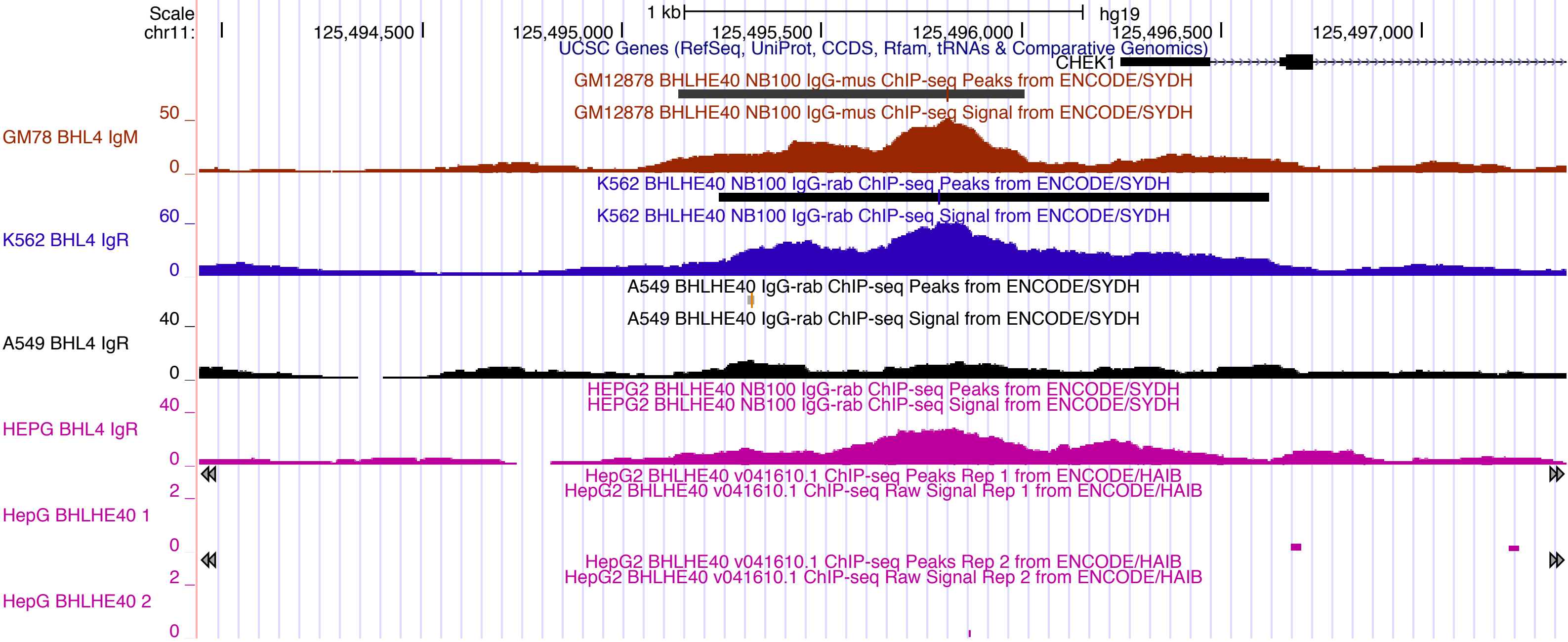

Scale
chr11:
1 kb
hg19
125,494,500
125,495,000
125,495,500
125,496,000
125,496,500
125,497,000
UCSC Genes (RefSeq, UniProt, CCDS, Rfam, tRNAs & Comparative Genomics)
CHEK1
GM12878 BHLHE40 NB100 IgG-mus ChIP-seq Peaks from ENCODE/SYDH
GM12878 BHLHE40 NB100 IgG-mus ChIP-seq Signal from ENCODE/SYDH
50 _
GM78 BHL4 IgM
0 _
K562 BHLHE40 NB100 IgG-rab ChIP-seq Peaks from ENCODE/SYDH
K562 BHLHE40 NB100 IgG-rab ChIP-seq Signal from ENCODE/SYDH
60 _
K562 BHL4 IgR
0
A549 BHLHE40 IgG-rab ChIP-seq Peaks from ENCODE/SYDH
A549 BHLHE40 IgG-rab ChIP-seq Signal from ENCODE/SYDH
40 _
A549 BHL4 IgR
0 _
HEPG2 BHLHE40 NB100 IgG-rab ChIP-seq Peaks from ENCODE/SYDH
HEPG2 BHLHE40 NB100 IgG-rab ChIP-seq Signal from ENCODE/SYDH
40 _
HEPG BHL4 IgR
0 _
HepG2 BHLHE40 v041610.1 ChIP-seq Peaks Rep 1 from ENCODE/HAIB
HepG2 BHLHE40 v041610.1 ChIP-seq Raw Signal Rep 1 from ENCODE/HAIB
2 _
HepG BHLHE40 1
0
HepG2 BHLHE40 v041610.1 ChIP-seq Peaks Rep 2 from ENCODE/HAIB
HepG2 BHLHE40 v041610.1 ChIP-seq Raw Signal Rep 2 from ENCODE/HAIB
2 _
HepG BHLHE40 2
0

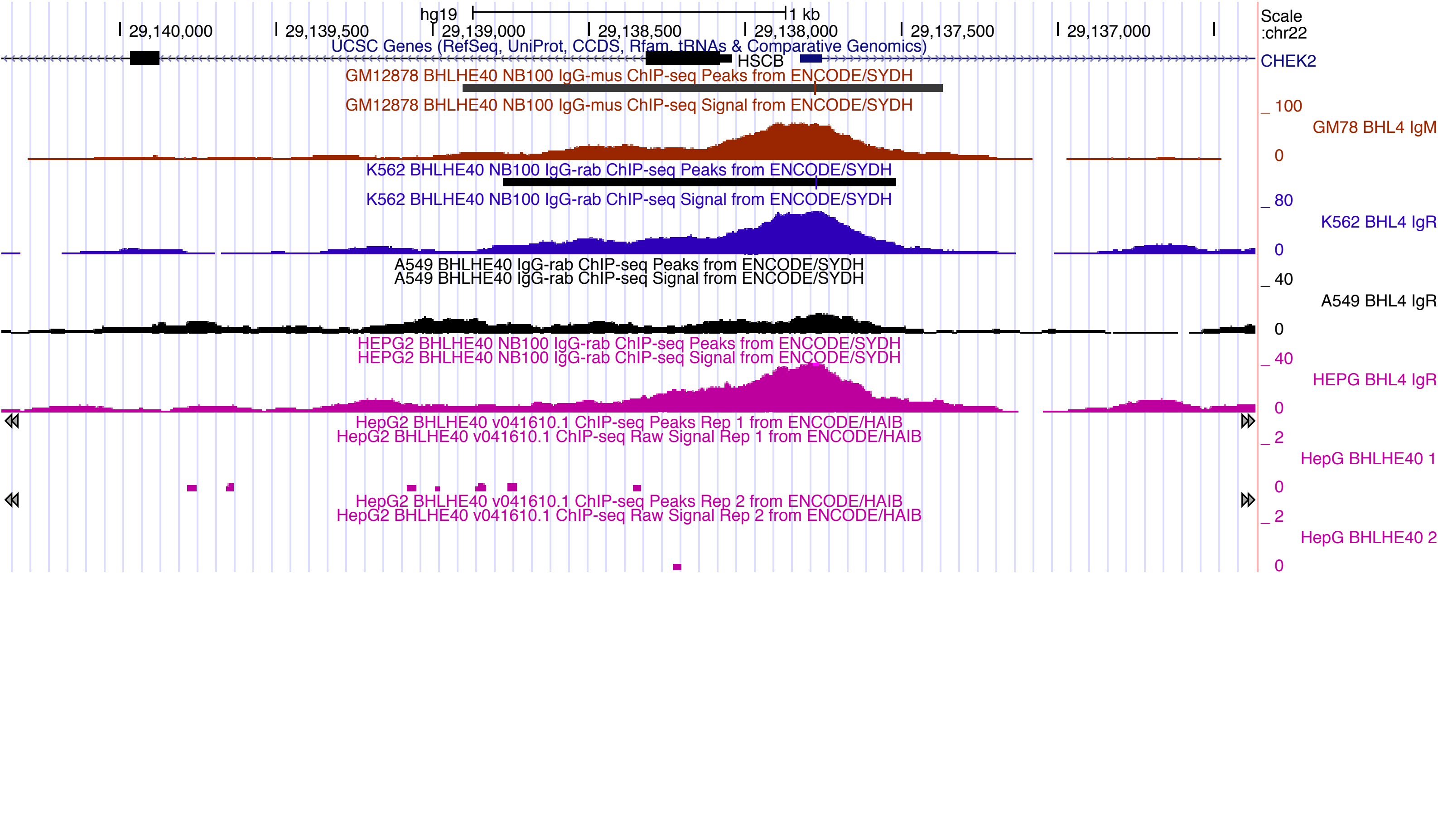

hg19
1 kb
Scale
chr22
29,140,000
29,139,500
29,139,000
29,138,500
29,138,000
29,137,500
29,137,000
UCSC Genes (RefSeq, UniProt, CCDS, Rfam, tRNAs & Comparative Genomics)
HSCB
CHEK2
GM12878 BHLHE40 NB100 IgG-mus ChIP-seq Peaks from ENCODE/SYDH
GM12878 BHLHE40 NB100 IgG-mus ChIP-seq Signal from ENCODE/SYDH
GM78 BHL4 IgM
K562 BHLHE40 NB100 IgG-rab ChIP-seq Peaks from ENCODE/SYDH
K562 BHLHE40 NB100 IgG-rab ChIP-seq Signal from ENCODE/SYDH
K562 BHL4 IgR
A549 BHLHE40 IgG-rab ChIP-seq Peaks from ENCODE/SYDH
A549 BHLHE40 IgG-rab ChIP-seq Signal from ENCODE/SYDH
A549 BHL4 IgR
HEPG2 BHLHE40 NB100 IgG-rab ChIP-seq Peaks from ENCODE/SYDH
HEPG2 BHLHE40 NB100 IgG-rab ChIP-seq Signal from ENCODE/SYDH
HEPG BHL4 IgR
HepG2 BHLHE40 v041610.1 ChIP-seq Peaks Rep 1 from ENCODE/HAIB
HepG2 BHLHE40 v041610.1 ChIP-seq Raw Signal Rep 1 from ENCODE/HAIB
HepG BHLHE40 1
HepG2 BHLHE40 v041610.1 ChIP-seq Peaks Rep 2 from ENCODE/HAIB
HepG2 BHLHE40 v041610.1 ChIP-seq Raw Signal Rep 2 from ENCODE/HAIB
HepG BHLHE40 2

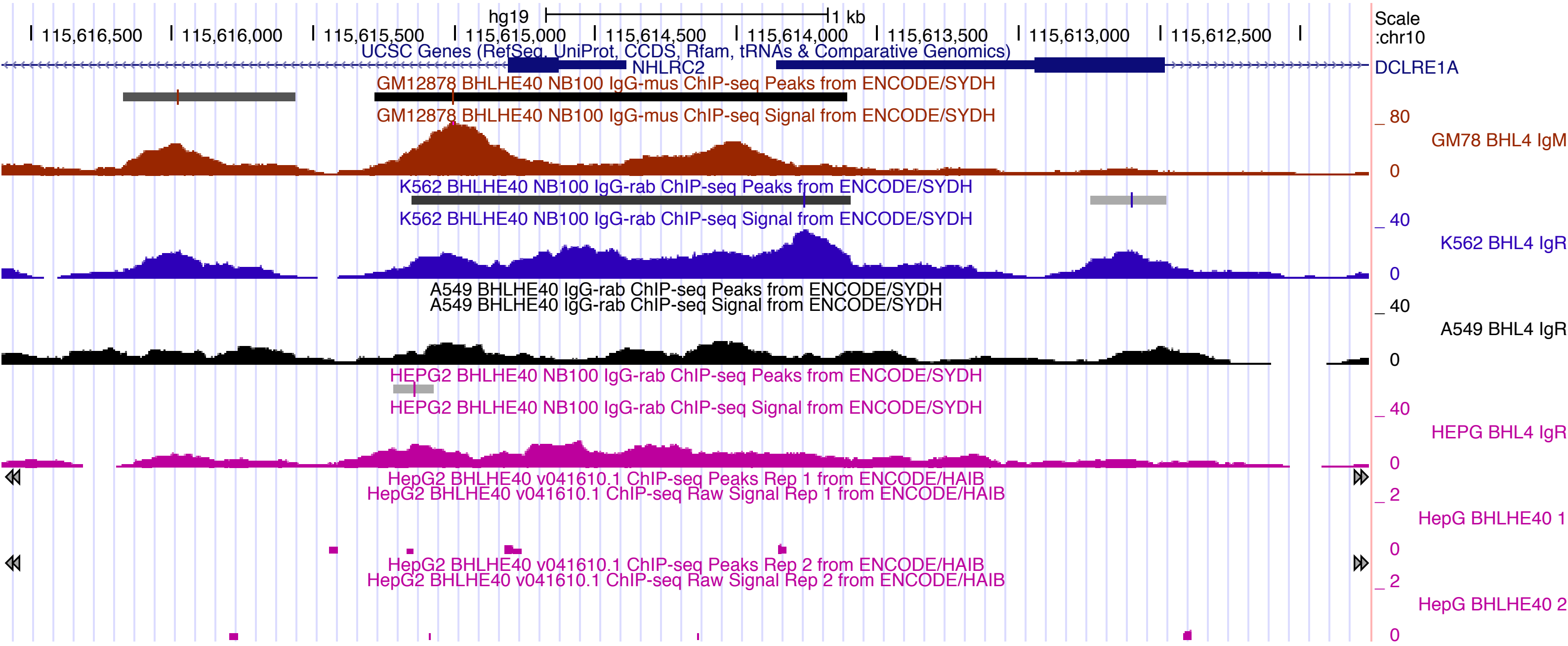
hg19
1 kb
Scale
chr10
115,616,500
115,616,000
115,615,500
115,615,000
115,614,500
115,614,000
115,613,500
115,613,000
115,612,500
UCSC Genes (RefSeq, UniProt, CCDS, Rfam, tRNAs & Comparative Genomics)
NHLRC2
DCLRE1A
GM12878 BHLHE40 NB100 IgG-mus ChIP-seq Peaks from ENCODE/SYDH
GM12878 BHLHE40 NB100 IgG-mus ChIP-seq Signal from ENCODE/SYDH
GM78 BHL4 IgM
K562 BHLHE40 NB100 IgG-rab ChIP-seq Peaks from ENCODE/SYDH
K562 BHLHE40 NB100 IgG-rab ChIP-seq Signal from ENCODE/SYDH
K562 BHL4 IgR
A549 BHLHE40 IgG-rab ChIP-seq Peaks from ENCODE/SYDH
A549 BHLHE40 IgG-rab ChIP-seq Signal from ENCODE/SYDH
A549 BHL4 IgR
HEPG2 BHLHE40 NB100 IgG-rab ChIP-seq Peaks from ENCODE/SYDH
HEPG2 BHLHE40 NB100 IgG-rab ChIP-seq Signal from ENCODE/SYDH
HEPG BHL4 IgR
HepG2 BHLHE40 v041610.1 ChIP-seq Peaks Rep 1 from ENCODE/HAIB
HepG2 BHLHE40 v041610.1 ChIP-seq Raw Signal Rep 1 from ENCODE/HAIB
HepG BHLHE40 1
HepG2 BHLHE40 v041610.1 ChIP-seq Peaks Rep 2 from ENCODE/HAIB
HepG2 BHLHE40 v041610.1 ChIP-seq Raw Signal Rep 2 from ENCODE/HAIB
HepG BHLHE40 2

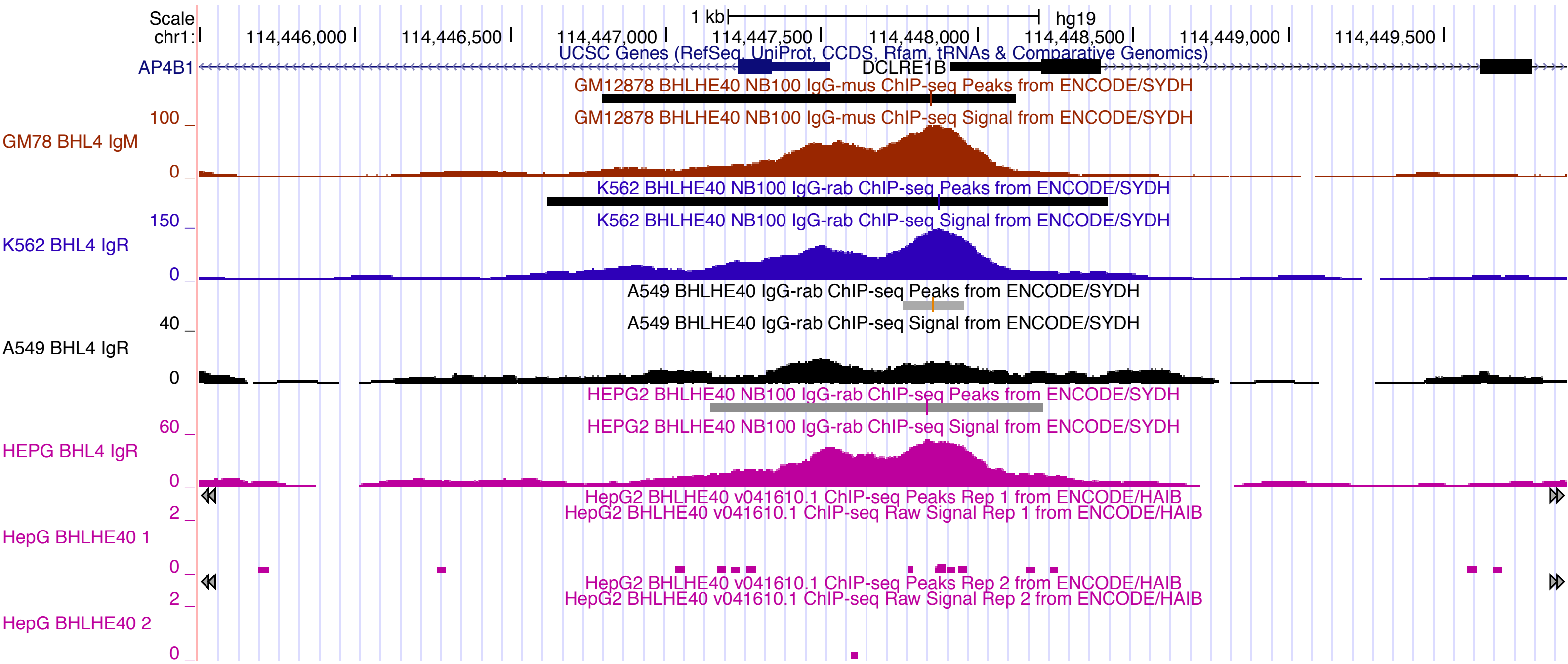

Scale
chr1:
1 kb
hg19
114,446,000
114,446,500
114,447,000
114,447,500
114,448,000
114,448,500
114,449,000
114,449,500
UCSC Genes (RefSeq, UniProt, CCDS, Rfam, tRNAs & Comparative Genomics)
AP4B1
DCLRE1B
GM12878 BHLHE40 NB100 IgG-mus ChIP-seq Peaks from ENCODE/SYDH
GM12878 BHLHE40 NB100 IgG-mus ChIP-seq Signal from ENCODE/SYDH
GM78 BHL4 IgM
100
0
K562 BHLHE40 NB100 IgG-rab ChIP-seq Peaks from ENCODE/SYDH
K562 BHLHE40 NB100 IgG-rab ChIP-seq Signal from ENCODE/SYDH
K562 BHL4 IgR
150
0
A549 BHLHE40 IgG-rab ChIP-seq Peaks from ENCODE/SYDH
A549 BHLHE40 IgG-rab ChIP-seq Signal from ENCODE/SYDH
A549 BHL4 IgR
40
0
HEPG2 BHLHE40 NB100 IgG-rab ChIP-seq Peaks from ENCODE/SYDH
HEPG2 BHLHE40 NB100 IgG-rab ChIP-seq Signal from ENCODE/SYDH
HEPG BHL4 IgR
60
0
HepG2 BHLHE40 v041610.1 ChIP-seq Peaks Rep 1 from ENCODE/HAIB
HepG2 BHLHE40 v041610.1 ChIP-seq Raw Signal Rep 1 from ENCODE/HAIB
HepG BHLHE40 1
2
0
HepG2 BHLHE40 v041610.1 ChIP-seq Peaks Rep 2 from ENCODE/HAIB
HepG2 BHLHE40 v041610.1 ChIP-seq Raw Signal Rep 2 from ENCODE/HAIB
HepG BHLHE40 2
2
0

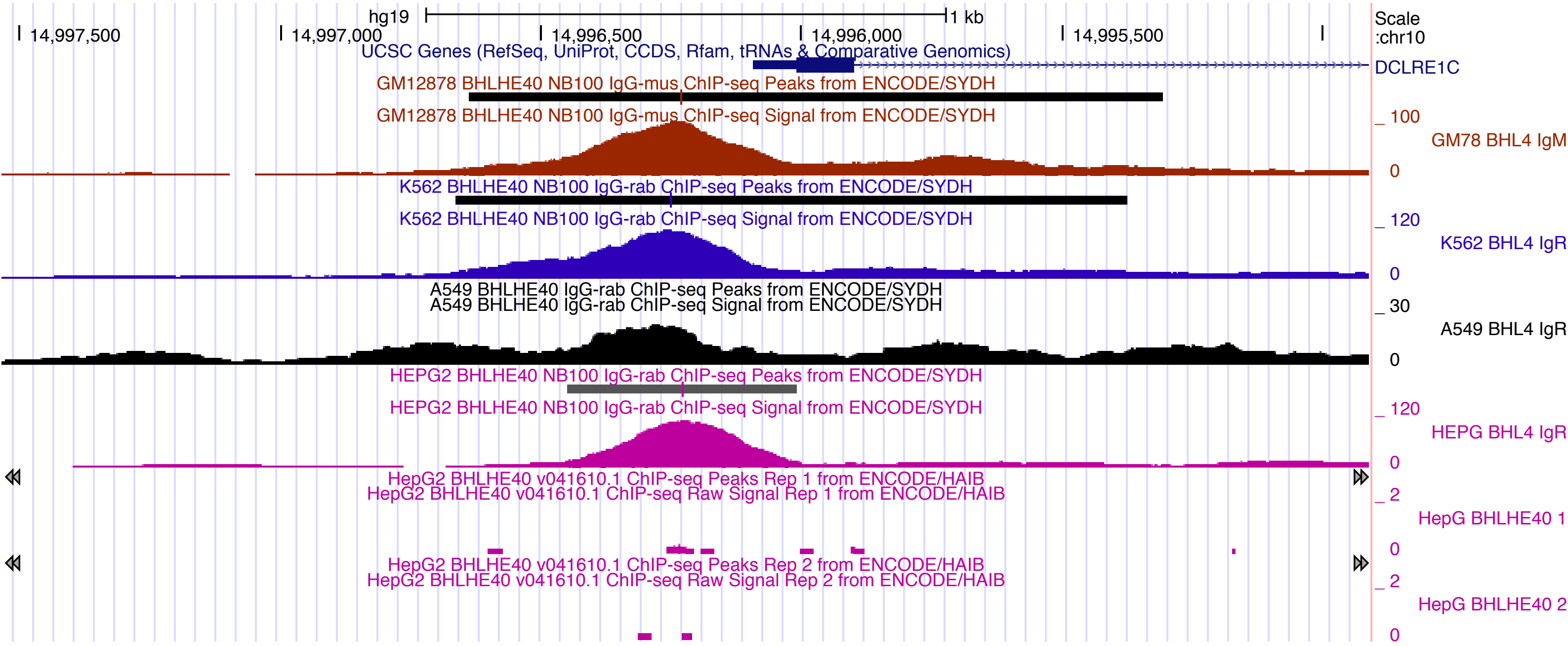

hg19
1 kb
Scale
:chr10
14,997,500
14,997,000
14,996,500
14,996,000
14,995,500
UCSC Genes (RefSeq, UniProt, CCDS, Rfam, tRNAs & Comparative Genomics)
DCLRE1C
GM12878 BHLHE40 NB100 IgG-mus ChIP-seq Peaks from ENCODE/SYDH
GM12878 BHLHE40 NB100 IgG-mus ChIP-seq Signal from ENCODE/SYDH
_ 100
GM78 BHL4 IgM
0
K562 BHLHE40 NB100 IgG-rab ChIP-seq Peaks from ENCODE/SYDH
K562 BHLHE40 NB100 IgG-rab ChIP-seq Signal from ENCODE/SYDH
_ 120
K562 BHL4 IgR
0
A549 BHLHE40 IgG-rab ChIP-seq Peaks from ENCODE/SYDH
A549 BHLHE40 IgG-rab ChIP-seq Signal from ENCODE/SYDH
_ 30
A549 BHL4 IgR
0
HEPG2 BHLHE40 NB100 IgG-rab ChIP-seq Peaks from ENCODE/SYDH
HEPG2 BHLHE40 NB100 IgG-rab ChIP-seq Signal from ENCODE/SYDH
_ 120
HEPG BHL4 IgR
0
HepG2 BHLHE40 v041610.1 ChIP-seq Peaks Rep 1 from ENCODE/HAIB
HepG2 BHLHE40 v041610.1 ChIP-seq Raw Signal Rep 1 from ENCODE/HAIB
_ 2
HepG BHLHE40 1
0
HepG2 BHLHE40 v041610.1 ChIP-seq Peaks Rep 2 from ENCODE/HAIB
HepG2 BHLHE40 v041610.1 ChIP-seq Raw Signal Rep 2 from ENCODE/HAIB
_ 2
HepG BHLHE40 2
0

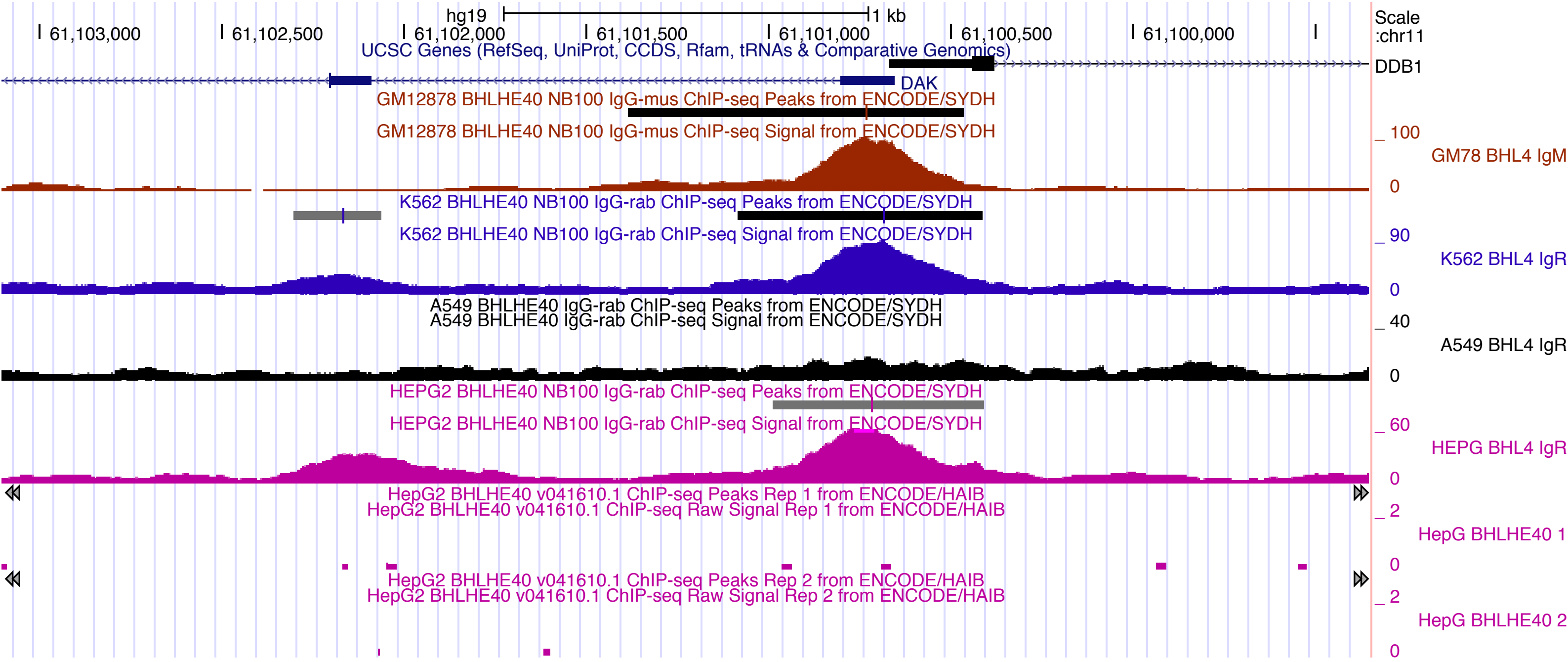
Scale
hg19
1 kb
chr11:
61,103,000
61,102,500
61,102,000
61,101,500
61,101,000
61,100,500
61,100,000
UCSC Genes (RefSeq, UniProt, CCDS, Rfam, tRNAs & Comparative Genomics)
DDB1
DAK
GM12878 BHLHE40 NB100 IgG-mus ChIP-seq Peaks from ENCODE/SYDH
GM12878 BHLHE40 NB100 IgG-mus ChIP-seq Signal from ENCODE/SYDH
100
GM78 BHL4 IgM
0
K562 BHLHE40 NB100 IgG-rab ChIP-seq Peaks from ENCODE/SYDH
K562 BHLHE40 NB100 IgG-rab ChIP-seq Signal from ENCODE/SYDH
90
K562 BHL4 IgR
0
A549 BHLHE40 IgG-rab ChIP-seq Peaks from ENCODE/SYDH
A549 BHLHE40 IgG-rab ChIP-seq Signal from ENCODE/SYDH
40
A549 BHL4 IgR
0
HEPG2 BHLHE40 NB100 IgG-rab ChIP-seq Peaks from ENCODE/SYDH
HEPG2 BHLHE40 NB100 IgG-rab ChIP-seq Signal from ENCODE/SYDH
60
HEPG BHL4 IgR
0
HepG2 BHLHE40 v041610.1 ChIP-seq Peaks Rep 1 from ENCODE/HAIB
HepG2 BHLHE40 v041610.1 ChIP-seq Raw Signal Rep 1 from ENCODE/HAIB
2
HepG BHLHE40 1
0
HepG2 BHLHE40 v041610.1 ChIP-seq Peaks Rep 2 from ENCODE/HAIB
HepG2 BHLHE40 v041610.1 ChIP-seq Raw Signal Rep 2 from ENCODE/HAIB
2
HepG BHLHE40 2
0

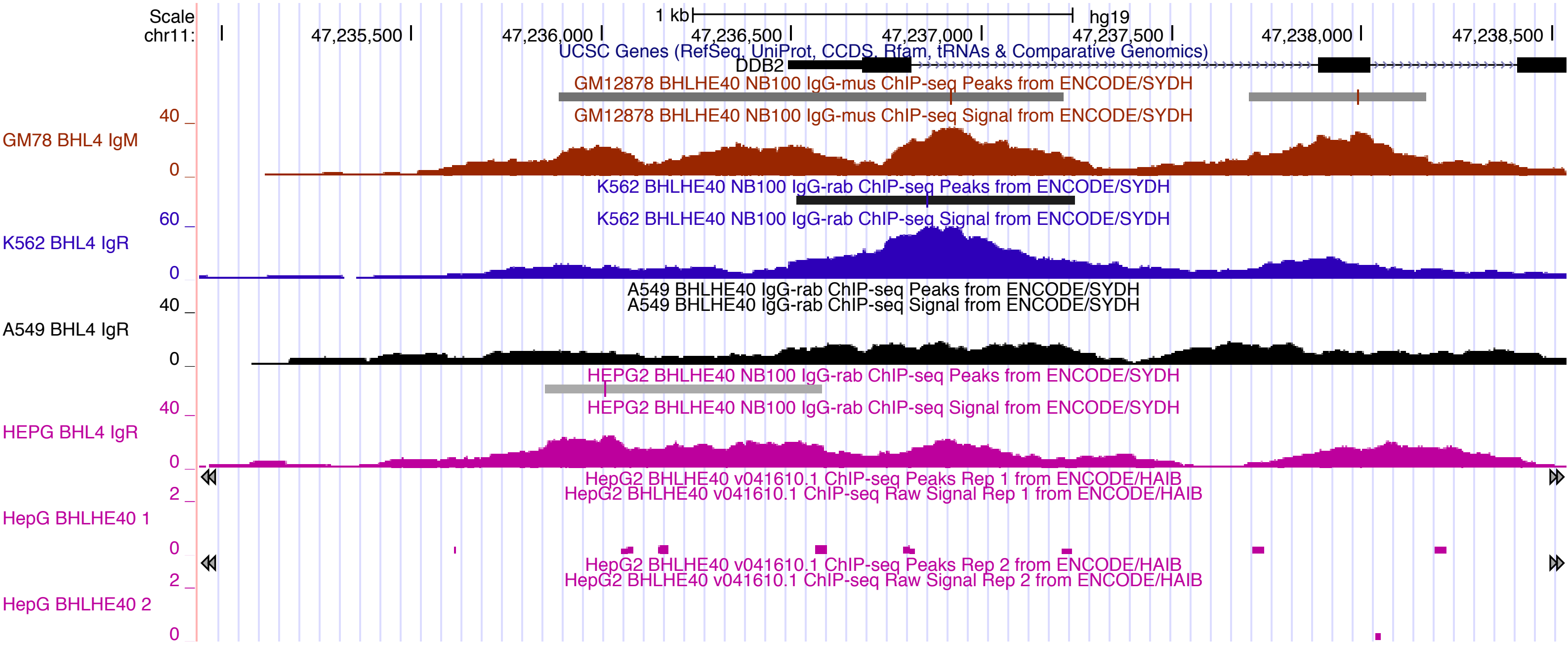
Scale
chr11:
1 kb
hg19
47,235,500
47,236,000
47,236,500
47,237,000
47,237,500
47,238,000
47,238,500
UCSC Genes (RefSeq, UniProt, CCDS, Rfam, tRNAs & Comparative Genomics)
DDB2
GM12878 BHLHE40 NB100 IgG-mus ChIP-seq Peaks from ENCODE/SYDH
GM12878 BHLHE40 NB100 IgG-mus ChIP-seq Signal from ENCODE/SYDH
GM78 BHL4 IgM
K562 BHLHE40 NB100 IgG-rab ChIP-seq Peaks from ENCODE/SYDH
K562 BHLHE40 NB100 IgG-rab ChIP-seq Signal from ENCODE/SYDH
K562 BHL4 IgR
A549 BHLHE40 IgG-rab ChIP-seq Peaks from ENCODE/SYDH
A549 BHLHE40 IgG-rab ChIP-seq Signal from ENCODE/SYDH
A549 BHL4 IgR
HEPG2 BHLHE40 NB100 IgG-rab ChIP-seq Peaks from ENCODE/SYDH
HEPG2 BHLHE40 NB100 IgG-rab ChIP-seq Signal from ENCODE/SYDH
HEPG BHL4 IgR
HepG2 BHLHE40 v041610.1 ChIP-seq Peaks Rep 1 from ENCODE/HAIB
HepG2 BHLHE40 v041610.1 ChIP-seq Raw Signal Rep 1 from ENCODE/HAIB
HepG BHLHE40 1
HepG2 BHLHE40 v041610.1 ChIP-seq Peaks Rep 2 from ENCODE/HAIB
HepG2 BHLHE40 v041610.1 ChIP-seq Raw Signal Rep 2 from ENCODE/HAIB
HepG BHLHE40 2

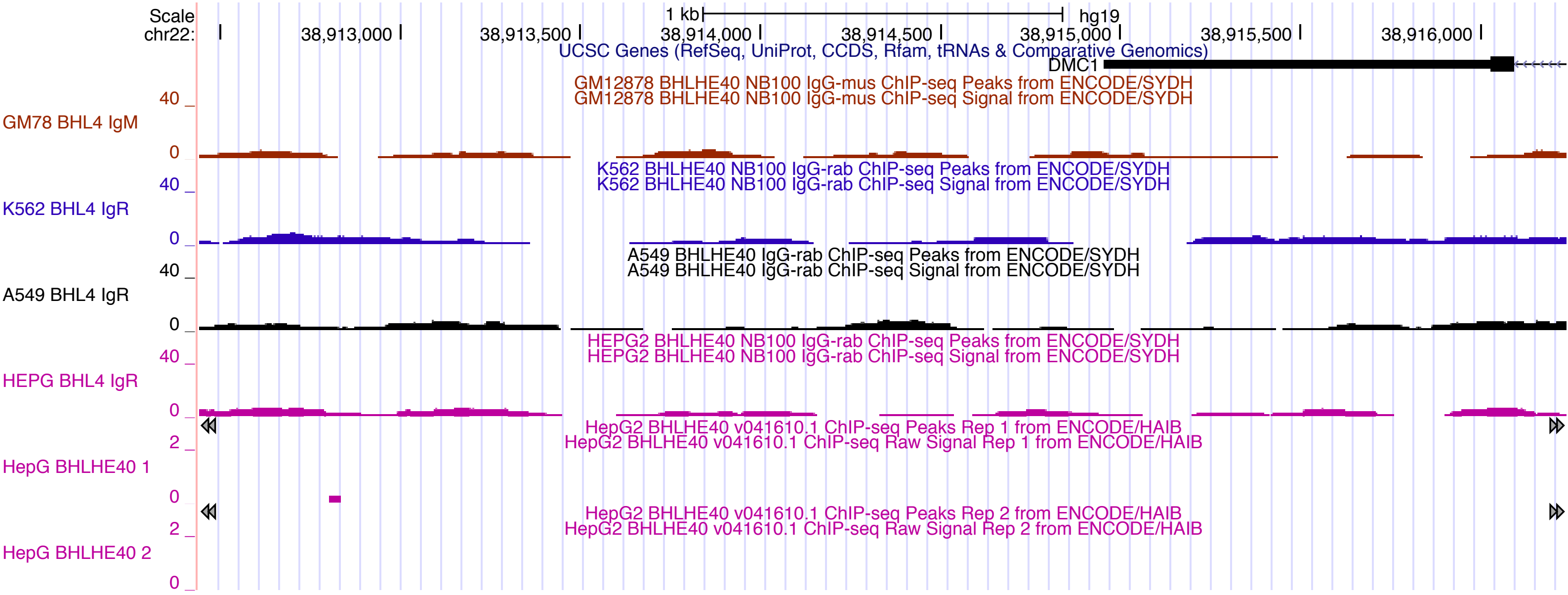

Scale
chr22:
1 kb
hg19
38,913,000
38,913,500
38,914,000
38,914,500
38,915,000
38,915,500
38,916,000
UCSC Genes (RefSeq, UniProt, CCDS, Rfam, tRNAs & Comparative Genomics)
DMC1
GM12878 BHLHE40 NB100 IgG-mus ChIP-seq Peaks from ENCODE/SYDH
GM12878 BHLHE40 NB100 IgG-mus ChIP-seq Signal from ENCODE/SYDH
GM78 BHL4 IgM
K562 BHLHE40 NB100 IgG-rab ChIP-seq Peaks from ENCODE/SYDH
K562 BHLHE40 NB100 IgG-rab ChIP-seq Signal from ENCODE/SYDH
K562 BHL4 IgR
A549 BHLHE40 IgG-rab ChIP-seq Peaks from ENCODE/SYDH
A549 BHLHE40 IgG-rab ChIP-seq Signal from ENCODE/SYDH
A549 BHL4 IgR
HEPG2 BHLHE40 NB100 IgG-rab ChIP-seq Peaks from ENCODE/SYDH
HEPG2 BHLHE40 NB100 IgG-rab ChIP-seq Signal from ENCODE/SYDH
HEPG BHL4 IgR
HepG2 BHLHE40 v041610.1 ChIP-seq Peaks Rep 1 from ENCODE/HAIB
HepG2 BHLHE40 v041610.1 ChIP-seq Raw Signal Rep 1 from ENCODE/HAIB
HepG BHLHE40 1
HepG2 BHLHE40 v041610.1 ChIP-seq Peaks Rep 2 from ENCODE/HAIB
HepG2 BHLHE40 v041610.1 ChIP-seq Raw Signal Rep 2 from ENCODE/HAIB
HepG BHLHE40 2

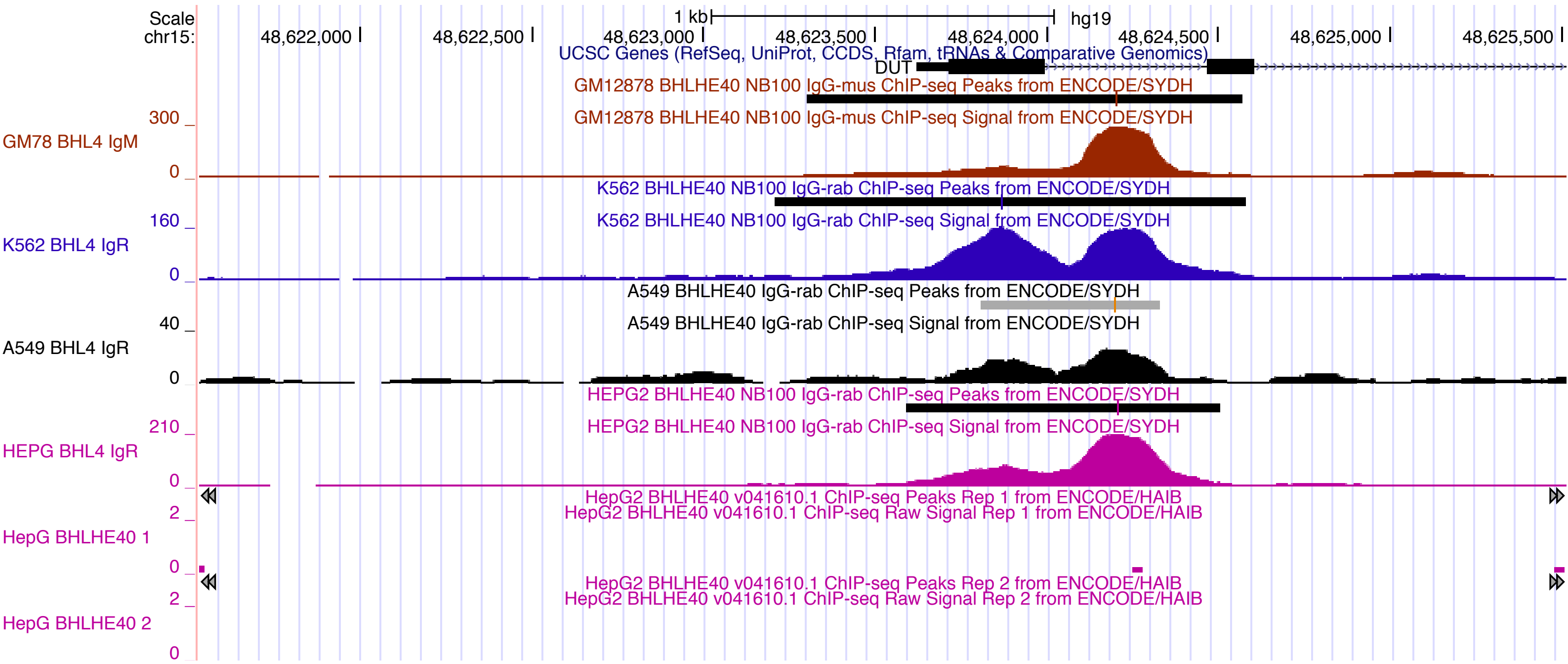

Scale
chr15:
1 kb
hg19
48,622,000
48,622,500
48,623,000
48,623,500
48,624,000
48,624,500
48,625,000
48,625,500
UCSC Genes (RefSeq, UniProt, CCDS, Rfam, tRNAs & Comparative Genomics)
DUT
GM12878 BHLHE40 NB100 IgG-mus ChIP-seq Peaks from ENCODE/SYDH
GM12878 BHLHE40 NB100 IgG-mus ChIP-seq Signal from ENCODE/SYDH
300
GM78 BHL4 IgM
0
K562 BHLHE40 NB100 IgG-rab ChIP-seq Peaks from ENCODE/SYDH
K562 BHLHE40 NB100 IgG-rab ChIP-seq Signal from ENCODE/SYDH
160
K562 BHL4 IgR
0
A549 BHLHE40 IgG-rab ChIP-seq Peaks from ENCODE/SYDH
A549 BHLHE40 IgG-rab ChIP-seq Signal from ENCODE/SYDH
40
A549 BHL4 IgR
0
HEPG2 BHLHE40 NB100 IgG-rab ChIP-seq Peaks from ENCODE/SYDH
HEPG2 BHLHE40 NB100 IgG-rab ChIP-seq Signal from ENCODE/SYDH
210
HEPG BHL4 IgR
0
HepG2 BHLHE40 v041610.1 ChIP-seq Peaks Rep 1 from ENCODE/HAIB
HepG2 BHLHE40 v041610.1 ChIP-seq Raw Signal Rep 1 from ENCODE/HAIB
2
HepG BHLHE40 1
0
HepG2 BHLHE40 v041610.1 ChIP-seq Peaks Rep 2 from ENCODE/HAIB
HepG2 BHLHE40 v041610.1 ChIP-seq Raw Signal Rep 2 from ENCODE/HAIB
2
HepG BHLHE40 2
0

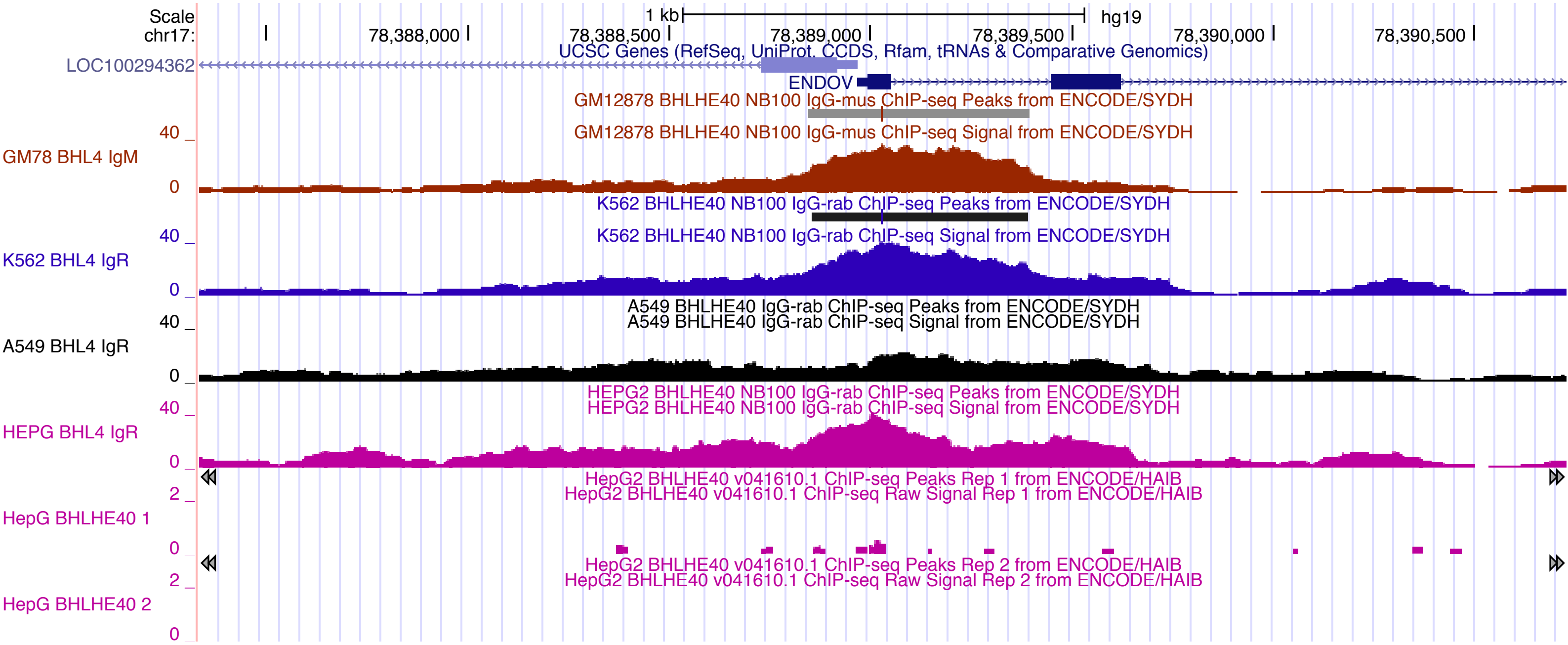
Scale
chr17:
1 kb
hg19
78,388,000
78,388,500
78,389,000
78,389,500
78,390,000
78,390,500
UCSC Genes (RefSeq, UniProt, CCDS, Rfam, tRNAs & Comparative Genomics)
LOC100294362
ENDOV
GM12878 BHLHE40 NB100 IgG-mus ChIP-seq Peaks from ENCODE/SYDH
GM12878 BHLHE40 NB100 IgG-mus ChIP-seq Signal from ENCODE/SYDH
GM78 BHL4 IgM
K562 BHLHE40 NB100 IgG-rab ChIP-seq Peaks from ENCODE/SYDH
K562 BHLHE40 NB100 IgG-rab ChIP-seq Signal from ENCODE/SYDH
K562 BHL4 IgR
A549 BHLHE40 IgG-rab ChIP-seq Peaks from ENCODE/SYDH
A549 BHLHE40 IgG-rab ChIP-seq Signal from ENCODE/SYDH
A549 BHL4 IgR
HEPG2 BHLHE40 NB100 IgG-rab ChIP-seq Peaks from ENCODE/SYDH
HEPG2 BHLHE40 NB100 IgG-rab ChIP-seq Signal from ENCODE/SYDH
HEPG BHL4 IgR
HepG2 BHLHE40 v041610.1 ChIP-seq Peaks Rep 1 from ENCODE/HAIB
HepG2 BHLHE40 v041610.1 ChIP-seq Raw Signal Rep 1 from ENCODE/HAIB
HepG BHLHE40 1
HepG2 BHLHE40 v041610.1 ChIP-seq Peaks Rep 2 from ENCODE/HAIB
HepG2 BHLHE40 v041610.1 ChIP-seq Raw Signal Rep 2 from ENCODE/HAIB
HepG BHLHE40 2

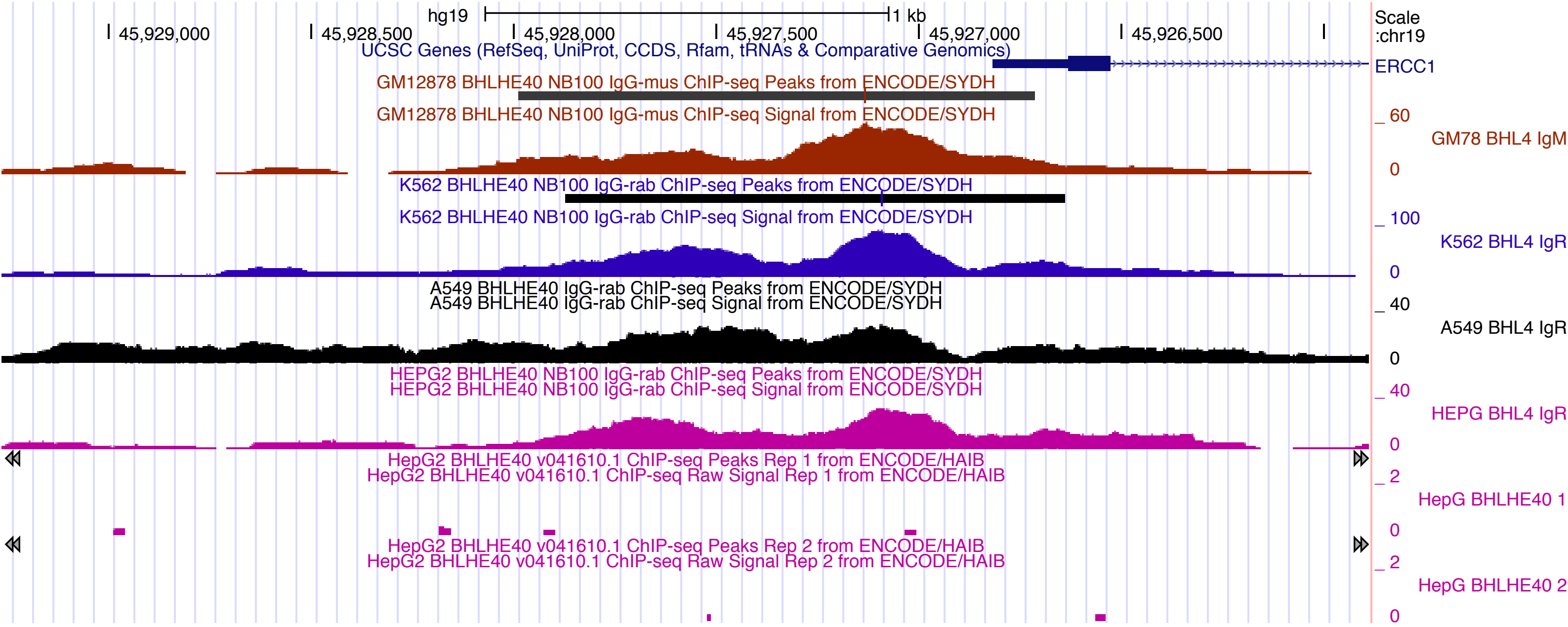

hg19
1 kb
Scale
:chr19
45,929,000
45,928,500
45,928,000
45,927,500
45,927,000
45,926,500
UCSC Genes (RefSeq, UniProt, CCDS, Rfam, tRNAs & Comparative Genomics)
ERCC1
GM12878 BHLHE40 NB100 IgG-mus ChIP-seq Peaks from ENCODE/SYDH
GM12878 BHLHE40 NB100 IgG-mus ChIP-seq Signal from ENCODE/SYDH
_ 60
GM78 BHL4 IgM
0
K562 BHLHE40 NB100 IgG-rab ChIP-seq Peaks from ENCODE/SYDH
K562 BHLHE40 NB100 IgG-rab ChIP-seq Signal from ENCODE/SYDH
_ 100
K562 BHL4 IgR
0
A549 BHLHE40 IgG-rab ChIP-seq Peaks from ENCODE/SYDH
A549 BHLHE40 IgG-rab ChIP-seq Signal from ENCODE/SYDH
_ 40
A549 BHL4 IgR
0
HEPG2 BHLHE40 NB100 IgG-rab ChIP-seq Peaks from ENCODE/SYDH
HEPG2 BHLHE40 NB100 IgG-rab ChIP-seq Signal from ENCODE/SYDH
_ 40
HEPG BHL4 IgR
0
HepG2 BHLHE40 v041610.1 ChIP-seq Peaks Rep 1 from ENCODE/HAIB
HepG2 BHLHE40 v041610.1 ChIP-seq Raw Signal Rep 1 from ENCODE/HAIB
_ 2
HepG BHLHE40 1
0
HepG2 BHLHE40 v041610.1 ChIP-seq Peaks Rep 2 from ENCODE/HAIB
HepG2 BHLHE40 v041610.1 ChIP-seq Raw Signal Rep 2 from ENCODE/HAIB
_ 2
HepG BHLHE40 2
0

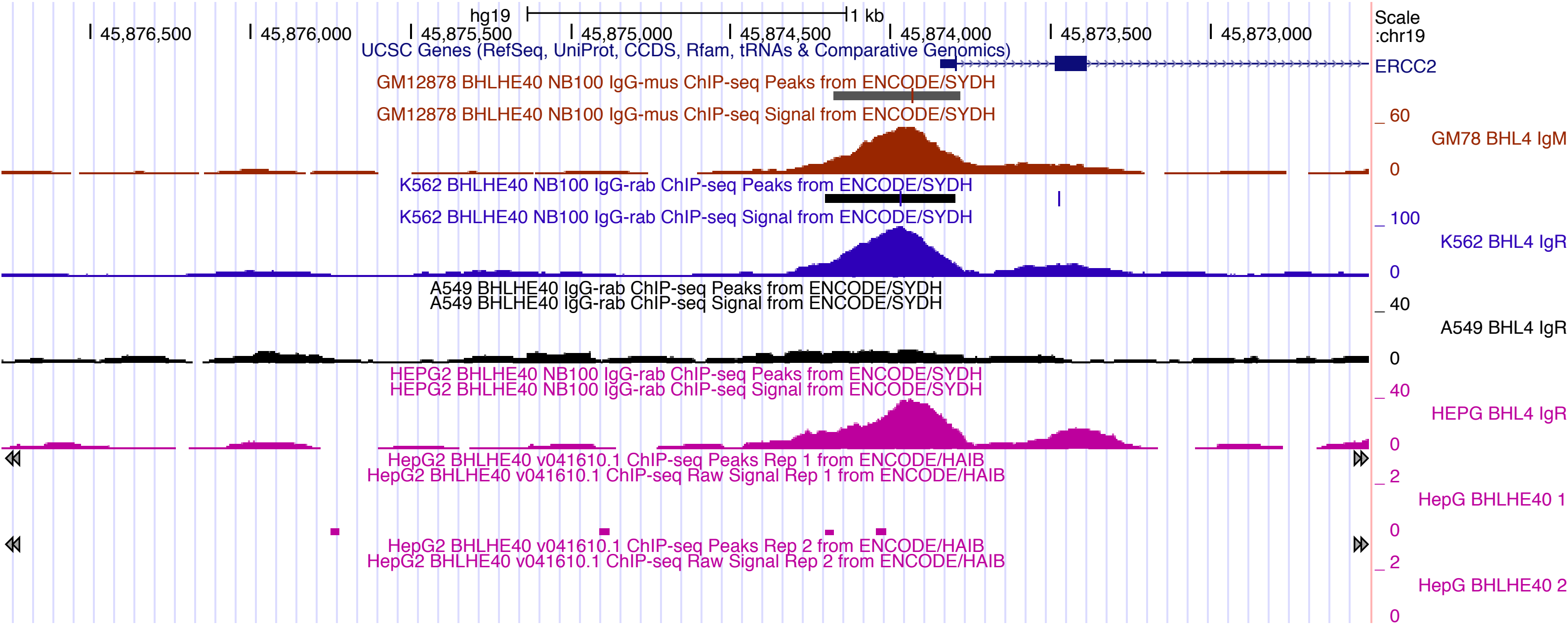

hg19
1 kb
Scale
:chr19
45,876,500
45,876,000
45,875,500
45,875,000
45,874,500
45,874,000
45,873,500
45,873,000
UCSC Genes (RefSeq, UniProt, CCDS, Rfam, tRNAs & Comparative Genomics)
ERCC2
GM12878 BHLHE40 NB100 IgG-mus ChIP-seq Peaks from ENCODE/SYDH
GM12878 BHLHE40 NB100 IgG-mus ChIP-seq Signal from ENCODE/SYDH
GM78 BHL4 IgM
K562 BHLHE40 NB100 IgG-rab ChIP-seq Peaks from ENCODE/SYDH
K562 BHLHE40 NB100 IgG-rab ChIP-seq Signal from ENCODE/SYDH
K562 BHL4 IgR
A549 BHLHE40 IgG-rab ChIP-seq Peaks from ENCODE/SYDH
A549 BHLHE40 IgG-rab ChIP-seq Signal from ENCODE/SYDH
A549 BHL4 IgR
HEPG2 BHLHE40 NB100 IgG-rab ChIP-seq Peaks from ENCODE/SYDH
HEPG2 BHLHE40 NB100 IgG-rab ChIP-seq Signal from ENCODE/SYDH
HEPG BHL4 IgR
HepG2 BHLHE40 v041610.1 ChIP-seq Peaks Rep 1 from ENCODE/HAIB
HepG2 BHLHE40 v041610.1 ChIP-seq Raw Signal Rep 1 from ENCODE/HAIB
HepG BHLHE40 1
HepG2 BHLHE40 v041610.1 ChIP-seq Peaks Rep 2 from ENCODE/HAIB
HepG2 BHLHE40 v041610.1 ChIP-seq Raw Signal Rep 2 from ENCODE/HAIB
HepG BHLHE40 2

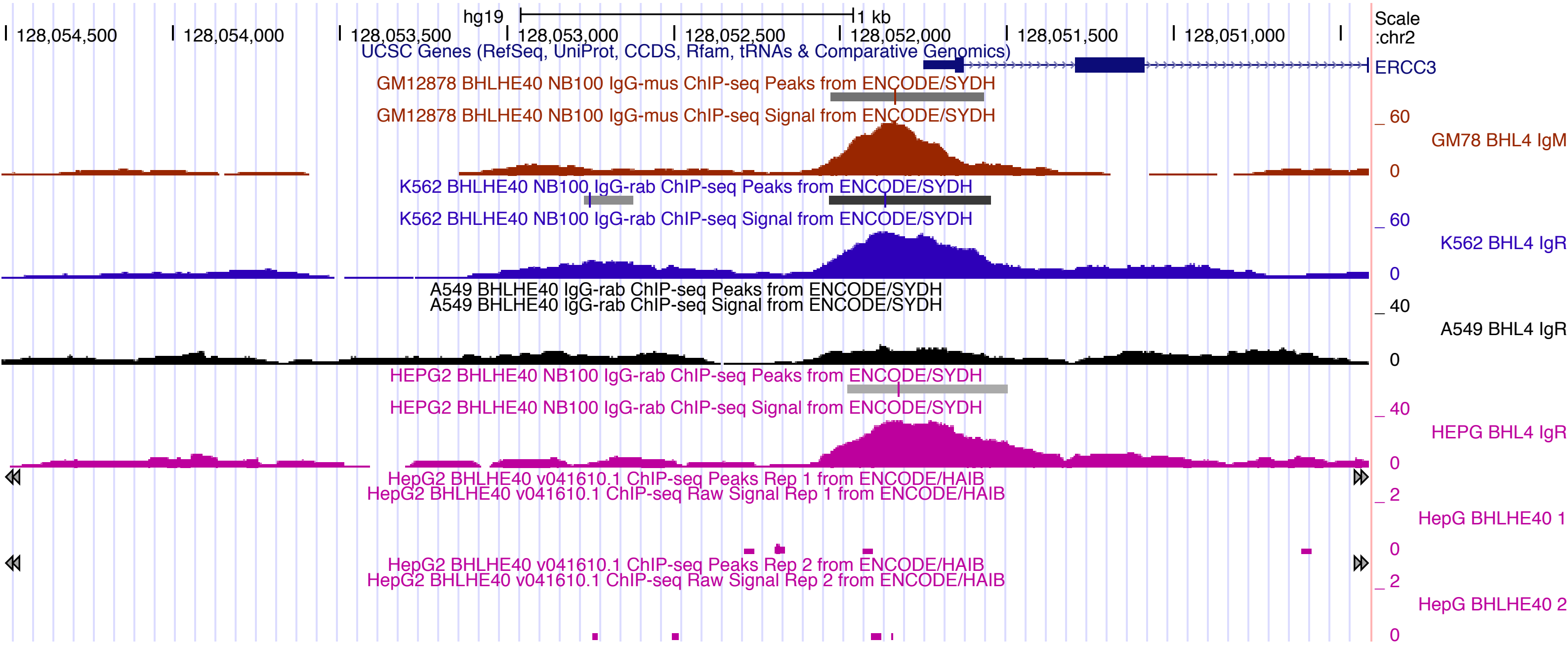

hg19
1 kb
Scale
:chr2
128,054,500
128,054,000
128,053,500
128,053,000
128,052,500
128,052,000
128,051,500
128,051,000
UCSC Genes (RefSeq, UniProt, CCDS, Rfam, tRNAs & Comparative Genomics)
ERCC3
GM12878 BHLHE40 NB100 IgG-mus ChIP-seq Peaks from ENCODE/SYDH
GM12878 BHLHE40 NB100 IgG-mus ChIP-seq Signal from ENCODE/SYDH
GM78 BHL4 IgM
K562 BHLHE40 NB100 IgG-rab ChIP-seq Peaks from ENCODE/SYDH
K562 BHLHE40 NB100 IgG-rab ChIP-seq Signal from ENCODE/SYDH
K562 BHL4 IgR
A549 BHLHE40 IgG-rab ChIP-seq Peaks from ENCODE/SYDH
A549 BHLHE40 IgG-rab ChIP-seq Signal from ENCODE/SYDH
A549 BHL4 IgR
HEPG2 BHLHE40 NB100 IgG-rab ChIP-seq Peaks from ENCODE/SYDH
HEPG2 BHLHE40 NB100 IgG-rab ChIP-seq Signal from ENCODE/SYDH
HEPG BHL4 IgR
HepG2 BHLHE40 v041610.1 ChIP-seq Peaks Rep 1 from ENCODE/HAIB
HepG2 BHLHE40 v041610.1 ChIP-seq Raw Signal Rep 1 from ENCODE/HAIB
HepG BHLHE40 1
HepG2 BHLHE40 v041610.1 ChIP-seq Peaks Rep 2 from ENCODE/HAIB
HepG2 BHLHE40 v041610.1 ChIP-seq Raw Signal Rep 2 from ENCODE/HAIB
HepG BHLHE40 2
60
0
60
0
40
0
40
0
2
0
2
0

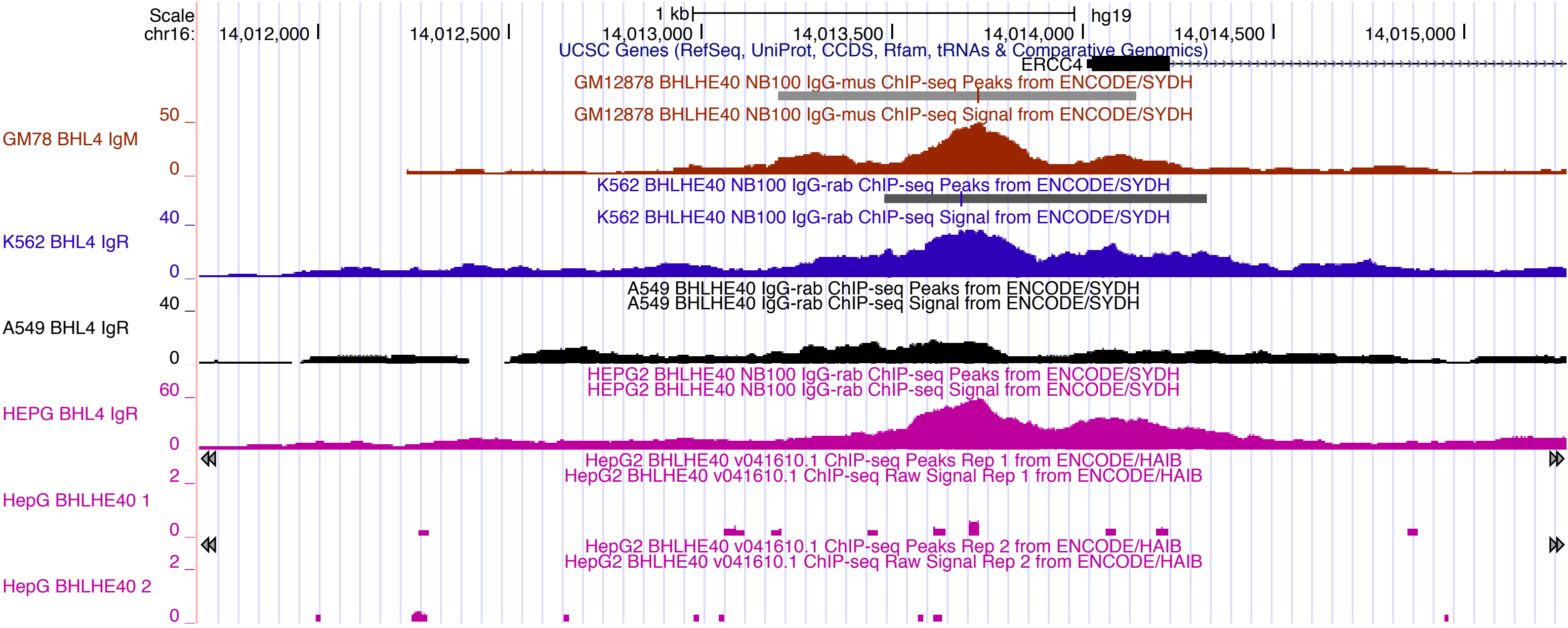

Scale
chr16:
1 kb
hg19
14,012,000
14,012,500
14,013,000
14,013,500
14,014,000
14,014,500
14,015,000
UCSC Genes (RefSeq, UniProt, CCDS, Rfam, tRNAs & Comparative Genomics)
ERCC4
GM12878 BHLHE40 NB100 IgG-mus ChIP-seq Peaks from ENCODE/SYDH
GM12878 BHLHE40 NB100 IgG-mus ChIP-seq Signal from ENCODE/SYDH
GM78 BHL4 IgM
50
0
K562 BHLHE40 NB100 IgG-rab ChIP-seq Peaks from ENCODE/SYDH
K562 BHLHE40 NB100 IgG-rab ChIP-seq Signal from ENCODE/SYDH
K562 BHL4 IgR
40
0
A549 BHLHE40 IgG-rab ChIP-seq Peaks from ENCODE/SYDH
A549 BHLHE40 IgG-rab ChIP-seq Signal from ENCODE/SYDH
A549 BHL4 IgR
40
0
HEPG2 BHLHE40 NB100 IgG-rab ChIP-seq Peaks from ENCODE/SYDH
HEPG2 BHLHE40 NB100 IgG-rab ChIP-seq Signal from ENCODE/SYDH
HEPG BHL4 IgR
60
0
HepG2 BHLHE40 v041610.1 ChIP-seq Peaks Rep 1 from ENCODE/HAIB
HepG2 BHLHE40 v041610.1 ChIP-seq Raw Signal Rep 1 from ENCODE/HAIB
HepG BHLHE40 1
2
0
HepG2 BHLHE40 v041610.1 ChIP-seq Peaks Rep 2 from ENCODE/HAIB
HepG2 BHLHE40 v041610.1 ChIP-seq Raw Signal Rep 2 from ENCODE/HAIB
HepG BHLHE40 2
2
0

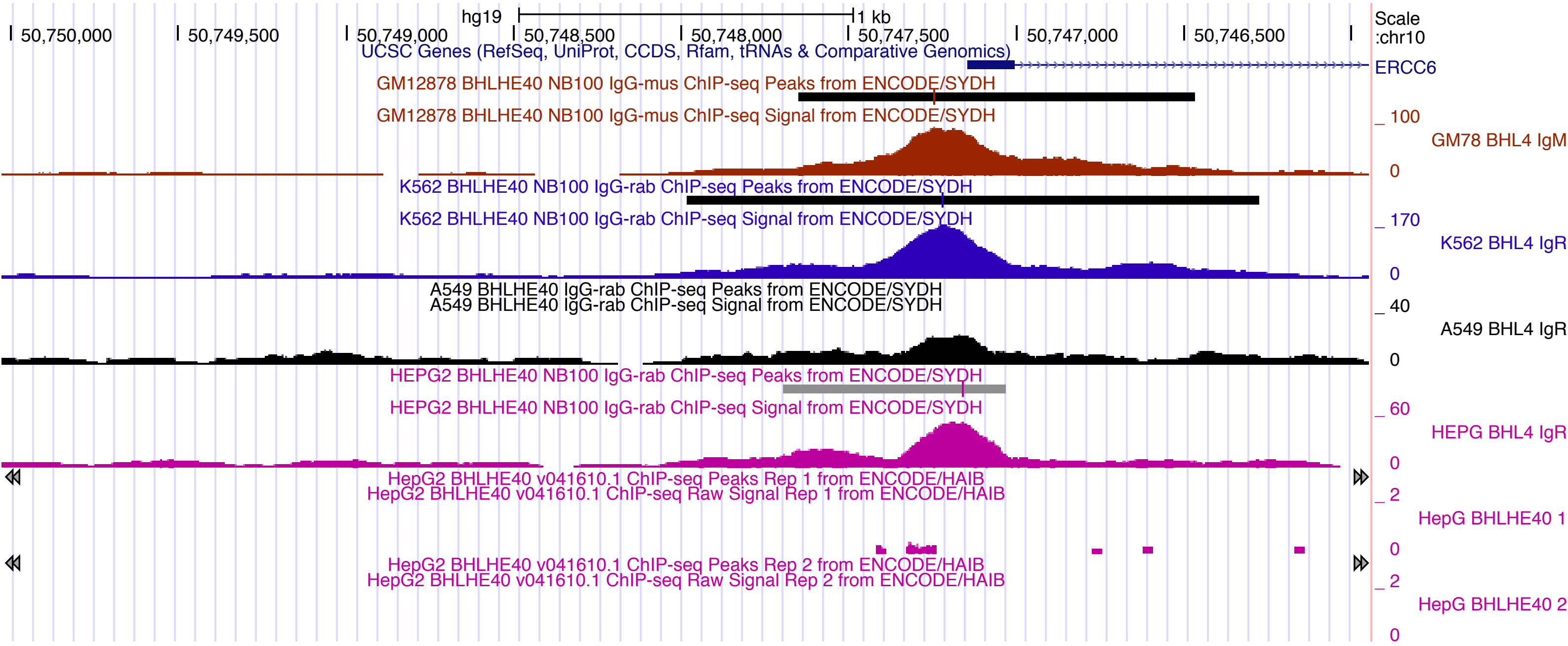

hg19
1 kb
Scale
:chr10
50,750,000
50,749,500
50,749,000
50,748,500
50,748,000
50,747,500
50,747,000
50,746,500
UCSC Genes (RefSeq, UniProt, CCDS, Rfam, tRNAs & Comparative Genomics)
ERCC6
GM12878 BHLHE40 NB100 IgG-mus ChIP-seq Peaks from ENCODE/SYDH
GM12878 BHLHE40 NB100 IgG-mus ChIP-seq Signal from ENCODE/SYDH
100
GM78 BHL4 IgM
0
K562 BHLHE40 NB100 IgG-rab ChIP-seq Peaks from ENCODE/SYDH
K562 BHLHE40 NB100 IgG-rab ChIP-seq Signal from ENCODE/SYDH
170
K562 BHL4 IgR
0
A549 BHLHE40 IgG-rab ChIP-seq Peaks from ENCODE/SYDH
A549 BHLHE40 IgG-rab ChIP-seq Signal from ENCODE/SYDH
40
A549 BHL4 IgR
0
HEPG2 BHLHE40 NB100 IgG-rab ChIP-seq Peaks from ENCODE/SYDH
HEPG2 BHLHE40 NB100 IgG-rab ChIP-seq Signal from ENCODE/SYDH
60
HEPG BHL4 IgR
0
HepG2 BHLHE40 v041610.1 ChIP-seq Peaks Rep 1 from ENCODE/HAIB
HepG2 BHLHE40 v041610.1 ChIP-seq Raw Signal Rep 1 from ENCODE/HAIB
2
HepG BHLHE40 1
0
HepG2 BHLHE40 v041610.1 ChIP-seq Peaks Rep 2 from ENCODE/HAIB
HepG2 BHLHE40 v041610.1 ChIP-seq Raw Signal Rep 2 from ENCODE/HAIB
2
HepG BHLHE40 2
0

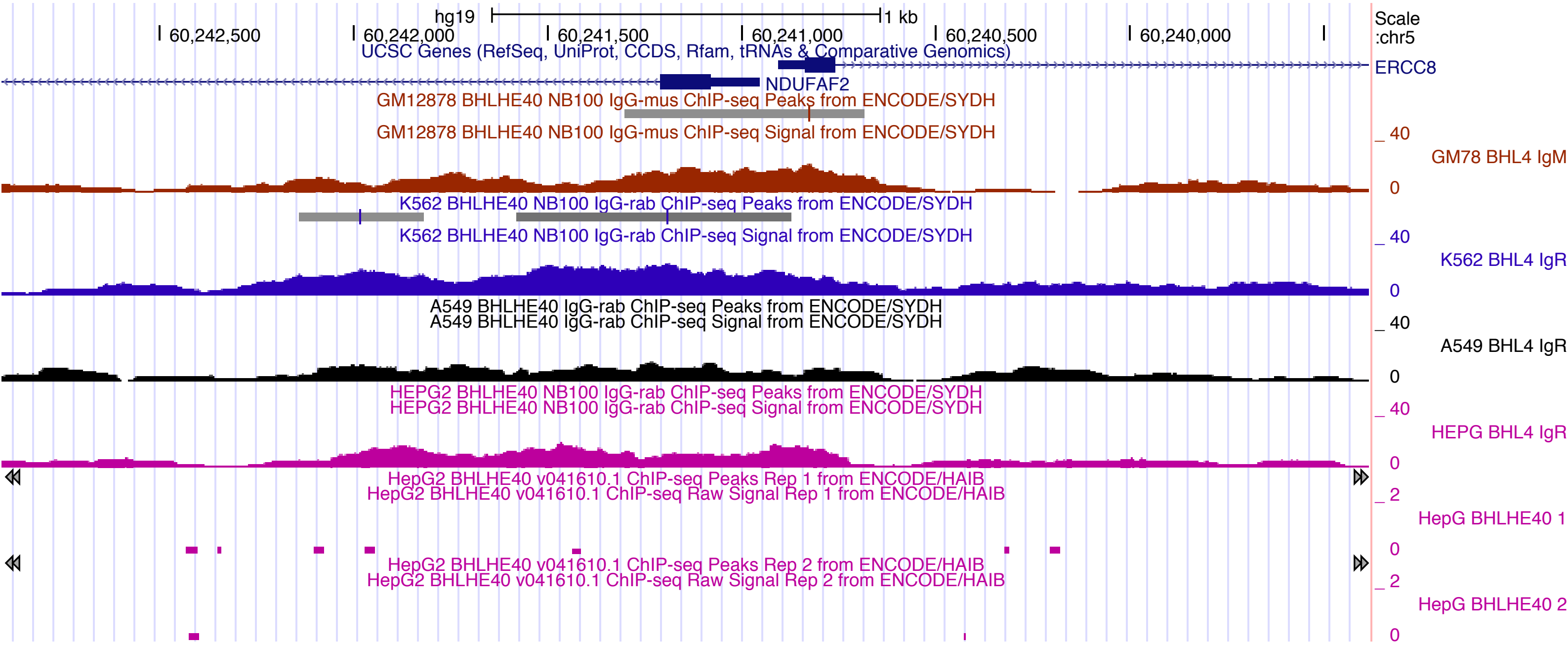
hg19
1 kb
Scale
:chr5
60,242,500
60,242,000
60,241,500
60,241,000
60,240,500
60,240,000
UCSC Genes (RefSeq, UniProt, CCDS, Rfam, tRNAs & Comparative Genomics)
ERCC8
NDUFAF2
GM12878 BHLHE40 NB100 IgG-mus ChIP-seq Peaks from ENCODE/SYDH
GM12878 BHLHE40 NB100 IgG-mus ChIP-seq Signal from ENCODE/SYDH
GM78 BHL4 IgM
K562 BHLHE40 NB100 IgG-rab ChIP-seq Peaks from ENCODE/SYDH
K562 BHLHE40 NB100 IgG-rab ChIP-seq Signal from ENCODE/SYDH
K562 BHL4 IgR
A549 BHLHE40 IgG-rab ChIP-seq Peaks from ENCODE/SYDH
A549 BHLHE40 IgG-rab ChIP-seq Signal from ENCODE/SYDH
A549 BHL4 IgR
HEPG2 BHLHE40 NB100 IgG-rab ChIP-seq Peaks from ENCODE/SYDH
HEPG2 BHLHE40 NB100 IgG-rab ChIP-seq Signal from ENCODE/SYDH
HEPG BHL4 IgR
HepG2 BHLHE40 v041610.1 ChIP-seq Peaks Rep 1 from ENCODE/HAIB
HepG2 BHLHE40 v041610.1 ChIP-seq Raw Signal Rep 1 from ENCODE/HAIB
HepG BHLHE40 1
HepG2 BHLHE40 v041610.1 ChIP-seq Peaks Rep 2 from ENCODE/HAIB
HepG2 BHLHE40 v041610.1 ChIP-seq Raw Signal Rep 2 from ENCODE/HAIB
HepG BHLHE40 2

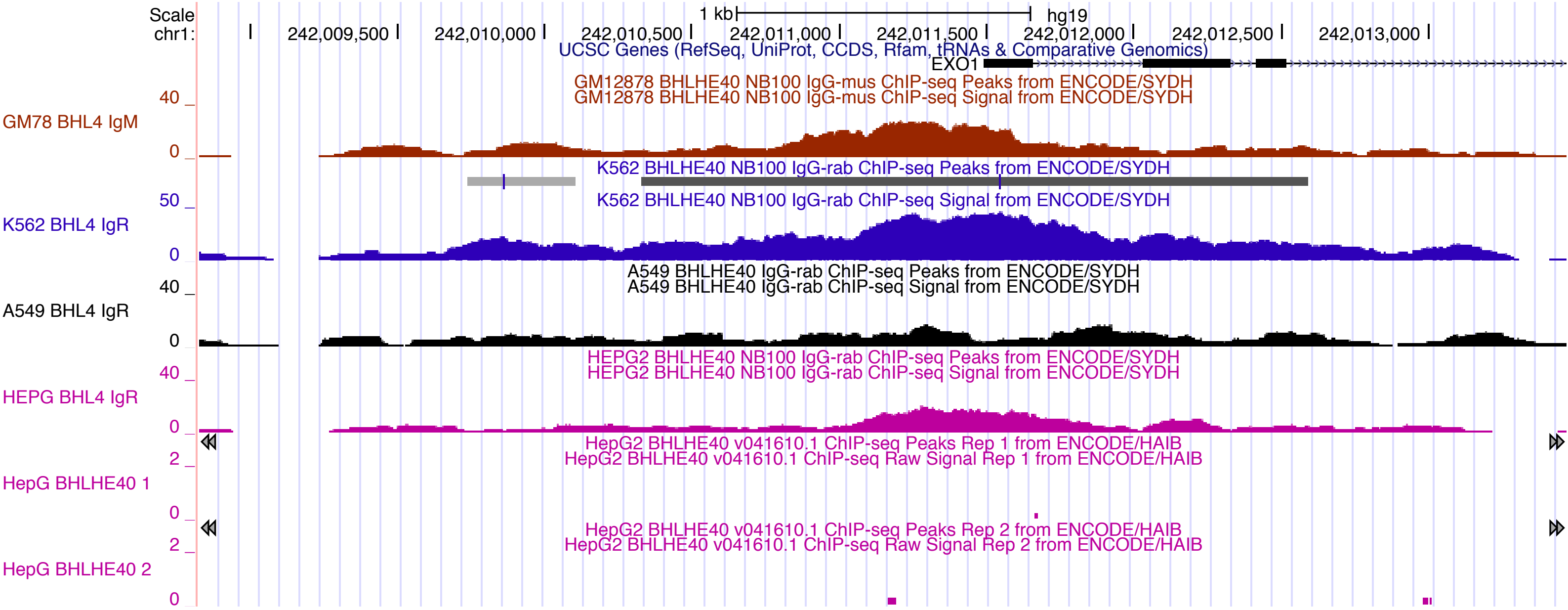

Scale
chr1:
1 kb
hg19
242,009,500
242,010,000
242,010,500
242,011,000
242,011,500
242,012,000
242,012,500
242,013,000
UCSC Genes (RefSeq, UniProt, CCDS, Rfam, tRNAs & Comparative Genomics)
EXO1
GM12878 BHLHE40 NB100 IgG-mus ChIP-seq Peaks from ENCODE/SYDH
GM12878 BHLHE40 NB100 IgG-mus ChIP-seq Signal from ENCODE/SYDH
40
GM78 BHL4 IgM
0
K562 BHLHE40 NB100 IgG-rab ChIP-seq Peaks from ENCODE/SYDH
50
K562 BHLHE40 NB100 IgG-rab ChIP-seq Signal from ENCODE/SYDH
K562 BHL4 IgR
0
A549 BHLHE40 IgG-rab ChIP-seq Peaks from ENCODE/SYDH
40
A549 BHLHE40 IgG-rab ChIP-seq Signal from ENCODE/SYDH
A549 BHL4 IgR
0
HEPG2 BHLHE40 NB100 IgG-rab ChIP-seq Peaks from ENCODE/SYDH
40
HEPG2 BHLHE40 NB100 IgG-rab ChIP-seq Signal from ENCODE/SYDH
HEPG BHL4 IgR
0
HepG2 BHLHE40 v041610.1 ChIP-seq Peaks Rep 1 from ENCODE/HAIB
2
HepG2 BHLHE40 v041610.1 ChIP-seq Raw Signal Rep 1 from ENCODE/HAIB
HepG BHLHE40 1
0
HepG2 BHLHE40 v041610.1 ChIP-seq Peaks Rep 2 from ENCODE/HAIB
2
HepG2 BHLHE40 v041610.1 ChIP-seq Raw Signal Rep 2 from ENCODE/HAIB
HepG BHLHE40 2
0

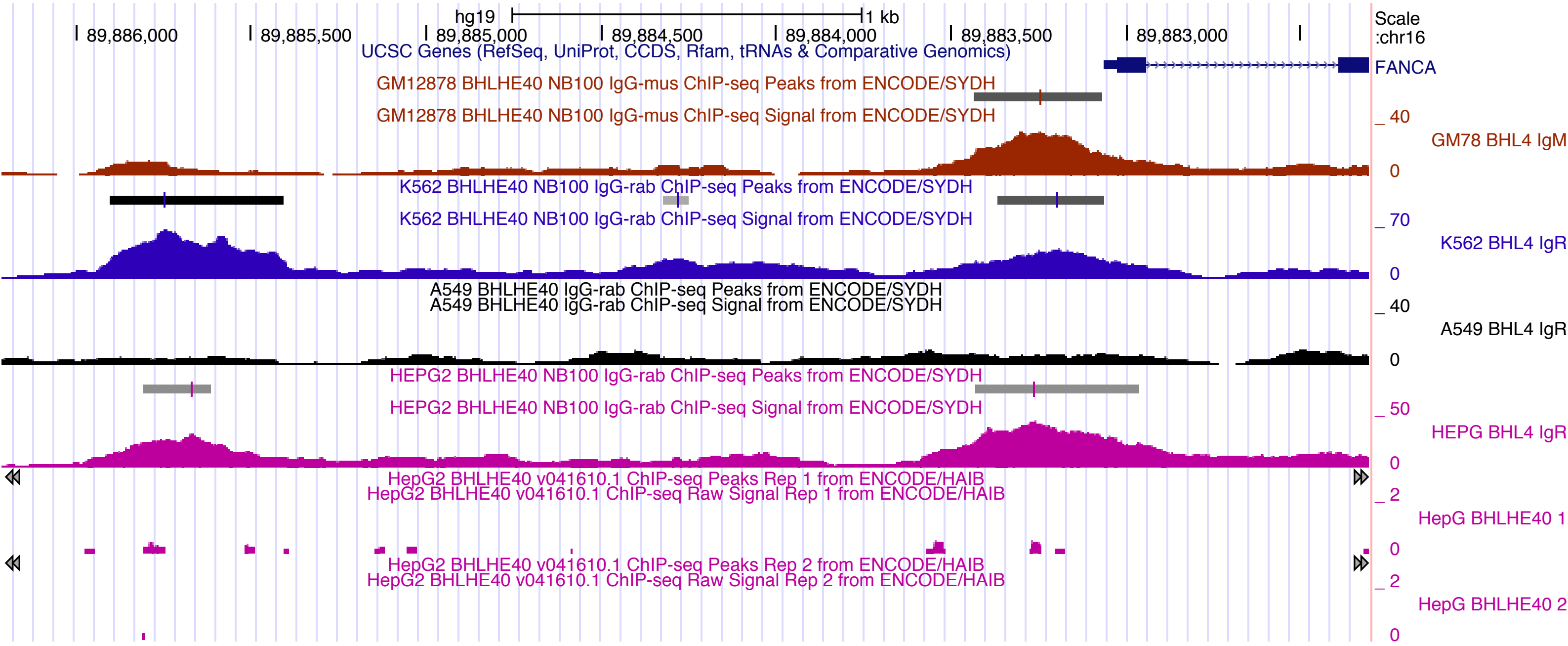

hg19
1 kb
Scale
:chr16
89,886,000
89,885,500
89,885,000
89,884,500
89,884,000
89,883,500
89,883,000
UCSC Genes (RefSeq, UniProt, CCDS, Rfam, tRNAs & Comparative Genomics)
FANCA
GM12878 BHLHE40 NB100 IgG-mus ChIP-seq Peaks from ENCODE/SYDH
GM12878 BHLHE40 NB100 IgG-mus ChIP-seq Signal from ENCODE/SYDH
_ 40
GM78 BHL4 IgM
0
K562 BHLHE40 NB100 IgG-rab ChIP-seq Peaks from ENCODE/SYDH
K562 BHLHE40 NB100 IgG-rab ChIP-seq Signal from ENCODE/SYDH
_ 70
K562 BHL4 IgR
0
A549 BHLHE40 IgG-rab ChIP-seq Peaks from ENCODE/SYDH
A549 BHLHE40 IgG-rab ChIP-seq Signal from ENCODE/SYDH
_ 40
A549 BHL4 IgR
0
HEPG2 BHLHE40 NB100 IgG-rab ChIP-seq Peaks from ENCODE/SYDH
HEPG2 BHLHE40 NB100 IgG-rab ChIP-seq Signal from ENCODE/SYDH
_ 50
HEPG BHL4 IgR
0
HepG2 BHLHE40 v041610.1 ChIP-seq Peaks Rep 1 from ENCODE/HAIB
HepG2 BHLHE40 v041610.1 ChIP-seq Raw Signal Rep 1 from ENCODE/HAIB
_ 2
HepG BHLHE40 1
0
HepG2 BHLHE40 v041610.1 ChIP-seq Peaks Rep 2 from ENCODE/HAIB
HepG2 BHLHE40 v041610.1 ChIP-seq Raw Signal Rep 2 from ENCODE/HAIB
_ 2
HepG BHLHE40 2
0

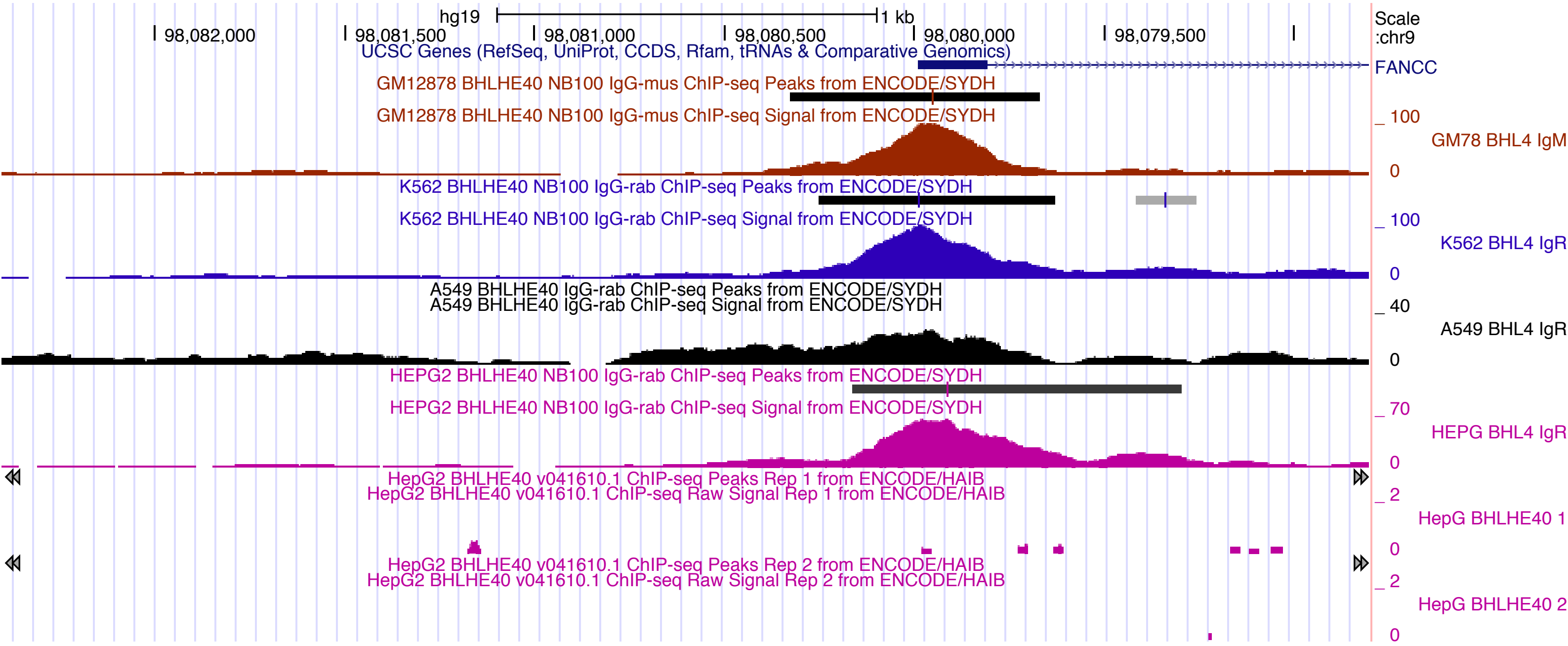
hg19
1 kb
Scale
:chr9
98,082,000
98,081,500
98,081,000
98,080,500
98,080,000
98,079,500
UCSC Genes (RefSeq, UniProt, CCDS, Rfam, tRNAs & Comparative Genomics)
FANCC
GM12878 BHLHE40 NB100 IgG-mus ChIP-seq Peaks from ENCODE/SYDH
GM12878 BHLHE40 NB100 IgG-mus ChIP-seq Signal from ENCODE/SYDH
100
GM78 BHL4 IgM
0
K562 BHLHE40 NB100 IgG-rab ChIP-seq Peaks from ENCODE/SYDH
K562 BHLHE40 NB100 IgG-rab ChIP-seq Signal from ENCODE/SYDH
100
K562 BHL4 IgR
0
A549 BHLHE40 IgG-rab ChIP-seq Peaks from ENCODE/SYDH
A549 BHLHE40 IgG-rab ChIP-seq Signal from ENCODE/SYDH
40
A549 BHL4 IgR
0
HEPG2 BHLHE40 NB100 IgG-rab ChIP-seq Peaks from ENCODE/SYDH
HEPG2 BHLHE40 NB100 IgG-rab ChIP-seq Signal from ENCODE/SYDH
70
HEPG BHL4 IgR
0
HepG2 BHLHE40 v041610.1 ChIP-seq Peaks Rep 1 from ENCODE/HAIB
HepG2 BHLHE40 v041610.1 ChIP-seq Raw Signal Rep 1 from ENCODE/HAIB
2
HepG BHLHE40 1
0
HepG2 BHLHE40 v041610.1 ChIP-seq Peaks Rep 2 from ENCODE/HAIB
HepG2 BHLHE40 v041610.1 ChIP-seq Raw Signal Rep 2 from ENCODE/HAIB
2
HepG BHLHE40 2
0

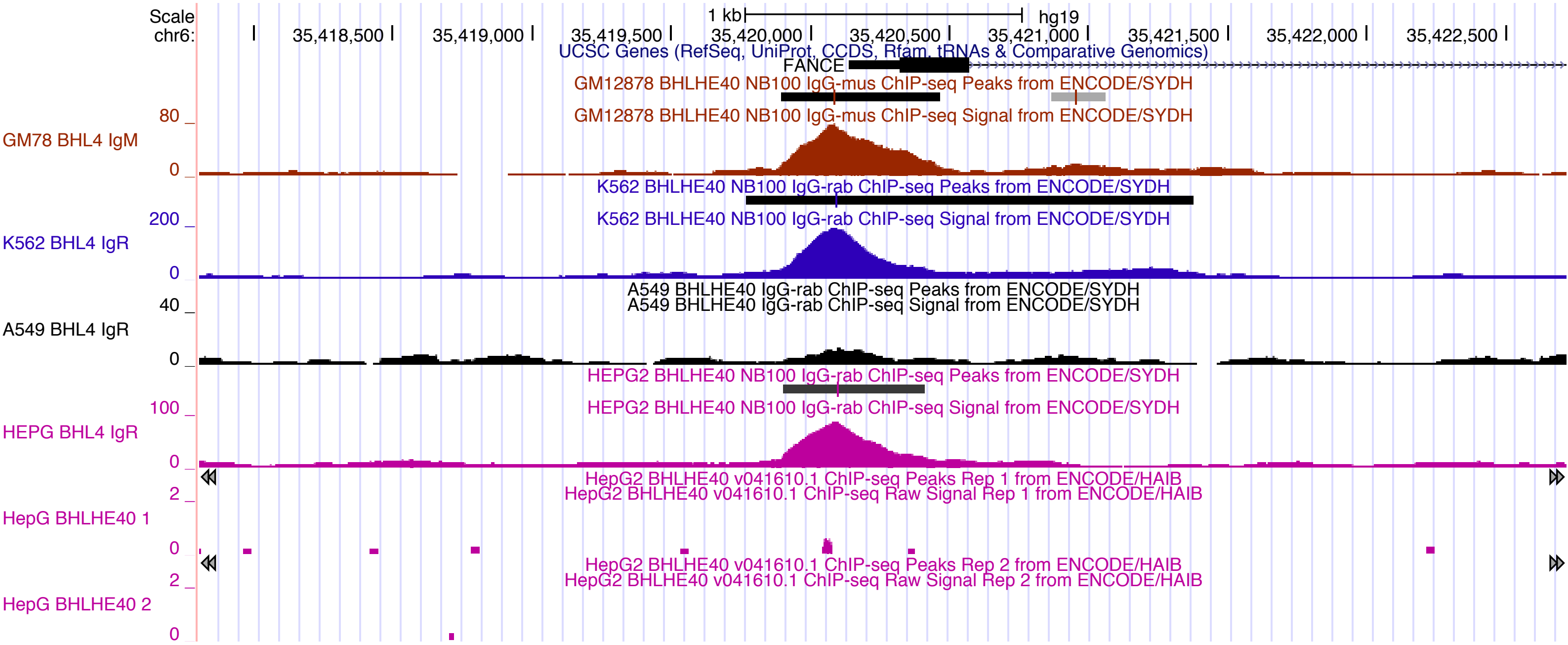
Scale
chr6:
1 kb
hg19
35,418,500
35,419,000
35,419,500
35,420,000
35,420,500
35,421,000
35,421,500
35,422,000
35,422,500
UCSC Genes (RefSeq, UniProt, CCDS, Rfam, tRNAs & Comparative Genomics)
FANCE
GM12878 BHLHE40 NB100 IgG-mus ChIP-seq Peaks from ENCODE/SYDH
GM12878 BHLHE40 NB100 IgG-mus ChIP-seq Signal from ENCODE/SYDH
GM78 BHL4 IgM
80
0
K562 BHLHE40 NB100 IgG-rab ChIP-seq Peaks from ENCODE/SYDH
K562 BHLHE40 NB100 IgG-rab ChIP-seq Signal from ENCODE/SYDH
K562 BHL4 IgR
200
0
A549 BHLHE40 IgG-rab ChIP-seq Peaks from ENCODE/SYDH
A549 BHLHE40 IgG-rab ChIP-seq Signal from ENCODE/SYDH
A549 BHL4 IgR
40
0
HEPG2 BHLHE40 NB100 IgG-rab ChIP-seq Peaks from ENCODE/SYDH
HEPG2 BHLHE40 NB100 IgG-rab ChIP-seq Signal from ENCODE/SYDH
HEPG BHL4 IgR
100
0
HepG2 BHLHE40 v041610.1 ChIP-seq Peaks Rep 1 from ENCODE/HAIB
HepG2 BHLHE40 v041610.1 ChIP-seq Raw Signal Rep 1 from ENCODE/HAIB
HepG BHLHE40 1
2
0
HepG2 BHLHE40 v041610.1 ChIP-seq Peaks Rep 2 from ENCODE/HAIB
HepG2 BHLHE40 v041610.1 ChIP-seq Raw Signal Rep 2 from ENCODE/HAIB
HepG BHLHE40 2
2
0

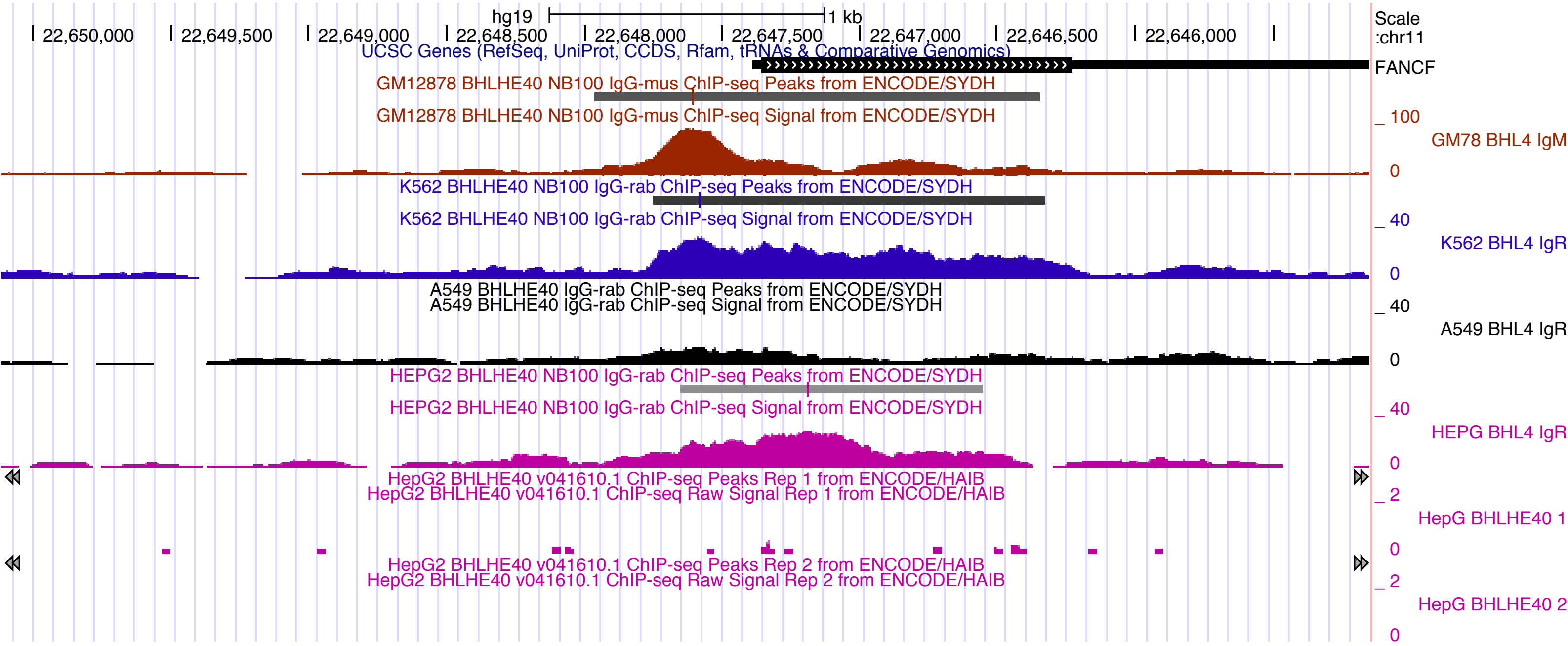

hg19
1 kb
Scale
:chr11
22,650,000
22,649,500
22,649,000
22,648,500
22,648,000
22,647,500
22,647,000
22,646,500
22,646,000
UCSC Genes (RefSeq, UniProt, CCDS, Rfam, tRNAs & Comparative Genomics)
FANCF
GM12878 BHLHE40 NB100 IgG-mus ChIP-seq Peaks from ENCODE/SYDH
GM12878 BHLHE40 NB100 IgG-mus ChIP-seq Signal from ENCODE/SYDH
100
GM78 BHL4 IgM
0
K562 BHLHE40 NB100 IgG-rab ChIP-seq Peaks from ENCODE/SYDH
K562 BHLHE40 NB100 IgG-rab ChIP-seq Signal from ENCODE/SYDH
40
K562 BHL4 IgR
0
A549 BHLHE40 IgG-rab ChIP-seq Peaks from ENCODE/SYDH
A549 BHLHE40 IgG-rab ChIP-seq Signal from ENCODE/SYDH
40
A549 BHL4 IgR
0
HEPG2 BHLHE40 NB100 IgG-rab ChIP-seq Peaks from ENCODE/SYDH
HEPG2 BHLHE40 NB100 IgG-rab ChIP-seq Signal from ENCODE/SYDH
40
HEPG BHL4 IgR
0
HepG2 BHLHE40 v041610.1 ChIP-seq Peaks Rep 1 from ENCODE/HAIB
HepG2 BHLHE40 v041610.1 ChIP-seq Raw Signal Rep 1 from ENCODE/HAIB
2
HepG BHLHE40 1
0
HepG2 BHLHE40 v041610.1 ChIP-seq Peaks Rep 2 from ENCODE/HAIB
HepG2 BHLHE40 v041610.1 ChIP-seq Raw Signal Rep 2 from ENCODE/HAIB
2
HepG BHLHE40 2
0

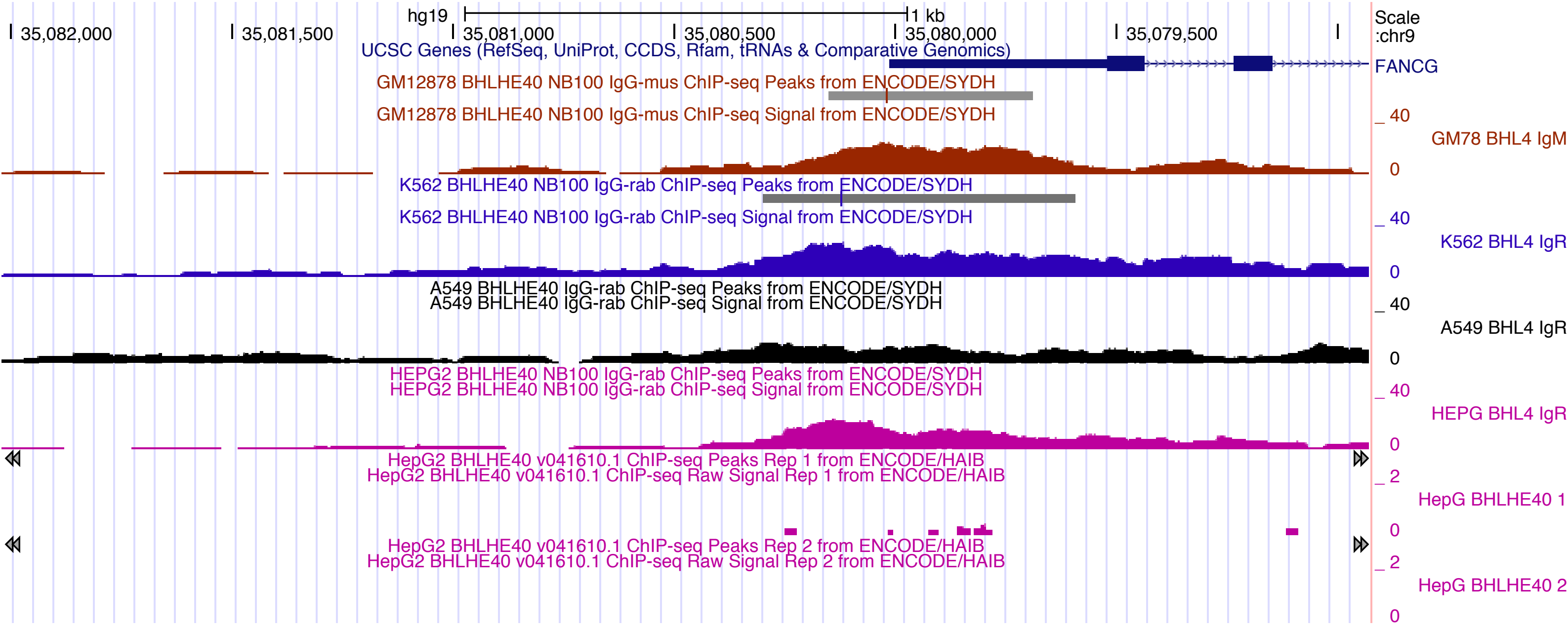

hg19
1 kb
Scale
:chr9
35,082,000
35,081,500
35,081,000
35,080,500
35,080,000
35,079,500
UCSC Genes (RefSeq, UniProt, CCDS, Rfam, tRNAs & Comparative Genomics)
FANCG
GM12878 BHLHE40 NB100 IgG-mus ChIP-seq Peaks from ENCODE/SYDH
GM12878 BHLHE40 NB100 IgG-mus ChIP-seq Signal from ENCODE/SYDH
GM78 BHL4 IgM
K562 BHLHE40 NB100 IgG-rab ChIP-seq Peaks from ENCODE/SYDH
K562 BHLHE40 NB100 IgG-rab ChIP-seq Signal from ENCODE/SYDH
K562 BHL4 IgR
A549 BHLHE40 IgG-rab ChIP-seq Peaks from ENCODE/SYDH
A549 BHLHE40 IgG-rab ChIP-seq Signal from ENCODE/SYDH
A549 BHL4 IgR
HEPG2 BHLHE40 NB100 IgG-rab ChIP-seq Peaks from ENCODE/SYDH
HEPG2 BHLHE40 NB100 IgG-rab ChIP-seq Signal from ENCODE/SYDH
HEPG BHL4 IgR
HepG2 BHLHE40 v041610.1 ChIP-seq Peaks Rep 1 from ENCODE/HAIB
HepG2 BHLHE40 v041610.1 ChIP-seq Raw Signal Rep 1 from ENCODE/HAIB
HepG BHLHE40 1
HepG2 BHLHE40 v041610.1 ChIP-seq Peaks Rep 2 from ENCODE/HAIB
HepG2 BHLHE40 v041610.1 ChIP-seq Raw Signal Rep 2 from ENCODE/HAIB
HepG BHLHE40 2
40
0
2

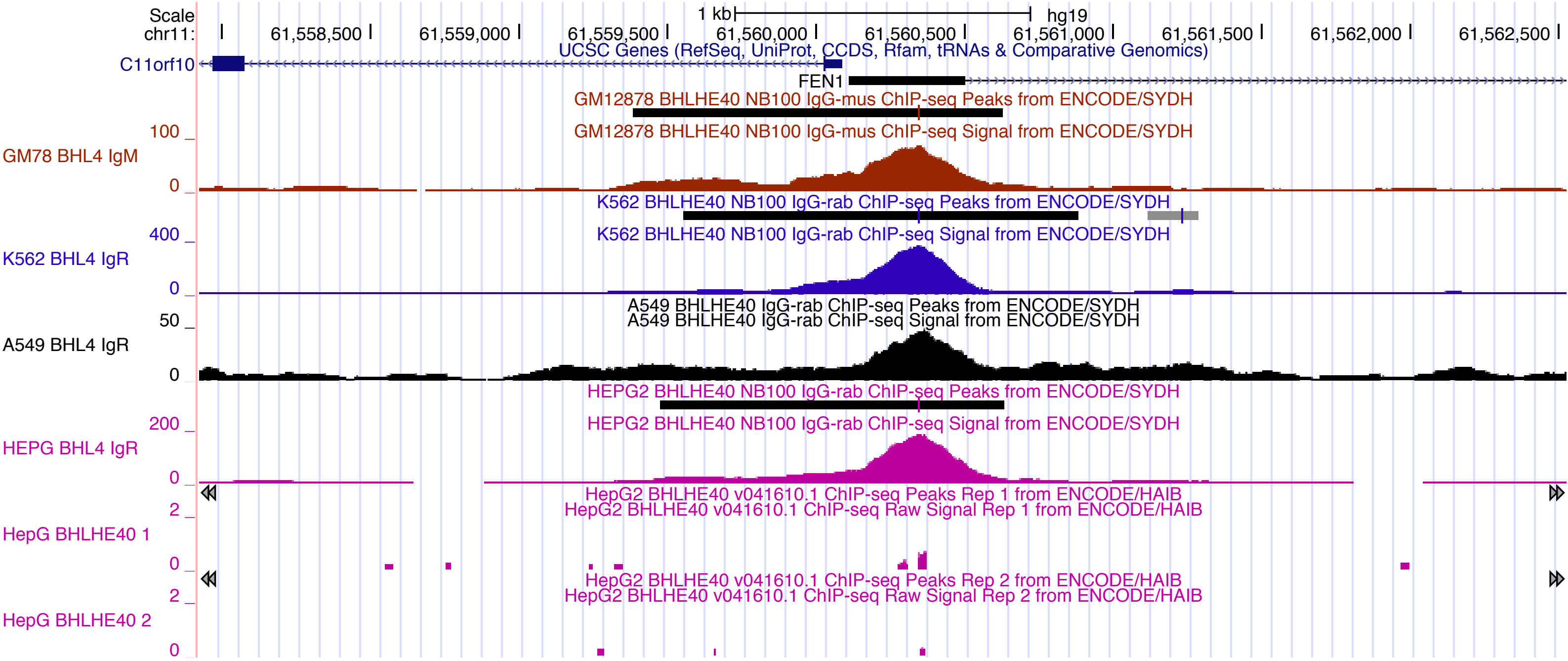

Scale
chr11:
1 kb
hg19
61,558,500
61,559,000
61,559,500
61,560,000
61,560,500
61,561,000
61,561,500
61,562,000
61,562,500
UCSC Genes (RefSeq, UniProt, CCDS, Rfam, tRNAs & Comparative Genomics)
C11orf10
FEN1
GM12878 BHLHE40 NB100 IgG-mus ChIP-seq Peaks from ENCODE/SYDH
GM12878 BHLHE40 NB100 IgG-mus ChIP-seq Signal from ENCODE/SYDH
100
GM78 BHL4 IgM
0
K562 BHLHE40 NB100 IgG-rab ChIP-seq Peaks from ENCODE/SYDH
K562 BHLHE40 NB100 IgG-rab ChIP-seq Signal from ENCODE/SYDH
400
K562 BHL4 IgR
0
A549 BHLHE40 IgG-rab ChIP-seq Peaks from ENCODE/SYDH
A549 BHLHE40 IgG-rab ChIP-seq Signal from ENCODE/SYDH
50
A549 BHL4 IgR
0
HEPG2 BHLHE40 NB100 IgG-rab ChIP-seq Peaks from ENCODE/SYDH
HEPG2 BHLHE40 NB100 IgG-rab ChIP-seq Signal from ENCODE/SYDH
200
HEPG BHL4 IgR
0
HepG2 BHLHE40 v041610.1 ChIP-seq Peaks Rep 1 from ENCODE/HAIB
HepG2 BHLHE40 v041610.1 ChIP-seq Raw Signal Rep 1 from ENCODE/HAIB
2
HepG BHLHE40 1
0
HepG2 BHLHE40 v041610.1 ChIP-seq Peaks Rep 2 from ENCODE/HAIB
HepG2 BHLHE40 v041610.1 ChIP-seq Raw Signal Rep 2 from ENCODE/HAIB
2
HepG BHLHE40 2
0

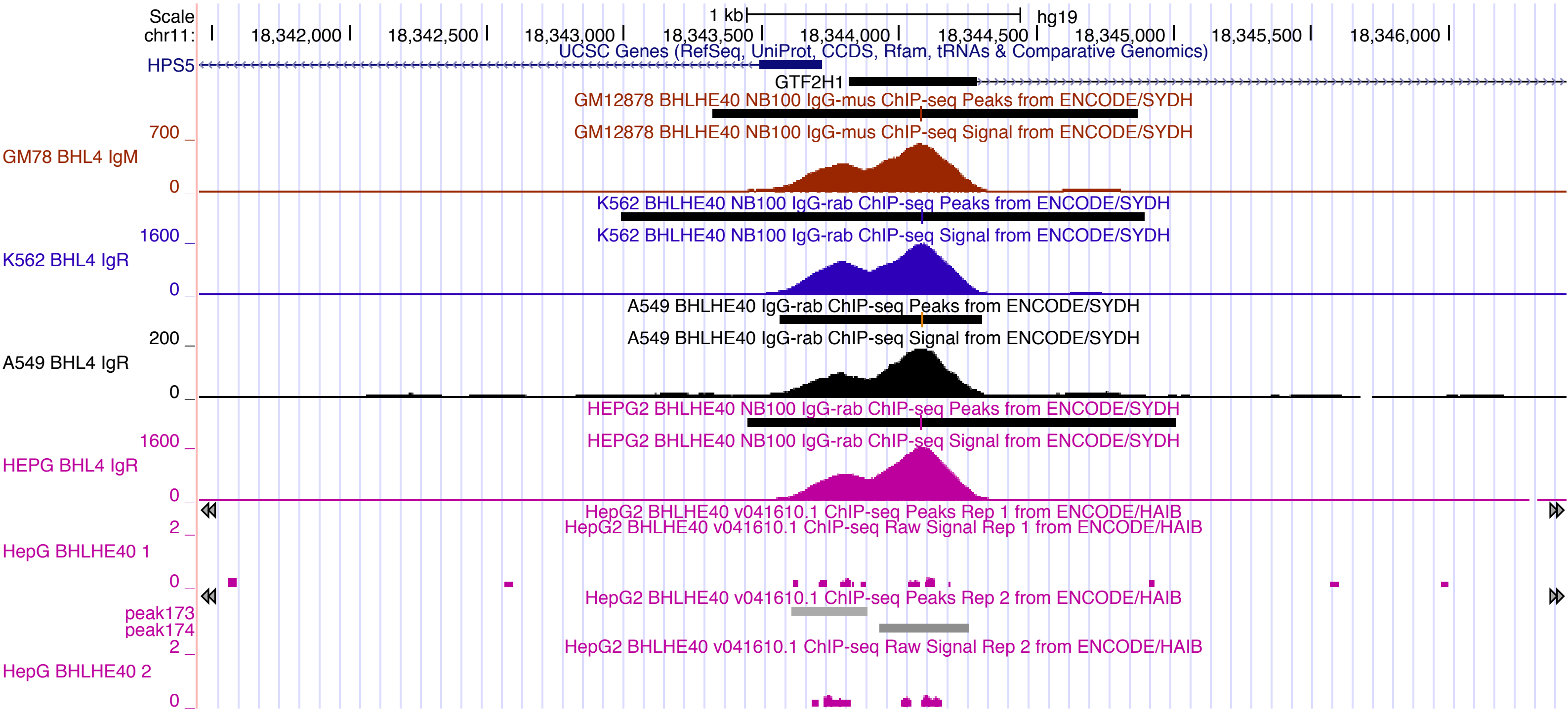
Scale
chr11:
1 kb
hg19
18,342,000
18,342,500
18,343,000
18,343,500
18,344,000
18,344,500
18,345,000
18,345,500
18,346,000
UCSC Genes (RefSeq, UniProt, CCDS, Rfam, tRNAs & Comparative Genomics)
HPS5
GTF2H1
GM12878 BHLHE40 NB100 IgG-mus ChIP-seq Peaks from ENCODE/SYDH
GM12878 BHLHE40 NB100 IgG-mus ChIP-seq Signal from ENCODE/SYDH
700
GM78 BHL4 IgM
0
K562 BHLHE40 NB100 IgG-rab ChIP-seq Peaks from ENCODE/SYDH
K562 BHLHE40 NB100 IgG-rab ChIP-seq Signal from ENCODE/SYDH
1600
K562 BHL4 IgR
0
A549 BHLHE40 IgG-rab ChIP-seq Peaks from ENCODE/SYDH
A549 BHLHE40 IgG-rab ChIP-seq Signal from ENCODE/SYDH
200
A549 BHL4 IgR
0
HEPG2 BHLHE40 NB100 IgG-rab ChIP-seq Peaks from ENCODE/SYDH
HEPG2 BHLHE40 NB100 IgG-rab ChIP-seq Signal from ENCODE/SYDH
1600
HEPG BHL4 IgR
0
HepG2 BHLHE40 v041610.1 ChIP-seq Peaks Rep 1 from ENCODE/HAIB
HepG2 BHLHE40 v041610.1 ChIP-seq Raw Signal Rep 1 from ENCODE/HAIB
2
HepG BHLHE40 1
0
HepG2 BHLHE40 v041610.1 ChIP-seq Peaks Rep 2 from ENCODE/HAIB
peak173
peak174
2
HepG2 BHLHE40 v041610.1 ChIP-seq Raw Signal Rep 2 from ENCODE/HAIB
HepG BHLHE40 2
0

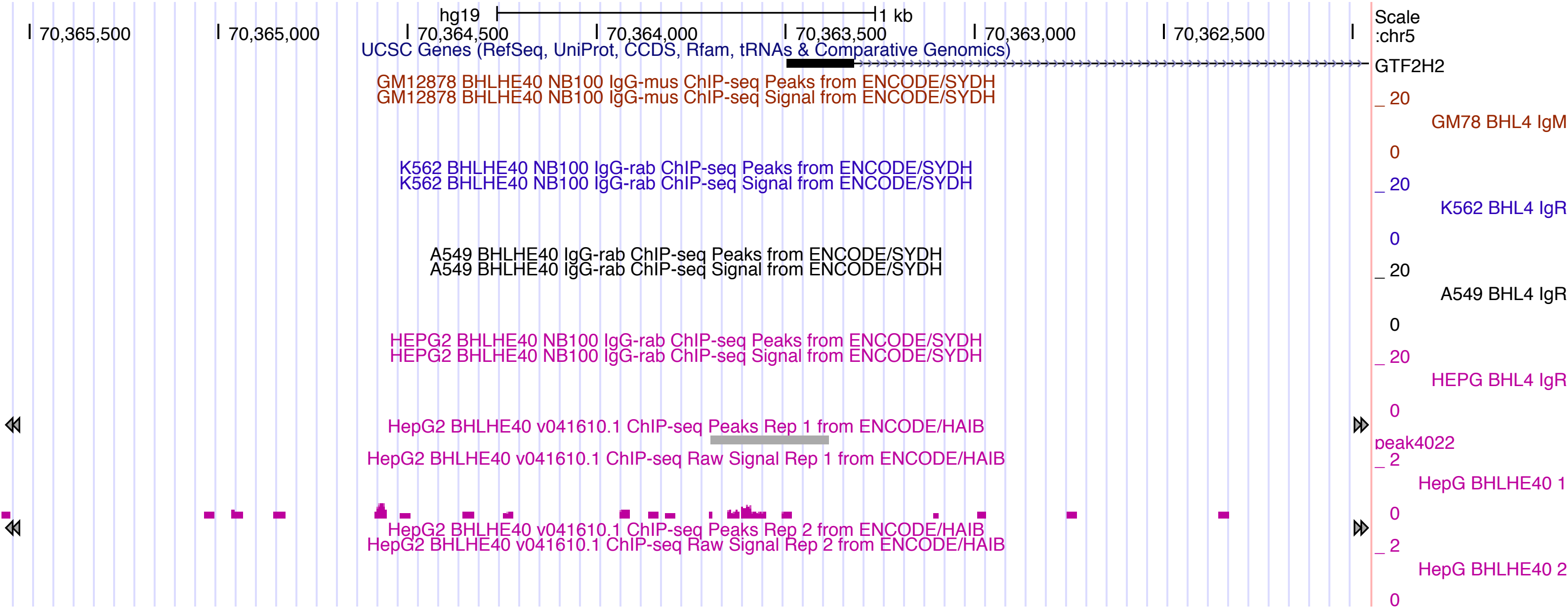

hg19
1 kb
Scale
:chr5
70,365,500
70,365,000
70,364,500
70,364,000
70,363,500
70,363,000
70,362,500
UCSC Genes (RefSeq, UniProt, CCDS, Rfam, tRNAs & Comparative Genomics)
GTF2H2
GM12878 BHLHE40 NB100 IgG-mus ChIP-seq Peaks from ENCODE/SYDH
GM12878 BHLHE40 NB100 IgG-mus ChIP-seq Signal from ENCODE/SYDH
_ 20
GM78 BHL4 IgM
0
K562 BHLHE40 NB100 IgG-rab ChIP-seq Peaks from ENCODE/SYDH
K562 BHLHE40 NB100 IgG-rab ChIP-seq Signal from ENCODE/SYDH
_ 20
K562 BHL4 IgR
0
A549 BHLHE40 IgG-rab ChIP-seq Peaks from ENCODE/SYDH
A549 BHLHE40 IgG-rab ChIP-seq Signal from ENCODE/SYDH
_ 20
A549 BHL4 IgR
0
HEPG2 BHLHE40 NB100 IgG-rab ChIP-seq Peaks from ENCODE/SYDH
HEPG2 BHLHE40 NB100 IgG-rab ChIP-seq Signal from ENCODE/SYDH
_ 20
HEPG BHL4 IgR
0
HepG2 BHLHE40 v041610.1 ChIP-seq Peaks Rep 1 from ENCODE/HAIB
peak4022
HepG2 BHLHE40 v041610.1 ChIP-seq Raw Signal Rep 1 from ENCODE/HAIB
_ 2
HepG BHLHE40 1
0
HepG2 BHLHE40 v041610.1 ChIP-seq Peaks Rep 2 from ENCODE/HAIB
HepG2 BHLHE40 v041610.1 ChIP-seq Raw Signal Rep 2 from ENCODE/HAIB
_ 2
HepG BHLHE40 2
0

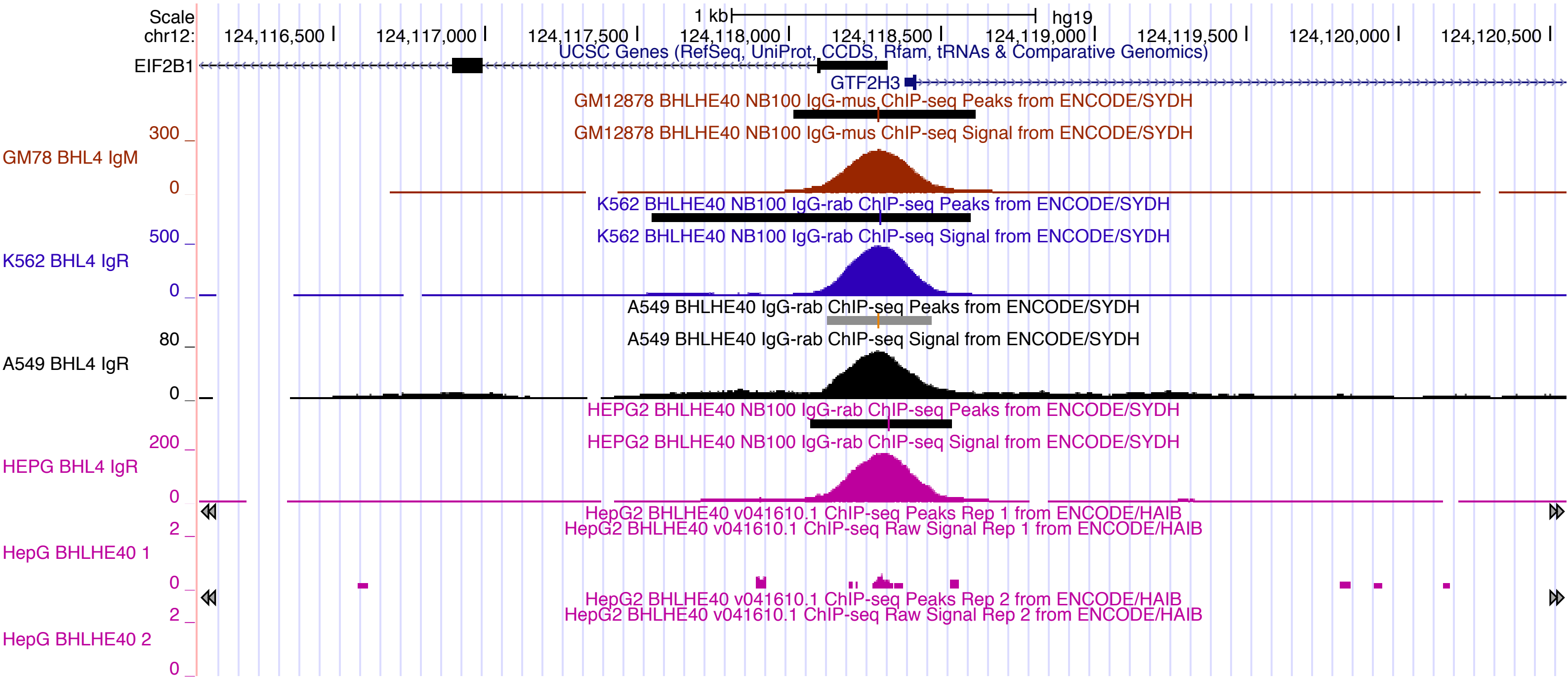

Scale
chr12:
1 kb
hg19
124,116,500
124,117,000
124,117,500
124,118,000
124,118,500
124,119,000
124,119,500
124,120,000
124,120,500
UCSC Genes (RefSeq, UniProt, CCDS, Rfam, tRNAs & Comparative Genomics)
EIF2B1
GTF2H3
GM12878 BHLHE40 NB100 IgG-mus ChIP-seq Peaks from ENCODE/SYDH
GM12878 BHLHE40 NB100 IgG-mus ChIP-seq Signal from ENCODE/SYDH
GM78 BHL4 IgM
300
0
K562 BHLHE40 NB100 IgG-rab ChIP-seq Peaks from ENCODE/SYDH
K562 BHLHE40 NB100 IgG-rab ChIP-seq Signal from ENCODE/SYDH
K562 BHL4 IgR
500
0
A549 BHLHE40 IgG-rab ChIP-seq Peaks from ENCODE/SYDH
A549 BHLHE40 IgG-rab ChIP-seq Signal from ENCODE/SYDH
A549 BHL4 IgR
80
0
HEPG2 BHLHE40 NB100 IgG-rab ChIP-seq Peaks from ENCODE/SYDH
HEPG2 BHLHE40 NB100 IgG-rab ChIP-seq Signal from ENCODE/SYDH
HEPG BHL4 IgR
200
0
HepG2 BHLHE40 v041610.1 ChIP-seq Peaks Rep 1 from ENCODE/HAIB
HepG2 BHLHE40 v041610.1 ChIP-seq Raw Signal Rep 1 from ENCODE/HAIB
HepG BHLHE40 1
2
0
HepG2 BHLHE40 v041610.1 ChIP-seq Peaks Rep 2 from ENCODE/HAIB
HepG2 BHLHE40 v041610.1 ChIP-seq Raw Signal Rep 2 from ENCODE/HAIB
HepG BHLHE40 2
2
0

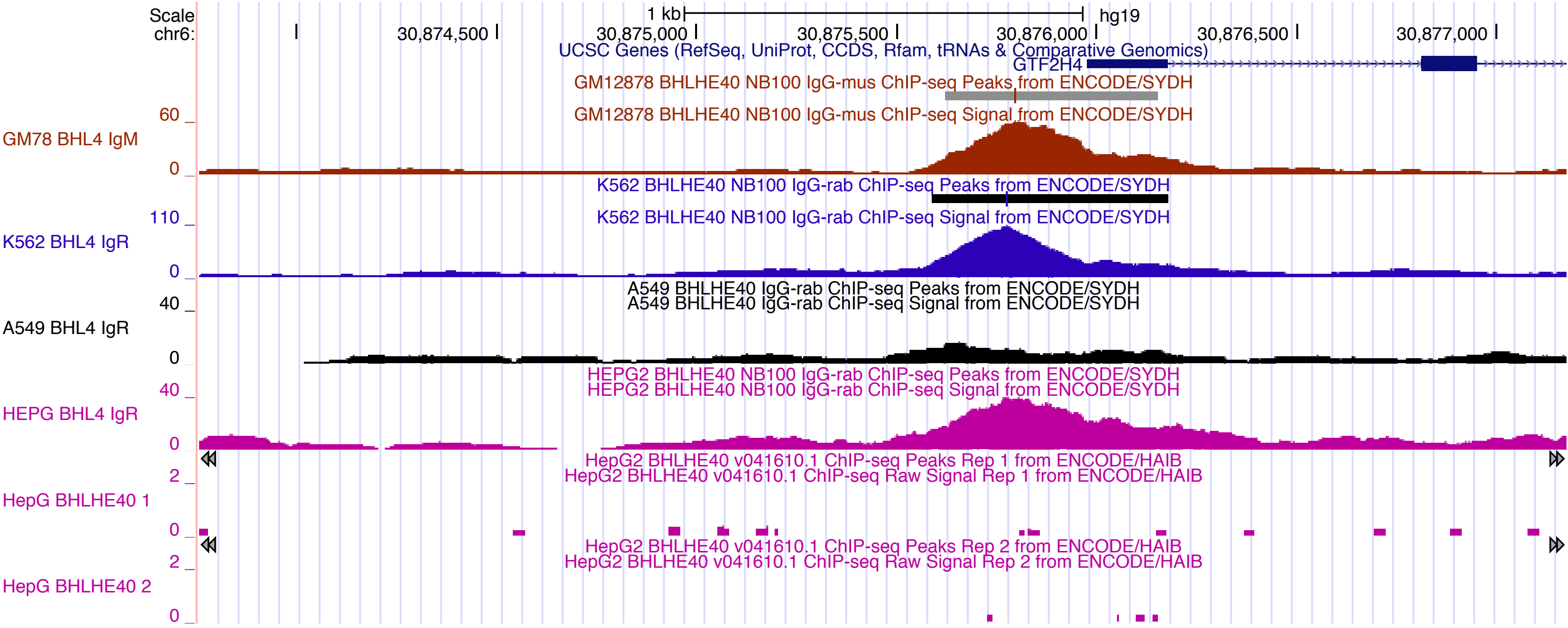
Scale
chr6:
1 kb
hg19
30,874,500
30,875,000
30,875,500
30,876,000
30,876,500
30,877,000
UCSC Genes (RefSeq, UniProt, CCDS, Rfam, tRNAs & Comparative Genomics)
GTF2H4
GM12878 BHLHE40 NB100 IgG-mus ChIP-seq Peaks from ENCODE/SYDH
GM12878 BHLHE40 NB100 IgG-mus ChIP-seq Signal from ENCODE/SYDH
60
GM78 BHL4 IgM
0
K562 BHLHE40 NB100 IgG-rab ChIP-seq Peaks from ENCODE/SYDH
K562 BHLHE40 NB100 IgG-rab ChIP-seq Signal from ENCODE/SYDH
110
K562 BHL4 IgR
0
A549 BHLHE40 IgG-rab ChIP-seq Peaks from ENCODE/SYDH
A549 BHLHE40 IgG-rab ChIP-seq Signal from ENCODE/SYDH
40
A549 BHL4 IgR
0
HEPG2 BHLHE40 NB100 IgG-rab ChIP-seq Peaks from ENCODE/SYDH
HEPG2 BHLHE40 NB100 IgG-rab ChIP-seq Signal from ENCODE/SYDH
40
HEPG BHL4 IgR
0
HepG2 BHLHE40 v041610.1 ChIP-seq Peaks Rep 1 from ENCODE/HAIB
HepG2 BHLHE40 v041610.1 ChIP-seq Raw Signal Rep 1 from ENCODE/HAIB
2
HepG BHLHE40 1
0
HepG2 BHLHE40 v041610.1 ChIP-seq Peaks Rep 2 from ENCODE/HAIB
HepG2 BHLHE40 v041610.1 ChIP-seq Raw Signal Rep 2 from ENCODE/HAIB
2
HepG BHLHE40 2
0

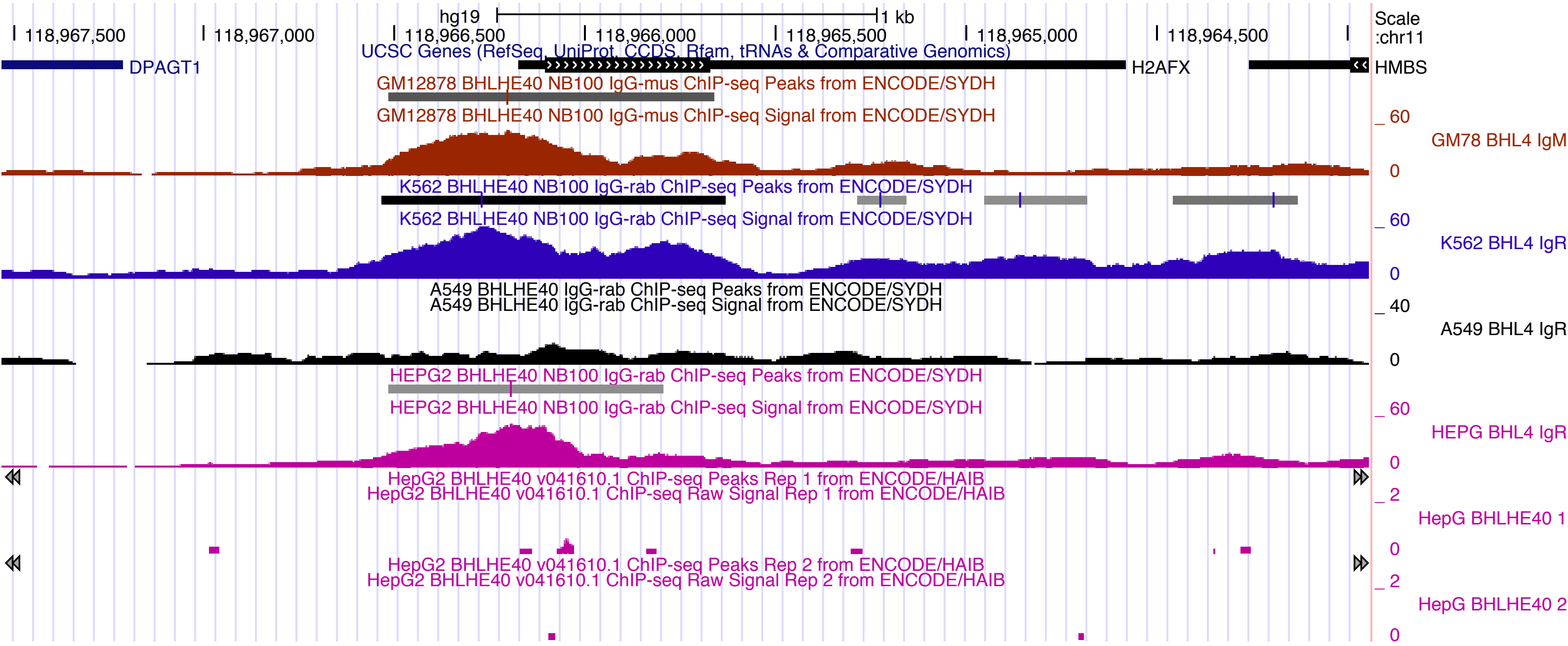

hg19
1 kb
Scale
chr11
118,967,500
118,967,000
118,966,500
118,966,000
118,965,500
118,965,000
118,964,500
UCSC Genes (RefSeq, UniProt, CCDS, Rfam, tRNAs & Comparative Genomics)
DPAGT1
H2AFX
HMBS
GM12878 BHLHE40 NB100 IgG-mus ChIP-seq Peaks from ENCODE/SYDH
GM12878 BHLHE40 NB100 IgG-mus ChIP-seq Signal from ENCODE/SYDH
GM78 BHL4 IgM
K562 BHLHE40 NB100 IgG-rab ChIP-seq Peaks from ENCODE/SYDH
K562 BHLHE40 NB100 IgG-rab ChIP-seq Signal from ENCODE/SYDH
K562 BHL4 IgR
A549 BHLHE40 IgG-rab ChIP-seq Peaks from ENCODE/SYDH
A549 BHLHE40 IgG-rab ChIP-seq Signal from ENCODE/SYDH
A549 BHL4 IgR
HEPG2 BHLHE40 NB100 IgG-rab ChIP-seq Peaks from ENCODE/SYDH
HEPG2 BHLHE40 NB100 IgG-rab ChIP-seq Signal from ENCODE/SYDH
HEPG BHL4 IgR
HepG2 BHLHE40 v041610.1 ChIP-seq Peaks Rep 1 from ENCODE/HAIB
HepG2 BHLHE40 v041610.1 ChIP-seq Raw Signal Rep 1 from ENCODE/HAIB
HepG BHLHE40 1
HepG2 BHLHE40 v041610.1 ChIP-seq Peaks Rep 2 from ENCODE/HAIB
HepG2 BHLHE40 v041610.1 ChIP-seq Raw Signal Rep 2 from ENCODE/HAIB
HepG BHLHE40 2
60
0
60
0
40
0
60
0
2
0
2
0

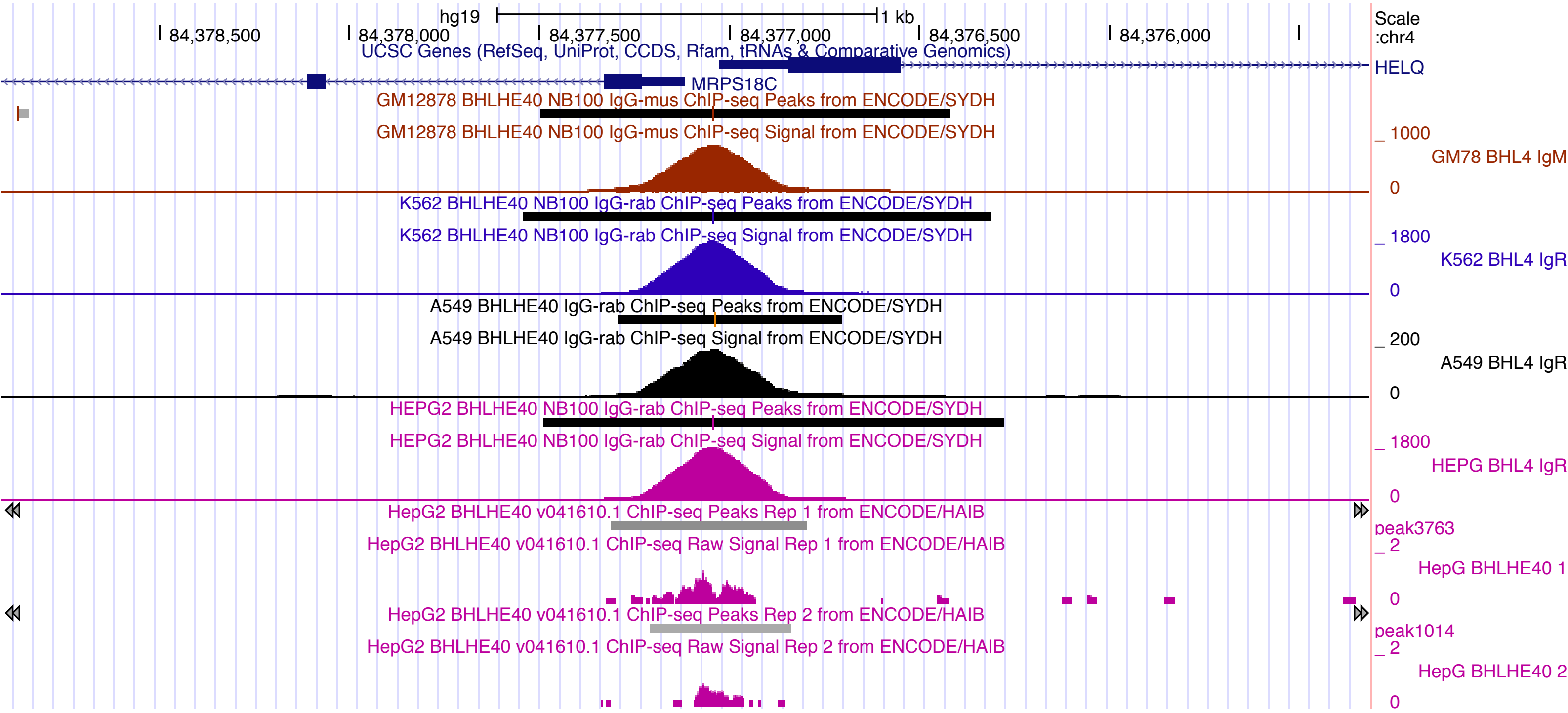
Scale
:chr4
hg19
1 kb
84,378,500
84,378,000
84,377,500
84,377,000
84,376,500
84,376,000
UCSC Genes (RefSeq, UniProt, CCDS, Rfam, tRNAs & Comparative Genomics)
HELQ
MRPS18C
GM12878 BHLHE40 NB100 IgG-mus ChIP-seq Peaks from ENCODE/SYDH
GM12878 BHLHE40 NB100 IgG-mus ChIP-seq Signal from ENCODE/SYDH
_ 1000
GM78 BHL4 IgM
0
K562 BHLHE40 NB100 IgG-rab ChIP-seq Peaks from ENCODE/SYDH
K562 BHLHE40 NB100 IgG-rab ChIP-seq Signal from ENCODE/SYDH
_ 1800
K562 BHL4 IgR
0
A549 BHLHE40 IgG-rab ChIP-seq Peaks from ENCODE/SYDH
A549 BHLHE40 IgG-rab ChIP-seq Signal from ENCODE/SYDH
_ 200
A549 BHL4 IgR
0
HEPG2 BHLHE40 NB100 IgG-rab ChIP-seq Peaks from ENCODE/SYDH
HEPG2 BHLHE40 NB100 IgG-rab ChIP-seq Signal from ENCODE/SYDH
_ 1800
HEPG BHL4 IgR
0
HepG2 BHLHE40 v041610.1 ChIP-seq Peaks Rep 1 from ENCODE/HAIB
peak3763
HepG2 BHLHE40 v041610.1 ChIP-seq Raw Signal Rep 1 from ENCODE/HAIB
_ 2
HepG BHLHE40 1
0
HepG2 BHLHE40 v041610.1 ChIP-seq Peaks Rep 2 from ENCODE/HAIB
peak1014
HepG2 BHLHE40 v041610.1 ChIP-seq Raw Signal Rep 2 from ENCODE/HAIB
_ 2
HepG BHLHE40 2
0

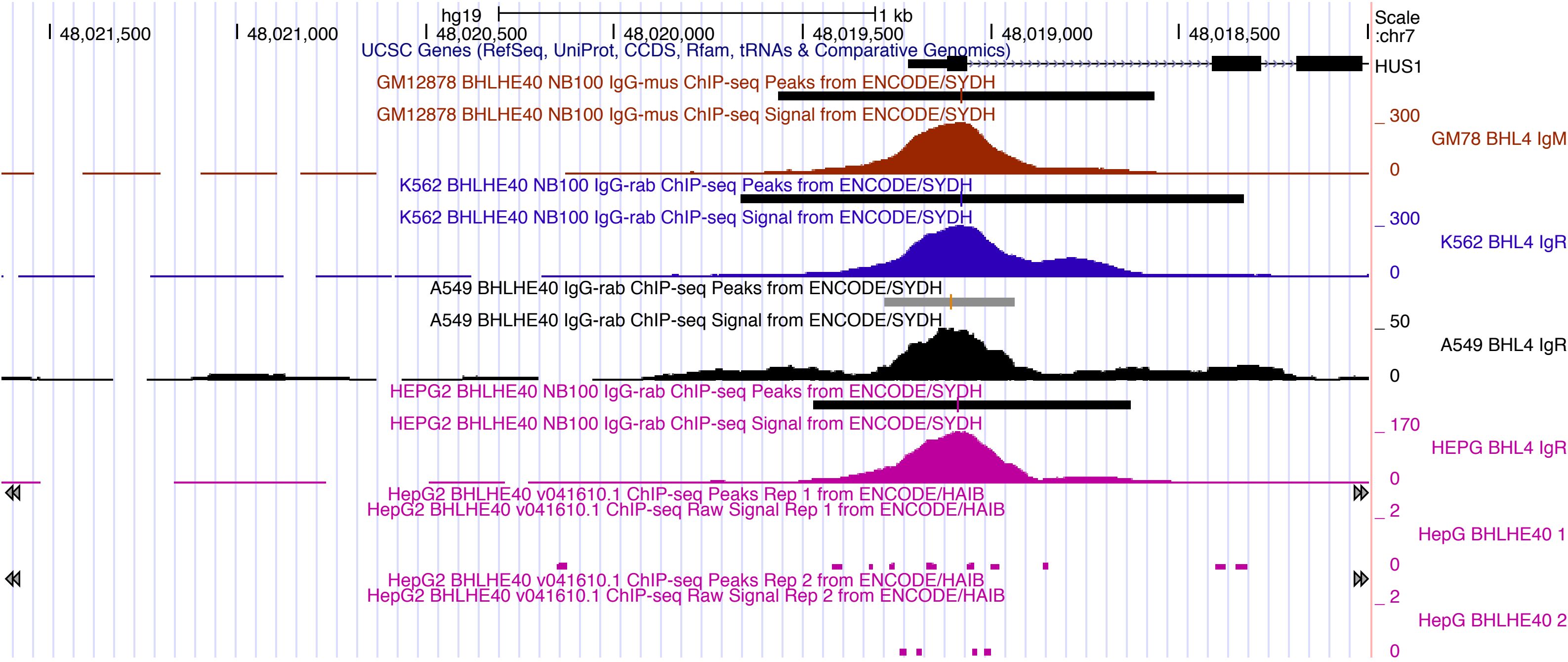

hg19
1 kb
Scale
:chr7
48,021,500
48,021,000
48,020,500
48,020,000
48,019,500
48,019,000
48,018,500
UCSC Genes (RefSeq, UniProt, CCDS, Rfam, tRNAs & Comparative Genomics)
HUS1
GM12878 BHLHE40 NB100 IgG-mus ChIP-seq Peaks from ENCODE/SYDH
GM12878 BHLHE40 NB100 IgG-mus ChIP-seq Signal from ENCODE/SYDH
300
GM78 BHL4 IgM
0
K562 BHLHE40 NB100 IgG-rab ChIP-seq Peaks from ENCODE/SYDH
K562 BHLHE40 NB100 IgG-rab ChIP-seq Signal from ENCODE/SYDH
300
K562 BHL4 IgR
0
A549 BHLHE40 IgG-rab ChIP-seq Peaks from ENCODE/SYDH
A549 BHLHE40 IgG-rab ChIP-seq Signal from ENCODE/SYDH
50
A549 BHL4 IgR
0
HEPG2 BHLHE40 NB100 IgG-rab ChIP-seq Peaks from ENCODE/SYDH
HEPG2 BHLHE40 NB100 IgG-rab ChIP-seq Signal from ENCODE/SYDH
170
HEPG BHL4 IgR
0
HepG2 BHLHE40 v041610.1 ChIP-seq Peaks Rep 1 from ENCODE/HAIB
HepG2 BHLHE40 v041610.1 ChIP-seq Raw Signal Rep 1 from ENCODE/HAIB
2
HepG BHLHE40 1
0
HepG2 BHLHE40 v041610.1 ChIP-seq Peaks Rep 2 from ENCODE/HAIB
HepG2 BHLHE40 v041610.1 ChIP-seq Raw Signal Rep 2 from ENCODE/HAIB
2
HepG BHLHE40 2
0

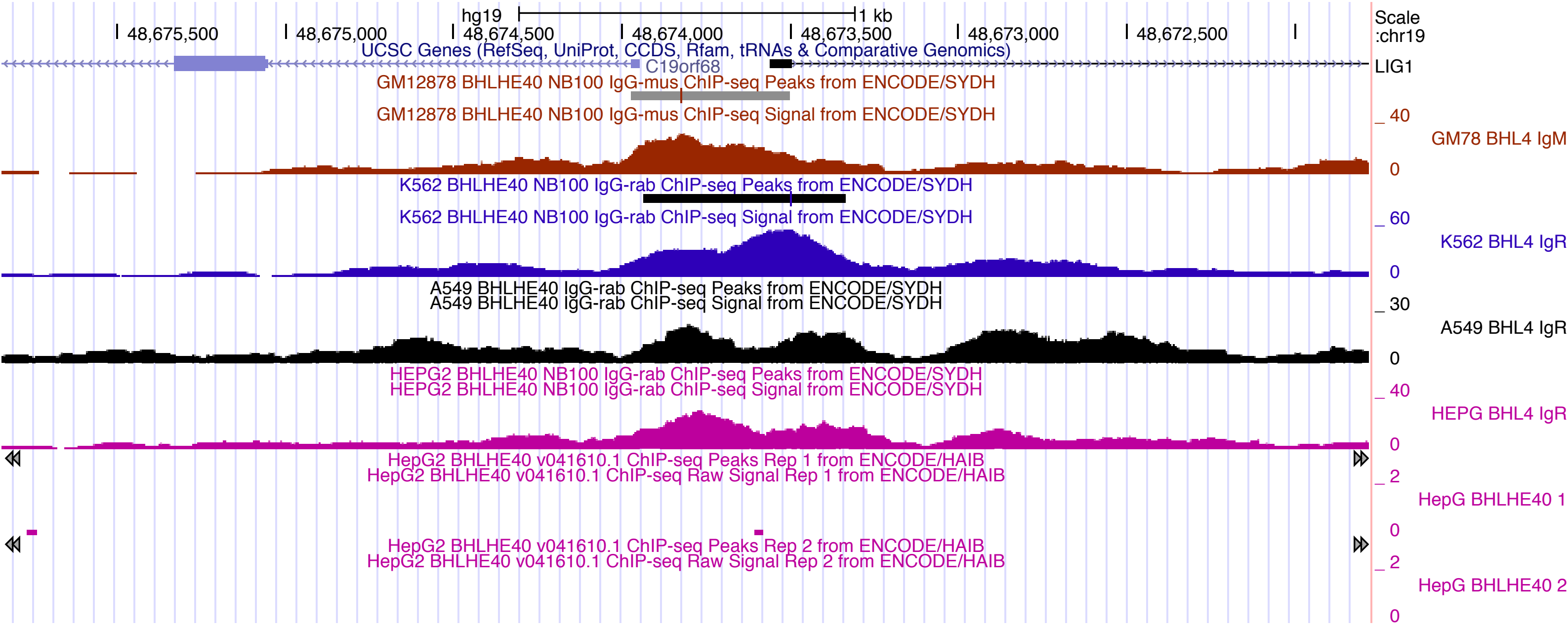
hg19
1 kb
Scale
:chr19
48,675,500
48,675,000
48,674,500
48,674,000
48,673,500
48,673,000
48,672,500
UCSC Genes (RefSeq, UniProt, CCDS, Rfam, tRNAs & Comparative Genomics)
C19orf68
LIG1
GM12878 BHLHE40 NB100 IgG-mus ChIP-seq Peaks from ENCODE/SYDH
GM12878 BHLHE40 NB100 IgG-mus ChIP-seq Signal from ENCODE/SYDH
GM78 BHL4 IgM
K562 BHLHE40 NB100 IgG-rab ChIP-seq Peaks from ENCODE/SYDH
K562 BHLHE40 NB100 IgG-rab ChIP-seq Signal from ENCODE/SYDH
K562 BHL4 IgR
A549 BHLHE40 IgG-rab ChIP-seq Peaks from ENCODE/SYDH
A549 BHLHE40 IgG-rab ChIP-seq Signal from ENCODE/SYDH
A549 BHL4 IgR
HEPG2 BHLHE40 NB100 IgG-rab ChIP-seq Peaks from ENCODE/SYDH
HEPG2 BHLHE40 NB100 IgG-rab ChIP-seq Signal from ENCODE/SYDH
HEPG BHL4 IgR
HepG2 BHLHE40 v041610.1 ChIP-seq Peaks Rep 1 from ENCODE/HAIB
HepG2 BHLHE40 v041610.1 ChIP-seq Raw Signal Rep 1 from ENCODE/HAIB
HepG BHLHE40 1
HepG2 BHLHE40 v041610.1 ChIP-seq Peaks Rep 2 from ENCODE/HAIB
HepG2 BHLHE40 v041610.1 ChIP-seq Raw Signal Rep 2 from ENCODE/HAIB
HepG BHLHE40 2

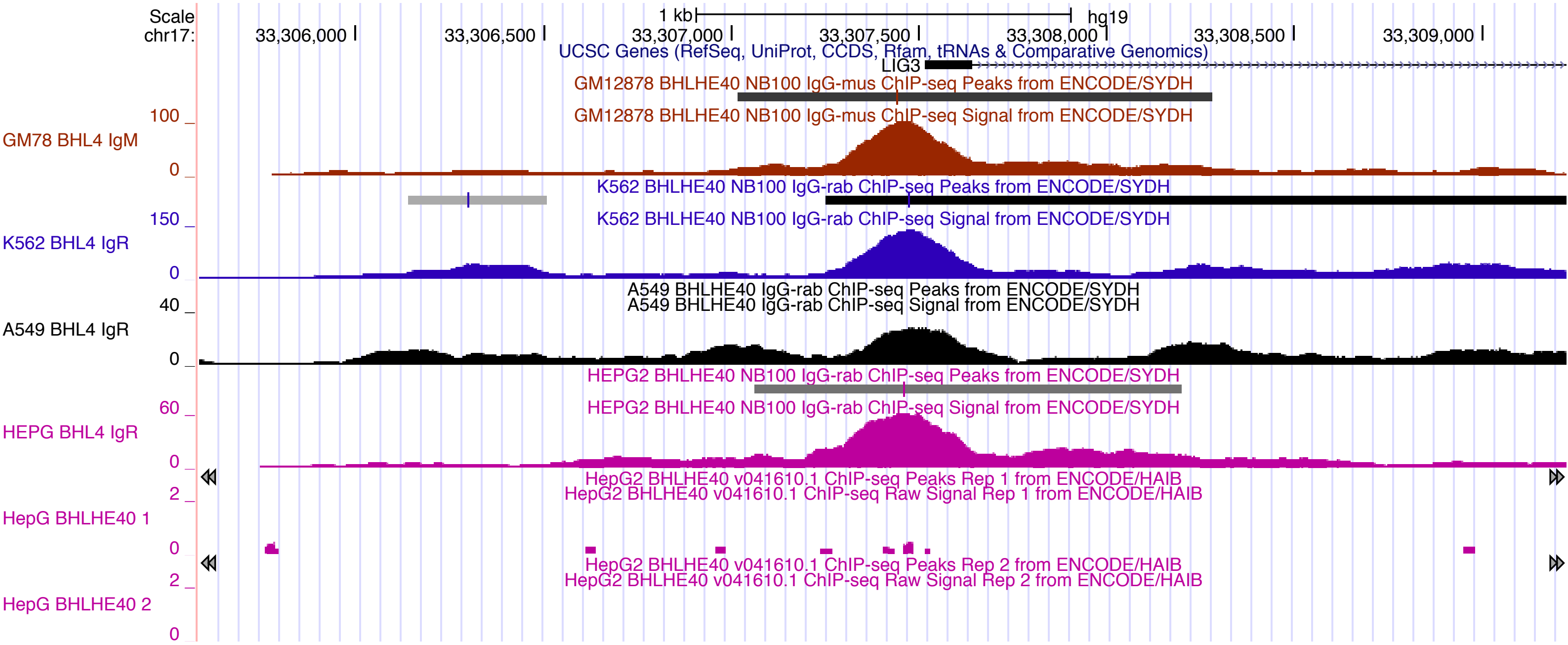

Scale
chr17:
1 kb
hg19
33,306,000
33,306,500
33,307,000
33,307,500
33,308,000
33,308,500
33,309,000
UCSC Genes (RefSeq, UniProt, CCDS, Rfam, tRNAs & Comparative Genomics)
LIG3
GM12878 BHLHE40 NB100 IgG-mus ChIP-seq Peaks from ENCODE/SYDH
GM12878 BHLHE40 NB100 IgG-mus ChIP-seq Signal from ENCODE/SYDH
GM78 BHL4 IgM
100
0
K562 BHLHE40 NB100 IgG-rab ChIP-seq Peaks from ENCODE/SYDH
K562 BHLHE40 NB100 IgG-rab ChIP-seq Signal from ENCODE/SYDH
K562 BHL4 IgR
150
0
A549 BHLHE40 IgG-rab ChIP-seq Peaks from ENCODE/SYDH
A549 BHLHE40 IgG-rab ChIP-seq Signal from ENCODE/SYDH
A549 BHL4 IgR
40
0
HEPG2 BHLHE40 NB100 IgG-rab ChIP-seq Peaks from ENCODE/SYDH
HEPG2 BHLHE40 NB100 IgG-rab ChIP-seq Signal from ENCODE/SYDH
HEPG BHL4 IgR
60
0
HepG2 BHLHE40 v041610.1 ChIP-seq Peaks Rep 1 from ENCODE/HAIB
HepG2 BHLHE40 v041610.1 ChIP-seq Raw Signal Rep 1 from ENCODE/HAIB
HepG BHLHE40 1
2
0
HepG2 BHLHE40 v041610.1 ChIP-seq Peaks Rep 2 from ENCODE/HAIB
HepG2 BHLHE40 v041610.1 ChIP-seq Raw Signal Rep 2 from ENCODE/HAIB
HepG BHLHE40 2
2
0

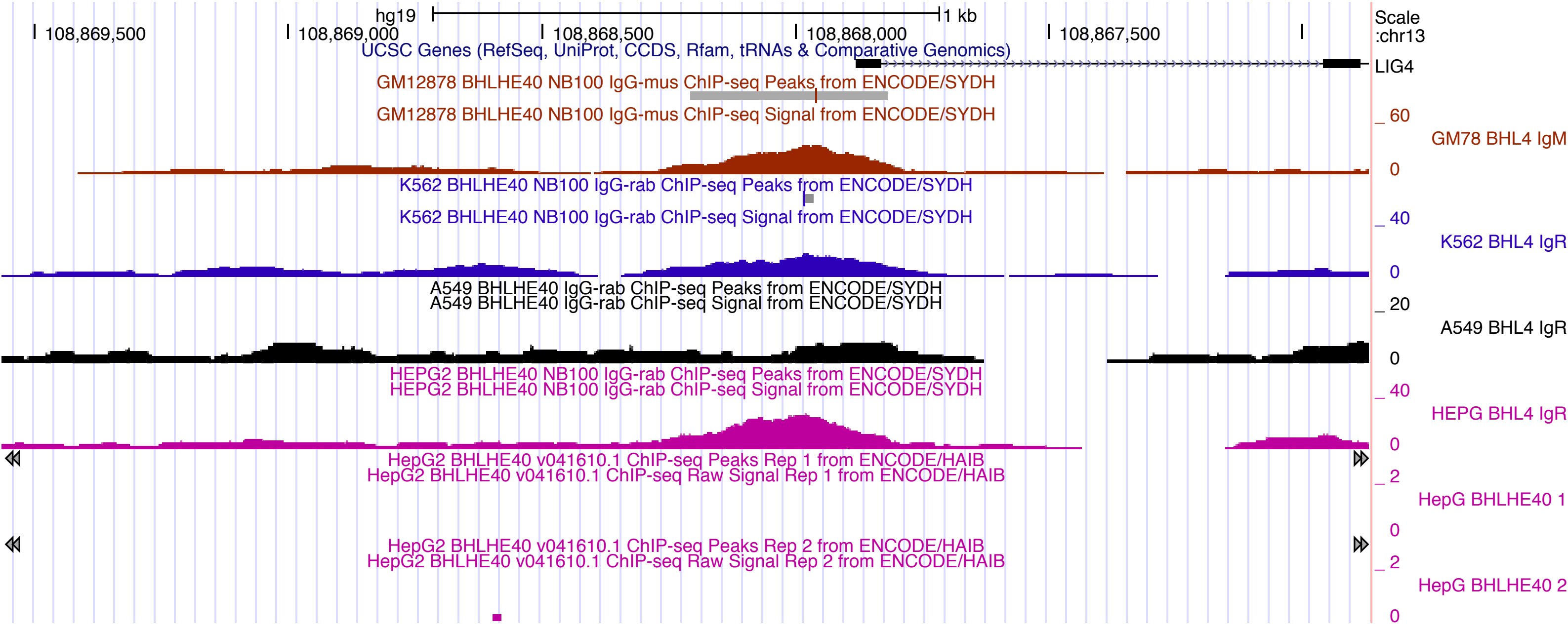

Scale
hg19
1 kb
chr13:
108,869,500
108,869,000
108,868,500
108,868,000
108,867,500
UCSC Genes (RefSeq, UniProt, CCDS, Rfam, tRNAs & Comparative Genomics)
LIG4
GM12878 BHLHE40 NB100 IgG-mus ChIP-seq Peaks from ENCODE/SYDH
GM12878 BHLHE40 NB100 IgG-mus ChIP-seq Signal from ENCODE/SYDH
GM78 BHL4 IgM
60
0
K562 BHLHE40 NB100 IgG-rab ChIP-seq Peaks from ENCODE/SYDH
K562 BHLHE40 NB100 IgG-rab ChIP-seq Signal from ENCODE/SYDH
K562 BHL4 IgR
40
0
A549 BHLHE40 IgG-rab ChIP-seq Peaks from ENCODE/SYDH
A549 BHLHE40 IgG-rab ChIP-seq Signal from ENCODE/SYDH
A549 BHL4 IgR
20
0
HEPG2 BHLHE40 NB100 IgG-rab ChIP-seq Peaks from ENCODE/SYDH
HEPG2 BHLHE40 NB100 IgG-rab ChIP-seq Signal from ENCODE/SYDH
HEPG BHL4 IgR
40
0
HepG2 BHLHE40 v041610.1 ChIP-seq Peaks Rep 1 from ENCODE/HAIB
HepG2 BHLHE40 v041610.1 ChIP-seq Raw Signal Rep 1 from ENCODE/HAIB
HepG BHLHE40 1
2
0
HepG2 BHLHE40 v041610.1 ChIP-seq Peaks Rep 2 from ENCODE/HAIB
HepG2 BHLHE40 v041610.1 ChIP-seq Raw Signal Rep 2 from ENCODE/HAIB
HepG BHLHE40 2
2
0

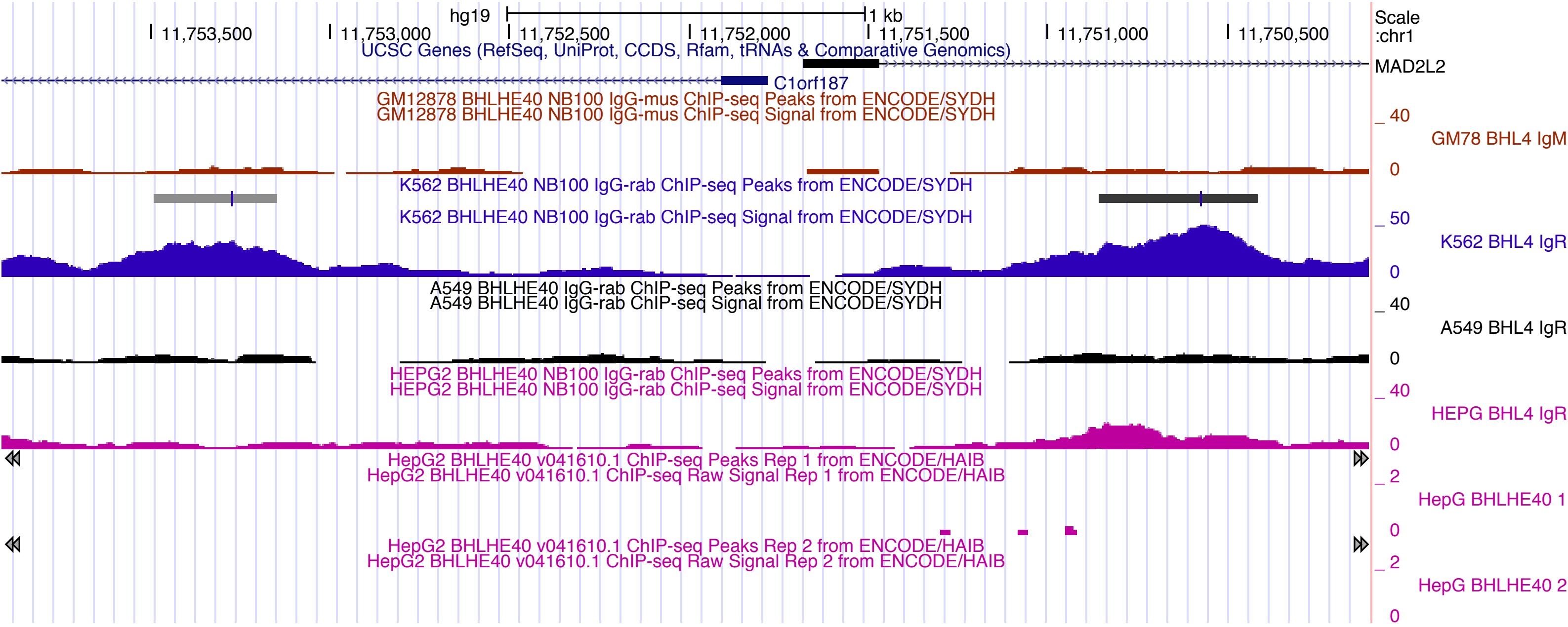

hg19
1 kb
Scale
:chr1
11,753,500
11,753,000
11,752,500
11,752,000
11,751,500
11,751,000
11,750,500
UCSC Genes (RefSeq, UniProt, CCDS, Rfam, tRNAs & Comparative Genomics)
MAD2L2
C1orf187
GM12878 BHLHE40 NB100 IgG-mus ChIP-seq Peaks from ENCODE/SYDH
GM12878 BHLHE40 NB100 IgG-mus ChIP-seq Signal from ENCODE/SYDH
GM78 BHL4 IgM
K562 BHLHE40 NB100 IgG-rab ChIP-seq Peaks from ENCODE/SYDH
K562 BHLHE40 NB100 IgG-rab ChIP-seq Signal from ENCODE/SYDH
K562 BHL4 IgR
A549 BHLHE40 IgG-rab ChIP-seq Peaks from ENCODE/SYDH
A549 BHLHE40 IgG-rab ChIP-seq Signal from ENCODE/SYDH
A549 BHL4 IgR
HEPG2 BHLHE40 NB100 IgG-rab ChIP-seq Peaks from ENCODE/SYDH
HEPG2 BHLHE40 NB100 IgG-rab ChIP-seq Signal from ENCODE/SYDH
HEPG BHL4 IgR
HepG2 BHLHE40 v041610.1 ChIP-seq Peaks Rep 1 from ENCODE/HAIB
HepG2 BHLHE40 v041610.1 ChIP-seq Raw Signal Rep 1 from ENCODE/HAIB
HepG BHLHE40 1
HepG2 BHLHE40 v041610.1 ChIP-seq Peaks Rep 2 from ENCODE/HAIB
HepG2 BHLHE40 v041610.1 ChIP-seq Raw Signal Rep 2 from ENCODE/HAIB
HepG BHLHE40 2
40
0
50
0
40
0
40
0
2
0
2
0

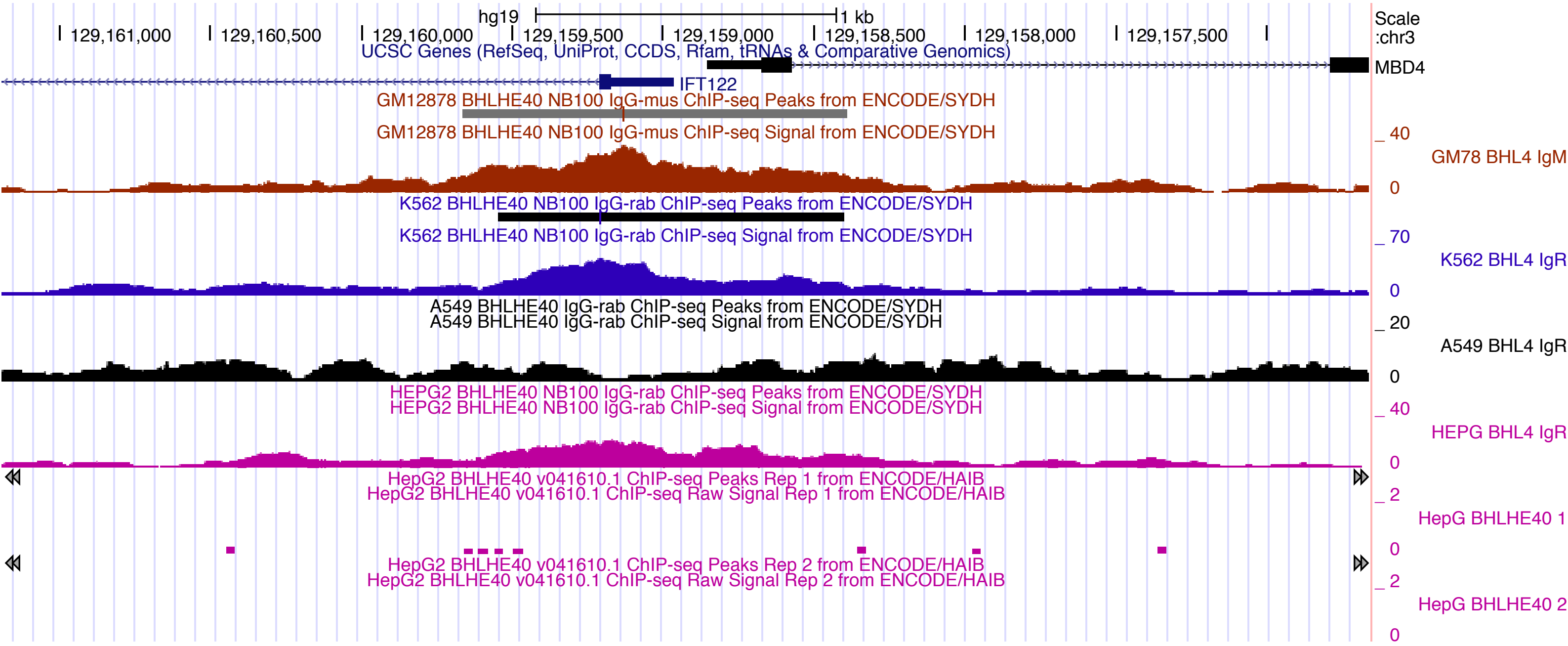
Scale
:chr3
hg19
1 kb
129,161,000
129,160,500
129,160,000
129,159,500
129,159,000
129,158,500
129,158,000
129,157,500
UCSC Genes (RefSeq, UniProt, CCDS, Rfam, tRNAs & Comparative Genomics)
MBD4
IFT122
GM12878 BHLHE40 NB100 IgG-mus ChIP-seq Peaks from ENCODE/SYDH
GM12878 BHLHE40 NB100 IgG-mus ChIP-seq Signal from ENCODE/SYDH
_ 40
GM78 BHL4 IgM
0
K562 BHLHE40 NB100 IgG-rab ChIP-seq Peaks from ENCODE/SYDH
K562 BHLHE40 NB100 IgG-rab ChIP-seq Signal from ENCODE/SYDH
_ 70
K562 BHL4 IgR
0
A549 BHLHE40 IgG-rab ChIP-seq Peaks from ENCODE/SYDH
A549 BHLHE40 IgG-rab ChIP-seq Signal from ENCODE/SYDH
_ 20
A549 BHL4 IgR
0
HEPG2 BHLHE40 NB100 IgG-rab ChIP-seq Peaks from ENCODE/SYDH
HEPG2 BHLHE40 NB100 IgG-rab ChIP-seq Signal from ENCODE/SYDH
_ 40
HEPG BHL4 IgR
0
HepG2 BHLHE40 v041610.1 ChIP-seq Peaks Rep 1 from ENCODE/HAIB
HepG2 BHLHE40 v041610.1 ChIP-seq Raw Signal Rep 1 from ENCODE/HAIB
_ 2
HepG BHLHE40 1
0
HepG2 BHLHE40 v041610.1 ChIP-seq Peaks Rep 2 from ENCODE/HAIB
HepG2 BHLHE40 v041610.1 ChIP-seq Raw Signal Rep 2 from ENCODE/HAIB
_ 2
HepG BHLHE40 2
0

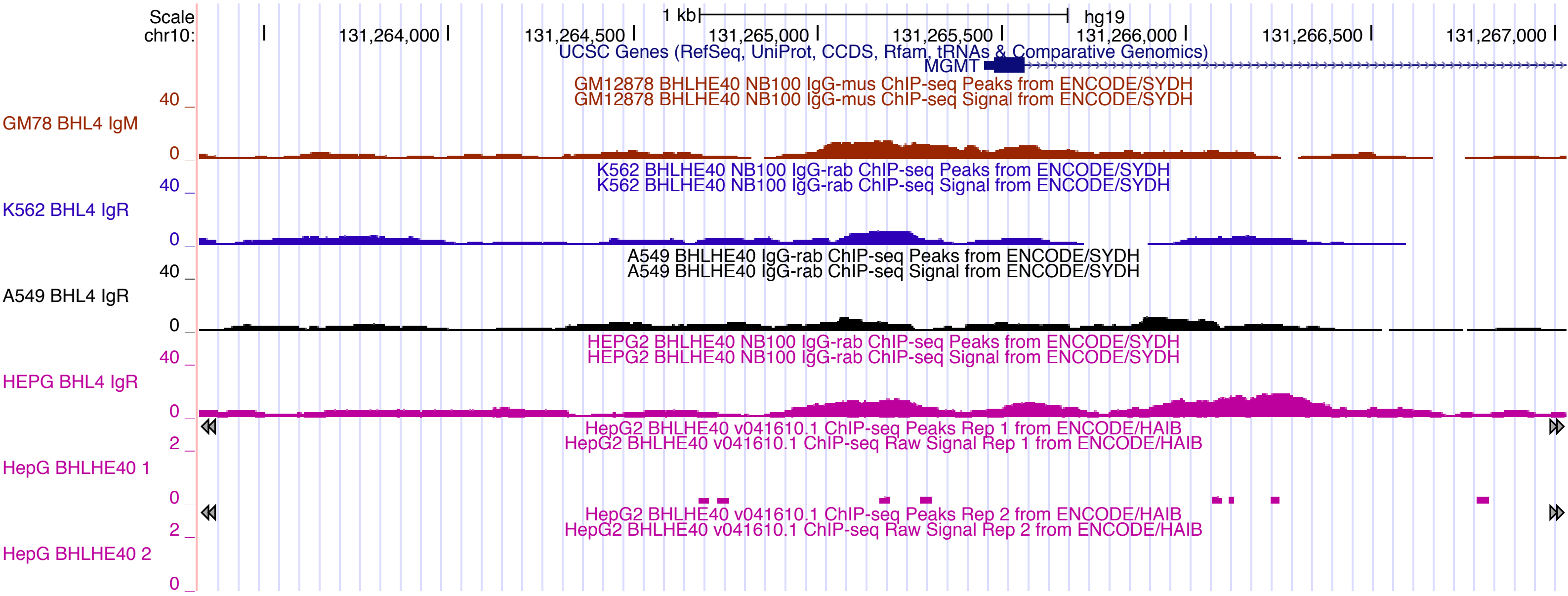

Scale
chr10:
1 kb
hg19
131,264,000
131,264,500
131,265,000
131,265,500
131,266,000
131,266,500
131,267,000
UCSC Genes (RefSeq, UniProt, CCDS, Rfam, tRNAs & Comparative Genomics)
MGMT
GM12878 BHLHE40 NB100 IgG-mus ChIP-seq Peaks from ENCODE/SYDH
GM12878 BHLHE40 NB100 IgG-mus ChIP-seq Signal from ENCODE/SYDH
GM78 BHL4 IgM
K562 BHLHE40 NB100 IgG-rab ChIP-seq Peaks from ENCODE/SYDH
K562 BHLHE40 NB100 IgG-rab ChIP-seq Signal from ENCODE/SYDH
K562 BHL4 IgR
A549 BHLHE40 IgG-rab ChIP-seq Peaks from ENCODE/SYDH
A549 BHLHE40 IgG-rab ChIP-seq Signal from ENCODE/SYDH
A549 BHL4 IgR
HEPG2 BHLHE40 NB100 IgG-rab ChIP-seq Peaks from ENCODE/SYDH
HEPG2 BHLHE40 NB100 IgG-rab ChIP-seq Signal from ENCODE/SYDH
HEPG BHL4 IgR
HepG2 BHLHE40 v041610.1 ChIP-seq Peaks Rep 1 from ENCODE/HAIB
HepG2 BHLHE40 v041610.1 ChIP-seq Raw Signal Rep 1 from ENCODE/HAIB
HepG BHLHE40 1
HepG2 BHLHE40 v041610.1 ChIP-seq Peaks Rep 2 from ENCODE/HAIB
HepG2 BHLHE40 v041610.1 ChIP-seq Raw Signal Rep 2 from ENCODE/HAIB
HepG BHLHE40 2
40
0
2

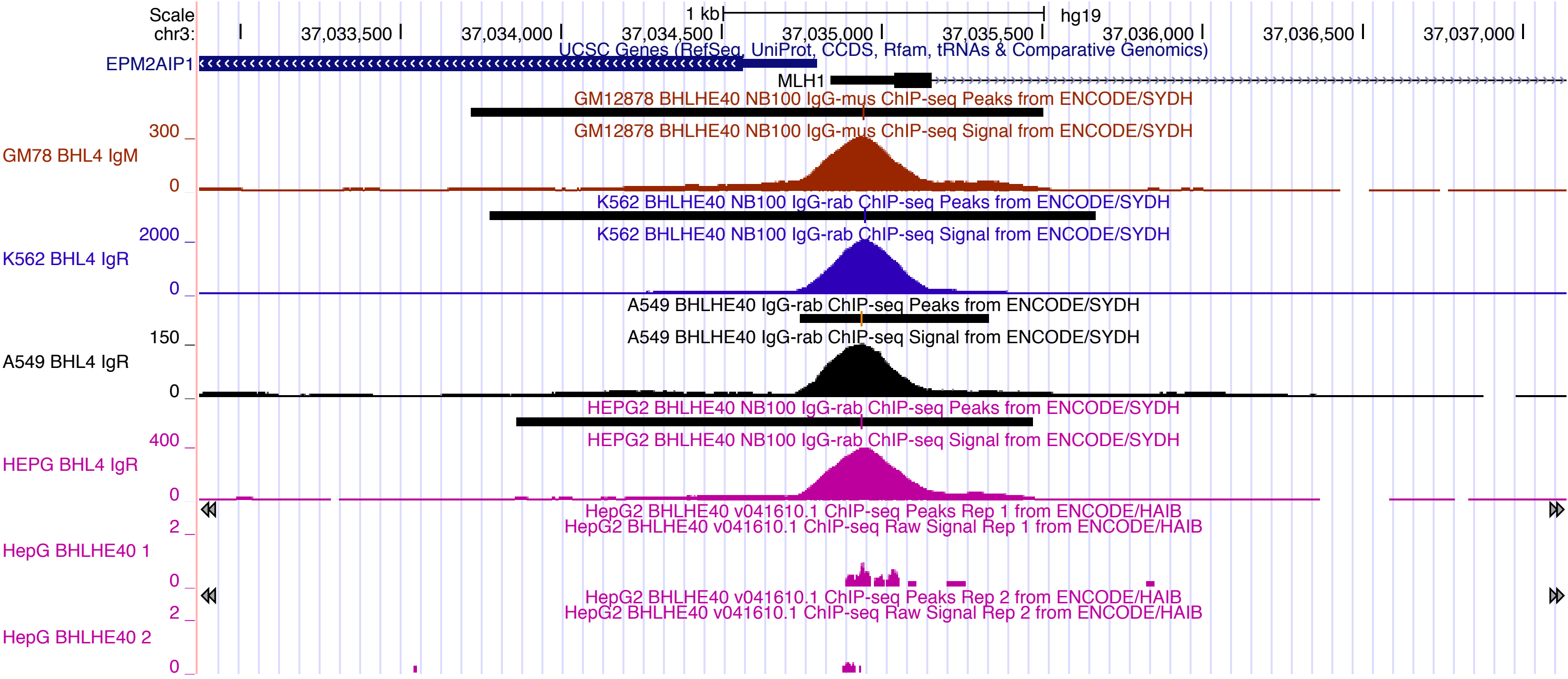

Scale
chr3:
1 kb
hg19
37,033,500
37,034,000
37,034,500
37,035,000
37,035,500
37,036,000
37,036,500
37,037,000
UCSC Genes (RefSeq, UniProt, CCDS, Rfam, tRNAs & Comparative Genomics)
EPM2AIP1
MLH1
GM12878 BHLHE40 NB100 IgG-mus ChIP-seq Peaks from ENCODE/SYDH
GM12878 BHLHE40 NB100 IgG-mus ChIP-seq Signal from ENCODE/SYDH
GM78 BHL4 IgM
300
0
K562 BHLHE40 NB100 IgG-rab ChIP-seq Peaks from ENCODE/SYDH
K562 BHLHE40 NB100 IgG-rab ChIP-seq Signal from ENCODE/SYDH
K562 BHL4 IgR
2000
0
A549 BHLHE40 IgG-rab ChIP-seq Peaks from ENCODE/SYDH
A549 BHLHE40 IgG-rab ChIP-seq Signal from ENCODE/SYDH
A549 BHL4 IgR
150
0
HEPG2 BHLHE40 NB100 IgG-rab ChIP-seq Peaks from ENCODE/SYDH
HEPG2 BHLHE40 NB100 IgG-rab ChIP-seq Signal from ENCODE/SYDH
HEPG BHL4 IgR
400
0
HepG2 BHLHE40 v041610.1 ChIP-seq Peaks Rep 1 from ENCODE/HAIB
HepG2 BHLHE40 v041610.1 ChIP-seq Raw Signal Rep 1 from ENCODE/HAIB
HepG BHLHE40 1
2
0
HepG2 BHLHE40 v041610.1 ChIP-seq Peaks Rep 2 from ENCODE/HAIB
HepG2 BHLHE40 v041610.1 ChIP-seq Raw Signal Rep 2 from ENCODE/HAIB
HepG BHLHE40 2
2
0

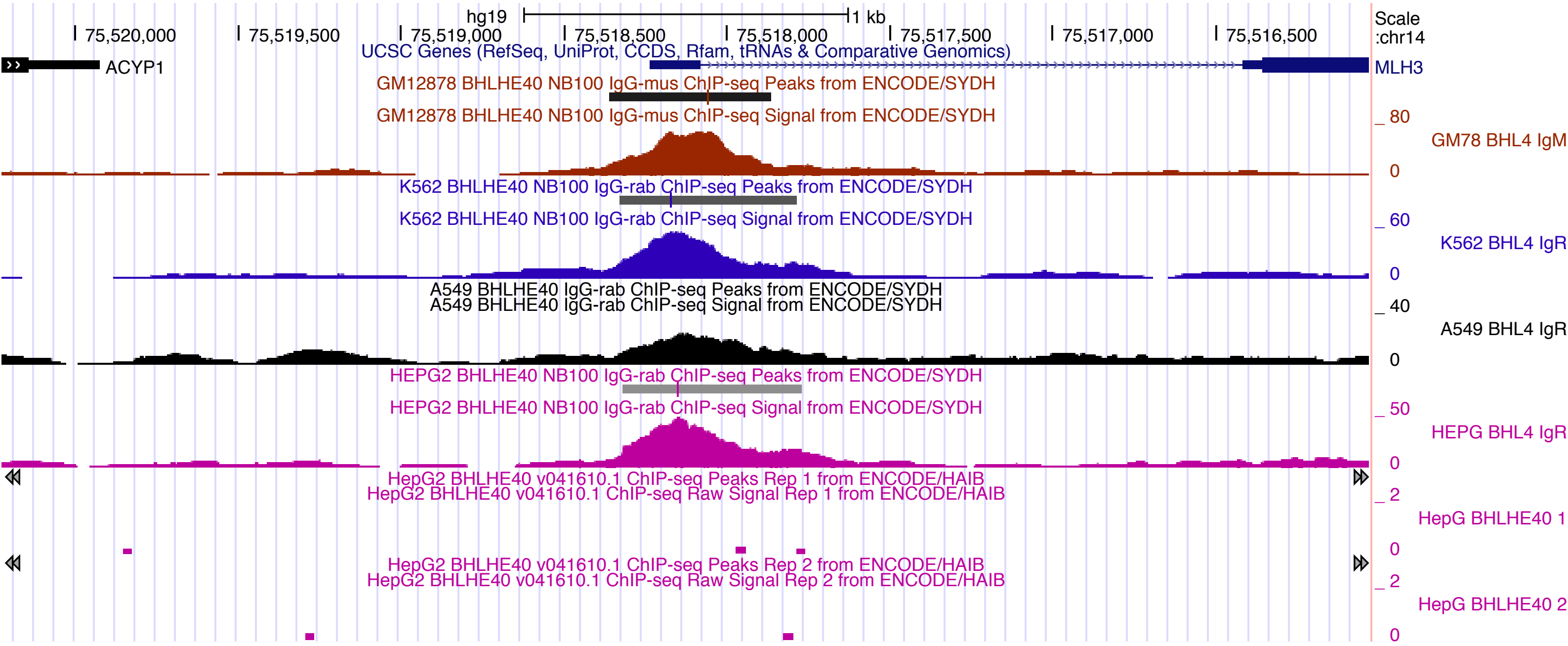

hg19
1 kb
Scale
:chr14
75,520,000
75,519,500
75,519,000
75,518,500
75,518,000
75,517,500
75,517,000
75,516,500
UCSC Genes (RefSeq, UniProt, CCDS, Rfam, tRNAs & Comparative Genomics)
ACYP1
MLH3
GM12878 BHLHE40 NB100 IgG-mus ChIP-seq Peaks from ENCODE/SYDH
GM12878 BHLHE40 NB100 IgG-mus ChIP-seq Signal from ENCODE/SYDH
80
GM78 BHL4 IgM
0
K562 BHLHE40 NB100 IgG-rab ChIP-seq Peaks from ENCODE/SYDH
K562 BHLHE40 NB100 IgG-rab ChIP-seq Signal from ENCODE/SYDH
60
K562 BHL4 IgR
0
A549 BHLHE40 IgG-rab ChIP-seq Peaks from ENCODE/SYDH
A549 BHLHE40 IgG-rab ChIP-seq Signal from ENCODE/SYDH
40
A549 BHL4 IgR
0
HEPG2 BHLHE40 NB100 IgG-rab ChIP-seq Peaks from ENCODE/SYDH
HEPG2 BHLHE40 NB100 IgG-rab ChIP-seq Signal from ENCODE/SYDH
50
HEPG BHL4 IgR
0
HepG2 BHLHE40 v041610.1 ChIP-seq Peaks Rep 1 from ENCODE/HAIB
HepG2 BHLHE40 v041610.1 ChIP-seq Raw Signal Rep 1 from ENCODE/HAIB
2
HepG BHLHE40 1
0
HepG2 BHLHE40 v041610.1 ChIP-seq Peaks Rep 2 from ENCODE/HAIB
HepG2 BHLHE40 v041610.1 ChIP-seq Raw Signal Rep 2 from ENCODE/HAIB
2
HepG BHLHE40 2
0

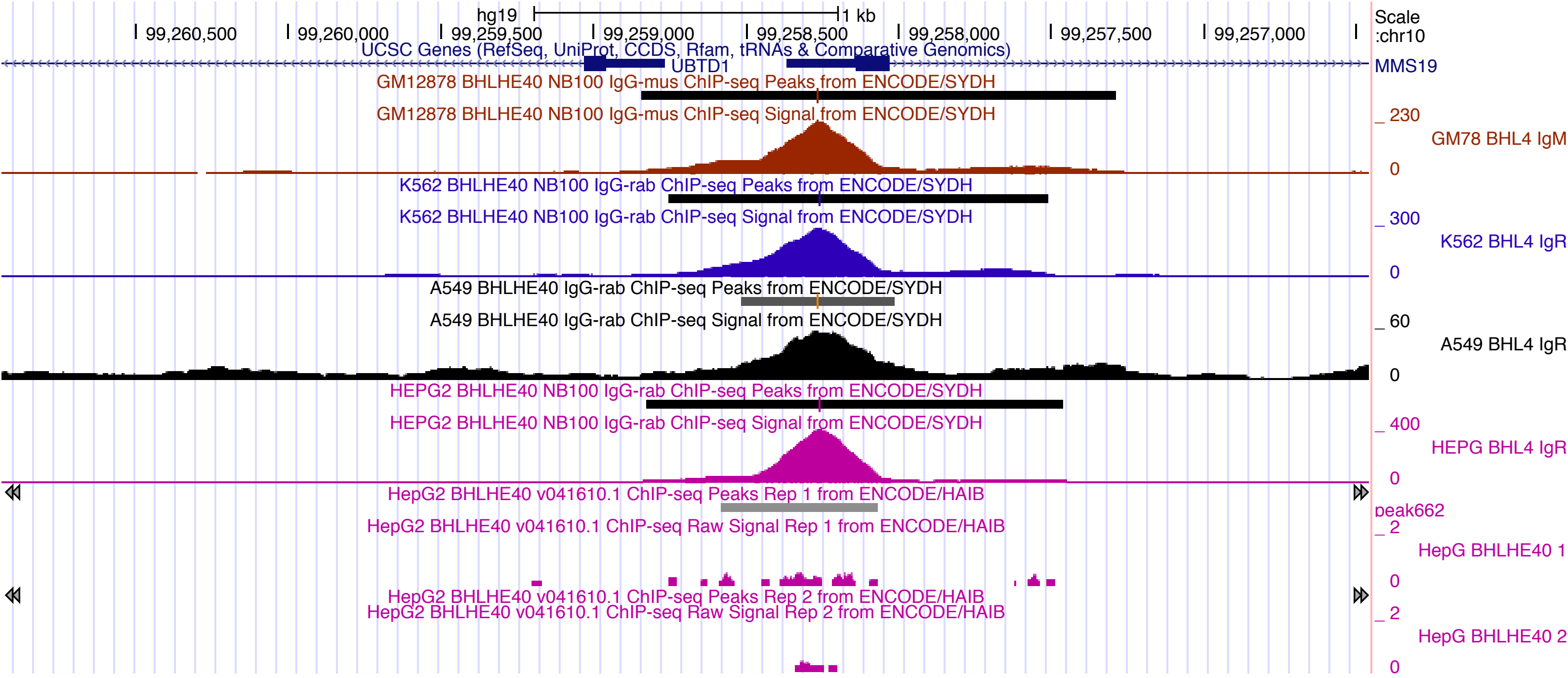

hg19
1 kb
Scale
:chr10
99,260,500
99,260,000
99,259,500
99,259,000
99,258,500
99,258,000
99,257,500
99,257,000
UCSC Genes (RefSeq, UniProt, CCDS, Rfam, tRNAs & Comparative Genomics)
UBTD1
MMS19
GM12878 BHLHE40 NB100 IgG-mus ChIP-seq Peaks from ENCODE/SYDH
GM12878 BHLHE40 NB100 IgG-mus ChIP-seq Signal from ENCODE/SYDH
_ 230
GM78 BHL4 IgM
0
K562 BHLHE40 NB100 IgG-rab ChIP-seq Peaks from ENCODE/SYDH
K562 BHLHE40 NB100 IgG-rab ChIP-seq Signal from ENCODE/SYDH
_ 300
K562 BHL4 IgR
0
A549 BHLHE40 IgG-rab ChIP-seq Peaks from ENCODE/SYDH
A549 BHLHE40 IgG-rab ChIP-seq Signal from ENCODE/SYDH
_ 60
A549 BHL4 IgR
0
HEPG2 BHLHE40 NB100 IgG-rab ChIP-seq Peaks from ENCODE/SYDH
HEPG2 BHLHE40 NB100 IgG-rab ChIP-seq Signal from ENCODE/SYDH
_ 400
HEPG BHL4 IgR
0
HepG2 BHLHE40 v041610.1 ChIP-seq Peaks Rep 1 from ENCODE/HAIB
peak662
HepG2 BHLHE40 v041610.1 ChIP-seq Raw Signal Rep 1 from ENCODE/HAIB
_ 2
HepG BHLHE40 1
0
HepG2 BHLHE40 v041610.1 ChIP-seq Peaks Rep 2 from ENCODE/HAIB
HepG2 BHLHE40 v041610.1 ChIP-seq Raw Signal Rep 2 from ENCODE/HAIB
_ 2
HepG BHLHE40 2
0

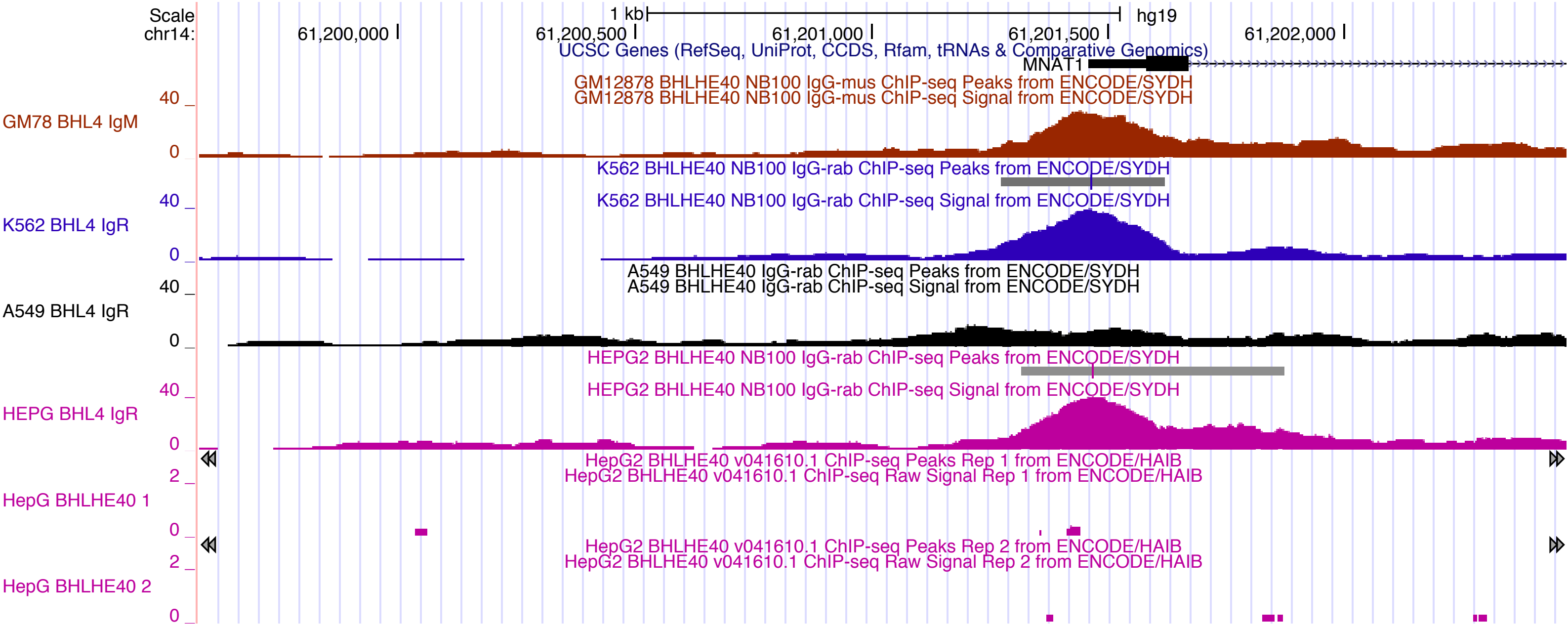
Scale
chr14:
1 kb
hg19
61,200,000
61,200,500
61,201,000
61,201,500
61,202,000
UCSC Genes (RefSeq, UniProt, CCDS, Rfam, tRNAs & Comparative Genomics)
MNAT1
GM12878 BHLHE40 NB100 IgG-mus ChIP-seq Peaks from ENCODE/SYDH
GM12878 BHLHE40 NB100 IgG-mus ChIP-seq Signal from ENCODE/SYDH
GM78 BHL4 IgM
K562 BHLHE40 NB100 IgG-rab ChIP-seq Peaks from ENCODE/SYDH
K562 BHLHE40 NB100 IgG-rab ChIP-seq Signal from ENCODE/SYDH
K562 BHL4 IgR
A549 BHLHE40 IgG-rab ChIP-seq Peaks from ENCODE/SYDH
A549 BHLHE40 IgG-rab ChIP-seq Signal from ENCODE/SYDH
A549 BHL4 IgR
HEPG2 BHLHE40 NB100 IgG-rab ChIP-seq Peaks from ENCODE/SYDH
HEPG2 BHLHE40 NB100 IgG-rab ChIP-seq Signal from ENCODE/SYDH
HEPG BHL4 IgR
HepG2 BHLHE40 v041610.1 ChIP-seq Peaks Rep 1 from ENCODE/HAIB
HepG2 BHLHE40 v041610.1 ChIP-seq Raw Signal Rep 1 from ENCODE/HAIB
HepG BHLHE40 1
HepG2 BHLHE40 v041610.1 ChIP-seq Peaks Rep 2 from ENCODE/HAIB
HepG2 BHLHE40 v041610.1 ChIP-seq Raw Signal Rep 2 from ENCODE/HAIB
HepG BHLHE40 2
40
0
2

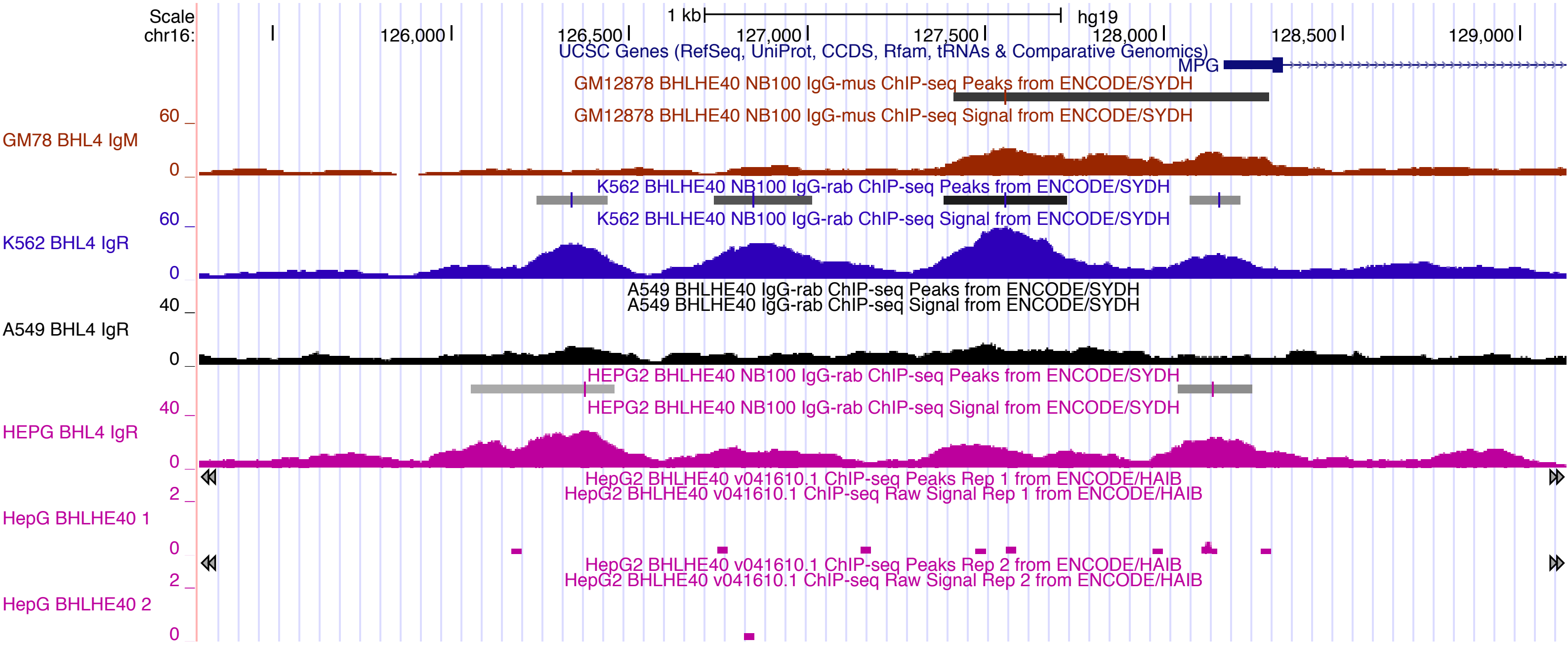
Scale
chr16:
1 kb
hg19
126,000
126,500
127,000
127,500
128,000
128,500
129,000
UCSC Genes (RefSeq, UniProt, CCDS, Rfam, tRNAs & Comparative Genomics)
MPG
GM12878 BHLHE40 NB100 IgG-mus ChIP-seq Peaks from ENCODE/SYDH
GM12878 BHLHE40 NB100 IgG-mus ChIP-seq Signal from ENCODE/SYDH
GM78 BHL4 IgM
K562 BHLHE40 NB100 IgG-rab ChIP-seq Peaks from ENCODE/SYDH
K562 BHLHE40 NB100 IgG-rab ChIP-seq Signal from ENCODE/SYDH
K562 BHL4 IgR
A549 BHLHE40 IgG-rab ChIP-seq Peaks from ENCODE/SYDH
A549 BHLHE40 IgG-rab ChIP-seq Signal from ENCODE/SYDH
A549 BHL4 IgR
HEPG2 BHLHE40 NB100 IgG-rab ChIP-seq Peaks from ENCODE/SYDH
HEPG2 BHLHE40 NB100 IgG-rab ChIP-seq Signal from ENCODE/SYDH
HEPG BHL4 IgR
HepG2 BHLHE40 v041610.1 ChIP-seq Peaks Rep 1 from ENCODE/HAIB
HepG2 BHLHE40 v041610.1 ChIP-seq Raw Signal Rep 1 from ENCODE/HAIB
HepG BHLHE40 1
HepG2 BHLHE40 v041610.1 ChIP-seq Peaks Rep 2 from ENCODE/HAIB
HepG2 BHLHE40 v041610.1 ChIP-seq Raw Signal Rep 2 from ENCODE/HAIB
HepG BHLHE40 2

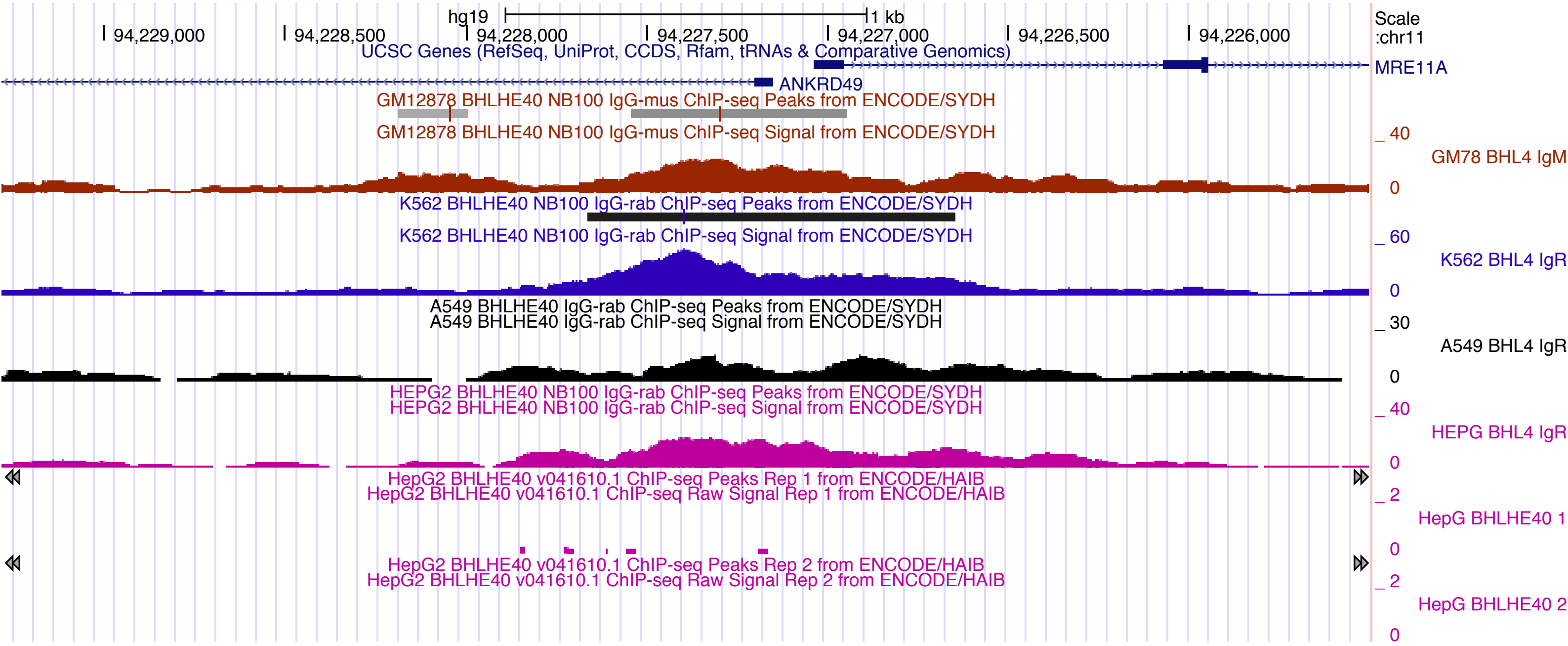

hg19
1 kb
Scale
:chr11
94,229,000
94,228,500
94,228,000
94,227,500
94,227,000
94,226,500
94,226,000
UCSC Genes (RefSeq, UniProt, CCDS, Rfam, tRNAs & Comparative Genomics)
MRE11A
ANKRD49
GM12878 BHLHE40 NB100 IgG-mus ChIP-seq Peaks from ENCODE/SYDH
GM12878 BHLHE40 NB100 IgG-mus ChIP-seq Signal from ENCODE/SYDH
_ 40
GM78 BHL4 IgM
0
K562 BHLHE40 NB100 IgG-rab ChIP-seq Peaks from ENCODE/SYDH
K562 BHLHE40 NB100 IgG-rab ChIP-seq Signal from ENCODE/SYDH
_ 60
K562 BHL4 IgR
0
A549 BHLHE40 IgG-rab ChIP-seq Peaks from ENCODE/SYDH
A549 BHLHE40 IgG-rab ChIP-seq Signal from ENCODE/SYDH
_ 30
A549 BHL4 IgR
0
HEPG2 BHLHE40 NB100 IgG-rab ChIP-seq Peaks from ENCODE/SYDH
HEPG2 BHLHE40 NB100 IgG-rab ChIP-seq Signal from ENCODE/SYDH
_ 40
HEPG BHL4 IgR
0
HepG2 BHLHE40 v041610.1 ChIP-seq Peaks Rep 1 from ENCODE/HAIB
HepG2 BHLHE40 v041610.1 ChIP-seq Raw Signal Rep 1 from ENCODE/HAIB
_ 2
HepG BHLHE40 1
0
HepG2 BHLHE40 v041610.1 ChIP-seq Peaks Rep 2 from ENCODE/HAIB
HepG2 BHLHE40 v041610.1 ChIP-seq Raw Signal Rep 2 from ENCODE/HAIB
_ 2
HepG BHLHE40 2
0

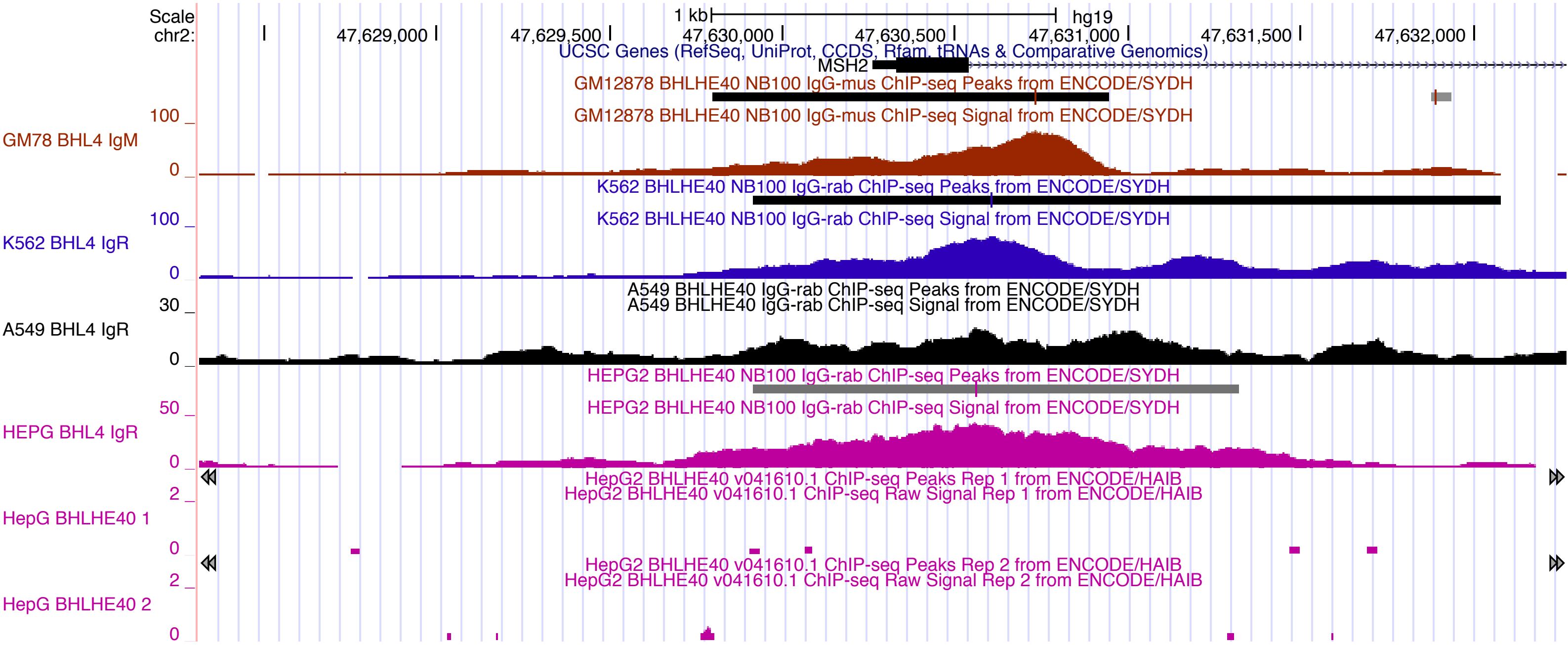

Scale
chr2:
1 kb
hg19
47,629,000
47,629,500
47,630,000
47,630,500
47,631,000
47,631,500
47,632,000
UCSC Genes (RefSeq, UniProt, CCDS, Rfam, tRNAs & Comparative Genomics)
MSH2
GM12878 BHLHE40 NB100 IgG-mus ChIP-seq Peaks from ENCODE/SYDH
GM12878 BHLHE40 NB100 IgG-mus ChIP-seq Signal from ENCODE/SYDH
GM78 BHL4 IgM
100
0
K562 BHLHE40 NB100 IgG-rab ChIP-seq Peaks from ENCODE/SYDH
K562 BHLHE40 NB100 IgG-rab ChIP-seq Signal from ENCODE/SYDH
K562 BHL4 IgR
A549 BHLHE40 IgG-rab ChIP-seq Peaks from ENCODE/SYDH
A549 BHLHE40 IgG-rab ChIP-seq Signal from ENCODE/SYDH
A549 BHL4 IgR
30
HEPG2 BHLHE40 NB100 IgG-rab ChIP-seq Peaks from ENCODE/SYDH
HEPG2 BHLHE40 NB100 IgG-rab ChIP-seq Signal from ENCODE/SYDH
HEPG BHL4 IgR
50
HepG2 BHLHE40 v041610.1 ChIP-seq Peaks Rep 1 from ENCODE/HAIB
HepG2 BHLHE40 v041610.1 ChIP-seq Raw Signal Rep 1 from ENCODE/HAIB
HepG BHLHE40 1
2
HepG2 BHLHE40 v041610.1 ChIP-seq Peaks Rep 2 from ENCODE/HAIB
HepG2 BHLHE40 v041610.1 ChIP-seq Raw Signal Rep 2 from ENCODE/HAIB
HepG BHLHE40 2

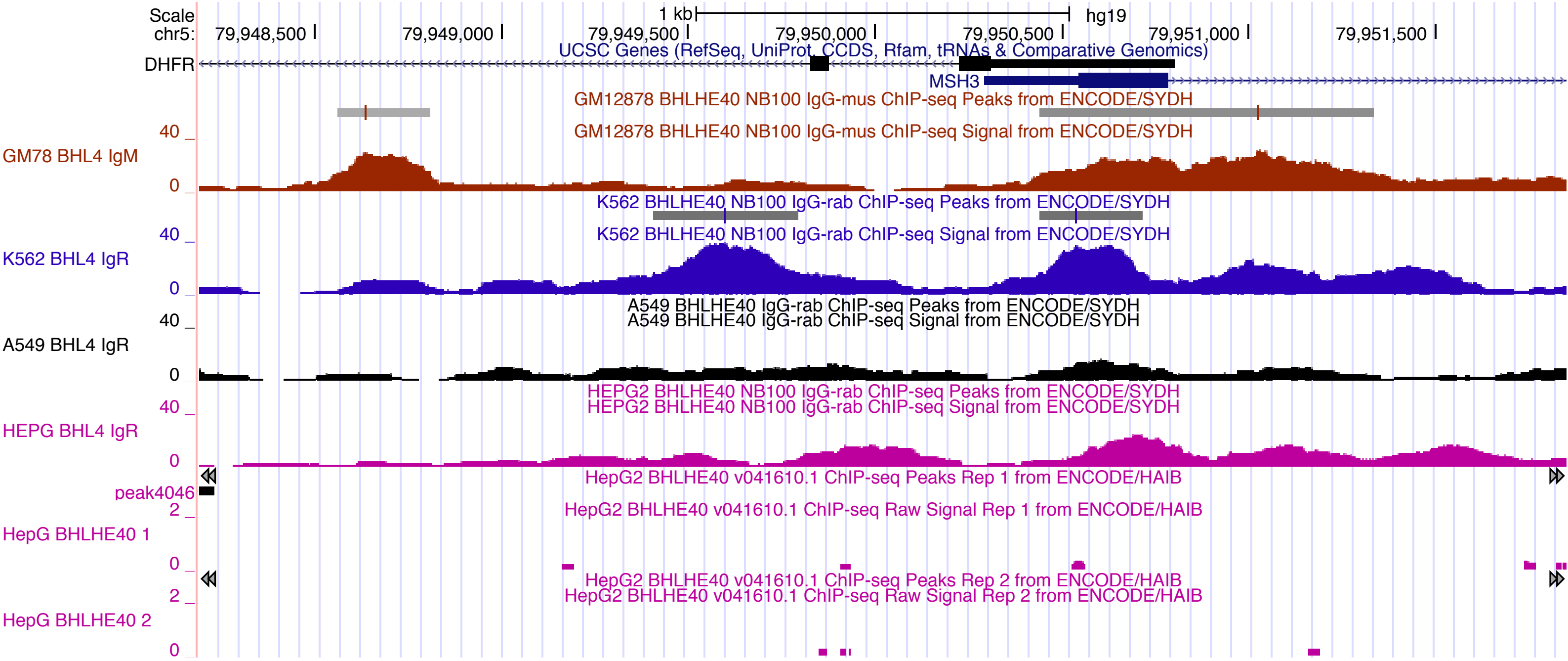
Scale
chr5:
1 kb
hg19
79,948,500
79,949,000
79,949,500
79,950,000
79,950,500
79,951,000
79,951,500
UCSC Genes (RefSeq, UniProt, CCDS, Rfam, tRNAs & Comparative Genomics)
DHFR
MSH3
GM12878 BHLHE40 NB100 IgG-mus ChIP-seq Peaks from ENCODE/SYDH
GM12878 BHLHE40 NB100 IgG-mus ChIP-seq Signal from ENCODE/SYDH
GM78 BHL4 IgM
K562 BHLHE40 NB100 IgG-rab ChIP-seq Peaks from ENCODE/SYDH
K562 BHLHE40 NB100 IgG-rab ChIP-seq Signal from ENCODE/SYDH
K562 BHL4 IgR
A549 BHLHE40 IgG-rab ChIP-seq Peaks from ENCODE/SYDH
A549 BHLHE40 IgG-rab ChIP-seq Signal from ENCODE/SYDH
A549 BHL4 IgR
HEPG2 BHLHE40 NB100 IgG-rab ChIP-seq Peaks from ENCODE/SYDH
HEPG2 BHLHE40 NB100 IgG-rab ChIP-seq Signal from ENCODE/SYDH
HEPG BHL4 IgR
HepG2 BHLHE40 v041610.1 ChIP-seq Peaks Rep 1 from ENCODE/HAIB
peak4046
HepG2 BHLHE40 v041610.1 ChIP-seq Raw Signal Rep 1 from ENCODE/HAIB
HepG BHLHE40 1
HepG2 BHLHE40 v041610.1 ChIP-seq Peaks Rep 2 from ENCODE/HAIB
HepG2 BHLHE40 v041610.1 ChIP-seq Raw Signal Rep 2 from ENCODE/HAIB
HepG BHLHE40 2
40
0
2

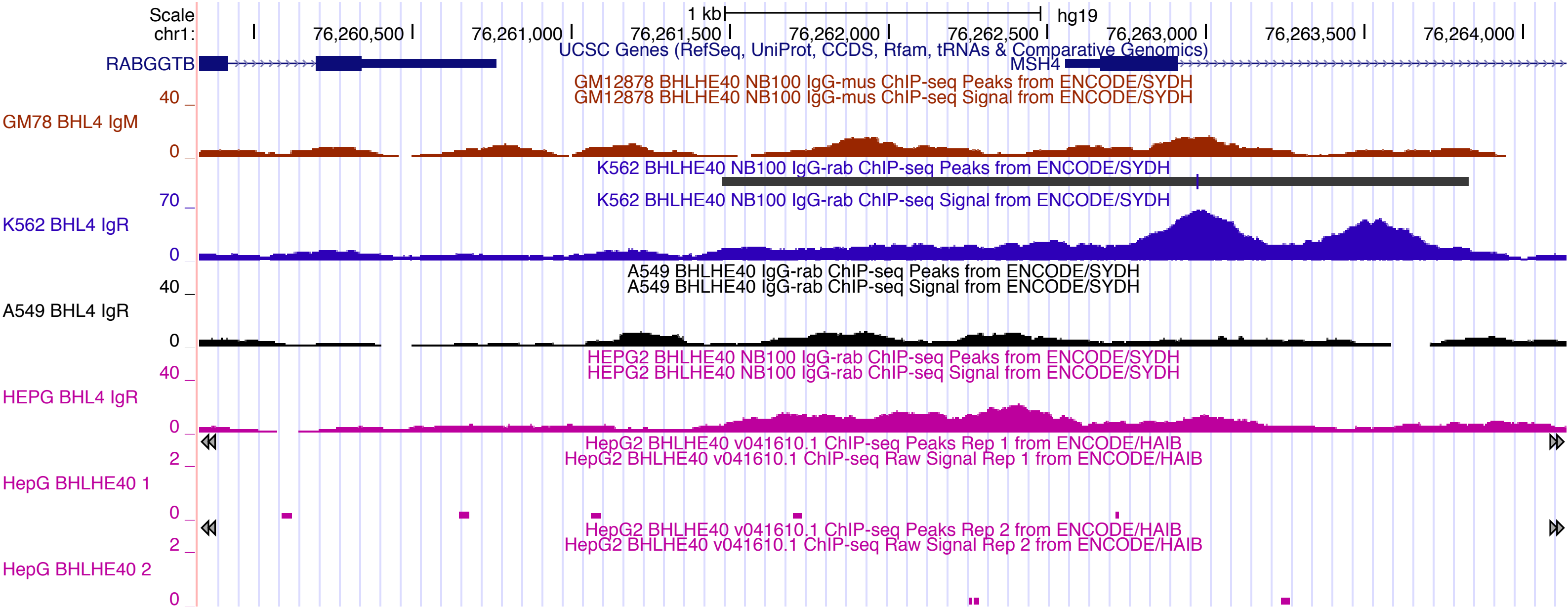
Scale
chr1:
1 kb
hg19
76,260,500
76,261,000
76,261,500
76,262,000
76,262,500
76,263,000
76,263,500
76,264,000
UCSC Genes (RefSeq, UniProt, CCDS, Rfam, tRNAs & Comparative Genomics)
RABGGTB
MSH4
GM12878 BHLHE40 NB100 IgG-mus ChIP-seq Peaks from ENCODE/SYDH
GM12878 BHLHE40 NB100 IgG-mus ChIP-seq Signal from ENCODE/SYDH
GM78 BHL4 IgM
40
0
K562 BHLHE40 NB100 IgG-rab ChIP-seq Peaks from ENCODE/SYDH
K562 BHLHE40 NB100 IgG-rab ChIP-seq Signal from ENCODE/SYDH
K562 BHL4 IgR
70
0
A549 BHLHE40 IgG-rab ChIP-seq Peaks from ENCODE/SYDH
A549 BHLHE40 IgG-rab ChIP-seq Signal from ENCODE/SYDH
A549 BHL4 IgR
40
0
HEPG2 BHLHE40 NB100 IgG-rab ChIP-seq Peaks from ENCODE/SYDH
HEPG2 BHLHE40 NB100 IgG-rab ChIP-seq Signal from ENCODE/SYDH
HEPG BHL4 IgR
40
0
HepG2 BHLHE40 v041610.1 ChIP-seq Peaks Rep 1 from ENCODE/HAIB
HepG2 BHLHE40 v041610.1 ChIP-seq Raw Signal Rep 1 from ENCODE/HAIB
HepG BHLHE40 1
2
0
HepG2 BHLHE40 v041610.1 ChIP-seq Peaks Rep 2 from ENCODE/HAIB
HepG2 BHLHE40 v041610.1 ChIP-seq Raw Signal Rep 2 from ENCODE/HAIB
HepG BHLHE40 2
2
0

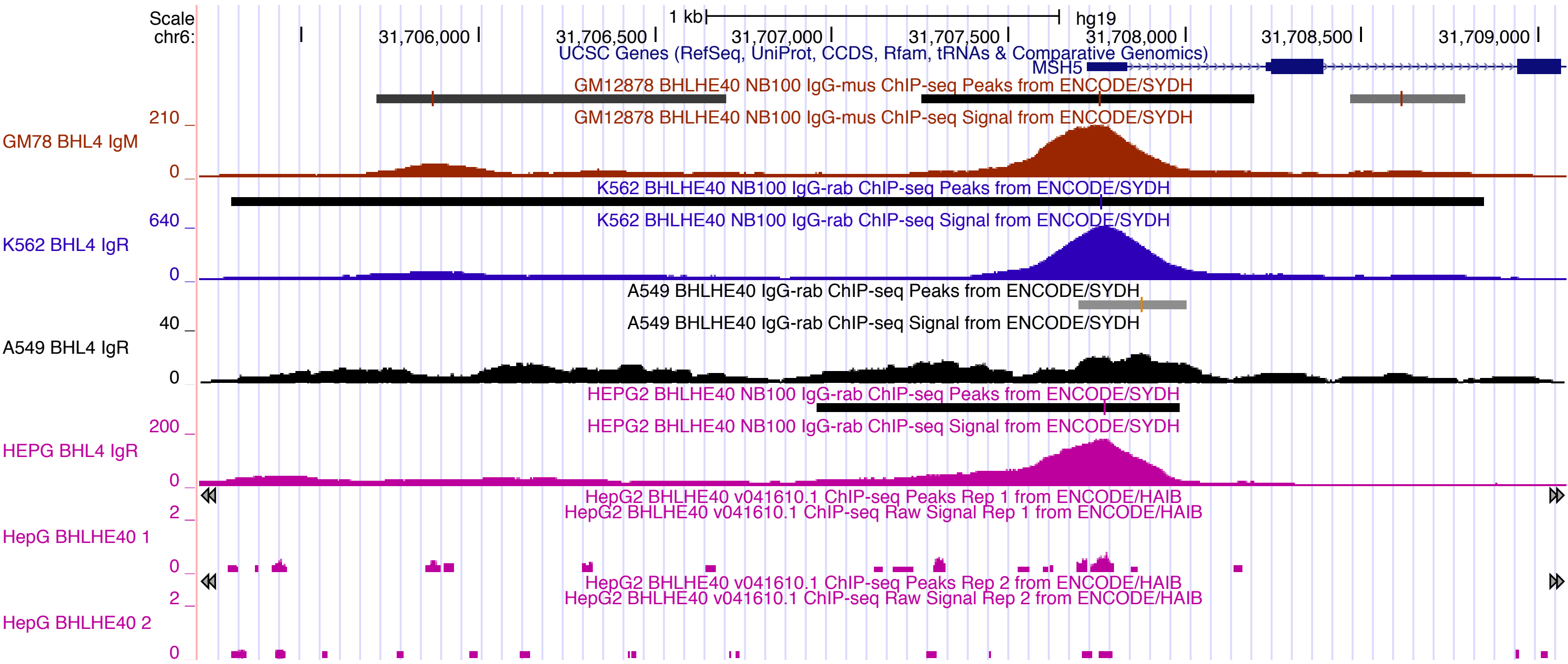

Scale
chr6:
1 kb
hg19
31,706,000
31,706,500
31,707,000
31,707,500
31,708,000
31,708,500
31,709,000
UCSC Genes (RefSeq, UniProt, CCDS, Rfam, tRNAs & Comparative Genomics)
MSH5
GM12878 BHLHE40 NB100 IgG-mus ChIP-seq Peaks from ENCODE/SYDH
GM12878 BHLHE40 NB100 IgG-mus ChIP-seq Signal from ENCODE/SYDH
GM78 BHL4 IgM
210
0
K562 BHLHE40 NB100 IgG-rab ChIP-seq Peaks from ENCODE/SYDH
K562 BHLHE40 NB100 IgG-rab ChIP-seq Signal from ENCODE/SYDH
K562 BHL4 IgR
640
0
A549 BHLHE40 IgG-rab ChIP-seq Peaks from ENCODE/SYDH
A549 BHLHE40 IgG-rab ChIP-seq Signal from ENCODE/SYDH
A549 BHL4 IgR
40
0
HEPG2 BHLHE40 NB100 IgG-rab ChIP-seq Peaks from ENCODE/SYDH
HEPG2 BHLHE40 NB100 IgG-rab ChIP-seq Signal from ENCODE/SYDH
HEPG BHL4 IgR
200
0
HepG2 BHLHE40 v041610.1 ChIP-seq Peaks Rep 1 from ENCODE/HAIB
HepG2 BHLHE40 v041610.1 ChIP-seq Raw Signal Rep 1 from ENCODE/HAIB
HepG BHLHE40 1
2
0
HepG2 BHLHE40 v041610.1 ChIP-seq Peaks Rep 2 from ENCODE/HAIB
HepG2 BHLHE40 v041610.1 ChIP-seq Raw Signal Rep 2 from ENCODE/HAIB
HepG BHLHE40 2
2
0

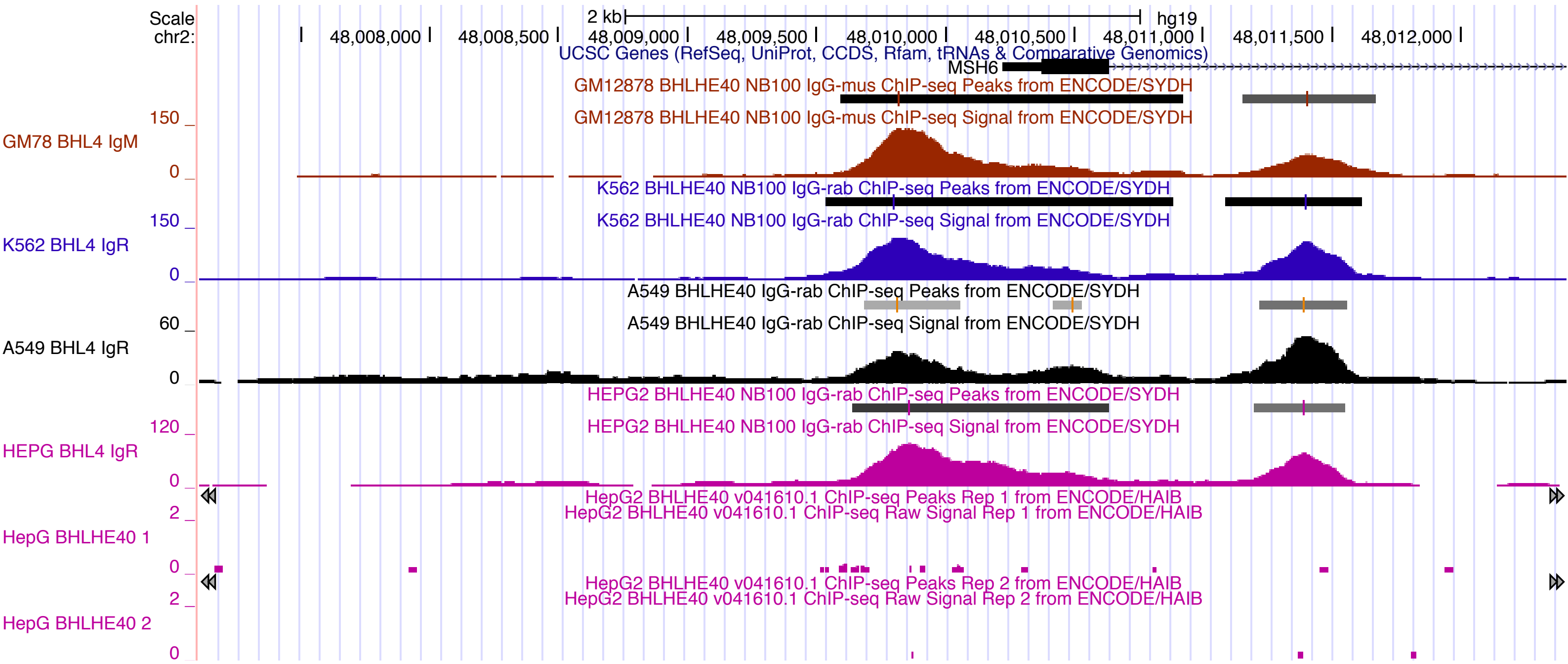

Scale
chr2:
2 kb
hg19
48,008,000
48,008,500
48,009,000
48,009,500
48,010,000
48,010,500
48,011,000
48,011,500
48,012,000
UCSC Genes (RefSeq, UniProt, CCDS, Rfam, tRNAs & Comparative Genomics)
MSH6
GM12878 BHLHE40 NB100 IgG-mus ChIP-seq Peaks from ENCODE/SYDH
GM12878 BHLHE40 NB100 IgG-mus ChIP-seq Signal from ENCODE/SYDH
GM78 BHL4 IgM
150
0
K562 BHLHE40 NB100 IgG-rab ChIP-seq Peaks from ENCODE/SYDH
K562 BHLHE40 NB100 IgG-rab ChIP-seq Signal from ENCODE/SYDH
K562 BHL4 IgR
150
0
A549 BHLHE40 IgG-rab ChIP-seq Peaks from ENCODE/SYDH
A549 BHLHE40 IgG-rab ChIP-seq Signal from ENCODE/SYDH
A549 BHL4 IgR
60
0
HEPG2 BHLHE40 NB100 IgG-rab ChIP-seq Peaks from ENCODE/SYDH
HEPG2 BHLHE40 NB100 IgG-rab ChIP-seq Signal from ENCODE/SYDH
HEPG BHL4 IgR
120
0
HepG2 BHLHE40 v041610.1 ChIP-seq Peaks Rep 1 from ENCODE/HAIB
HepG2 BHLHE40 v041610.1 ChIP-seq Raw Signal Rep 1 from ENCODE/HAIB
HepG BHLHE40 1
2
0
HepG2 BHLHE40 v041610.1 ChIP-seq Peaks Rep 2 from ENCODE/HAIB
HepG2 BHLHE40 v041610.1 ChIP-seq Raw Signal Rep 2 from ENCODE/HAIB
HepG BHLHE40 2
2
0

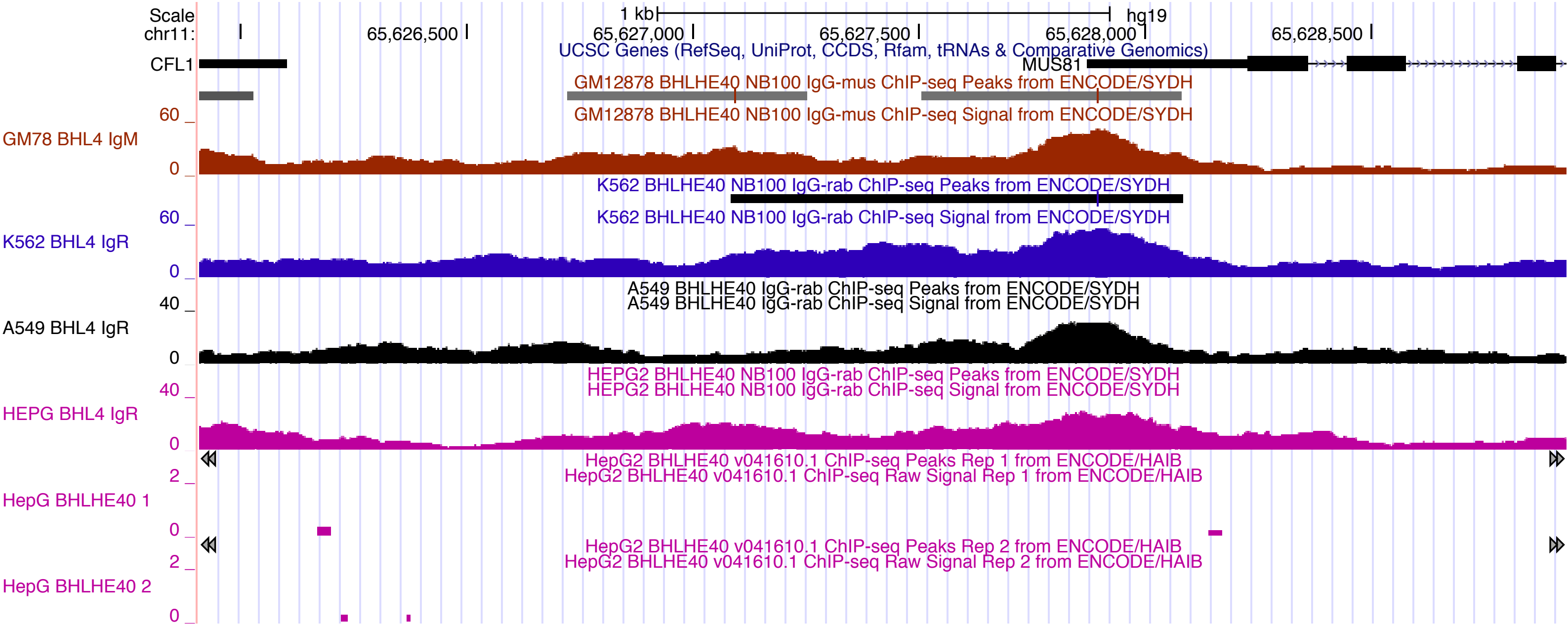
Scale
chr11:
1 kb
hg19
65,626,500
65,627,000
65,627,500
65,628,000
65,628,500
UCSC Genes (RefSeq, UniProt, CCDS, Rfam, tRNAs & Comparative Genomics)
CFL1
MUS81
GM12878 BHLHE40 NB100 IgG-mus ChIP-seq Peaks from ENCODE/SYDH
GM12878 BHLHE40 NB100 IgG-mus ChIP-seq Signal from ENCODE/SYDH
GM78 BHL4 IgM
K562 BHLHE40 NB100 IgG-rab ChIP-seq Peaks from ENCODE/SYDH
K562 BHLHE40 NB100 IgG-rab ChIP-seq Signal from ENCODE/SYDH
K562 BHL4 IgR
A549 BHLHE40 IgG-rab ChIP-seq Peaks from ENCODE/SYDH
A549 BHLHE40 IgG-rab ChIP-seq Signal from ENCODE/SYDH
A549 BHL4 IgR
HEPG2 BHLHE40 NB100 IgG-rab ChIP-seq Peaks from ENCODE/SYDH
HEPG2 BHLHE40 NB100 IgG-rab ChIP-seq Signal from ENCODE/SYDH
HEPG BHL4 IgR
HepG2 BHLHE40 v041610.1 ChIP-seq Peaks Rep 1 from ENCODE/HAIB
HepG2 BHLHE40 v041610.1 ChIP-seq Raw Signal Rep 1 from ENCODE/HAIB
HepG BHLHE40 1
HepG2 BHLHE40 v041610.1 ChIP-seq Peaks Rep 2 from ENCODE/HAIB
HepG2 BHLHE40 v041610.1 ChIP-seq Raw Signal Rep 2 from ENCODE/HAIB
HepG BHLHE40 2

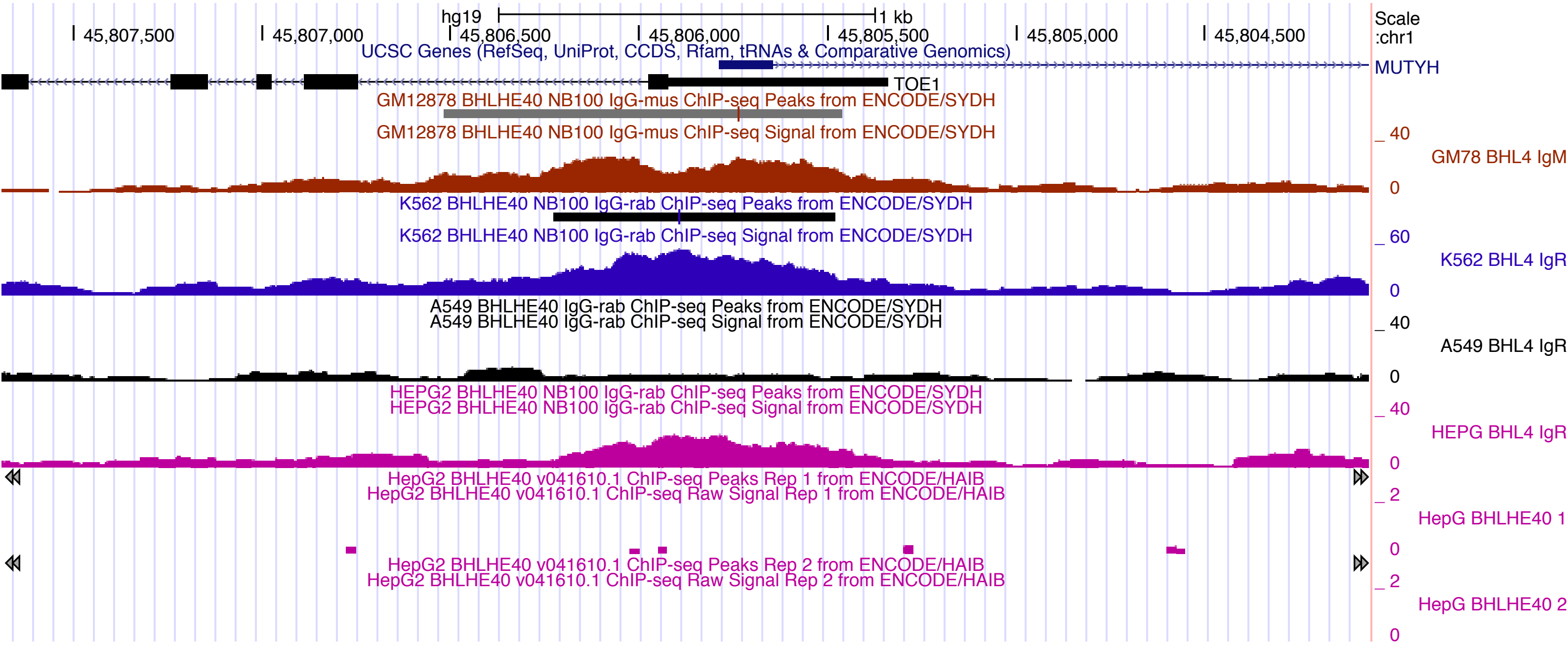

hg19
1 kb
Scale
:chr1
45,807,500
45,807,000
45,806,500
45,806,000
45,805,500
45,805,000
45,804,500
UCSC Genes (RefSeq, UniProt, CCDS, Rfam, tRNAs & Comparative Genomics)
MUTYH
TOE1
GM12878 BHLHE40 NB100 IgG-mus ChIP-seq Peaks from ENCODE/SYDH
GM12878 BHLHE40 NB100 IgG-mus ChIP-seq Signal from ENCODE/SYDH
GM78 BHL4 IgM
K562 BHLHE40 NB100 IgG-rab ChIP-seq Peaks from ENCODE/SYDH
K562 BHLHE40 NB100 IgG-rab ChIP-seq Signal from ENCODE/SYDH
K562 BHL4 IgR
A549 BHLHE40 IgG-rab ChIP-seq Peaks from ENCODE/SYDH
A549 BHLHE40 IgG-rab ChIP-seq Signal from ENCODE/SYDH
A549 BHL4 IgR
HEPG2 BHLHE40 NB100 IgG-rab ChIP-seq Peaks from ENCODE/SYDH
HEPG2 BHLHE40 NB100 IgG-rab ChIP-seq Signal from ENCODE/SYDH
HEPG BHL4 IgR
HepG2 BHLHE40 v041610.1 ChIP-seq Peaks Rep 1 from ENCODE/HAIB
HepG2 BHLHE40 v041610.1 ChIP-seq Raw Signal Rep 1 from ENCODE/HAIB
HepG BHLHE40 1
HepG2 BHLHE40 v041610.1 ChIP-seq Peaks Rep 2 from ENCODE/HAIB
HepG2 BHLHE40 v041610.1 ChIP-seq Raw Signal Rep 2 from ENCODE/HAIB
HepG BHLHE40 2

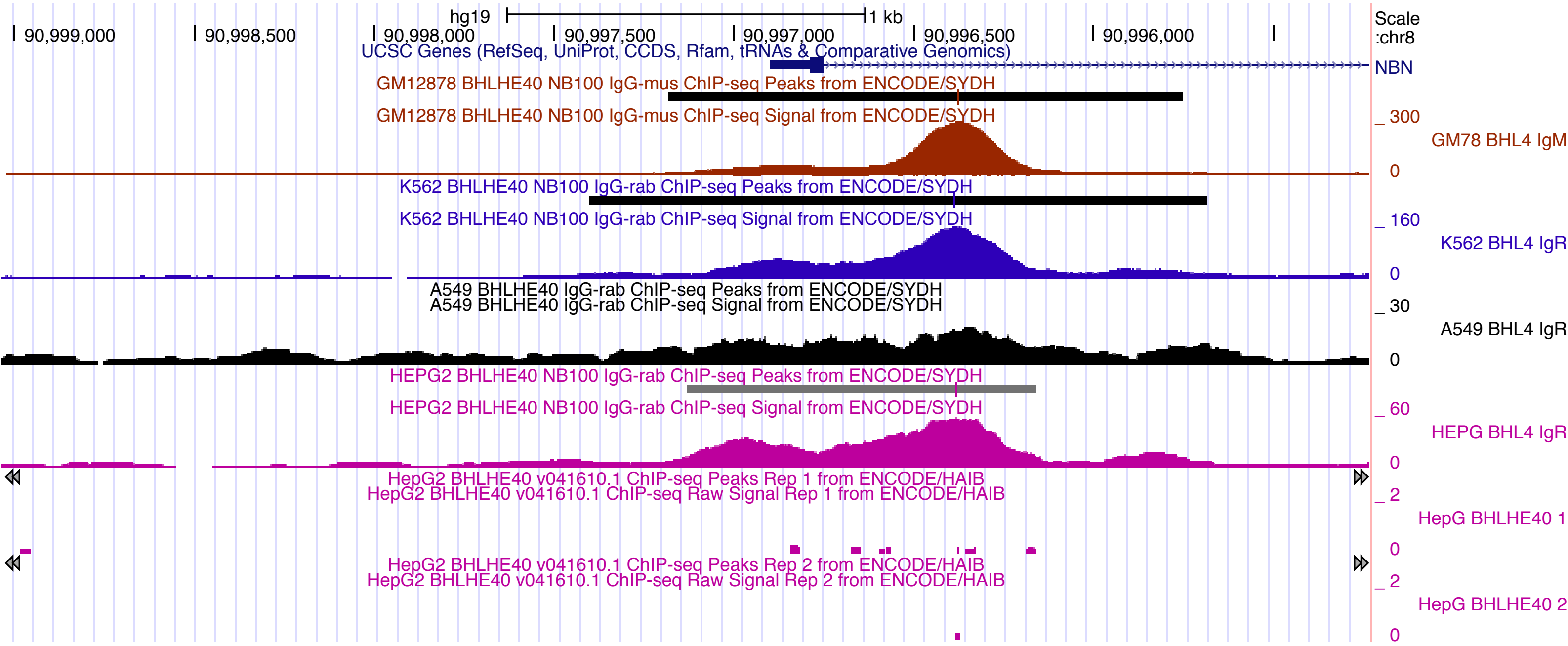

hg19
1 kb
Scale
:chr8
90,999,000
90,998,500
90,998,000
90,997,500
90,997,000
90,996,500
90,996,000
UCSC Genes (RefSeq, UniProt, CCDS, Rfam, tRNAs & Comparative Genomics)
NBN
GM12878 BHLHE40 NB100 IgG-mus ChIP-seq Peaks from ENCODE/SYDH
GM12878 BHLHE40 NB100 IgG-mus ChIP-seq Signal from ENCODE/SYDH
300
GM78 BHL4 IgM
0
K562 BHLHE40 NB100 IgG-rab ChIP-seq Peaks from ENCODE/SYDH
K562 BHLHE40 NB100 IgG-rab ChIP-seq Signal from ENCODE/SYDH
160
K562 BHL4 IgR
0
A549 BHLHE40 IgG-rab ChIP-seq Peaks from ENCODE/SYDH
A549 BHLHE40 IgG-rab ChIP-seq Signal from ENCODE/SYDH
30
A549 BHL4 IgR
0
HEPG2 BHLHE40 NB100 IgG-rab ChIP-seq Peaks from ENCODE/SYDH
HEPG2 BHLHE40 NB100 IgG-rab ChIP-seq Signal from ENCODE/SYDH
60
HEPG BHL4 IgR
0
HepG2 BHLHE40 v041610.1 ChIP-seq Peaks Rep 1 from ENCODE/HAIB
HepG2 BHLHE40 v041610.1 ChIP-seq Raw Signal Rep 1 from ENCODE/HAIB
2
HepG BHLHE40 1
0
HepG2 BHLHE40 v041610.1 ChIP-seq Peaks Rep 2 from ENCODE/HAIB
HepG2 BHLHE40 v041610.1 ChIP-seq Raw Signal Rep 2 from ENCODE/HAIB
2
HepG BHLHE40 2
0

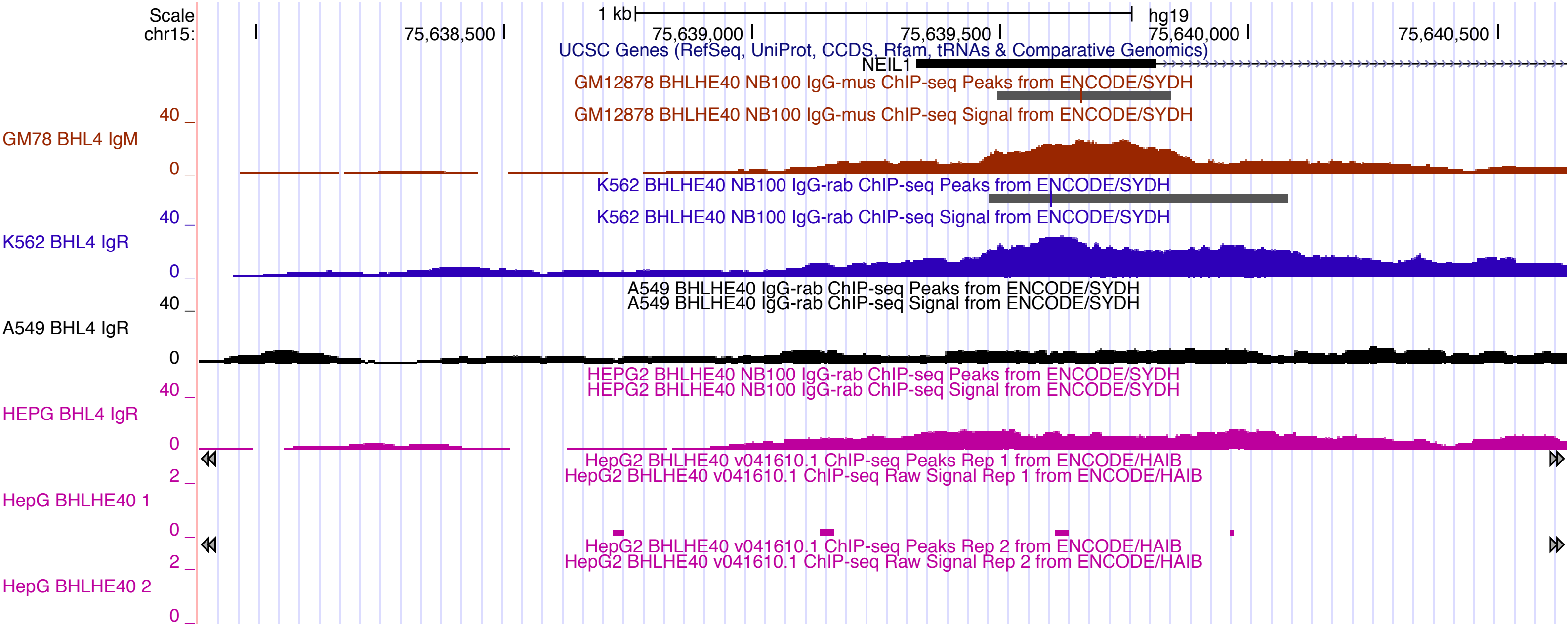
Scale
chr15:
1 kb
hg19
75,638,500
75,639,000
75,639,500
75,640,000
75,640,500
UCSC Genes (RefSeq, UniProt, CCDS, Rfam, tRNAs & Comparative Genomics)
NEIL1
GM12878 BHLHE40 NB100 IgG-mus ChIP-seq Peaks from ENCODE/SYDH
GM12878 BHLHE40 NB100 IgG-mus ChIP-seq Signal from ENCODE/SYDH
GM78 BHL4 IgM
K562 BHLHE40 NB100 IgG-rab ChIP-seq Peaks from ENCODE/SYDH
K562 BHLHE40 NB100 IgG-rab ChIP-seq Signal from ENCODE/SYDH
K562 BHL4 IgR
A549 BHLHE40 IgG-rab ChIP-seq Peaks from ENCODE/SYDH
A549 BHLHE40 IgG-rab ChIP-seq Signal from ENCODE/SYDH
A549 BHL4 IgR
HEPG2 BHLHE40 NB100 IgG-rab ChIP-seq Peaks from ENCODE/SYDH
HEPG2 BHLHE40 NB100 IgG-rab ChIP-seq Signal from ENCODE/SYDH
HEPG BHL4 IgR
HepG2 BHLHE40 v041610.1 ChIP-seq Peaks Rep 1 from ENCODE/HAIB
HepG2 BHLHE40 v041610.1 ChIP-seq Raw Signal Rep 1 from ENCODE/HAIB
HepG BHLHE40 1
HepG2 BHLHE40 v041610.1 ChIP-seq Peaks Rep 2 from ENCODE/HAIB
HepG2 BHLHE40 v041610.1 ChIP-seq Raw Signal Rep 2 from ENCODE/HAIB
HepG BHLHE40 2

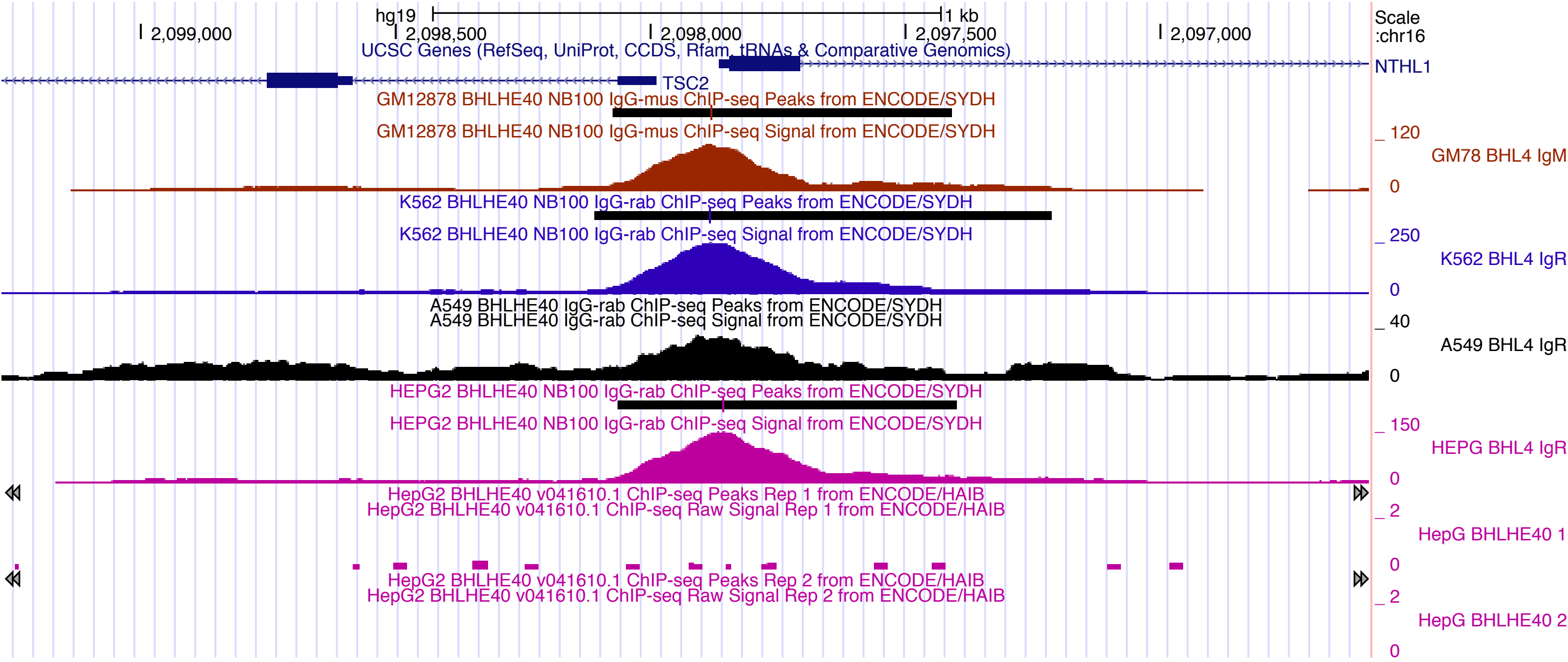

Scale
hg19
1 kb
:chr16
2,099,000
2,098,500
2,098,000
2,097,500
2,097,000
UCSC Genes (RefSeq, UniProt, CCDS, Rfam, tRNAs & Comparative Genomics)
NTHL1
TSC2
GM12878 BHLHE40 NB100 IgG-mus ChIP-seq Peaks from ENCODE/SYDH
GM12878 BHLHE40 NB100 IgG-mus ChIP-seq Signal from ENCODE/SYDH
120
GM78 BHL4 IgM
0
K562 BHLHE40 NB100 IgG-rab ChIP-seq Peaks from ENCODE/SYDH
K562 BHLHE40 NB100 IgG-rab ChIP-seq Signal from ENCODE/SYDH
250
K562 BHL4 IgR
0
A549 BHLHE40 IgG-rab ChIP-seq Peaks from ENCODE/SYDH
A549 BHLHE40 IgG-rab ChIP-seq Signal from ENCODE/SYDH
40
A549 BHL4 IgR
0
HEPG2 BHLHE40 NB100 IgG-rab ChIP-seq Peaks from ENCODE/SYDH
HEPG2 BHLHE40 NB100 IgG-rab ChIP-seq Signal from ENCODE/SYDH
150
HEPG BHL4 IgR
0
HepG2 BHLHE40 v041610.1 ChIP-seq Peaks Rep 1 from ENCODE/HAIB
HepG2 BHLHE40 v041610.1 ChIP-seq Raw Signal Rep 1 from ENCODE/HAIB
2
HepG BHLHE40 1
0
HepG2 BHLHE40 v041610.1 ChIP-seq Peaks Rep 2 from ENCODE/HAIB
HepG2 BHLHE40 v041610.1 ChIP-seq Raw Signal Rep 2 from ENCODE/HAIB
2
HepG BHLHE40 2
0

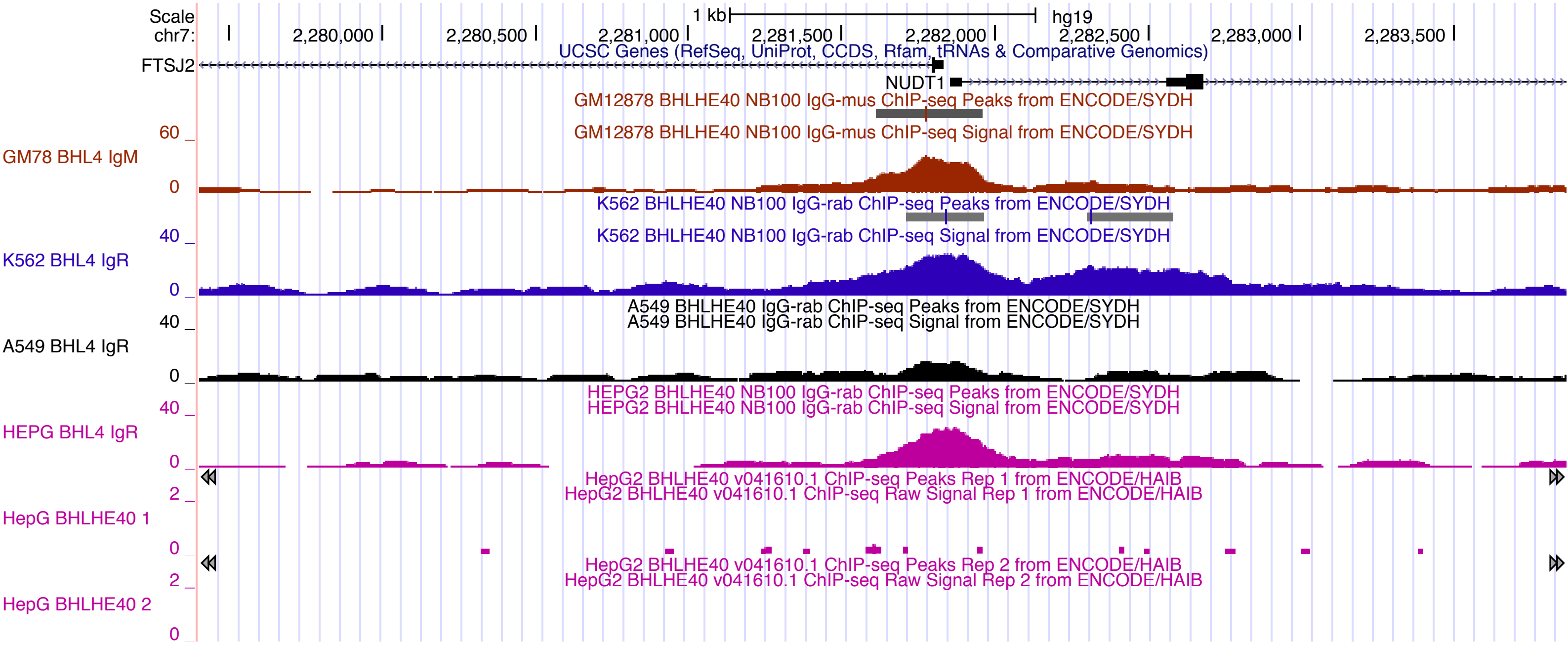

Scale
chr7:
1 kb
hg19
2,280,000
2,280,500
2,281,000
2,281,500
2,282,000
2,282,500
2,283,000
2,283,500
UCSC Genes (RefSeq, UniProt, CCDS, Rfam, tRNAs & Comparative Genomics)
FTSJ2
NUDT1
GM12878 BHLHE40 NB100 IgG-mus ChIP-seq Peaks from ENCODE/SYDH
GM12878 BHLHE40 NB100 IgG-mus ChIP-seq Signal from ENCODE/SYDH
GM78 BHL4 IgM
60
0
K562 BHLHE40 NB100 IgG-rab ChIP-seq Peaks from ENCODE/SYDH
K562 BHLHE40 NB100 IgG-rab ChIP-seq Signal from ENCODE/SYDH
K562 BHL4 IgR
40
0
A549 BHLHE40 IgG-rab ChIP-seq Peaks from ENCODE/SYDH
A549 BHLHE40 IgG-rab ChIP-seq Signal from ENCODE/SYDH
A549 BHL4 IgR
40
0
HEPG2 BHLHE40 NB100 IgG-rab ChIP-seq Peaks from ENCODE/SYDH
HEPG2 BHLHE40 NB100 IgG-rab ChIP-seq Signal from ENCODE/SYDH
HEPG BHL4 IgR
40
0
HepG2 BHLHE40 v041610.1 ChIP-seq Peaks Rep 1 from ENCODE/HAIB
HepG2 BHLHE40 v041610.1 ChIP-seq Raw Signal Rep 1 from ENCODE/HAIB
HepG BHLHE40 1
2
0
HepG2 BHLHE40 v041610.1 ChIP-seq Peaks Rep 2 from ENCODE/HAIB
HepG2 BHLHE40 v041610.1 ChIP-seq Raw Signal Rep 2 from ENCODE/HAIB
HepG BHLHE40 2
2
0

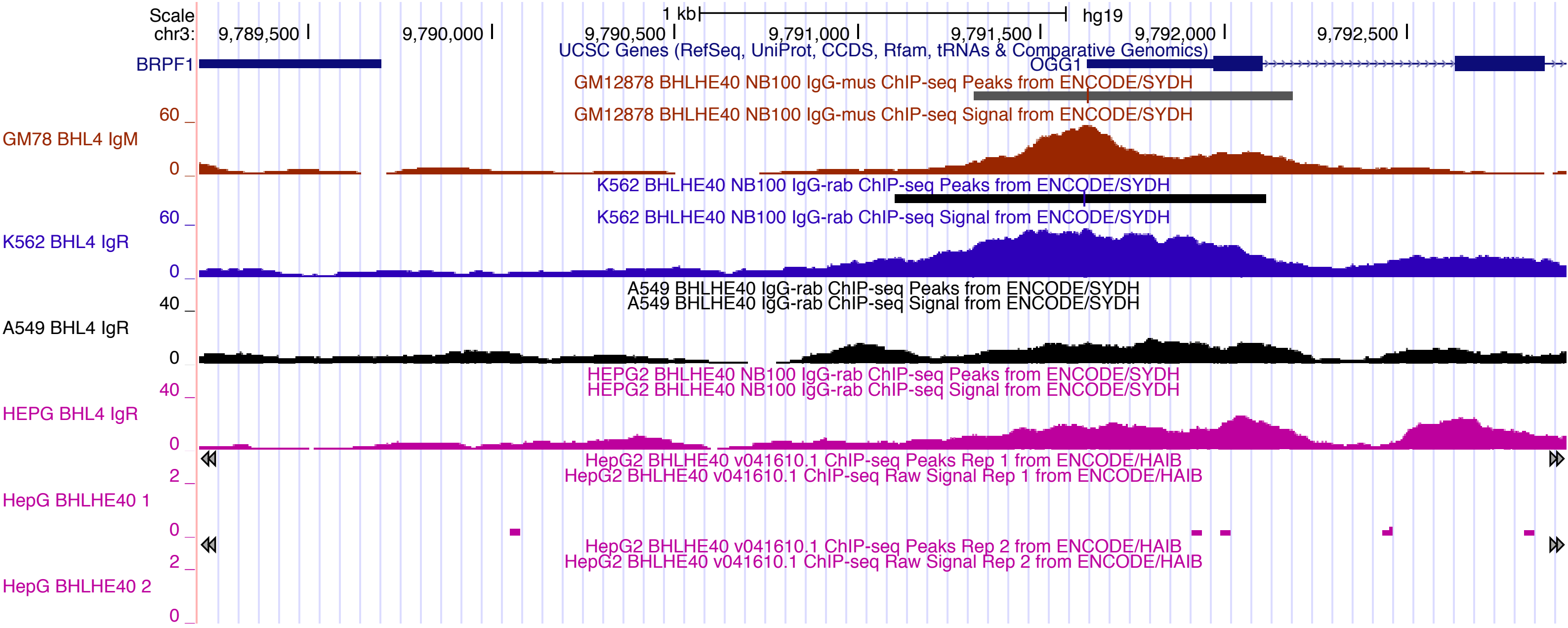

Scale
chr3:
1 kb
hg19
9,789,500
9,790,000
9,790,500
9,791,000
9,791,500
9,792,000
9,792,500
UCSC Genes (RefSeq, UniProt, CCDS, Rfam, tRNAs & Comparative Genomics)
BRPF1
OGG1
GM12878 BHLHE40 NB100 IgG-mus ChIP-seq Peaks from ENCODE/SYDH
GM12878 BHLHE40 NB100 IgG-mus ChIP-seq Signal from ENCODE/SYDH
GM78 BHL4 IgM
60
0
K562 BHLHE40 NB100 IgG-rab ChIP-seq Peaks from ENCODE/SYDH
K562 BHLHE40 NB100 IgG-rab ChIP-seq Signal from ENCODE/SYDH
K562 BHL4 IgR
60
0
A549 BHLHE40 IgG-rab ChIP-seq Peaks from ENCODE/SYDH
A549 BHLHE40 IgG-rab ChIP-seq Signal from ENCODE/SYDH
A549 BHL4 IgR
40
0
HEPG2 BHLHE40 NB100 IgG-rab ChIP-seq Peaks from ENCODE/SYDH
HEPG2 BHLHE40 NB100 IgG-rab ChIP-seq Signal from ENCODE/SYDH
HEPG BHL4 IgR
40
0
HepG2 BHLHE40 v041610.1 ChIP-seq Peaks Rep 1 from ENCODE/HAIB
HepG2 BHLHE40 v041610.1 ChIP-seq Raw Signal Rep 1 from ENCODE/HAIB
HepG BHLHE40 1
2
0
HepG2 BHLHE40 v041610.1 ChIP-seq Peaks Rep 2 from ENCODE/HAIB
HepG2 BHLHE40 v041610.1 ChIP-seq Raw Signal Rep 2 from ENCODE/HAIB
HepG BHLHE40 2
2
0

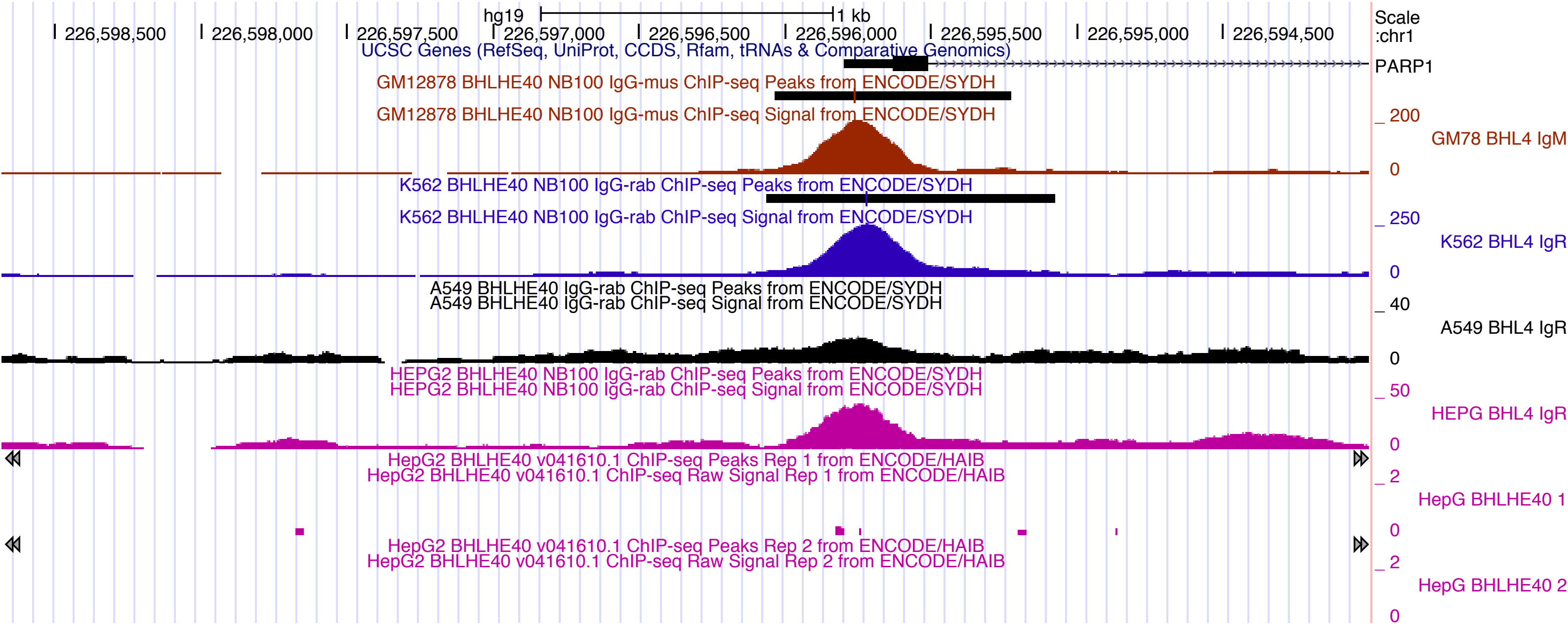
hg19
1 kb
Scale
:chr1
226,598,500
226,598,000
226,597,500
226,597,000
226,596,500
226,596,000
226,595,500
226,595,000
226,594,500
UCSC Genes (RefSeq, UniProt, CCDS, Rfam, tRNAs & Comparative Genomics)
PARP1
GM12878 BHLHE40 NB100 IgG-mus ChIP-seq Peaks from ENCODE/SYDH
GM12878 BHLHE40 NB100 IgG-mus ChIP-seq Signal from ENCODE/SYDH
200
GM78 BHL4 IgM
0
K562 BHLHE40 NB100 IgG-rab ChIP-seq Peaks from ENCODE/SYDH
K562 BHLHE40 NB100 IgG-rab ChIP-seq Signal from ENCODE/SYDH
250
K562 BHL4 IgR
0
A549 BHLHE40 IgG-rab ChIP-seq Peaks from ENCODE/SYDH
A549 BHLHE40 IgG-rab ChIP-seq Signal from ENCODE/SYDH
40
A549 BHL4 IgR
0
HEPG2 BHLHE40 NB100 IgG-rab ChIP-seq Peaks from ENCODE/SYDH
HEPG2 BHLHE40 NB100 IgG-rab ChIP-seq Signal from ENCODE/SYDH
50
HEPG BHL4 IgR
0
HepG2 BHLHE40 v041610.1 ChIP-seq Peaks Rep 1 from ENCODE/HAIB
HepG2 BHLHE40 v041610.1 ChIP-seq Raw Signal Rep 1 from ENCODE/HAIB
2
HepG BHLHE40 1
0
HepG2 BHLHE40 v041610.1 ChIP-seq Peaks Rep 2 from ENCODE/HAIB
HepG2 BHLHE40 v041610.1 ChIP-seq Raw Signal Rep 2 from ENCODE/HAIB
2
HepG BHLHE40 2
0

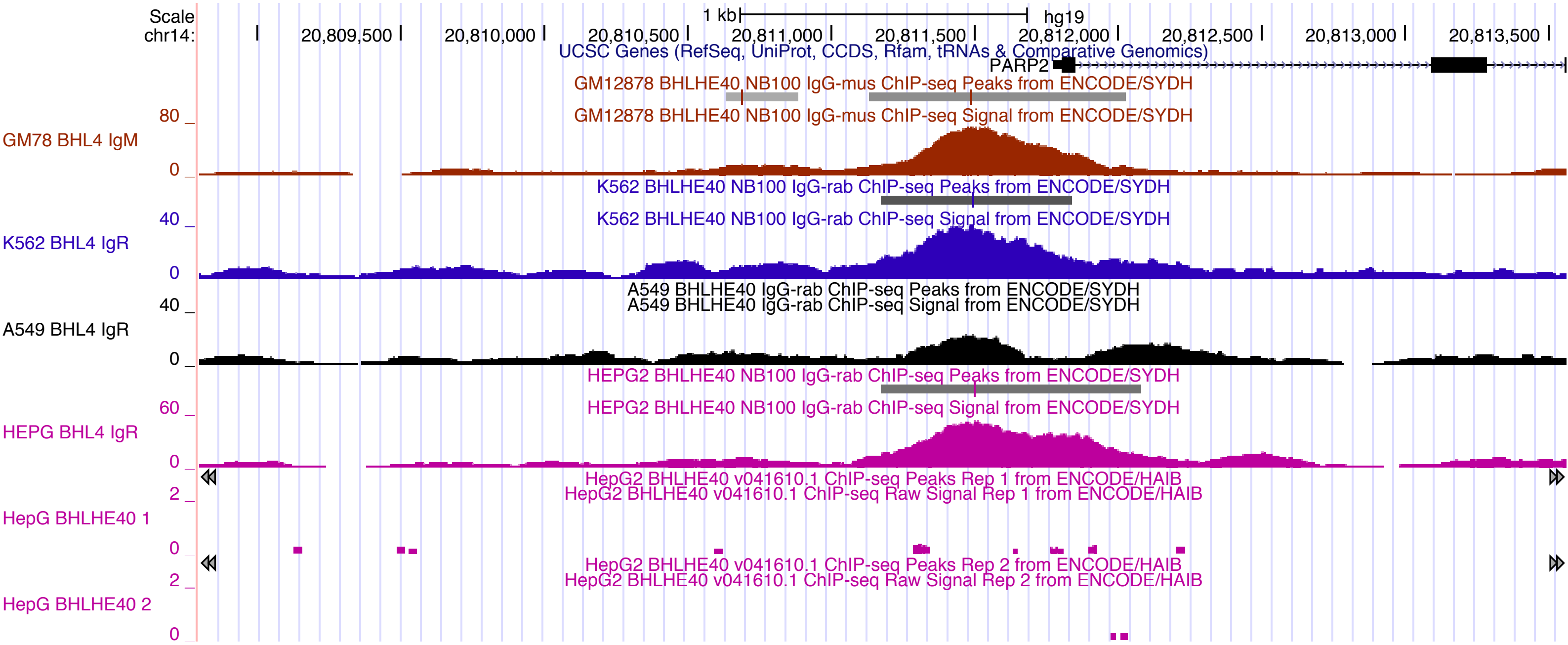
Scale
chr14:
1 kb
hg19
20,809,500
20,810,000
20,810,500
20,811,000
20,811,500
20,812,000
20,812,500
20,813,000
20,813,500
UCSC Genes (RefSeq, UniProt, CCDS, Rfam, tRNAs & Comparative Genomics)
PARP2
GM12878 BHLHE40 NB100 IgG-mus ChIP-seq Peaks from ENCODE/SYDH
GM12878 BHLHE40 NB100 IgG-mus ChIP-seq Signal from ENCODE/SYDH
GM78 BHL4 IgM
80 _
0 _
K562 BHLHE40 NB100 IgG-rab ChIP-seq Peaks from ENCODE/SYDH
K562 BHLHE40 NB100 IgG-rab ChIP-seq Signal from ENCODE/SYDH
K562 BHL4 IgR
40 _
0
A549 BHLHE40 IgG-rab ChIP-seq Peaks from ENCODE/SYDH
A549 BHLHE40 IgG-rab ChIP-seq Signal from ENCODE/SYDH
A549 BHL4 IgR
40 _
0 _
HEPG2 BHLHE40 NB100 IgG-rab ChIP-seq Peaks from ENCODE/SYDH
HEPG2 BHLHE40 NB100 IgG-rab ChIP-seq Signal from ENCODE/SYDH
HEPG BHL4 IgR
60 _
0 _
HepG2 BHLHE40 v041610.1 ChIP-seq Peaks Rep 1 from ENCODE/HAIB
HepG2 BHLHE40 v041610.1 ChIP-seq Raw Signal Rep 1 from ENCODE/HAIB
HepG BHLHE40 1
2 _
0
HepG2 BHLHE40 v041610.1 ChIP-seq Peaks Rep 2 from ENCODE/HAIB
HepG2 BHLHE40 v041610.1 ChIP-seq Raw Signal Rep 2 from ENCODE/HAIB
HepG BHLHE40 2
2 _
0

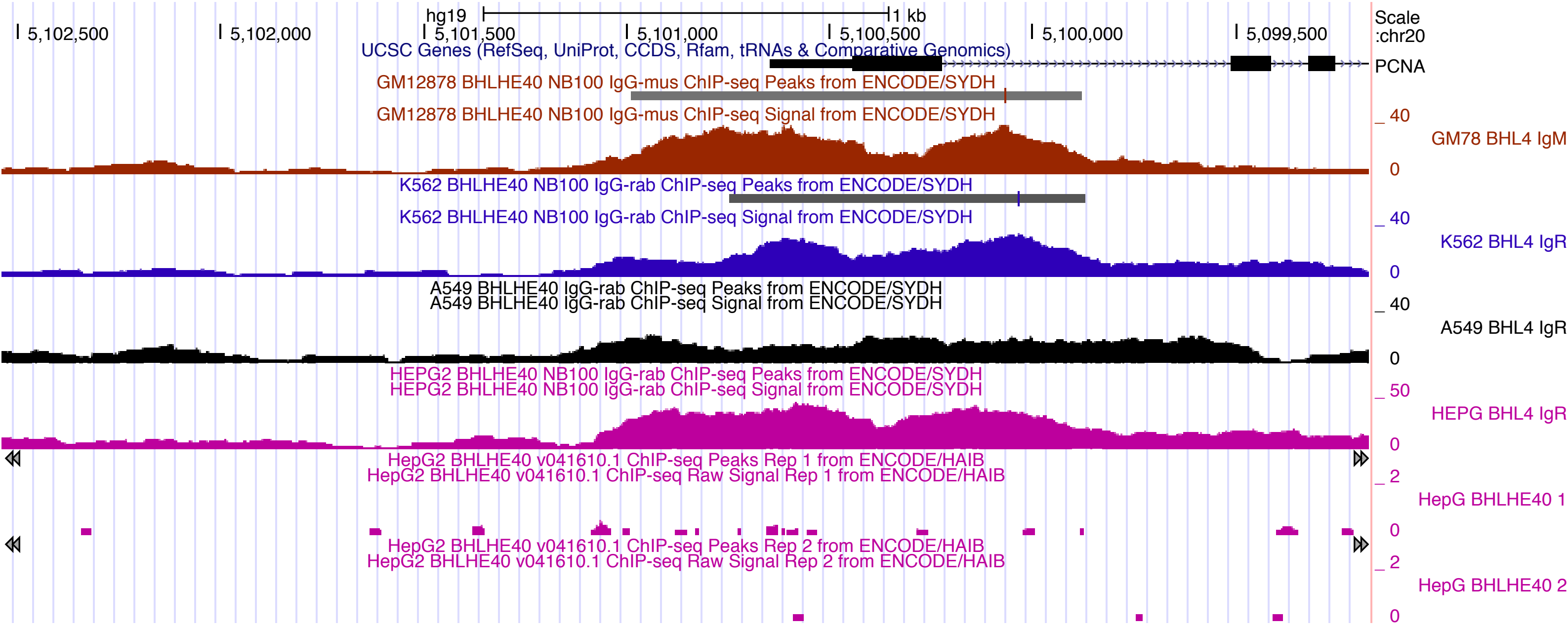

hg19
1 kb
Scale
chr20:
5,102,500
5,102,000
5,101,500
5,101,000
5,100,500
5,100,000
5,099,500
UCSC Genes (RefSeq, UniProt, CCDS, Rfam, tRNAs & Comparative Genomics)
PCNA
GM12878 BHLHE40 NB100 IgG-mus ChIP-seq Peaks from ENCODE/SYDH
GM12878 BHLHE40 NB100 IgG-mus ChIP-seq Signal from ENCODE/SYDH
GM78 BHL4 IgM
K562 BHLHE40 NB100 IgG-rab ChIP-seq Peaks from ENCODE/SYDH
K562 BHLHE40 NB100 IgG-rab ChIP-seq Signal from ENCODE/SYDH
K562 BHL4 IgR
A549 BHLHE40 IgG-rab ChIP-seq Peaks from ENCODE/SYDH
A549 BHLHE40 IgG-rab ChIP-seq Signal from ENCODE/SYDH
A549 BHL4 IgR
HEPG2 BHLHE40 NB100 IgG-rab ChIP-seq Peaks from ENCODE/SYDH
HEPG2 BHLHE40 NB100 IgG-rab ChIP-seq Signal from ENCODE/SYDH
HEPG BHL4 IgR
HepG2 BHLHE40 v041610.1 ChIP-seq Peaks Rep 1 from ENCODE/HAIB
HepG2 BHLHE40 v041610.1 ChIP-seq Raw Signal Rep 1 from ENCODE/HAIB
HepG BHLHE40 1
HepG2 BHLHE40 v041610.1 ChIP-seq Peaks Rep 2 from ENCODE/HAIB
HepG2 BHLHE40 v041610.1 ChIP-seq Raw Signal Rep 2 from ENCODE/HAIB
HepG BHLHE40 2
40
0
50
2

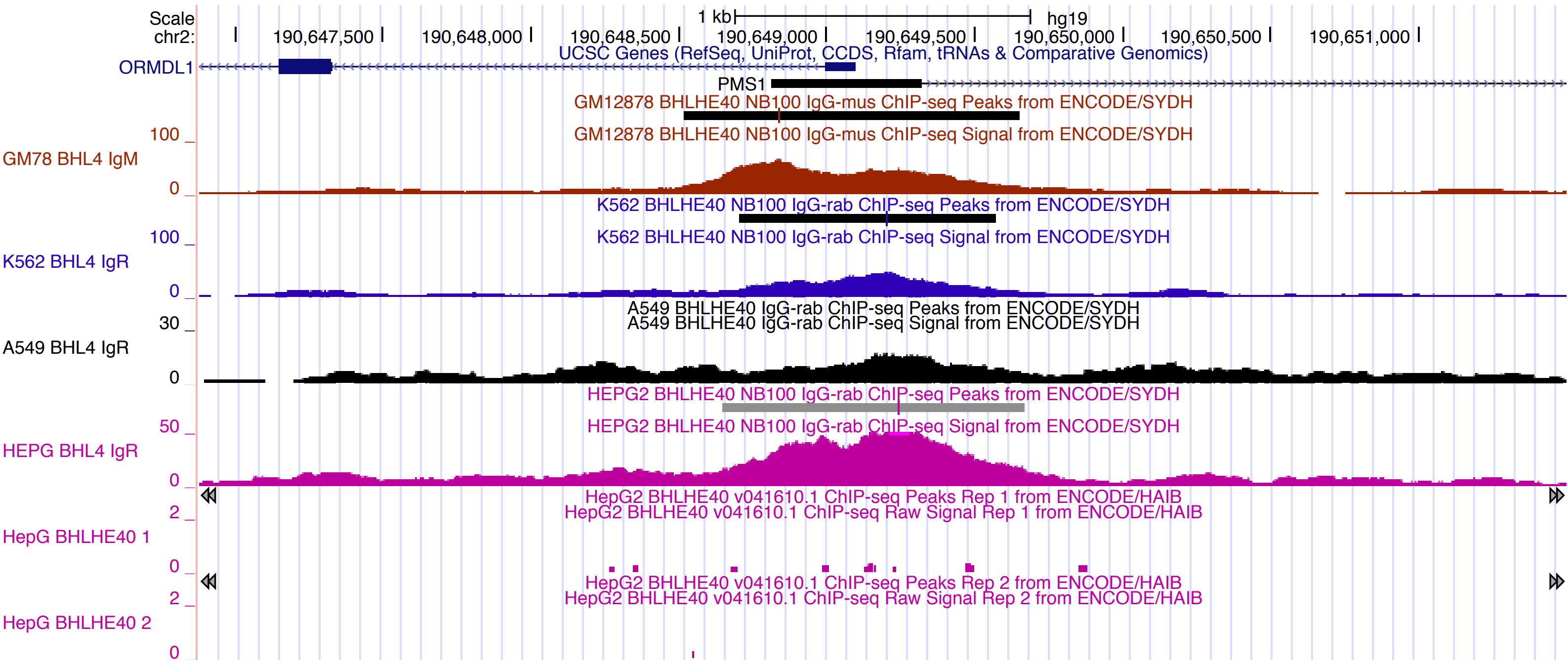
Scale
chr2:
1 kb
hg19
190,647,500
190,648,000
190,648,500
190,649,000
190,649,500
190,650,000
190,650,500
190,651,000
UCSC Genes (RefSeq, UniProt, CCDS, Rfam, tRNAs & Comparative Genomics)
ORMDL1
PMS1
GM12878 BHLHE40 NB100 IgG-mus ChIP-seq Peaks from ENCODE/SYDH
GM12878 BHLHE40 NB100 IgG-mus ChIP-seq Signal from ENCODE/SYDH
100
GM78 BHL4 IgM
0
K562 BHLHE40 NB100 IgG-rab ChIP-seq Peaks from ENCODE/SYDH
K562 BHLHE40 NB100 IgG-rab ChIP-seq Signal from ENCODE/SYDH
100
K562 BHL4 IgR
0
A549 BHLHE40 IgG-rab ChIP-seq Peaks from ENCODE/SYDH
A549 BHLHE40 IgG-rab ChIP-seq Signal from ENCODE/SYDH
30
A549 BHL4 IgR
0
HEPG2 BHLHE40 NB100 IgG-rab ChIP-seq Peaks from ENCODE/SYDH
HEPG2 BHLHE40 NB100 IgG-rab ChIP-seq Signal from ENCODE/SYDH
50
HEPG BHL4 IgR
0
HepG2 BHLHE40 v041610.1 ChIP-seq Peaks Rep 1 from ENCODE/HAIB
HepG2 BHLHE40 v041610.1 ChIP-seq Raw Signal Rep 1 from ENCODE/HAIB
2
HepG BHLHE40 1
0
HepG2 BHLHE40 v041610.1 ChIP-seq Peaks Rep 2 from ENCODE/HAIB
HepG2 BHLHE40 v041610.1 ChIP-seq Raw Signal Rep 2 from ENCODE/HAIB
2
HepG BHLHE40 2
0

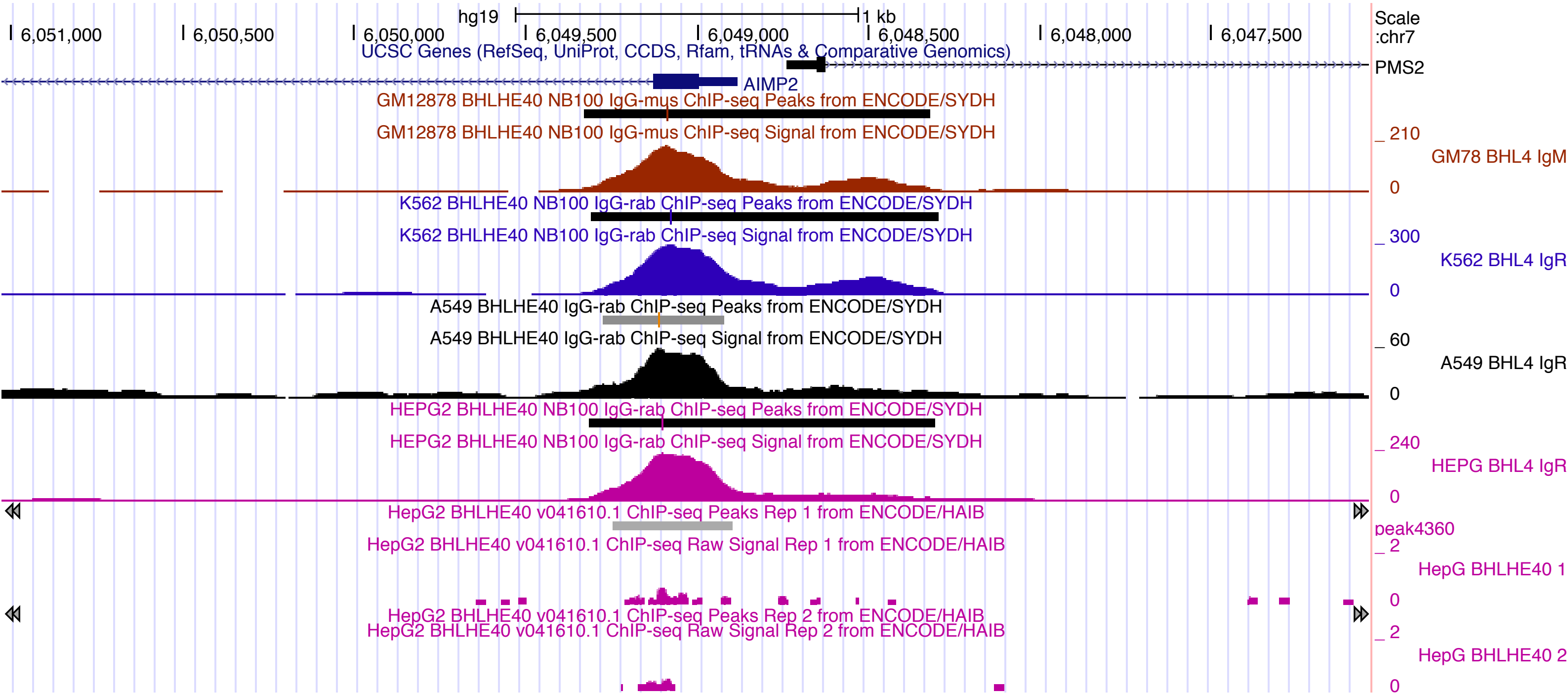

hg19
1 kb
Scale
:chr7
6,051,000
6,050,500
6,050,000
6,049,500
6,049,000
6,048,500
6,048,000
6,047,500
UCSC Genes (RefSeq, UniProt, CCDS, Rfam, tRNAs & Comparative Genomics)
PMS2
AIMP2
GM12878 BHLHE40 NB100 IgG-mus ChIP-seq Peaks from ENCODE/SYDH
GM12878 BHLHE40 NB100 IgG-mus ChIP-seq Signal from ENCODE/SYDH
_ 210
GM78 BHL4 IgM
0
K562 BHLHE40 NB100 IgG-rab ChIP-seq Peaks from ENCODE/SYDH
K562 BHLHE40 NB100 IgG-rab ChIP-seq Signal from ENCODE/SYDH
_ 300
K562 BHL4 IgR
0
A549 BHLHE40 IgG-rab ChIP-seq Peaks from ENCODE/SYDH
A549 BHLHE40 IgG-rab ChIP-seq Signal from ENCODE/SYDH
_ 60
A549 BHL4 IgR
0
HEPG2 BHLHE40 NB100 IgG-rab ChIP-seq Peaks from ENCODE/SYDH
HEPG2 BHLHE40 NB100 IgG-rab ChIP-seq Signal from ENCODE/SYDH
_ 240
HEPG BHL4 IgR
0
HepG2 BHLHE40 v041610.1 ChIP-seq Peaks Rep 1 from ENCODE/HAIB
peak4360
HepG2 BHLHE40 v041610.1 ChIP-seq Raw Signal Rep 1 from ENCODE/HAIB
_ 2
HepG BHLHE40 1
0
HepG2 BHLHE40 v041610.1 ChIP-seq Peaks Rep 2 from ENCODE/HAIB
HepG2 BHLHE40 v041610.1 ChIP-seq Raw Signal Rep 2 from ENCODE/HAIB
_ 2
HepG BHLHE40 2
0

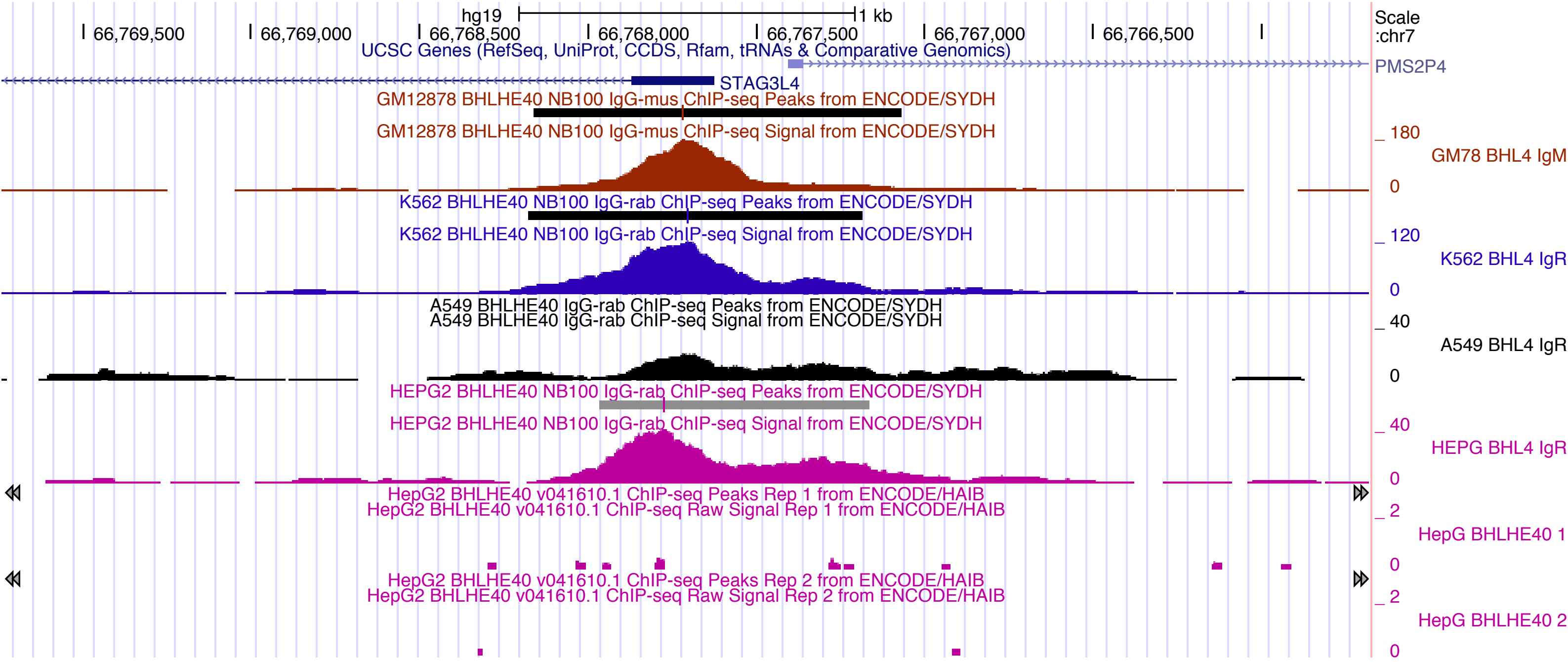

hg19
1 kb
Scale
:chr7
66,769,500
66,769,000
66,768,500
66,768,000
66,767,500
66,767,000
66,766,500
UCSC Genes (RefSeq, UniProt, CCDS, Rfam, tRNAs & Comparative Genomics)
PMS2P4
STAG3L4
GM12878 BHLHE40 NB100 IgG-mus ChIP-seq Peaks from ENCODE/SYDH
GM12878 BHLHE40 NB100 IgG-mus ChIP-seq Signal from ENCODE/SYDH
_ 180
GM78 BHL4 IgM
0
K562 BHLHE40 NB100 IgG-rab ChIP-seq Peaks from ENCODE/SYDH
K562 BHLHE40 NB100 IgG-rab ChIP-seq Signal from ENCODE/SYDH
_ 120
K562 BHL4 IgR
0
A549 BHLHE40 IgG-rab ChIP-seq Peaks from ENCODE/SYDH
A549 BHLHE40 IgG-rab ChIP-seq Signal from ENCODE/SYDH
_ 40
A549 BHL4 IgR
0
HEPG2 BHLHE40 NB100 IgG-rab ChIP-seq Peaks from ENCODE/SYDH
HEPG2 BHLHE40 NB100 IgG-rab ChIP-seq Signal from ENCODE/SYDH
_ 40
HEPG BHL4 IgR
0
HepG2 BHLHE40 v041610.1 ChIP-seq Peaks Rep 1 from ENCODE/HAIB
HepG2 BHLHE40 v041610.1 ChIP-seq Raw Signal Rep 1 from ENCODE/HAIB
_ 2
HepG BHLHE40 1
0
HepG2 BHLHE40 v041610.1 ChIP-seq Peaks Rep 2 from ENCODE/HAIB
HepG2 BHLHE40 v041610.1 ChIP-seq Raw Signal Rep 2 from ENCODE/HAIB
_ 2
HepG BHLHE40 2
0

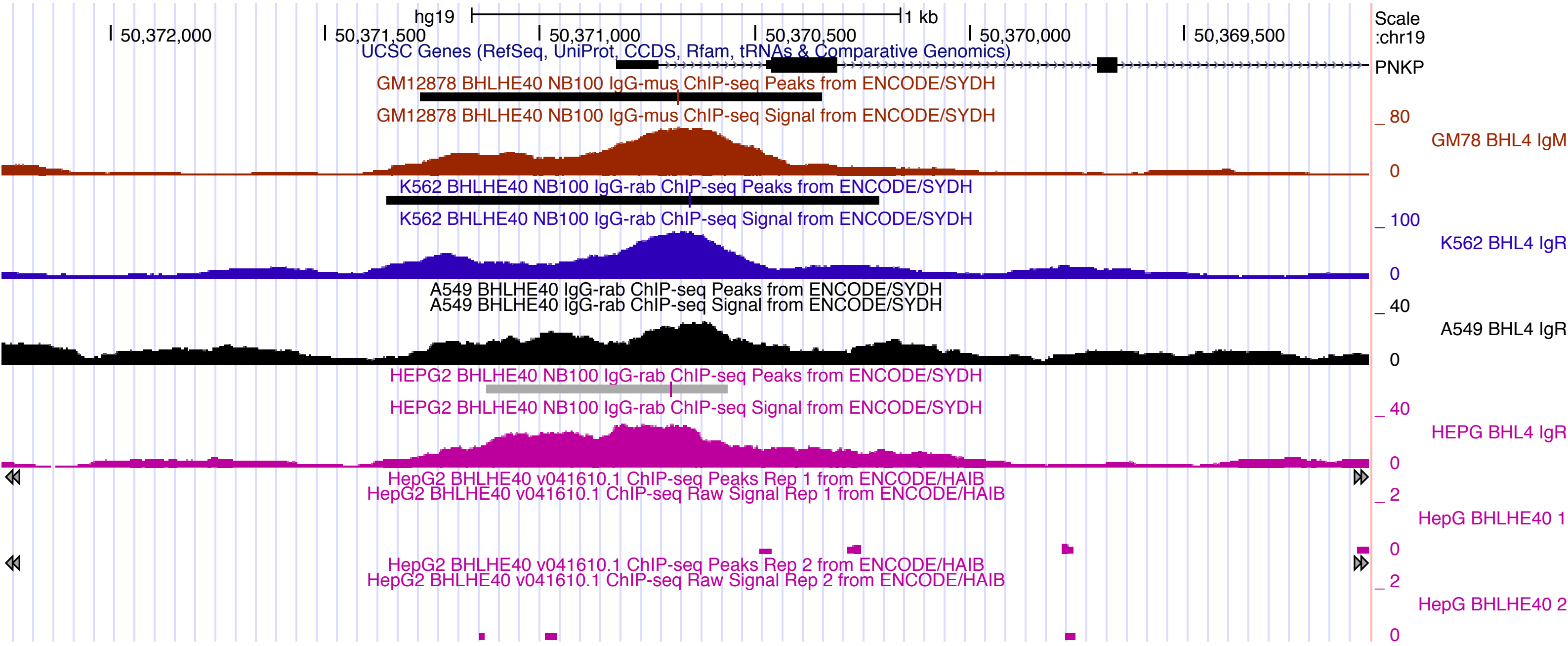

hg19
1 kb
Scale
:chr19
50,372,000
50,371,500
50,371,000
50,370,500
50,370,000
50,369,500
UCSC Genes (RefSeq, UniProt, CCDS, Rfam, tRNAs & Comparative Genomics)
PNKP
GM12878 BHLHE40 NB100 IgG-mus ChIP-seq Peaks from ENCODE/SYDH
GM12878 BHLHE40 NB100 IgG-mus ChIP-seq Signal from ENCODE/SYDH
GM78 BHL4 IgM
K562 BHLHE40 NB100 IgG-rab ChIP-seq Peaks from ENCODE/SYDH
K562 BHLHE40 NB100 IgG-rab ChIP-seq Signal from ENCODE/SYDH
K562 BHL4 IgR
A549 BHLHE40 IgG-rab ChIP-seq Peaks from ENCODE/SYDH
A549 BHLHE40 IgG-rab ChIP-seq Signal from ENCODE/SYDH
A549 BHL4 IgR
HEPG2 BHLHE40 NB100 IgG-rab ChIP-seq Peaks from ENCODE/SYDH
HEPG2 BHLHE40 NB100 IgG-rab ChIP-seq Signal from ENCODE/SYDH
HEPG BHL4 IgR
HepG2 BHLHE40 v041610.1 ChIP-seq Peaks Rep 1 from ENCODE/HAIB
HepG2 BHLHE40 v041610.1 ChIP-seq Raw Signal Rep 1 from ENCODE/HAIB
HepG BHLHE40 1
HepG2 BHLHE40 v041610.1 ChIP-seq Peaks Rep 2 from ENCODE/HAIB
HepG2 BHLHE40 v041610.1 ChIP-seq Raw Signal Rep 2 from ENCODE/HAIB
HepG BHLHE40 2
80
0
100
0
40
0
40
0
2
0
2
0

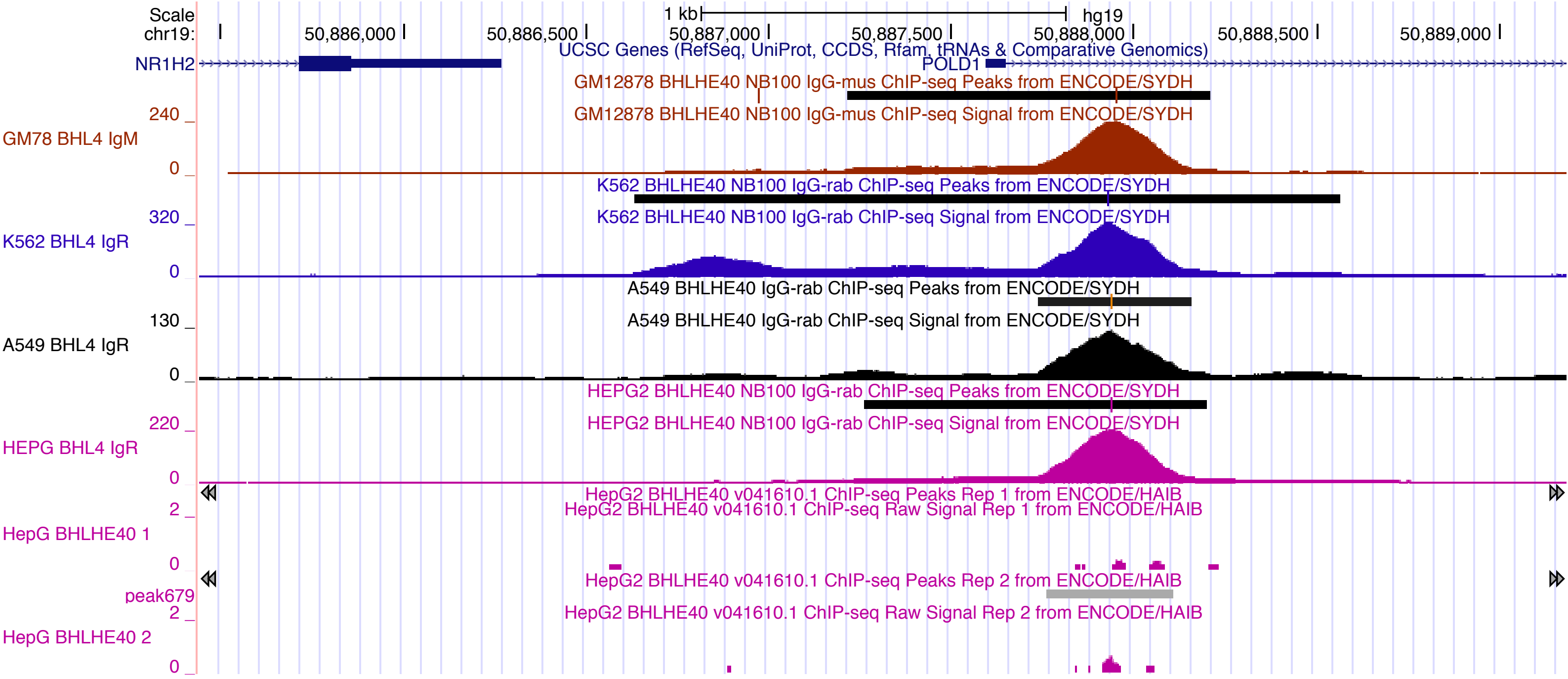
Scale
chr19:
1 kb
hg19
50,886,000
50,886,500
50,887,000
50,887,500
50,888,000
50,888,500
50,889,000
UCSC Genes (RefSeq, UniProt, CCDS, Rfam, tRNAs & Comparative Genomics)
NR1H2
POLD1
GM12878 BHLHE40 NB100 IgG-mus ChIP-seq Peaks from ENCODE/SYDH
GM12878 BHLHE40 NB100 IgG-mus ChIP-seq Signal from ENCODE/SYDH
240
GM78 BHL4 IgM
0
K562 BHLHE40 NB100 IgG-rab ChIP-seq Peaks from ENCODE/SYDH
K562 BHLHE40 NB100 IgG-rab ChIP-seq Signal from ENCODE/SYDH
320
K562 BHL4 IgR
0
A549 BHLHE40 IgG-rab ChIP-seq Peaks from ENCODE/SYDH
A549 BHLHE40 IgG-rab ChIP-seq Signal from ENCODE/SYDH
130
A549 BHL4 IgR
0
HEPG2 BHLHE40 NB100 IgG-rab ChIP-seq Peaks from ENCODE/SYDH
HEPG2 BHLHE40 NB100 IgG-rab ChIP-seq Signal from ENCODE/SYDH
220
HEPG BHL4 IgR
0
HepG2 BHLHE40 v041610.1 ChIP-seq Peaks Rep 1 from ENCODE/HAIB
HepG2 BHLHE40 v041610.1 ChIP-seq Raw Signal Rep 1 from ENCODE/HAIB
2
HepG BHLHE40 1
0
HepG2 BHLHE40 v041610.1 ChIP-seq Peaks Rep 2 from ENCODE/HAIB
peak679
2
HepG2 BHLHE40 v041610.1 ChIP-seq Raw Signal Rep 2 from ENCODE/HAIB
HepG BHLHE40 2
0

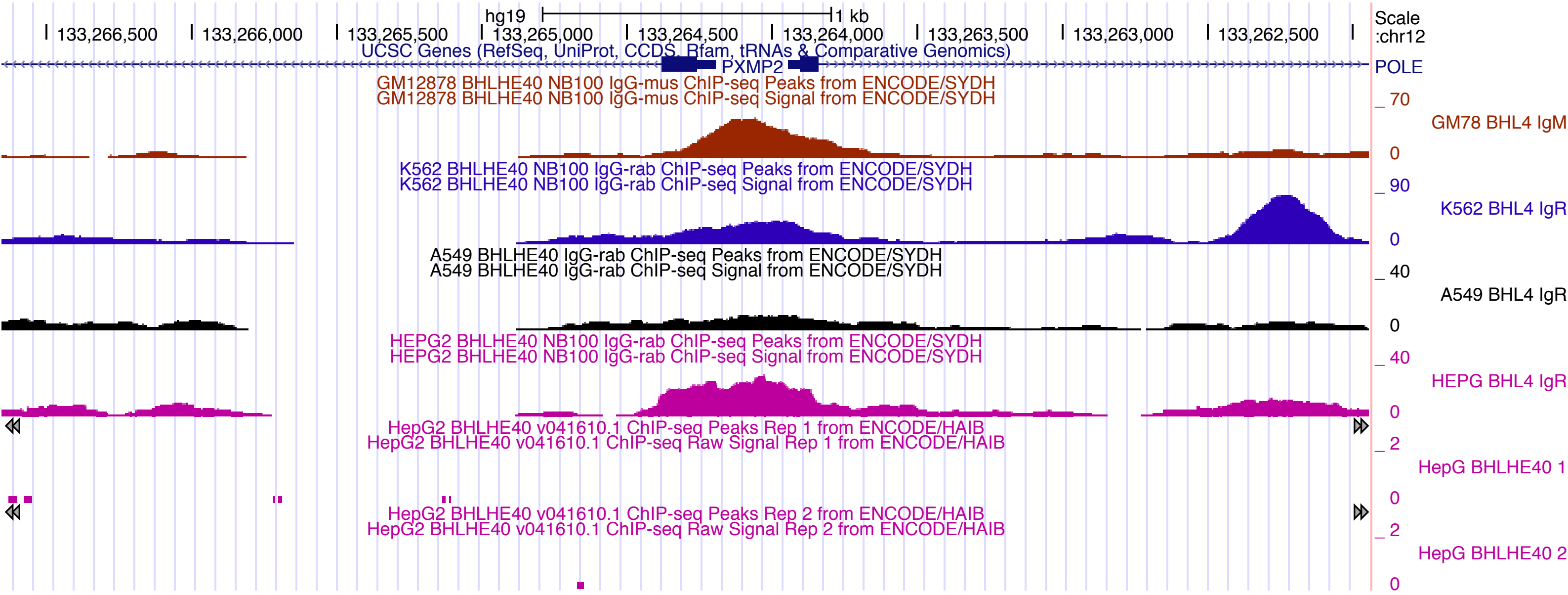

hg19
1 kb
Scale
:chr12
133,266,500
133,266,000
133,265,500
133,265,000
133,264,500
133,264,000
133,263,500
133,263,000
133,262,500
UCSC Genes (RefSeq, UniProt, CCDS, Rfam, tRNAs & Comparative Genomics)
PXMP2
POLE
GM12878 BHLHE40 NB100 IgG-mus ChIP-seq Peaks from ENCODE/SYDH
GM12878 BHLHE40 NB100 IgG-mus ChIP-seq Signal from ENCODE/SYDH
GM78 BHL4 IgM
K562 BHLHE40 NB100 IgG-rab ChIP-seq Peaks from ENCODE/SYDH
K562 BHLHE40 NB100 IgG-rab ChIP-seq Signal from ENCODE/SYDH
K562 BHL4 IgR
A549 BHLHE40 IgG-rab ChIP-seq Peaks from ENCODE/SYDH
A549 BHLHE40 IgG-rab ChIP-seq Signal from ENCODE/SYDH
A549 BHL4 IgR
HEPG2 BHLHE40 NB100 IgG-rab ChIP-seq Peaks from ENCODE/SYDH
HEPG2 BHLHE40 NB100 IgG-rab ChIP-seq Signal from ENCODE/SYDH
HEPG BHL4 IgR
HepG2 BHLHE40 v041610.1 ChIP-seq Peaks Rep 1 from ENCODE/HAIB
HepG2 BHLHE40 v041610.1 ChIP-seq Raw Signal Rep 1 from ENCODE/HAIB
HepG BHLHE40 1
HepG2 BHLHE40 v041610.1 ChIP-seq Peaks Rep 2 from ENCODE/HAIB
HepG2 BHLHE40 v041610.1 ChIP-seq Raw Signal Rep 2 from ENCODE/HAIB
HepG BHLHE40 2
_ 70
0
_ 90
0
_ 40
0
_ 40
0
_ 2
0
_ 2
0

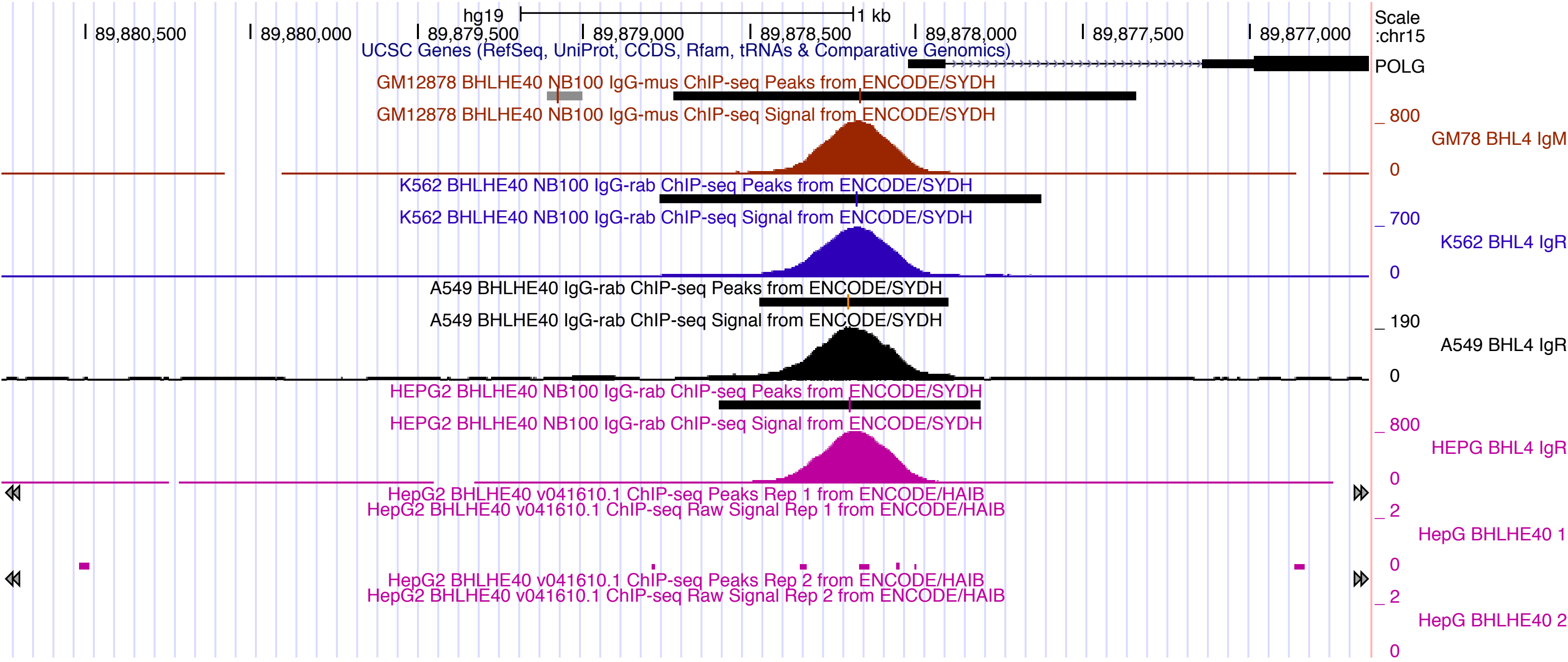
hg19
1 kb
Scale
:chr15
89,880,500
89,880,000
89,879,500
89,879,000
89,878,500
89,878,000
89,877,500
89,877,000
UCSC Genes (RefSeq, UniProt, CCDS, Rfam, tRNAs & Comparative Genomics)
POLG
GM12878 BHLHE40 NB100 IgG-mus ChIP-seq Peaks from ENCODE/SYDH
GM12878 BHLHE40 NB100 IgG-mus ChIP-seq Signal from ENCODE/SYDH
800
GM78 BHL4 IgM
0
K562 BHLHE40 NB100 IgG-rab ChIP-seq Peaks from ENCODE/SYDH
K562 BHLHE40 NB100 IgG-rab ChIP-seq Signal from ENCODE/SYDH
700
K562 BHL4 IgR
0
A549 BHLHE40 IgG-rab ChIP-seq Peaks from ENCODE/SYDH
A549 BHLHE40 IgG-rab ChIP-seq Signal from ENCODE/SYDH
190
A549 BHL4 IgR
0
HEPG2 BHLHE40 NB100 IgG-rab ChIP-seq Peaks from ENCODE/SYDH
HEPG2 BHLHE40 NB100 IgG-rab ChIP-seq Signal from ENCODE/SYDH
800
HEPG BHL4 IgR
0
HepG2 BHLHE40 v041610.1 ChIP-seq Peaks Rep 1 from ENCODE/HAIB
HepG2 BHLHE40 v041610.1 ChIP-seq Raw Signal Rep 1 from ENCODE/HAIB
2
HepG BHLHE40 1
0
HepG2 BHLHE40 v041610.1 ChIP-seq Peaks Rep 2 from ENCODE/HAIB
HepG2 BHLHE40 v041610.1 ChIP-seq Raw Signal Rep 2 from ENCODE/HAIB
2
HepG BHLHE40 2
0

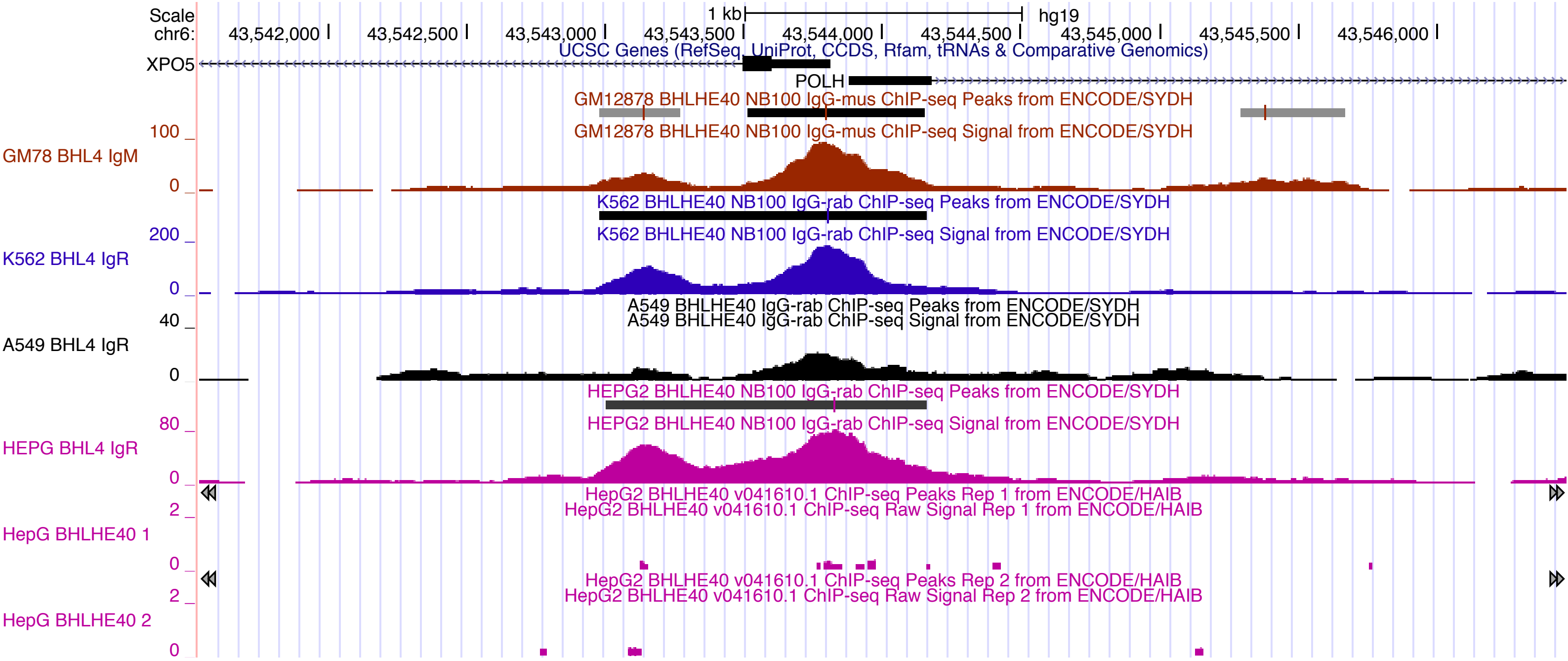

Scale
chr6:
1 kb
hg19
43,542,000
43,542,500
43,543,000
43,543,500
43,544,000
43,544,500
43,545,000
43,545,500
43,546,000
UCSC Genes (RefSeq, UniProt, CCDS, Rfam, tRNAs & Comparative Genomics)
XPO5
POLH
GM12878 BHLHE40 NB100 IgG-mus ChIP-seq Peaks from ENCODE/SYDH
GM12878 BHLHE40 NB100 IgG-mus ChIP-seq Signal from ENCODE/SYDH
GM78 BHL4 IgM
100
0
K562 BHLHE40 NB100 IgG-rab ChIP-seq Peaks from ENCODE/SYDH
K562 BHLHE40 NB100 IgG-rab ChIP-seq Signal from ENCODE/SYDH
K562 BHL4 IgR
200
0
A549 BHLHE40 IgG-rab ChIP-seq Peaks from ENCODE/SYDH
A549 BHLHE40 IgG-rab ChIP-seq Signal from ENCODE/SYDH
A549 BHL4 IgR
40
0
HEPG2 BHLHE40 NB100 IgG-rab ChIP-seq Peaks from ENCODE/SYDH
HEPG2 BHLHE40 NB100 IgG-rab ChIP-seq Signal from ENCODE/SYDH
HEPG BHL4 IgR
80
0
HepG2 BHLHE40 v041610.1 ChIP-seq Peaks Rep 1 from ENCODE/HAIB
HepG2 BHLHE40 v041610.1 ChIP-seq Raw Signal Rep 1 from ENCODE/HAIB
HepG BHLHE40 1
2
0
HepG2 BHLHE40 v041610.1 ChIP-seq Peaks Rep 2 from ENCODE/HAIB
HepG2 BHLHE40 v041610.1 ChIP-seq Raw Signal Rep 2 from ENCODE/HAIB
HepG BHLHE40 2
2
0

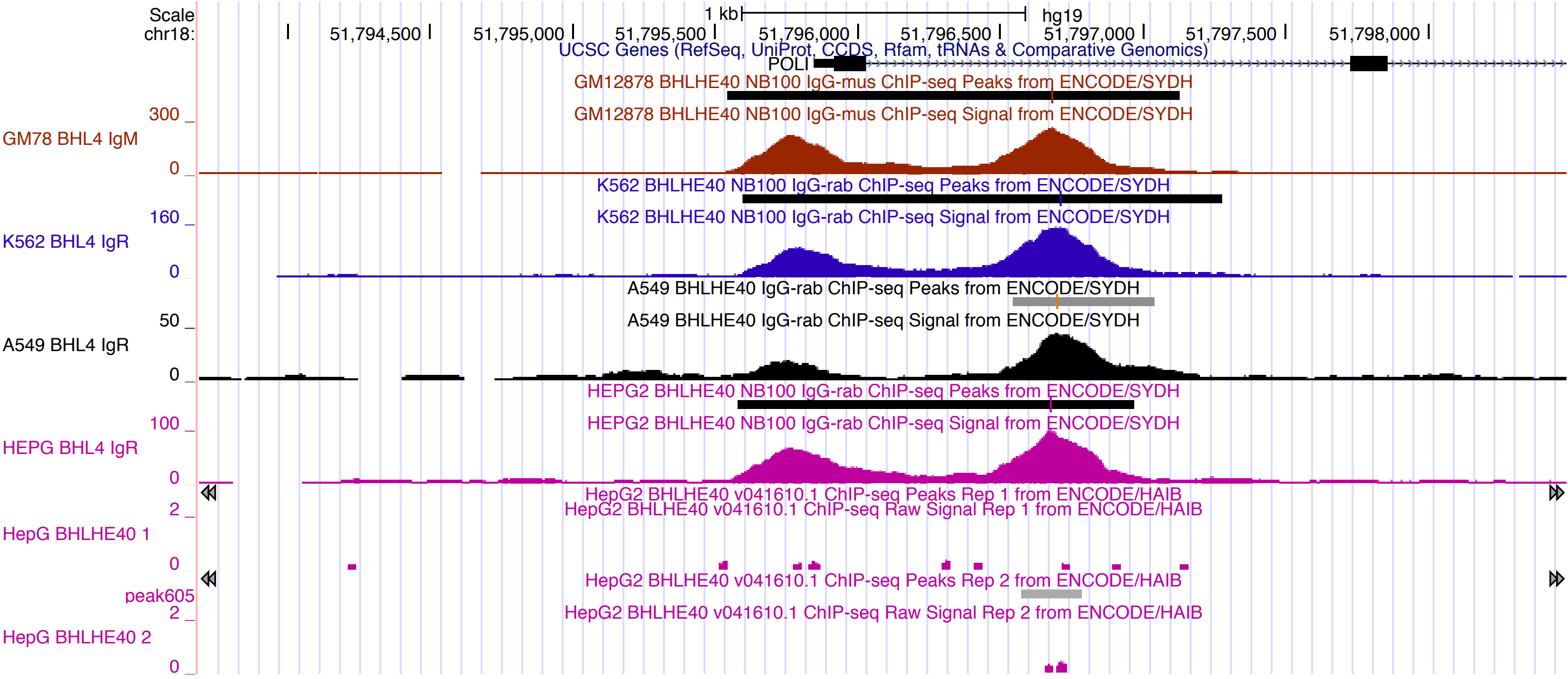
Scale
chr18:
1 kb
hg19
51,794,500
51,795,000
51,795,500
51,796,000
51,796,500
51,797,000
51,797,500
51,798,000
UCSC Genes (RefSeq, UniProt, CCDS, Rfam, tRNAs & Comparative Genomics)
POLI
GM12878 BHLHE40 NB100 IgG-mus ChIP-seq Peaks from ENCODE/SYDH
GM12878 BHLHE40 NB100 IgG-mus ChIP-seq Signal from ENCODE/SYDH
GM78 BHL4 IgM
300
0
K562 BHLHE40 NB100 IgG-rab ChIP-seq Peaks from ENCODE/SYDH
K562 BHLHE40 NB100 IgG-rab ChIP-seq Signal from ENCODE/SYDH
K562 BHL4 IgR
160
0
A549 BHLHE40 IgG-rab ChIP-seq Peaks from ENCODE/SYDH
A549 BHLHE40 IgG-rab ChIP-seq Signal from ENCODE/SYDH
A549 BHL4 IgR
50
0
HEPG2 BHLHE40 NB100 IgG-rab ChIP-seq Peaks from ENCODE/SYDH
HEPG2 BHLHE40 NB100 IgG-rab ChIP-seq Signal from ENCODE/SYDH
HEPG BHL4 IgR
100
0
HepG2 BHLHE40 v041610.1 ChIP-seq Peaks Rep 1 from ENCODE/HAIB
HepG2 BHLHE40 v041610.1 ChIP-seq Raw Signal Rep 1 from ENCODE/HAIB
HepG BHLHE40 1
2
0
HepG2 BHLHE40 v041610.1 ChIP-seq Peaks Rep 2 from ENCODE/HAIB
peak605
HepG2 BHLHE40 v041610.1 ChIP-seq Raw Signal Rep 2 from ENCODE/HAIB
HepG BHLHE40 2
2
0

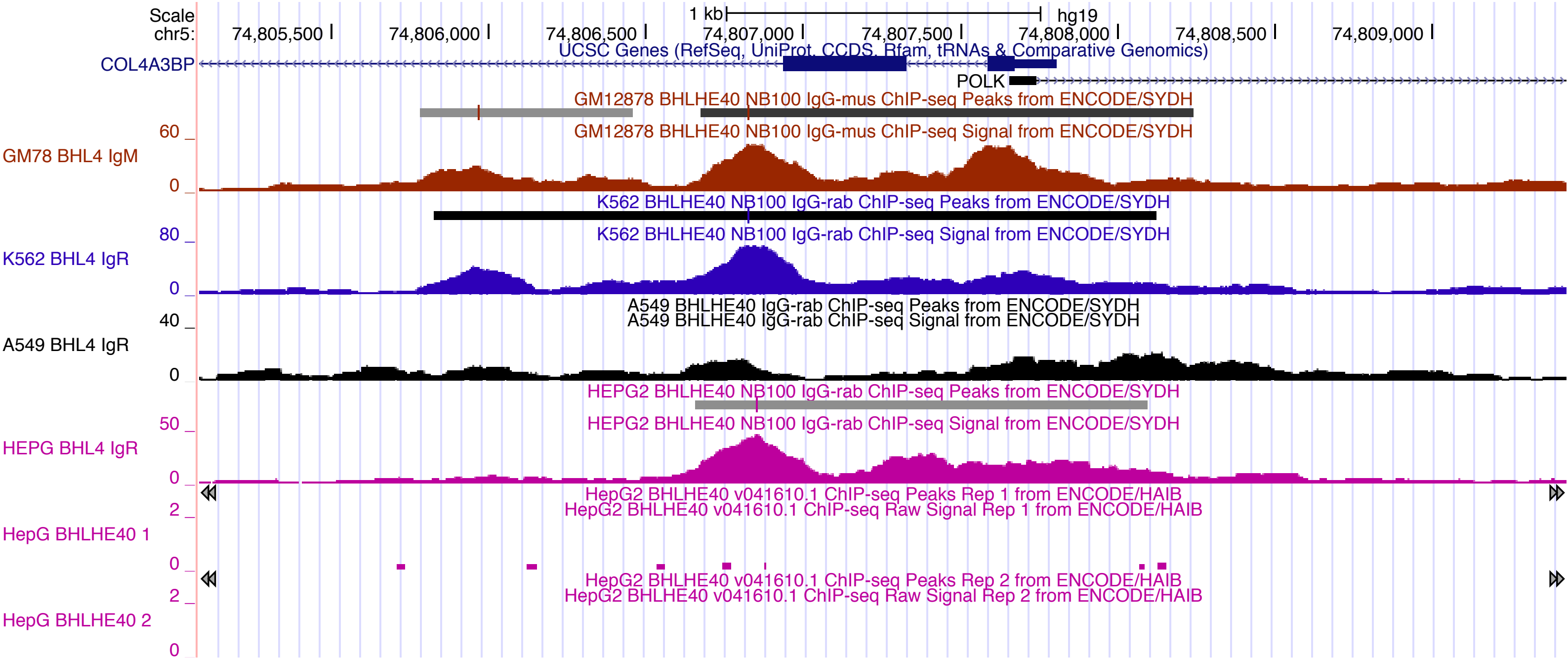

Scale
chr5:
1 kb
hg19
74,805,500
74,806,000
74,806,500
74,807,000
74,807,500
74,808,000
74,808,500
74,809,000
UCSC Genes (RefSeq, UniProt, CCDS, Rfam, tRNAs & Comparative Genomics)
COL4A3BP
POLK
GM12878 BHLHE40 NB100 IgG-mus ChIP-seq Peaks from ENCODE/SYDH
GM12878 BHLHE40 NB100 IgG-mus ChIP-seq Signal from ENCODE/SYDH
GM78 BHL4 IgM
60
0
K562 BHLHE40 NB100 IgG-rab ChIP-seq Peaks from ENCODE/SYDH
K562 BHLHE40 NB100 IgG-rab ChIP-seq Signal from ENCODE/SYDH
K562 BHL4 IgR
80
0
A549 BHLHE40 IgG-rab ChIP-seq Peaks from ENCODE/SYDH
A549 BHLHE40 IgG-rab ChIP-seq Signal from ENCODE/SYDH
A549 BHL4 IgR
40
0
HEPG2 BHLHE40 NB100 IgG-rab ChIP-seq Peaks from ENCODE/SYDH
HEPG2 BHLHE40 NB100 IgG-rab ChIP-seq Signal from ENCODE/SYDH
HEPG BHL4 IgR
50
0
HepG2 BHLHE40 v041610.1 ChIP-seq Peaks Rep 1 from ENCODE/HAIB
HepG2 BHLHE40 v041610.1 ChIP-seq Raw Signal Rep 1 from ENCODE/HAIB
HepG BHLHE40 1
2
0
HepG2 BHLHE40 v041610.1 ChIP-seq Peaks Rep 2 from ENCODE/HAIB
HepG2 BHLHE40 v041610.1 ChIP-seq Raw Signal Rep 2 from ENCODE/HAIB
HepG BHLHE40 2
2
0

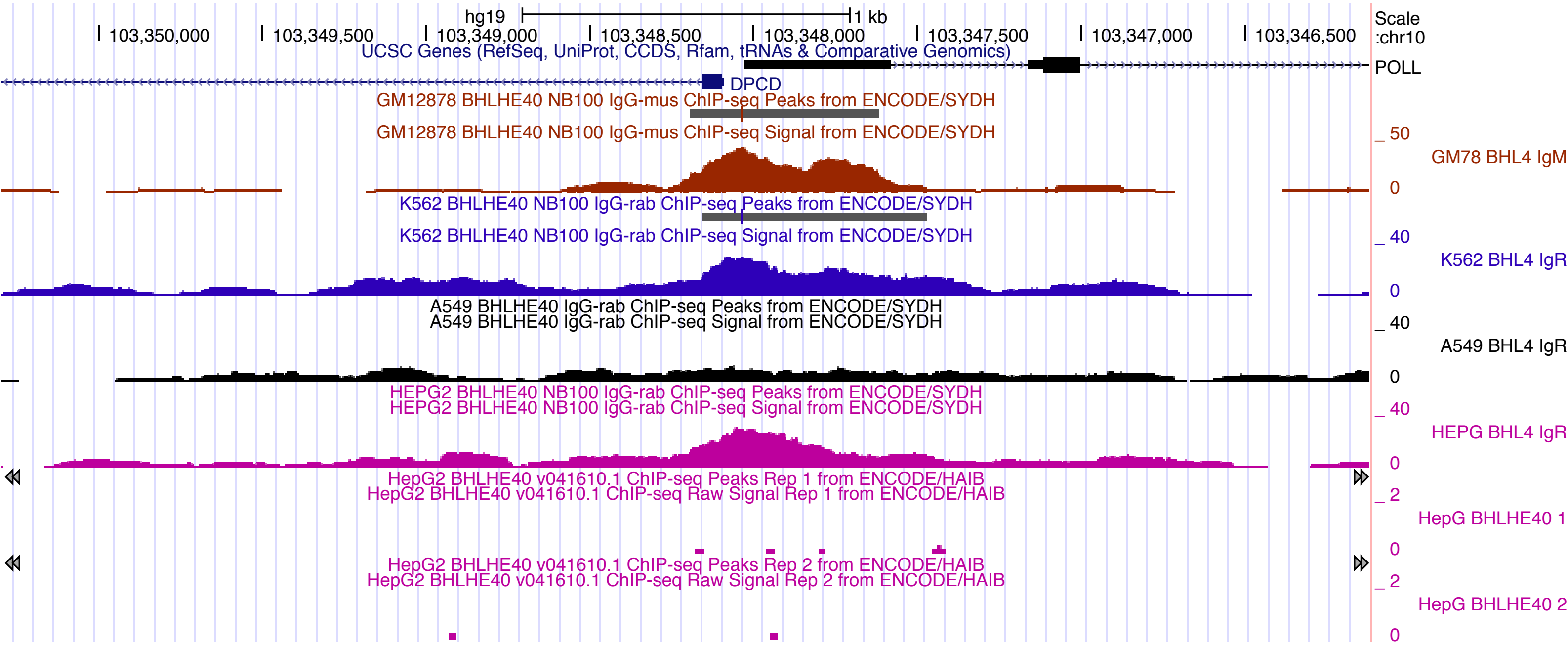

hg19
1 kb
Scale
:chr10
103,350,000
103,349,500
103,349,000
103,348,500
103,348,000
103,347,500
103,347,000
103,346,500
UCSC Genes (RefSeq, UniProt, CCDS, Rfam, tRNAs & Comparative Genomics)
POLL
DPCD
GM12878 BHLHE40 NB100 IgG-mus ChIP-seq Peaks from ENCODE/SYDH
GM12878 BHLHE40 NB100 IgG-mus ChIP-seq Signal from ENCODE/SYDH
50
GM78 BHL4 IgM
0
K562 BHLHE40 NB100 IgG-rab ChIP-seq Peaks from ENCODE/SYDH
K562 BHLHE40 NB100 IgG-rab ChIP-seq Signal from ENCODE/SYDH
40
K562 BHL4 IgR
0
A549 BHLHE40 IgG-rab ChIP-seq Peaks from ENCODE/SYDH
A549 BHLHE40 IgG-rab ChIP-seq Signal from ENCODE/SYDH
40
A549 BHL4 IgR
0
HEPG2 BHLHE40 NB100 IgG-rab ChIP-seq Peaks from ENCODE/SYDH
HEPG2 BHLHE40 NB100 IgG-rab ChIP-seq Signal from ENCODE/SYDH
40
HEPG BHL4 IgR
0
HepG2 BHLHE40 v041610.1 ChIP-seq Peaks Rep 1 from ENCODE/HAIB
HepG2 BHLHE40 v041610.1 ChIP-seq Raw Signal Rep 1 from ENCODE/HAIB
2
HepG BHLHE40 1
0
HepG2 BHLHE40 v041610.1 ChIP-seq Peaks Rep 2 from ENCODE/HAIB
HepG2 BHLHE40 v041610.1 ChIP-seq Raw Signal Rep 2 from ENCODE/HAIB
2
HepG BHLHE40 2
0

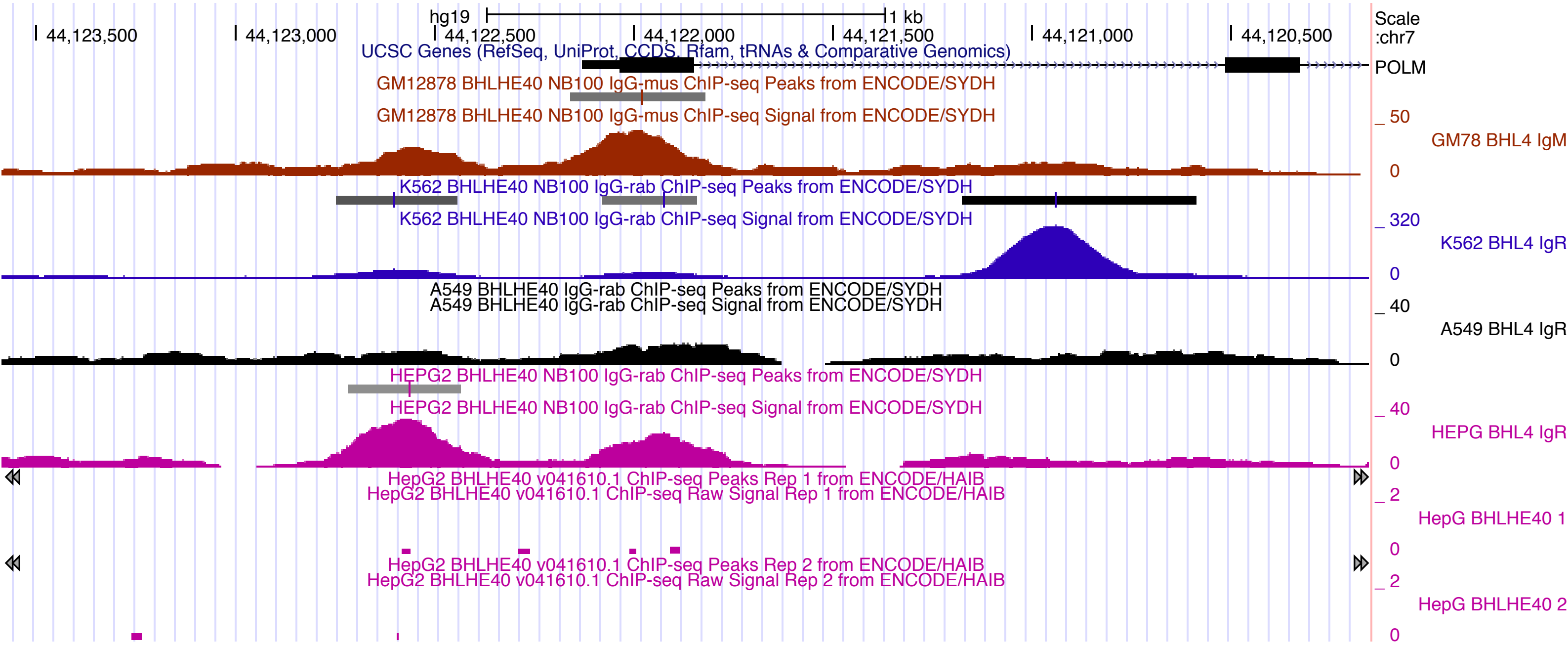

hg19
1 kb
Scale
:chr7
44,123,500
44,123,000
44,122,500
44,122,000
44,121,500
44,121,000
44,120,500
UCSC Genes (RefSeq, UniProt, CCDS, Rfam, tRNAs & Comparative Genomics)
POLM
GM12878 BHLHE40 NB100 IgG-mus ChIP-seq Peaks from ENCODE/SYDH
GM12878 BHLHE40 NB100 IgG-mus ChIP-seq Signal from ENCODE/SYDH
_ 50
GM78 BHL4 IgM
0
K562 BHLHE40 NB100 IgG-rab ChIP-seq Peaks from ENCODE/SYDH
K562 BHLHE40 NB100 IgG-rab ChIP-seq Signal from ENCODE/SYDH
_ 320
K562 BHL4 IgR
0
A549 BHLHE40 IgG-rab ChIP-seq Peaks from ENCODE/SYDH
A549 BHLHE40 IgG-rab ChIP-seq Signal from ENCODE/SYDH
_ 40
A549 BHL4 IgR
0
HEPG2 BHLHE40 NB100 IgG-rab ChIP-seq Peaks from ENCODE/SYDH
HEPG2 BHLHE40 NB100 IgG-rab ChIP-seq Signal from ENCODE/SYDH
_ 40
HEPG BHL4 IgR
0
HepG2 BHLHE40 v041610.1 ChIP-seq Peaks Rep 1 from ENCODE/HAIB
HepG2 BHLHE40 v041610.1 ChIP-seq Raw Signal Rep 1 from ENCODE/HAIB
_ 2
HepG BHLHE40 1
0
HepG2 BHLHE40 v041610.1 ChIP-seq Peaks Rep 2 from ENCODE/HAIB
HepG2 BHLHE40 v041610.1 ChIP-seq Raw Signal Rep 2 from ENCODE/HAIB
_ 2
HepG BHLHE40 2
0

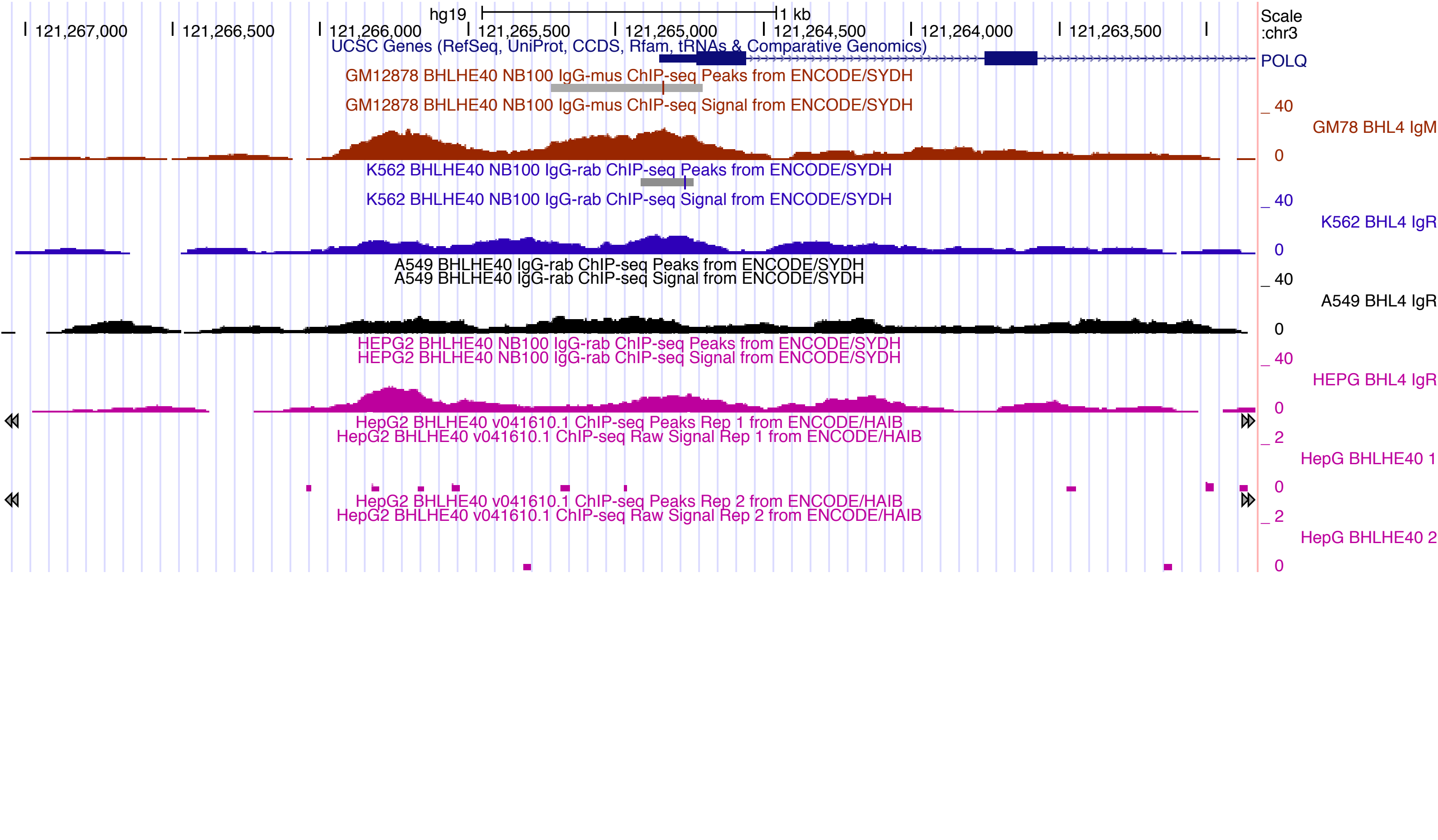

hg19
1 kb
Scale
:chr3
121,267,000
121,266,500
121,266,000
121,265,500
121,265,000
121,264,500
121,264,000
121,263,500
UCSC Genes (RefSeq, UniProt, CCDS, Rfam, tRNAs & Comparative Genomics)
POLQ
GM12878 BHLHE40 NB100 IgG-mus ChIP-seq Peaks from ENCODE/SYDH
GM12878 BHLHE40 NB100 IgG-mus ChIP-seq Signal from ENCODE/SYDH
GM78 BHL4 IgM
K562 BHLHE40 NB100 IgG-rab ChIP-seq Peaks from ENCODE/SYDH
K562 BHLHE40 NB100 IgG-rab ChIP-seq Signal from ENCODE/SYDH
K562 BHL4 IgR
A549 BHLHE40 IgG-rab ChIP-seq Peaks from ENCODE/SYDH
A549 BHLHE40 IgG-rab ChIP-seq Signal from ENCODE/SYDH
A549 BHL4 IgR
HEPG2 BHLHE40 NB100 IgG-rab ChIP-seq Peaks from ENCODE/SYDH
HEPG2 BHLHE40 NB100 IgG-rab ChIP-seq Signal from ENCODE/SYDH
HEPG BHL4 IgR
HepG2 BHLHE40 v041610.1 ChIP-seq Peaks Rep 1 from ENCODE/HAIB
HepG2 BHLHE40 v041610.1 ChIP-seq Raw Signal Rep 1 from ENCODE/HAIB
HepG BHLHE40 1
HepG2 BHLHE40 v041610.1 ChIP-seq Peaks Rep 2 from ENCODE/HAIB
HepG2 BHLHE40 v041610.1 ChIP-seq Raw Signal Rep 2 from ENCODE/HAIB
HepG BHLHE40 2

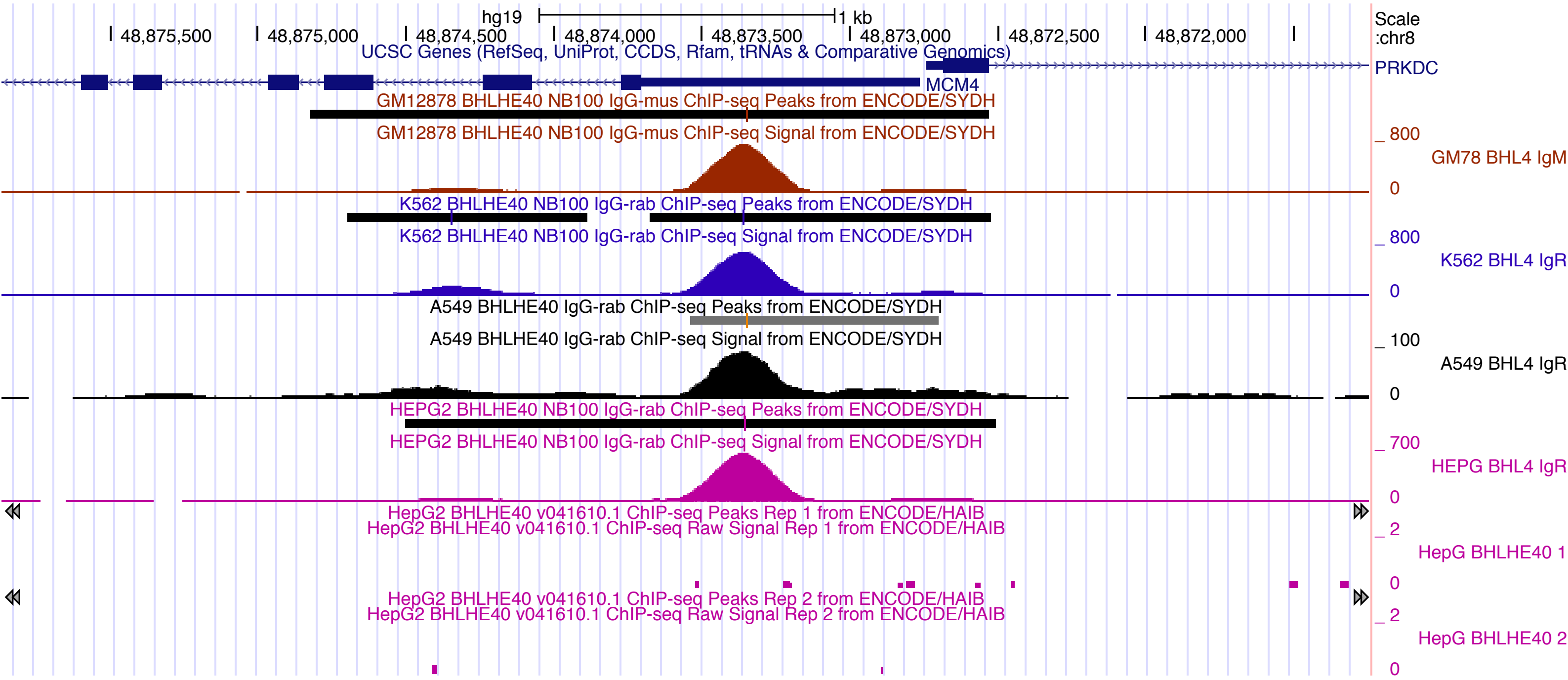

hg19
1 kb
Scale
:chr8
48,875,500
48,875,000
48,874,500
48,874,000
48,873,500
48,873,000
48,872,500
48,872,000
UCSC Genes (RefSeq, UniProt, CCDS, Rfam, tRNAs & Comparative Genomics)
PRKDC
MCM4
GM12878 BHLHE40 NB100 IgG-mus ChIP-seq Peaks from ENCODE/SYDH
GM12878 BHLHE40 NB100 IgG-mus ChIP-seq Signal from ENCODE/SYDH
800
GM78 BHL4 IgM
0
K562 BHLHE40 NB100 IgG-rab ChIP-seq Peaks from ENCODE/SYDH
K562 BHLHE40 NB100 IgG-rab ChIP-seq Signal from ENCODE/SYDH
800
K562 BHL4 IgR
0
A549 BHLHE40 IgG-rab ChIP-seq Peaks from ENCODE/SYDH
A549 BHLHE40 IgG-rab ChIP-seq Signal from ENCODE/SYDH
100
A549 BHL4 IgR
0
HEPG2 BHLHE40 NB100 IgG-rab ChIP-seq Peaks from ENCODE/SYDH
HEPG2 BHLHE40 NB100 IgG-rab ChIP-seq Signal from ENCODE/SYDH
700
HEPG BHL4 IgR
0
HepG2 BHLHE40 v041610.1 ChIP-seq Peaks Rep 1 from ENCODE/HAIB
HepG2 BHLHE40 v041610.1 ChIP-seq Raw Signal Rep 1 from ENCODE/HAIB
2
HepG BHLHE40 1
0
HepG2 BHLHE40 v041610.1 ChIP-seq Peaks Rep 2 from ENCODE/HAIB
HepG2 BHLHE40 v041610.1 ChIP-seq Raw Signal Rep 2 from ENCODE/HAIB
2
HepG BHLHE40 2
0

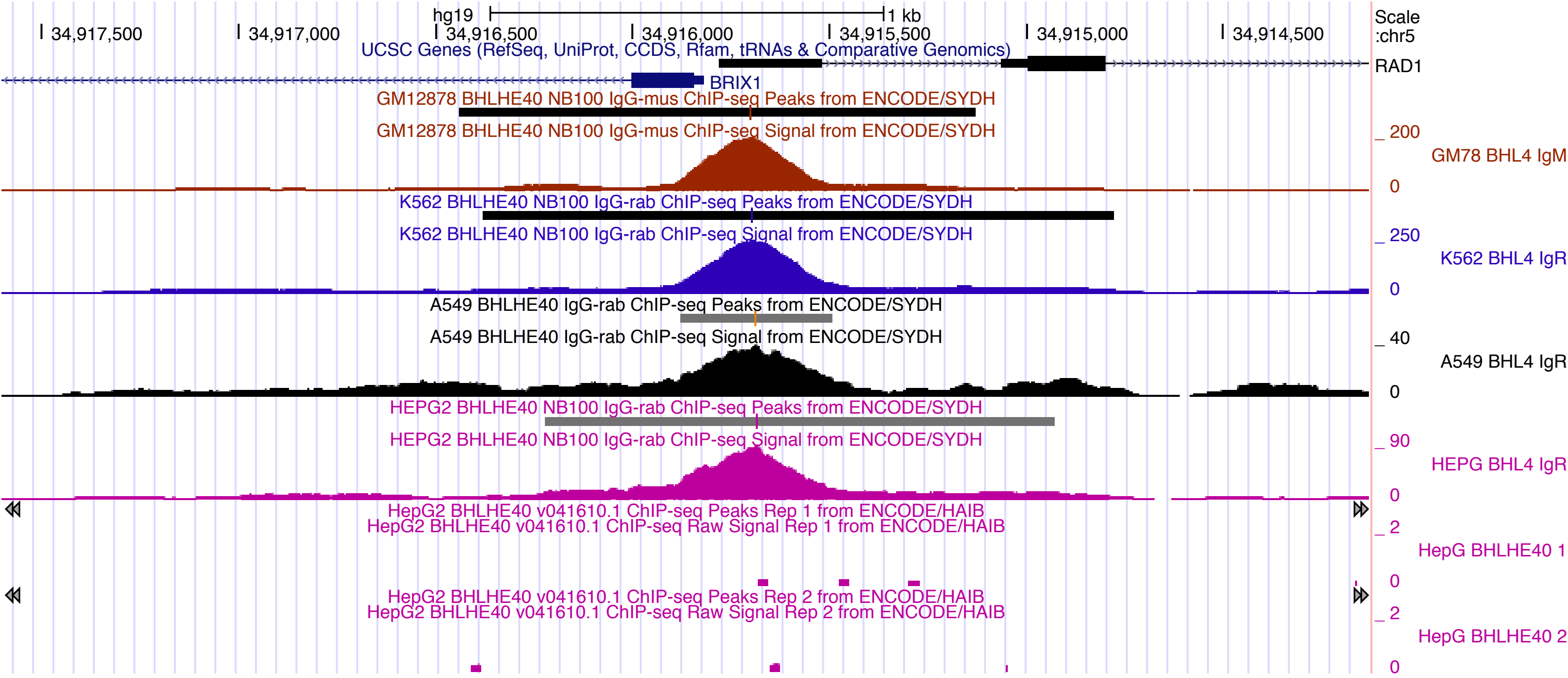

hg19
1 kb
Scale
:chr5
34,917,500
34,917,000
34,916,500
34,916,000
34,915,500
34,915,000
34,914,500
UCSC Genes (RefSeq, UniProt, CCDS, Rfam, tRNAs & Comparative Genomics)
RAD1
BRIX1
GM12878 BHLHE40 NB100 IgG-mus ChIP-seq Peaks from ENCODE/SYDH
GM12878 BHLHE40 NB100 IgG-mus ChIP-seq Signal from ENCODE/SYDH
200
GM78 BHL4 IgM
0
K562 BHLHE40 NB100 IgG-rab ChIP-seq Peaks from ENCODE/SYDH
K562 BHLHE40 NB100 IgG-rab ChIP-seq Signal from ENCODE/SYDH
250
K562 BHL4 IgR
0
A549 BHLHE40 IgG-rab ChIP-seq Peaks from ENCODE/SYDH
A549 BHLHE40 IgG-rab ChIP-seq Signal from ENCODE/SYDH
40
A549 BHL4 IgR
0
HEPG2 BHLHE40 NB100 IgG-rab ChIP-seq Peaks from ENCODE/SYDH
HEPG2 BHLHE40 NB100 IgG-rab ChIP-seq Signal from ENCODE/SYDH
90
HEPG BHL4 IgR
0
HepG2 BHLHE40 v041610.1 ChIP-seq Peaks Rep 1 from ENCODE/HAIB
HepG2 BHLHE40 v041610.1 ChIP-seq Raw Signal Rep 1 from ENCODE/HAIB
2
HepG BHLHE40 1
0
HepG2 BHLHE40 v041610.1 ChIP-seq Peaks Rep 2 from ENCODE/HAIB
HepG2 BHLHE40 v041610.1 ChIP-seq Raw Signal Rep 2 from ENCODE/HAIB
2
HepG BHLHE40 2
0

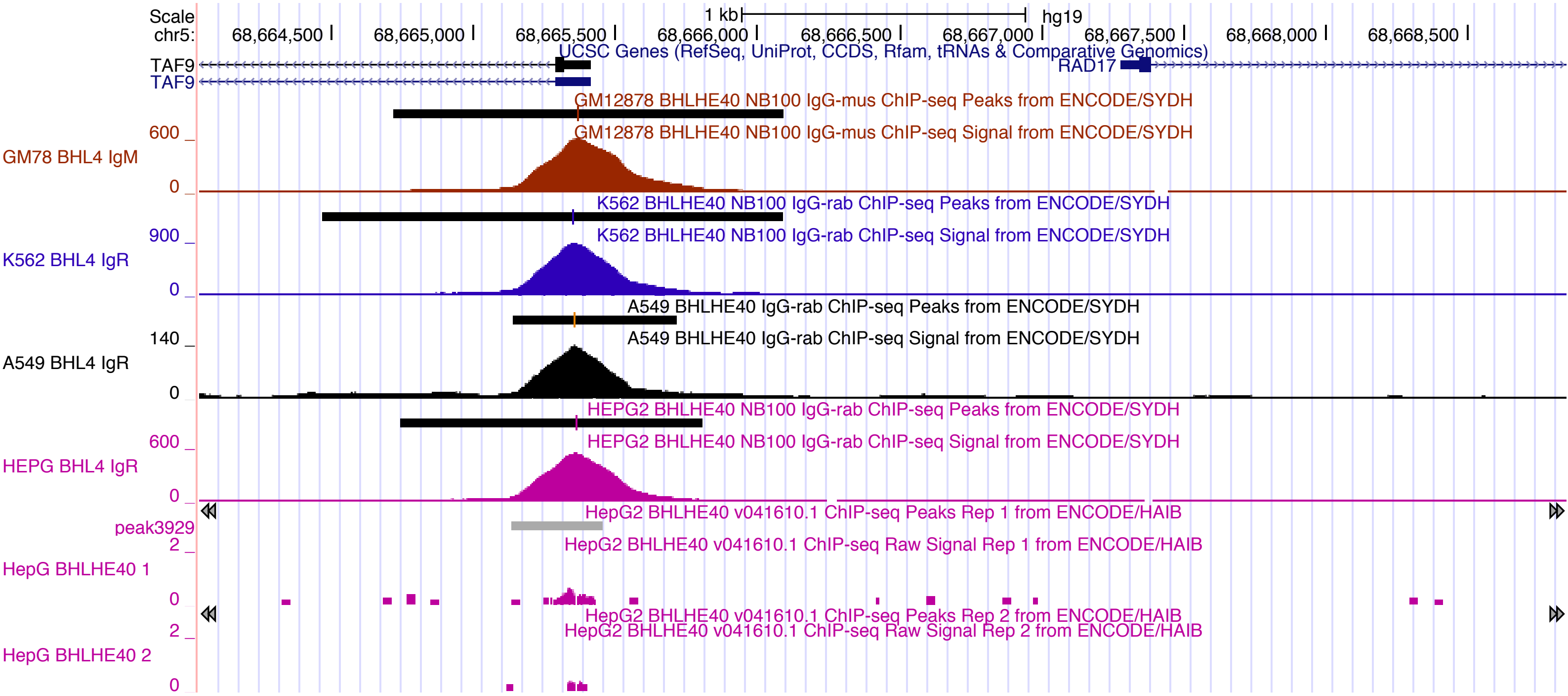

Scale
chr5:
1 kb
hg19
68,664,500
68,665,000
68,665,500
68,666,000
68,666,500
68,667,000
68,667,500
68,668,000
68,668,500
UCSC Genes (RefSeq, UniProt, CCDS, Rfam, tRNAs & Comparative Genomics)
TAF9
TAF9
RAD17
GM12878 BHLHE40 NB100 IgG-mus ChIP-seq Peaks from ENCODE/SYDH
GM12878 BHLHE40 NB100 IgG-mus ChIP-seq Signal from ENCODE/SYDH
GM78 BHL4 IgM
600
0
K562 BHLHE40 NB100 IgG-rab ChIP-seq Peaks from ENCODE/SYDH
K562 BHLHE40 NB100 IgG-rab ChIP-seq Signal from ENCODE/SYDH
K562 BHL4 IgR
900
0
A549 BHLHE40 IgG-rab ChIP-seq Peaks from ENCODE/SYDH
A549 BHLHE40 IgG-rab ChIP-seq Signal from ENCODE/SYDH
A549 BHL4 IgR
140
0
HEPG2 BHLHE40 NB100 IgG-rab ChIP-seq Peaks from ENCODE/SYDH
HEPG2 BHLHE40 NB100 IgG-rab ChIP-seq Signal from ENCODE/SYDH
HEPG BHL4 IgR
600
0
HepG2 BHLHE40 v041610.1 ChIP-seq Peaks Rep 1 from ENCODE/HAIB
peak3929
HepG2 BHLHE40 v041610.1 ChIP-seq Raw Signal Rep 1 from ENCODE/HAIB
HepG BHLHE40 1
2
0
HepG2 BHLHE40 v041610.1 ChIP-seq Peaks Rep 2 from ENCODE/HAIB
HepG2 BHLHE40 v041610.1 ChIP-seq Raw Signal Rep 2 from ENCODE/HAIB
HepG BHLHE40 2
2
0

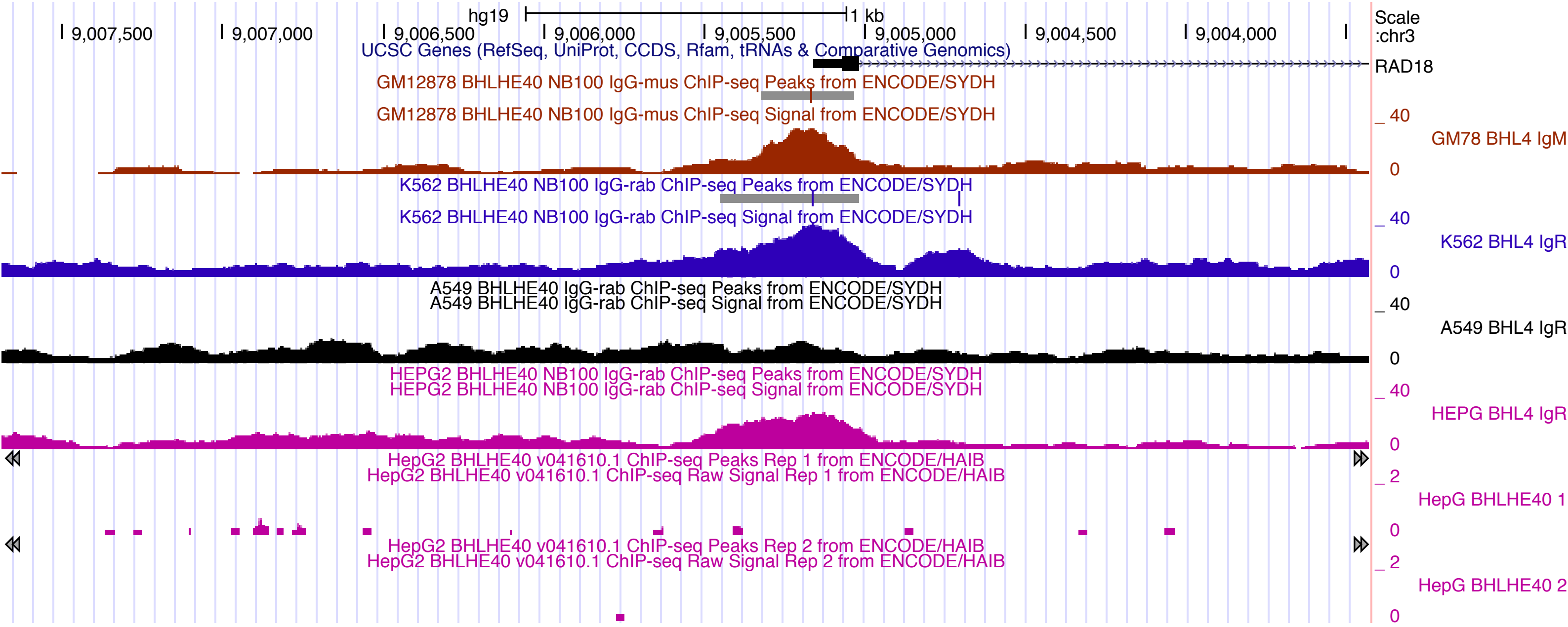

hg19
1 kb
Scale
:chr3
9,007,500
9,007,000
9,006,500
9,006,000
9,005,500
9,005,000
9,004,500
9,004,000
UCSC Genes (RefSeq, UniProt, CCDS, Rfam, tRNAs & Comparative Genomics)
RAD18
GM12878 BHLHE40 NB100 IgG-mus ChIP-seq Peaks from ENCODE/SYDH
GM12878 BHLHE40 NB100 IgG-mus ChIP-seq Signal from ENCODE/SYDH
GM78 BHL4 IgM
K562 BHLHE40 NB100 IgG-rab ChIP-seq Peaks from ENCODE/SYDH
K562 BHLHE40 NB100 IgG-rab ChIP-seq Signal from ENCODE/SYDH
K562 BHL4 IgR
A549 BHLHE40 IgG-rab ChIP-seq Peaks from ENCODE/SYDH
A549 BHLHE40 IgG-rab ChIP-seq Signal from ENCODE/SYDH
A549 BHL4 IgR
HEPG2 BHLHE40 NB100 IgG-rab ChIP-seq Peaks from ENCODE/SYDH
HEPG2 BHLHE40 NB100 IgG-rab ChIP-seq Signal from ENCODE/SYDH
HEPG BHL4 IgR
HepG2 BHLHE40 v041610.1 ChIP-seq Peaks Rep 1 from ENCODE/HAIB
HepG2 BHLHE40 v041610.1 ChIP-seq Raw Signal Rep 1 from ENCODE/HAIB
HepG BHLHE40 1
HepG2 BHLHE40 v041610.1 ChIP-seq Peaks Rep 2 from ENCODE/HAIB
HepG2 BHLHE40 v041610.1 ChIP-seq Raw Signal Rep 2 from ENCODE/HAIB
HepG BHLHE40 2
40
0
2

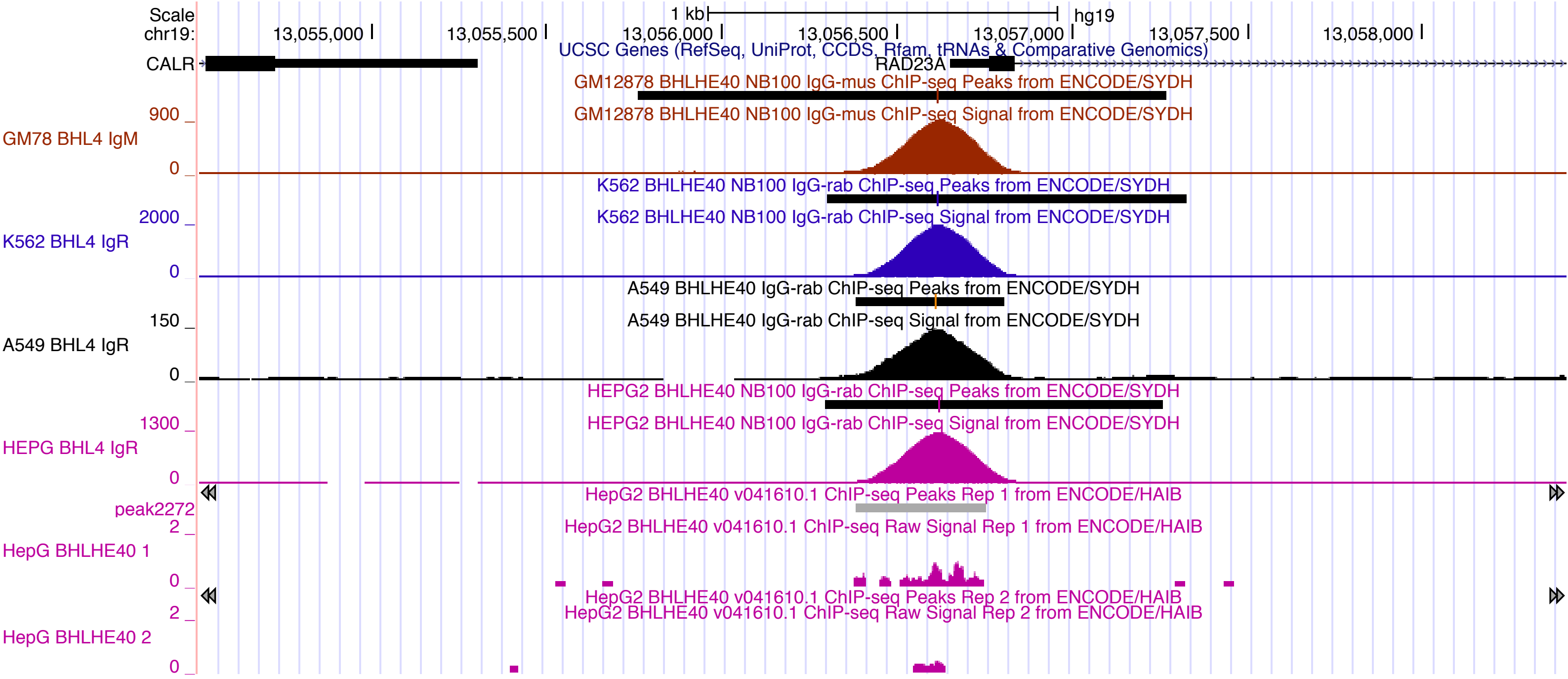
Scale
chr19:
1 kb
hg19
13,055,000
13,055,500
13,056,000
13,056,500
13,057,000
13,057,500
13,058,000
UCSC Genes (RefSeq, UniProt, CCDS, Rfam, tRNAs & Comparative Genomics)
CALR
RAD23A
GM12878 BHLHE40 NB100 IgG-mus ChIP-seq Peaks from ENCODE/SYDH
GM12878 BHLHE40 NB100 IgG-mus ChIP-seq Signal from ENCODE/SYDH
900
GM78 BHL4 IgM
0
K562 BHLHE40 NB100 IgG-rab ChIP-seq Peaks from ENCODE/SYDH
K562 BHLHE40 NB100 IgG-rab ChIP-seq Signal from ENCODE/SYDH
2000
K562 BHL4 IgR
0
A549 BHLHE40 IgG-rab ChIP-seq Peaks from ENCODE/SYDH
A549 BHLHE40 IgG-rab ChIP-seq Signal from ENCODE/SYDH
150
A549 BHL4 IgR
0
HEPG2 BHLHE40 NB100 IgG-rab ChIP-seq Peaks from ENCODE/SYDH
HEPG2 BHLHE40 NB100 IgG-rab ChIP-seq Signal from ENCODE/SYDH
1300
HEPG BHL4 IgR
0
HepG2 BHLHE40 v041610.1 ChIP-seq Peaks Rep 1 from ENCODE/HAIB
peak2272
HepG2 BHLHE40 v041610.1 ChIP-seq Raw Signal Rep 1 from ENCODE/HAIB
2
HepG BHLHE40 1
0
HepG2 BHLHE40 v041610.1 ChIP-seq Peaks Rep 2 from ENCODE/HAIB
HepG2 BHLHE40 v041610.1 ChIP-seq Raw Signal Rep 2 from ENCODE/HAIB
2
HepG BHLHE40 2
0

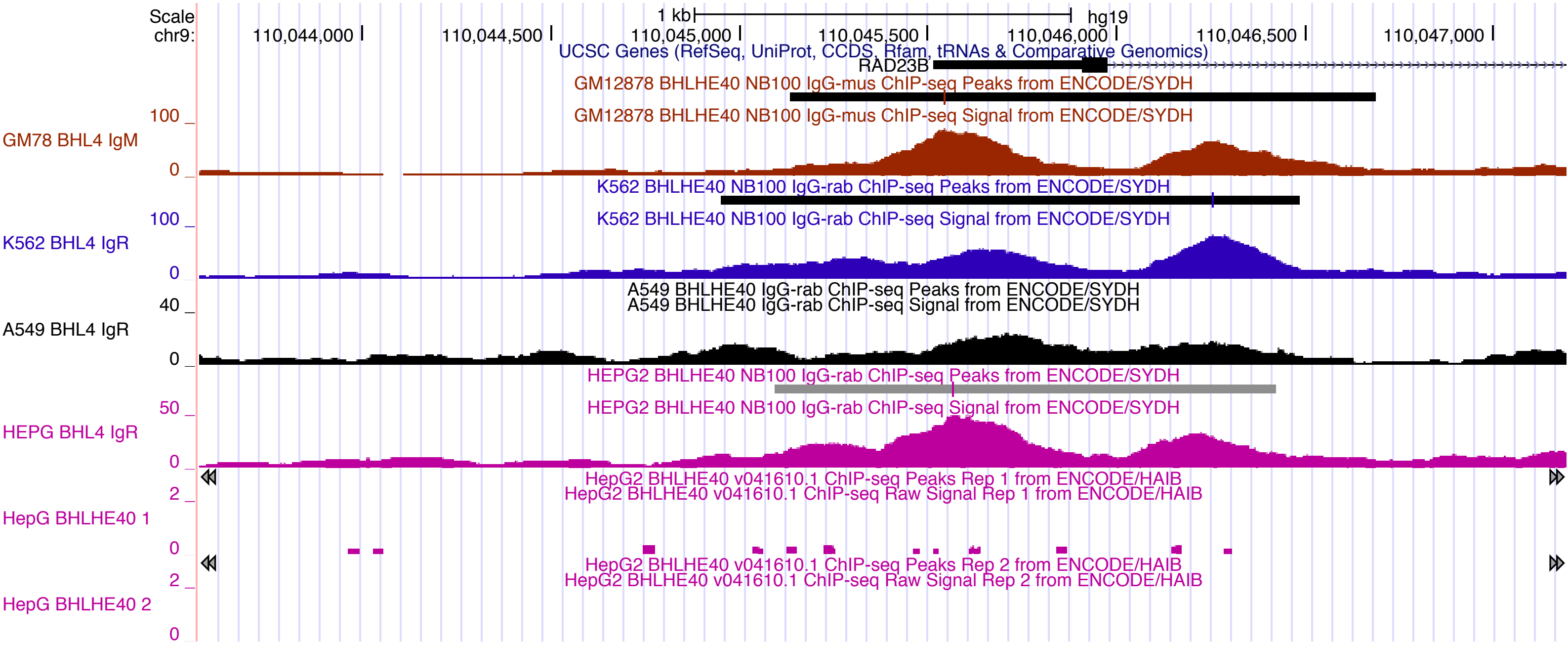

Scale
chr9:
1 kb
hg19
110,044,000
110,044,500
110,045,000
110,045,500
110,046,000
110,046,500
110,047,000
UCSC Genes (RefSeq, UniProt, CCDS, Rfam, tRNAs & Comparative Genomics)
RAD23B
GM12878 BHLHE40 NB100 IgG-mus ChIP-seq Peaks from ENCODE/SYDH
GM12878 BHLHE40 NB100 IgG-mus ChIP-seq Signal from ENCODE/SYDH
GM78 BHL4 IgM
100 _
0 _
K562 BHLHE40 NB100 IgG-rab ChIP-seq Peaks from ENCODE/SYDH
K562 BHLHE40 NB100 IgG-rab ChIP-seq Signal from ENCODE/SYDH
K562 BHL4 IgR
100 _
0
A549 BHLHE40 IgG-rab ChIP-seq Peaks from ENCODE/SYDH
A549 BHLHE40 IgG-rab ChIP-seq Signal from ENCODE/SYDH
A549 BHL4 IgR
40 _
0 _
HEPG2 BHLHE40 NB100 IgG-rab ChIP-seq Peaks from ENCODE/SYDH
HEPG2 BHLHE40 NB100 IgG-rab ChIP-seq Signal from ENCODE/SYDH
HEPG BHL4 IgR
50 _
0 _
HepG2 BHLHE40 v041610.1 ChIP-seq Peaks Rep 1 from ENCODE/HAIB
HepG2 BHLHE40 v041610.1 ChIP-seq Raw Signal Rep 1 from ENCODE/HAIB
HepG BHLHE40 1
2 _
0
HepG2 BHLHE40 v041610.1 ChIP-seq Peaks Rep 2 from ENCODE/HAIB
HepG2 BHLHE40 v041610.1 ChIP-seq Raw Signal Rep 2 from ENCODE/HAIB
HepG BHLHE40 2
2 _
0

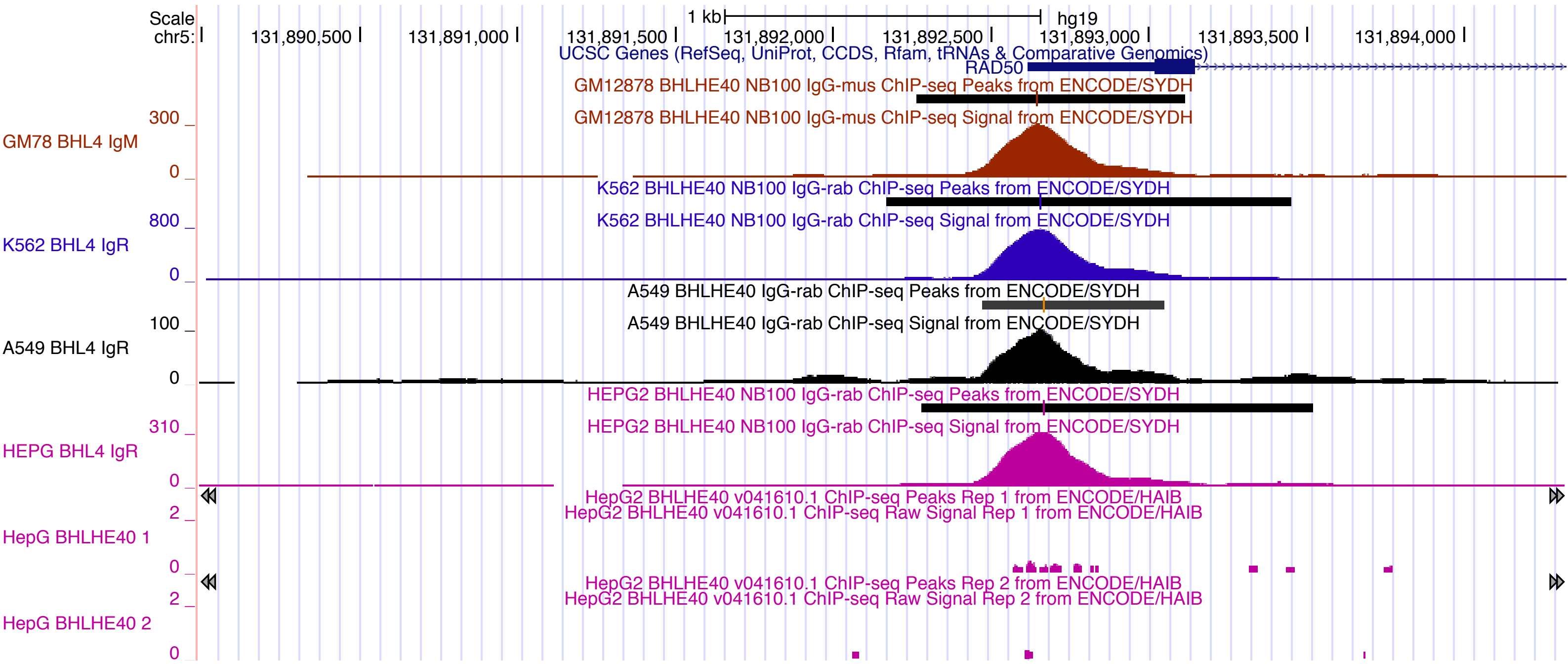

Scale
chr5:
1 kb
hg19
131,890,500
131,891,000
131,891,500
131,892,000
131,892,500
131,893,000
131,893,500
131,894,000
UCSC Genes (RefSeq, UniProt, CCDS, Rfam, tRNAs & Comparative Genomics)
RAD50
GM12878 BHLHE40 NB100 IgG-mus ChIP-seq Peaks from ENCODE/SYDH
GM12878 BHLHE40 NB100 IgG-mus ChIP-seq Signal from ENCODE/SYDH
300
GM78 BHL4 IgM
0
K562 BHLHE40 NB100 IgG-rab ChIP-seq Peaks from ENCODE/SYDH
K562 BHLHE40 NB100 IgG-rab ChIP-seq Signal from ENCODE/SYDH
800
K562 BHL4 IgR
0
A549 BHLHE40 IgG-rab ChIP-seq Peaks from ENCODE/SYDH
A549 BHLHE40 IgG-rab ChIP-seq Signal from ENCODE/SYDH
100
A549 BHL4 IgR
0
HEPG2 BHLHE40 NB100 IgG-rab ChIP-seq Peaks from ENCODE/SYDH
HEPG2 BHLHE40 NB100 IgG-rab ChIP-seq Signal from ENCODE/SYDH
310
HEPG BHL4 IgR
0
HepG2 BHLHE40 v041610.1 ChIP-seq Peaks Rep 1 from ENCODE/HAIB
HepG2 BHLHE40 v041610.1 ChIP-seq Raw Signal Rep 1 from ENCODE/HAIB
2
HepG BHLHE40 1
0
HepG2 BHLHE40 v041610.1 ChIP-seq Peaks Rep 2 from ENCODE/HAIB
HepG2 BHLHE40 v041610.1 ChIP-seq Raw Signal Rep 2 from ENCODE/HAIB
2
HepG BHLHE40 2
0

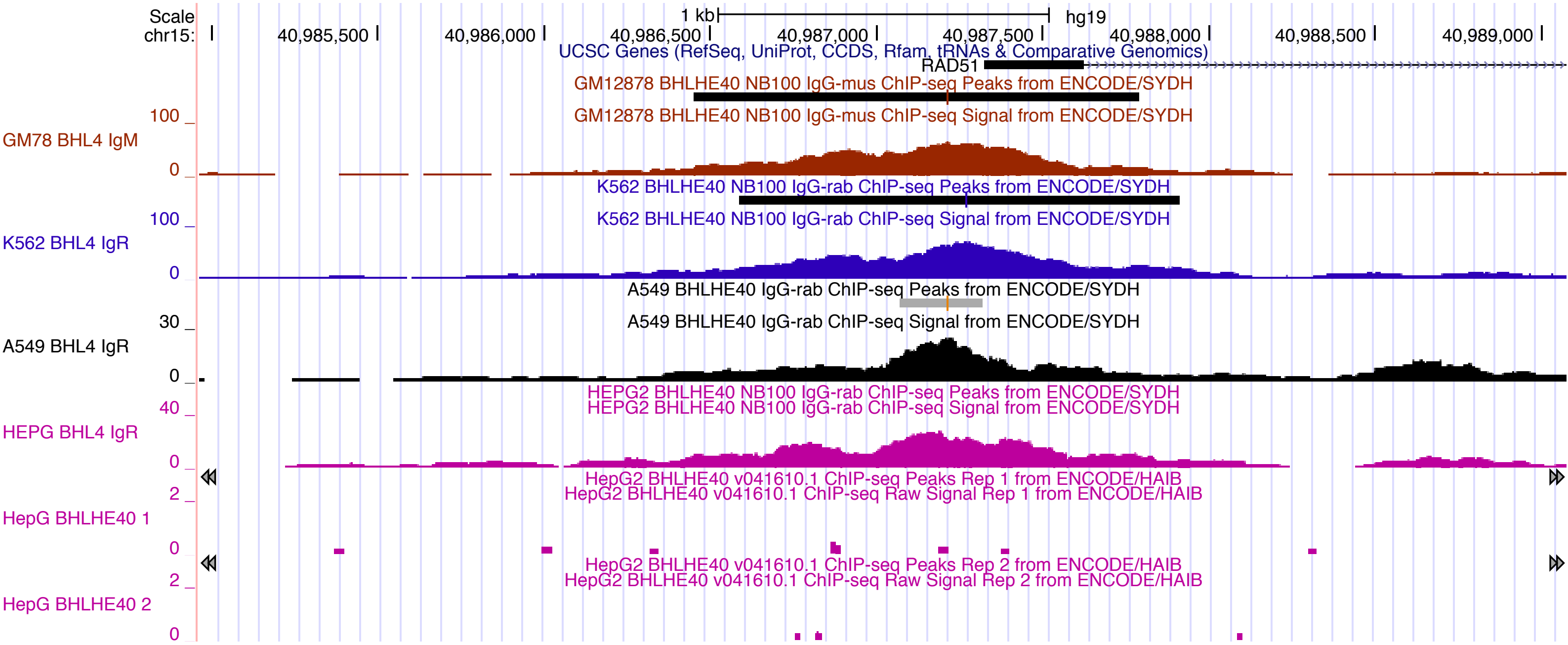

Scale
chr15:
1 kb
hg19
40,985,500
40,986,000
40,986,500
40,987,000
40,987,500
40,988,000
40,988,500
40,989,000
UCSC Genes (RefSeq, UniProt, CCDS, Rfam, tRNAs & Comparative Genomics)
RAD51
GM12878 BHLHE40 NB100 IgG-mus ChIP-seq Peaks from ENCODE/SYDH
GM12878 BHLHE40 NB100 IgG-mus ChIP-seq Signal from ENCODE/SYDH
GM78 BHL4 IgM
100
0
K562 BHLHE40 NB100 IgG-rab ChIP-seq Peaks from ENCODE/SYDH
K562 BHLHE40 NB100 IgG-rab ChIP-seq Signal from ENCODE/SYDH
K562 BHL4 IgR
100
0
A549 BHLHE40 IgG-rab ChIP-seq Peaks from ENCODE/SYDH
A549 BHLHE40 IgG-rab ChIP-seq Signal from ENCODE/SYDH
A549 BHL4 IgR
30
0
HEPG2 BHLHE40 NB100 IgG-rab ChIP-seq Peaks from ENCODE/SYDH
HEPG2 BHLHE40 NB100 IgG-rab ChIP-seq Signal from ENCODE/SYDH
HEPG BHL4 IgR
40
0
HepG2 BHLHE40 v041610.1 ChIP-seq Peaks Rep 1 from ENCODE/HAIB
HepG2 BHLHE40 v041610.1 ChIP-seq Raw Signal Rep 1 from ENCODE/HAIB
HepG BHLHE40 1
2
0
HepG2 BHLHE40 v041610.1 ChIP-seq Peaks Rep 2 from ENCODE/HAIB
HepG2 BHLHE40 v041610.1 ChIP-seq Raw Signal Rep 2 from ENCODE/HAIB
HepG BHLHE40 2
2
0

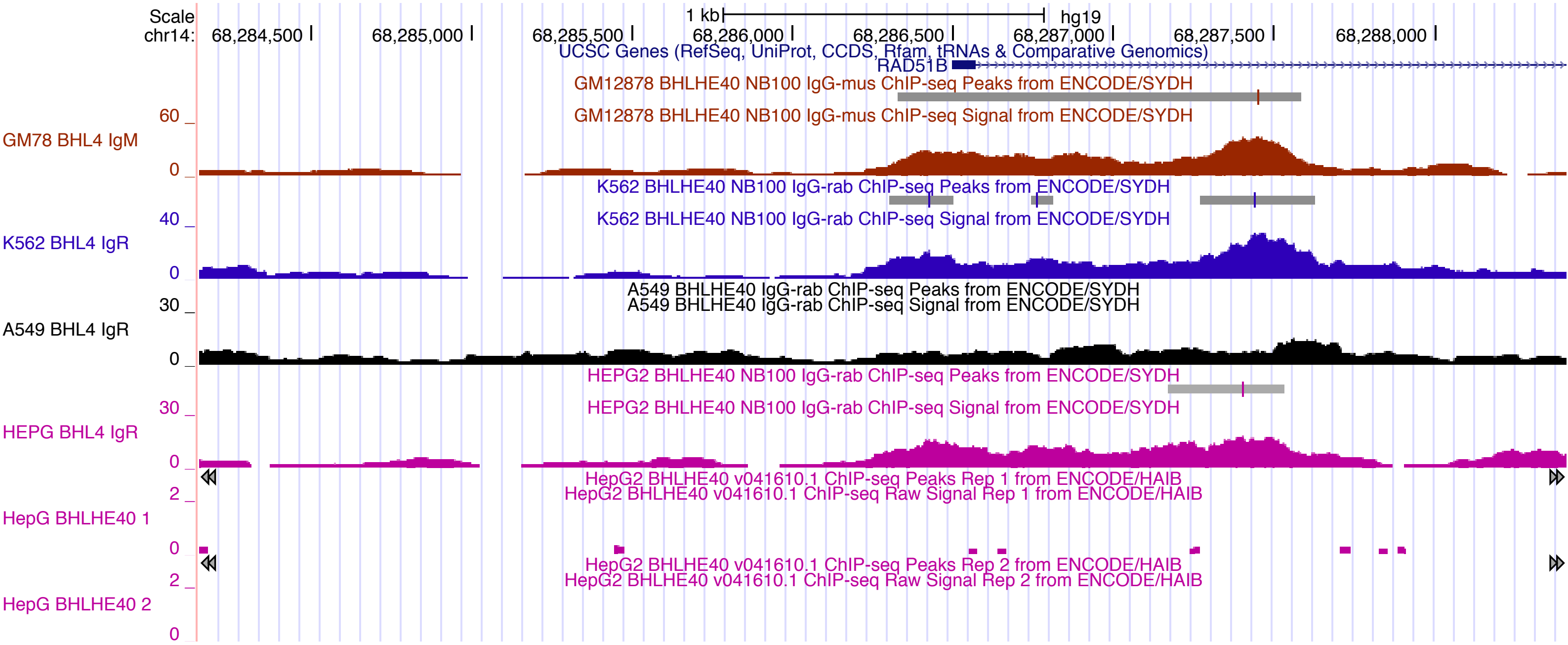

Scale
chr14:
1 kb
hg19
68,284,500
68,285,000
68,285,500
68,286,000
68,286,500
68,287,000
68,287,500
68,288,000
UCSC Genes (RefSeq, UniProt, CCDS, Rfam, tRNAs & Comparative Genomics)
RAD51B
GM12878 BHLHE40 NB100 IgG-mus ChIP-seq Peaks from ENCODE/SYDH
GM12878 BHLHE40 NB100 IgG-mus ChIP-seq Signal from ENCODE/SYDH
60
GM78 BHL4 IgM
0
K562 BHLHE40 NB100 IgG-rab ChIP-seq Peaks from ENCODE/SYDH
K562 BHLHE40 NB100 IgG-rab ChIP-seq Signal from ENCODE/SYDH
40
K562 BHL4 IgR
0
A549 BHLHE40 IgG-rab ChIP-seq Peaks from ENCODE/SYDH
A549 BHLHE40 IgG-rab ChIP-seq Signal from ENCODE/SYDH
30
A549 BHL4 IgR
0
HEPG2 BHLHE40 NB100 IgG-rab ChIP-seq Peaks from ENCODE/SYDH
HEPG2 BHLHE40 NB100 IgG-rab ChIP-seq Signal from ENCODE/SYDH
30
HEPG BHL4 IgR
0
HepG2 BHLHE40 v041610.1 ChIP-seq Peaks Rep 1 from ENCODE/HAIB
HepG2 BHLHE40 v041610.1 ChIP-seq Raw Signal Rep 1 from ENCODE/HAIB
2
HepG BHLHE40 1
0
HepG2 BHLHE40 v041610.1 ChIP-seq Peaks Rep 2 from ENCODE/HAIB
HepG2 BHLHE40 v041610.1 ChIP-seq Raw Signal Rep 2 from ENCODE/HAIB
2
HepG BHLHE40 2
0

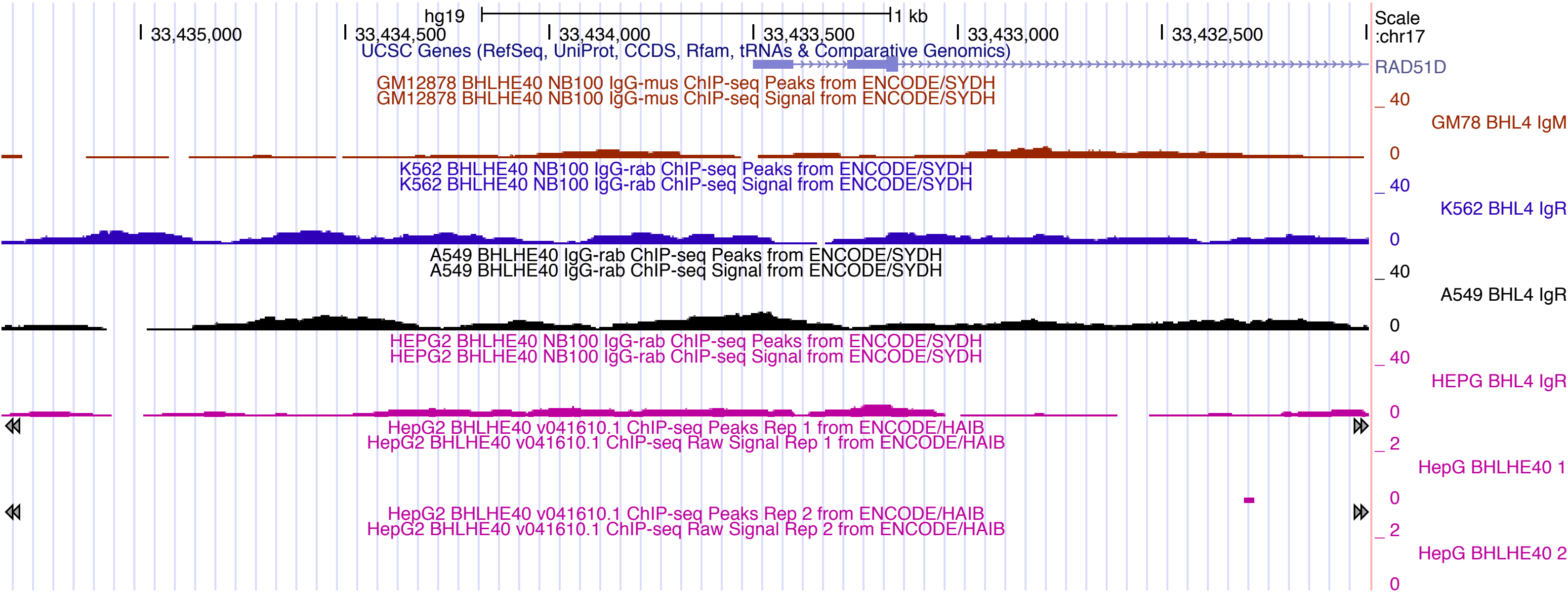

hg19
1 kb
Scale
chr17:
33,435,000
33,434,500
33,434,000
33,433,500
33,433,000
33,432,500
UCSC Genes (RefSeq, UniProt, CCDS, Rfam, tRNAs & Comparative Genomics)
RAD51D
GM12878 BHLHE40 NB100 IgG-mus ChIP-seq Peaks from ENCODE/SYDH
GM12878 BHLHE40 NB100 IgG-mus ChIP-seq Signal from ENCODE/SYDH
GM78 BHL4 IgM
K562 BHLHE40 NB100 IgG-rab ChIP-seq Peaks from ENCODE/SYDH
K562 BHLHE40 NB100 IgG-rab ChIP-seq Signal from ENCODE/SYDH
K562 BHL4 IgR
A549 BHLHE40 IgG-rab ChIP-seq Peaks from ENCODE/SYDH
A549 BHLHE40 IgG-rab ChIP-seq Signal from ENCODE/SYDH
A549 BHL4 IgR
HEPG2 BHLHE40 NB100 IgG-rab ChIP-seq Peaks from ENCODE/SYDH
HEPG2 BHLHE40 NB100 IgG-rab ChIP-seq Signal from ENCODE/SYDH
HEPG BHL4 IgR
HepG2 BHLHE40 v041610.1 ChIP-seq Peaks Rep 1 from ENCODE/HAIB
HepG2 BHLHE40 v041610.1 ChIP-seq Raw Signal Rep 1 from ENCODE/HAIB
HepG BHLHE40 1
HepG2 BHLHE40 v041610.1 ChIP-seq Peaks Rep 2 from ENCODE/HAIB
HepG2 BHLHE40 v041610.1 ChIP-seq Raw Signal Rep 2 from ENCODE/HAIB
HepG BHLHE40 2

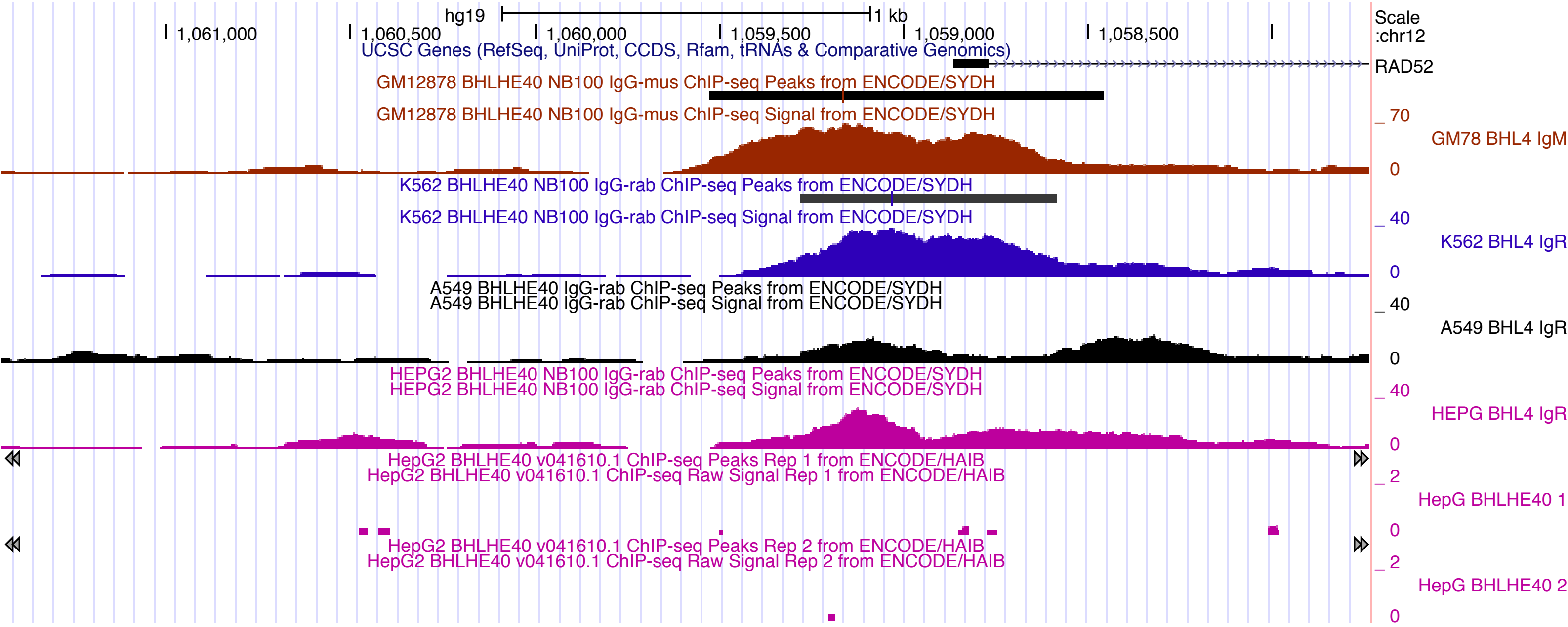

Scale
hg19
1 kb
chr12:
1,061,000
1,060,500
1,060,000
1,059,500
1,059,000
1,058,500
UCSC Genes (RefSeq, UniProt, CCDS, Rfam, tRNAs & Comparative Genomics)
RAD52
GM12878 BHLHE40 NB100 IgG-mus ChIP-seq Peaks from ENCODE/SYDH
GM12878 BHLHE40 NB100 IgG-mus ChIP-seq Signal from ENCODE/SYDH
GM78 BHL4 IgM
70
0
K562 BHLHE40 NB100 IgG-rab ChIP-seq Peaks from ENCODE/SYDH
K562 BHLHE40 NB100 IgG-rab ChIP-seq Signal from ENCODE/SYDH
K562 BHL4 IgR
40
0
A549 BHLHE40 IgG-rab ChIP-seq Peaks from ENCODE/SYDH
A549 BHLHE40 IgG-rab ChIP-seq Signal from ENCODE/SYDH
A549 BHL4 IgR
40
0
HEPG2 BHLHE40 NB100 IgG-rab ChIP-seq Peaks from ENCODE/SYDH
HEPG2 BHLHE40 NB100 IgG-rab ChIP-seq Signal from ENCODE/SYDH
HEPG BHL4 IgR
40
0
HepG2 BHLHE40 v041610.1 ChIP-seq Peaks Rep 1 from ENCODE/HAIB
HepG2 BHLHE40 v041610.1 ChIP-seq Raw Signal Rep 1 from ENCODE/HAIB
HepG BHLHE40 1
2
0
HepG2 BHLHE40 v041610.1 ChIP-seq Peaks Rep 2 from ENCODE/HAIB
HepG2 BHLHE40 v041610.1 ChIP-seq Raw Signal Rep 2 from ENCODE/HAIB
HepG BHLHE40 2
2
0

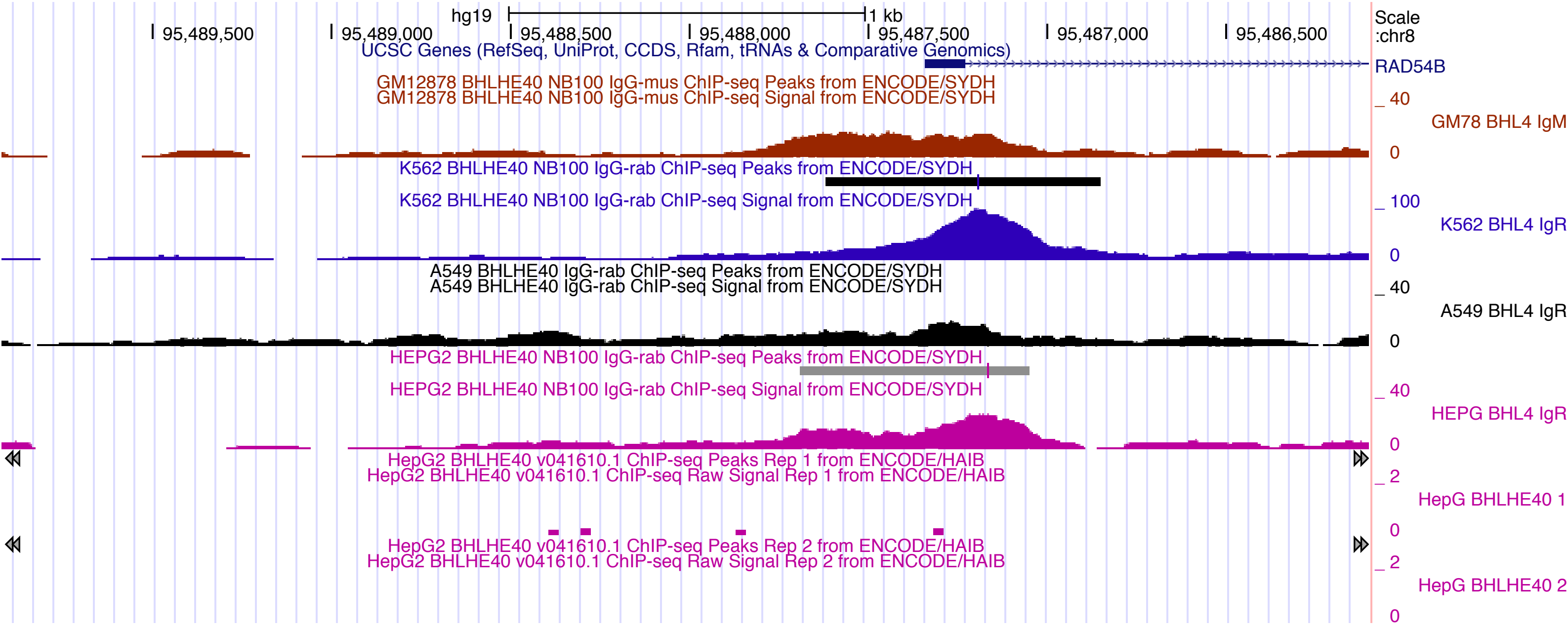

hg19
1 kb
Scale
:chr8
95,489,500
95,489,000
95,488,500
95,488,000
95,487,500
95,487,000
95,486,500
UCSC Genes (RefSeq, UniProt, CCDS, Rfam, tRNAs & Comparative Genomics)
RAD54B
GM12878 BHLHE40 NB100 IgG-mus ChIP-seq Peaks from ENCODE/SYDH
GM12878 BHLHE40 NB100 IgG-mus ChIP-seq Signal from ENCODE/SYDH
_ 40
GM78 BHL4 IgM
0
K562 BHLHE40 NB100 IgG-rab ChIP-seq Peaks from ENCODE/SYDH
K562 BHLHE40 NB100 IgG-rab ChIP-seq Signal from ENCODE/SYDH
_ 100
K562 BHL4 IgR
0
A549 BHLHE40 IgG-rab ChIP-seq Peaks from ENCODE/SYDH
A549 BHLHE40 IgG-rab ChIP-seq Signal from ENCODE/SYDH
_ 40
A549 BHL4 IgR
0
HEPG2 BHLHE40 NB100 IgG-rab ChIP-seq Peaks from ENCODE/SYDH
HEPG2 BHLHE40 NB100 IgG-rab ChIP-seq Signal from ENCODE/SYDH
_ 40
HEPG BHL4 IgR
0
HepG2 BHLHE40 v041610.1 ChIP-seq Peaks Rep 1 from ENCODE/HAIB
HepG2 BHLHE40 v041610.1 ChIP-seq Raw Signal Rep 1 from ENCODE/HAIB
_ 2
HepG BHLHE40 1
0
HepG2 BHLHE40 v041610.1 ChIP-seq Peaks Rep 2 from ENCODE/HAIB
HepG2 BHLHE40 v041610.1 ChIP-seq Raw Signal Rep 2 from ENCODE/HAIB
_ 2
HepG BHLHE40 2
0

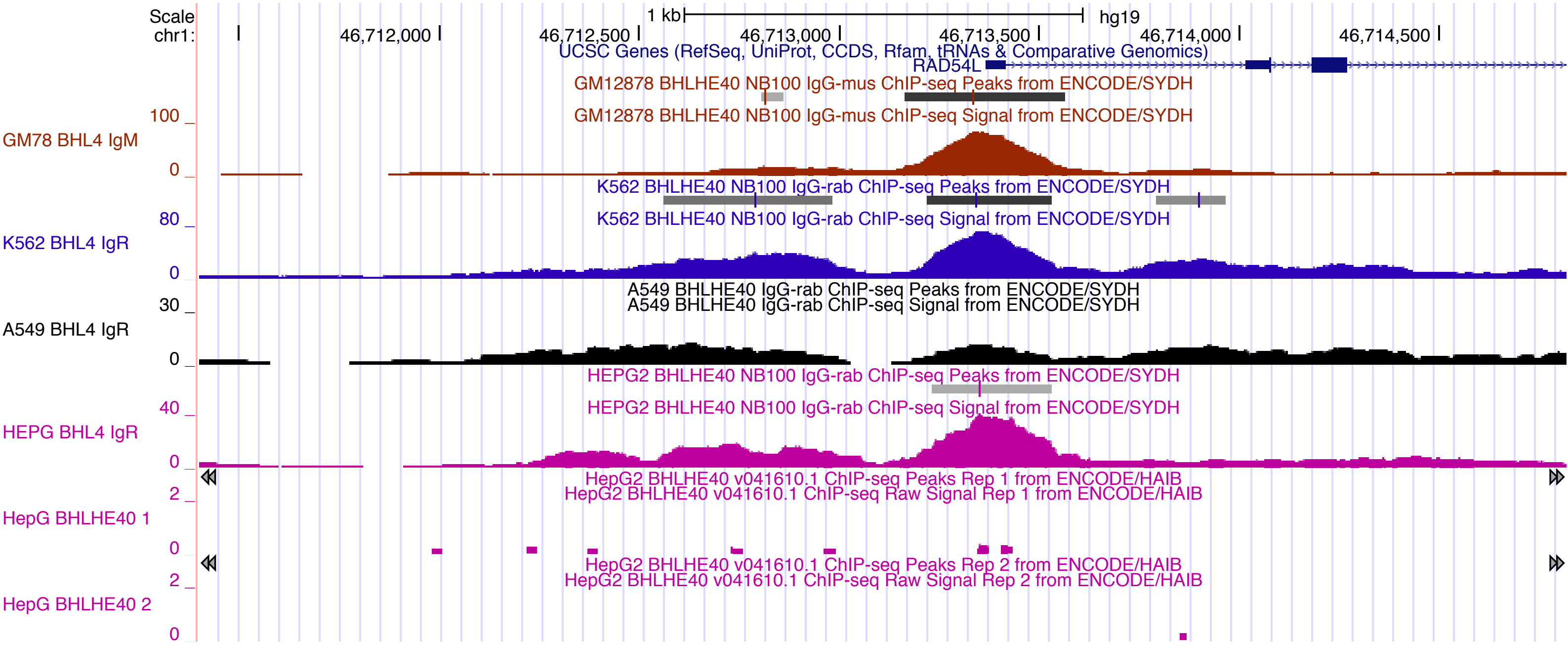

Scale
chr1:
1 kb
hg19
46,712,000
46,712,500
46,713,000
46,713,500
46,714,000
46,714,500
UCSC Genes (RefSeq, UniProt, CCDS, Rfam, tRNAs & Comparative Genomics)
RAD54L
GM12878 BHLHE40 NB100 IgG-mus ChIP-seq Peaks from ENCODE/SYDH
GM12878 BHLHE40 NB100 IgG-mus ChIP-seq Signal from ENCODE/SYDH
GM78 BHL4 IgM
100
0
K562 BHLHE40 NB100 IgG-rab ChIP-seq Peaks from ENCODE/SYDH
K562 BHLHE40 NB100 IgG-rab ChIP-seq Signal from ENCODE/SYDH
K562 BHL4 IgR
80
0
A549 BHLHE40 IgG-rab ChIP-seq Peaks from ENCODE/SYDH
A549 BHLHE40 IgG-rab ChIP-seq Signal from ENCODE/SYDH
A549 BHL4 IgR
30
0
HEPG2 BHLHE40 NB100 IgG-rab ChIP-seq Peaks from ENCODE/SYDH
HEPG2 BHLHE40 NB100 IgG-rab ChIP-seq Signal from ENCODE/SYDH
HEPG BHL4 IgR
40
0
HepG2 BHLHE40 v041610.1 ChIP-seq Peaks Rep 1 from ENCODE/HAIB
HepG2 BHLHE40 v041610.1 ChIP-seq Raw Signal Rep 1 from ENCODE/HAIB
HepG BHLHE40 1
2
0
HepG2 BHLHE40 v041610.1 ChIP-seq Peaks Rep 2 from ENCODE/HAIB
HepG2 BHLHE40 v041610.1 ChIP-seq Raw Signal Rep 2 from ENCODE/HAIB
HepG BHLHE40 2
2
0

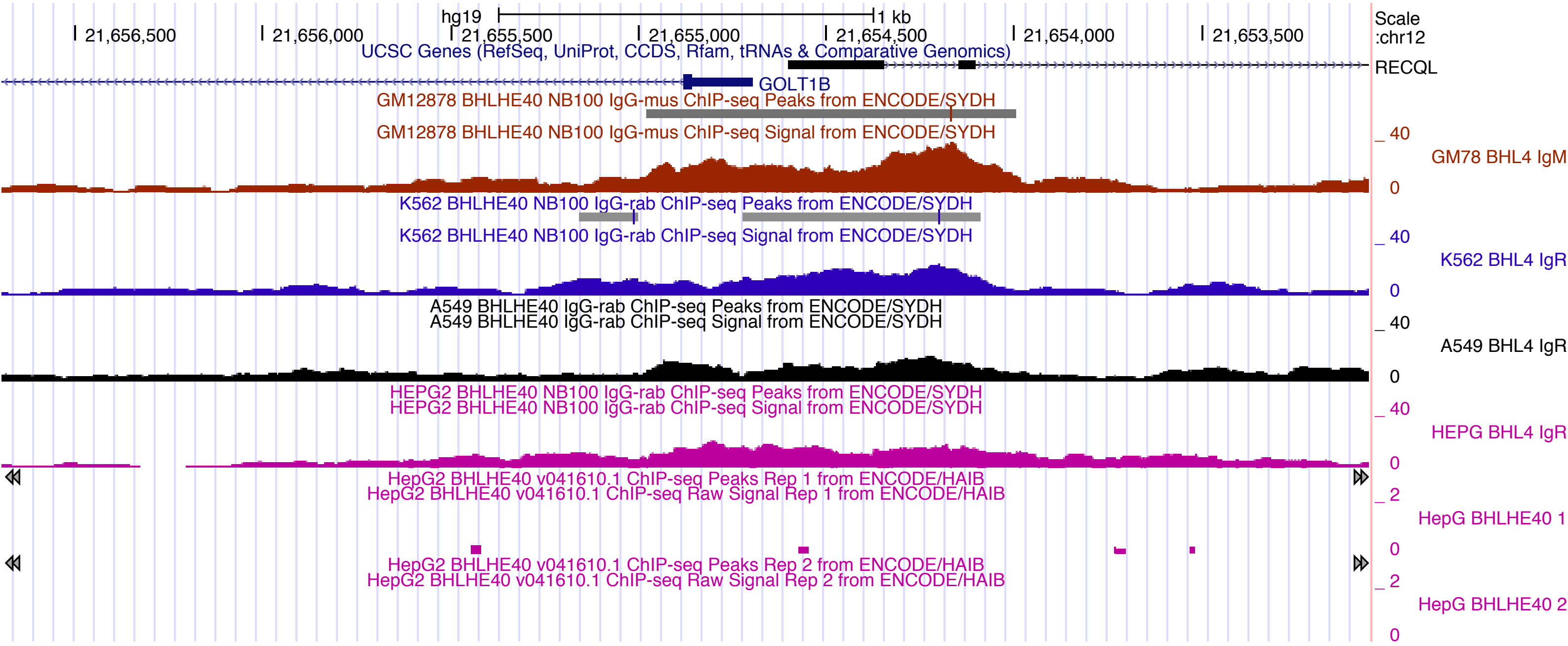

Scale
hg19
1 kb
chr12:
21,656,500
21,656,000
21,655,500
21,655,000
21,654,500
21,654,000
21,653,500
UCSC Genes (RefSeq, UniProt, CCDS, Rfam, tRNAs & Comparative Genomics)
RECQL
GOLT1B
GM12878 BHLHE40 NB100 IgG-mus ChIP-seq Peaks from ENCODE/SYDH
GM12878 BHLHE40 NB100 IgG-mus ChIP-seq Signal from ENCODE/SYDH
GM78 BHL4 IgM
K562 BHLHE40 NB100 IgG-rab ChIP-seq Peaks from ENCODE/SYDH
K562 BHLHE40 NB100 IgG-rab ChIP-seq Signal from ENCODE/SYDH
K562 BHL4 IgR
A549 BHLHE40 IgG-rab ChIP-seq Peaks from ENCODE/SYDH
A549 BHLHE40 IgG-rab ChIP-seq Signal from ENCODE/SYDH
A549 BHL4 IgR
HEPG2 BHLHE40 NB100 IgG-rab ChIP-seq Peaks from ENCODE/SYDH
HEPG2 BHLHE40 NB100 IgG-rab ChIP-seq Signal from ENCODE/SYDH
HEPG BHL4 IgR
HepG2 BHLHE40 v041610.1 ChIP-seq Peaks Rep 1 from ENCODE/HAIB
HepG2 BHLHE40 v041610.1 ChIP-seq Raw Signal Rep 1 from ENCODE/HAIB
HepG BHLHE40 1
HepG2 BHLHE40 v041610.1 ChIP-seq Peaks Rep 2 from ENCODE/HAIB
HepG2 BHLHE40 v041610.1 ChIP-seq Raw Signal Rep 2 from ENCODE/HAIB
HepG BHLHE40 2
40
0
2

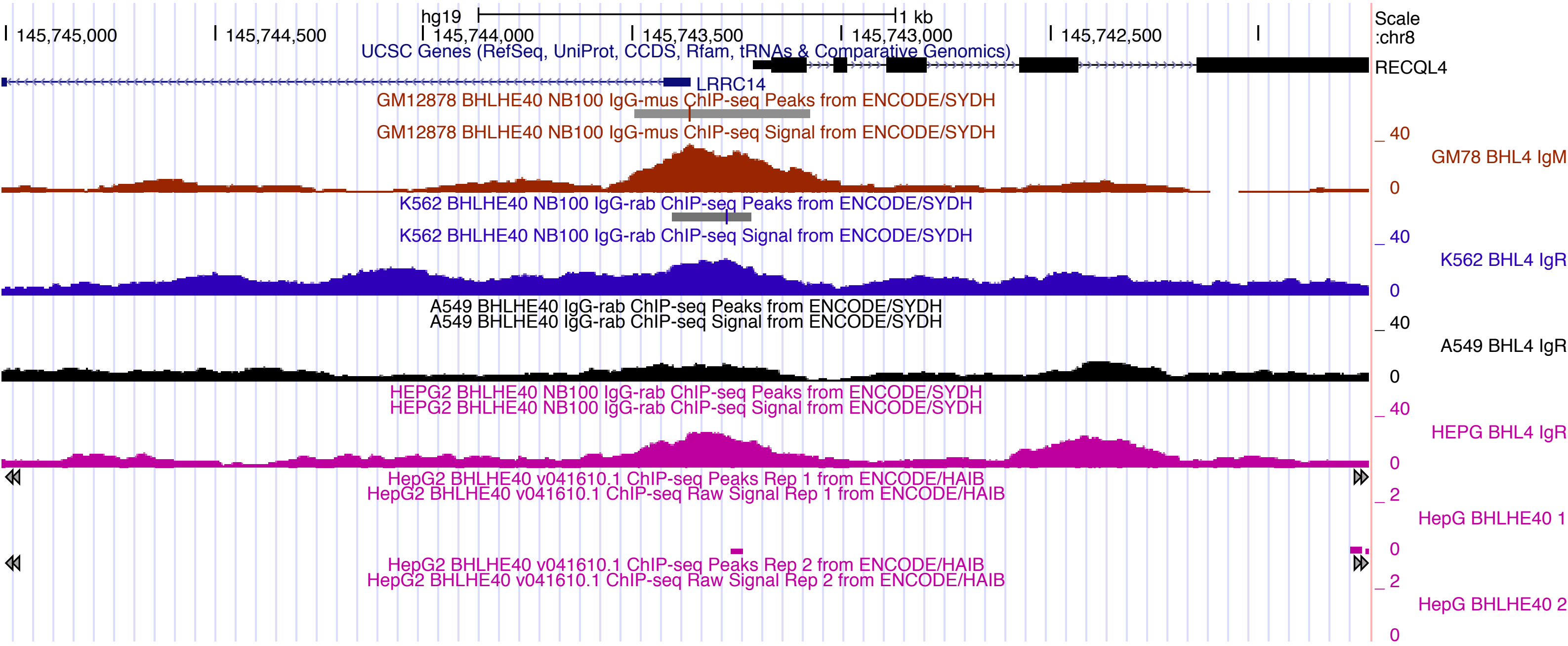

Scale
:chr8
hg19
1 kb
145,745,000
145,744,500
145,744,000
145,743,500
145,743,000
145,742,500
UCSC Genes (RefSeq, UniProt, CCDS, Rfam, tRNAs & Comparative Genomics)
RECQL4
LRRC14
GM12878 BHLHE40 NB100 IgG-mus ChIP-seq Peaks from ENCODE/SYDH
GM12878 BHLHE40 NB100 IgG-mus ChIP-seq Signal from ENCODE/SYDH
GM78 BHL4 IgM
K562 BHLHE40 NB100 IgG-rab ChIP-seq Peaks from ENCODE/SYDH
K562 BHLHE40 NB100 IgG-rab ChIP-seq Signal from ENCODE/SYDH
K562 BHL4 IgR
A549 BHLHE40 IgG-rab ChIP-seq Peaks from ENCODE/SYDH
A549 BHLHE40 IgG-rab ChIP-seq Signal from ENCODE/SYDH
A549 BHL4 IgR
HEPG2 BHLHE40 NB100 IgG-rab ChIP-seq Peaks from ENCODE/SYDH
HEPG2 BHLHE40 NB100 IgG-rab ChIP-seq Signal from ENCODE/SYDH
HEPG BHL4 IgR
HepG2 BHLHE40 v041610.1 ChIP-seq Peaks Rep 1 from ENCODE/HAIB
HepG2 BHLHE40 v041610.1 ChIP-seq Raw Signal Rep 1 from ENCODE/HAIB
HepG BHLHE40 1
HepG2 BHLHE40 v041610.1 ChIP-seq Peaks Rep 2 from ENCODE/HAIB
HepG2 BHLHE40 v041610.1 ChIP-seq Raw Signal Rep 2 from ENCODE/HAIB
HepG BHLHE40 2
_ 40
0
_ 40
0
_ 40
0
_ 40
0
_ 2
0
_ 2
0

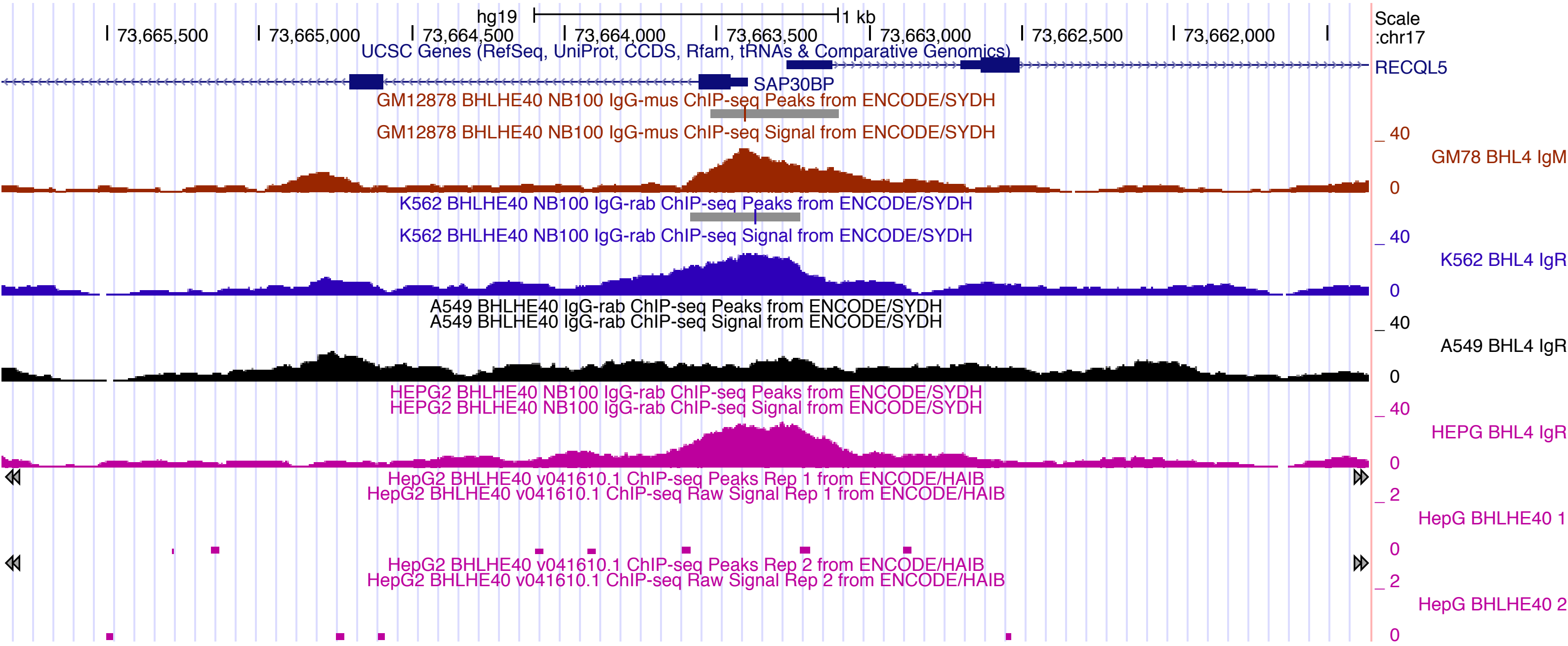
hg19
1 kb
Scale
:chr17
73,665,500
73,665,000
73,664,500
73,664,000
73,663,500
73,663,000
73,662,500
73,662,000
UCSC Genes (RefSeq, UniProt, CCDS, Rfam, tRNAs & Comparative Genomics)
RECQL5
SAP30BP
GM12878 BHLHE40 NB100 IgG-mus ChIP-seq Peaks from ENCODE/SYDH
GM12878 BHLHE40 NB100 IgG-mus ChIP-seq Signal from ENCODE/SYDH
GM78 BHL4 IgM
K562 BHLHE40 NB100 IgG-rab ChIP-seq Peaks from ENCODE/SYDH
K562 BHLHE40 NB100 IgG-rab ChIP-seq Signal from ENCODE/SYDH
K562 BHL4 IgR
A549 BHLHE40 IgG-rab ChIP-seq Peaks from ENCODE/SYDH
A549 BHLHE40 IgG-rab ChIP-seq Signal from ENCODE/SYDH
A549 BHL4 IgR
HEPG2 BHLHE40 NB100 IgG-rab ChIP-seq Peaks from ENCODE/SYDH
HEPG2 BHLHE40 NB100 IgG-rab ChIP-seq Signal from ENCODE/SYDH
HEPG BHL4 IgR
HepG2 BHLHE40 v041610.1 ChIP-seq Peaks Rep 1 from ENCODE/HAIB
HepG2 BHLHE40 v041610.1 ChIP-seq Raw Signal Rep 1 from ENCODE/HAIB
HepG BHLHE40 1
HepG2 BHLHE40 v041610.1 ChIP-seq Peaks Rep 2 from ENCODE/HAIB
HepG2 BHLHE40 v041610.1 ChIP-seq Raw Signal Rep 2 from ENCODE/HAIB
HepG BHLHE40 2
_ 40
0
_ 2

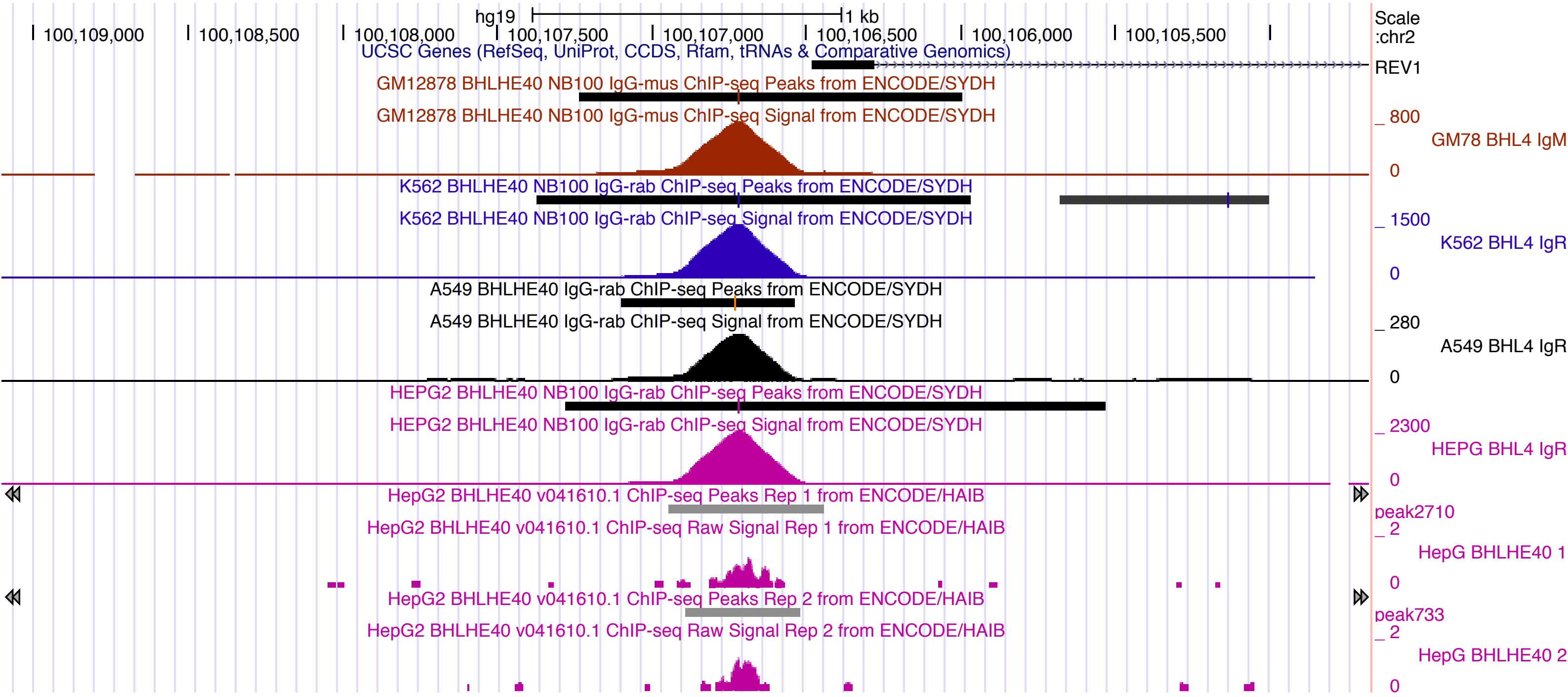

hg19
1 kb
Scale
:chr2
100,109,000
100,108,500
100,108,000
100,107,500
100,107,000
100,106,500
100,106,000
100,105,500
UCSC Genes (RefSeq, UniProt, CCDS, Rfam, tRNAs & Comparative Genomics)
REV1
GM12878 BHLHE40 NB100 IgG-mus ChIP-seq Peaks from ENCODE/SYDH
GM12878 BHLHE40 NB100 IgG-mus ChIP-seq Signal from ENCODE/SYDH
_ 800
GM78 BHL4 IgM
0
K562 BHLHE40 NB100 IgG-rab ChIP-seq Peaks from ENCODE/SYDH
K562 BHLHE40 NB100 IgG-rab ChIP-seq Signal from ENCODE/SYDH
_ 1500
K562 BHL4 IgR
0
A549 BHLHE40 IgG-rab ChIP-seq Peaks from ENCODE/SYDH
A549 BHLHE40 IgG-rab ChIP-seq Signal from ENCODE/SYDH
_ 280
A549 BHL4 IgR
0
HEPG2 BHLHE40 NB100 IgG-rab ChIP-seq Peaks from ENCODE/SYDH
HEPG2 BHLHE40 NB100 IgG-rab ChIP-seq Signal from ENCODE/SYDH
_ 2300
HEPG BHL4 IgR
0
HepG2 BHLHE40 v041610.1 ChIP-seq Peaks Rep 1 from ENCODE/HAIB
peak2710
HepG2 BHLHE40 v041610.1 ChIP-seq Raw Signal Rep 1 from ENCODE/HAIB
_ 2
HepG BHLHE40 1
0
HepG2 BHLHE40 v041610.1 ChIP-seq Peaks Rep 2 from ENCODE/HAIB
peak733
HepG2 BHLHE40 v041610.1 ChIP-seq Raw Signal Rep 2 from ENCODE/HAIB
_ 2
HepG BHLHE40 2
0

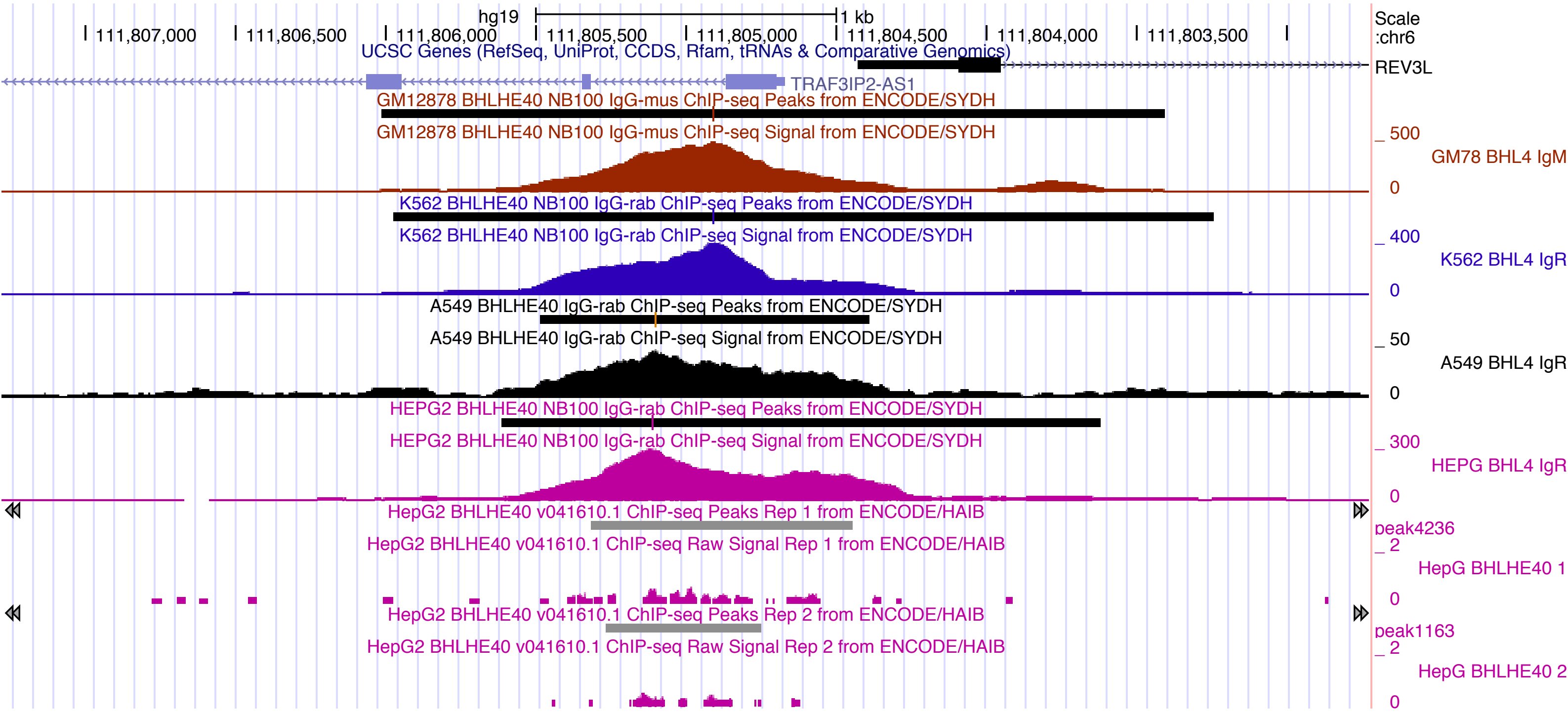
hg19
1 kb
111,807,000
111,806,500
111,806,000
111,805,500
111,805,000
111,804,500
111,804,000
111,803,500
Scale
:chr6
UCSC Genes (RefSeq, UniProt, CCDS, Rfam, tRNAs & Comparative Genomics)
REV3L
TRAF3IP2-AS1
GM12878 BHLHE40 NB100 IgG-mus ChIP-seq Peaks from ENCODE/SYDH
GM12878 BHLHE40 NB100 IgG-mus ChIP-seq Signal from ENCODE/SYDH
_ 500
GM78 BHL4 IgM
0
K562 BHLHE40 NB100 IgG-rab ChIP-seq Peaks from ENCODE/SYDH
K562 BHLHE40 NB100 IgG-rab ChIP-seq Signal from ENCODE/SYDH
_ 400
K562 BHL4 IgR
0
A549 BHLHE40 IgG-rab ChIP-seq Peaks from ENCODE/SYDH
A549 BHLHE40 IgG-rab ChIP-seq Signal from ENCODE/SYDH
_ 50
A549 BHL4 IgR
0
HEPG2 BHLHE40 NB100 IgG-rab ChIP-seq Peaks from ENCODE/SYDH
HEPG2 BHLHE40 NB100 IgG-rab ChIP-seq Signal from ENCODE/SYDH
_ 300
HEPG BHL4 IgR
0
HepG2 BHLHE40 v041610.1 ChIP-seq Peaks Rep 1 from ENCODE/HAIB
peak4236
HepG2 BHLHE40 v041610.1 ChIP-seq Raw Signal Rep 1 from ENCODE/HAIB
_ 2
HepG BHLHE40 1
0
HepG2 BHLHE40 v041610.1 ChIP-seq Peaks Rep 2 from ENCODE/HAIB
peak1163
HepG2 BHLHE40 v041610.1 ChIP-seq Raw Signal Rep 2 from ENCODE/HAIB
_ 2
HepG BHLHE40 2
0

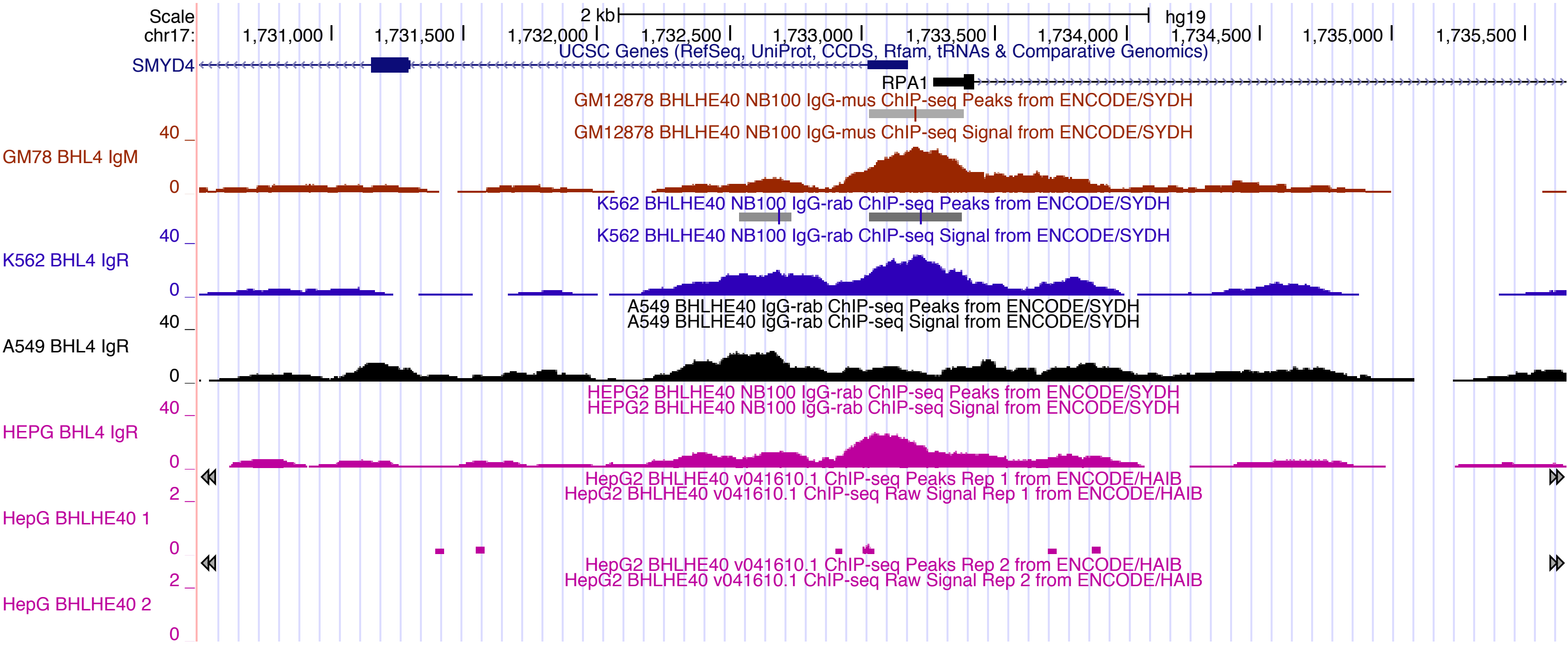

Scale
chr17:
2 kb
hg19
1,731,000
1,731,500
1,732,000
1,732,500
1,733,000
1,733,500
1,734,000
1,734,500
1,735,000
1,735,500
UCSC Genes (RefSeq, UniProt, CCDS, Rfam, tRNAs & Comparative Genomics)
SMYD4
RPA1
GM12878 BHLHE40 NB100 IgG-mus ChIP-seq Peaks from ENCODE/SYDH
GM12878 BHLHE40 NB100 IgG-mus ChIP-seq Signal from ENCODE/SYDH
GM78 BHL4 IgM
K562 BHLHE40 NB100 IgG-rab ChIP-seq Peaks from ENCODE/SYDH
K562 BHLHE40 NB100 IgG-rab ChIP-seq Signal from ENCODE/SYDH
K562 BHL4 IgR
A549 BHLHE40 IgG-rab ChIP-seq Peaks from ENCODE/SYDH
A549 BHLHE40 IgG-rab ChIP-seq Signal from ENCODE/SYDH
A549 BHL4 IgR
HEPG2 BHLHE40 NB100 IgG-rab ChIP-seq Peaks from ENCODE/SYDH
HEPG2 BHLHE40 NB100 IgG-rab ChIP-seq Signal from ENCODE/SYDH
HEPG BHL4 IgR
HepG2 BHLHE40 v041610.1 ChIP-seq Peaks Rep 1 from ENCODE/HAIB
HepG2 BHLHE40 v041610.1 ChIP-seq Raw Signal Rep 1 from ENCODE/HAIB
HepG BHLHE40 1
HepG2 BHLHE40 v041610.1 ChIP-seq Peaks Rep 2 from ENCODE/HAIB
HepG2 BHLHE40 v041610.1 ChIP-seq Raw Signal Rep 2 from ENCODE/HAIB
HepG BHLHE40 2

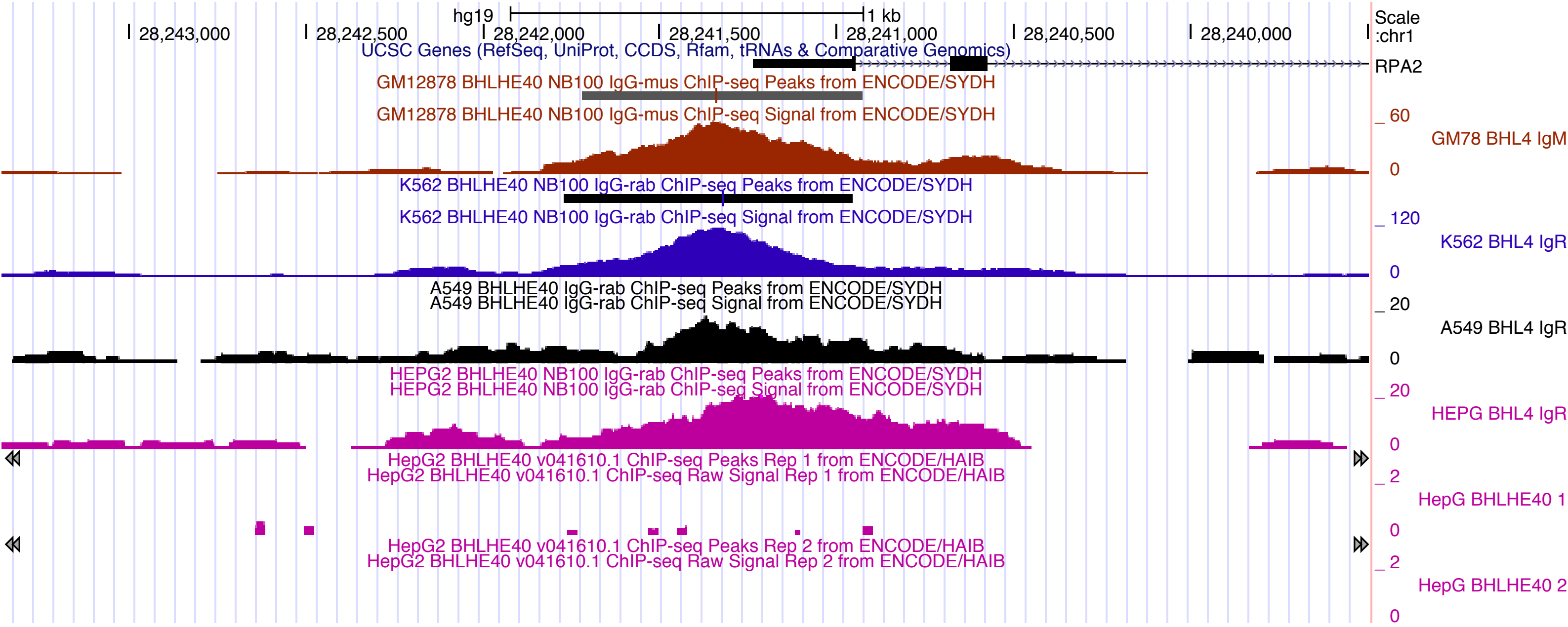
hg19
1 kb
Scale
chr1
28,243,000
28,242,500
28,242,000
28,241,500
28,241,000
28,240,500
28,240,000
UCSC Genes (RefSeq, UniProt, CCDS, Rfam, tRNAs & Comparative Genomics)
RPA2
GM12878 BHLHE40 NB100 IgG-mus ChIP-seq Peaks from ENCODE/SYDH
GM12878 BHLHE40 NB100 IgG-mus ChIP-seq Signal from ENCODE/SYDH
GM78 BHL4 IgM
K562 BHLHE40 NB100 IgG-rab ChIP-seq Peaks from ENCODE/SYDH
K562 BHLHE40 NB100 IgG-rab ChIP-seq Signal from ENCODE/SYDH
K562 BHL4 IgR
A549 BHLHE40 IgG-rab ChIP-seq Peaks from ENCODE/SYDH
A549 BHLHE40 IgG-rab ChIP-seq Signal from ENCODE/SYDH
A549 BHL4 IgR
HEPG2 BHLHE40 NB100 IgG-rab ChIP-seq Peaks from ENCODE/SYDH
HEPG2 BHLHE40 NB100 IgG-rab ChIP-seq Signal from ENCODE/SYDH
HEPG BHL4 IgR
HepG2 BHLHE40 v041610.1 ChIP-seq Peaks Rep 1 from ENCODE/HAIB
HepG2 BHLHE40 v041610.1 ChIP-seq Raw Signal Rep 1 from ENCODE/HAIB
HepG BHLHE40 1
HepG2 BHLHE40 v041610.1 ChIP-seq Peaks Rep 2 from ENCODE/HAIB
HepG2 BHLHE40 v041610.1 ChIP-seq Raw Signal Rep 2 from ENCODE/HAIB
HepG BHLHE40 2
60
0
120
0
20
0
20
0
2
0
2
0

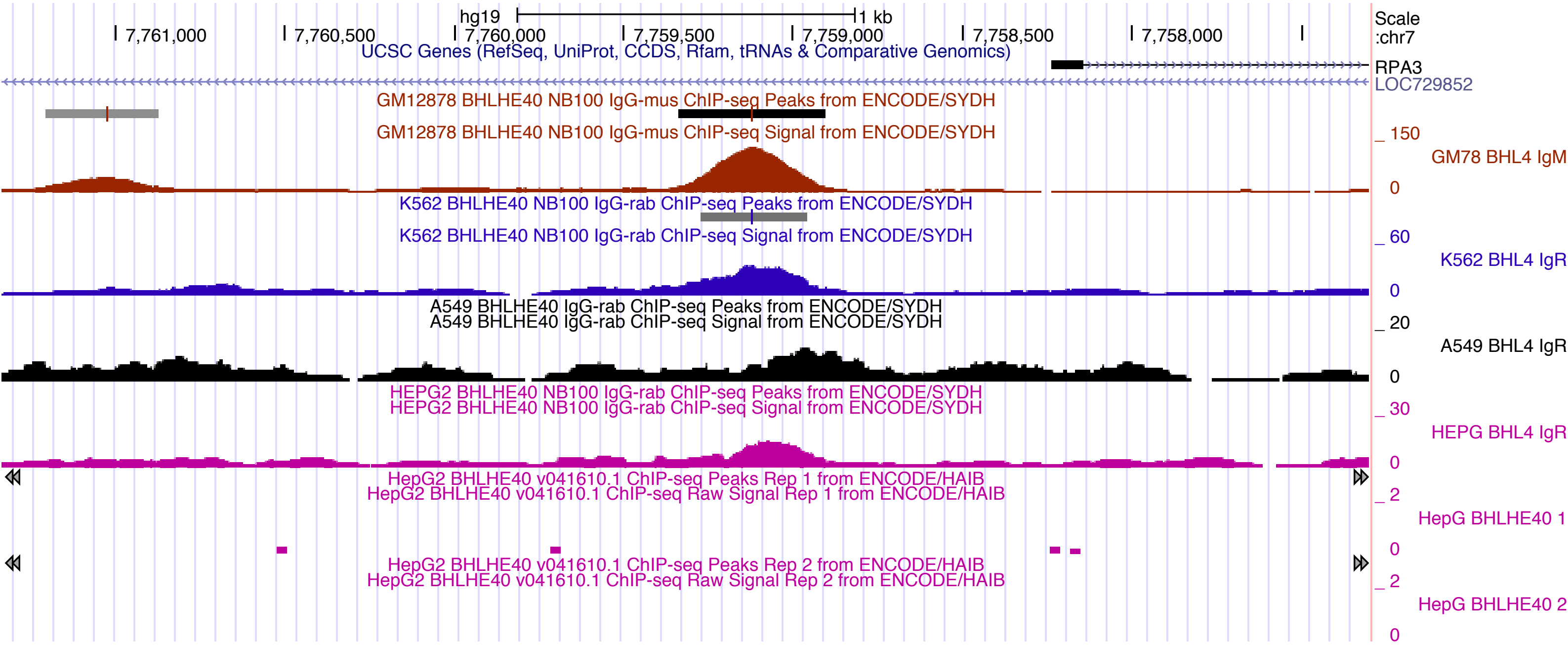

hg19
1 kb
Scale
:chr7
7,761,000
7,760,500
7,760,000
7,759,500
7,759,000
7,758,500
7,758,000
UCSC Genes (RefSeq, UniProt, CCDS, Rfam, tRNAs & Comparative Genomics)
RPA3
LOC729852
GM12878 BHLHE40 NB100 IgG-mus ChIP-seq Peaks from ENCODE/SYDH
GM12878 BHLHE40 NB100 IgG-mus ChIP-seq Signal from ENCODE/SYDH
_ 150
GM78 BHL4 IgM
0
K562 BHLHE40 NB100 IgG-rab ChIP-seq Peaks from ENCODE/SYDH
K562 BHLHE40 NB100 IgG-rab ChIP-seq Signal from ENCODE/SYDH
_ 60
K562 BHL4 IgR
0
A549 BHLHE40 IgG-rab ChIP-seq Peaks from ENCODE/SYDH
A549 BHLHE40 IgG-rab ChIP-seq Signal from ENCODE/SYDH
_ 20
A549 BHL4 IgR
0
HEPG2 BHLHE40 NB100 IgG-rab ChIP-seq Peaks from ENCODE/SYDH
HEPG2 BHLHE40 NB100 IgG-rab ChIP-seq Signal from ENCODE/SYDH
_ 30
HEPG BHL4 IgR
0
HepG2 BHLHE40 v041610.1 ChIP-seq Peaks Rep 1 from ENCODE/HAIB
HepG2 BHLHE40 v041610.1 ChIP-seq Raw Signal Rep 1 from ENCODE/HAIB
_ 2
HepG BHLHE40 1
0
HepG2 BHLHE40 v041610.1 ChIP-seq Peaks Rep 2 from ENCODE/HAIB
HepG2 BHLHE40 v041610.1 ChIP-seq Raw Signal Rep 2 from ENCODE/HAIB
_ 2
HepG BHLHE40 2
0

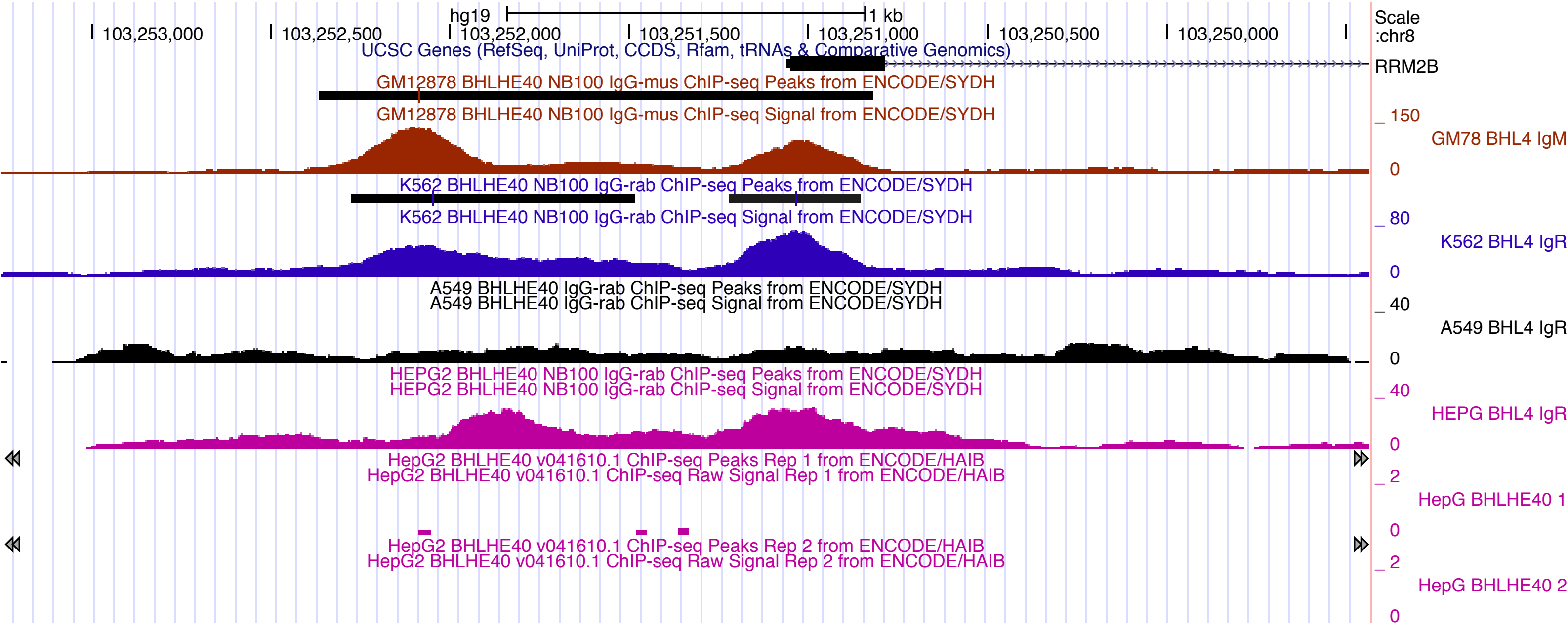

hg19
1 kb
Scale
:chr8
103,253,000
103,252,500
103,252,000
103,251,500
103,251,000
103,250,500
103,250,000
UCSC Genes (RefSeq, UniProt, CCDS, Rfam, tRNAs & Comparative Genomics)
RRM2B
GM12878 BHLHE40 NB100 IgG-mus ChIP-seq Peaks from ENCODE/SYDH
GM12878 BHLHE40 NB100 IgG-mus ChIP-seq Signal from ENCODE/SYDH
150
GM78 BHL4 IgM
0
K562 BHLHE40 NB100 IgG-rab ChIP-seq Peaks from ENCODE/SYDH
K562 BHLHE40 NB100 IgG-rab ChIP-seq Signal from ENCODE/SYDH
80
K562 BHL4 IgR
0
A549 BHLHE40 IgG-rab ChIP-seq Peaks from ENCODE/SYDH
A549 BHLHE40 IgG-rab ChIP-seq Signal from ENCODE/SYDH
40
A549 BHL4 IgR
0
HEPG2 BHLHE40 NB100 IgG-rab ChIP-seq Peaks from ENCODE/SYDH
HEPG2 BHLHE40 NB100 IgG-rab ChIP-seq Signal from ENCODE/SYDH
40
HEPG BHL4 IgR
0
HepG2 BHLHE40 v041610.1 ChIP-seq Peaks Rep 1 from ENCODE/HAIB
HepG2 BHLHE40 v041610.1 ChIP-seq Raw Signal Rep 1 from ENCODE/HAIB
2
HepG BHLHE40 1
0
HepG2 BHLHE40 v041610.1 ChIP-seq Peaks Rep 2 from ENCODE/HAIB
HepG2 BHLHE40 v041610.1 ChIP-seq Raw Signal Rep 2 from ENCODE/HAIB
2
HepG BHLHE40 2
0

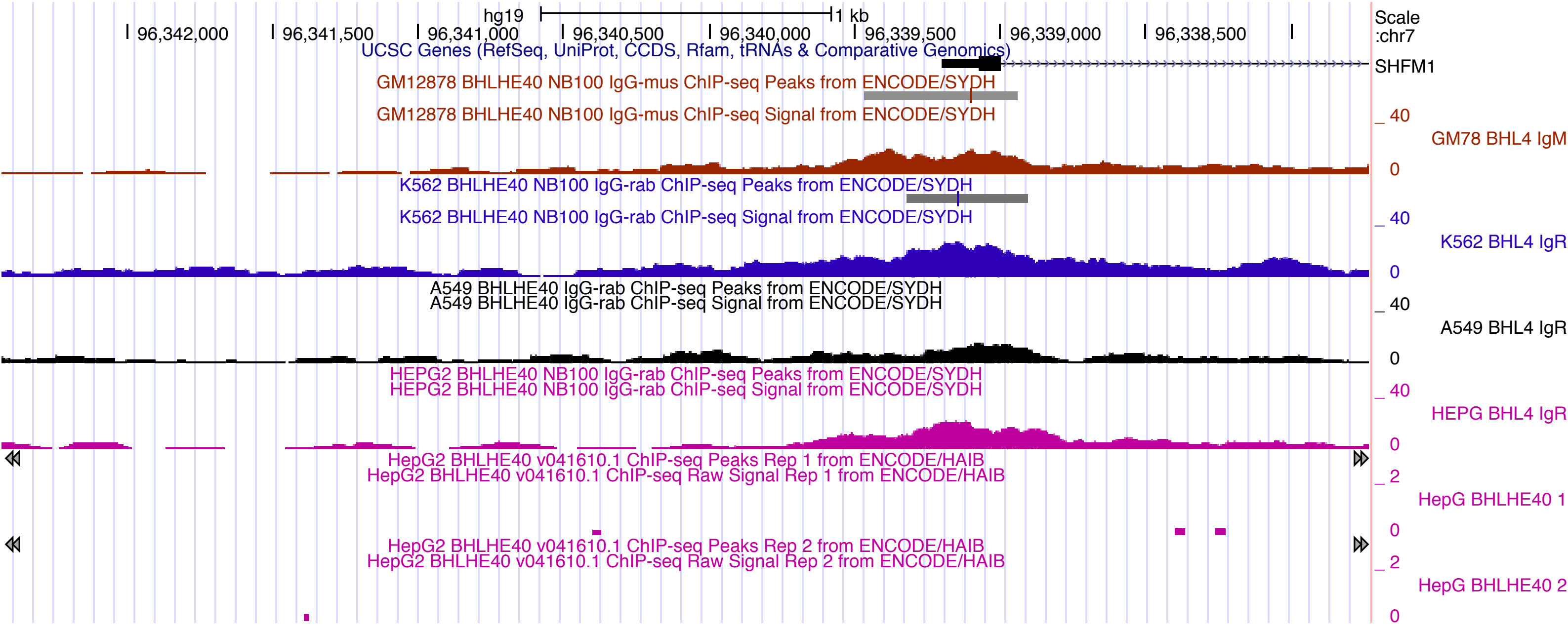
hg19
1 kb
Scale
:chr7
96,342,000
96,341,500
96,341,000
96,340,500
96,340,000
96,339,500
96,339,000
96,338,500
UCSC Genes (RefSeq, UniProt, CCDS, Rfam, tRNAs & Comparative Genomics)
SHFM1
GM12878 BHLHE40 NB100 IgG-mus ChIP-seq Peaks from ENCODE/SYDH
GM12878 BHLHE40 NB100 IgG-mus ChIP-seq Signal from ENCODE/SYDH
GM78 BHL4 IgM
K562 BHLHE40 NB100 IgG-rab ChIP-seq Peaks from ENCODE/SYDH
K562 BHLHE40 NB100 IgG-rab ChIP-seq Signal from ENCODE/SYDH
K562 BHL4 IgR
A549 BHLHE40 IgG-rab ChIP-seq Peaks from ENCODE/SYDH
A549 BHLHE40 IgG-rab ChIP-seq Signal from ENCODE/SYDH
A549 BHL4 IgR
HEPG2 BHLHE40 NB100 IgG-rab ChIP-seq Peaks from ENCODE/SYDH
HEPG2 BHLHE40 NB100 IgG-rab ChIP-seq Signal from ENCODE/SYDH
HEPG BHL4 IgR
HepG2 BHLHE40 v041610.1 ChIP-seq Peaks Rep 1 from ENCODE/HAIB
HepG2 BHLHE40 v041610.1 ChIP-seq Raw Signal Rep 1 from ENCODE/HAIB
HepG BHLHE40 1
HepG2 BHLHE40 v041610.1 ChIP-seq Peaks Rep 2 from ENCODE/HAIB
HepG2 BHLHE40 v041610.1 ChIP-seq Raw Signal Rep 2 from ENCODE/HAIB
HepG BHLHE40 2

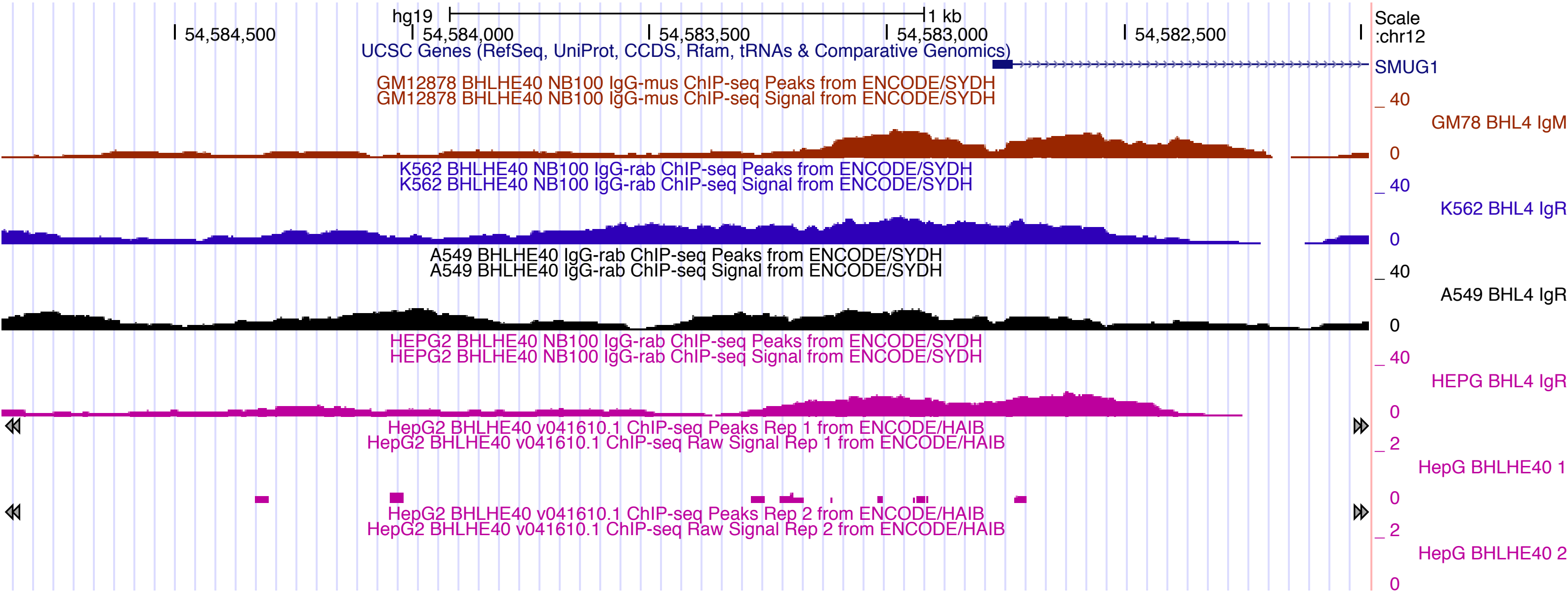

hg19
1 kb
Scale
:chr12
54,584,500
54,584,000
54,583,500
54,583,000
54,582,500
UCSC Genes (RefSeq, UniProt, CCDS, Rfam, tRNAs & Comparative Genomics)
SMUG1
GM12878 BHLHE40 NB100 IgG-mus ChIP-seq Peaks from ENCODE/SYDH
GM12878 BHLHE40 NB100 IgG-mus ChIP-seq Signal from ENCODE/SYDH
GM78 BHL4 IgM
K562 BHLHE40 NB100 IgG-rab ChIP-seq Peaks from ENCODE/SYDH
K562 BHLHE40 NB100 IgG-rab ChIP-seq Signal from ENCODE/SYDH
K562 BHL4 IgR
A549 BHLHE40 IgG-rab ChIP-seq Peaks from ENCODE/SYDH
A549 BHLHE40 IgG-rab ChIP-seq Signal from ENCODE/SYDH
A549 BHL4 IgR
HEPG2 BHLHE40 NB100 IgG-rab ChIP-seq Peaks from ENCODE/SYDH
HEPG2 BHLHE40 NB100 IgG-rab ChIP-seq Signal from ENCODE/SYDH
HEPG BHL4 IgR
HepG2 BHLHE40 v041610.1 ChIP-seq Peaks Rep 1 from ENCODE/HAIB
HepG2 BHLHE40 v041610.1 ChIP-seq Raw Signal Rep 1 from ENCODE/HAIB
HepG BHLHE40 1
HepG2 BHLHE40 v041610.1 ChIP-seq Peaks Rep 2 from ENCODE/HAIB
HepG2 BHLHE40 v041610.1 ChIP-seq Raw Signal Rep 2 from ENCODE/HAIB
HepG BHLHE40 2
40
0
2

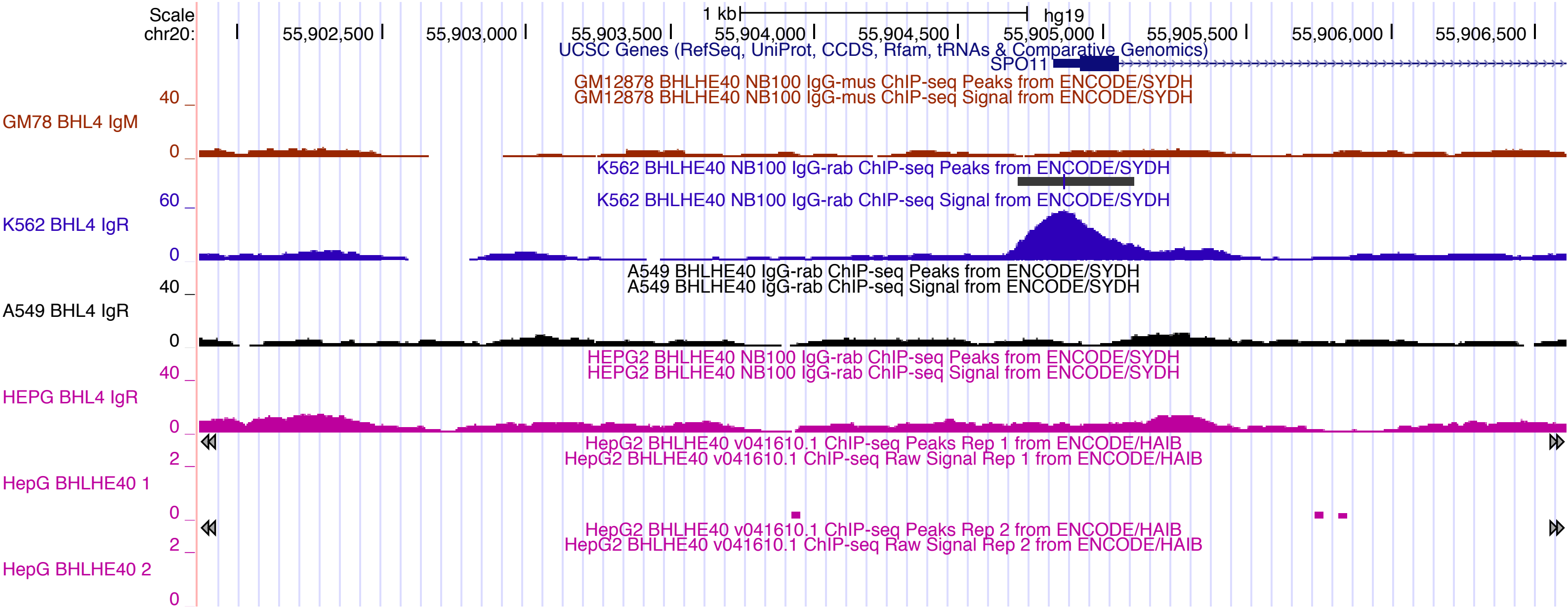
Scale
chr20:
1 kb
hg19
55,902,500
55,903,000
55,903,500
55,904,000
55,904,500
55,905,000
55,905,500
55,906,000
55,906,500
UCSC Genes (RefSeq, UniProt, CCDS, Rfam, tRNAs & Comparative Genomics)
SPO11
GM12878 BHLHE40 NB100 IgG-mus ChIP-seq Peaks from ENCODE/SYDH
GM12878 BHLHE40 NB100 IgG-mus ChIP-seq Signal from ENCODE/SYDH
40 _
GM78 BHL4 IgM
0 _
K562 BHLHE40 NB100 IgG-rab ChIP-seq Peaks from ENCODE/SYDH
K562 BHLHE40 NB100 IgG-rab ChIP-seq Signal from ENCODE/SYDH
60 _
K562 BHL4 IgR
0
A549 BHLHE40 IgG-rab ChIP-seq Peaks from ENCODE/SYDH
A549 BHLHE40 IgG-rab ChIP-seq Signal from ENCODE/SYDH
40 _
A549 BHL4 IgR
0
HEPG2 BHLHE40 NB100 IgG-rab ChIP-seq Peaks from ENCODE/SYDH
HEPG2 BHLHE40 NB100 IgG-rab ChIP-seq Signal from ENCODE/SYDH
40 _
HEPG BHL4 IgR
0
HepG2 BHLHE40 v041610.1 ChIP-seq Peaks Rep 1 from ENCODE/HAIB
HepG2 BHLHE40 v041610.1 ChIP-seq Raw Signal Rep 1 from ENCODE/HAIB
2 _
HepG BHLHE40 1
0 _
HepG2 BHLHE40 v041610.1 ChIP-seq Peaks Rep 2 from ENCODE/HAIB
HepG2 BHLHE40 v041610.1 ChIP-seq Raw Signal Rep 2 from ENCODE/HAIB
2 _
HepG BHLHE40 2
0

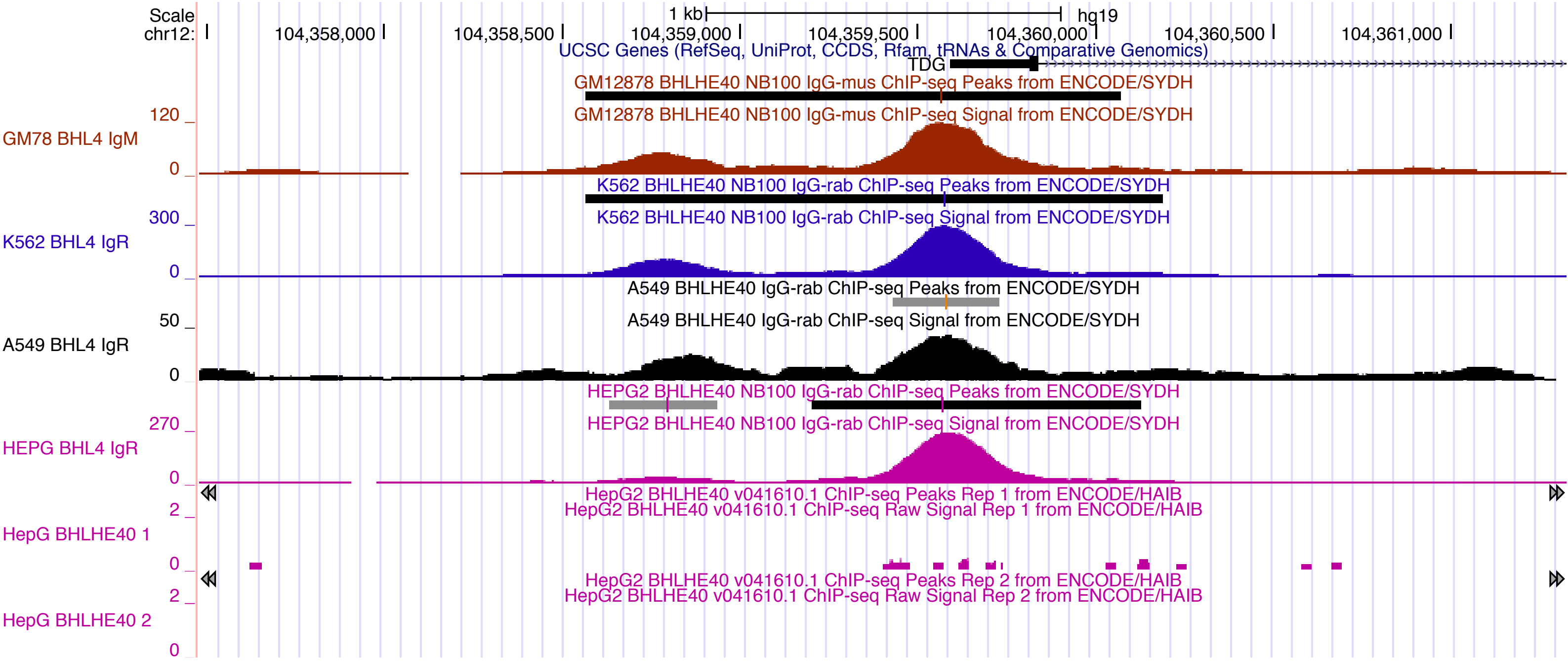

Scale
chr12:
1 kb
hg19
104,358,000
104,358,500
104,359,000
104,359,500
104,360,000
104,360,500
104,361,000
UCSC Genes (RefSeq, UniProt, CCDS, Rfam, tRNAs & Comparative Genomics)
TDG
GM12878 BHLHE40 NB100 IgG-mus ChIP-seq Peaks from ENCODE/SYDH
GM12878 BHLHE40 NB100 IgG-mus ChIP-seq Signal from ENCODE/SYDH
GM78 BHL4 IgM
120
0
K562 BHLHE40 NB100 IgG-rab ChIP-seq Peaks from ENCODE/SYDH
K562 BHLHE40 NB100 IgG-rab ChIP-seq Signal from ENCODE/SYDH
K562 BHL4 IgR
300
0
A549 BHLHE40 IgG-rab ChIP-seq Peaks from ENCODE/SYDH
A549 BHLHE40 IgG-rab ChIP-seq Signal from ENCODE/SYDH
A549 BHL4 IgR
50
0
HEPG2 BHLHE40 NB100 IgG-rab ChIP-seq Peaks from ENCODE/SYDH
HEPG2 BHLHE40 NB100 IgG-rab ChIP-seq Signal from ENCODE/SYDH
HEPG BHL4 IgR
270
0
HepG2 BHLHE40 v041610.1 ChIP-seq Peaks Rep 1 from ENCODE/HAIB
HepG2 BHLHE40 v041610.1 ChIP-seq Raw Signal Rep 1 from ENCODE/HAIB
HepG BHLHE40 1
2
0
HepG2 BHLHE40 v041610.1 ChIP-seq Peaks Rep 2 from ENCODE/HAIB
HepG2 BHLHE40 v041610.1 ChIP-seq Raw Signal Rep 2 from ENCODE/HAIB
HepG BHLHE40 2
2
0

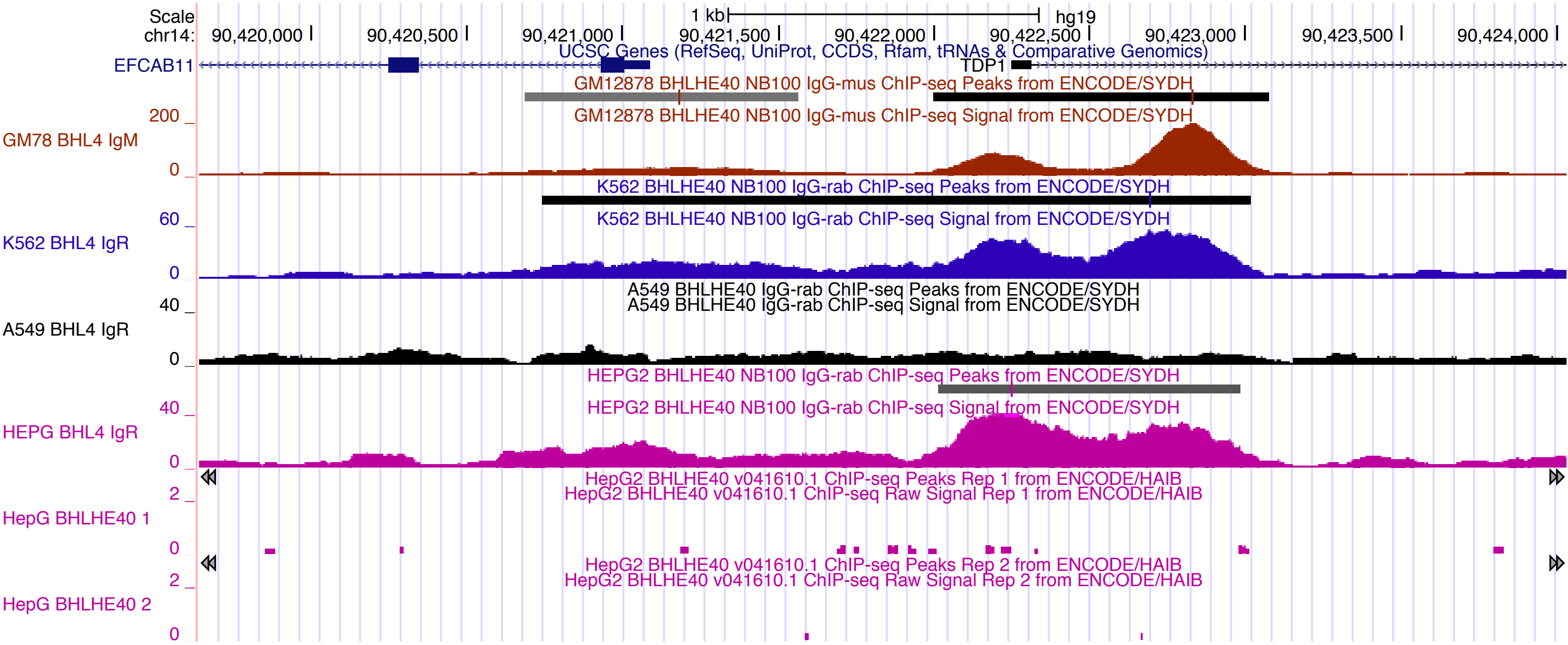

Scale
chr14:
1 kb
hg19
90,420,000
90,420,500
90,421,000
90,421,500
90,422,000
90,422,500
90,423,000
90,423,500
90,424,000
UCSC Genes (RefSeq, UniProt, CCDS, Rfam, tRNAs & Comparative Genomics)
EFCAB11
TDP1
GM12878 BHLHE40 NB100 IgG-mus ChIP-seq Peaks from ENCODE/SYDH
GM12878 BHLHE40 NB100 IgG-mus ChIP-seq Signal from ENCODE/SYDH
200
GM78 BHL4 IgM
0
K562 BHLHE40 NB100 IgG-rab ChIP-seq Peaks from ENCODE/SYDH
K562 BHLHE40 NB100 IgG-rab ChIP-seq Signal from ENCODE/SYDH
60
K562 BHL4 IgR
0
A549 BHLHE40 IgG-rab ChIP-seq Peaks from ENCODE/SYDH
A549 BHLHE40 IgG-rab ChIP-seq Signal from ENCODE/SYDH
40
A549 BHL4 IgR
0
HEPG2 BHLHE40 NB100 IgG-rab ChIP-seq Peaks from ENCODE/SYDH
HEPG2 BHLHE40 NB100 IgG-rab ChIP-seq Signal from ENCODE/SYDH
40
HEPG BHL4 IgR
0
HepG2 BHLHE40 v041610.1 ChIP-seq Peaks Rep 1 from ENCODE/HAIB
HepG2 BHLHE40 v041610.1 ChIP-seq Raw Signal Rep 1 from ENCODE/HAIB
2
HepG BHLHE40 1
0
HepG2 BHLHE40 v041610.1 ChIP-seq Peaks Rep 2 from ENCODE/HAIB
HepG2 BHLHE40 v041610.1 ChIP-seq Raw Signal Rep 2 from ENCODE/HAIB
2
HepG BHLHE40 2
0

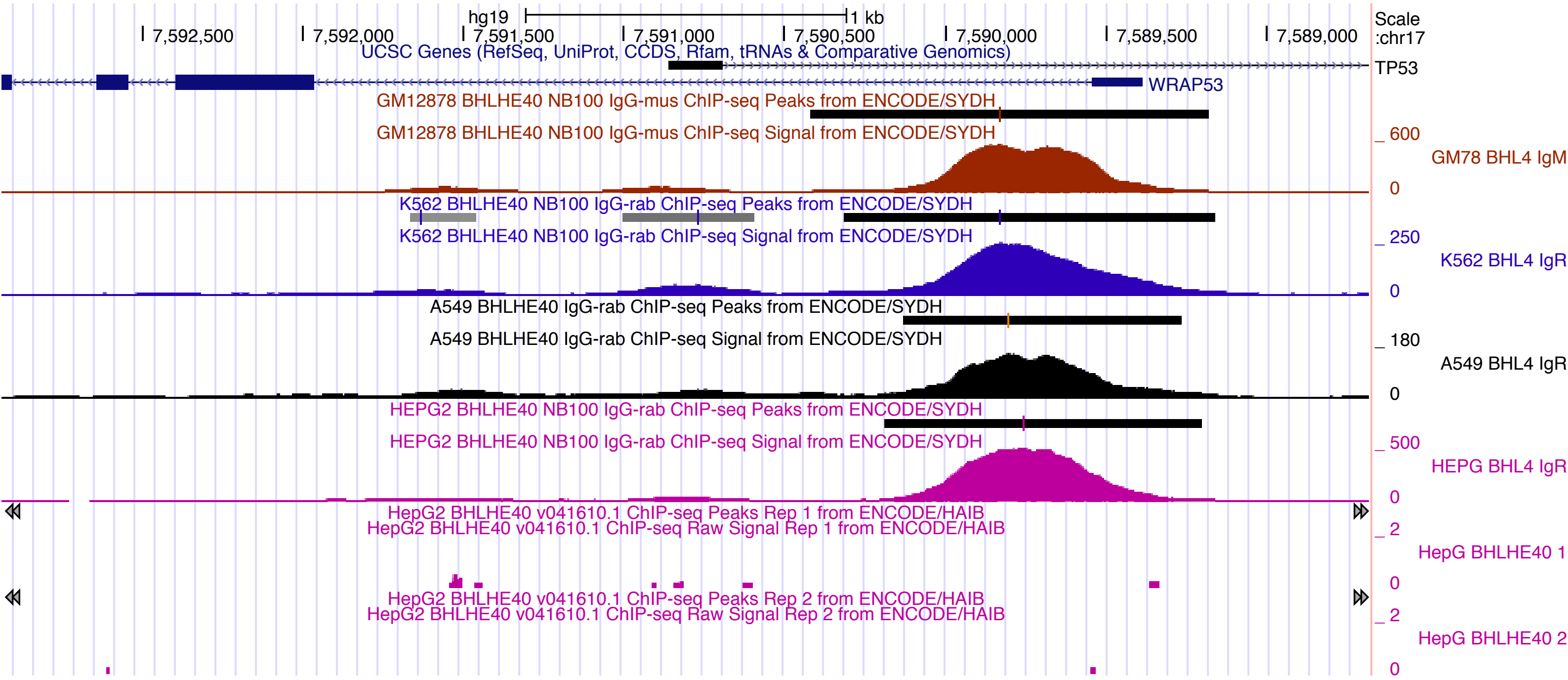

Scale
hg19
1 kb
chr17:
7,592,500
7,592,000
7,591,500
7,591,000
7,590,500
7,590,000
7,589,500
7,589,000
UCSC Genes (RefSeq, UniProt, CCDS, Rfam, tRNAs & Comparative Genomics)
TP53
WRAP53
GM12878 BHLHE40 NB100 IgG-mus ChIP-seq Peaks from ENCODE/SYDH
GM12878 BHLHE40 NB100 IgG-mus ChIP-seq Signal from ENCODE/SYDH
GM78 BHL4 IgM
K562 BHLHE40 NB100 IgG-rab ChIP-seq Peaks from ENCODE/SYDH
K562 BHLHE40 NB100 IgG-rab ChIP-seq Signal from ENCODE/SYDH
K562 BHL4 IgR
A549 BHLHE40 IgG-rab ChIP-seq Peaks from ENCODE/SYDH
A549 BHLHE40 IgG-rab ChIP-seq Signal from ENCODE/SYDH
A549 BHL4 IgR
HEPG2 BHLHE40 NB100 IgG-rab ChIP-seq Peaks from ENCODE/SYDH
HEPG2 BHLHE40 NB100 IgG-rab ChIP-seq Signal from ENCODE/SYDH
HEPG BHL4 IgR
HepG2 BHLHE40 v041610.1 ChIP-seq Peaks Rep 1 from ENCODE/HAIB
HepG2 BHLHE40 v041610.1 ChIP-seq Raw Signal Rep 1 from ENCODE/HAIB
HepG BHLHE40 1
HepG2 BHLHE40 v041610.1 ChIP-seq Peaks Rep 2 from ENCODE/HAIB
HepG2 BHLHE40 v041610.1 ChIP-seq Raw Signal Rep 2 from ENCODE/HAIB
HepG BHLHE40 2
600
250
180
500
2
0

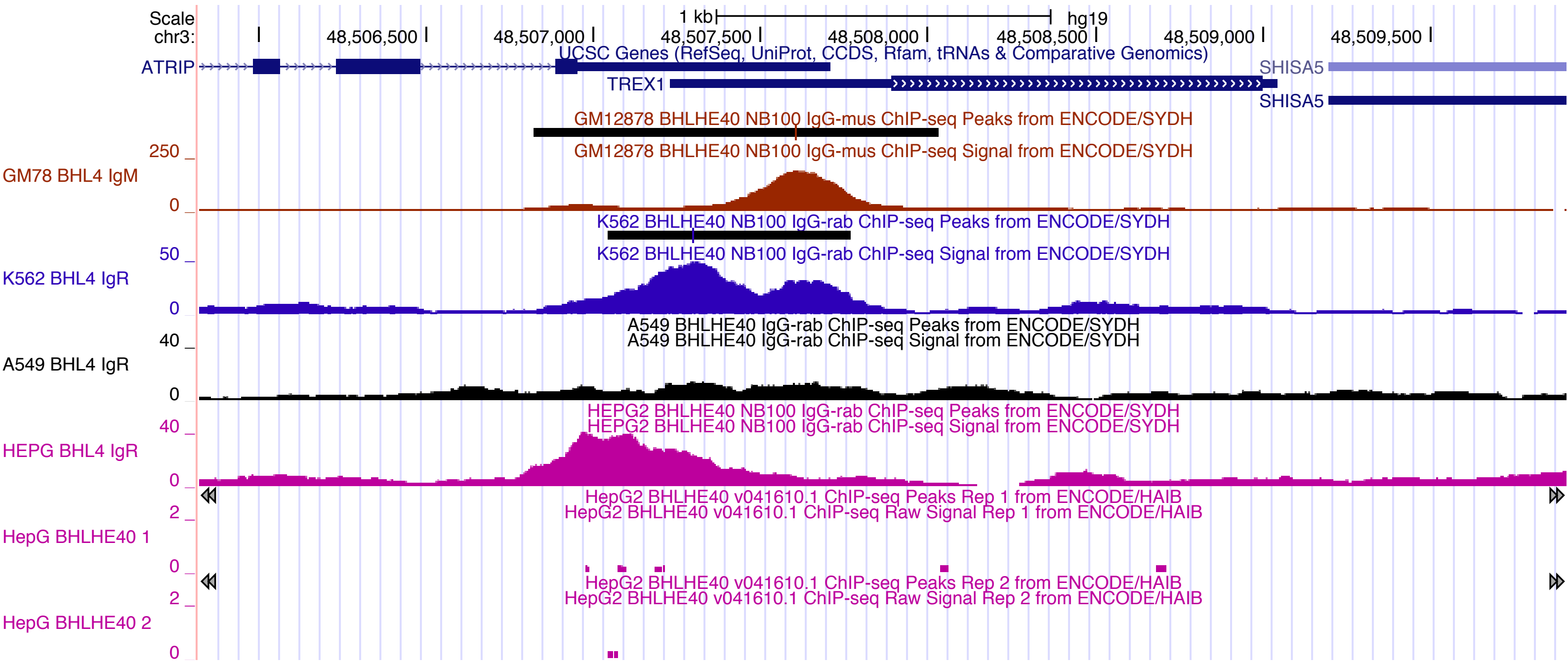

Scale
chr3:
1 kb
hg19
48,506,500
48,507,000
48,507,500
48,508,000
48,508,500
48,509,000
48,509,500
UCSC Genes (RefSeq, UniProt, CCDS, Rfam, tRNAs & Comparative Genomics)
ATRIP
TREX1
SHISA5
SHISA5
GM12878 BHLHE40 NB100 IgG-mus ChIP-seq Peaks from ENCODE/SYDH
GM12878 BHLHE40 NB100 IgG-mus ChIP-seq Signal from ENCODE/SYDH
GM78 BHL4 IgM
250
0
K562 BHLHE40 NB100 IgG-rab ChIP-seq Peaks from ENCODE/SYDH
K562 BHLHE40 NB100 IgG-rab ChIP-seq Signal from ENCODE/SYDH
K562 BHL4 IgR
50
0
A549 BHLHE40 IgG-rab ChIP-seq Peaks from ENCODE/SYDH
A549 BHLHE40 IgG-rab ChIP-seq Signal from ENCODE/SYDH
A549 BHL4 IgR
40
0
HEPG2 BHLHE40 NB100 IgG-rab ChIP-seq Peaks from ENCODE/SYDH
HEPG2 BHLHE40 NB100 IgG-rab ChIP-seq Signal from ENCODE/SYDH
HEPG BHL4 IgR
40
0
HepG2 BHLHE40 v041610.1 ChIP-seq Peaks Rep 1 from ENCODE/HAIB
HepG2 BHLHE40 v041610.1 ChIP-seq Raw Signal Rep 1 from ENCODE/HAIB
HepG BHLHE40 1
2
0
HepG2 BHLHE40 v041610.1 ChIP-seq Peaks Rep 2 from ENCODE/HAIB
HepG2 BHLHE40 v041610.1 ChIP-seq Raw Signal Rep 2 from ENCODE/HAIB
HepG BHLHE40 2
2
0

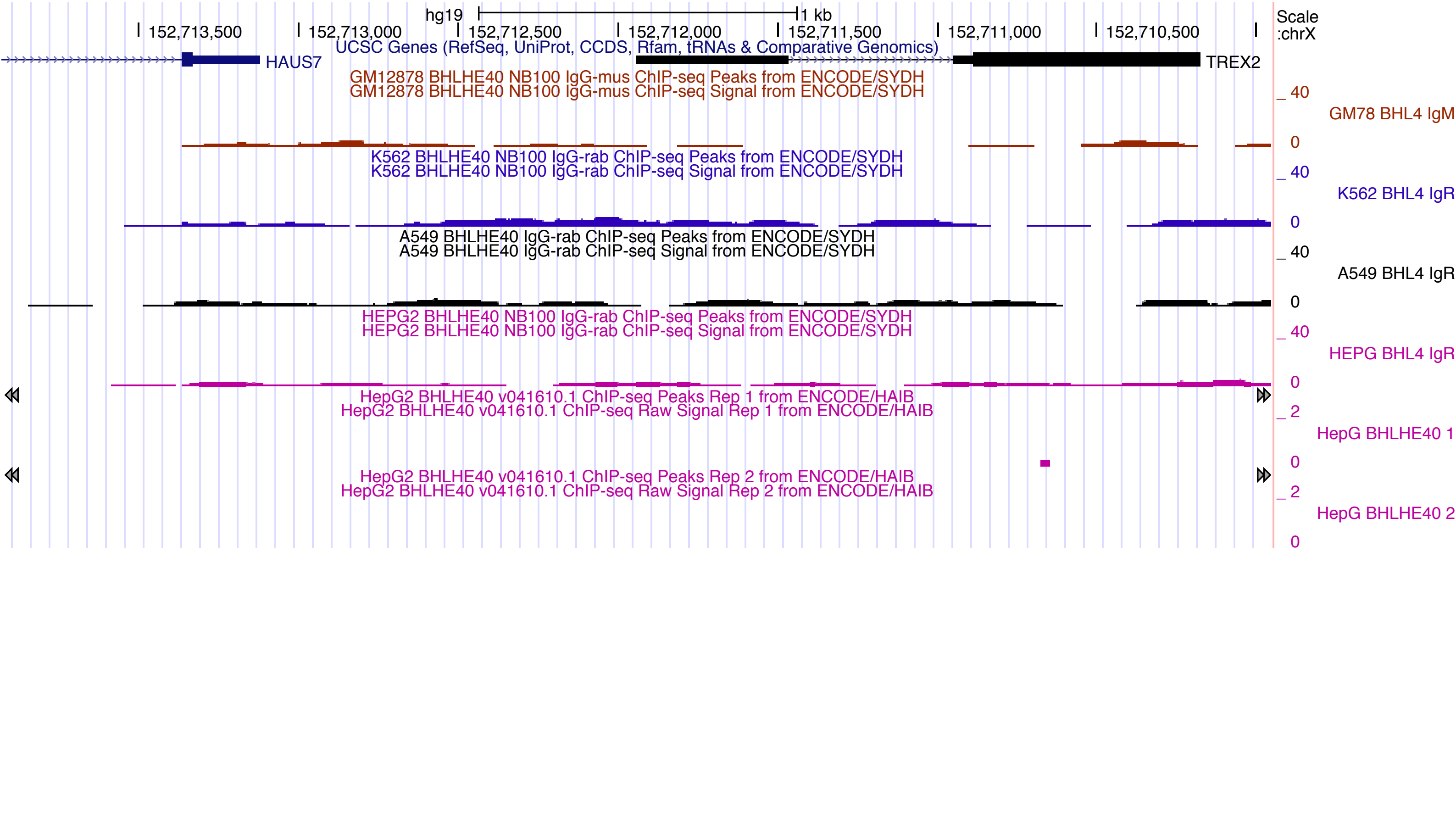

hg19
1 kb
Scale
:chrX
152,713,500
152,713,000
152,712,500
152,712,000
152,711,500
152,711,000
152,710,500
UCSC Genes (RefSeq, UniProt, CCDS, Rfam, tRNAs & Comparative Genomics)
HAUS7
TREX2
GM12878 BHLHE40 NB100 IgG-mus ChIP-seq Peaks from ENCODE/SYDH
GM12878 BHLHE40 NB100 IgG-mus ChIP-seq Signal from ENCODE/SYDH
GM78 BHL4 IgM
K562 BHLHE40 NB100 IgG-rab ChIP-seq Peaks from ENCODE/SYDH
K562 BHLHE40 NB100 IgG-rab ChIP-seq Signal from ENCODE/SYDH
K562 BHL4 IgR
A549 BHLHE40 IgG-rab ChIP-seq Peaks from ENCODE/SYDH
A549 BHLHE40 IgG-rab ChIP-seq Signal from ENCODE/SYDH
A549 BHL4 IgR
HEPG2 BHLHE40 NB100 IgG-rab ChIP-seq Peaks from ENCODE/SYDH
HEPG2 BHLHE40 NB100 IgG-rab ChIP-seq Signal from ENCODE/SYDH
HEPG BHL4 IgR
HepG2 BHLHE40 v041610.1 ChIP-seq Peaks Rep 1 from ENCODE/HAIB
HepG2 BHLHE40 v041610.1 ChIP-seq Raw Signal Rep 1 from ENCODE/HAIB
HepG BHLHE40 1
HepG2 BHLHE40 v041610.1 ChIP-seq Peaks Rep 2 from ENCODE/HAIB
HepG2 BHLHE40 v041610.1 ChIP-seq Raw Signal Rep 2 from ENCODE/HAIB
HepG BHLHE40 2
_ 40
0
_ 2

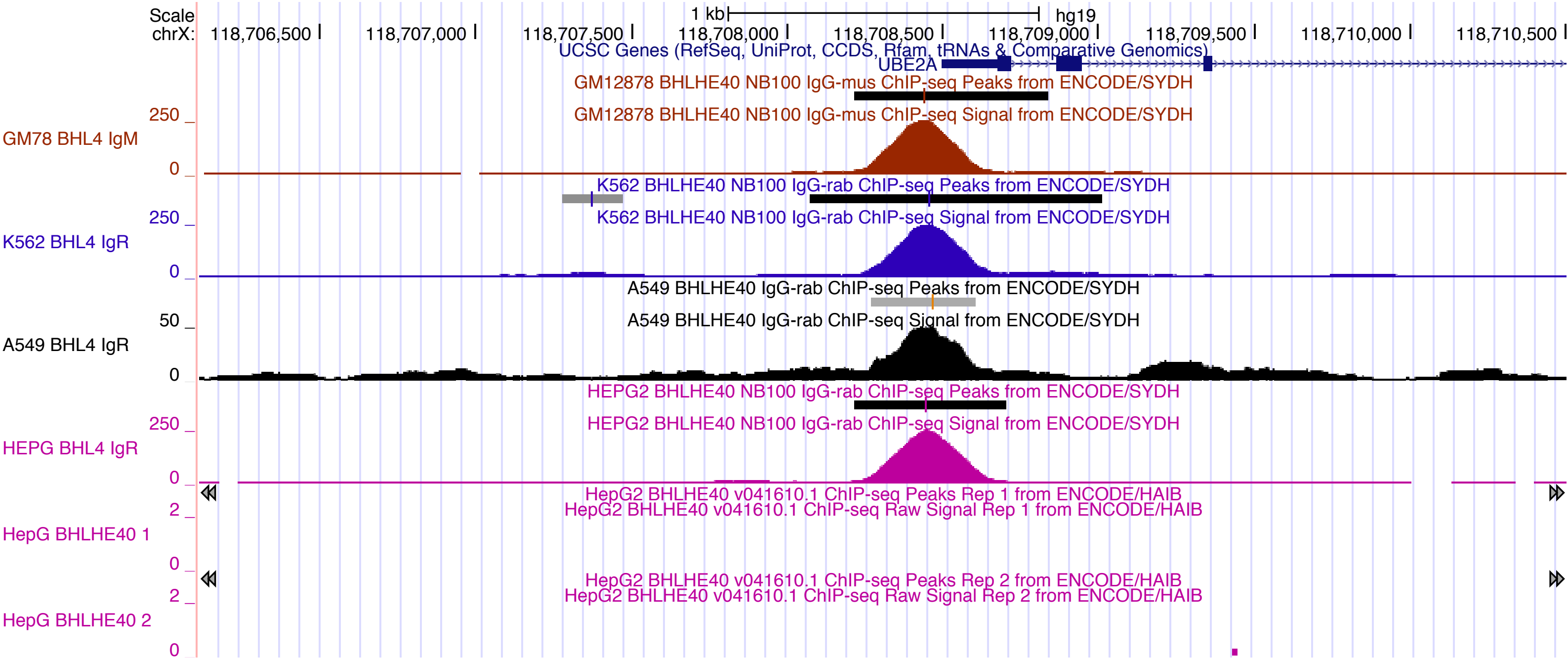

Scale
chrX:
1 kb
hg19
118,706,500
118,707,000
118,707,500
118,708,000
118,708,500
118,709,000
118,709,500
118,710,000
118,710,500
UCSC Genes (RefSeq, UniProt, CCDS, Rfam, tRNAs & Comparative Genomics)
UBE2A
GM12878 BHLHE40 NB100 IgG-mus ChIP-seq Peaks from ENCODE/SYDH
GM12878 BHLHE40 NB100 IgG-mus ChIP-seq Signal from ENCODE/SYDH
GM78 BHL4 IgM
250
0
K562 BHLHE40 NB100 IgG-rab ChIP-seq Peaks from ENCODE/SYDH
K562 BHLHE40 NB100 IgG-rab ChIP-seq Signal from ENCODE/SYDH
K562 BHL4 IgR
250
0
A549 BHLHE40 IgG-rab ChIP-seq Peaks from ENCODE/SYDH
A549 BHLHE40 IgG-rab ChIP-seq Signal from ENCODE/SYDH
A549 BHL4 IgR
50
0
HEPG2 BHLHE40 NB100 IgG-rab ChIP-seq Peaks from ENCODE/SYDH
HEPG2 BHLHE40 NB100 IgG-rab ChIP-seq Signal from ENCODE/SYDH
HEPG BHL4 IgR
250
0
HepG2 BHLHE40 v041610.1 ChIP-seq Peaks Rep 1 from ENCODE/HAIB
HepG2 BHLHE40 v041610.1 ChIP-seq Raw Signal Rep 1 from ENCODE/HAIB
HepG BHLHE40 1
2
0
HepG2 BHLHE40 v041610.1 ChIP-seq Peaks Rep 2 from ENCODE/HAIB
HepG2 BHLHE40 v041610.1 ChIP-seq Raw Signal Rep 2 from ENCODE/HAIB
HepG BHLHE40 2
2
0

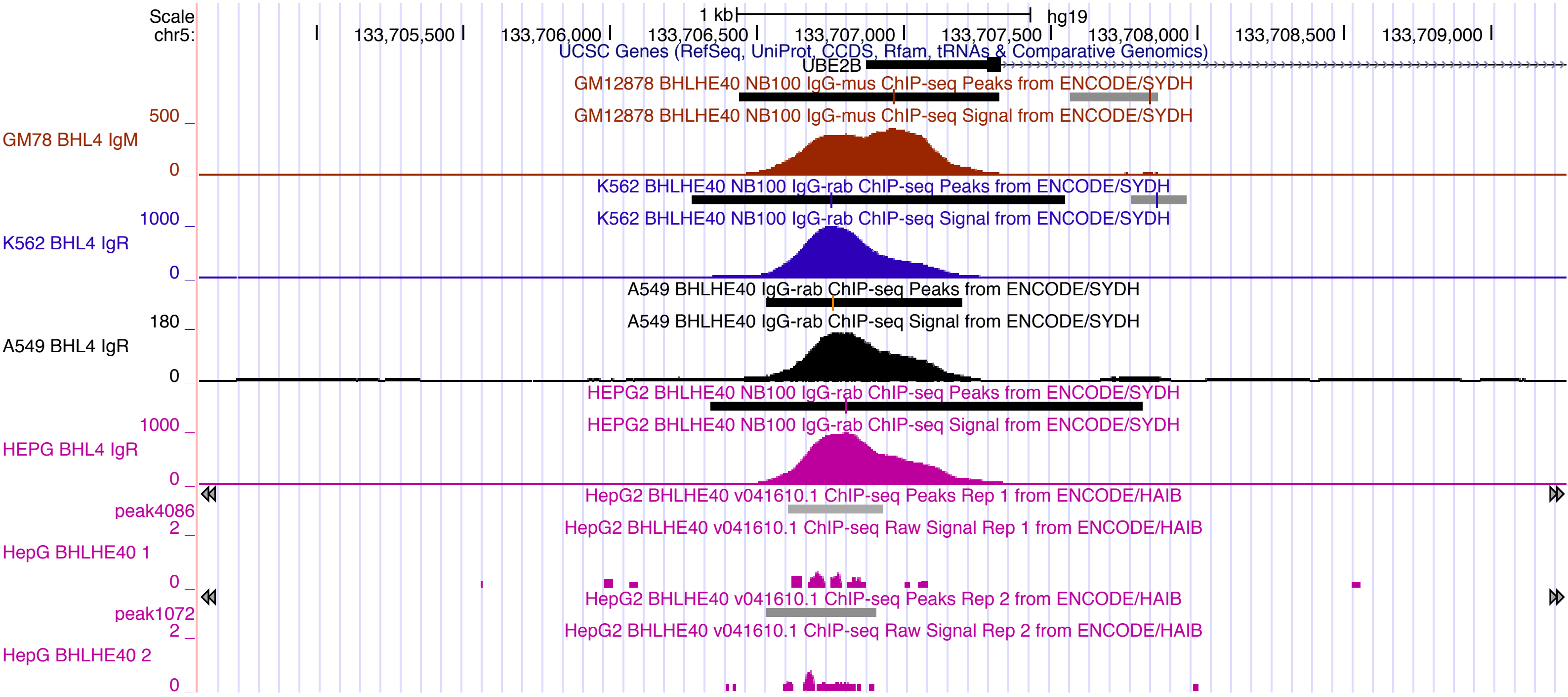

Scale
chr5:
1 kb
hg19
133,705,500
133,706,000
133,706,500
133,707,000
133,707,500
133,708,000
133,708,500
133,709,000
UCSC Genes (RefSeq, UniProt, CCDS, Rfam, tRNAs & Comparative Genomics)
UBE2B
GM12878 BHLHE40 NB100 IgG-mus ChIP-seq Peaks from ENCODE/SYDH
GM12878 BHLHE40 NB100 IgG-mus ChIP-seq Signal from ENCODE/SYDH
500
GM78 BHL4 IgM
0
K562 BHLHE40 NB100 IgG-rab ChIP-seq Peaks from ENCODE/SYDH
K562 BHLHE40 NB100 IgG-rab ChIP-seq Signal from ENCODE/SYDH
1000
K562 BHL4 IgR
0
A549 BHLHE40 IgG-rab ChIP-seq Peaks from ENCODE/SYDH
A549 BHLHE40 IgG-rab ChIP-seq Signal from ENCODE/SYDH
180
A549 BHL4 IgR
0
HEPG2 BHLHE40 NB100 IgG-rab ChIP-seq Peaks from ENCODE/SYDH
HEPG2 BHLHE40 NB100 IgG-rab ChIP-seq Signal from ENCODE/SYDH
1000
HEPG BHL4 IgR
0
HepG2 BHLHE40 v041610.1 ChIP-seq Peaks Rep 1 from ENCODE/HAIB
peak4086
2
HepG2 BHLHE40 v041610.1 ChIP-seq Raw Signal Rep 1 from ENCODE/HAIB
HepG BHLHE40 1
0
HepG2 BHLHE40 v041610.1 ChIP-seq Peaks Rep 2 from ENCODE/HAIB
peak1072
2
HepG2 BHLHE40 v041610.1 ChIP-seq Raw Signal Rep 2 from ENCODE/HAIB
HepG BHLHE40 2
0

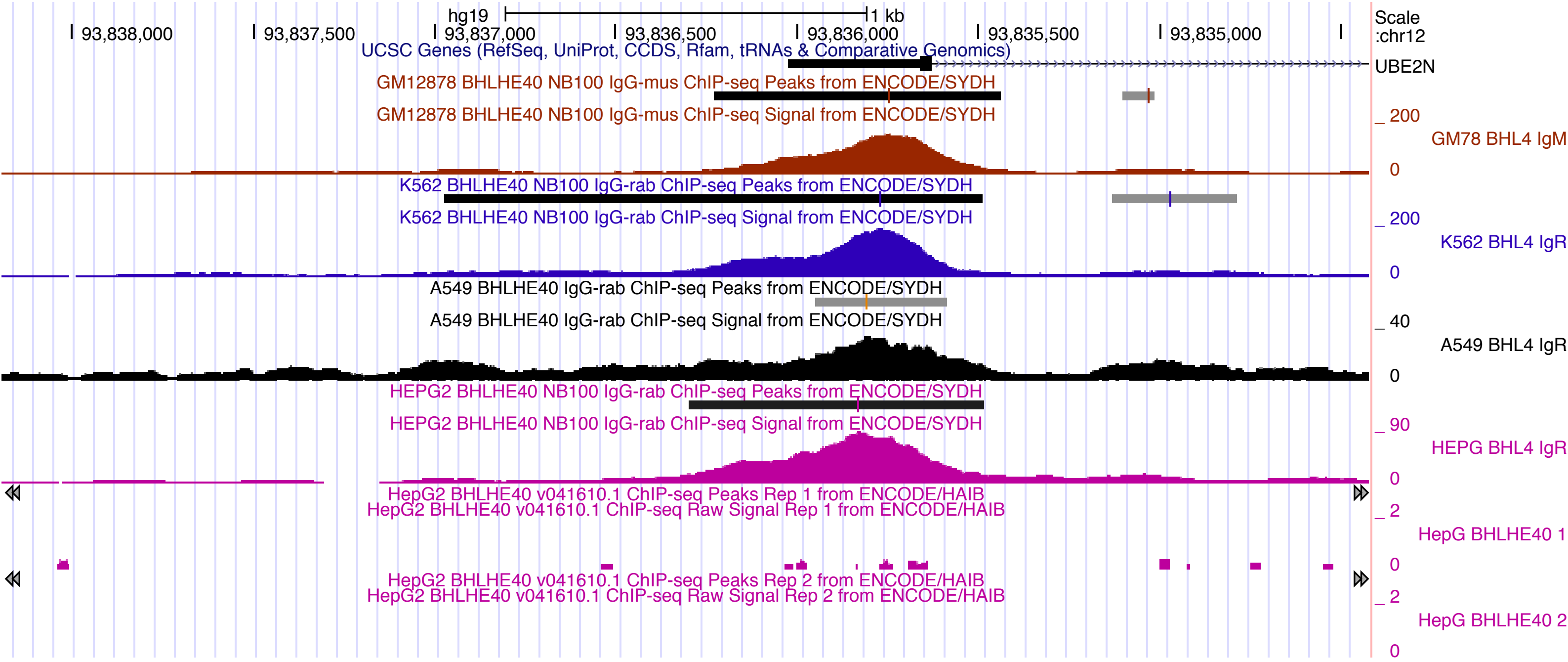
hg19
1 kb
Scale
:chr12
93,838,000
93,837,500
93,837,000
93,836,500
93,836,000
93,835,500
93,835,000
UCSC Genes (RefSeq, UniProt, CCDS, Rfam, tRNAs & Comparative Genomics)
UBE2N
GM12878 BHLHE40 NB100 IgG-mus ChIP-seq Peaks from ENCODE/SYDH
GM12878 BHLHE40 NB100 IgG-mus ChIP-seq Signal from ENCODE/SYDH
_ 200
GM78 BHL4 IgM
0
K562 BHLHE40 NB100 IgG-rab ChIP-seq Peaks from ENCODE/SYDH
K562 BHLHE40 NB100 IgG-rab ChIP-seq Signal from ENCODE/SYDH
_ 200
K562 BHL4 IgR
0
A549 BHLHE40 IgG-rab ChIP-seq Peaks from ENCODE/SYDH
A549 BHLHE40 IgG-rab ChIP-seq Signal from ENCODE/SYDH
_ 40
A549 BHL4 IgR
0
HEPG2 BHLHE40 NB100 IgG-rab ChIP-seq Peaks from ENCODE/SYDH
HEPG2 BHLHE40 NB100 IgG-rab ChIP-seq Signal from ENCODE/SYDH
_ 90
HEPG BHL4 IgR
0
HepG2 BHLHE40 v041610.1 ChIP-seq Peaks Rep 1 from ENCODE/HAIB
HepG2 BHLHE40 v041610.1 ChIP-seq Raw Signal Rep 1 from ENCODE/HAIB
_ 2
HepG BHLHE40 1
0
HepG2 BHLHE40 v041610.1 ChIP-seq Peaks Rep 2 from ENCODE/HAIB
HepG2 BHLHE40 v041610.1 ChIP-seq Raw Signal Rep 2 from ENCODE/HAIB
_ 2
HepG BHLHE40 2
0

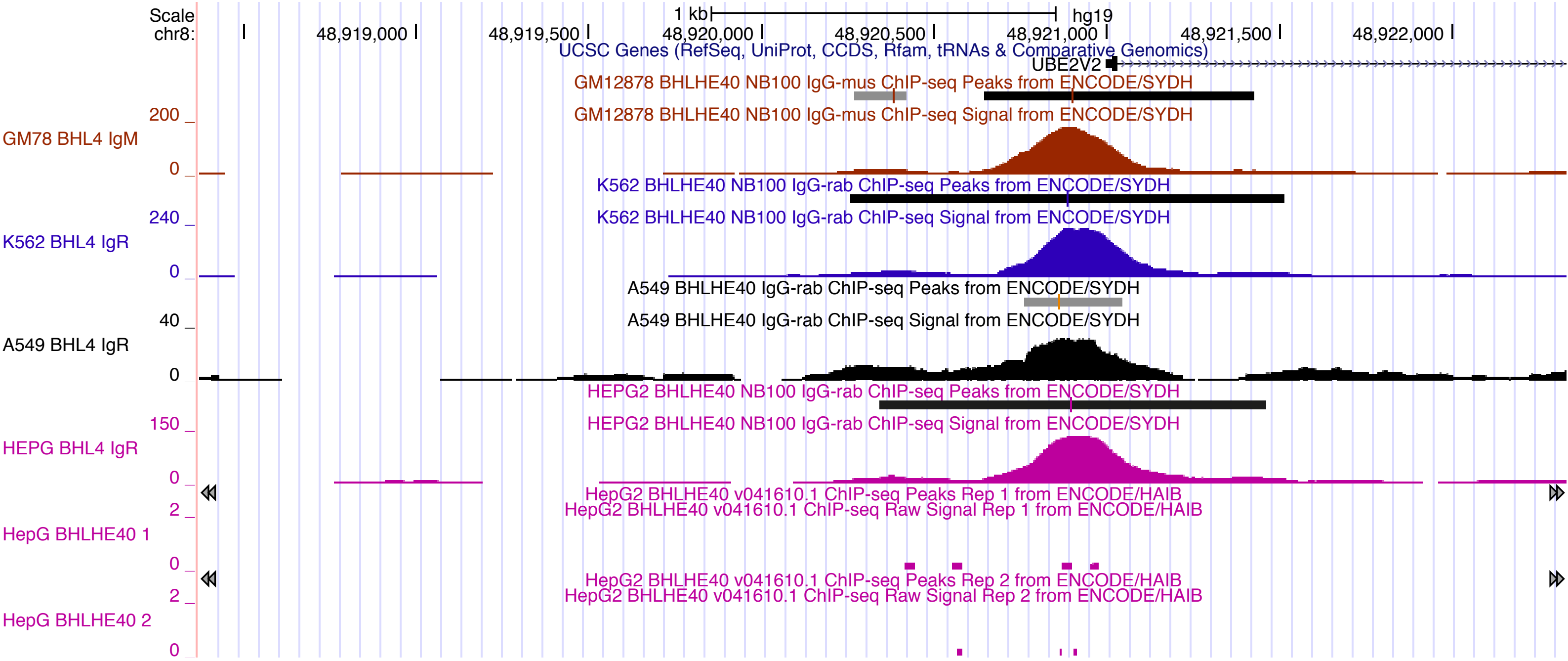
Scale
chr8:
1 kb
hg19
48,919,000
48,919,500
48,920,000
48,920,500
48,921,000
48,921,500
48,922,000
UCSC Genes (RefSeq, UniProt, CCDS, Rfam, tRNAs & Comparative Genomics)
UBE2V2
GM12878 BHLHE40 NB100 IgG-mus ChIP-seq Peaks from ENCODE/SYDH
GM12878 BHLHE40 NB100 IgG-mus ChIP-seq Signal from ENCODE/SYDH
200
GM78 BHL4 IgM
0
K562 BHLHE40 NB100 IgG-rab ChIP-seq Peaks from ENCODE/SYDH
K562 BHLHE40 NB100 IgG-rab ChIP-seq Signal from ENCODE/SYDH
240
K562 BHL4 IgR
0
A549 BHLHE40 IgG-rab ChIP-seq Peaks from ENCODE/SYDH
A549 BHLHE40 IgG-rab ChIP-seq Signal from ENCODE/SYDH
40
A549 BHL4 IgR
0
HEPG2 BHLHE40 NB100 IgG-rab ChIP-seq Peaks from ENCODE/SYDH
HEPG2 BHLHE40 NB100 IgG-rab ChIP-seq Signal from ENCODE/SYDH
150
HEPG BHL4 IgR
0
HepG2 BHLHE40 v041610.1 ChIP-seq Peaks Rep 1 from ENCODE/HAIB
HepG2 BHLHE40 v041610.1 ChIP-seq Raw Signal Rep 1 from ENCODE/HAIB
2
HepG BHLHE40 1
0
HepG2 BHLHE40 v041610.1 ChIP-seq Peaks Rep 2 from ENCODE/HAIB
HepG2 BHLHE40 v041610.1 ChIP-seq Raw Signal Rep 2 from ENCODE/HAIB
2
HepG BHLHE40 2
0

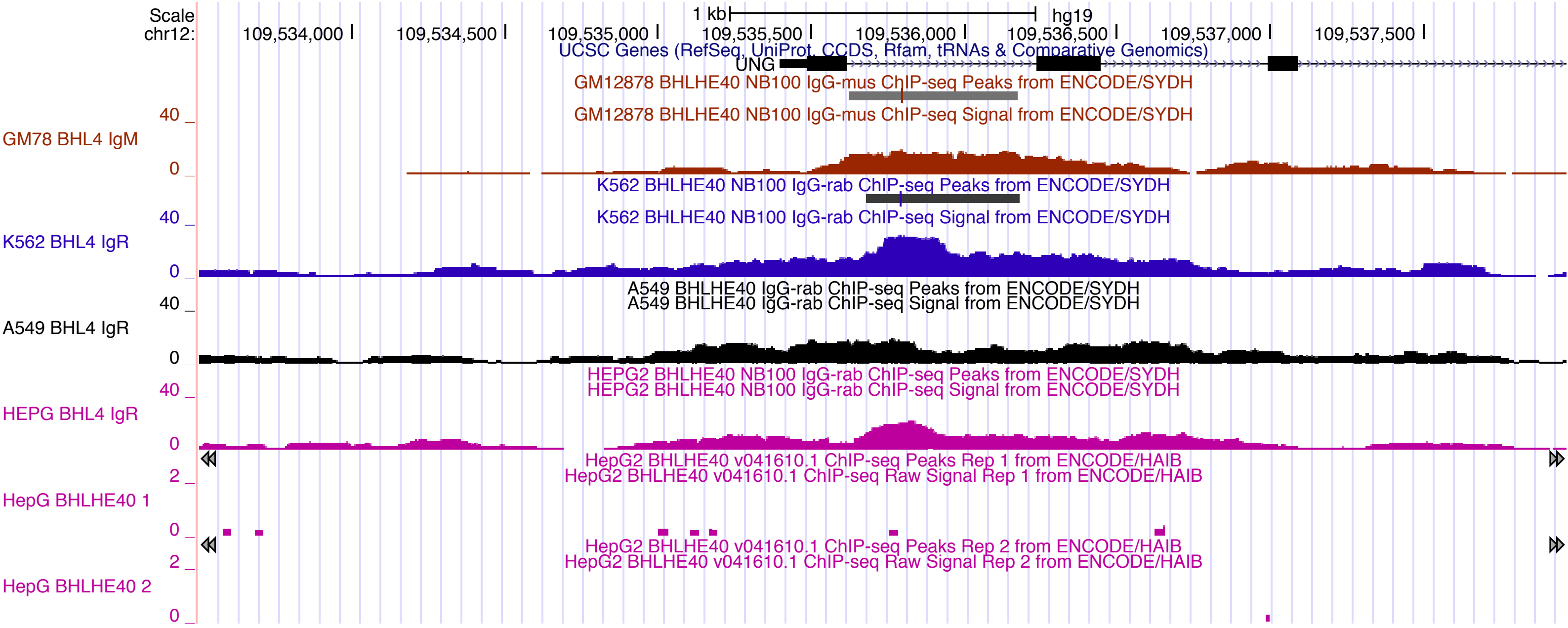
Scale
chr12:
1 kb
hg19
109,534,000
109,534,500
109,535,000
109,535,500
109,536,000
109,536,500
109,537,000
109,537,500
UCSC Genes (RefSeq, UniProt, CCDS, Rfam, tRNAs & Comparative Genomics)
UNG
GM12878 BHLHE40 NB100 IgG-mus ChIP-seq Peaks from ENCODE/SYDH
GM12878 BHLHE40 NB100 IgG-mus ChIP-seq Signal from ENCODE/SYDH
GM78 BHL4 IgM
K562 BHLHE40 NB100 IgG-rab ChIP-seq Peaks from ENCODE/SYDH
K562 BHLHE40 NB100 IgG-rab ChIP-seq Signal from ENCODE/SYDH
K562 BHL4 IgR
A549 BHLHE40 IgG-rab ChIP-seq Peaks from ENCODE/SYDH
A549 BHLHE40 IgG-rab ChIP-seq Signal from ENCODE/SYDH
A549 BHL4 IgR
HEPG2 BHLHE40 NB100 IgG-rab ChIP-seq Peaks from ENCODE/SYDH
HEPG2 BHLHE40 NB100 IgG-rab ChIP-seq Signal from ENCODE/SYDH
HEPG BHL4 IgR
HepG2 BHLHE40 v041610.1 ChIP-seq Peaks Rep 1 from ENCODE/HAIB
HepG2 BHLHE40 v041610.1 ChIP-seq Raw Signal Rep 1 from ENCODE/HAIB
HepG BHLHE40 1
HepG2 BHLHE40 v041610.1 ChIP-seq Peaks Rep 2 from ENCODE/HAIB
HepG2 BHLHE40 v041610.1 ChIP-seq Raw Signal Rep 2 from ENCODE/HAIB
HepG BHLHE40 2

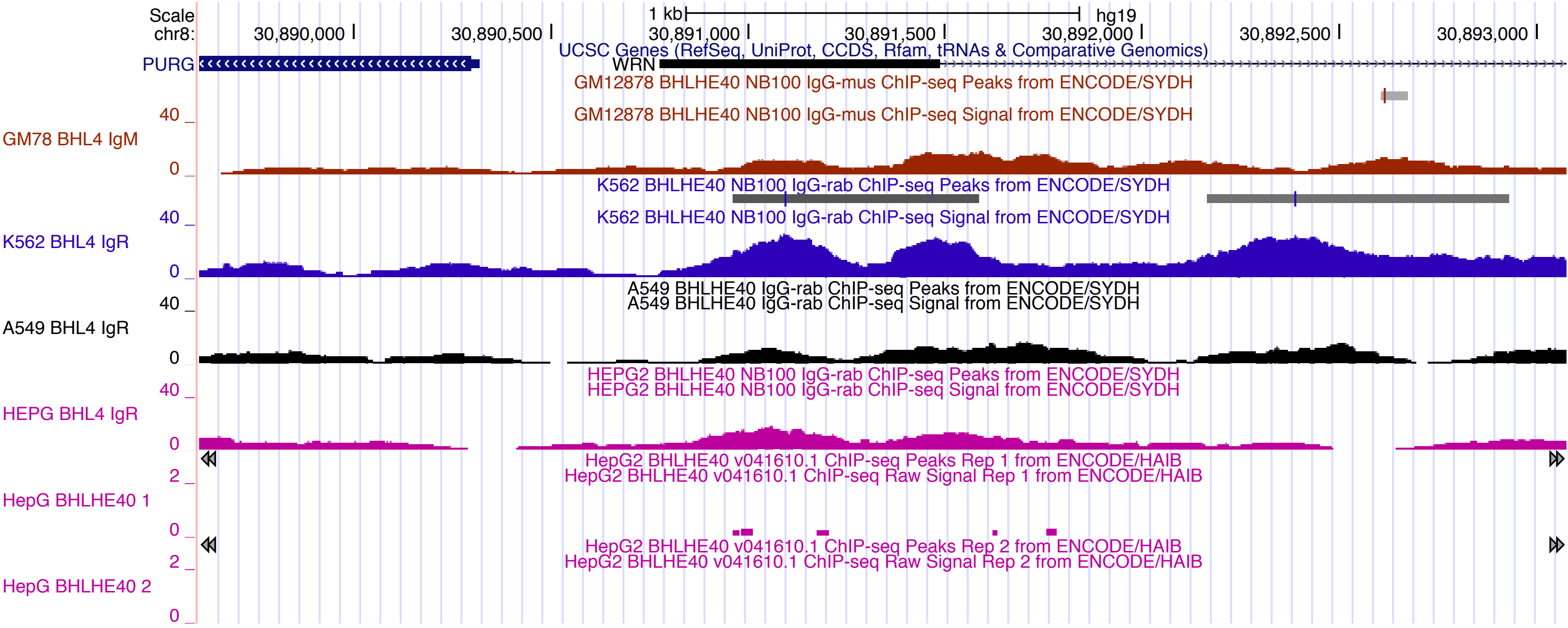
Scale
chr8:
1 kb
hg19
30,890,000
30,890,500
30,891,000
30,891,500
30,892,000
30,892,500
30,893,000
UCSC Genes (RefSeq, UniProt, CCDS, Rfam, tRNAs & Comparative Genomics)
PURG
WRN
GM12878 BHLHE40 NB100 IgG-mus ChIP-seq Peaks from ENCODE/SYDH
GM12878 BHLHE40 NB100 IgG-mus ChIP-seq Signal from ENCODE/SYDH
GM78 BHL4 IgM
40
0
K562 BHLHE40 NB100 IgG-rab ChIP-seq Peaks from ENCODE/SYDH
K562 BHLHE40 NB100 IgG-rab ChIP-seq Signal from ENCODE/SYDH
K562 BHL4 IgR
40
0
A549 BHLHE40 IgG-rab ChIP-seq Peaks from ENCODE/SYDH
A549 BHLHE40 IgG-rab ChIP-seq Signal from ENCODE/SYDH
A549 BHL4 IgR
40
0
HEPG2 BHLHE40 NB100 IgG-rab ChIP-seq Peaks from ENCODE/SYDH
HEPG2 BHLHE40 NB100 IgG-rab ChIP-seq Signal from ENCODE/SYDH
HEPG BHL4 IgR
40
0
HepG2 BHLHE40 v041610.1 ChIP-seq Peaks Rep 1 from ENCODE/HAIB
HepG2 BHLHE40 v041610.1 ChIP-seq Raw Signal Rep 1 from ENCODE/HAIB
HepG BHLHE40 1
2
0
HepG2 BHLHE40 v041610.1 ChIP-seq Peaks Rep 2 from ENCODE/HAIB
HepG2 BHLHE40 v041610.1 ChIP-seq Raw Signal Rep 2 from ENCODE/HAIB
HepG BHLHE40 2
2
0

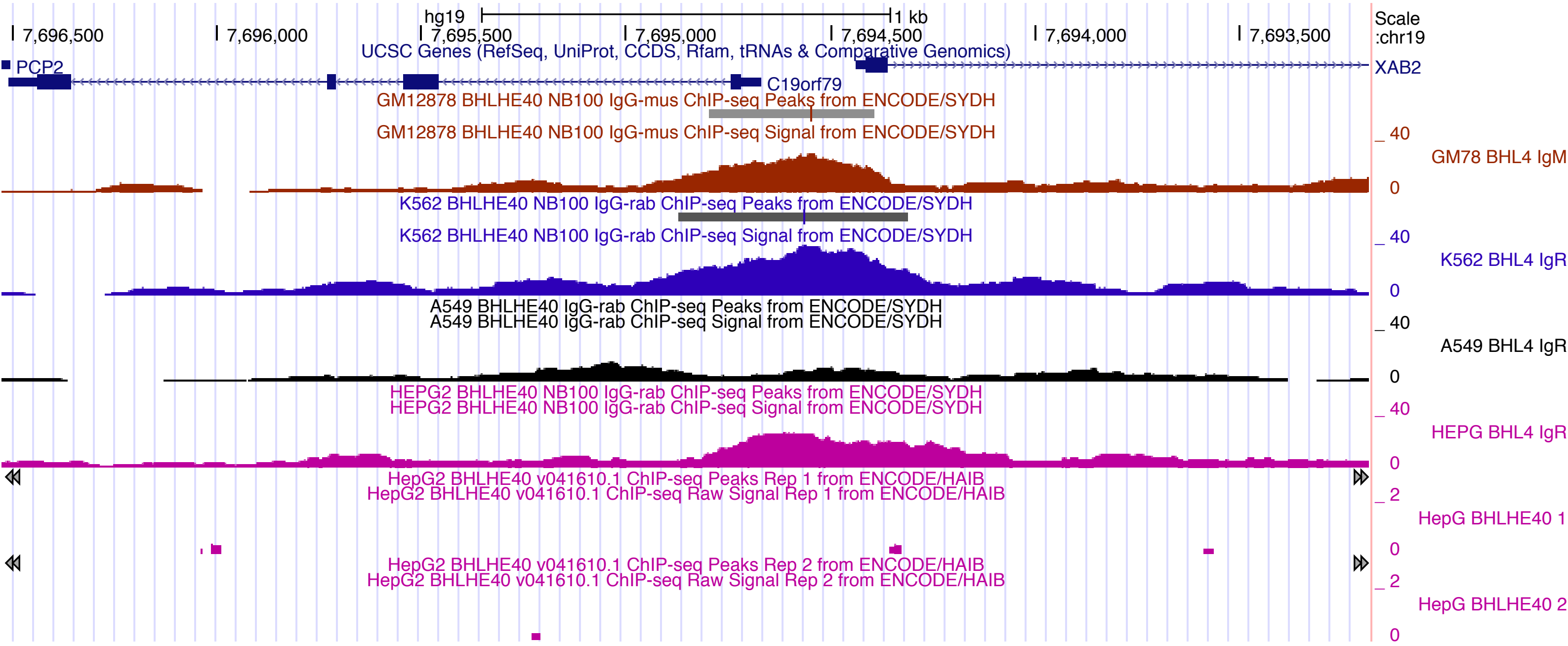

hg19
1 kb
Scale
:chr19
7,696,500
7,696,000
7,695,500
7,695,000
7,694,500
7,694,000
7,693,500
UCSC Genes (RefSeq, UniProt, CCDS, Rfam, tRNAs & Comparative Genomics)
PCP2
C19orf79
XAB2
GM12878 BHLHE40 NB100 IgG-mus ChIP-seq Peaks from ENCODE/SYDH
GM12878 BHLHE40 NB100 IgG-mus ChIP-seq Signal from ENCODE/SYDH
GM78 BHL4 IgM
K562 BHLHE40 NB100 IgG-rab ChIP-seq Peaks from ENCODE/SYDH
K562 BHLHE40 NB100 IgG-rab ChIP-seq Signal from ENCODE/SYDH
K562 BHL4 IgR
A549 BHLHE40 IgG-rab ChIP-seq Peaks from ENCODE/SYDH
A549 BHLHE40 IgG-rab ChIP-seq Signal from ENCODE/SYDH
A549 BHL4 IgR
HEPG2 BHLHE40 NB100 IgG-rab ChIP-seq Peaks from ENCODE/SYDH
HEPG2 BHLHE40 NB100 IgG-rab ChIP-seq Signal from ENCODE/SYDH
HEPG BHL4 IgR
HepG2 BHLHE40 v041610.1 ChIP-seq Peaks Rep 1 from ENCODE/HAIB
HepG2 BHLHE40 v041610.1 ChIP-seq Raw Signal Rep 1 from ENCODE/HAIB
HepG BHLHE40 1
HepG2 BHLHE40 v041610.1 ChIP-seq Peaks Rep 2 from ENCODE/HAIB
HepG2 BHLHE40 v041610.1 ChIP-seq Raw Signal Rep 2 from ENCODE/HAIB
HepG BHLHE40 2
40
0
2

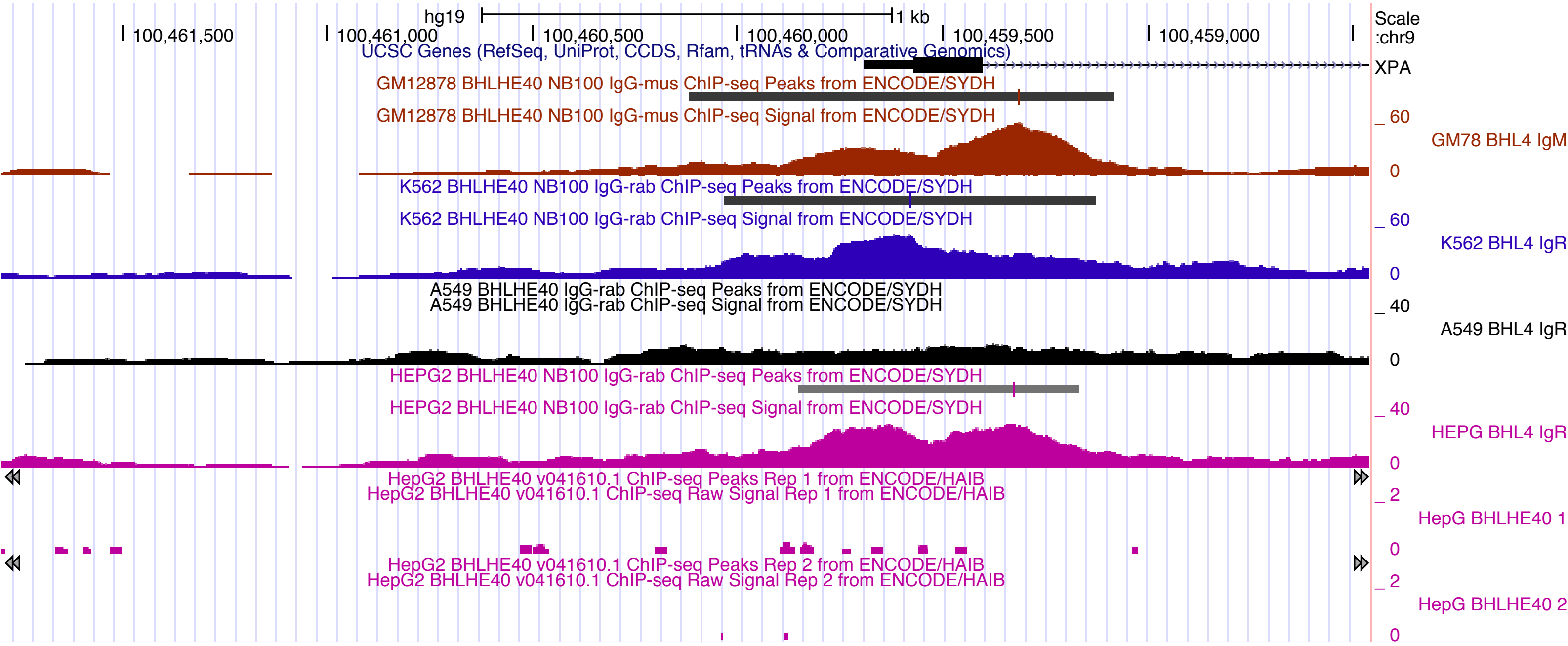
hg19
1 kb
Scale
:chr9
100,461,500
100,461,000
100,460,500
100,460,000
100,459,500
100,459,000
UCSC Genes (RefSeq, UniProt, CCDS, Rfam, tRNAs & Comparative Genomics)
XPA
GM12878 BHLHE40 NB100 IgG-mus ChIP-seq Peaks from ENCODE/SYDH
GM12878 BHLHE40 NB100 IgG-mus ChIP-seq Signal from ENCODE/SYDH
_ 60
GM78 BHL4 IgM
0
K562 BHLHE40 NB100 IgG-rab ChIP-seq Peaks from ENCODE/SYDH
K562 BHLHE40 NB100 IgG-rab ChIP-seq Signal from ENCODE/SYDH
_ 60
K562 BHL4 IgR
0
A549 BHLHE40 IgG-rab ChIP-seq Peaks from ENCODE/SYDH
A549 BHLHE40 IgG-rab ChIP-seq Signal from ENCODE/SYDH
_ 40
A549 BHL4 IgR
0
HEPG2 BHLHE40 NB100 IgG-rab ChIP-seq Peaks from ENCODE/SYDH
HEPG2 BHLHE40 NB100 IgG-rab ChIP-seq Signal from ENCODE/SYDH
_ 40
HEPG BHL4 IgR
0
HepG2 BHLHE40 v041610.1 ChIP-seq Peaks Rep 1 from ENCODE/HAIB
HepG2 BHLHE40 v041610.1 ChIP-seq Raw Signal Rep 1 from ENCODE/HAIB
_ 2
HepG BHLHE40 1
0
HepG2 BHLHE40 v041610.1 ChIP-seq Peaks Rep 2 from ENCODE/HAIB
HepG2 BHLHE40 v041610.1 ChIP-seq Raw Signal Rep 2 from ENCODE/HAIB
_ 2
HepG BHLHE40 2
0

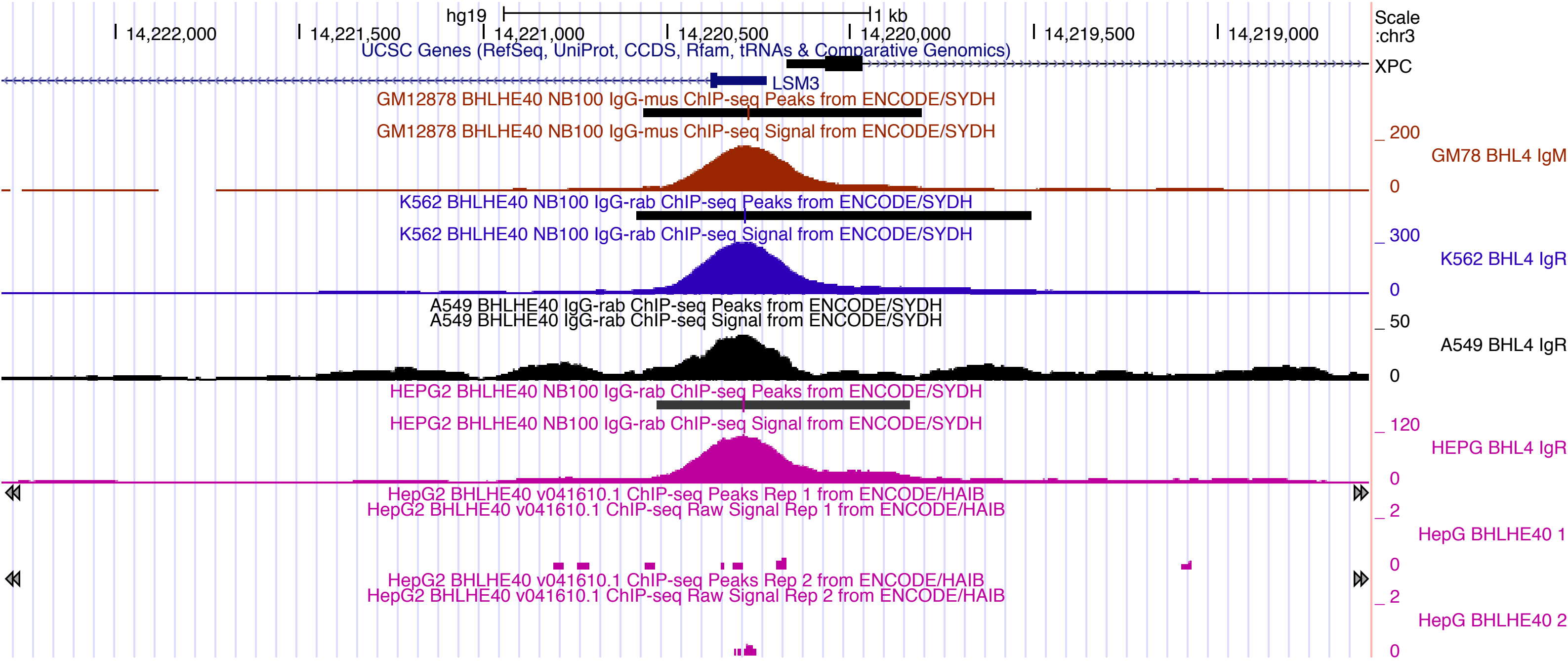
hg19
1 kb
Scale
:chr3
14,222,000
14,221,500
14,221,000
14,220,500
14,220,000
14,219,500
14,219,000
UCSC Genes (RefSeq, UniProt, CCDS, Rfam, tRNAs & Comparative Genomics)
XPC
LSM3
GM12878 BHLHE40 NB100 IgG-mus ChIP-seq Peaks from ENCODE/SYDH
GM12878 BHLHE40 NB100 IgG-mus ChIP-seq Signal from ENCODE/SYDH
200
GM78 BHL4 IgM
0
K562 BHLHE40 NB100 IgG-rab ChIP-seq Peaks from ENCODE/SYDH
K562 BHLHE40 NB100 IgG-rab ChIP-seq Signal from ENCODE/SYDH
300
K562 BHL4 IgR
0
A549 BHLHE40 IgG-rab ChIP-seq Peaks from ENCODE/SYDH
A549 BHLHE40 IgG-rab ChIP-seq Signal from ENCODE/SYDH
50
A549 BHL4 IgR
0
HEPG2 BHLHE40 NB100 IgG-rab ChIP-seq Peaks from ENCODE/SYDH
HEPG2 BHLHE40 NB100 IgG-rab ChIP-seq Signal from ENCODE/SYDH
120
HEPG BHL4 IgR
0
HepG2 BHLHE40 v041610.1 ChIP-seq Peaks Rep 1 from ENCODE/HAIB
HepG2 BHLHE40 v041610.1 ChIP-seq Raw Signal Rep 1 from ENCODE/HAIB
2
HepG BHLHE40 1
0
HepG2 BHLHE40 v041610.1 ChIP-seq Peaks Rep 2 from ENCODE/HAIB
HepG2 BHLHE40 v041610.1 ChIP-seq Raw Signal Rep 2 from ENCODE/HAIB
2
HepG BHLHE40 2
0

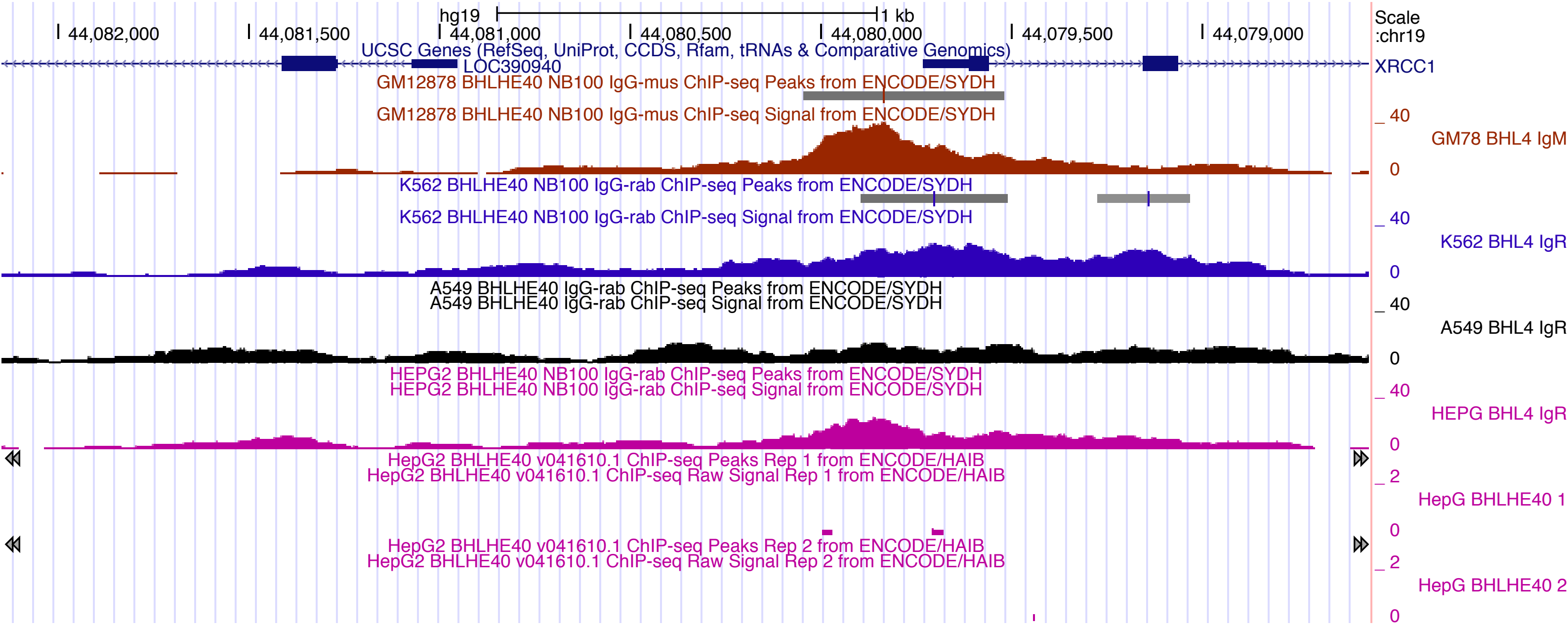

hg19
1 kb
Scale
:chr19
44,082,000
44,081,500
44,081,000
44,080,500
44,080,000
44,079,500
44,079,000
UCSC Genes (RefSeq, UniProt, CCDS, Rfam, tRNAs & Comparative Genomics)
LOC390940
XRCC1
GM12878 BHLHE40 NB100 IgG-mus ChIP-seq Peaks from ENCODE/SYDH
GM12878 BHLHE40 NB100 IgG-mus ChIP-seq Signal from ENCODE/SYDH
GM78 BHL4 IgM
K562 BHLHE40 NB100 IgG-rab ChIP-seq Peaks from ENCODE/SYDH
K562 BHLHE40 NB100 IgG-rab ChIP-seq Signal from ENCODE/SYDH
K562 BHL4 IgR
A549 BHLHE40 IgG-rab ChIP-seq Peaks from ENCODE/SYDH
A549 BHLHE40 IgG-rab ChIP-seq Signal from ENCODE/SYDH
A549 BHL4 IgR
HEPG2 BHLHE40 NB100 IgG-rab ChIP-seq Peaks from ENCODE/SYDH
HEPG2 BHLHE40 NB100 IgG-rab ChIP-seq Signal from ENCODE/SYDH
HEPG BHL4 IgR
HepG2 BHLHE40 v041610.1 ChIP-seq Peaks Rep 1 from ENCODE/HAIB
HepG2 BHLHE40 v041610.1 ChIP-seq Raw Signal Rep 1 from ENCODE/HAIB
HepG BHLHE40 1
HepG2 BHLHE40 v041610.1 ChIP-seq Peaks Rep 2 from ENCODE/HAIB
HepG2 BHLHE40 v041610.1 ChIP-seq Raw Signal Rep 2 from ENCODE/HAIB
HepG BHLHE40 2
_ 40
0
_ 2

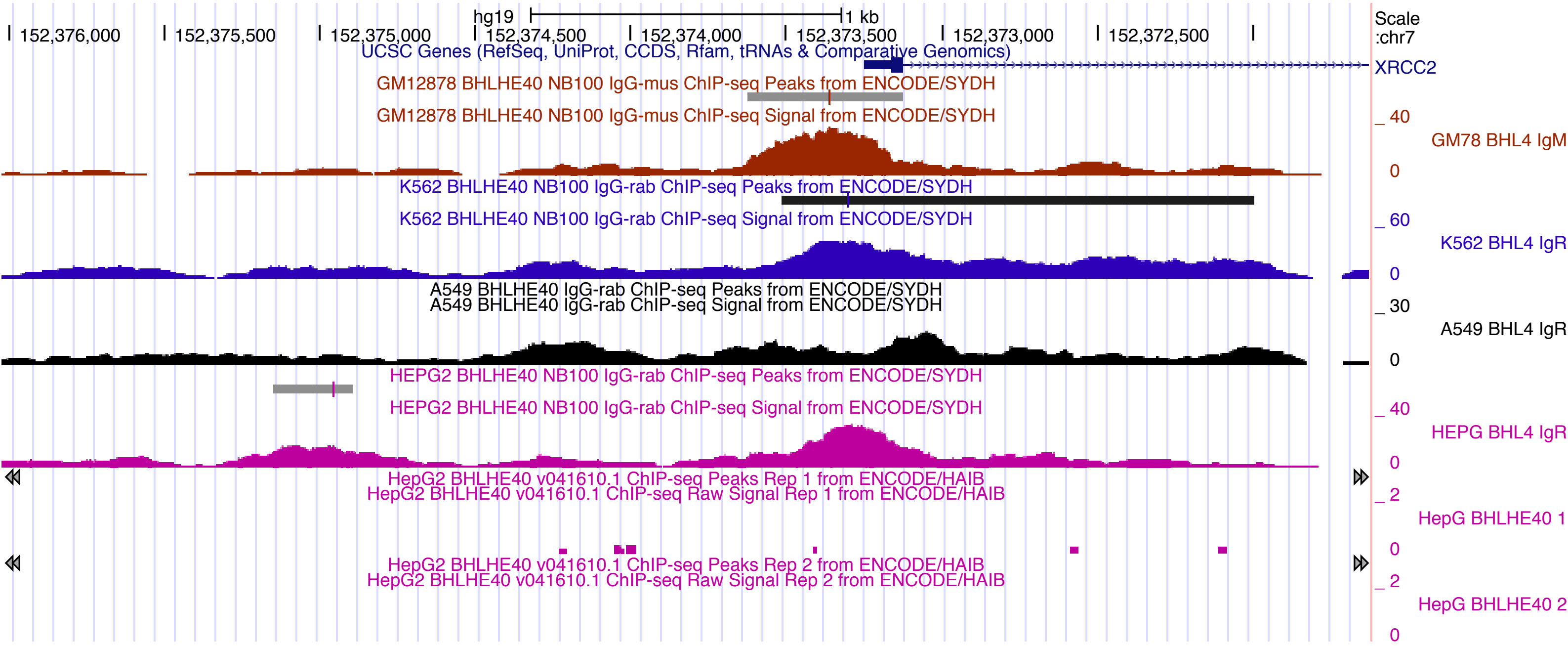

hg19
1 kb
Scale
:chr7
152,376,000
152,375,500
152,375,000
152,374,500
152,374,000
152,373,500
152,373,000
152,372,500
UCSC Genes (RefSeq, UniProt, CCDS, Rfam, tRNAs & Comparative Genomics)
XRCC2
GM12878 BHLHE40 NB100 IgG-mus ChIP-seq Peaks from ENCODE/SYDH
GM12878 BHLHE40 NB100 IgG-mus ChIP-seq Signal from ENCODE/SYDH
_ 40
GM78 BHL4 IgM
0
K562 BHLHE40 NB100 IgG-rab ChIP-seq Peaks from ENCODE/SYDH
K562 BHLHE40 NB100 IgG-rab ChIP-seq Signal from ENCODE/SYDH
_ 60
K562 BHL4 IgR
0
A549 BHLHE40 IgG-rab ChIP-seq Peaks from ENCODE/SYDH
A549 BHLHE40 IgG-rab ChIP-seq Signal from ENCODE/SYDH
_ 30
A549 BHL4 IgR
0
HEPG2 BHLHE40 NB100 IgG-rab ChIP-seq Peaks from ENCODE/SYDH
HEPG2 BHLHE40 NB100 IgG-rab ChIP-seq Signal from ENCODE/SYDH
_ 40
HEPG BHL4 IgR
0
HepG2 BHLHE40 v041610.1 ChIP-seq Peaks Rep 1 from ENCODE/HAIB
HepG2 BHLHE40 v041610.1 ChIP-seq Raw Signal Rep 1 from ENCODE/HAIB
_ 2
HepG BHLHE40 1
0
HepG2 BHLHE40 v041610.1 ChIP-seq Peaks Rep 2 from ENCODE/HAIB
HepG2 BHLHE40 v041610.1 ChIP-seq Raw Signal Rep 2 from ENCODE/HAIB
_ 2
HepG BHLHE40 2
0

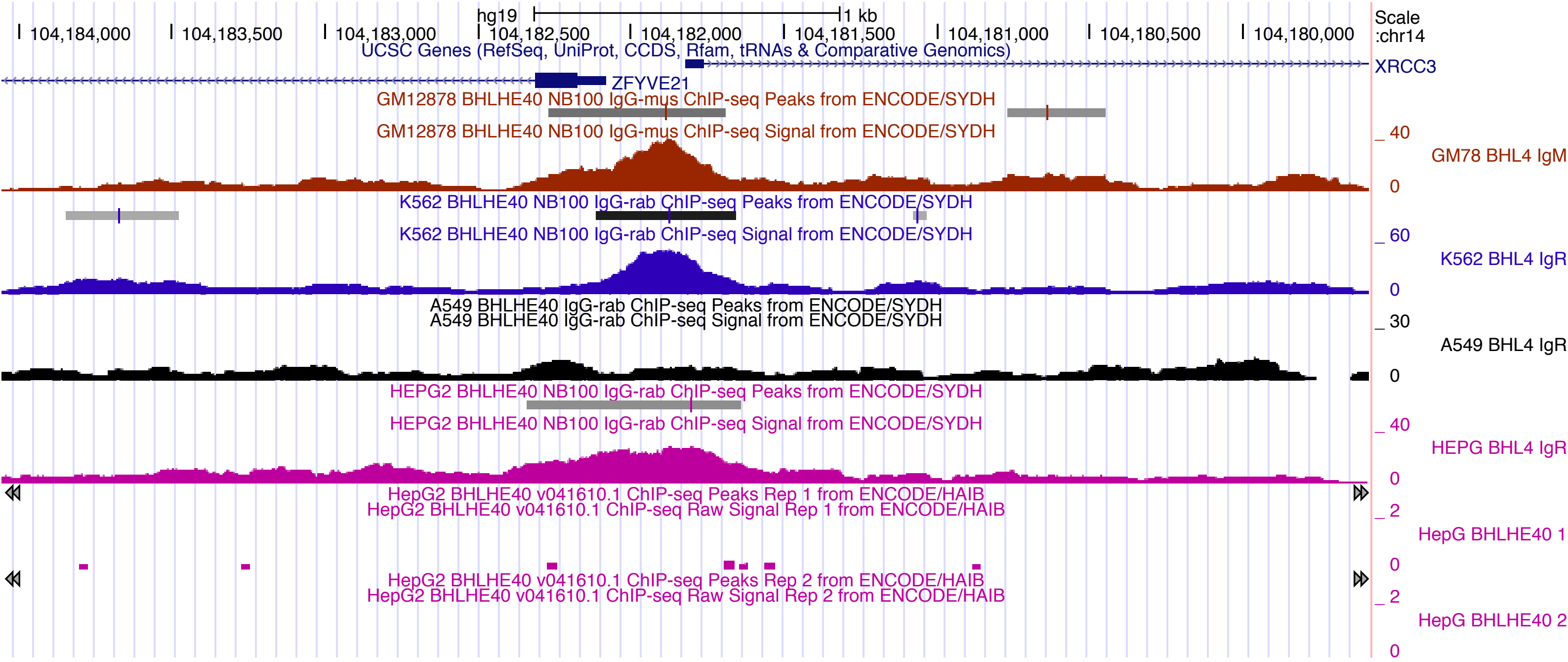

hg19
1 kb
Scale
:chr14
104,184,000
104,183,500
104,183,000
104,182,500
104,182,000
104,181,500
104,181,000
104,180,500
104,180,000
UCSC Genes (RefSeq, UniProt, CCDS, Rfam, tRNAs & Comparative Genomics)
ZFYVE21
XRCC3
GM12878 BHLHE40 NB100 IgG-mus ChIP-seq Peaks from ENCODE/SYDH
GM12878 BHLHE40 NB100 IgG-mus ChIP-seq Signal from ENCODE/SYDH
40
GM78 BHL4 IgM
0
K562 BHLHE40 NB100 IgG-rab ChIP-seq Peaks from ENCODE/SYDH
K562 BHLHE40 NB100 IgG-rab ChIP-seq Signal from ENCODE/SYDH
60
K562 BHL4 IgR
0
A549 BHLHE40 IgG-rab ChIP-seq Peaks from ENCODE/SYDH
A549 BHLHE40 IgG-rab ChIP-seq Signal from ENCODE/SYDH
30
A549 BHL4 IgR
0
HEPG2 BHLHE40 NB100 IgG-rab ChIP-seq Peaks from ENCODE/SYDH
HEPG2 BHLHE40 NB100 IgG-rab ChIP-seq Signal from ENCODE/SYDH
40
HEPG BHL4 IgR
0
HepG2 BHLHE40 v041610.1 ChIP-seq Peaks Rep 1 from ENCODE/HAIB
HepG2 BHLHE40 v041610.1 ChIP-seq Raw Signal Rep 1 from ENCODE/HAIB
2
HepG BHLHE40 1
0
HepG2 BHLHE40 v041610.1 ChIP-seq Peaks Rep 2 from ENCODE/HAIB
HepG2 BHLHE40 v041610.1 ChIP-seq Raw Signal Rep 2 from ENCODE/HAIB
2
HepG BHLHE40 2
0

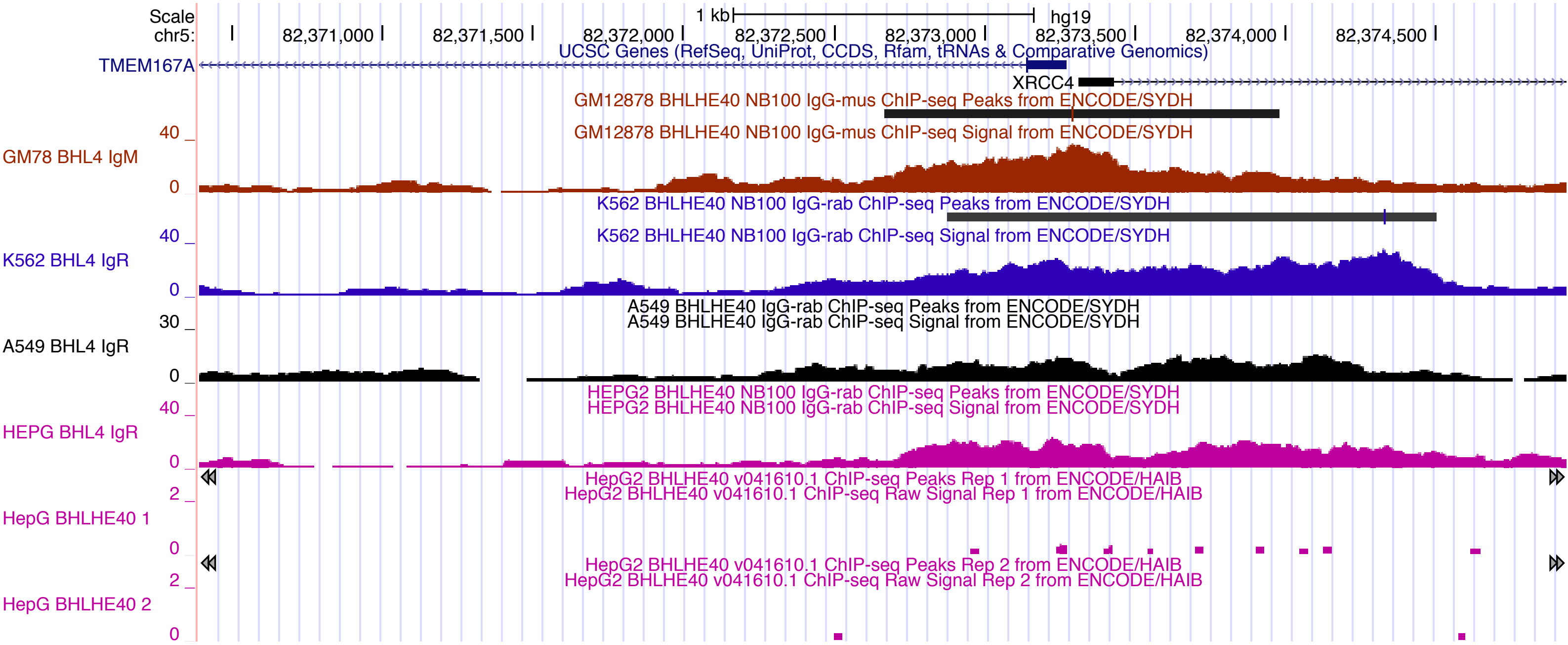
Scale
chr5:
1 kb
hg19
82,371,000
82,371,500
82,372,000
82,372,500
82,373,000
82,373,500
82,374,000
82,374,500
UCSC Genes (RefSeq, UniProt, CCDS, Rfam, tRNAs & Comparative Genomics)
TMEM167A
XRCC4
GM12878 BHLHE40 NB100 IgG-mus ChIP-seq Peaks from ENCODE/SYDH
GM12878 BHLHE40 NB100 IgG-mus ChIP-seq Signal from ENCODE/SYDH
GM78 BHL4 IgM
40
0
K562 BHLHE40 NB100 IgG-rab ChIP-seq Peaks from ENCODE/SYDH
K562 BHLHE40 NB100 IgG-rab ChIP-seq Signal from ENCODE/SYDH
K562 BHL4 IgR
40
0
A549 BHLHE40 IgG-rab ChIP-seq Peaks from ENCODE/SYDH
A549 BHLHE40 IgG-rab ChIP-seq Signal from ENCODE/SYDH
A549 BHL4 IgR
30
0
HEPG2 BHLHE40 NB100 IgG-rab ChIP-seq Peaks from ENCODE/SYDH
HEPG2 BHLHE40 NB100 IgG-rab ChIP-seq Signal from ENCODE/SYDH
HEPG BHL4 IgR
40
0
HepG2 BHLHE40 v041610.1 ChIP-seq Peaks Rep 1 from ENCODE/HAIB
HepG2 BHLHE40 v041610.1 ChIP-seq Raw Signal Rep 1 from ENCODE/HAIB
HepG BHLHE40 1
2
0
HepG2 BHLHE40 v041610.1 ChIP-seq Peaks Rep 2 from ENCODE/HAIB
HepG2 BHLHE40 v041610.1 ChIP-seq Raw Signal Rep 2 from ENCODE/HAIB
HepG BHLHE40 2
2
0

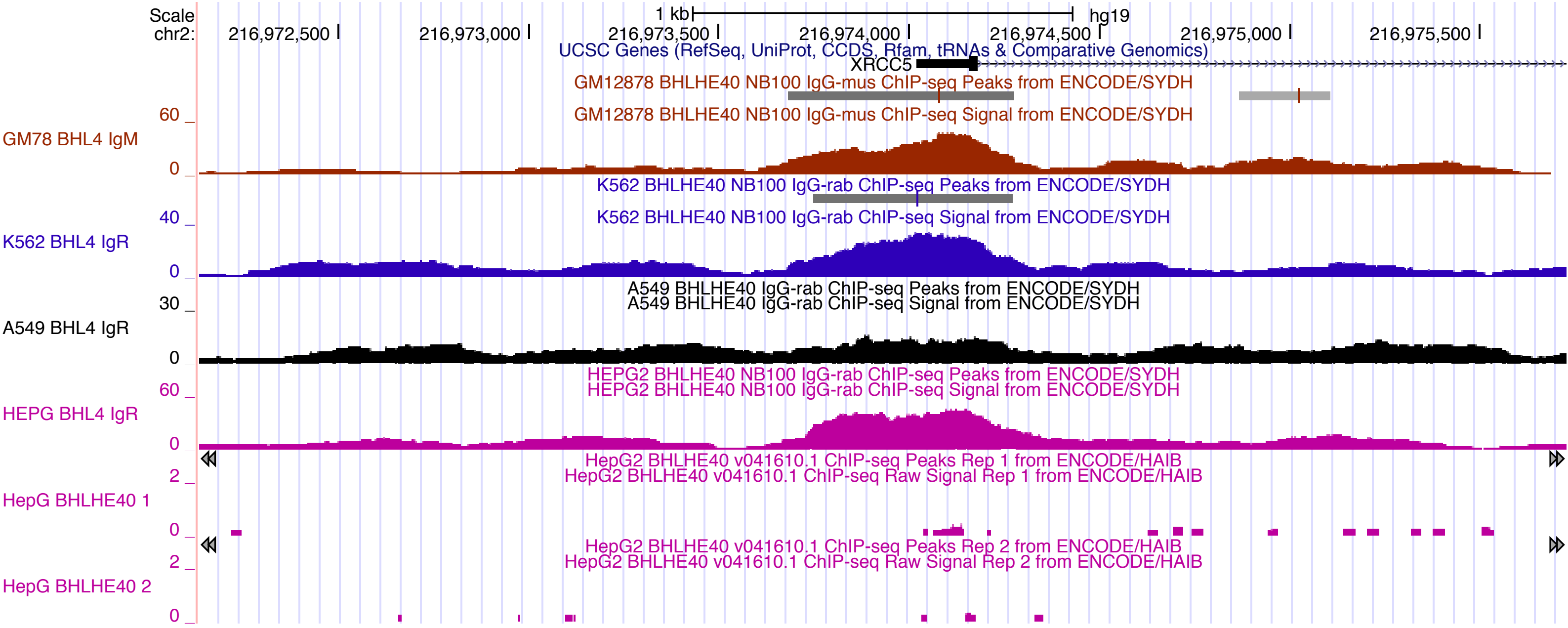
Scale
chr2:
1 kb
hg19
216,972,500
216,973,000
216,973,500
216,974,000
216,974,500
216,975,000
216,975,500
UCSC Genes (RefSeq, UniProt, CCDS, Rfam, tRNAs & Comparative Genomics)
XRCC5
GM12878 BHLHE40 NB100 IgG-mus ChIP-seq Peaks from ENCODE/SYDH
GM12878 BHLHE40 NB100 IgG-mus ChIP-seq Signal from ENCODE/SYDH
GM78 BHL4 IgM
60
0
K562 BHLHE40 NB100 IgG-rab ChIP-seq Peaks from ENCODE/SYDH
K562 BHLHE40 NB100 IgG-rab ChIP-seq Signal from ENCODE/SYDH
K562 BHL4 IgR
40
0
A549 BHLHE40 IgG-rab ChIP-seq Peaks from ENCODE/SYDH
A549 BHLHE40 IgG-rab ChIP-seq Signal from ENCODE/SYDH
A549 BHL4 IgR
30
0
HEPG2 BHLHE40 NB100 IgG-rab ChIP-seq Peaks from ENCODE/SYDH
HEPG2 BHLHE40 NB100 IgG-rab ChIP-seq Signal from ENCODE/SYDH
HEPG BHL4 IgR
60
0
HepG2 BHLHE40 v041610.1 ChIP-seq Peaks Rep 1 from ENCODE/HAIB
HepG2 BHLHE40 v041610.1 ChIP-seq Raw Signal Rep 1 from ENCODE/HAIB
HepG BHLHE40 1
2
0
HepG2 BHLHE40 v041610.1 ChIP-seq Peaks Rep 2 from ENCODE/HAIB
HepG2 BHLHE40 v041610.1 ChIP-seq Raw Signal Rep 2 from ENCODE/HAIB
HepG BHLHE40 2
2
0

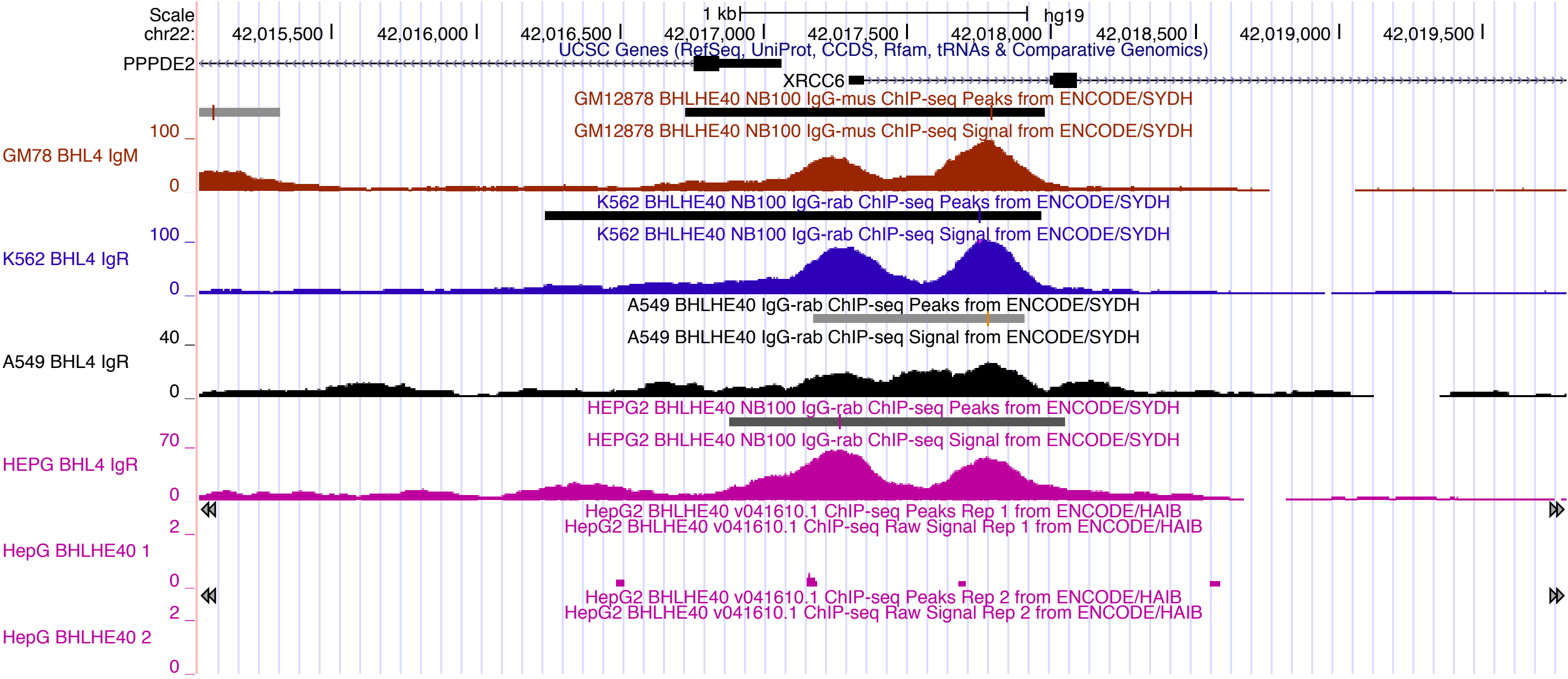

Scale
chr22:
1 kb
hg19
42,015,500
42,016,000
42,016,500
42,017,000
42,017,500
42,018,000
42,018,500
42,019,000
42,019,500
UCSC Genes (RefSeq, UniProt, CCDS, Rfam, tRNAs & Comparative Genomics)
PPPDE2
XRCC6
GM12878 BHLHE40 NB100 IgG-mus ChIP-seq Peaks from ENCODE/SYDH
GM12878 BHLHE40 NB100 IgG-mus ChIP-seq Signal from ENCODE/SYDH
GM78 BHL4 IgM
100
0
K562 BHLHE40 NB100 IgG-rab ChIP-seq Peaks from ENCODE/SYDH
K562 BHLHE40 NB100 IgG-rab ChIP-seq Signal from ENCODE/SYDH
K562 BHL4 IgR
100
0
A549 BHLHE40 IgG-rab ChIP-seq Peaks from ENCODE/SYDH
A549 BHLHE40 IgG-rab ChIP-seq Signal from ENCODE/SYDH
A549 BHL4 IgR
40
0
HEPG2 BHLHE40 NB100 IgG-rab ChIP-seq Peaks from ENCODE/SYDH
HEPG2 BHLHE40 NB100 IgG-rab ChIP-seq Signal from ENCODE/SYDH
HEPG BHL4 IgR
70
0
HepG2 BHLHE40 v041610.1 ChIP-seq Peaks Rep 1 from ENCODE/HAIB
HepG2 BHLHE40 v041610.1 ChIP-seq Raw Signal Rep 1 from ENCODE/HAIB
HepG BHLHE40 1
2
0
HepG2 BHLHE40 v041610.1 ChIP-seq Peaks Rep 2 from ENCODE/HAIB
HepG2 BHLHE40 v041610.1 ChIP-seq Raw Signal Rep 2 from ENCODE/HAIB
HepG BHLHE40 2
2
0
